# Supplementary figures and images for: Glutamine catabolism supports amino acid biosynthesis and suppresses the integrated stress response to promote photoreceptor survival (part 1 of 4)
Source: eLife. 2025 May 21;13:RP100747. doi: 10.7554/eLife.100747 (PMC12094702; doi:10.7554/eLife.100747)

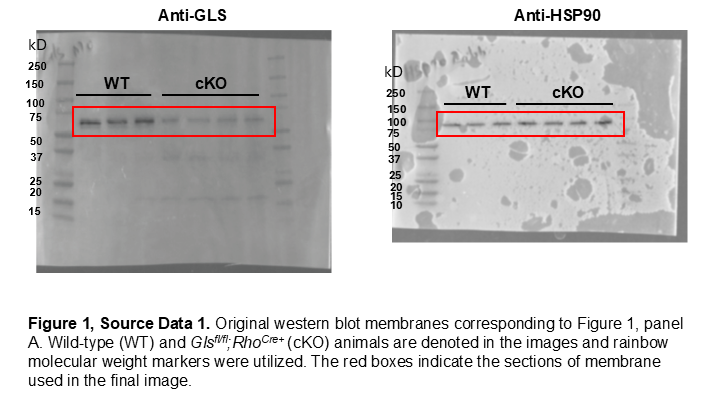

Supplement: Figure 1—source data 1. — Wild-type (WT) and Glsfl/fl;RhoCre+ (cKO) animals are denoted in the images and rainbow molecular weight markers were utilized. The red boxes indicate the sections of membrane used in the final image. [file elife-100747-fig1-data1.zip › Figure 1 - Source Data 1 (annotated western file)/Figure 1 - Source Data 1.tif]

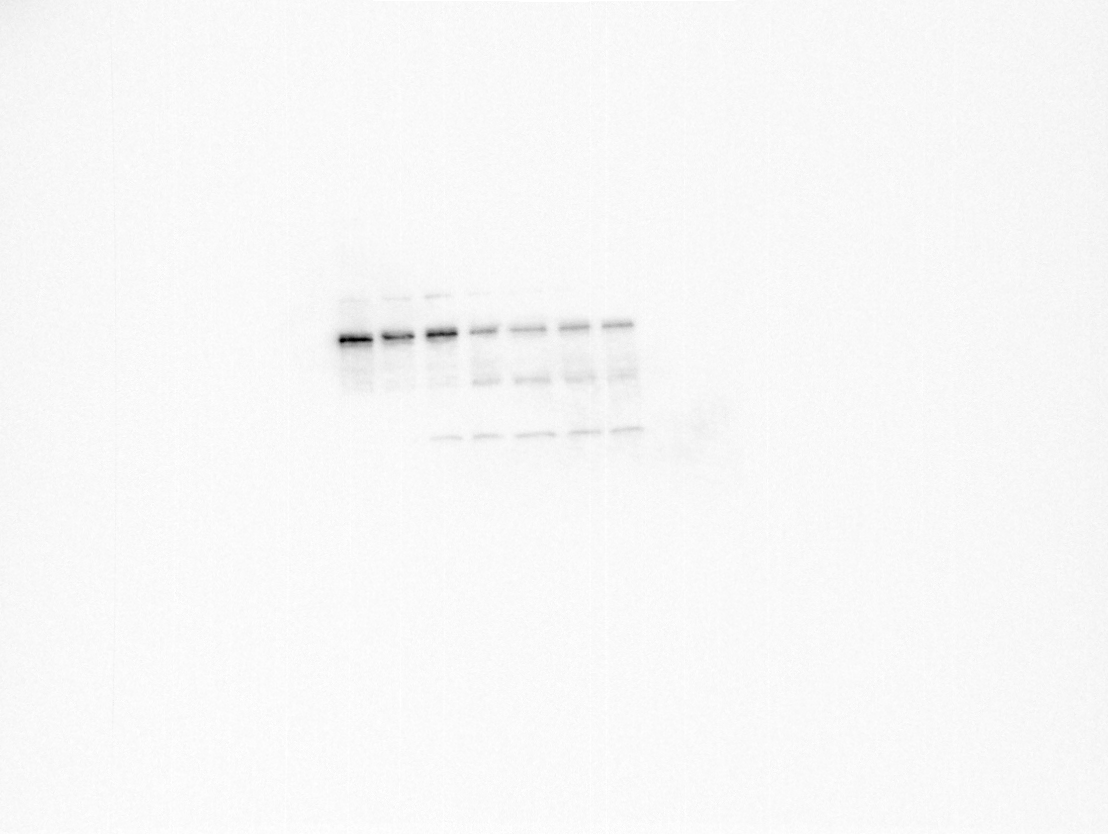

Supplement: Figure 1—source data 2. [file elife-100747-fig1-data2.zip › Figure 1 - Source Data 2 (original western files)/gls-mo-ptg/2022-1118-154646.tif]

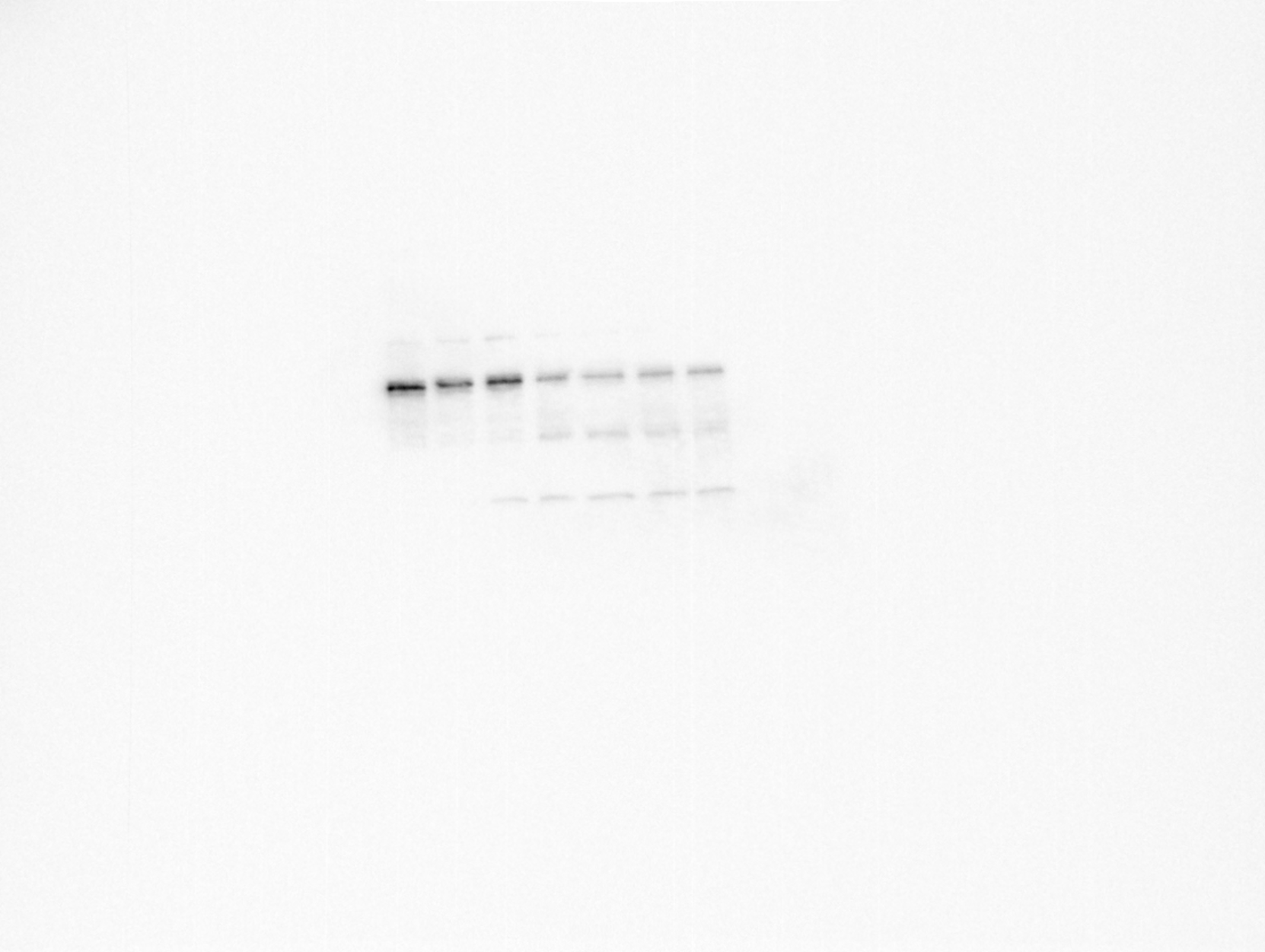

Supplement: Figure 1—source data 2. [file elife-100747-fig1-data2.zip › Figure 1 - Source Data 2 (original western files)/gls-mo-ptg/2022-1118-154646_pub.tif]

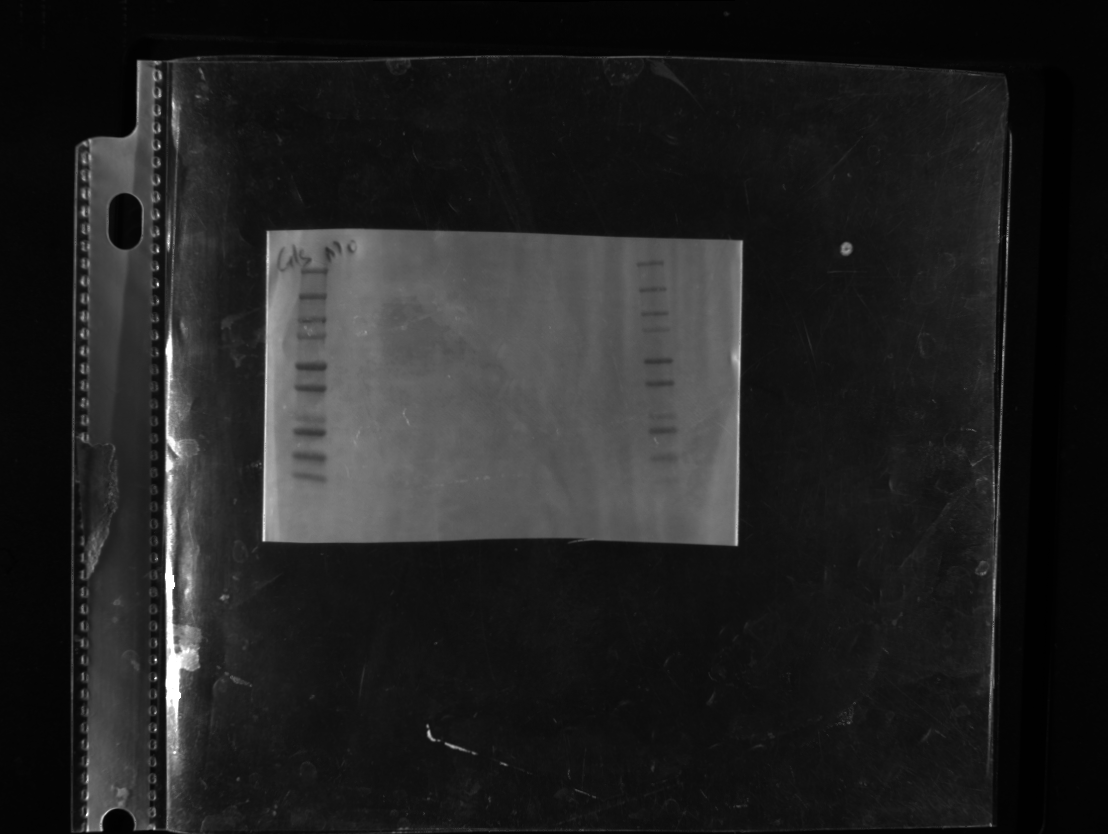

Supplement: Figure 1—source data 2. [file elife-100747-fig1-data2.zip › Figure 1 - Source Data 2 (original western files)/gls-mo-ptg/2022-1118-154648.tif]

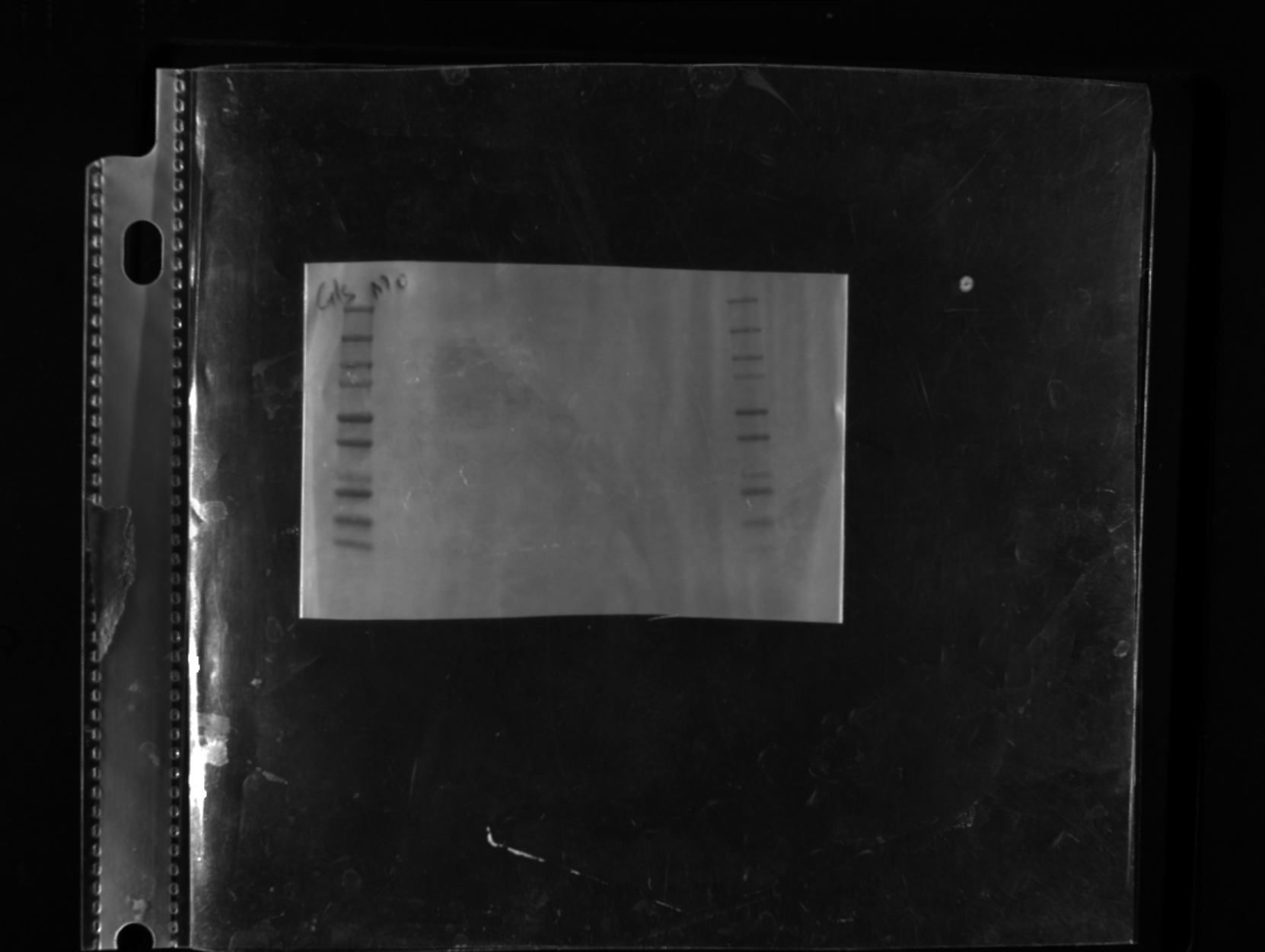

Supplement: Figure 1—source data 2. [file elife-100747-fig1-data2.zip › Figure 1 - Source Data 2 (original western files)/gls-mo-ptg/2022-1118-154648_pub.tif]

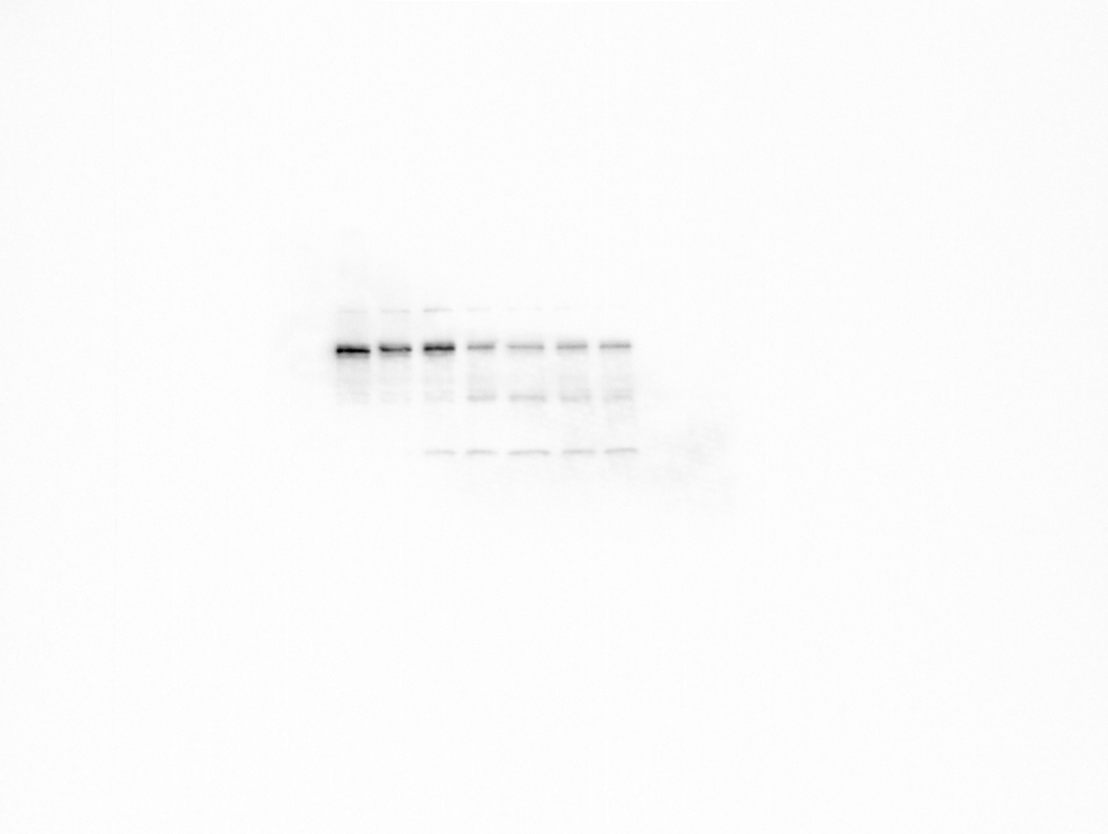

Supplement: Figure 1—source data 2. [file elife-100747-fig1-data2.zip › Figure 1 - Source Data 2 (original western files)/gls-mo-ptg/2022-1118-154649.tif]

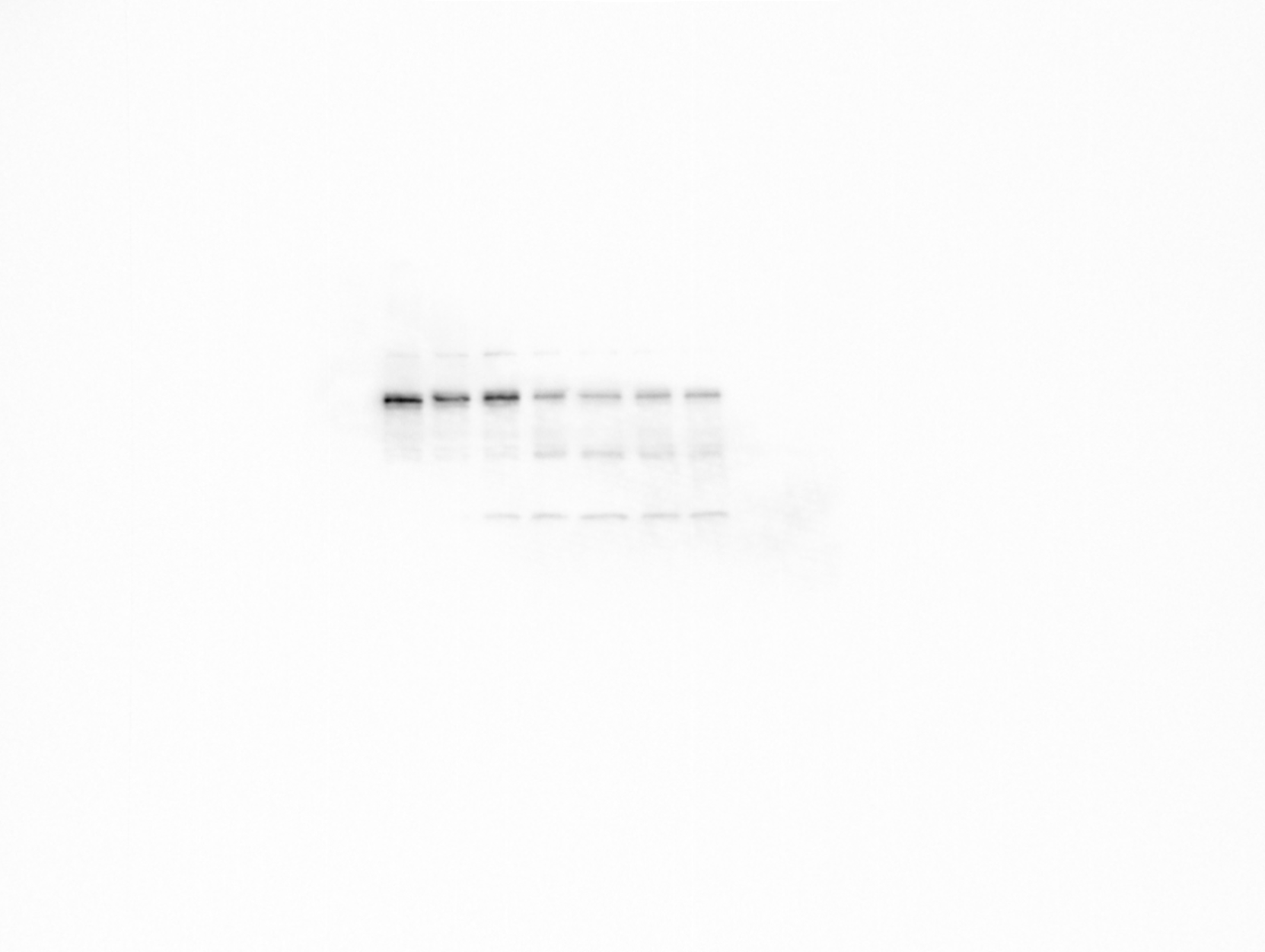

Supplement: Figure 1—source data 2. [file elife-100747-fig1-data2.zip › Figure 1 - Source Data 2 (original western files)/gls-mo-ptg/2022-1118-154649_pub.tif]

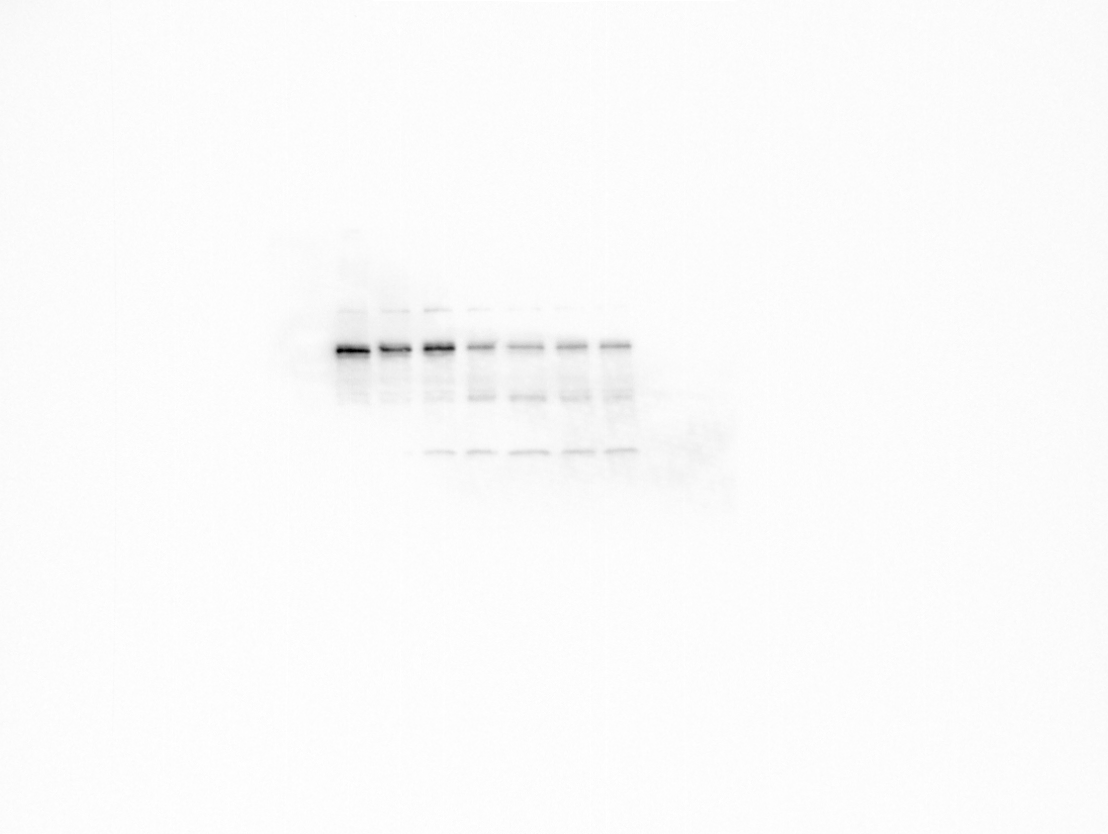

Supplement: Figure 1—source data 2. [file elife-100747-fig1-data2.zip › Figure 1 - Source Data 2 (original western files)/gls-mo-ptg/S1F1-1118-154650.tif]

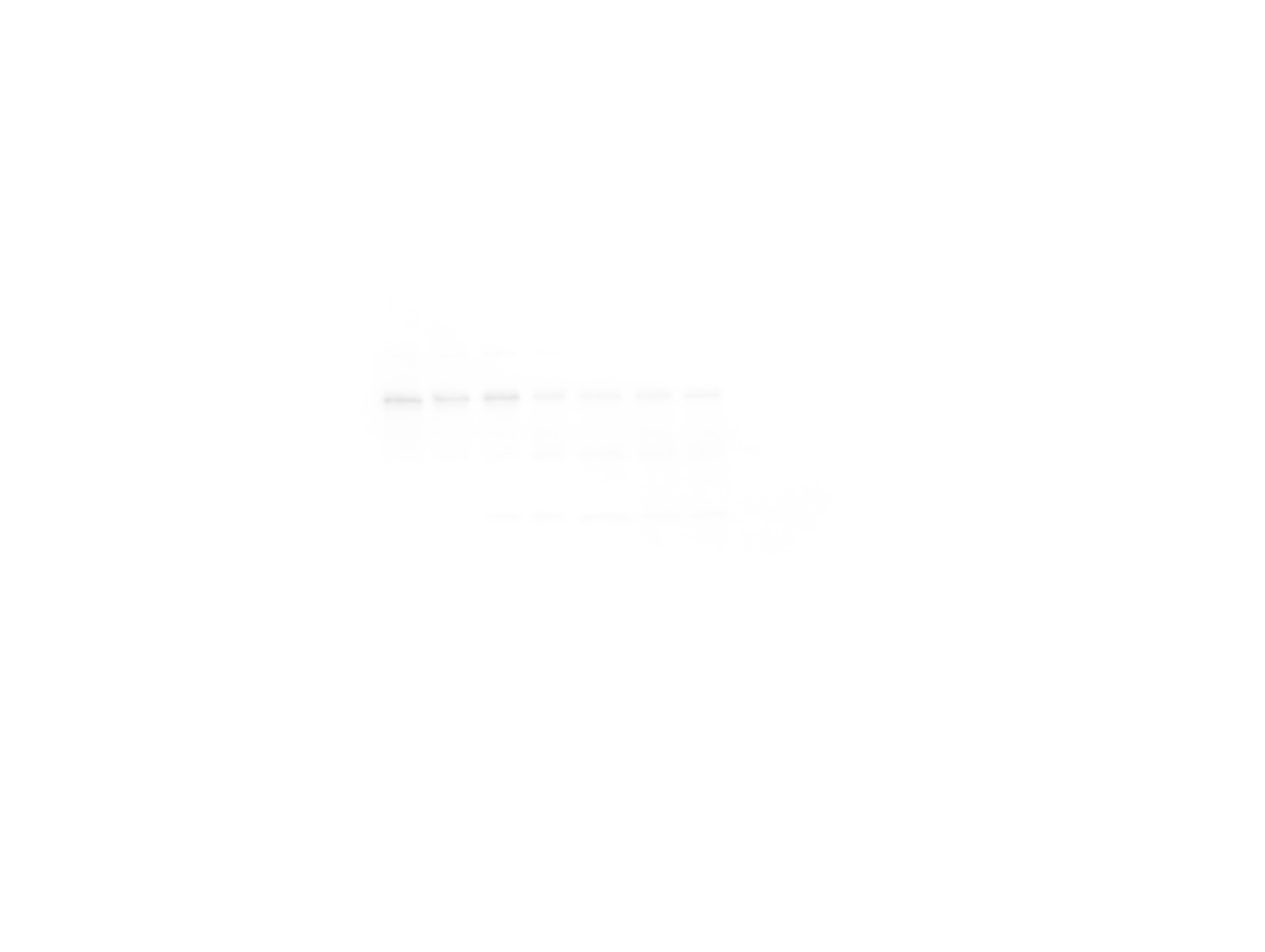

Supplement: Figure 1—source data 2. [file elife-100747-fig1-data2.zip › Figure 1 - Source Data 2 (original western files)/gls-mo-ptg/S1F1-1118-154650_pub.tif]

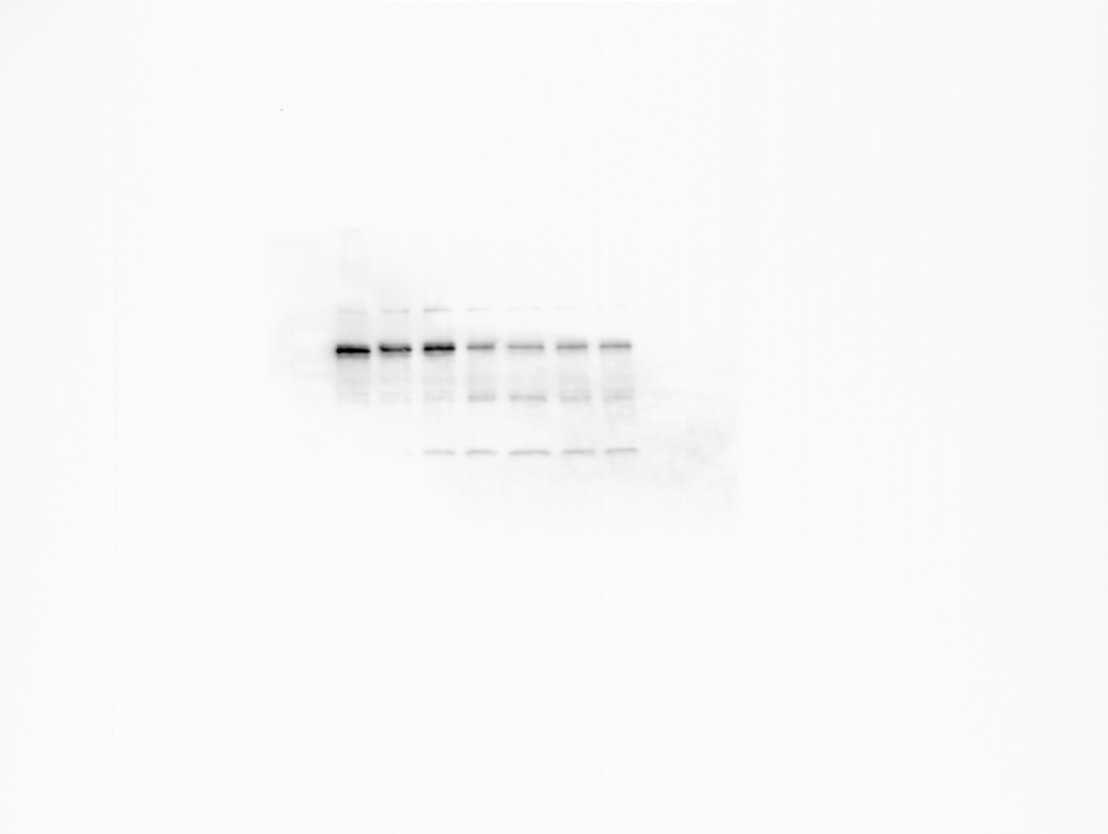

Supplement: Figure 1—source data 2. [file elife-100747-fig1-data2.zip › Figure 1 - Source Data 2 (original western files)/gls-mo-ptg/S1F10-1118-154705.tif]

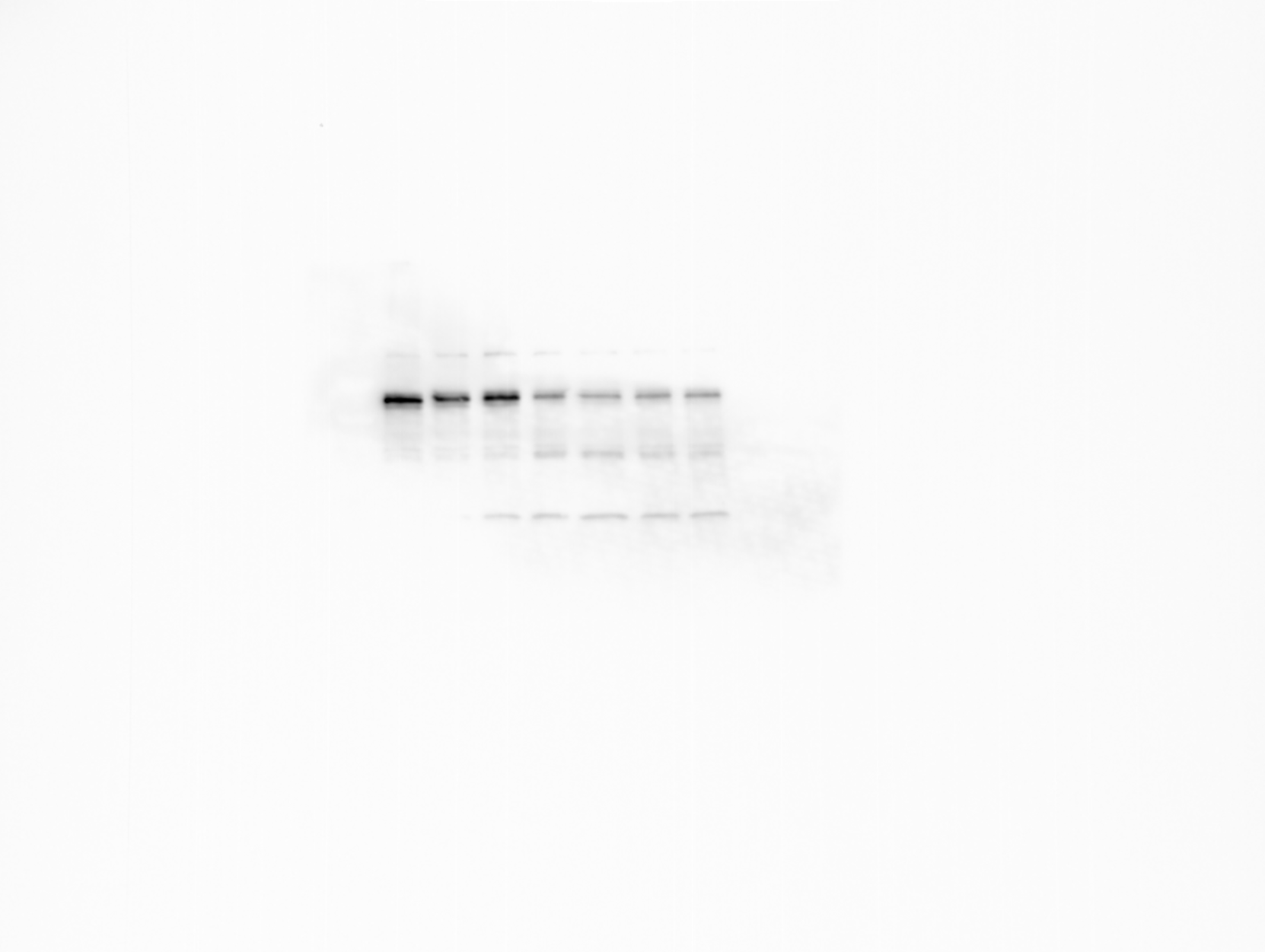

Supplement: Figure 1—source data 2. [file elife-100747-fig1-data2.zip › Figure 1 - Source Data 2 (original western files)/gls-mo-ptg/S1F10-1118-154705_pub.tif]

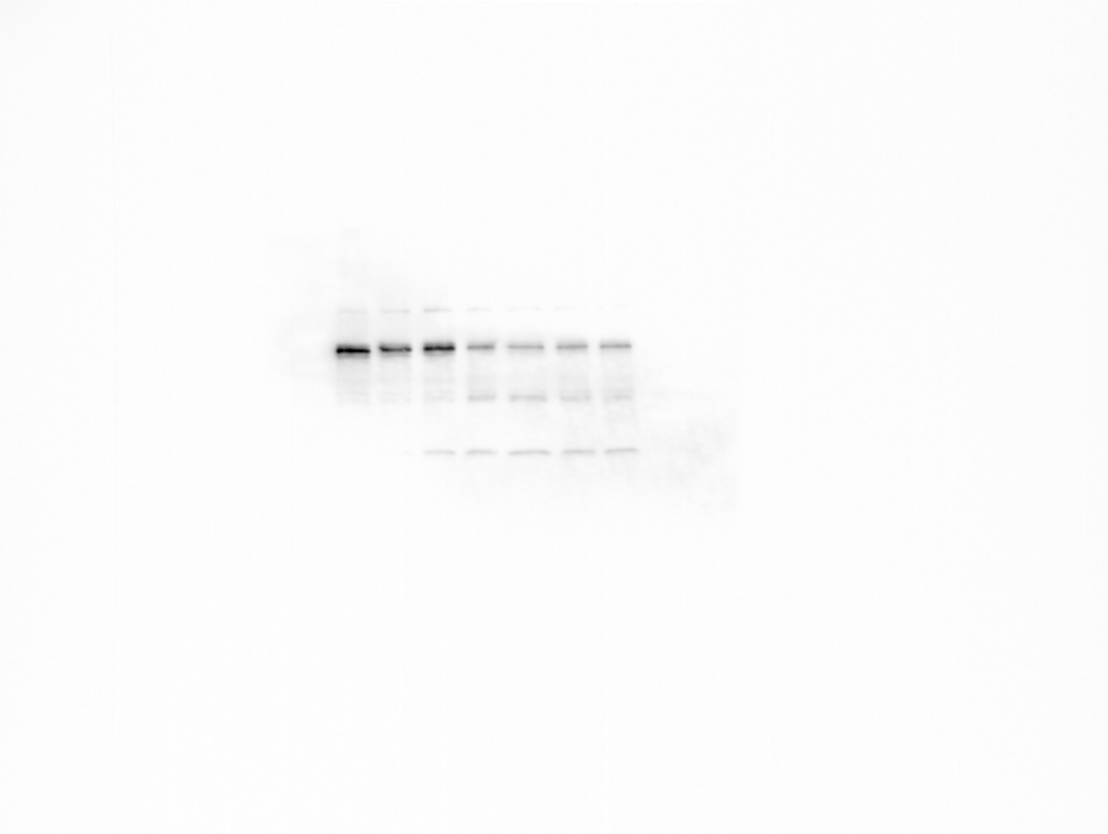

Supplement: Figure 1—source data 2. [file elife-100747-fig1-data2.zip › Figure 1 - Source Data 2 (original western files)/gls-mo-ptg/S1F2-1118-154652.tif]

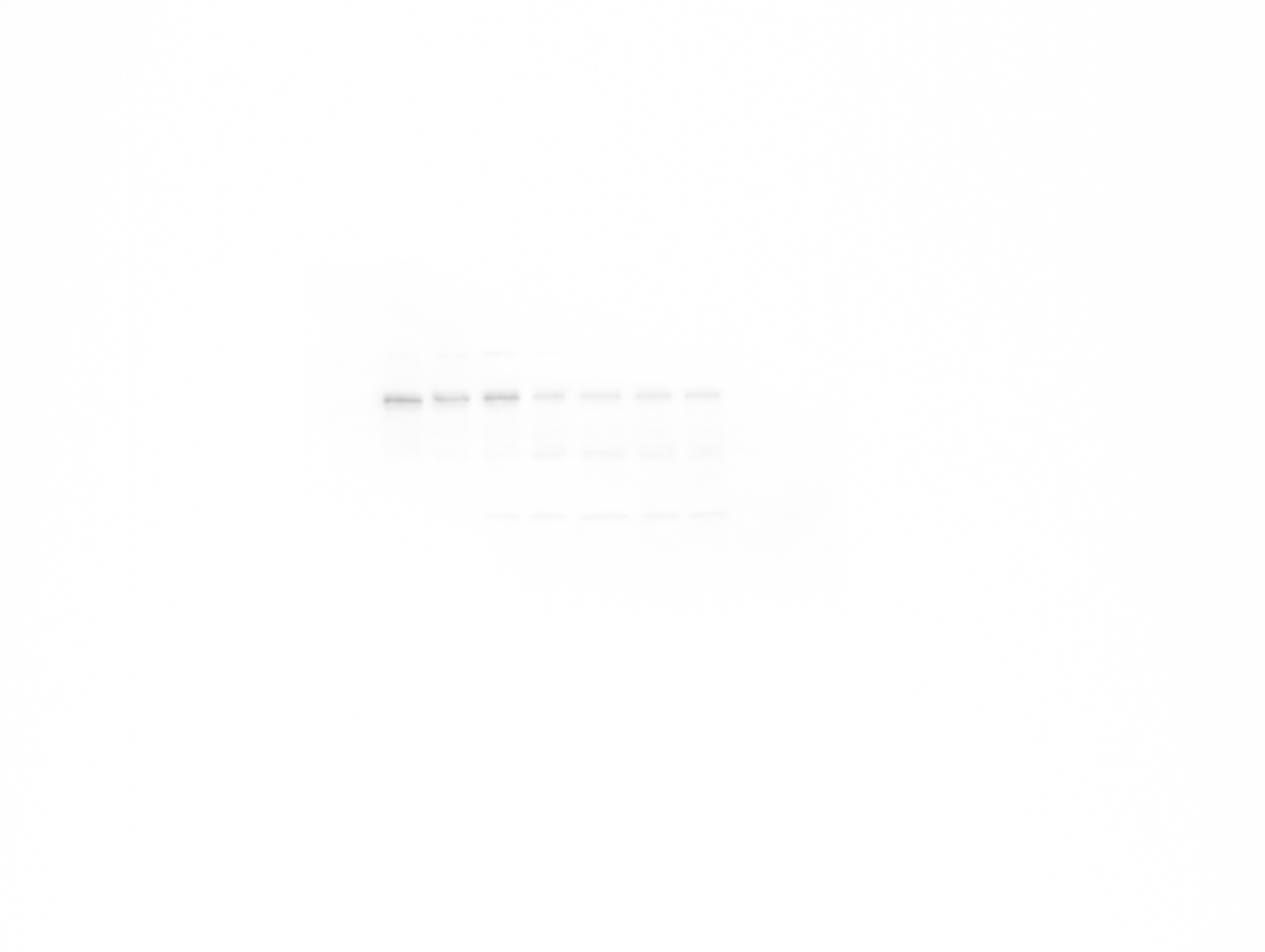

Supplement: Figure 1—source data 2. [file elife-100747-fig1-data2.zip › Figure 1 - Source Data 2 (original western files)/gls-mo-ptg/S1F2-1118-154652_pub.tif]

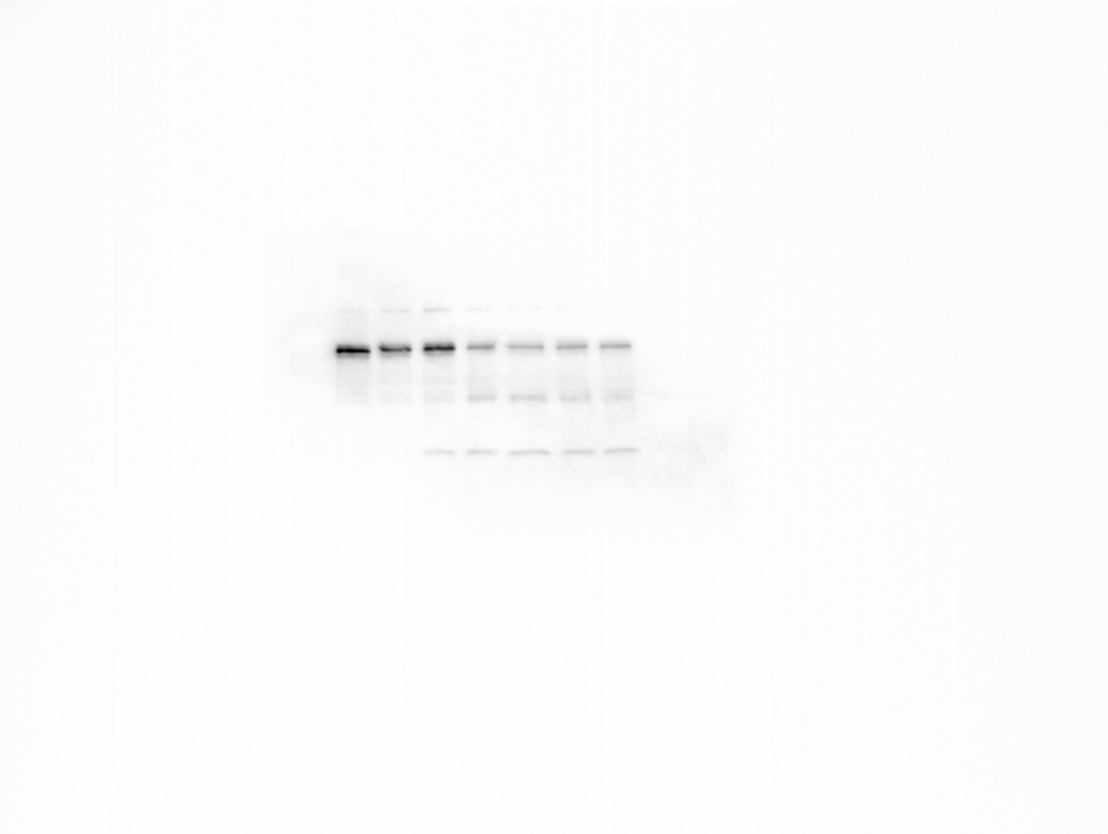

Supplement: Figure 1—source data 2. [file elife-100747-fig1-data2.zip › Figure 1 - Source Data 2 (original western files)/gls-mo-ptg/S1F3-1118-154654.tif]

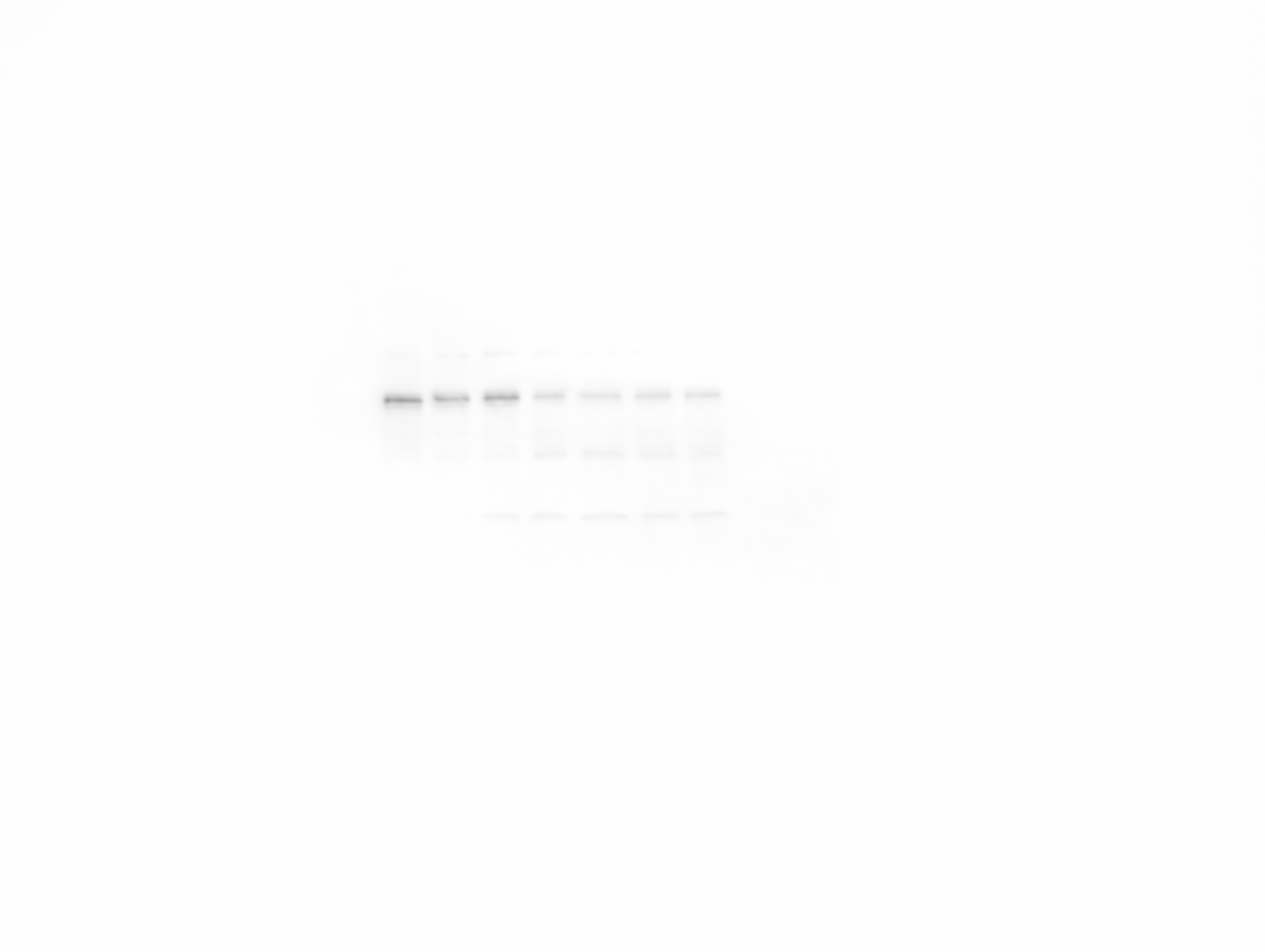

Supplement: Figure 1—source data 2. [file elife-100747-fig1-data2.zip › Figure 1 - Source Data 2 (original western files)/gls-mo-ptg/S1F3-1118-154654_pub.tif]

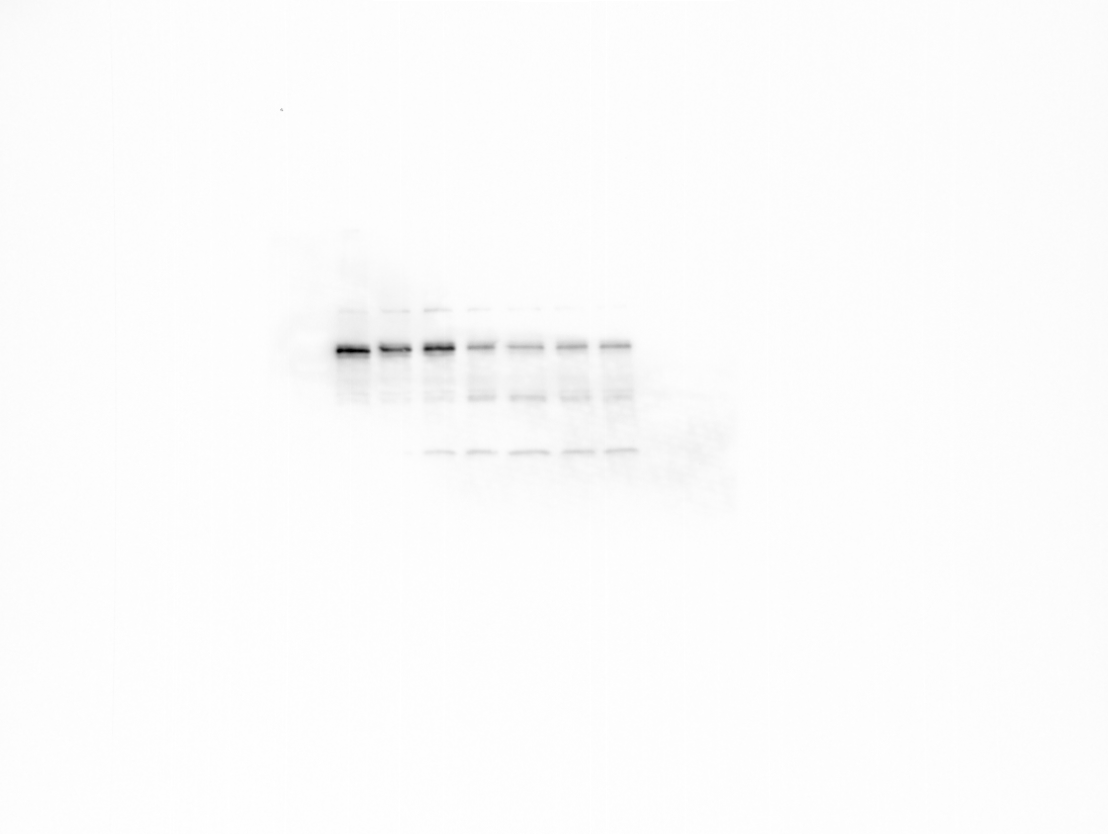

Supplement: Figure 1—source data 2. [file elife-100747-fig1-data2.zip › Figure 1 - Source Data 2 (original western files)/gls-mo-ptg/S1F4-1118-154655.tif]

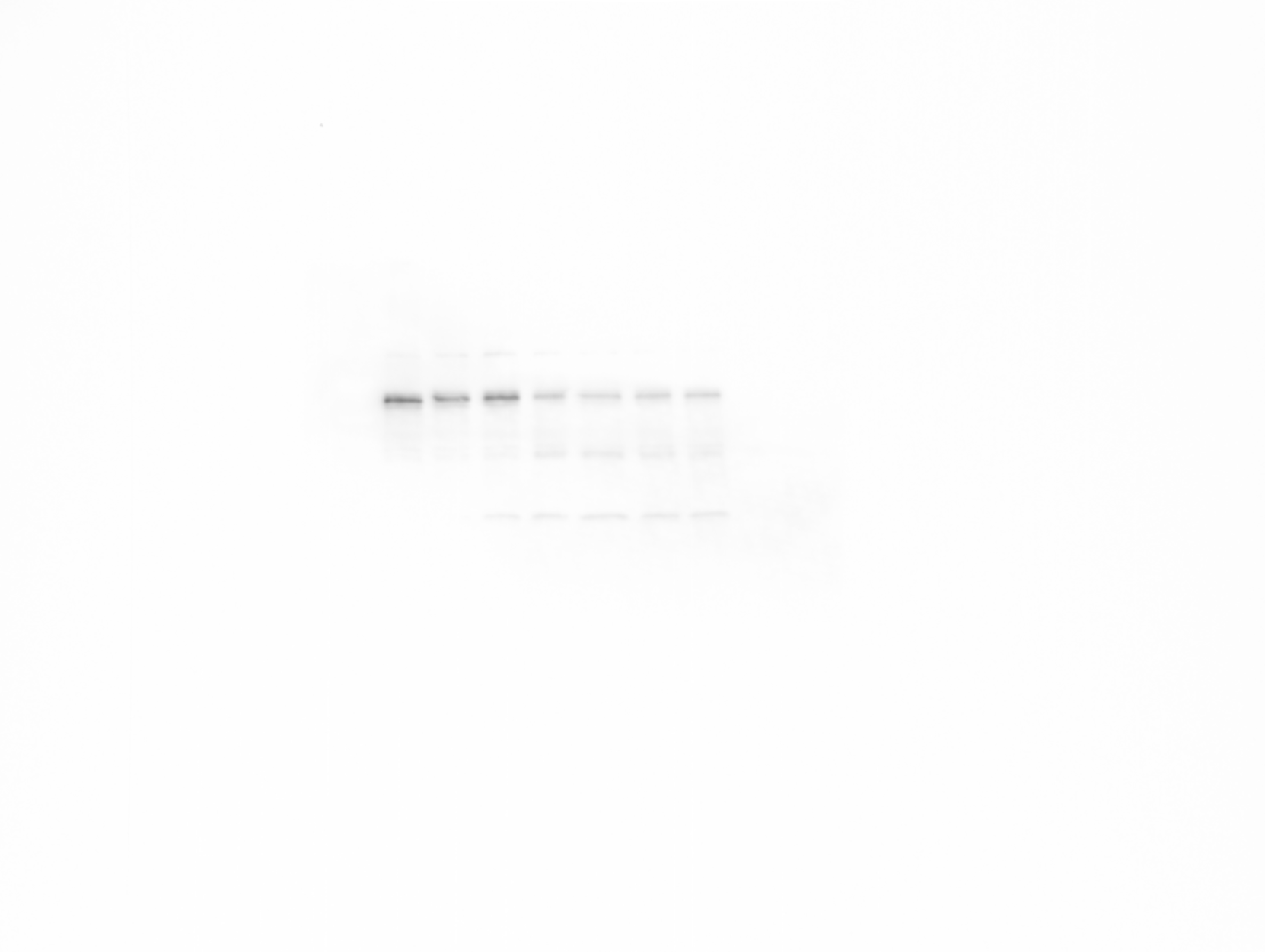

Supplement: Figure 1—source data 2. [file elife-100747-fig1-data2.zip › Figure 1 - Source Data 2 (original western files)/gls-mo-ptg/S1F4-1118-154655_pub.tif]

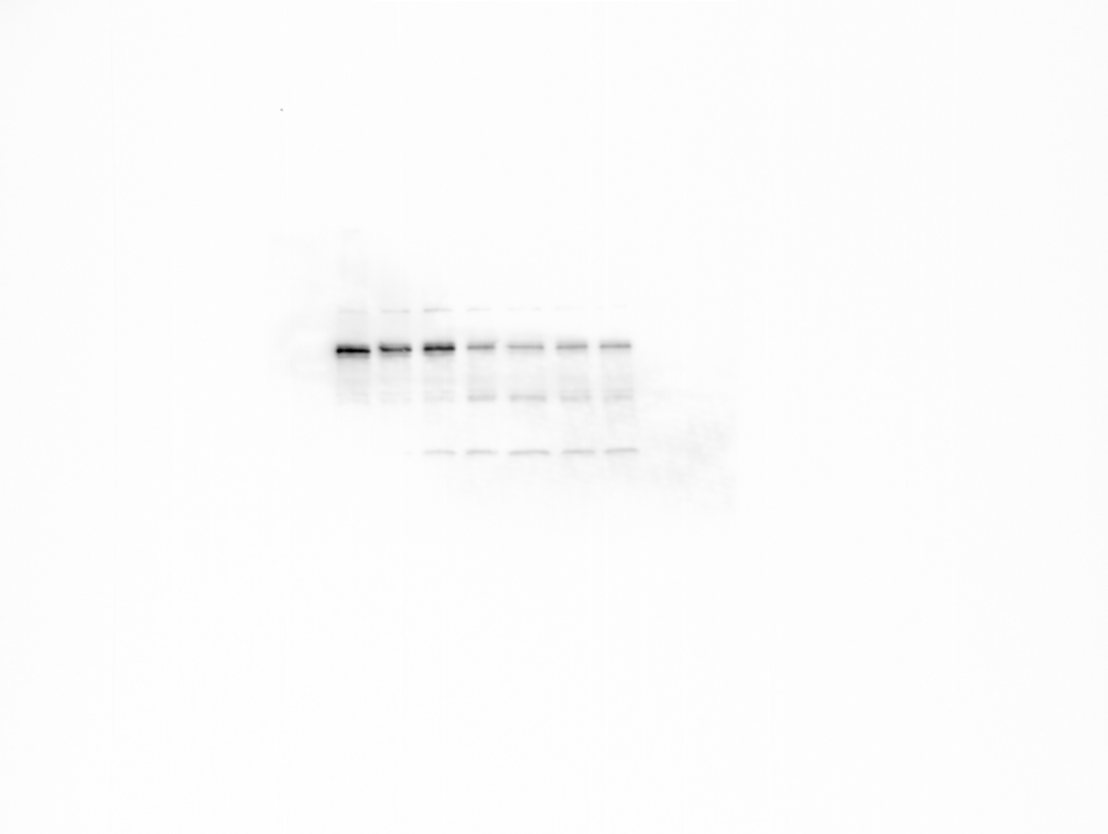

Supplement: Figure 1—source data 2. [file elife-100747-fig1-data2.zip › Figure 1 - Source Data 2 (original western files)/gls-mo-ptg/S1F5-1118-154657.tif]

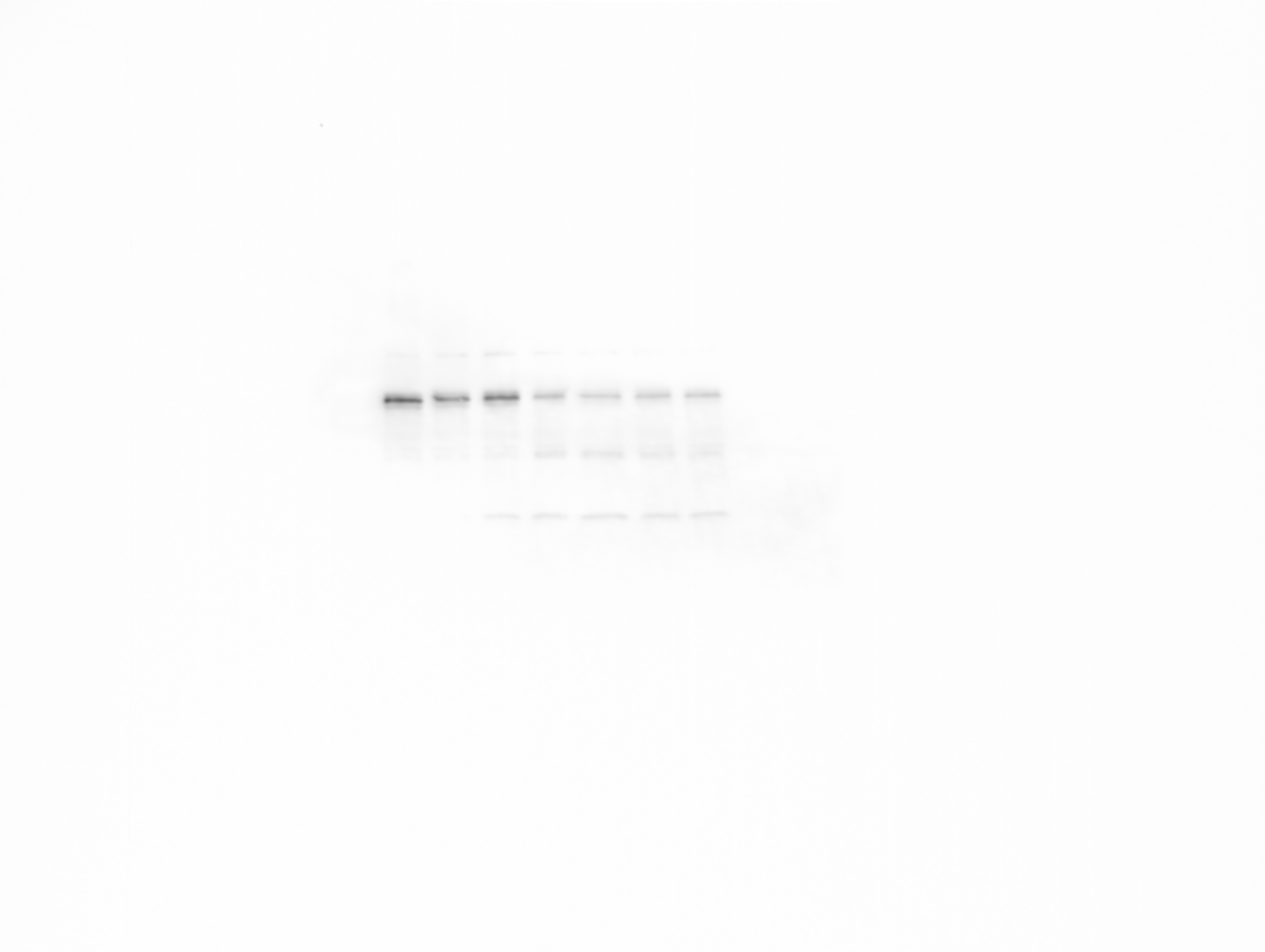

Supplement: Figure 1—source data 2. [file elife-100747-fig1-data2.zip › Figure 1 - Source Data 2 (original western files)/gls-mo-ptg/S1F5-1118-154657_pub.tif]

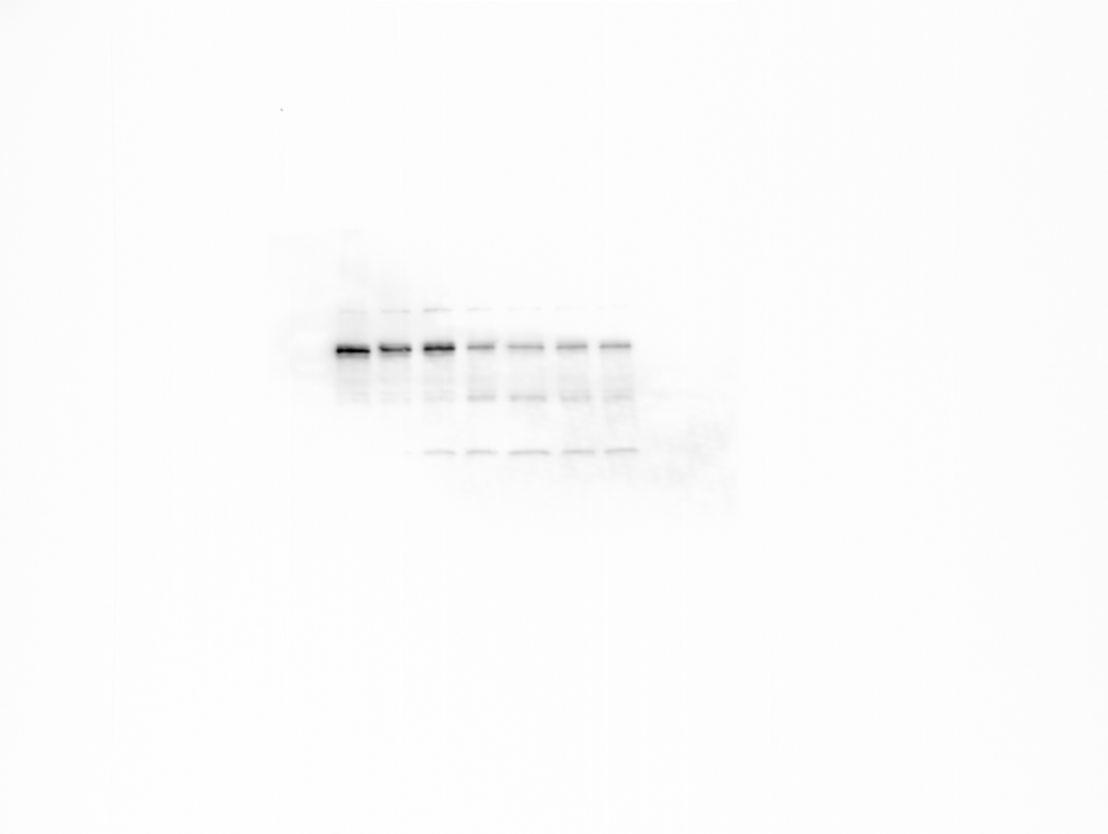

Supplement: Figure 1—source data 2. [file elife-100747-fig1-data2.zip › Figure 1 - Source Data 2 (original western files)/gls-mo-ptg/S1F6-1118-154659.tif]

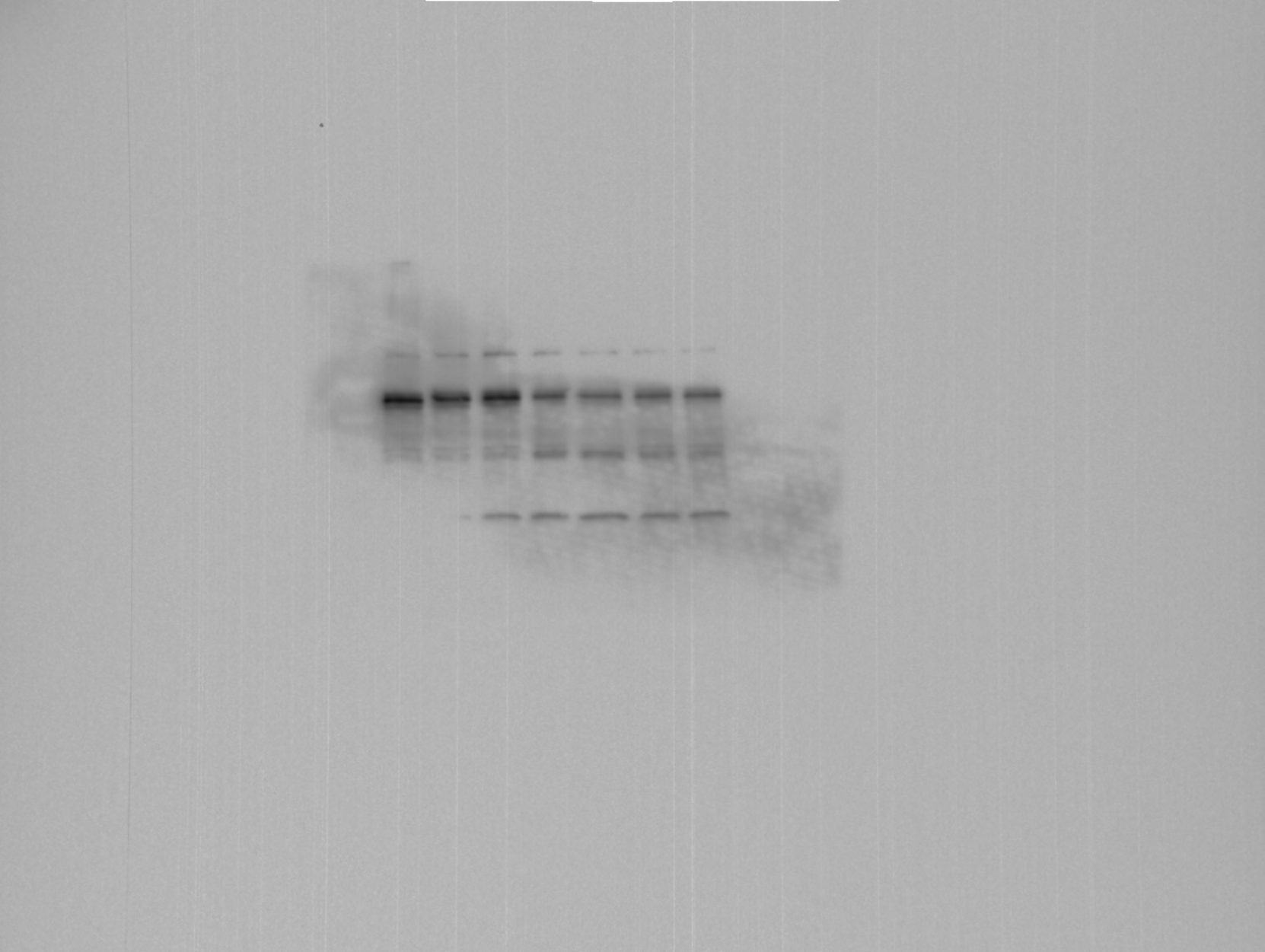

Supplement: Figure 1—source data 2. [file elife-100747-fig1-data2.zip › Figure 1 - Source Data 2 (original western files)/gls-mo-ptg/S1F6-1118-154659_pub.tif]

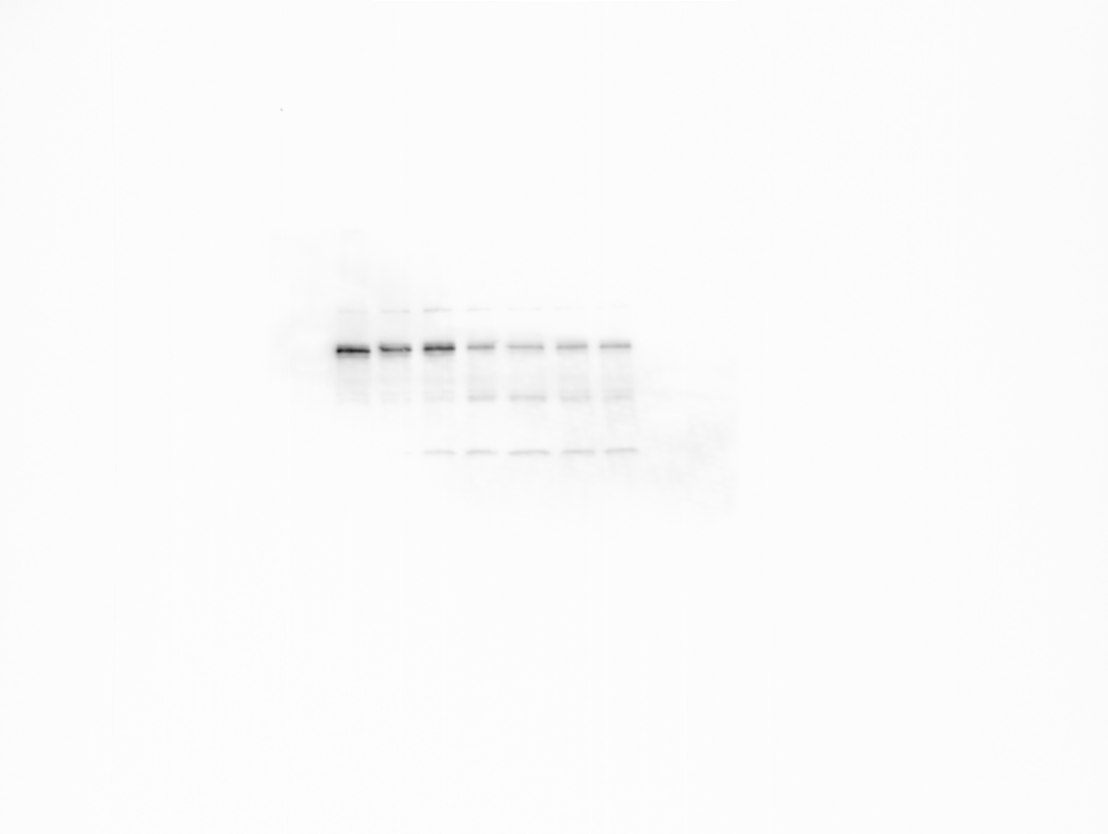

Supplement: Figure 1—source data 2. [file elife-100747-fig1-data2.zip › Figure 1 - Source Data 2 (original western files)/gls-mo-ptg/S1F7-1118-154700.tif]

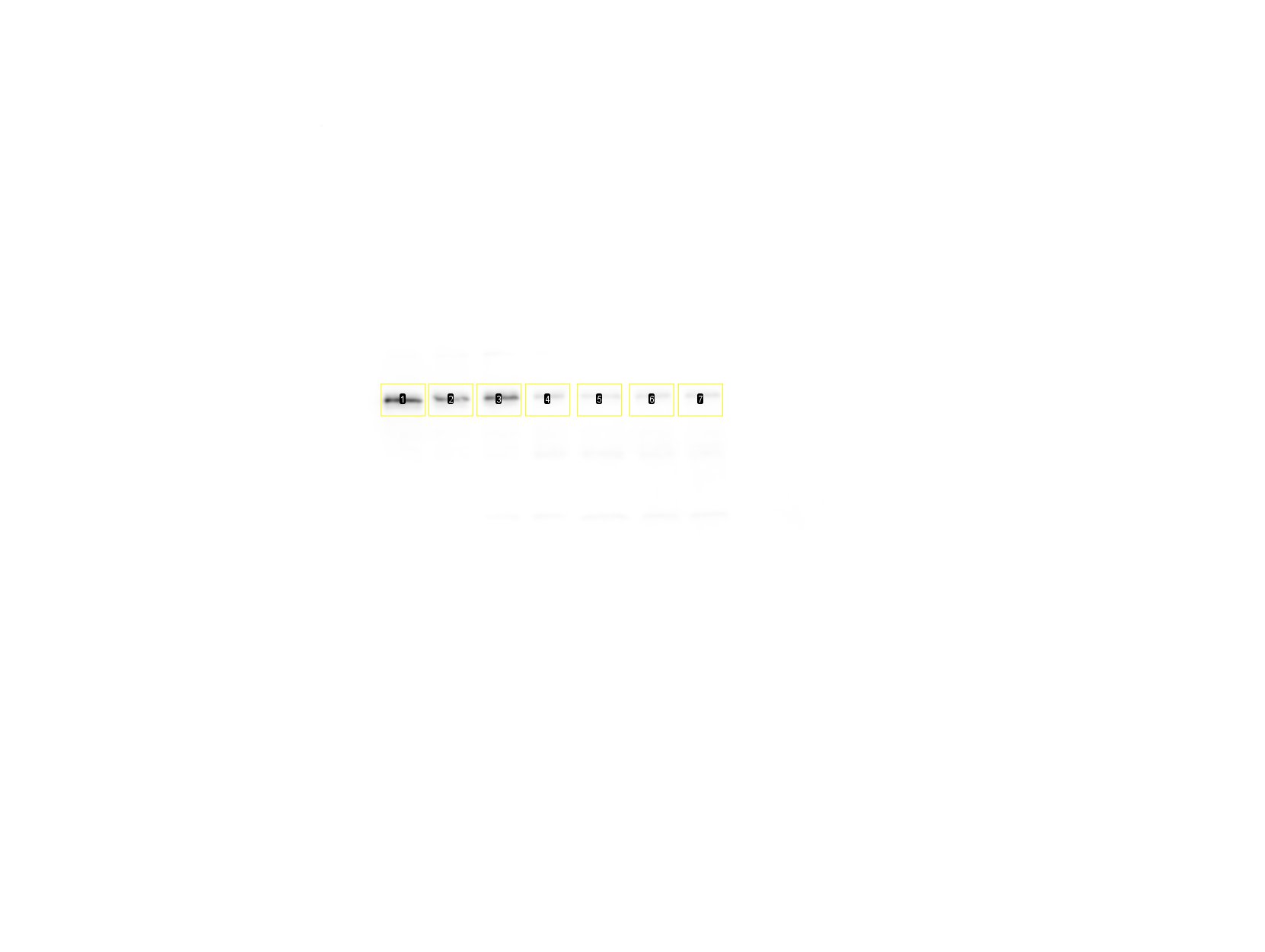

Supplement: Figure 1—source data 2. [file elife-100747-fig1-data2.zip › Figure 1 - Source Data 2 (original western files)/gls-mo-ptg/S1F7-1118-154700_pub.jpg]

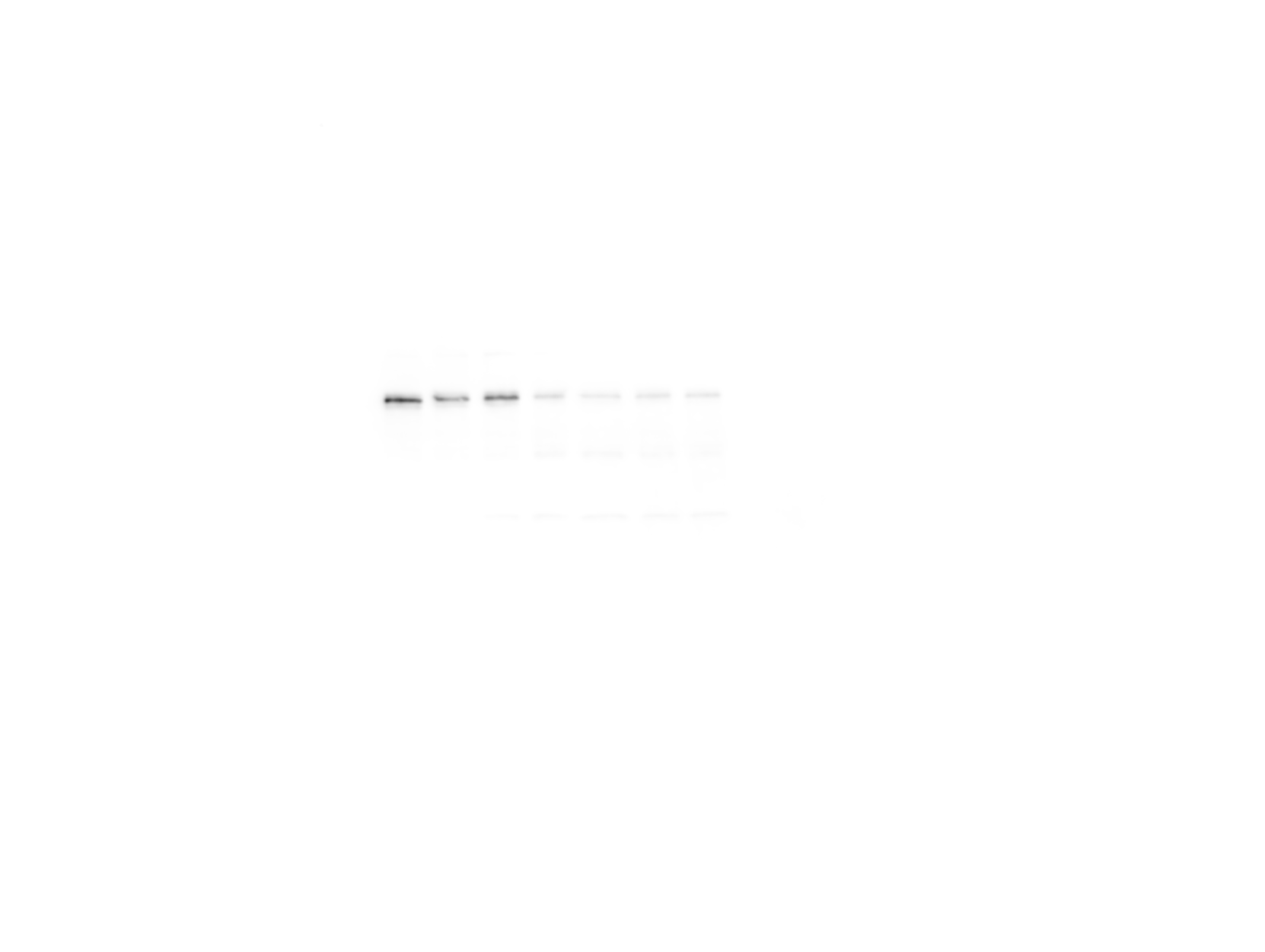

Supplement: Figure 1—source data 2. [file elife-100747-fig1-data2.zip › Figure 1 - Source Data 2 (original western files)/gls-mo-ptg/S1F7-1118-154700_pub.tif]

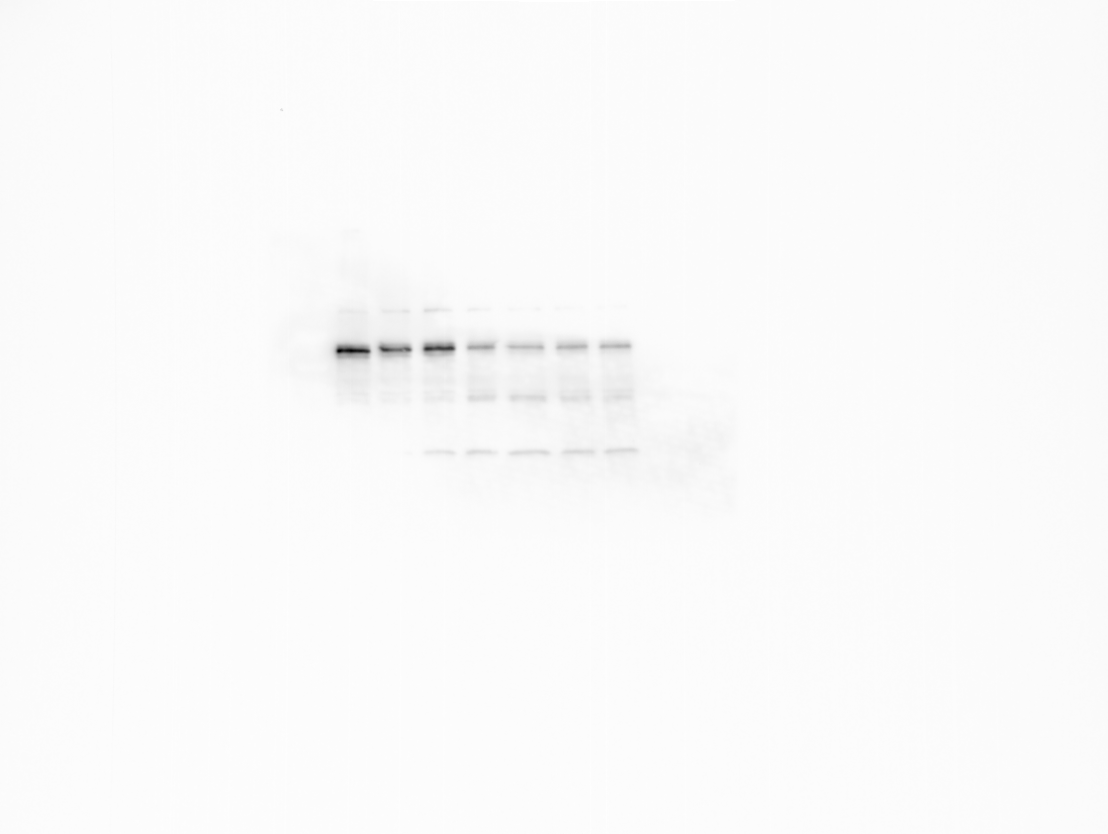

Supplement: Figure 1—source data 2. [file elife-100747-fig1-data2.zip › Figure 1 - Source Data 2 (original western files)/gls-mo-ptg/S1F8-1118-154702.tif]

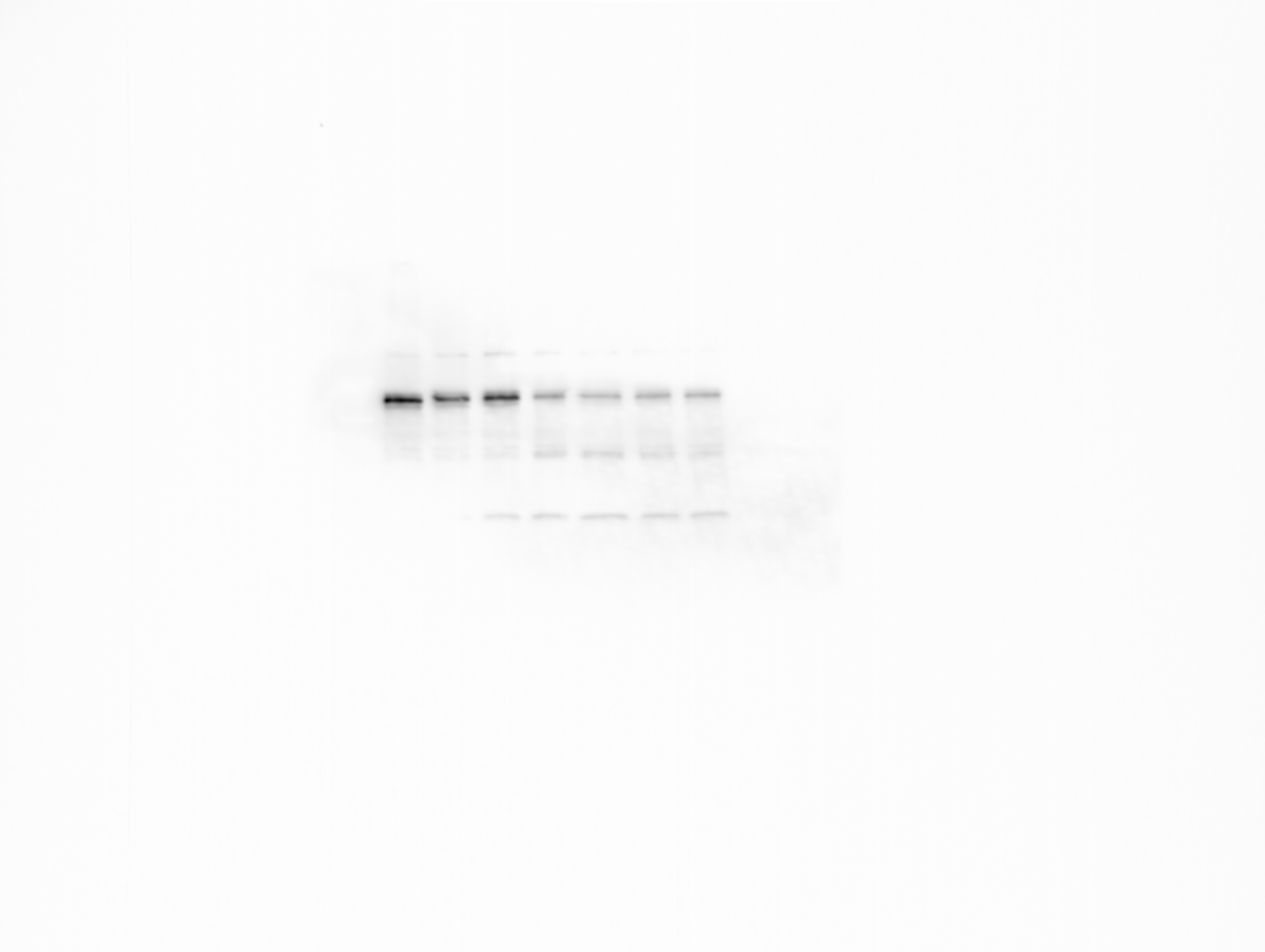

Supplement: Figure 1—source data 2. [file elife-100747-fig1-data2.zip › Figure 1 - Source Data 2 (original western files)/gls-mo-ptg/S1F8-1118-154702_pub.tif]

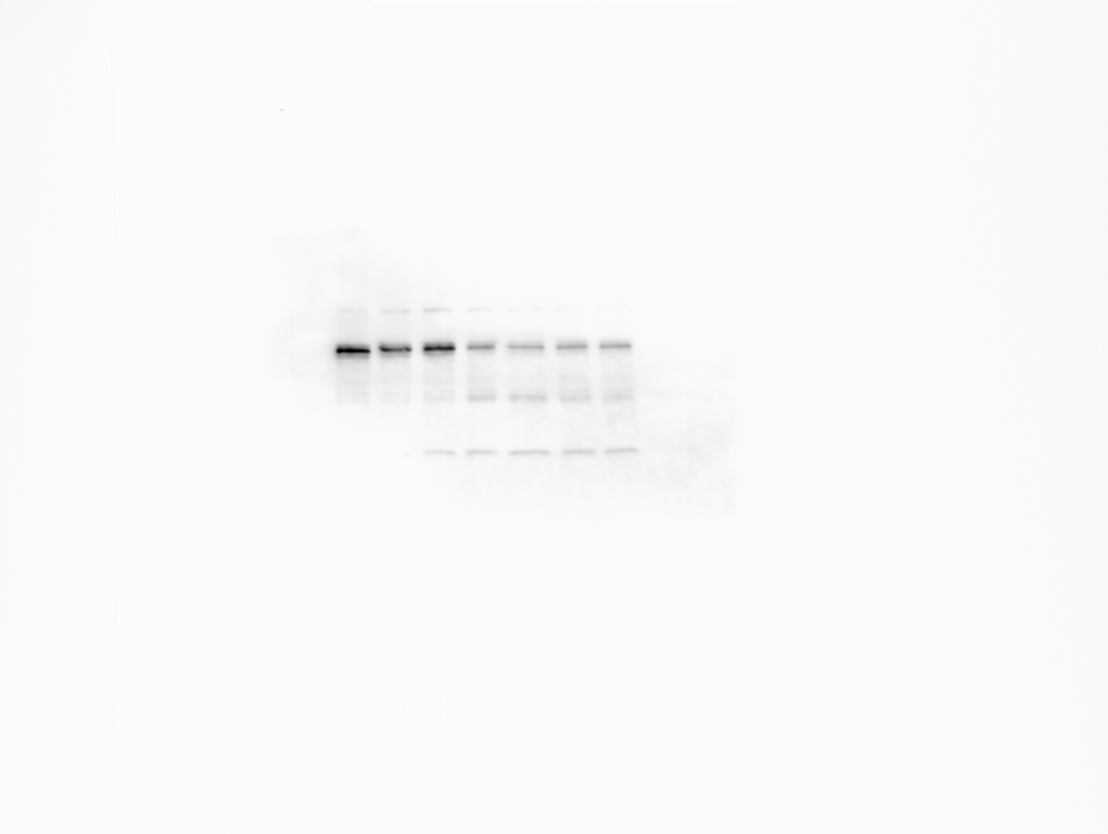

Supplement: Figure 1—source data 2. [file elife-100747-fig1-data2.zip › Figure 1 - Source Data 2 (original western files)/gls-mo-ptg/S1F9-1118-154704.tif]

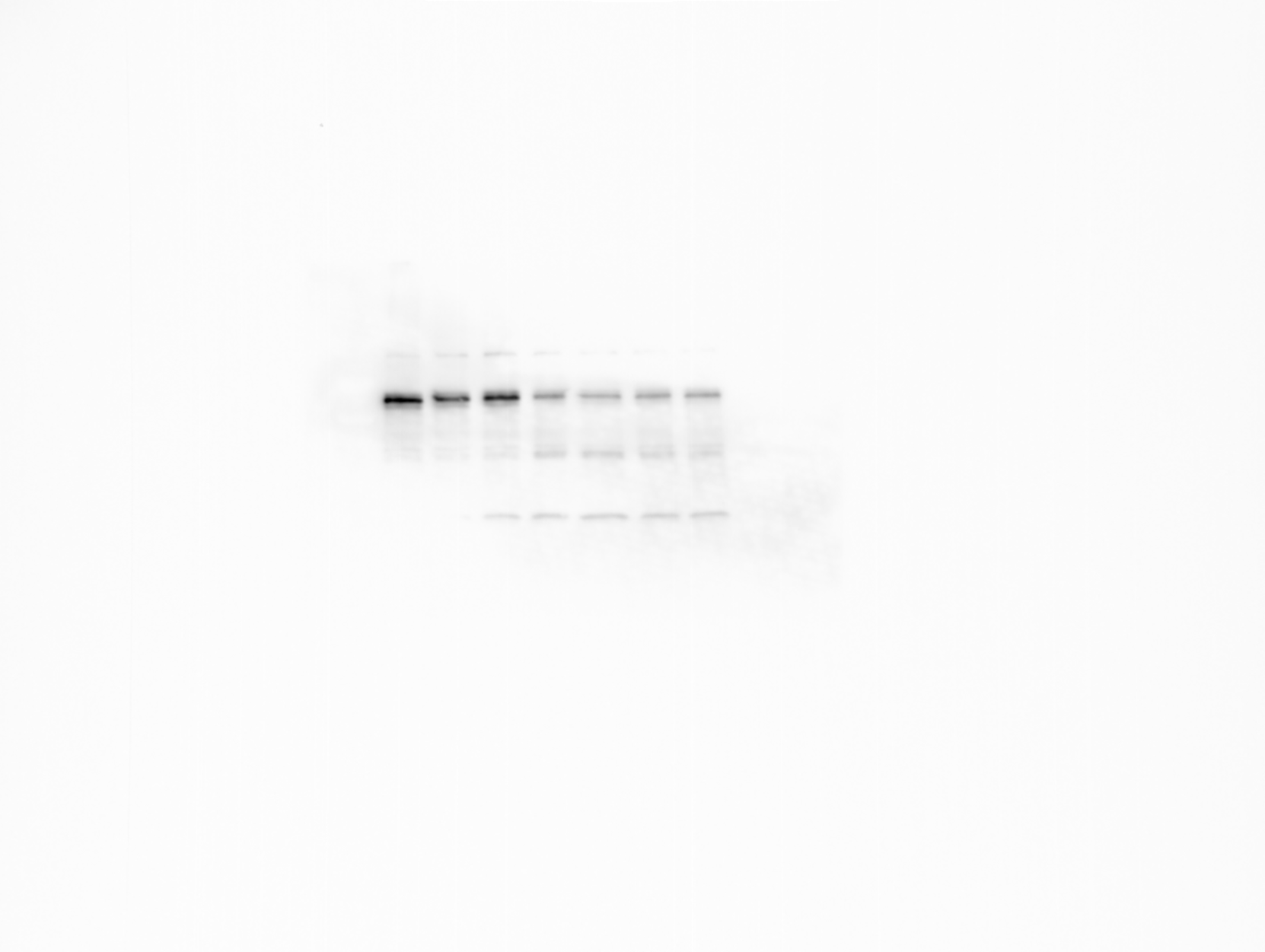

Supplement: Figure 1—source data 2. [file elife-100747-fig1-data2.zip › Figure 1 - Source Data 2 (original western files)/gls-mo-ptg/S1F9-1118-154704_pub.tif]

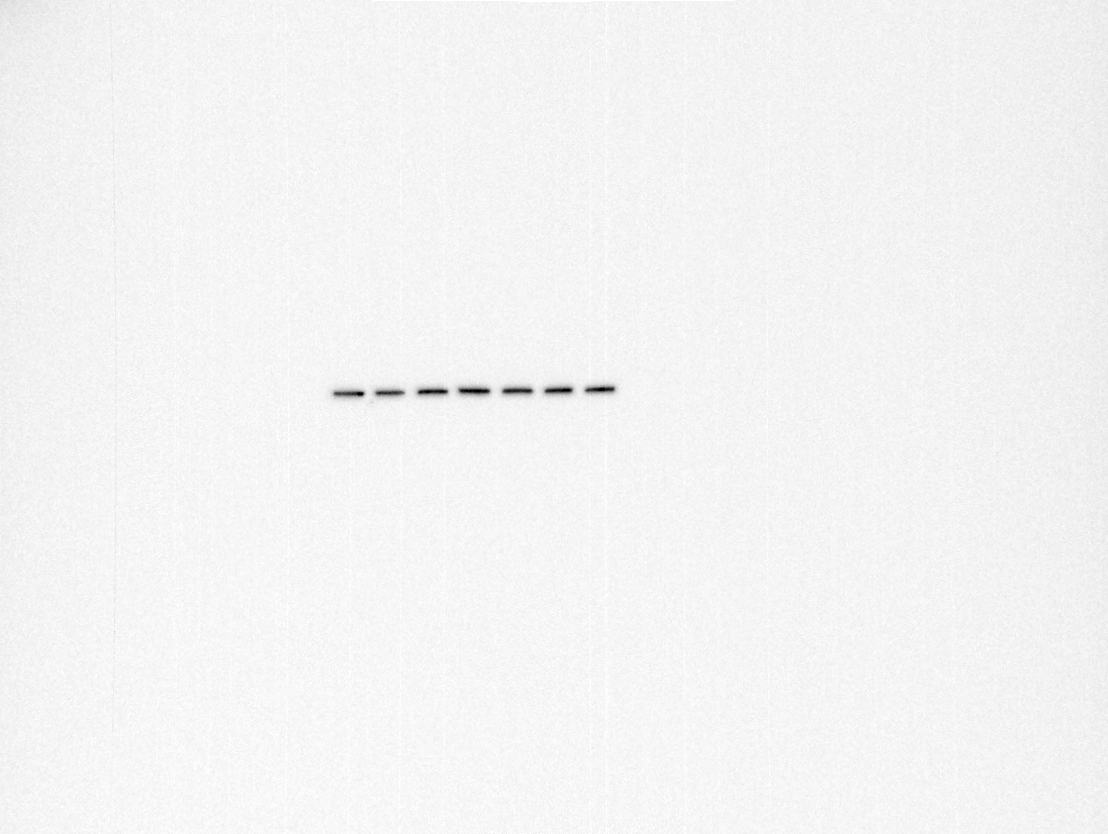

Supplement: Figure 1—source data 2. [file elife-100747-fig1-data2.zip › Figure 1 - Source Data 2 (original western files)/hsp90/2022-1118-155132.tif]

Office DEPOT®

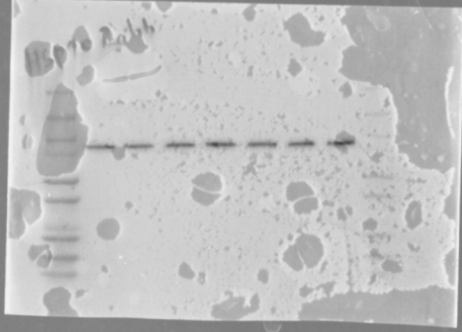

Supplement: Figure 1—source data 2. [file elife-100747-fig1-data2.zip › Figure 1 - Source Data 2 (original western files)/hsp90/2022-1118-155132_pub.pdf]

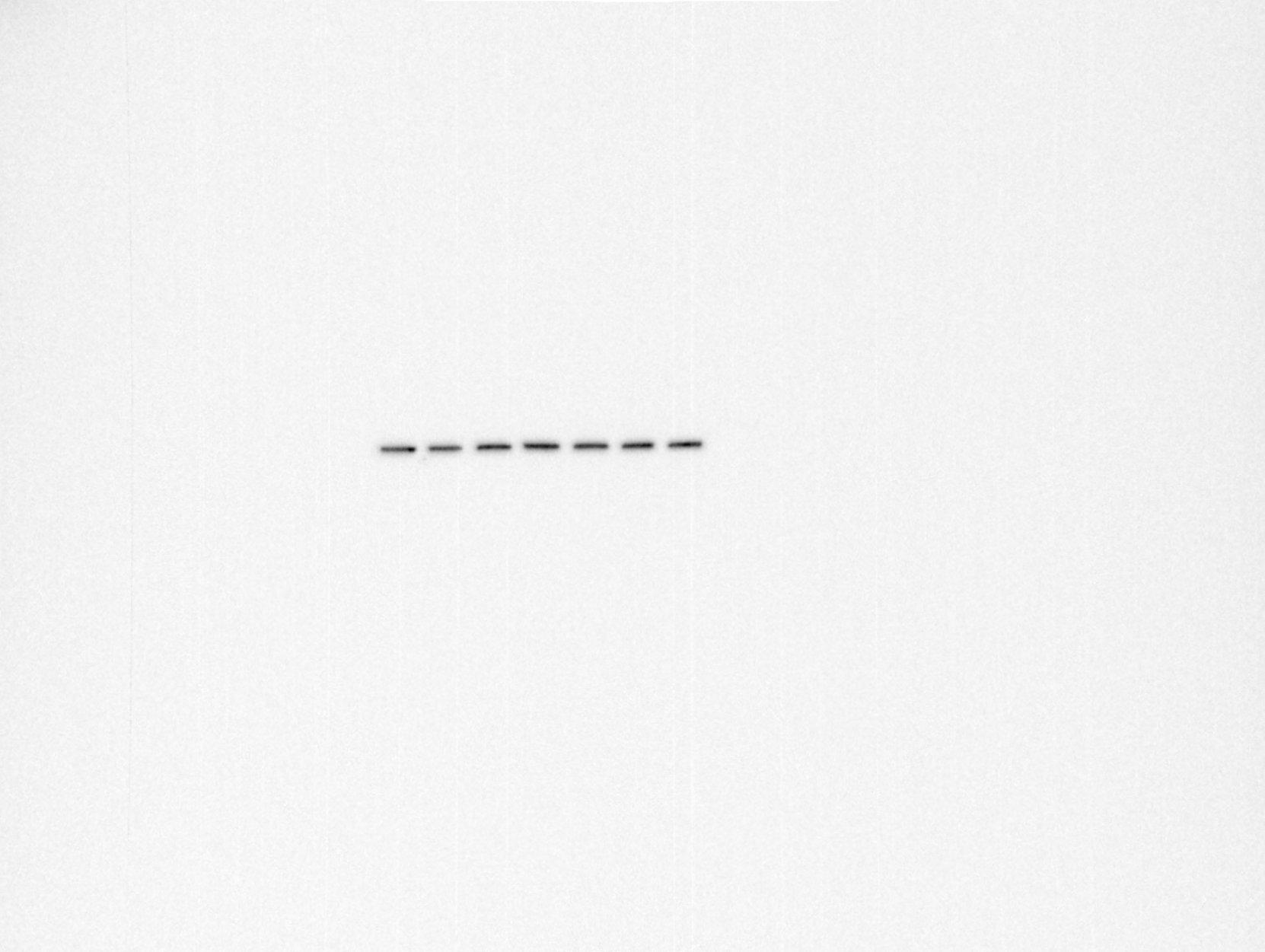

Supplement: Figure 1—source data 2. [file elife-100747-fig1-data2.zip › Figure 1 - Source Data 2 (original western files)/hsp90/2022-1118-155132_pub.tif]

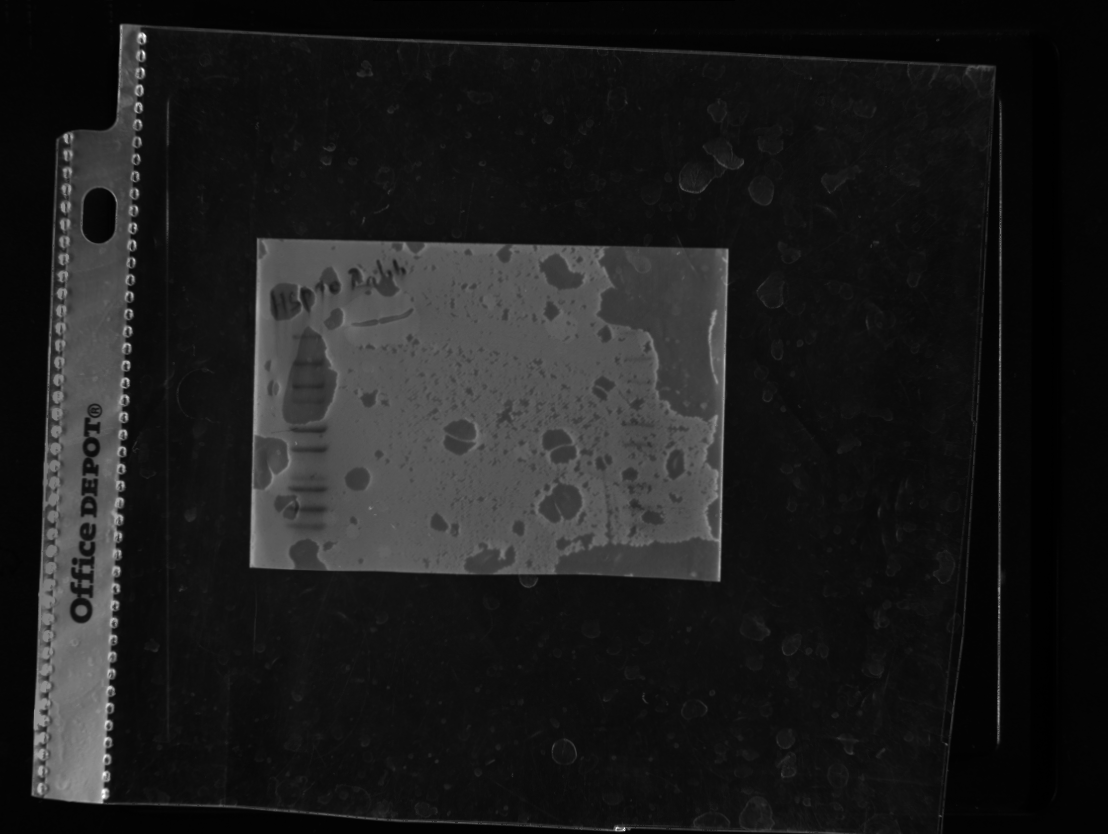

Supplement: Figure 1—source data 2. [file elife-100747-fig1-data2.zip › Figure 1 - Source Data 2 (original western files)/hsp90/2022-1118-155134.tif]

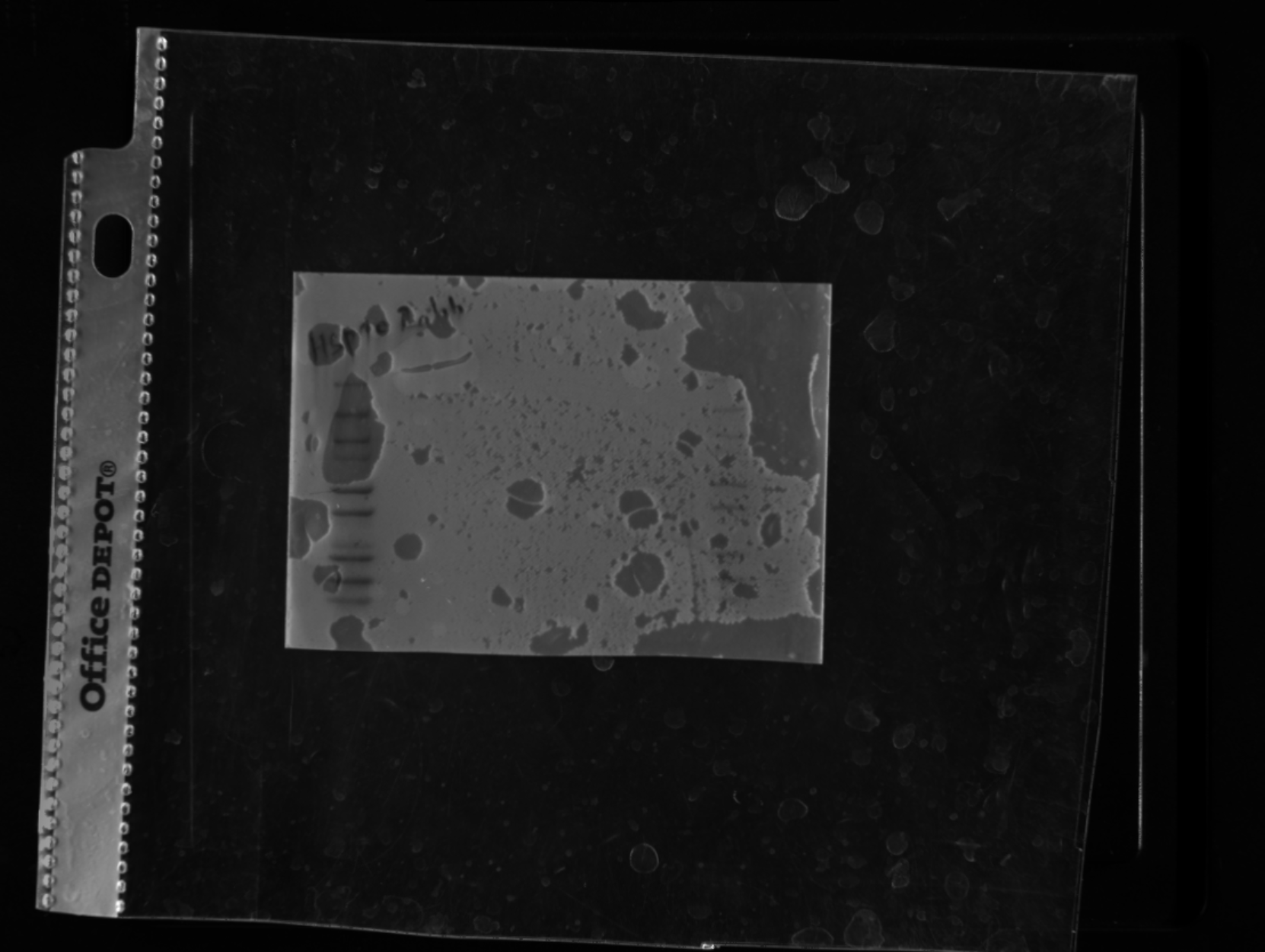

Supplement: Figure 1—source data 2. [file elife-100747-fig1-data2.zip › Figure 1 - Source Data 2 (original western files)/hsp90/2022-1118-155134_pub.tif]

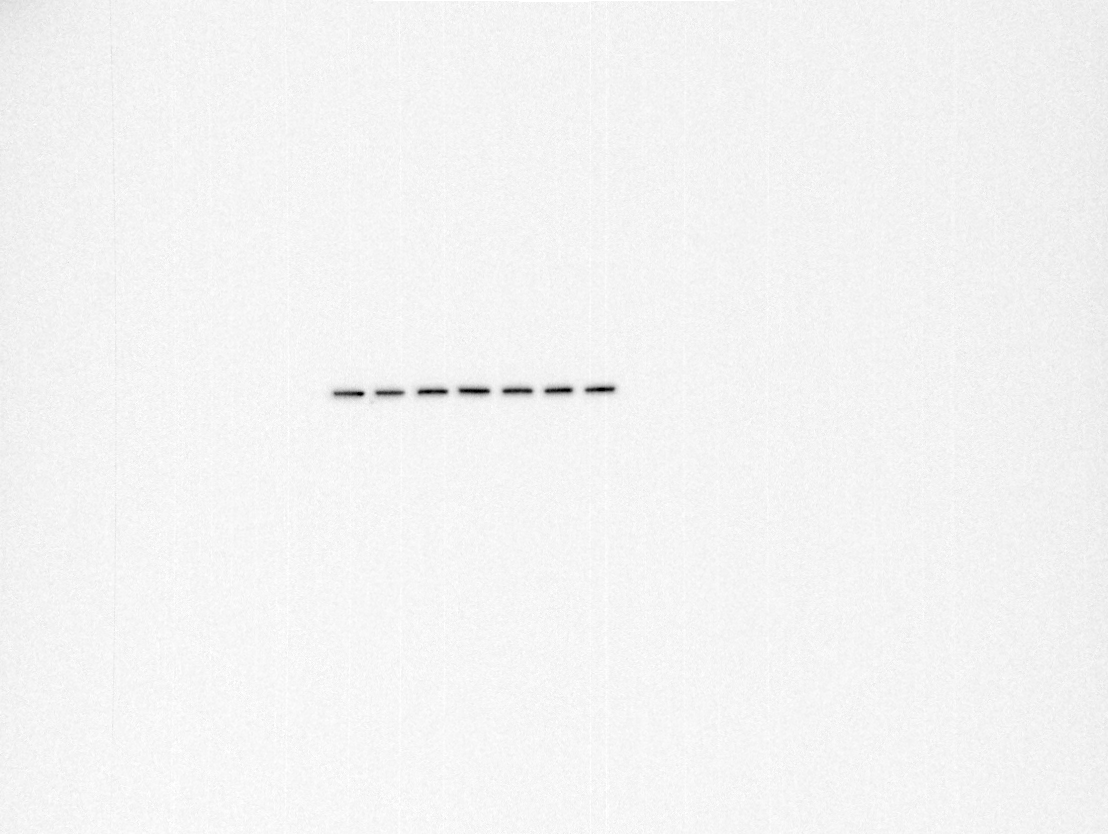

Supplement: Figure 1—source data 2. [file elife-100747-fig1-data2.zip › Figure 1 - Source Data 2 (original western files)/hsp90/2022-1118-155136.tif]

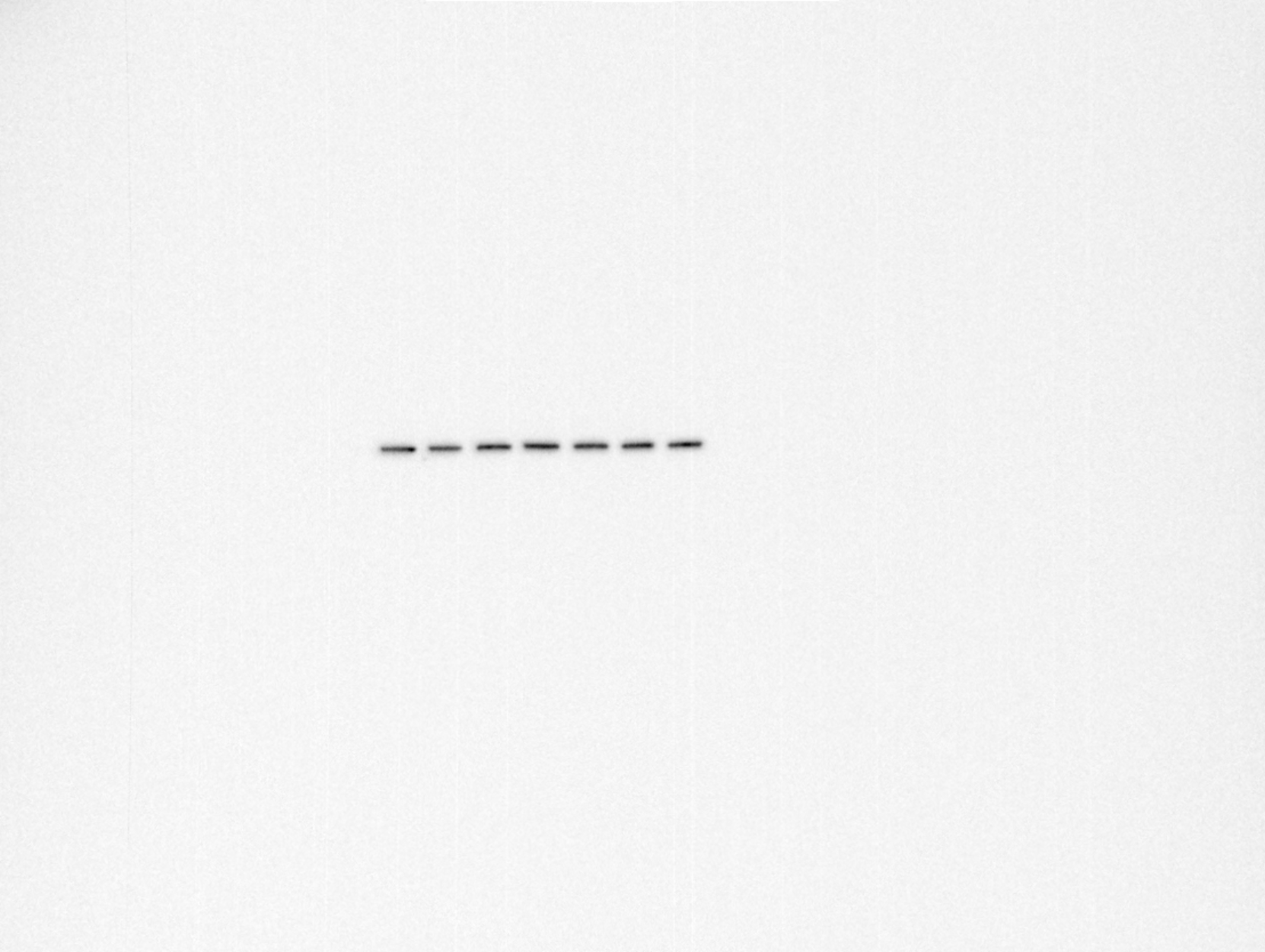

Supplement: Figure 1—source data 2. [file elife-100747-fig1-data2.zip › Figure 1 - Source Data 2 (original western files)/hsp90/2022-1118-155136_pub.tif]

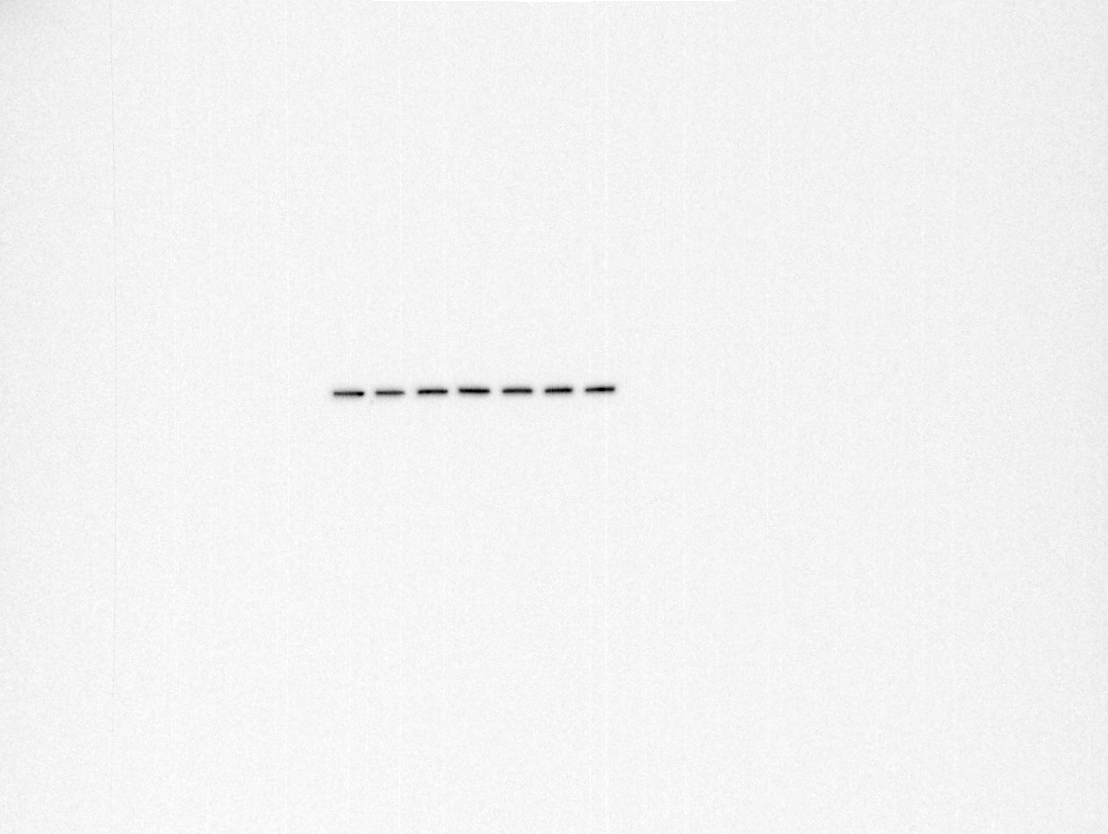

Supplement: Figure 1—source data 2. [file elife-100747-fig1-data2.zip › Figure 1 - Source Data 2 (original western files)/hsp90/S2F1-1118-155137.tif]

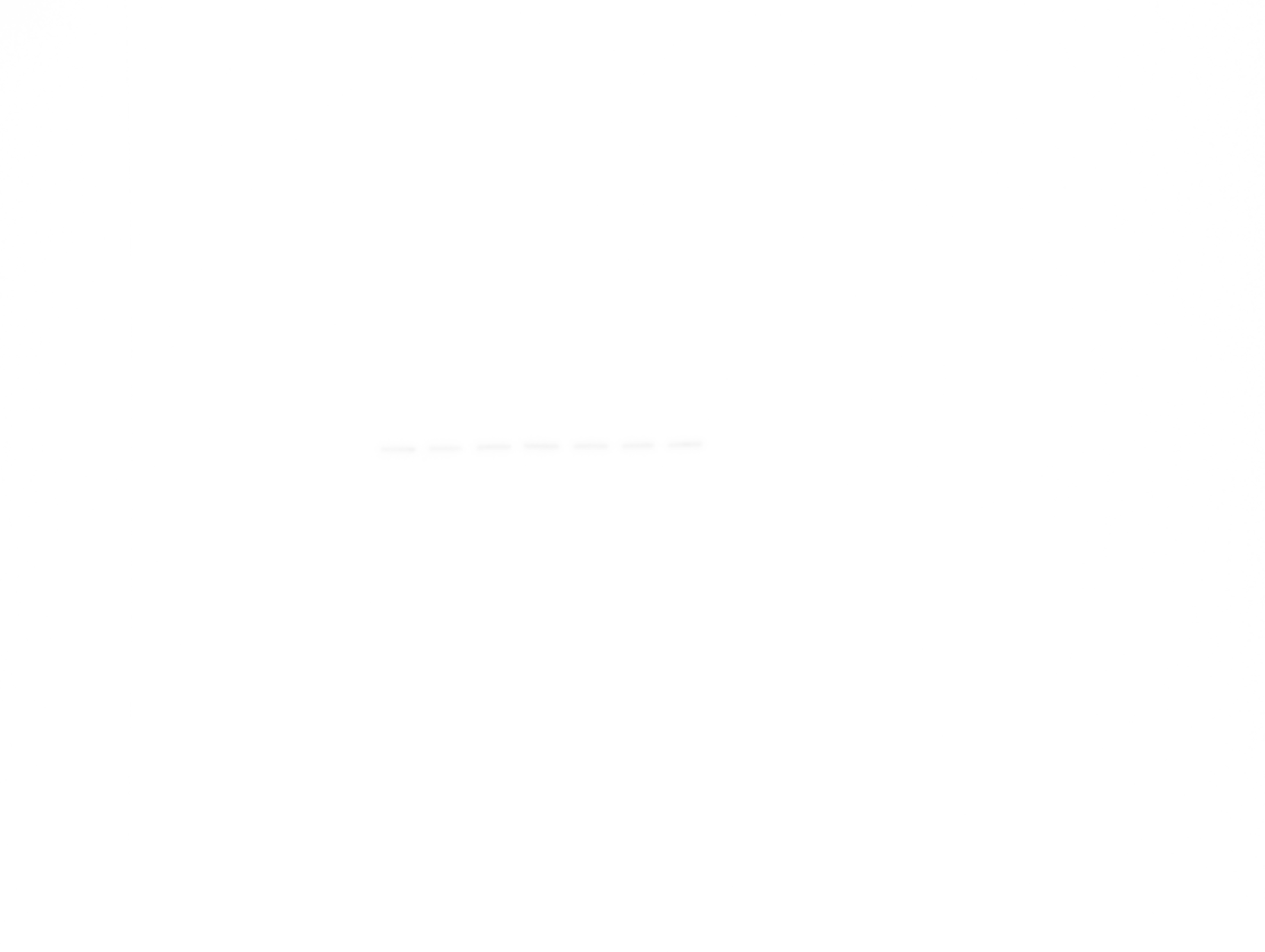

Supplement: Figure 1—source data 2. [file elife-100747-fig1-data2.zip › Figure 1 - Source Data 2 (original western files)/hsp90/S2F1-1118-155137_pub.tif]

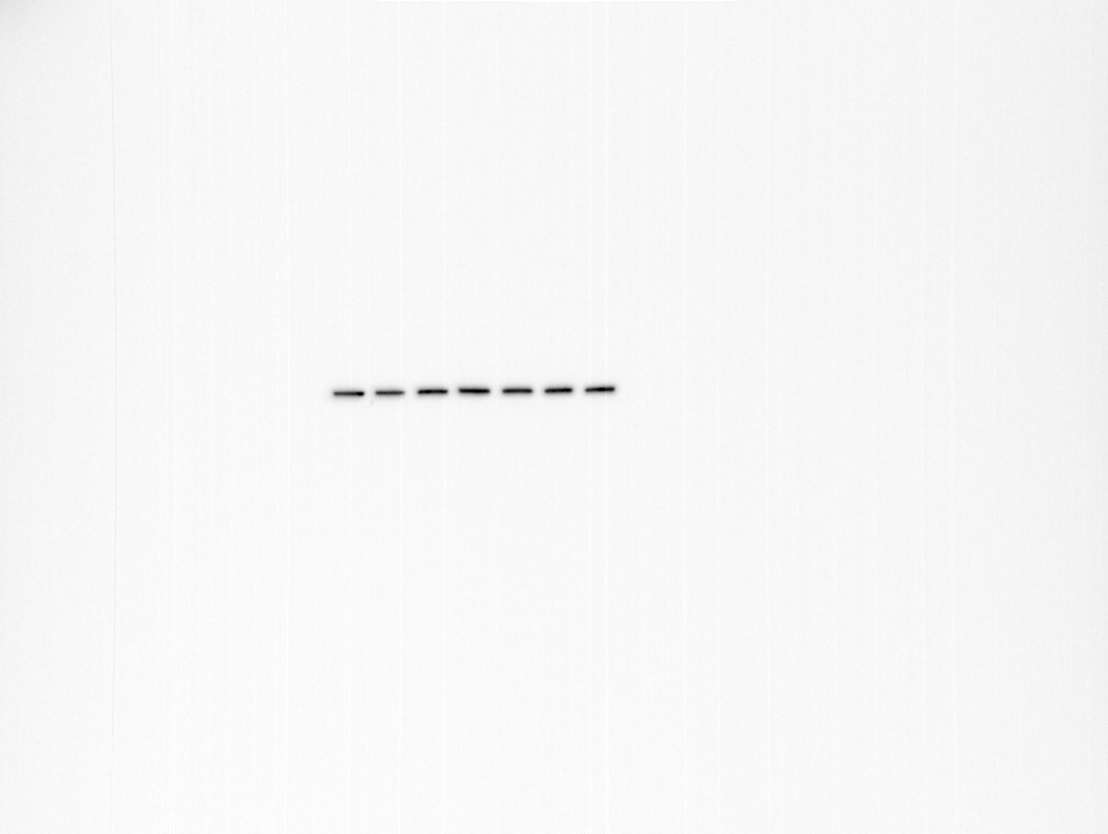

Supplement: Figure 1—source data 2. [file elife-100747-fig1-data2.zip › Figure 1 - Source Data 2 (original western files)/hsp90/S2F10-1118-155153.tif]

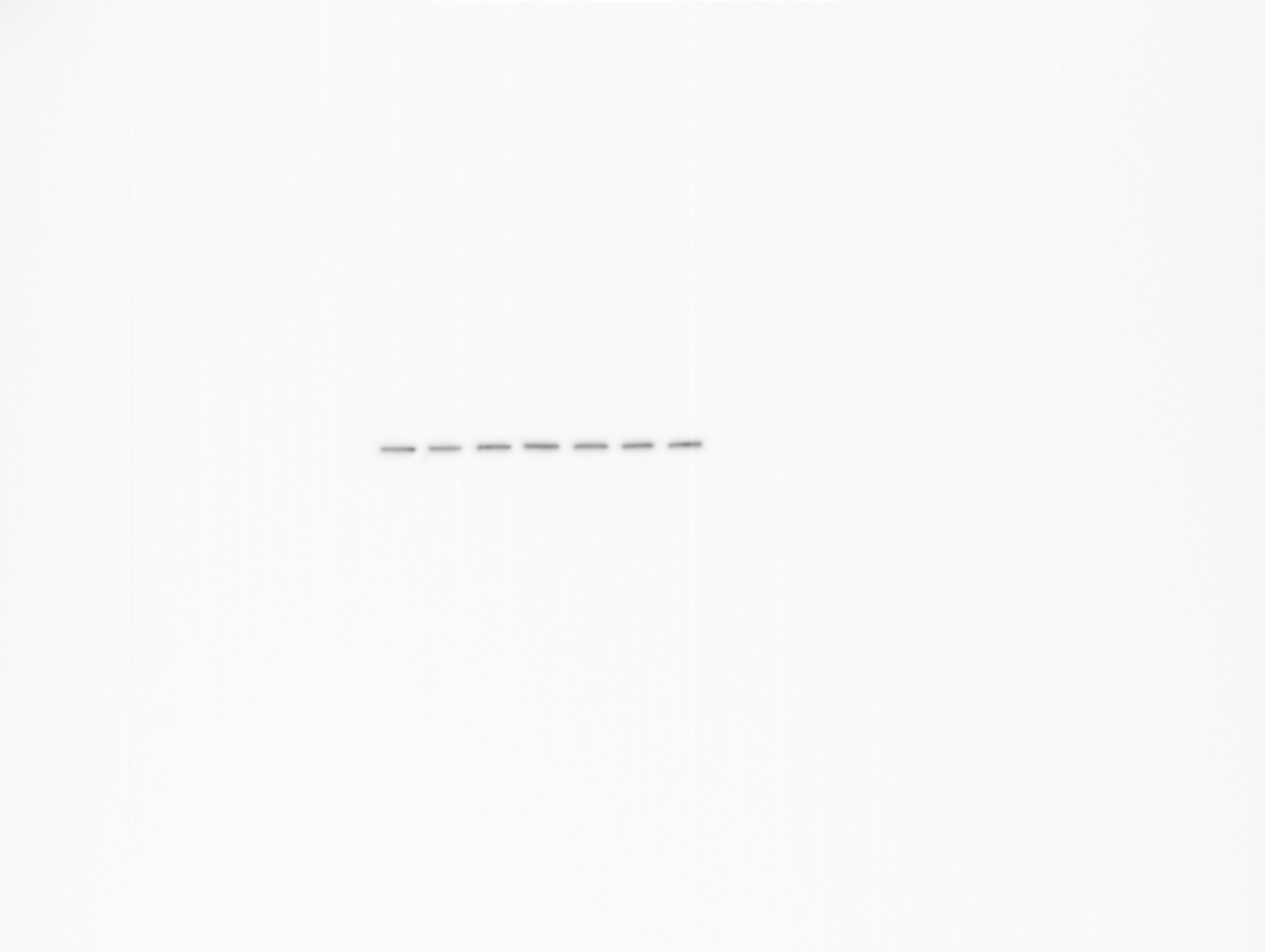

Supplement: Figure 1—source data 2. [file elife-100747-fig1-data2.zip › Figure 1 - Source Data 2 (original western files)/hsp90/S2F10-1118-155153_pub.tif]

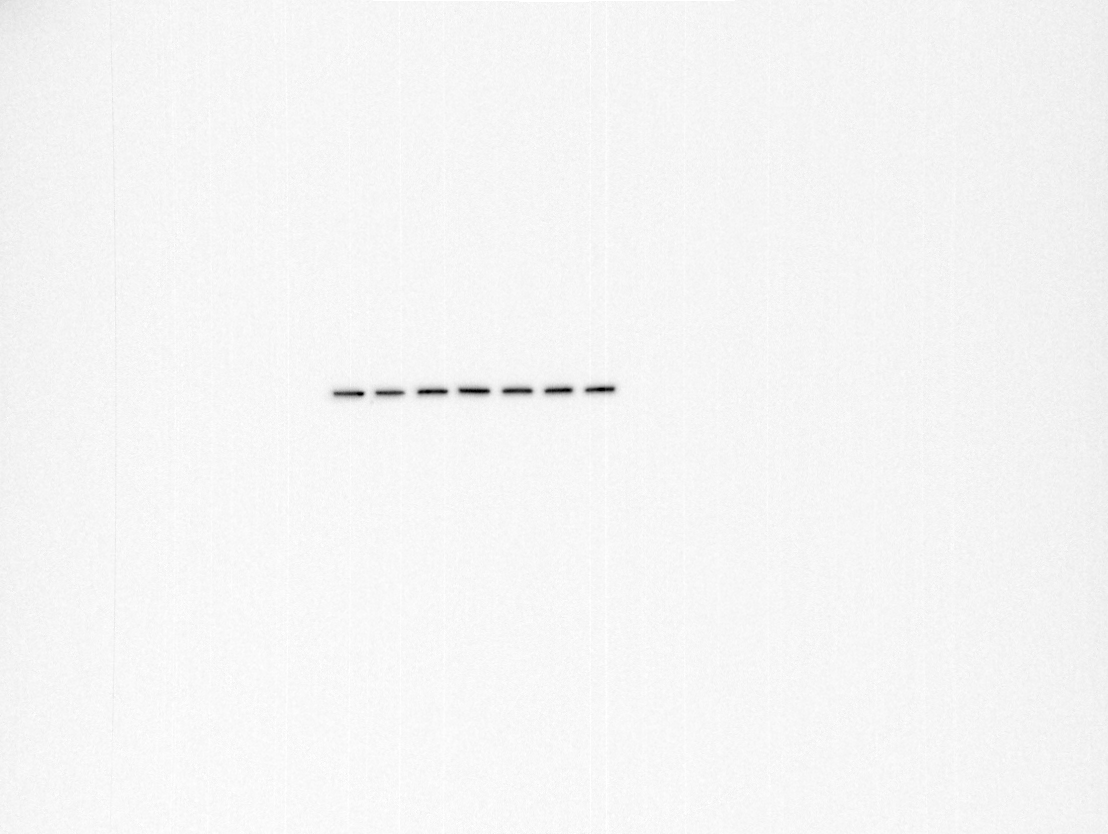

Supplement: Figure 1—source data 2. [file elife-100747-fig1-data2.zip › Figure 1 - Source Data 2 (original western files)/hsp90/S2F2-1118-155139.tif]

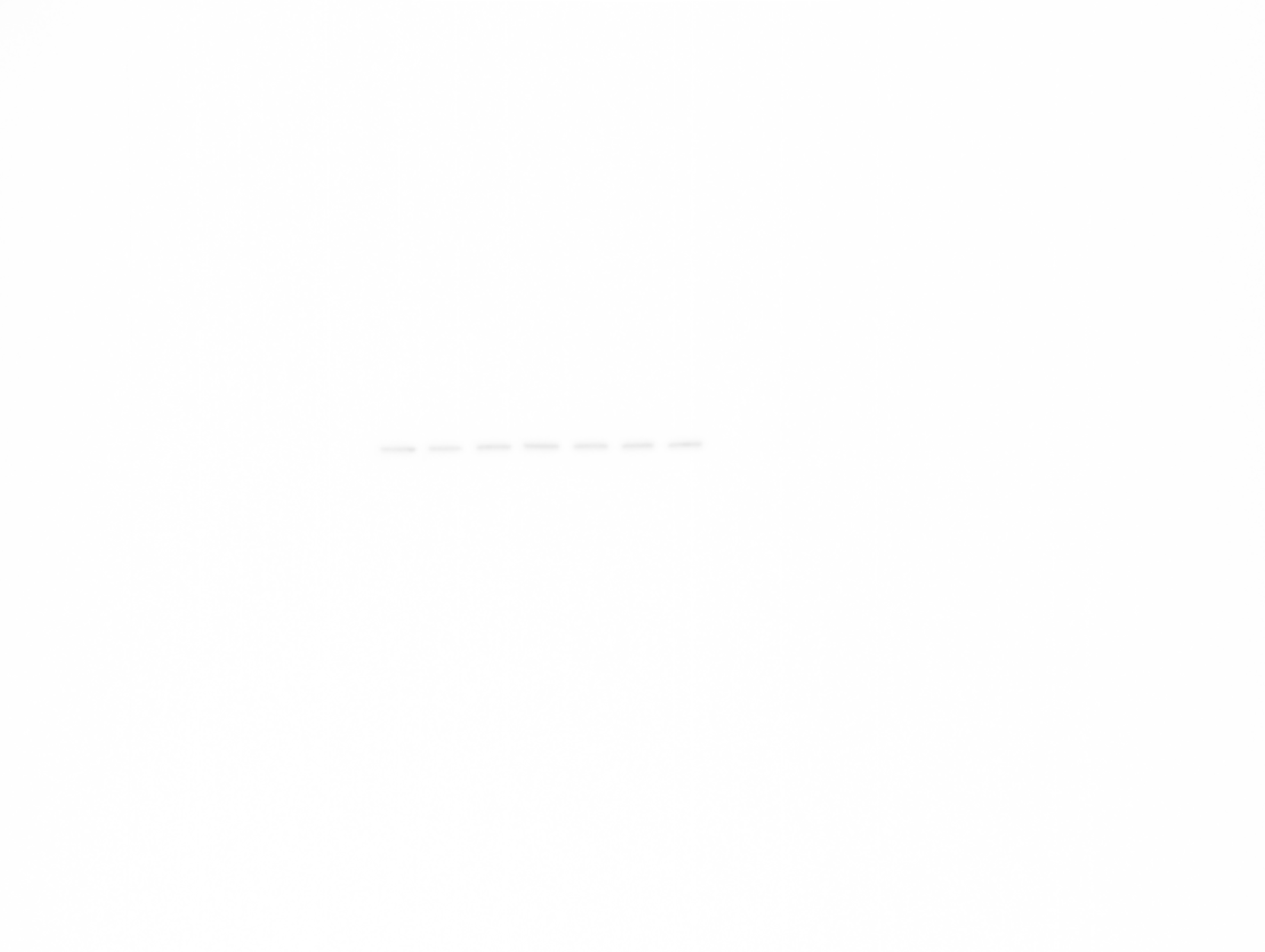

Supplement: Figure 1—source data 2. [file elife-100747-fig1-data2.zip › Figure 1 - Source Data 2 (original western files)/hsp90/S2F2-1118-155139_pub.tif]

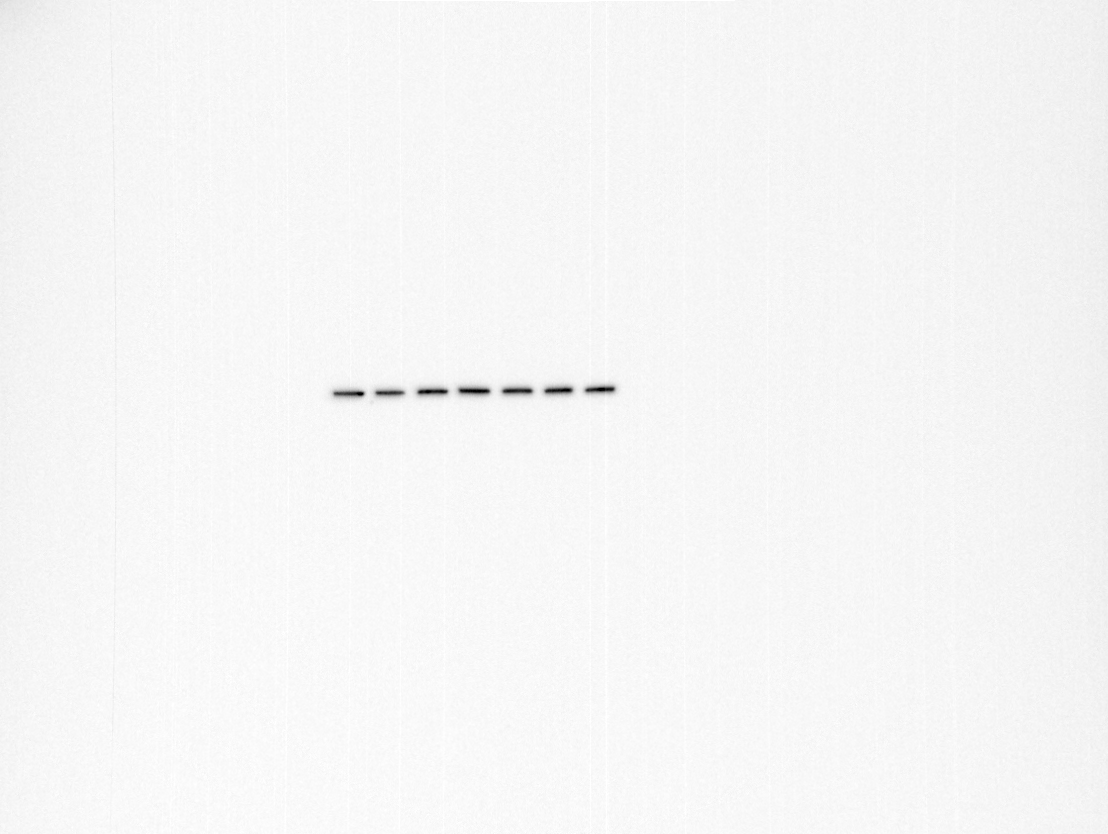

Supplement: Figure 1—source data 2. [file elife-100747-fig1-data2.zip › Figure 1 - Source Data 2 (original western files)/hsp90/S2F3-1118-155141.tif]

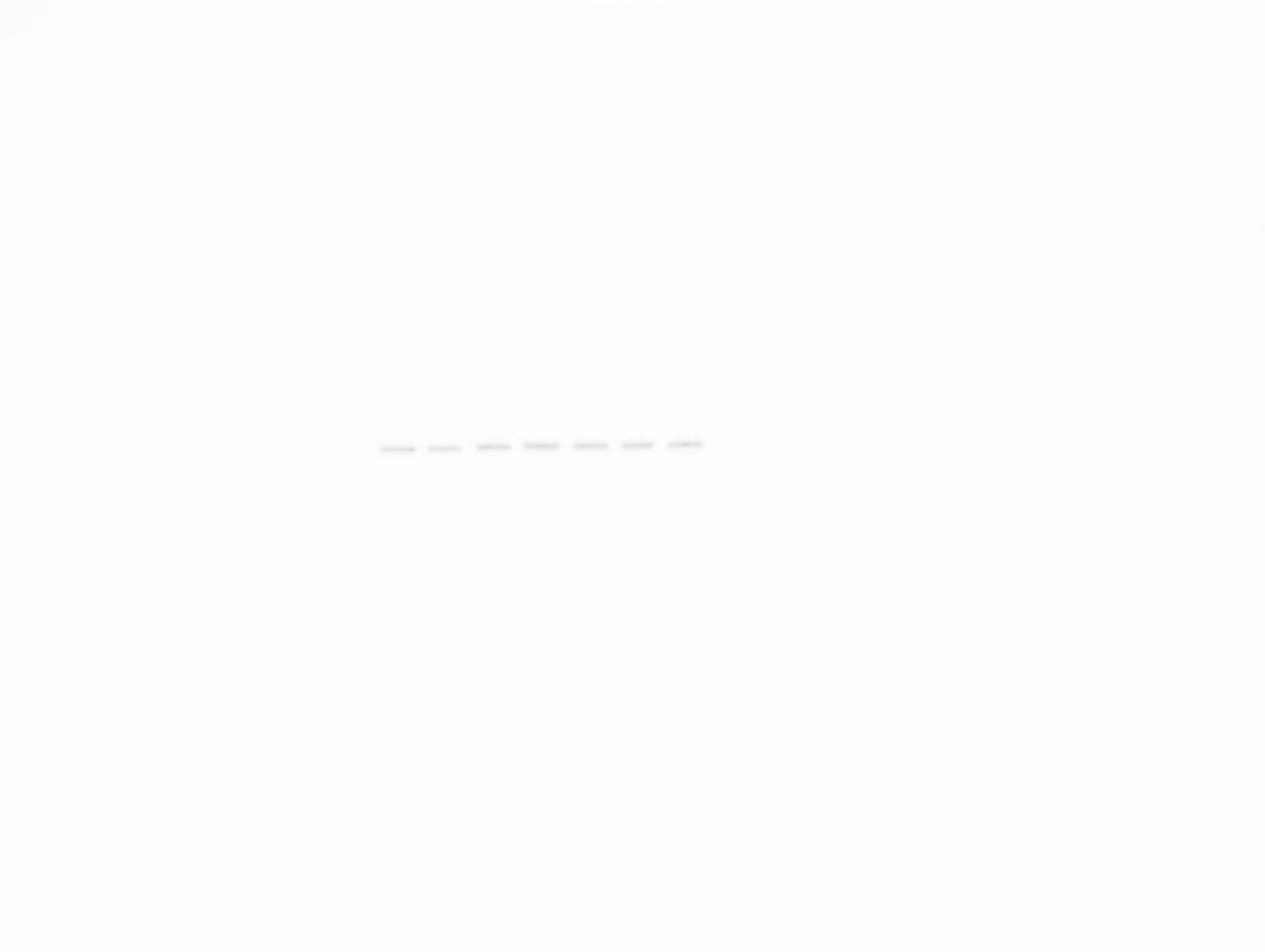

Supplement: Figure 1—source data 2. [file elife-100747-fig1-data2.zip › Figure 1 - Source Data 2 (original western files)/hsp90/S2F3-1118-155141_pub.tif]

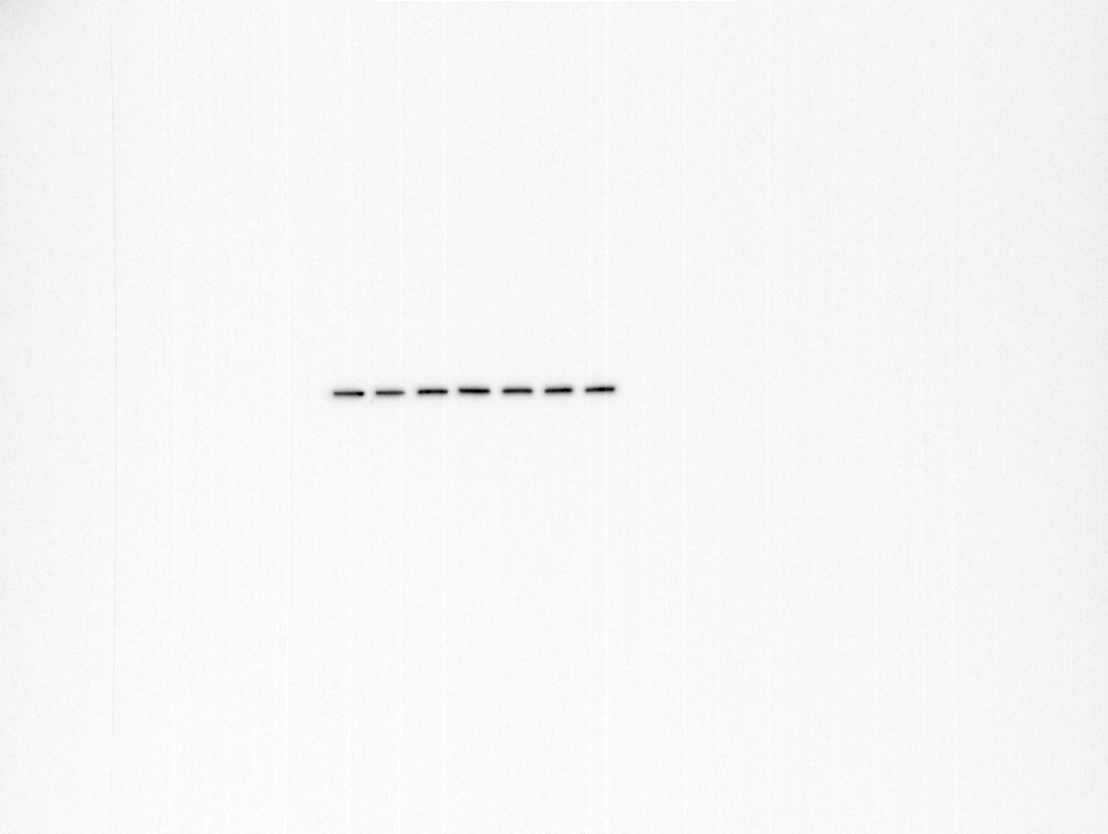

Supplement: Figure 1—source data 2. [file elife-100747-fig1-data2.zip › Figure 1 - Source Data 2 (original western files)/hsp90/S2F4-1118-155143.tif]

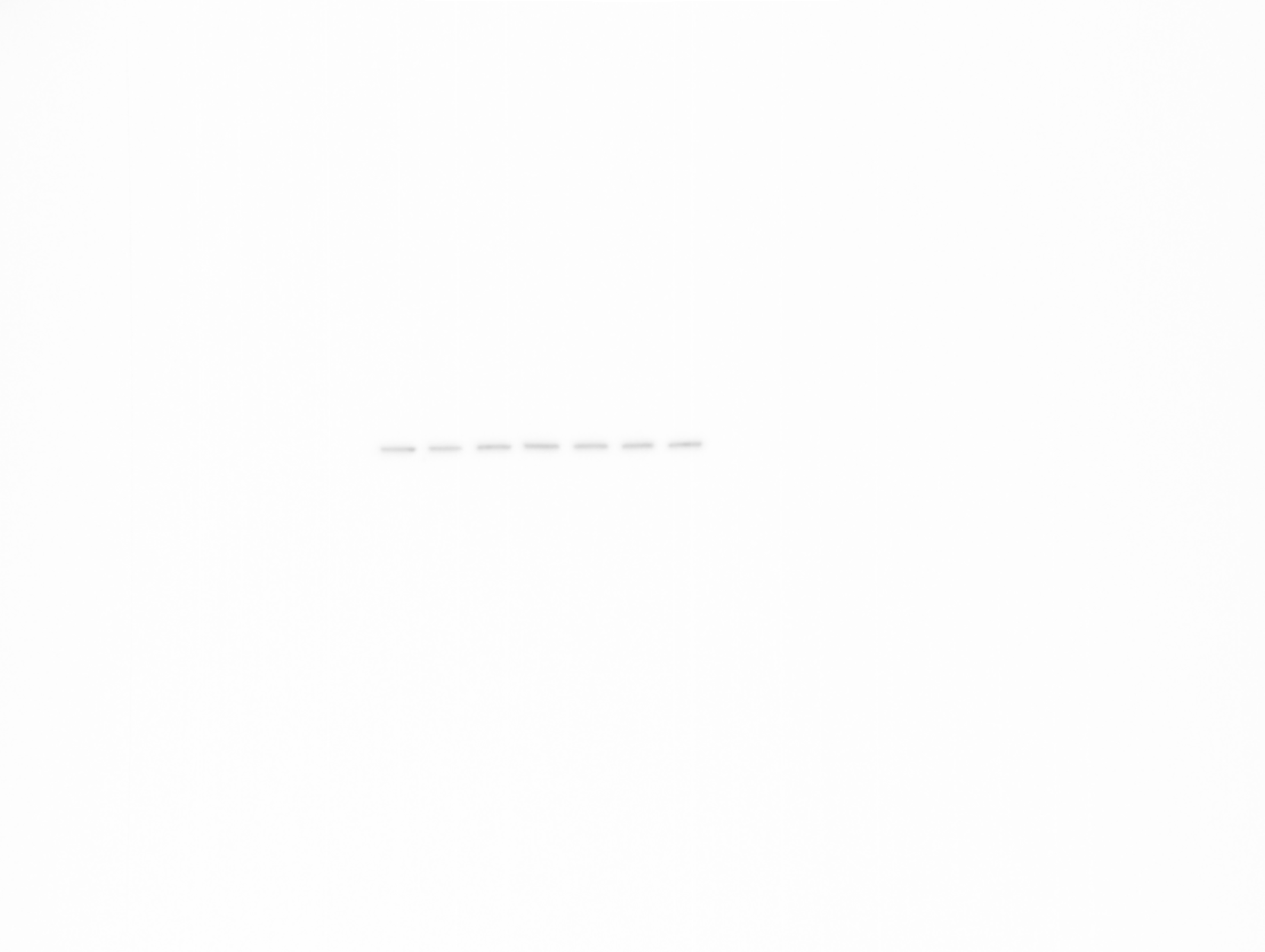

Supplement: Figure 1—source data 2. [file elife-100747-fig1-data2.zip › Figure 1 - Source Data 2 (original western files)/hsp90/S2F4-1118-155143_pub.tif]

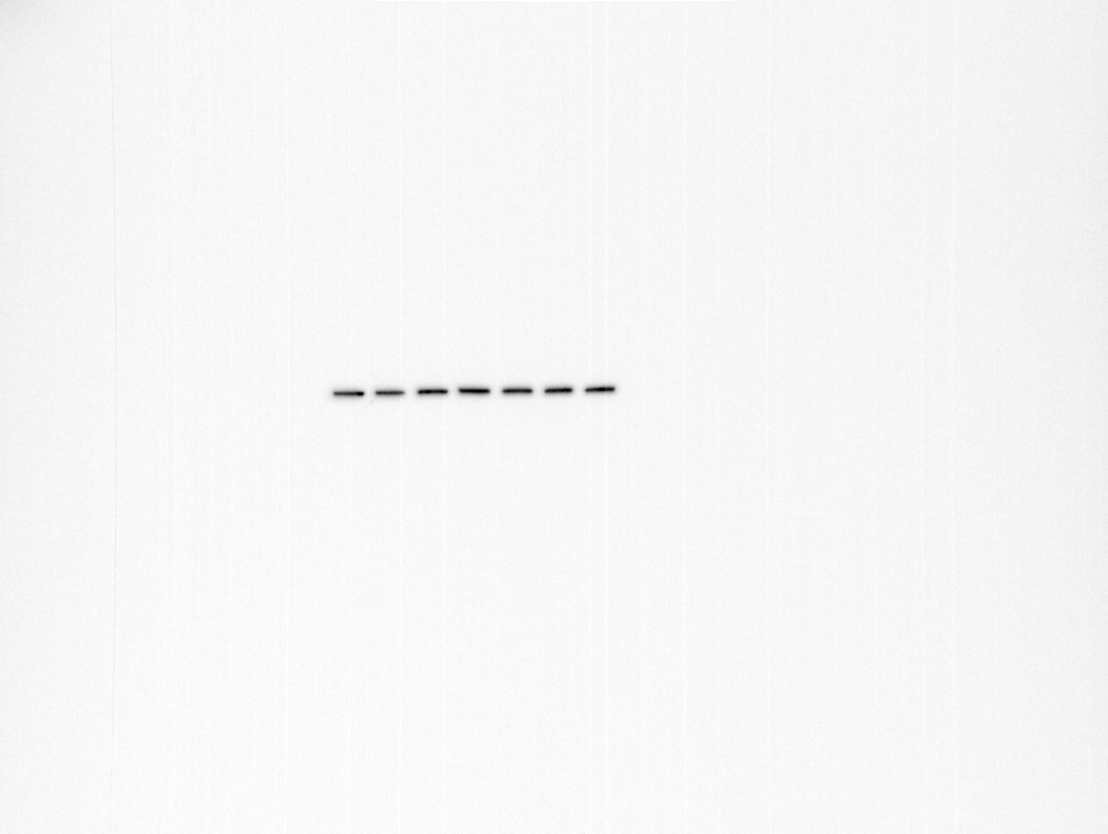

Supplement: Figure 1—source data 2. [file elife-100747-fig1-data2.zip › Figure 1 - Source Data 2 (original western files)/hsp90/S2F5-1118-155144.tif]

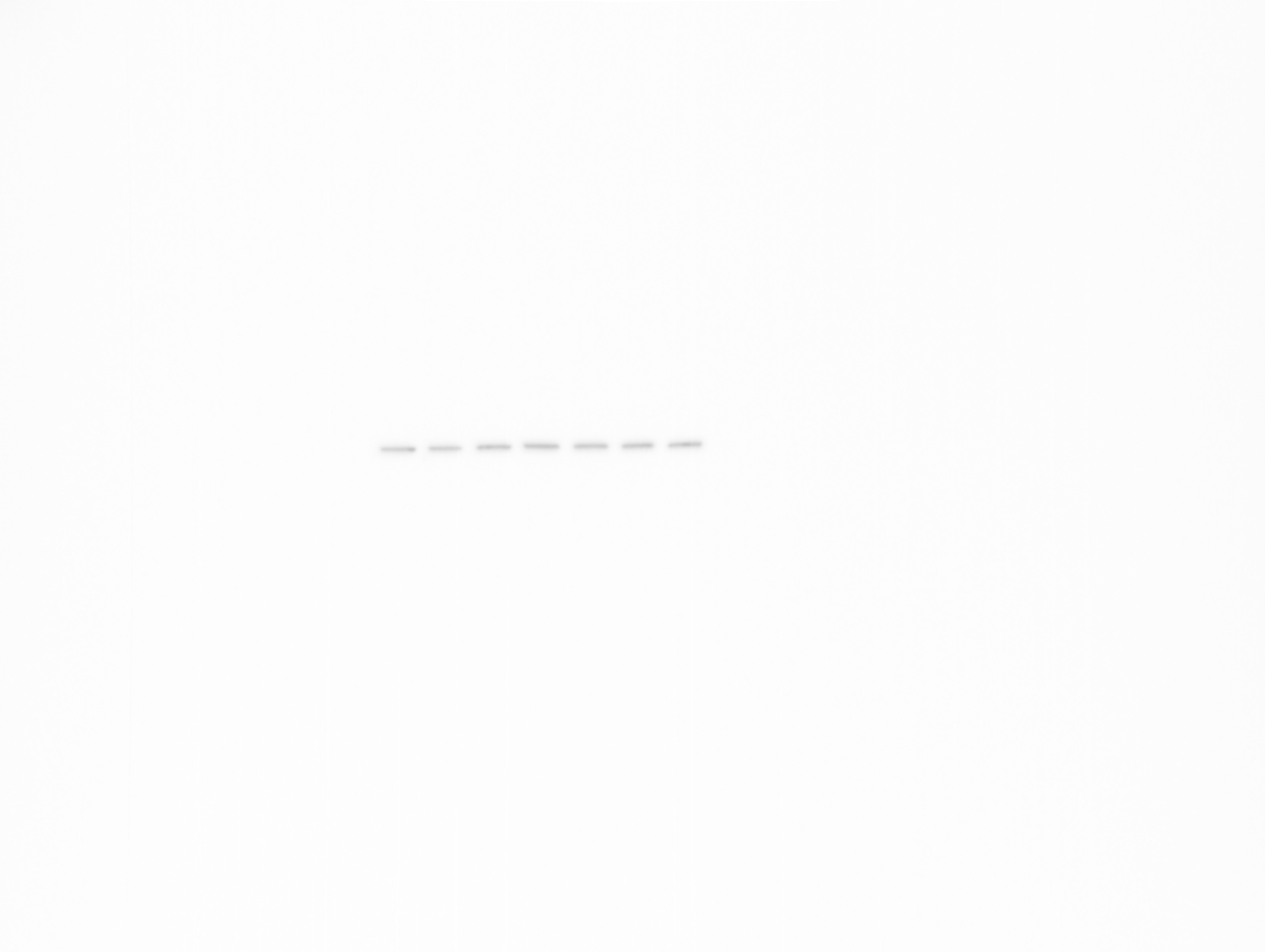

Supplement: Figure 1—source data 2. [file elife-100747-fig1-data2.zip › Figure 1 - Source Data 2 (original western files)/hsp90/S2F5-1118-155144_pub.tif]

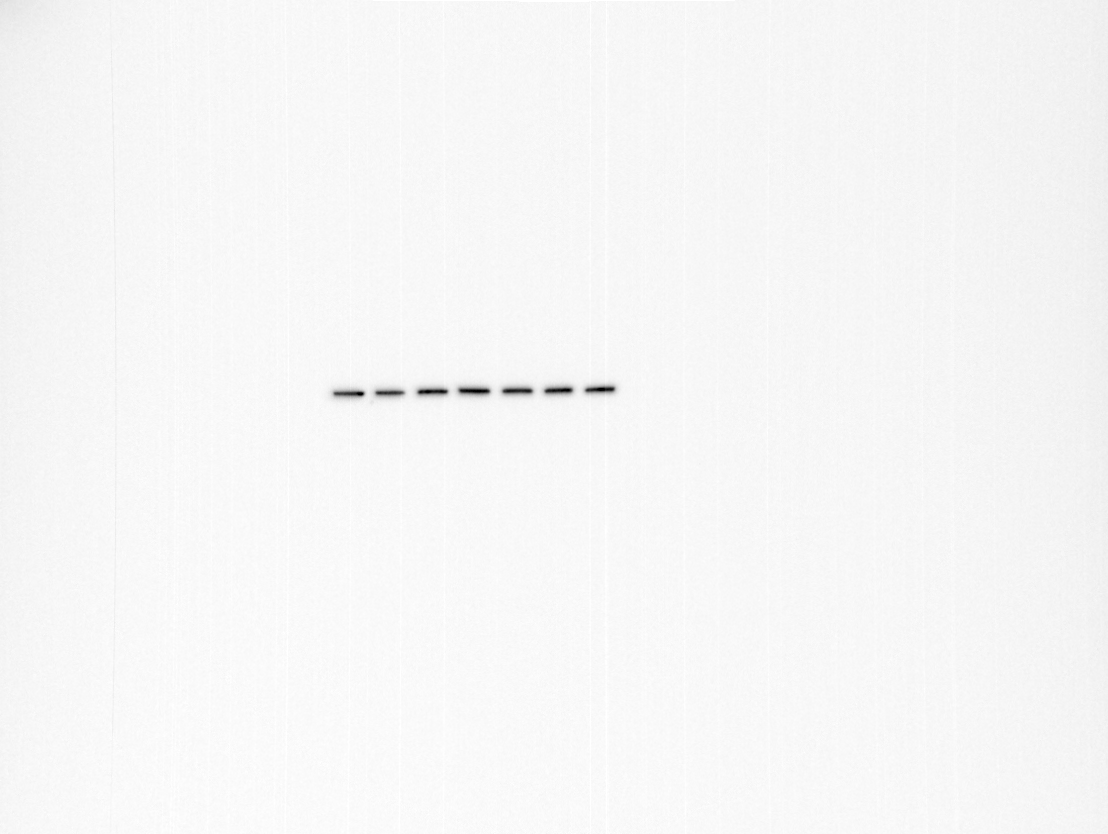

Supplement: Figure 1—source data 2. [file elife-100747-fig1-data2.zip › Figure 1 - Source Data 2 (original western files)/hsp90/S2F6-1118-155146.tif]

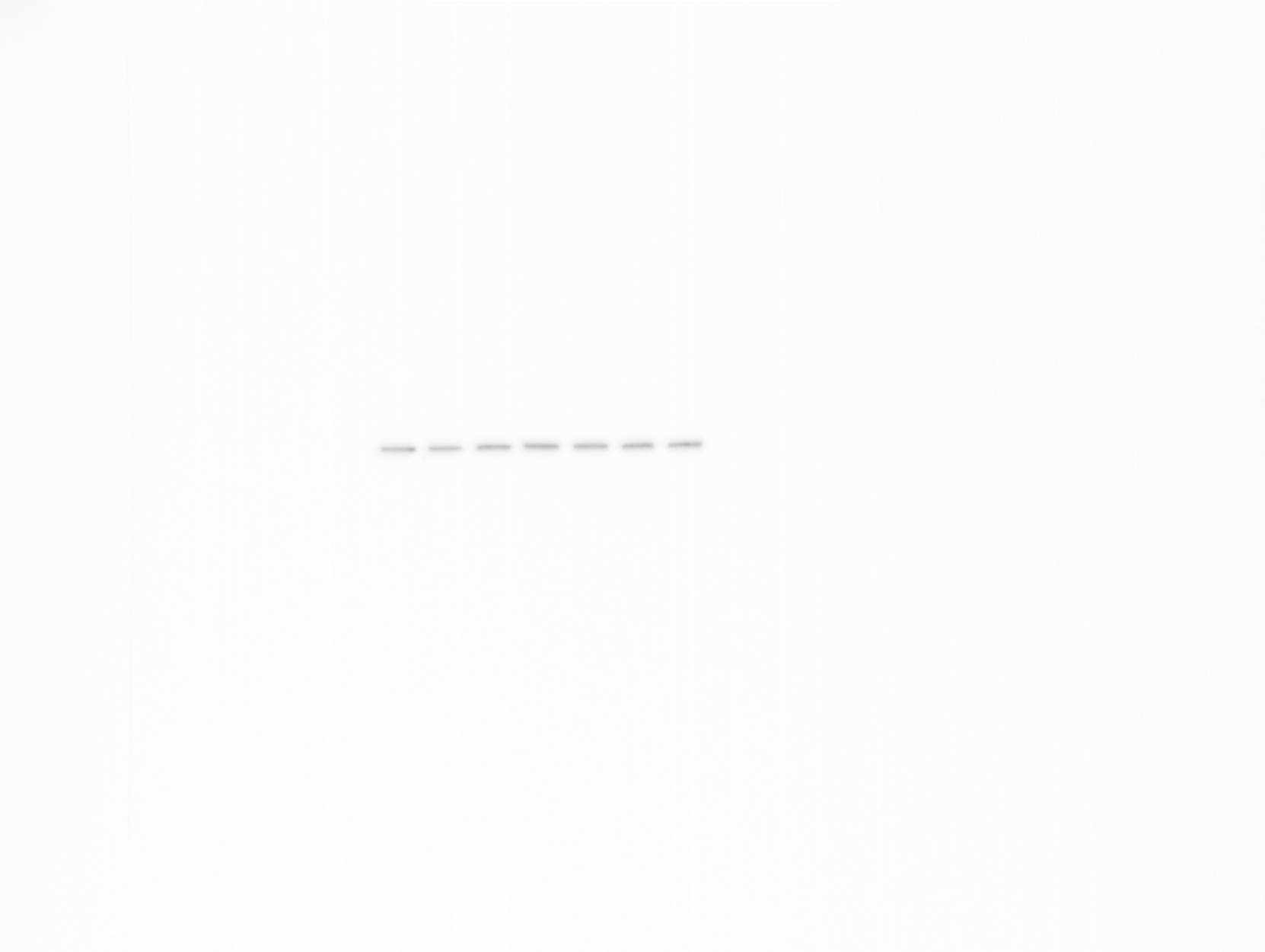

Supplement: Figure 1—source data 2. [file elife-100747-fig1-data2.zip › Figure 1 - Source Data 2 (original western files)/hsp90/S2F6-1118-155146_pub.tif]

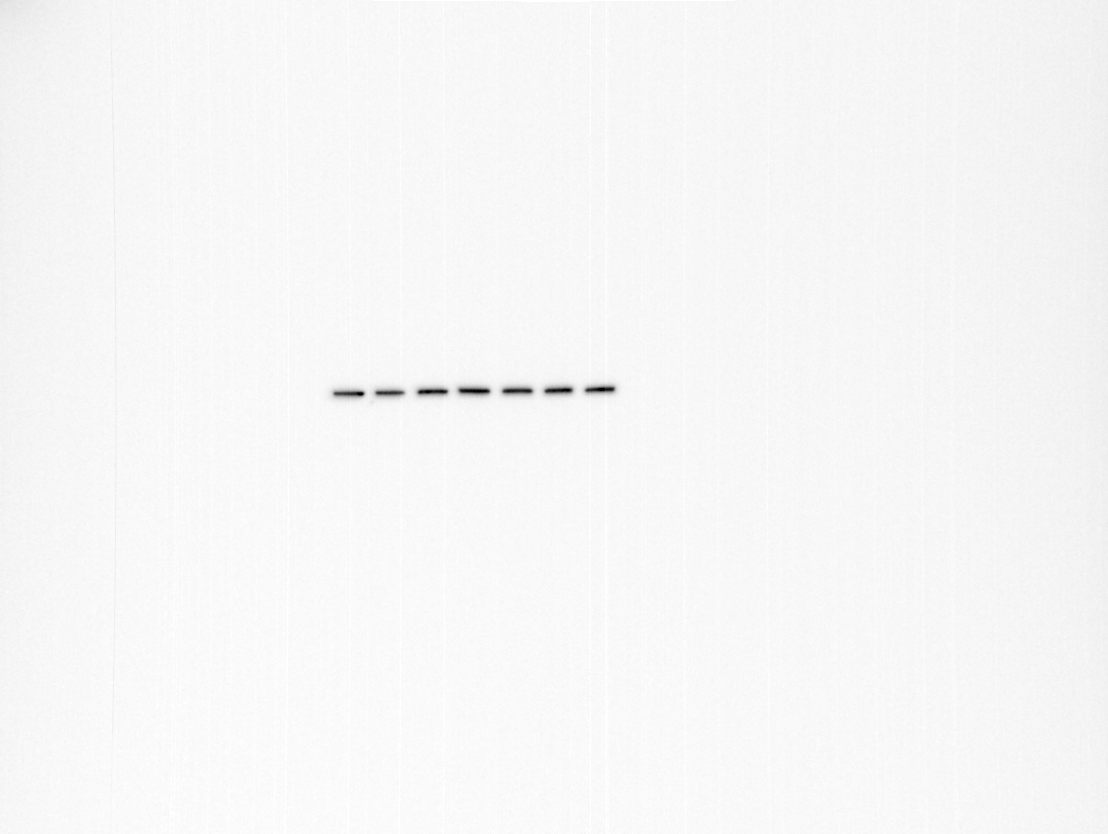

Supplement: Figure 1—source data 2. [file elife-100747-fig1-data2.zip › Figure 1 - Source Data 2 (original western files)/hsp90/S2F7-1118-155148.tif]

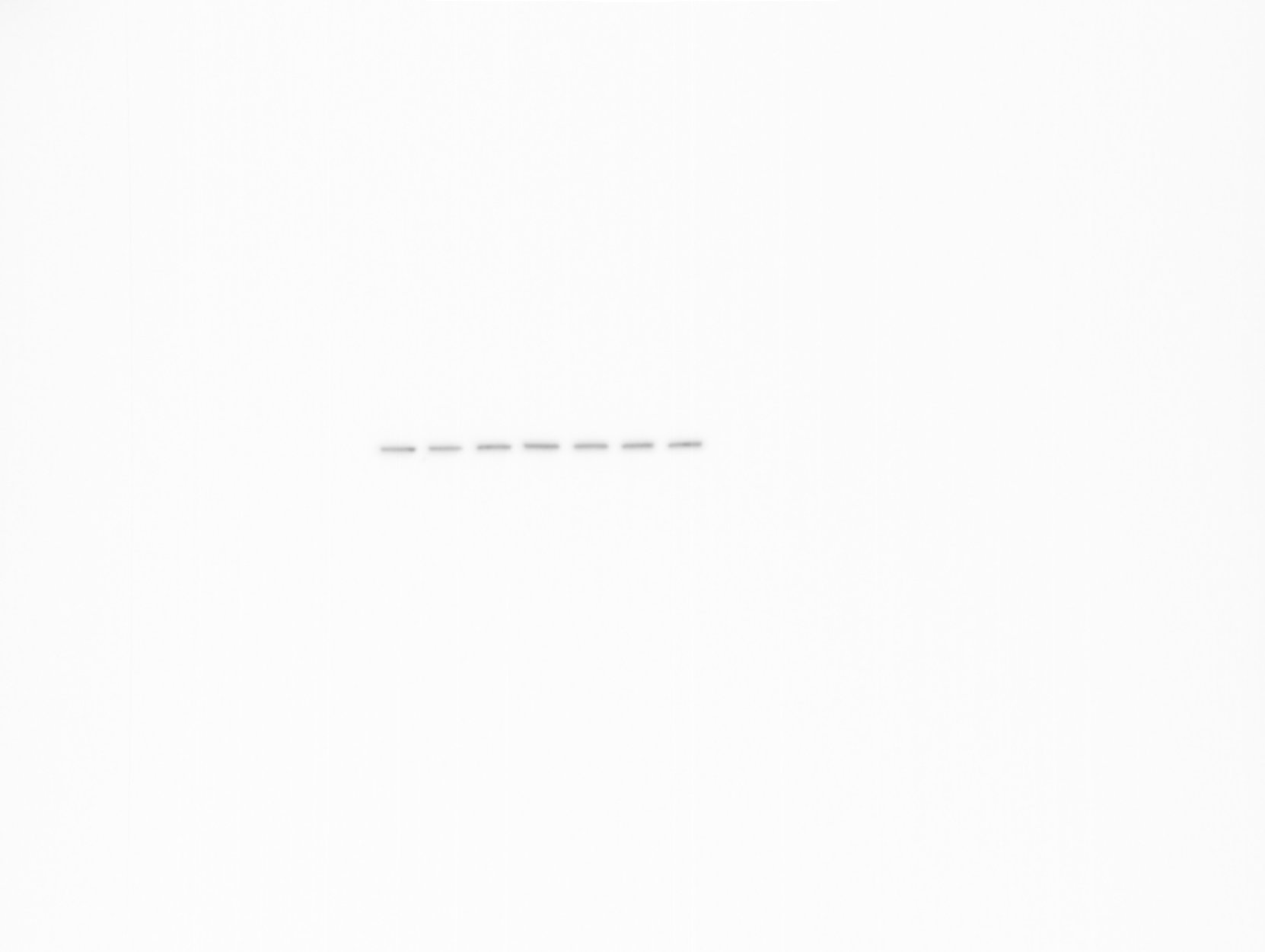

Supplement: Figure 1—source data 2. [file elife-100747-fig1-data2.zip › Figure 1 - Source Data 2 (original western files)/hsp90/S2F7-1118-155148_pub.jpg]

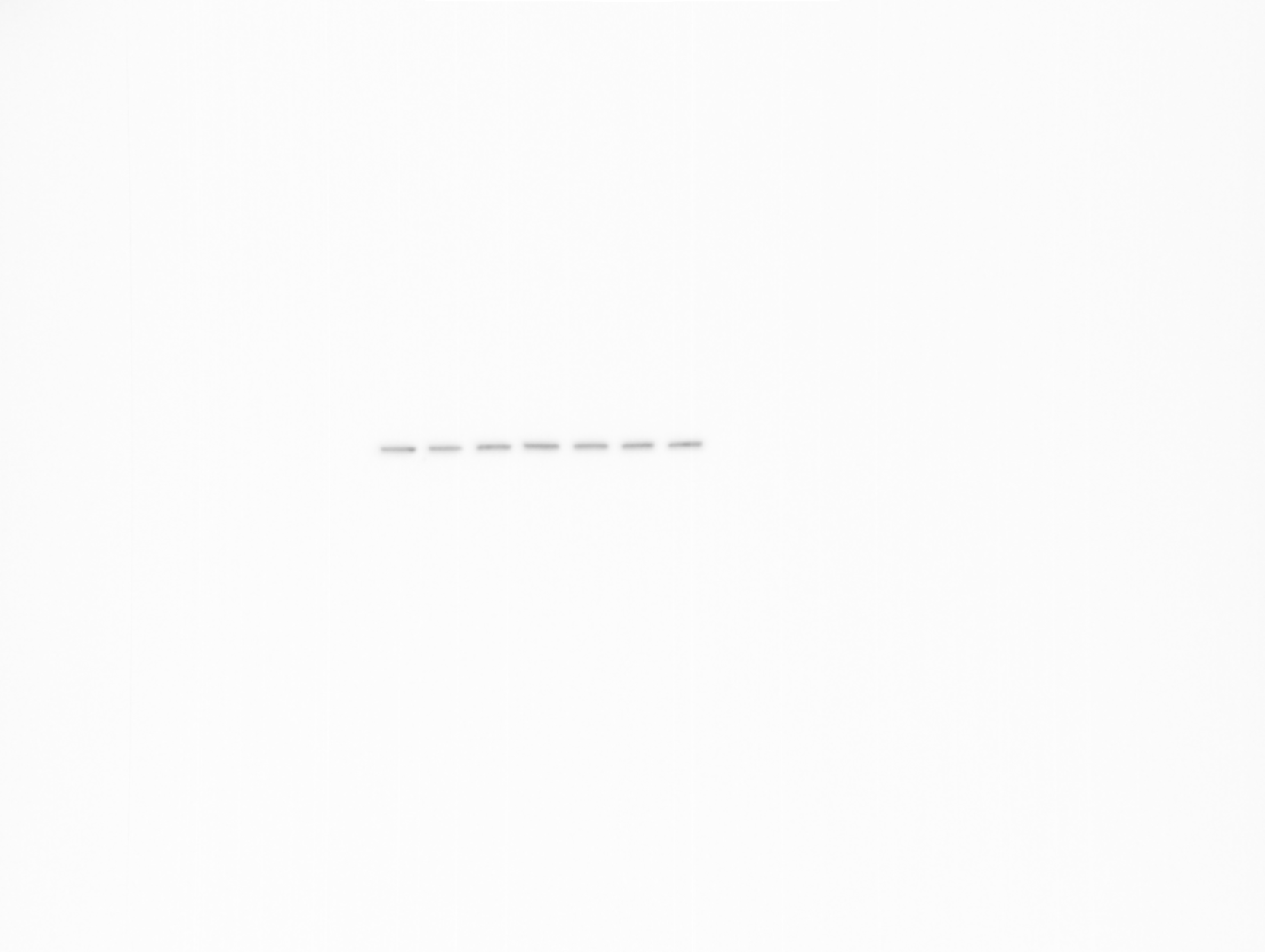

Supplement: Figure 1—source data 2. [file elife-100747-fig1-data2.zip › Figure 1 - Source Data 2 (original western files)/hsp90/S2F7-1118-155148_pub.tif]

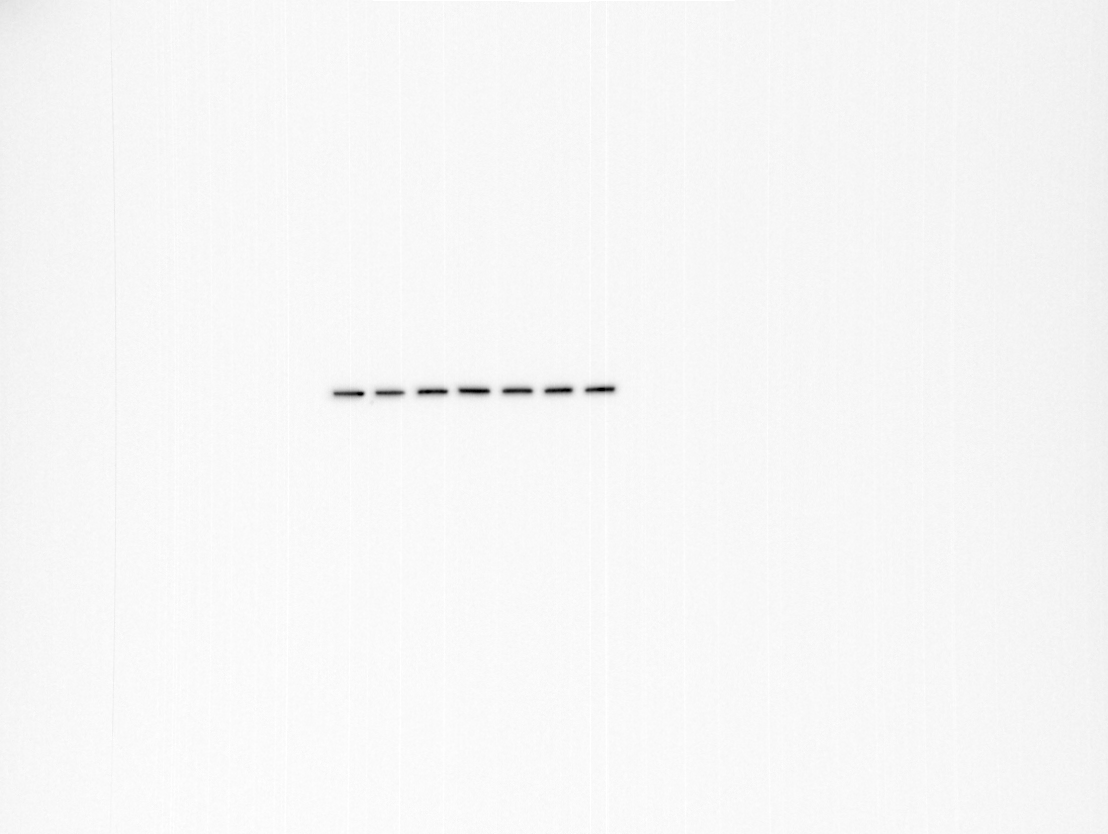

Supplement: Figure 1—source data 2. [file elife-100747-fig1-data2.zip › Figure 1 - Source Data 2 (original western files)/hsp90/S2F8-1118-155149.tif]

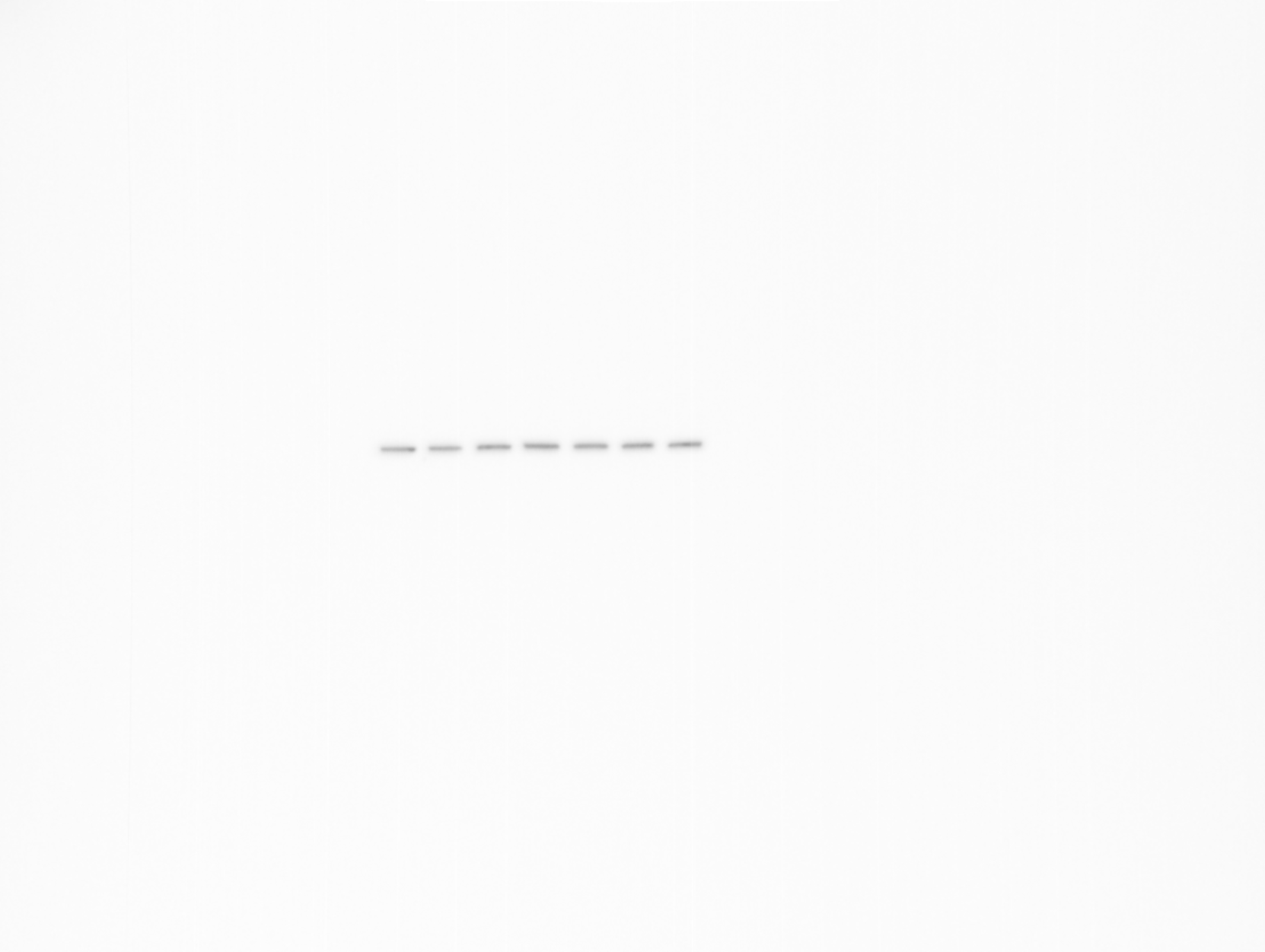

Supplement: Figure 1—source data 2. [file elife-100747-fig1-data2.zip › Figure 1 - Source Data 2 (original western files)/hsp90/S2F8-1118-155149_pub.tif]

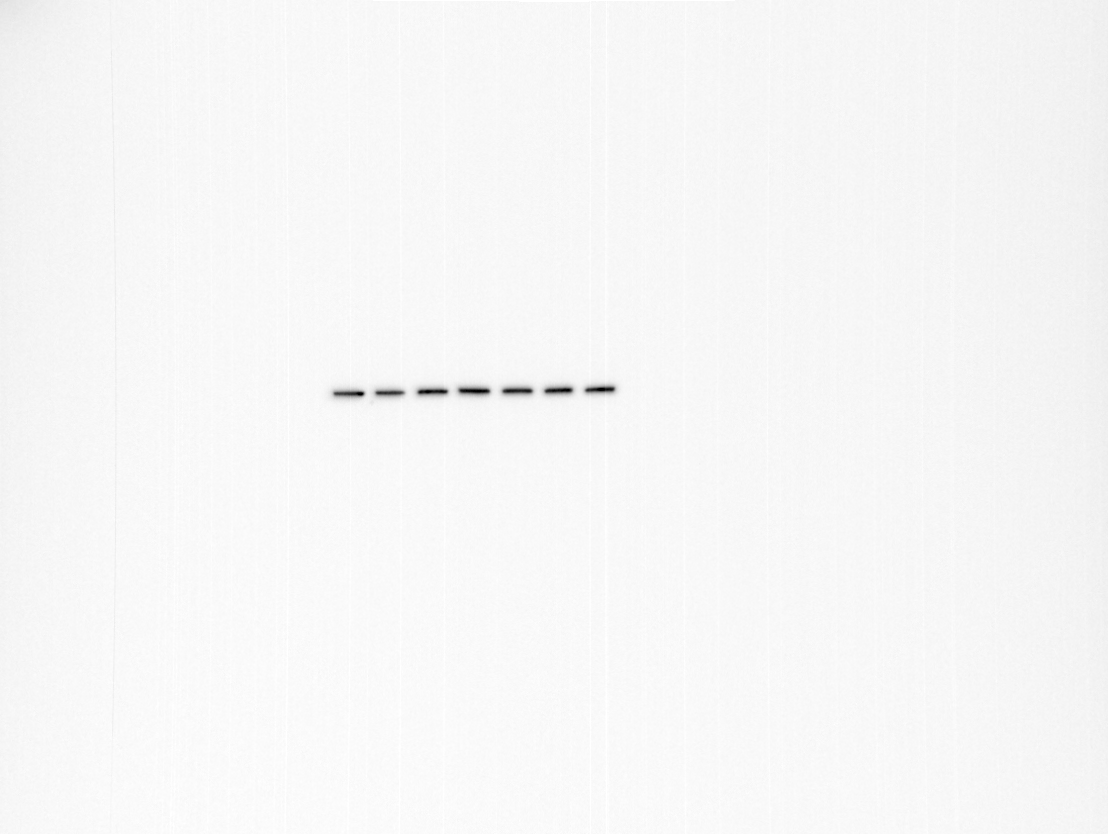

Supplement: Figure 1—source data 2. [file elife-100747-fig1-data2.zip › Figure 1 - Source Data 2 (original western files)/hsp90/S2F9-1118-155151.tif]

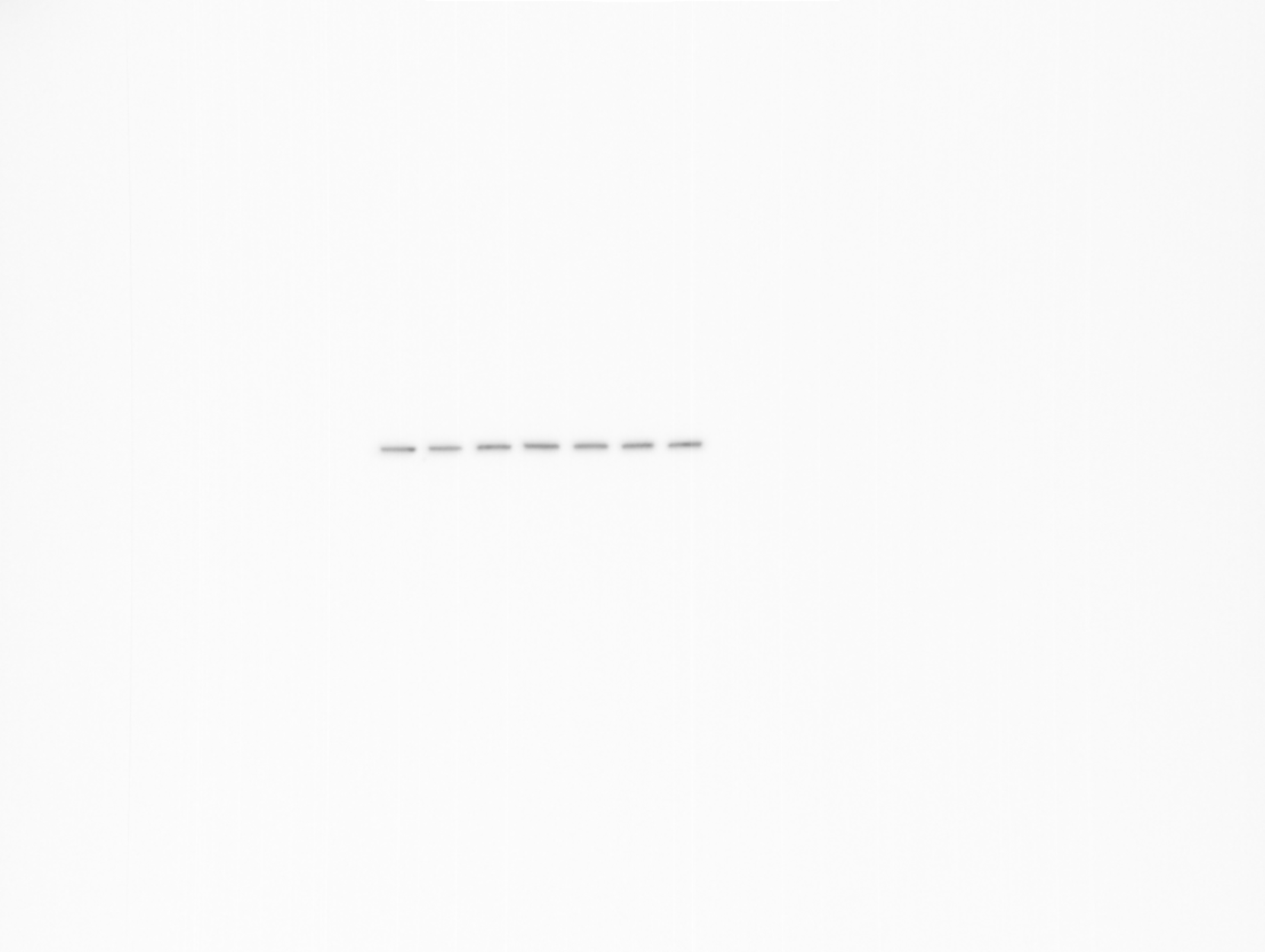

Supplement: Figure 1—source data 2. [file elife-100747-fig1-data2.zip › Figure 1 - Source Data 2 (original western files)/hsp90/S2F9-1118-155151_pub.tif]

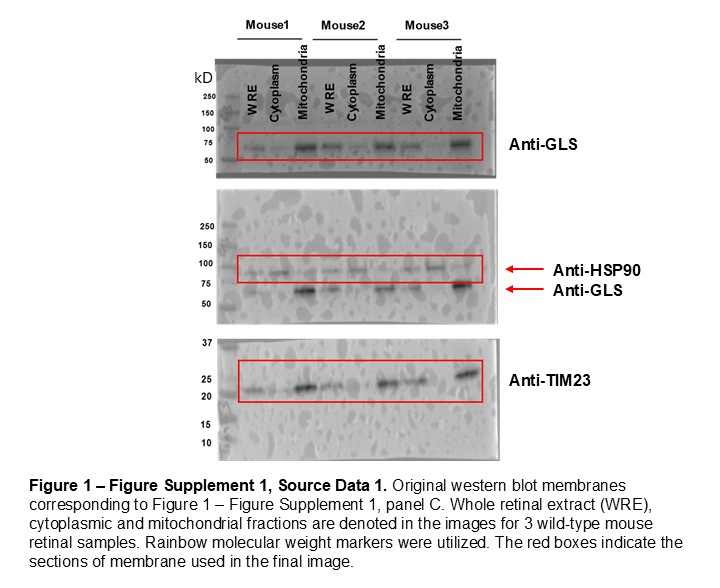

Supplement: Figure 1—figure supplement 1—source data 1. — Whole retinal extract (WRE), cytoplasmic and mitochondrial fractions are denoted in the images for three wild-type mouse retinal samples. Rainbow molecular weight markers were utilized. The red boxes indicate the sections of membrane used in the final image. [file elife-100747-fig1-figsupp1-data1.zip › Figure 1 - Figure Supplement 1 - Source Data 1 (annotated western file)/Figure 1 - Figure Supplement 1 - Source Data 1.tif]

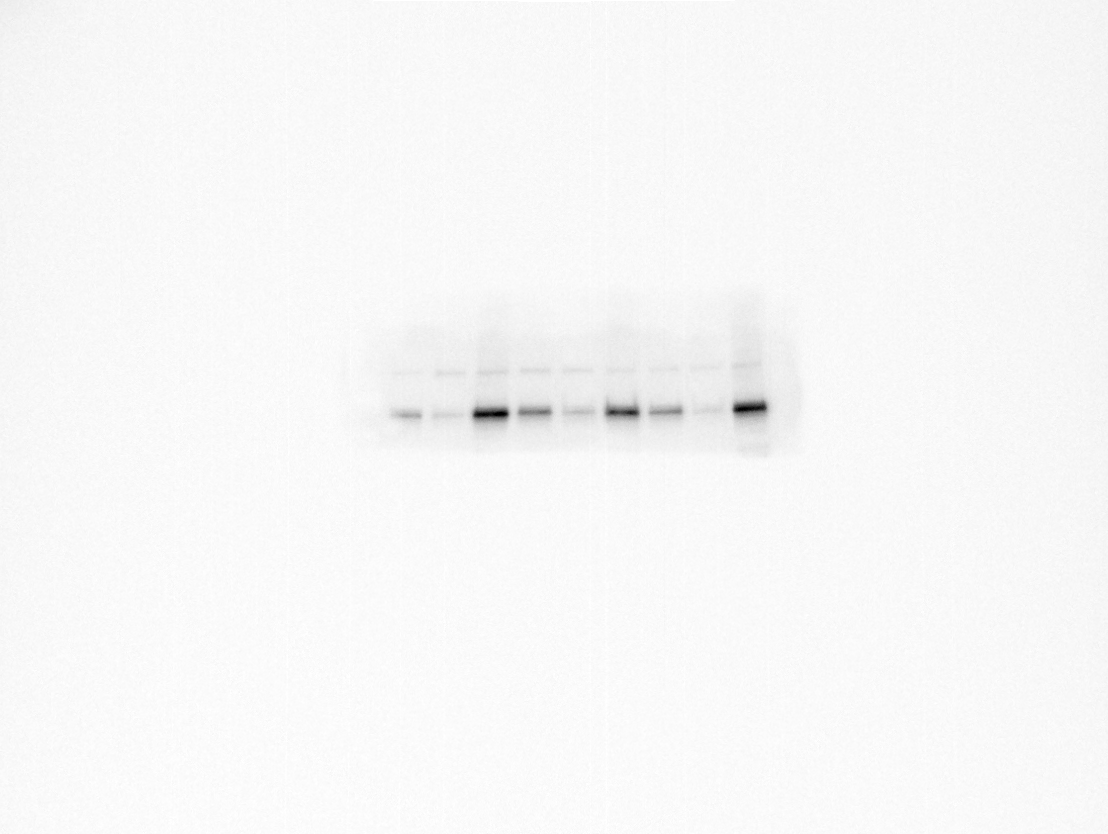

Supplement: Figure 1—figure supplement 1—source data 2. [file elife-100747-fig1-figsupp1-data2.zip › Figure 1 - Figure Supplement 1 - Source Data 2 (original western files)/gls_Proteintech_pico/2022-0422-161107.tif]

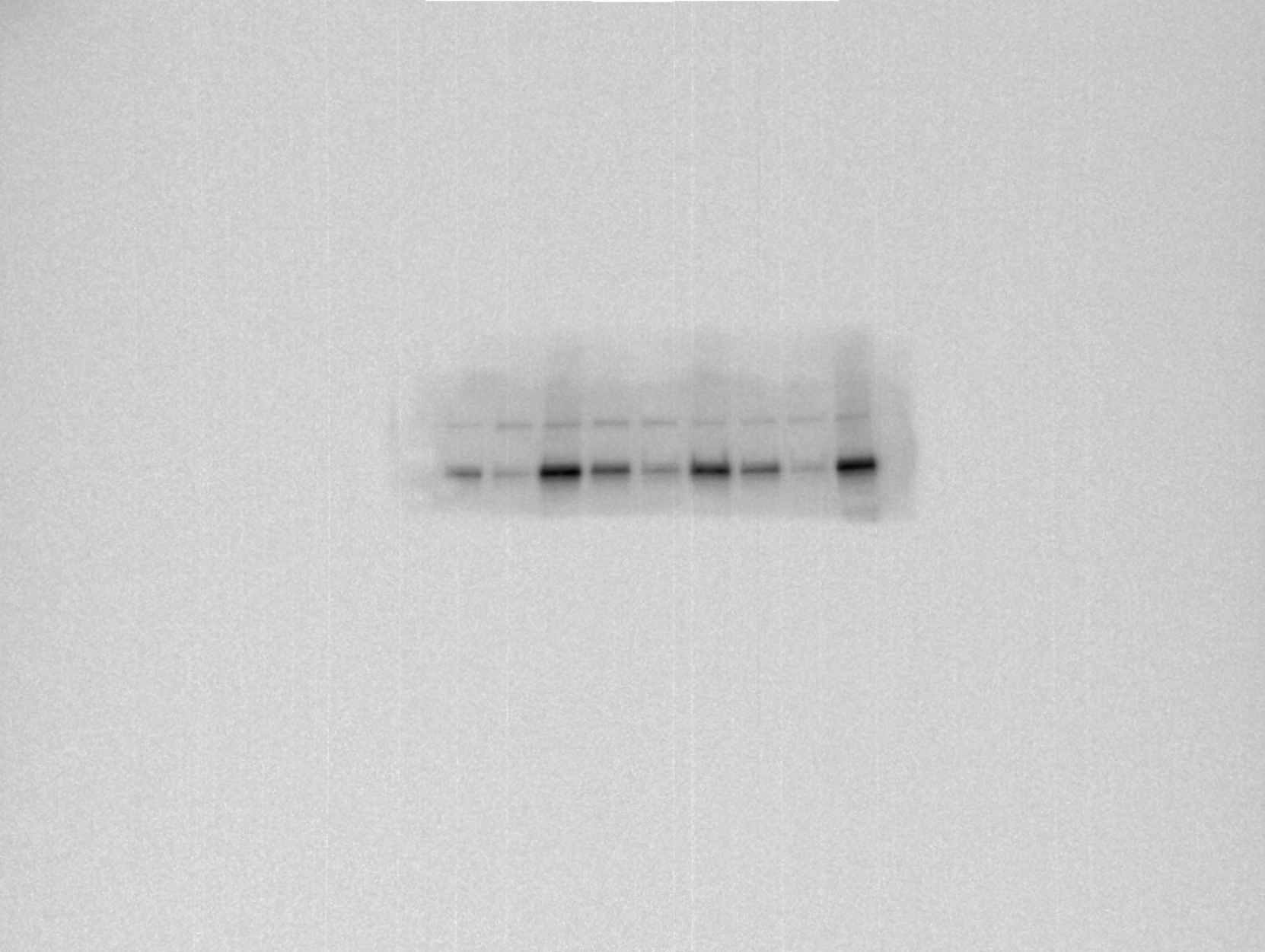

Supplement: Figure 1—figure supplement 1—source data 2. [file elife-100747-fig1-figsupp1-data2.zip › Figure 1 - Figure Supplement 1 - Source Data 2 (original western files)/gls_Proteintech_pico/2022-0422-161107_pub.tif]

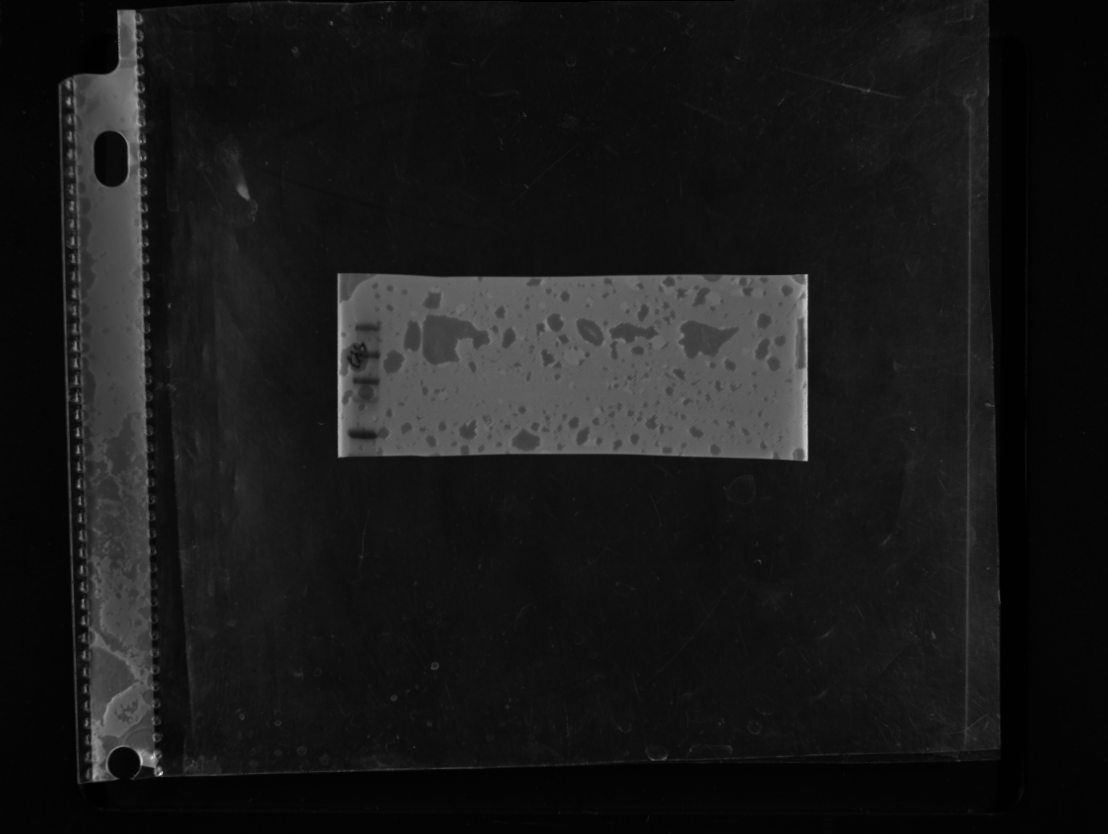

Supplement: Figure 1—figure supplement 1—source data 2. [file elife-100747-fig1-figsupp1-data2.zip › Figure 1 - Figure Supplement 1 - Source Data 2 (original western files)/gls_Proteintech_pico/2022-0422-161108.tif]

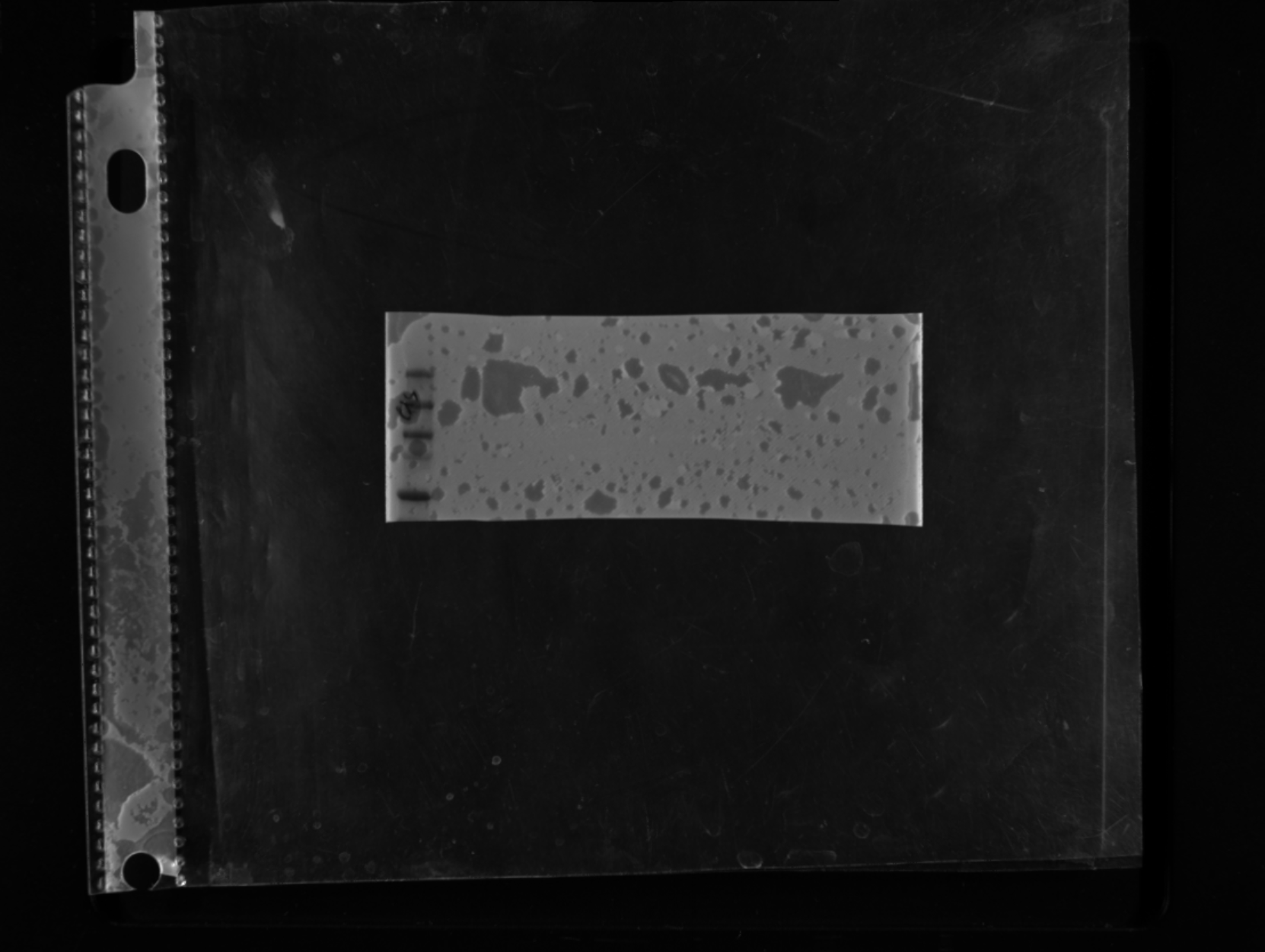

Supplement: Figure 1—figure supplement 1—source data 2. [file elife-100747-fig1-figsupp1-data2.zip › Figure 1 - Figure Supplement 1 - Source Data 2 (original western files)/gls_Proteintech_pico/2022-0422-161108_pub.tif]

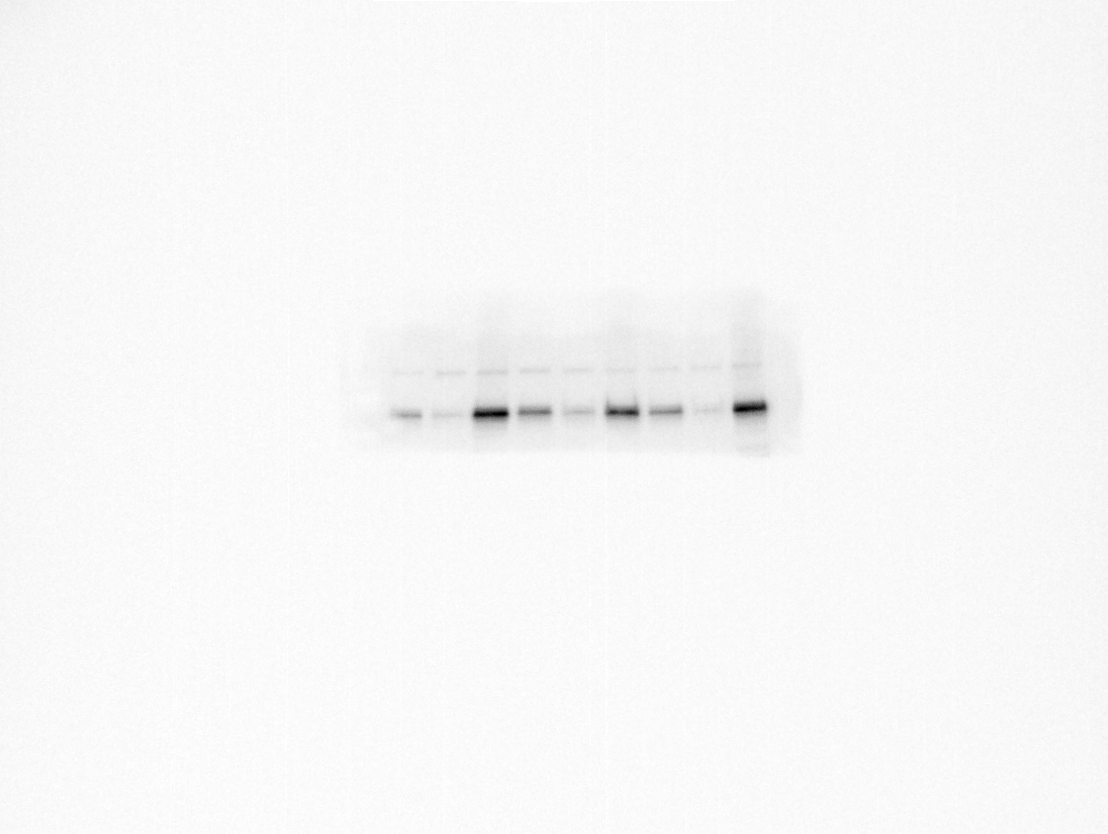

Supplement: Figure 1—figure supplement 1—source data 2. [file elife-100747-fig1-figsupp1-data2.zip › Figure 1 - Figure Supplement 1 - Source Data 2 (original western files)/gls_Proteintech_pico/2022-0422-161110.tif]

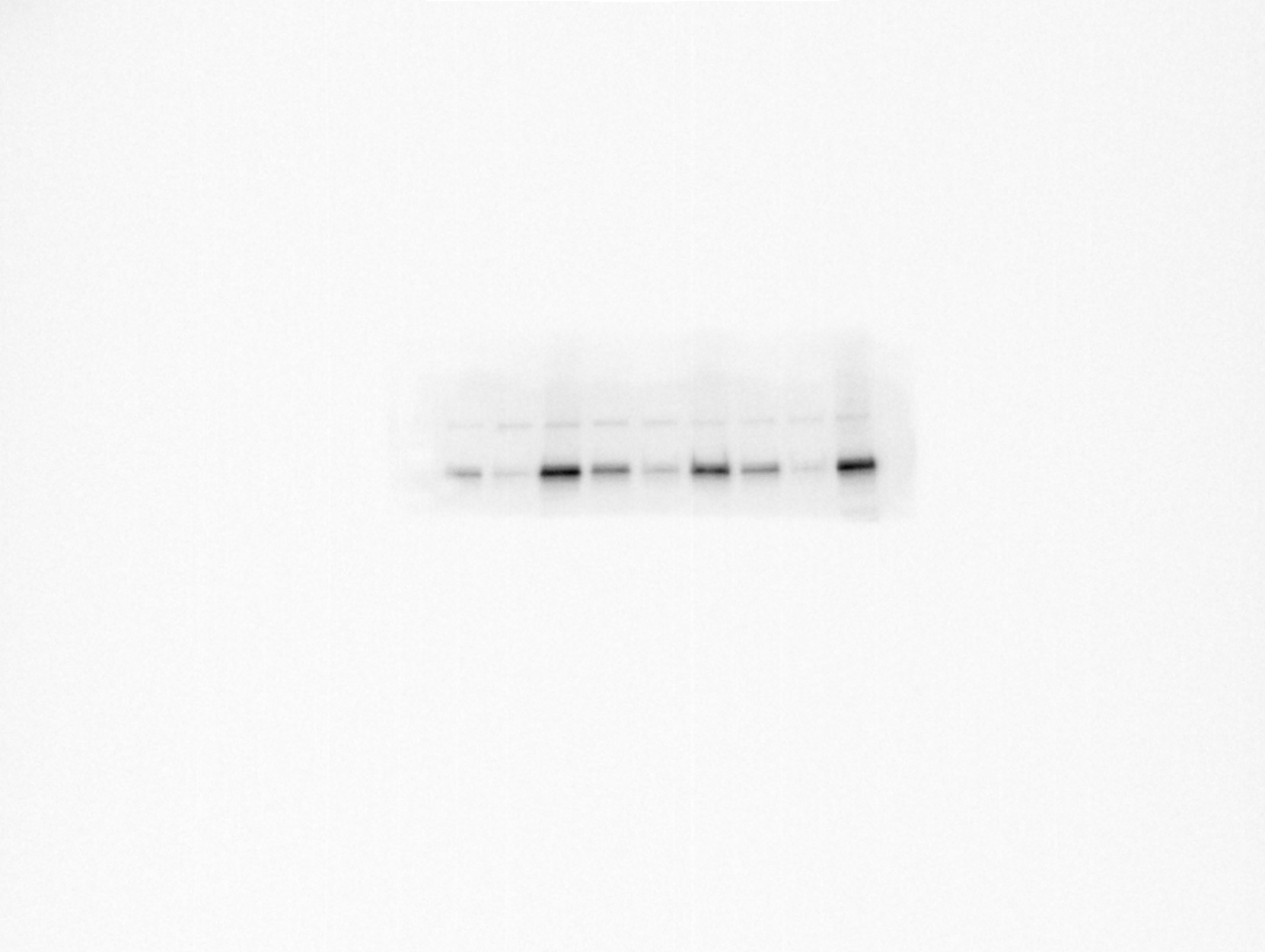

Supplement: Figure 1—figure supplement 1—source data 2. [file elife-100747-fig1-figsupp1-data2.zip › Figure 1 - Figure Supplement 1 - Source Data 2 (original western files)/gls_Proteintech_pico/2022-0422-161110_pub.tif]

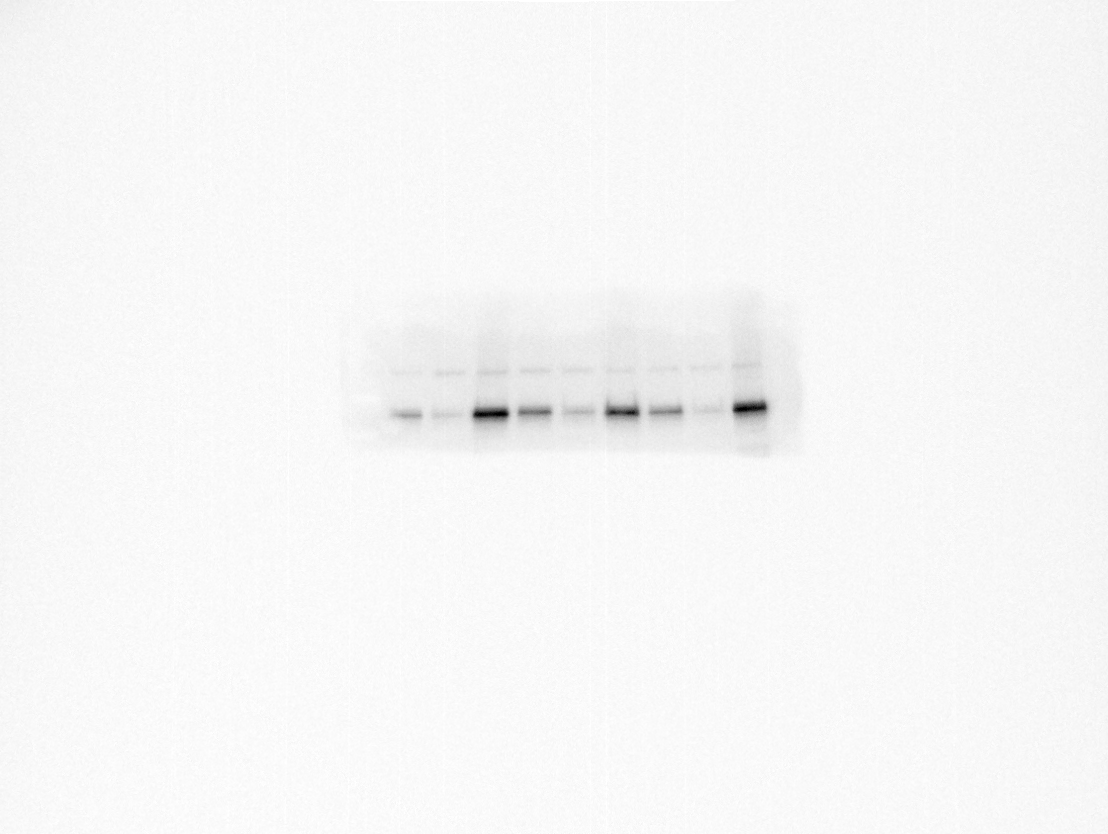

Supplement: Figure 1—figure supplement 1—source data 2. [file elife-100747-fig1-figsupp1-data2.zip › Figure 1 - Figure Supplement 1 - Source Data 2 (original western files)/gls_Proteintech_pico/S1F1-0422-161111.tif]

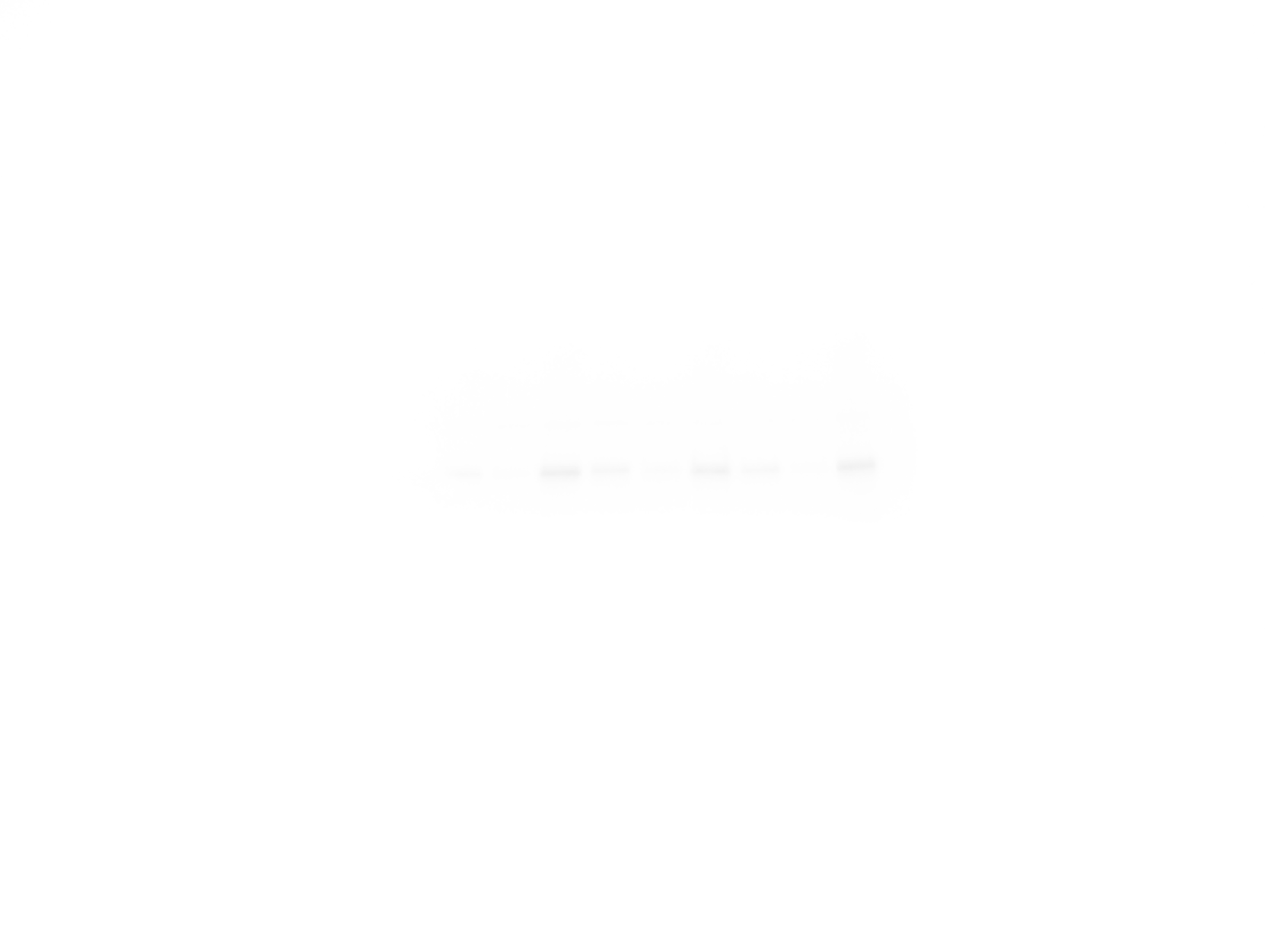

Supplement: Figure 1—figure supplement 1—source data 2. [file elife-100747-fig1-figsupp1-data2.zip › Figure 1 - Figure Supplement 1 - Source Data 2 (original western files)/gls_Proteintech_pico/S1F1-0422-161111_pub.tif]

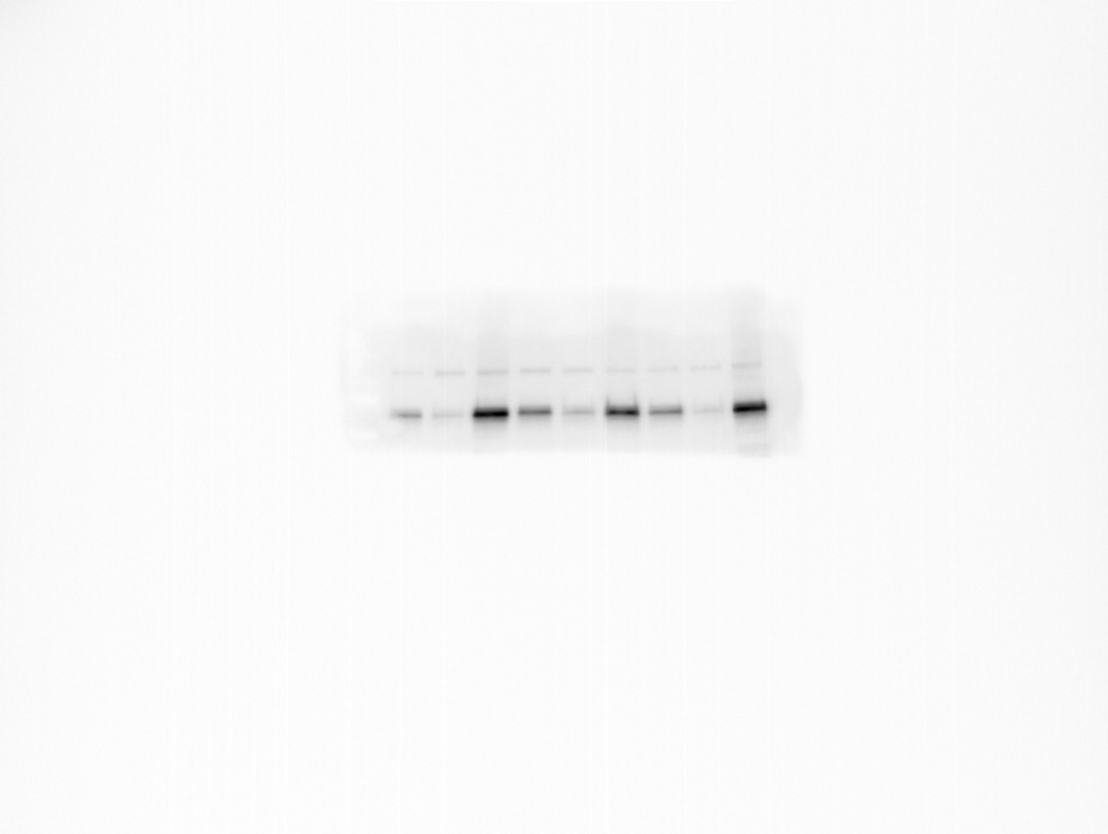

Supplement: Figure 1—figure supplement 1—source data 2. [file elife-100747-fig1-figsupp1-data2.zip › Figure 1 - Figure Supplement 1 - Source Data 2 (original western files)/gls_Proteintech_pico/S1F10-0422-161125.tif]

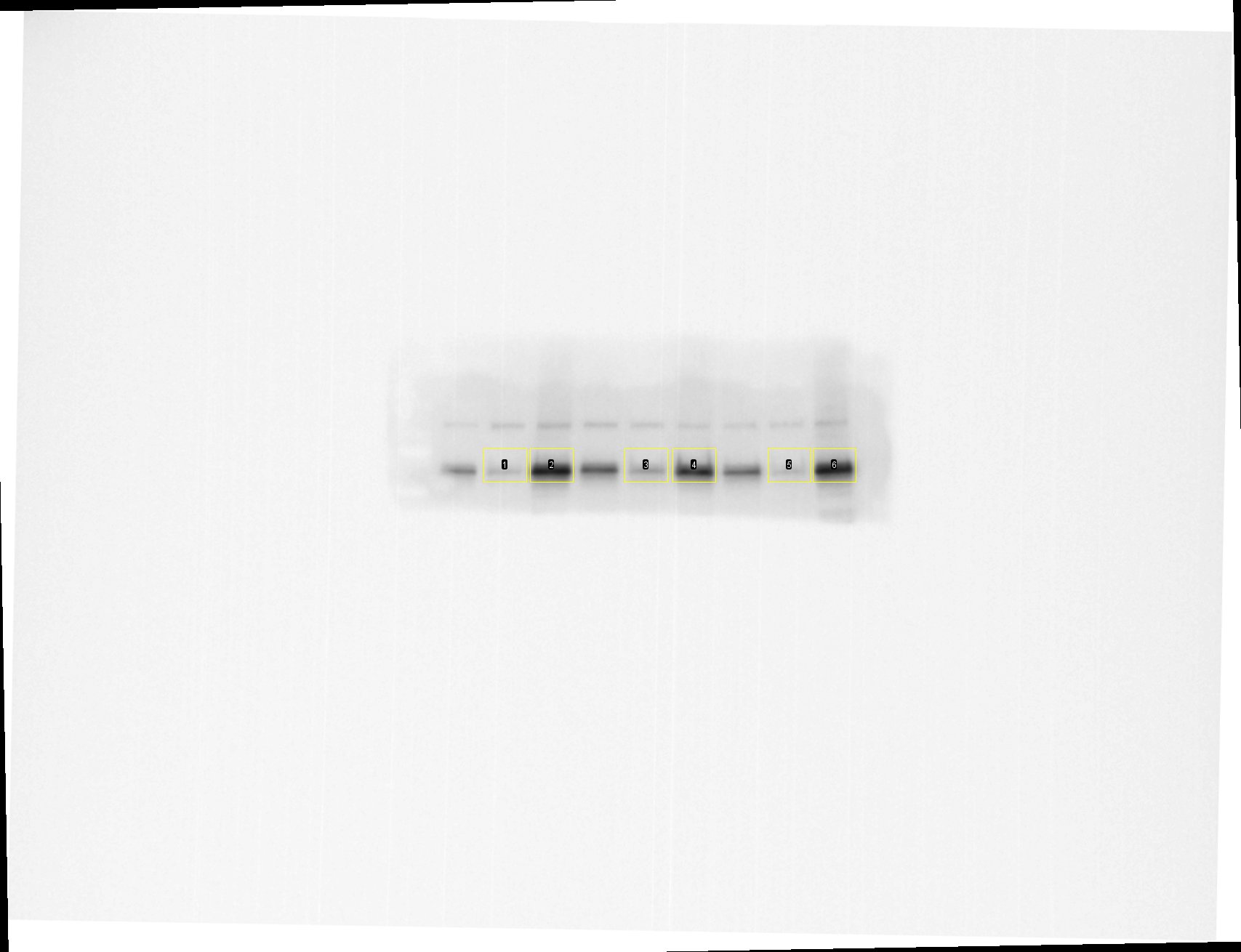

Supplement: Figure 1—figure supplement 1—source data 2. [file elife-100747-fig1-figsupp1-data2.zip › Figure 1 - Figure Supplement 1 - Source Data 2 (original western files)/gls_Proteintech_pico/S1F10-0422-161125_pub.jpg]

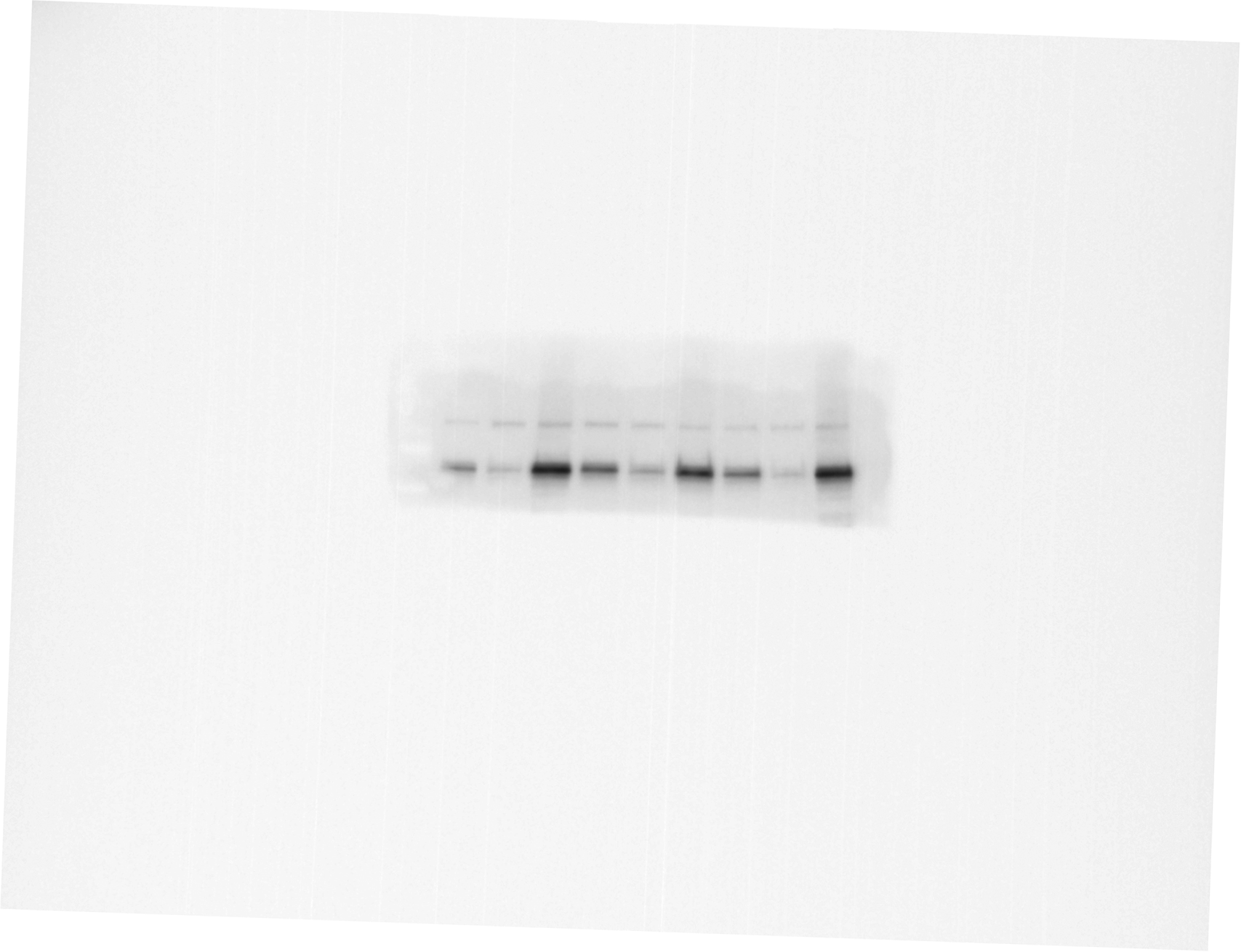

Supplement: Figure 1—figure supplement 1—source data 2. [file elife-100747-fig1-figsupp1-data2.zip › Figure 1 - Figure Supplement 1 - Source Data 2 (original western files)/gls_Proteintech_pico/S1F10-0422-161125_pub.tif]

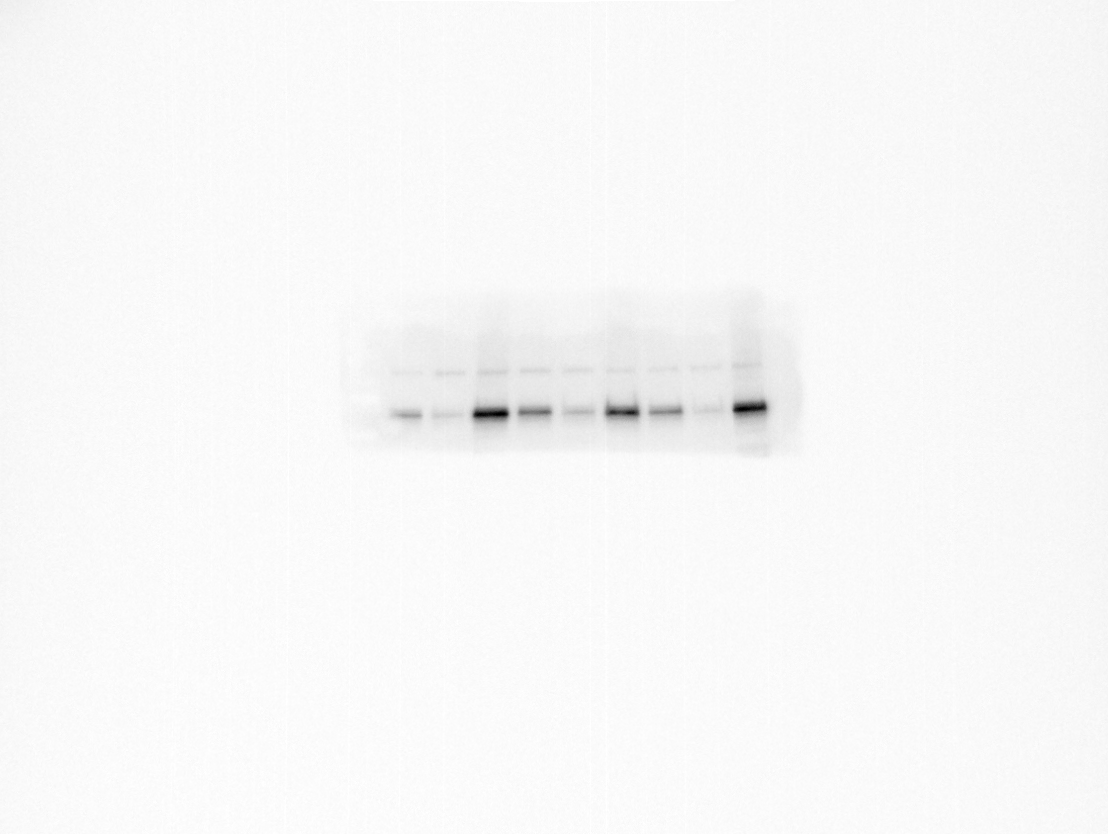

Supplement: Figure 1—figure supplement 1—source data 2. [file elife-100747-fig1-figsupp1-data2.zip › Figure 1 - Figure Supplement 1 - Source Data 2 (original western files)/gls_Proteintech_pico/S1F2-0422-161113.tif]

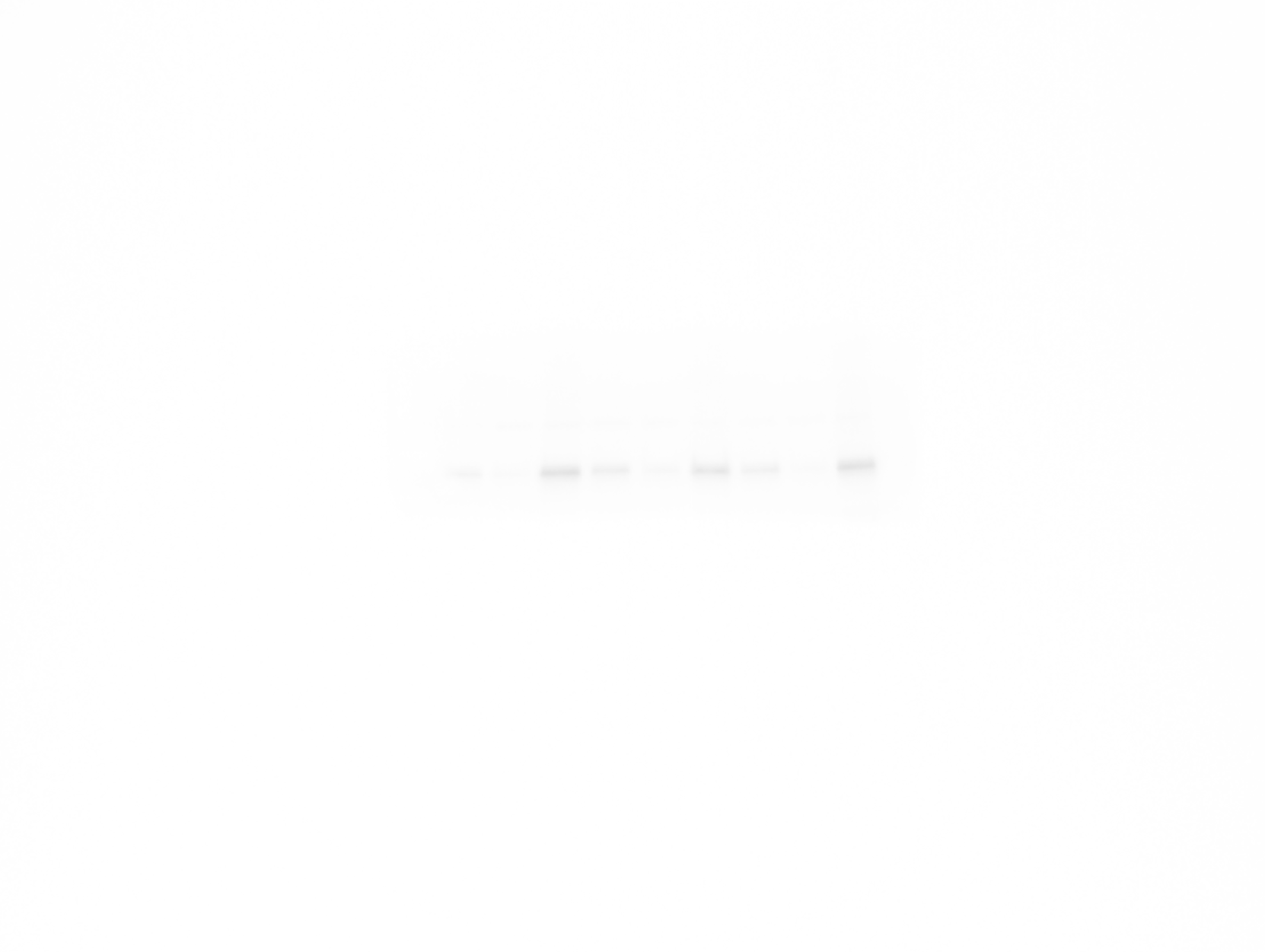

Supplement: Figure 1—figure supplement 1—source data 2. [file elife-100747-fig1-figsupp1-data2.zip › Figure 1 - Figure Supplement 1 - Source Data 2 (original western files)/gls_Proteintech_pico/S1F2-0422-161113_pub.tif]

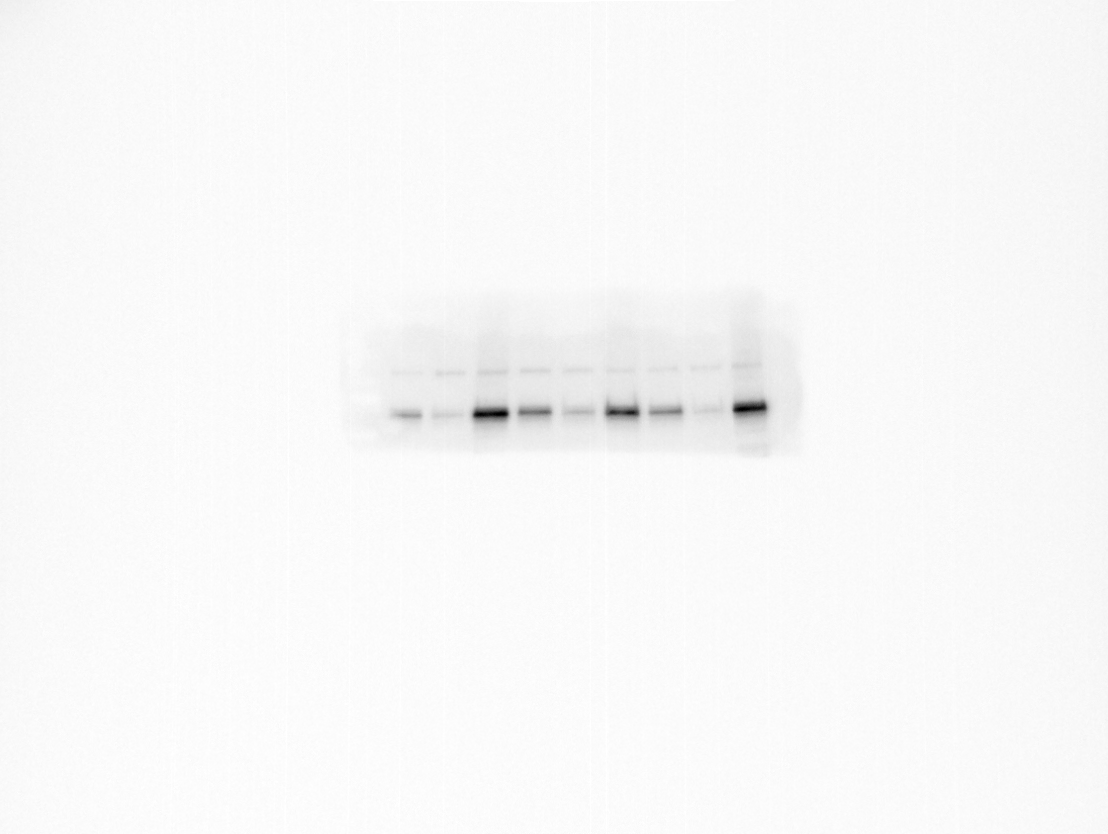

Supplement: Figure 1—figure supplement 1—source data 2. [file elife-100747-fig1-figsupp1-data2.zip › Figure 1 - Figure Supplement 1 - Source Data 2 (original western files)/gls_Proteintech_pico/S1F3-0422-161114.tif]

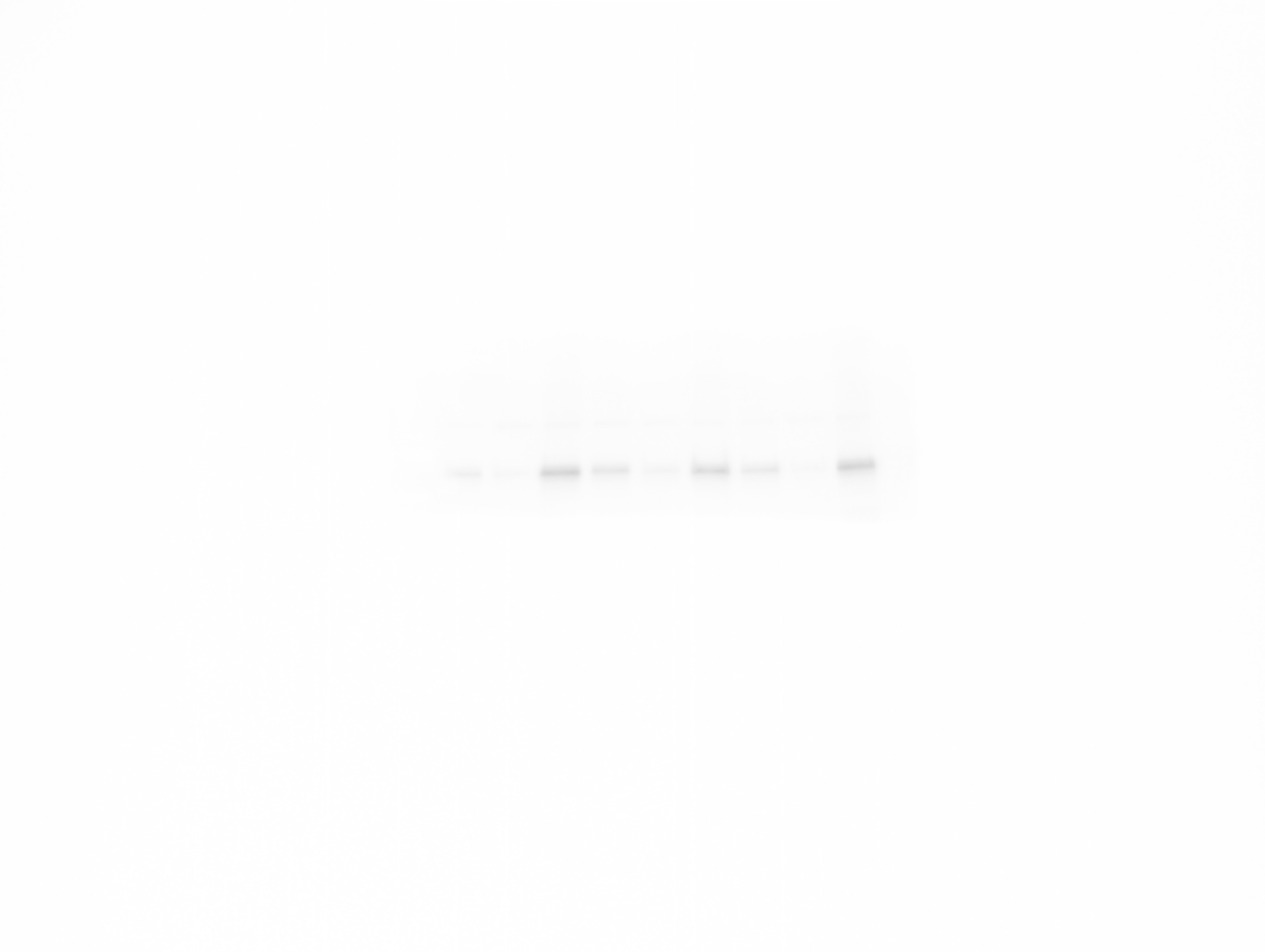

Supplement: Figure 1—figure supplement 1—source data 2. [file elife-100747-fig1-figsupp1-data2.zip › Figure 1 - Figure Supplement 1 - Source Data 2 (original western files)/gls_Proteintech_pico/S1F3-0422-161114_pub.tif]

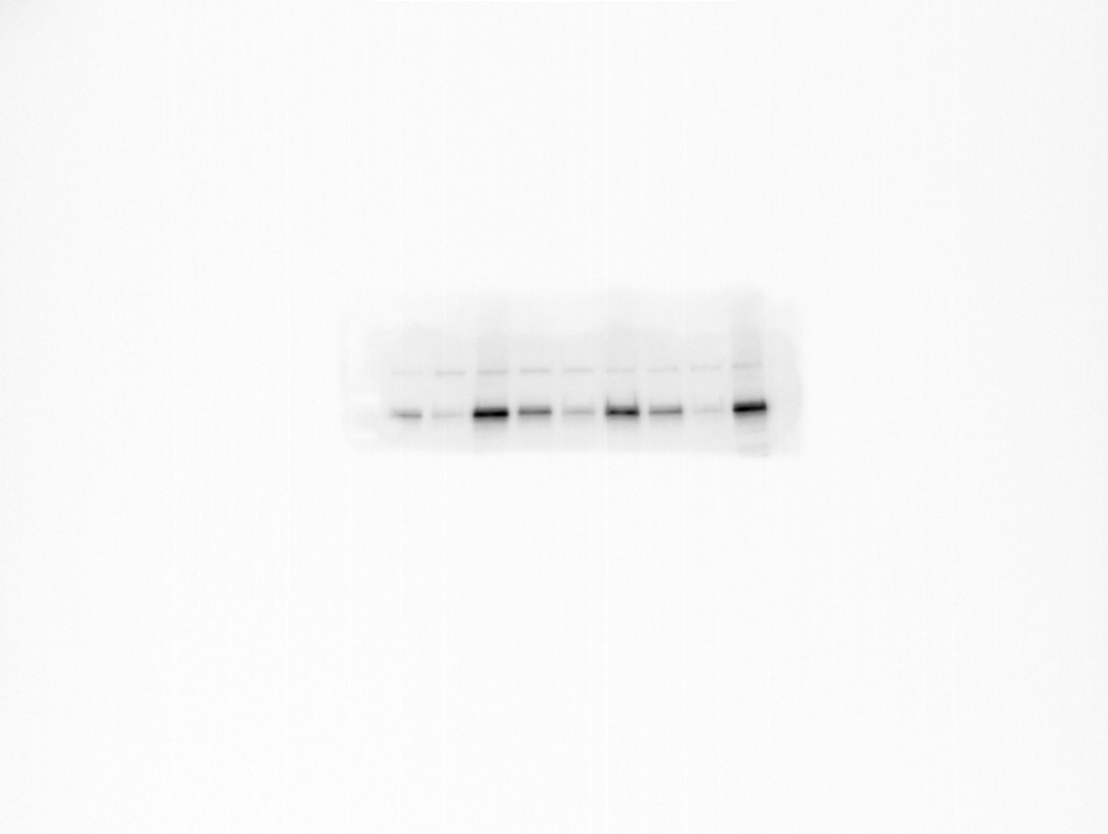

Supplement: Figure 1—figure supplement 1—source data 2. [file elife-100747-fig1-figsupp1-data2.zip › Figure 1 - Figure Supplement 1 - Source Data 2 (original western files)/gls_Proteintech_pico/S1F4-0422-161116.tif]

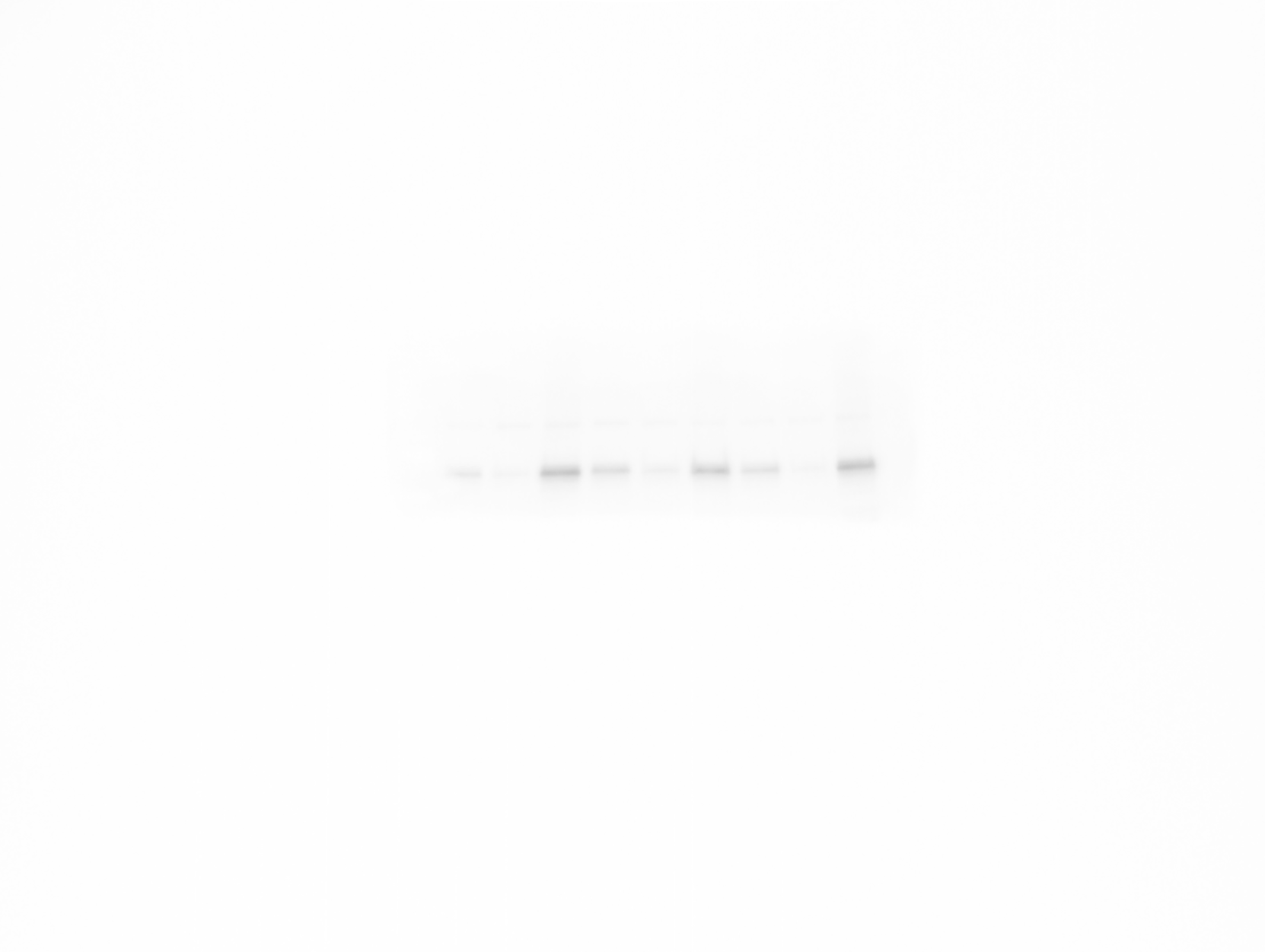

Supplement: Figure 1—figure supplement 1—source data 2. [file elife-100747-fig1-figsupp1-data2.zip › Figure 1 - Figure Supplement 1 - Source Data 2 (original western files)/gls_Proteintech_pico/S1F4-0422-161116_pub.tif]

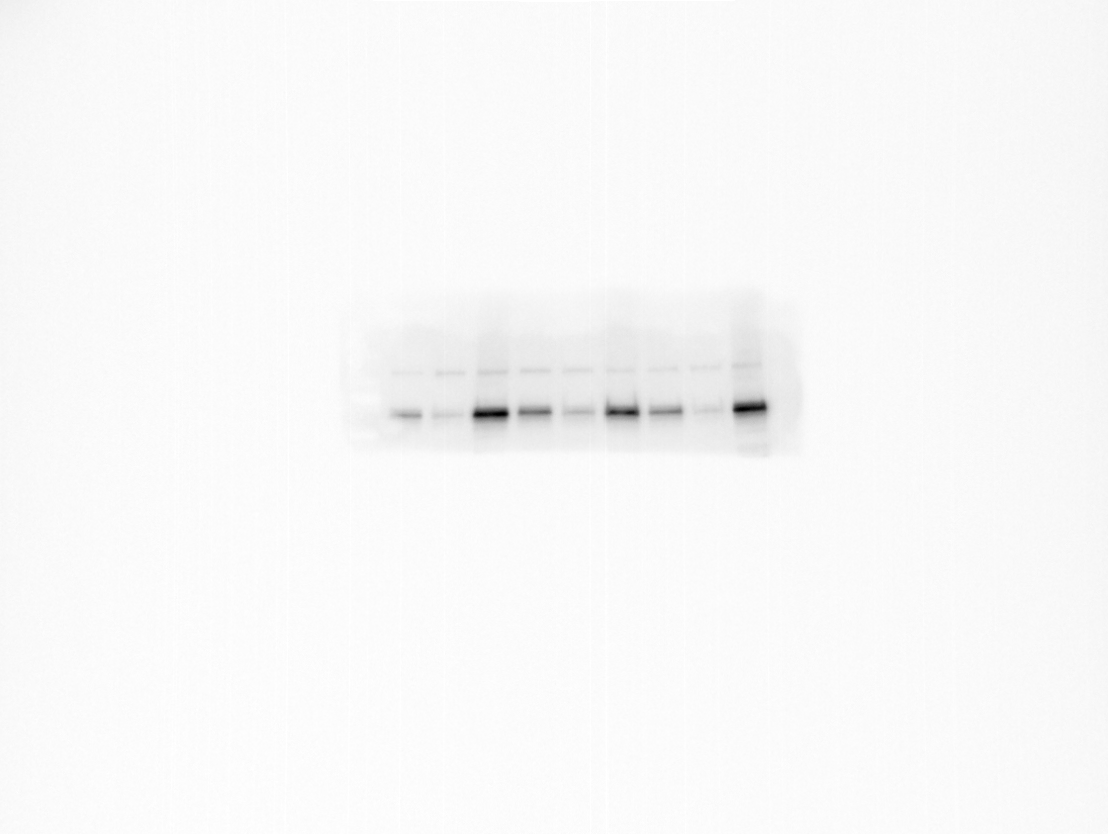

Supplement: Figure 1—figure supplement 1—source data 2. [file elife-100747-fig1-figsupp1-data2.zip › Figure 1 - Figure Supplement 1 - Source Data 2 (original western files)/gls_Proteintech_pico/S1F5-0422-161118.tif]

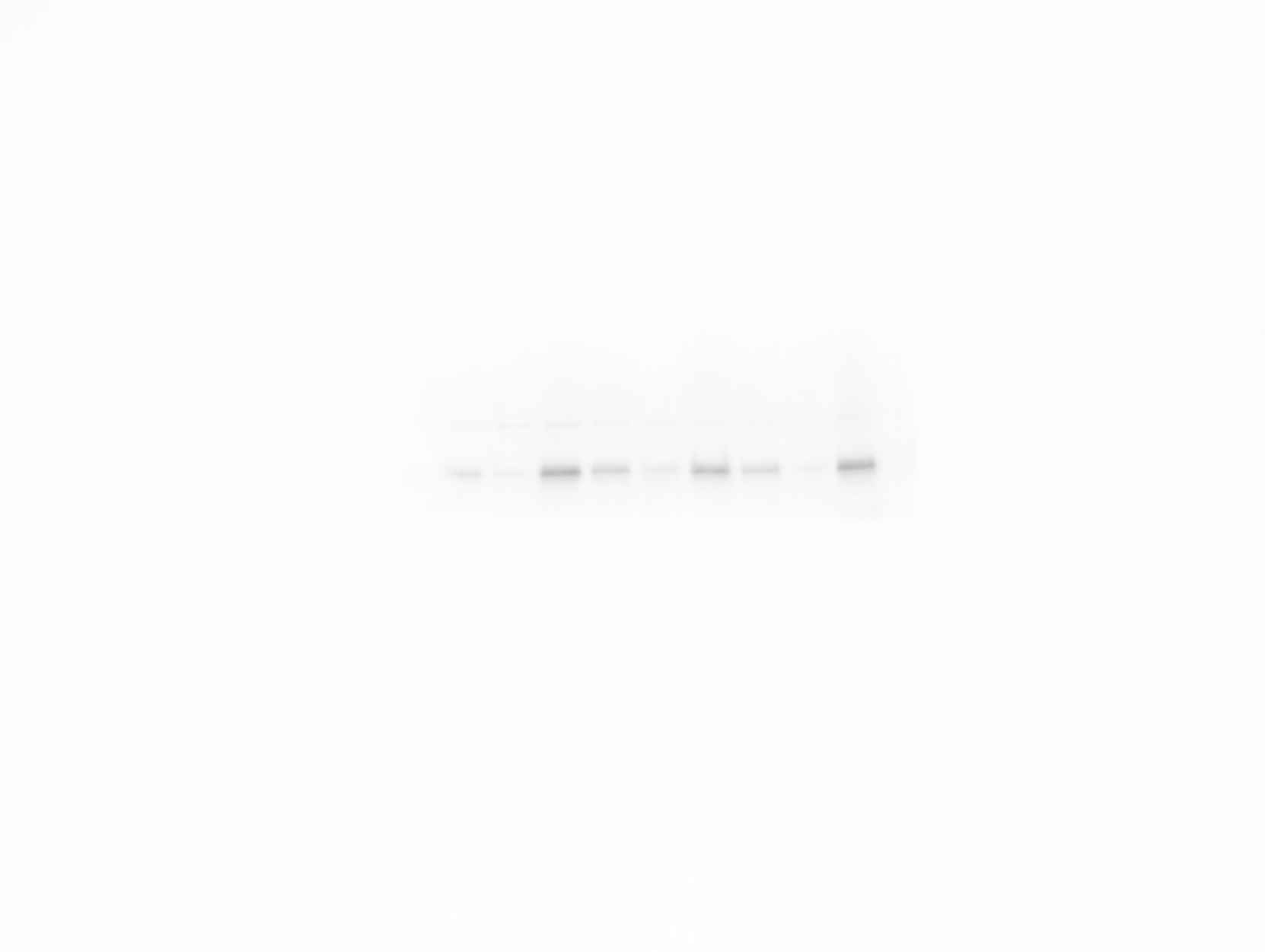

Supplement: Figure 1—figure supplement 1—source data 2. [file elife-100747-fig1-figsupp1-data2.zip › Figure 1 - Figure Supplement 1 - Source Data 2 (original western files)/gls_Proteintech_pico/S1F5-0422-161118_pub.tif]

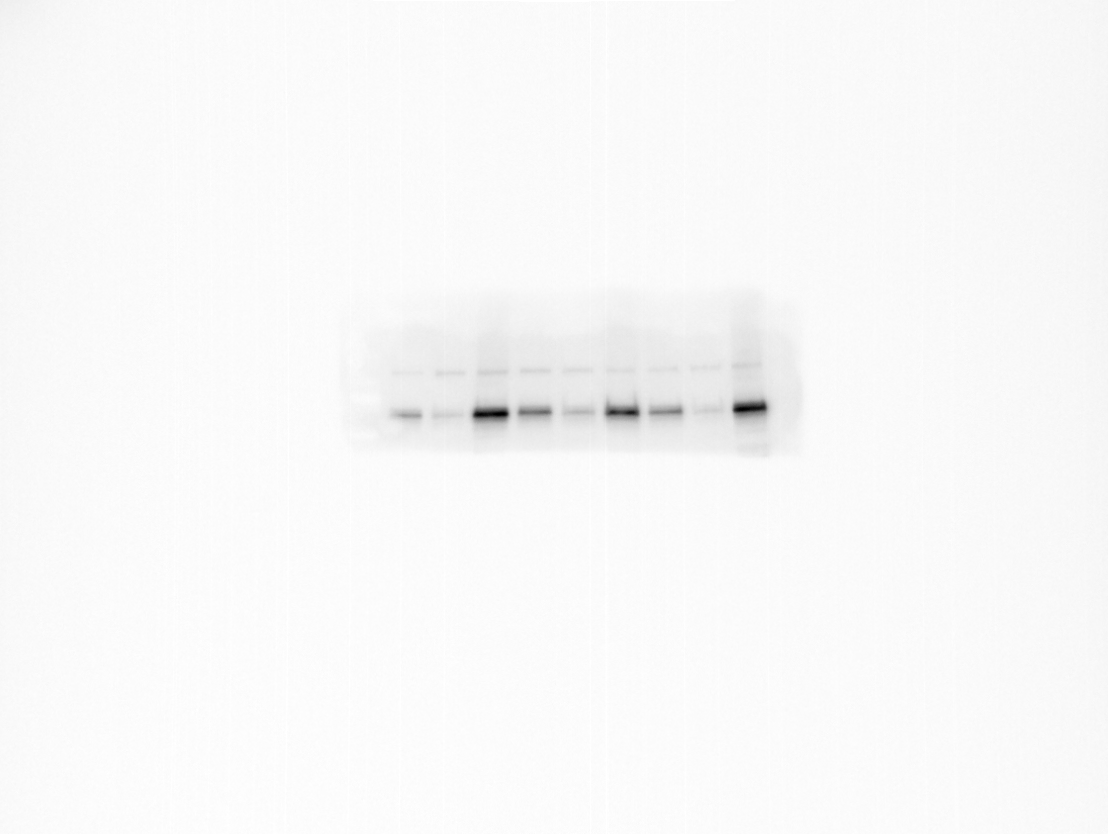

Supplement: Figure 1—figure supplement 1—source data 2. [file elife-100747-fig1-figsupp1-data2.zip › Figure 1 - Figure Supplement 1 - Source Data 2 (original western files)/gls_Proteintech_pico/S1F6-0422-161119.tif]

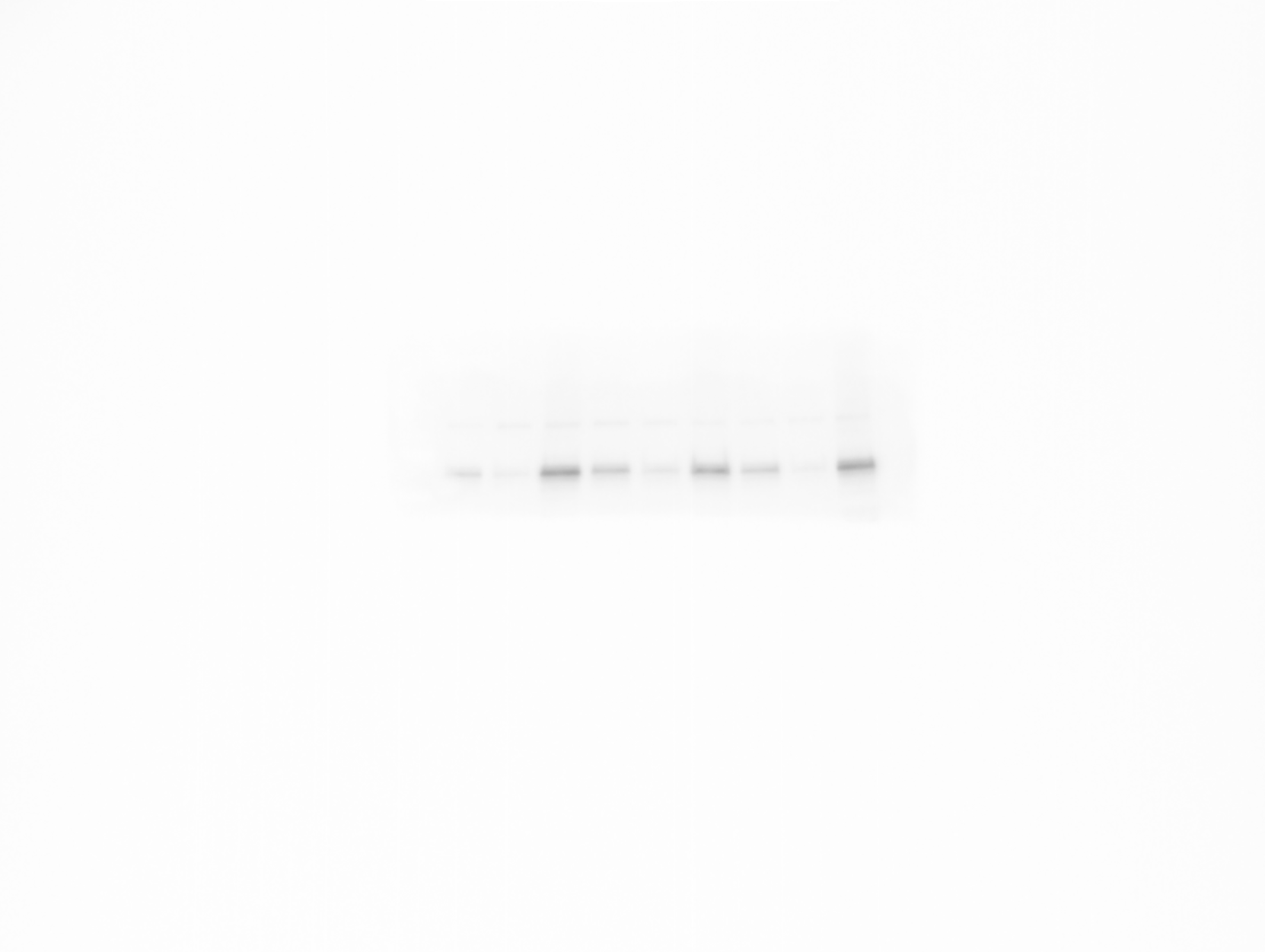

Supplement: Figure 1—figure supplement 1—source data 2. [file elife-100747-fig1-figsupp1-data2.zip › Figure 1 - Figure Supplement 1 - Source Data 2 (original western files)/gls_Proteintech_pico/S1F6-0422-161119_pub.tif]

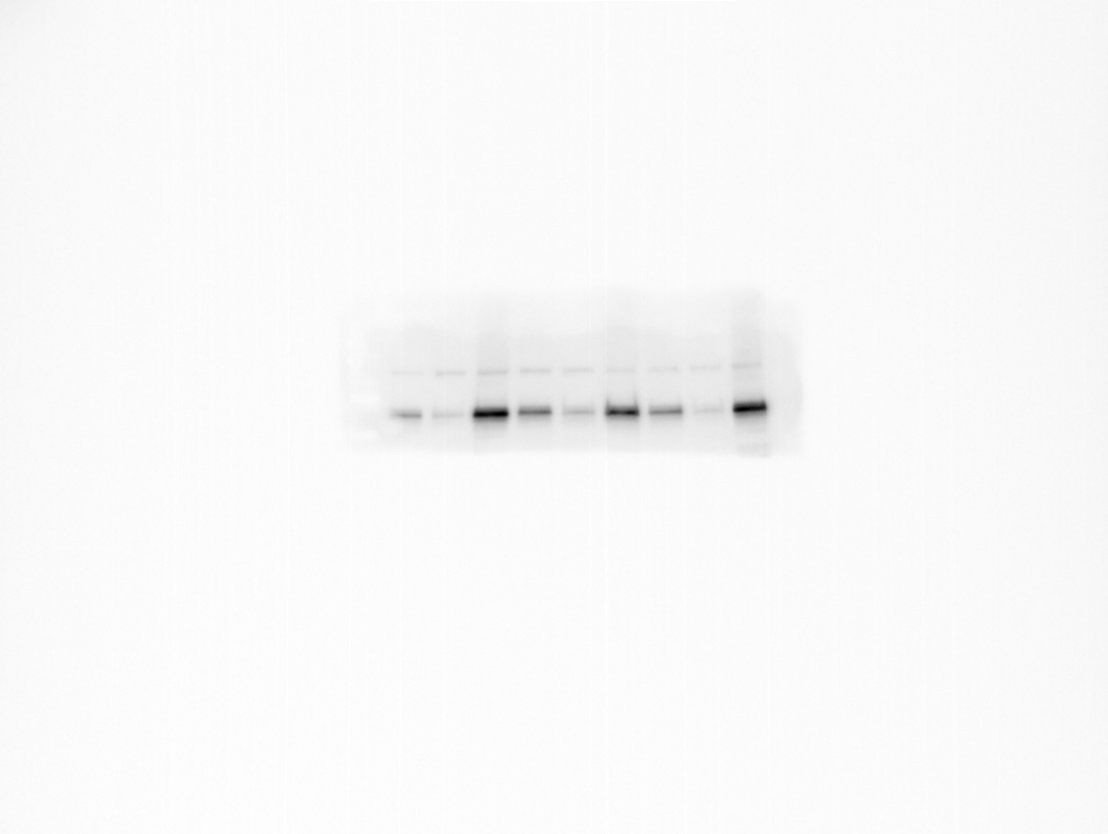

Supplement: Figure 1—figure supplement 1—source data 2. [file elife-100747-fig1-figsupp1-data2.zip › Figure 1 - Figure Supplement 1 - Source Data 2 (original western files)/gls_Proteintech_pico/S1F7-0422-161121.tif]

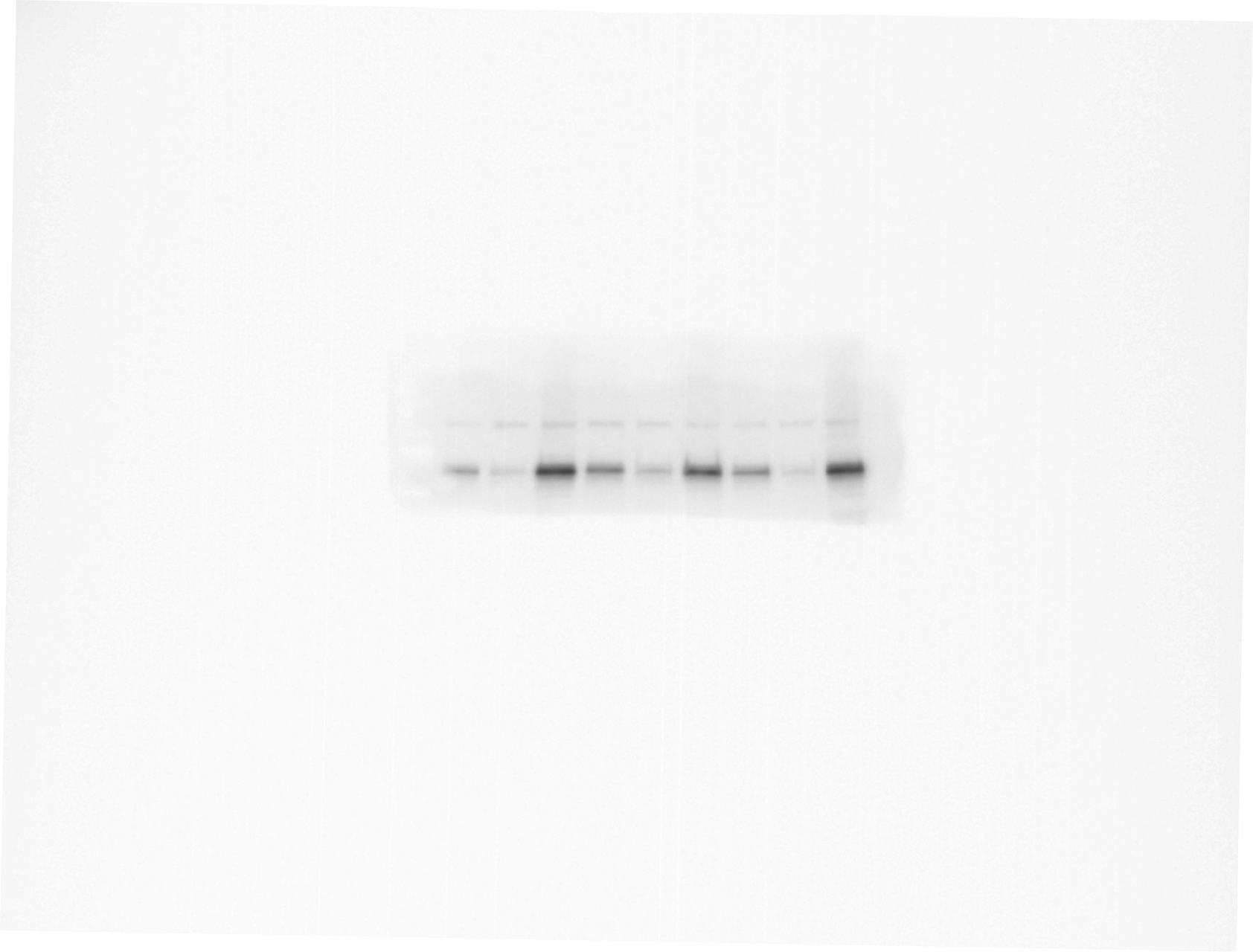

Supplement: Figure 1—figure supplement 1—source data 2. [file elife-100747-fig1-figsupp1-data2.zip › Figure 1 - Figure Supplement 1 - Source Data 2 (original western files)/gls_Proteintech_pico/S1F7-0422-161121_pub.tif]

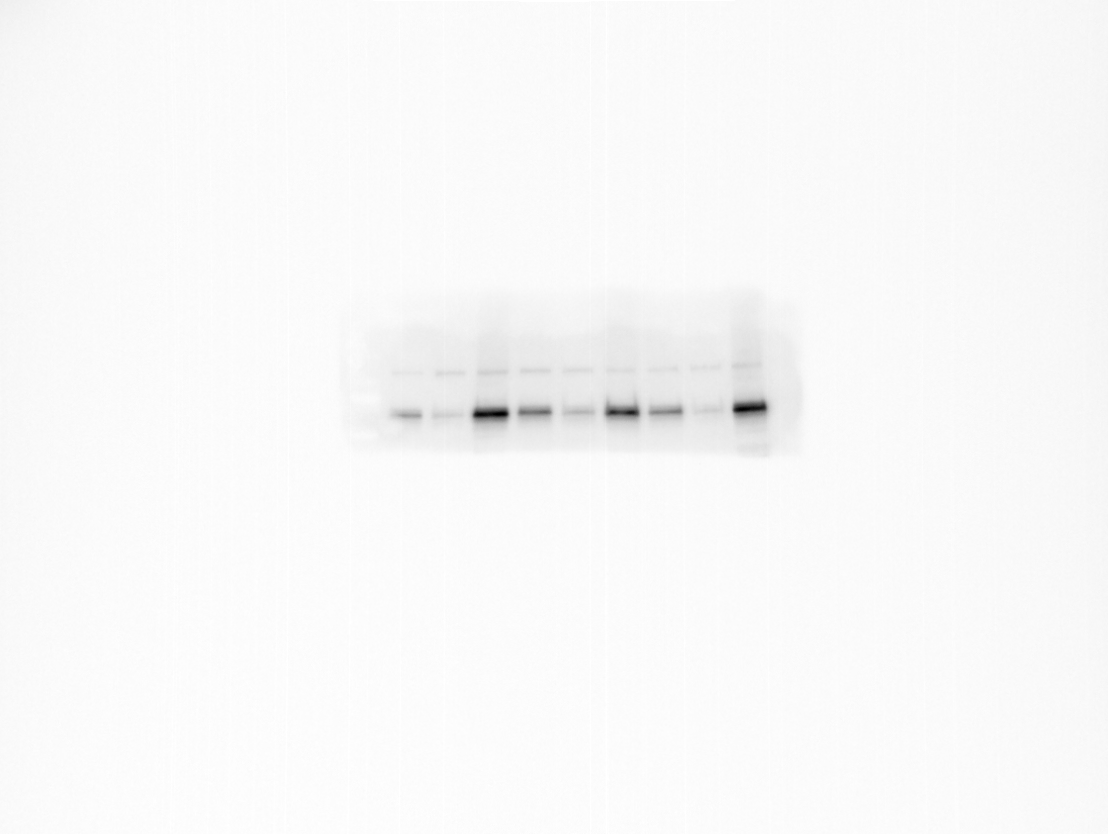

Supplement: Figure 1—figure supplement 1—source data 2. [file elife-100747-fig1-figsupp1-data2.zip › Figure 1 - Figure Supplement 1 - Source Data 2 (original western files)/gls_Proteintech_pico/S1F8-0422-161122.tif]

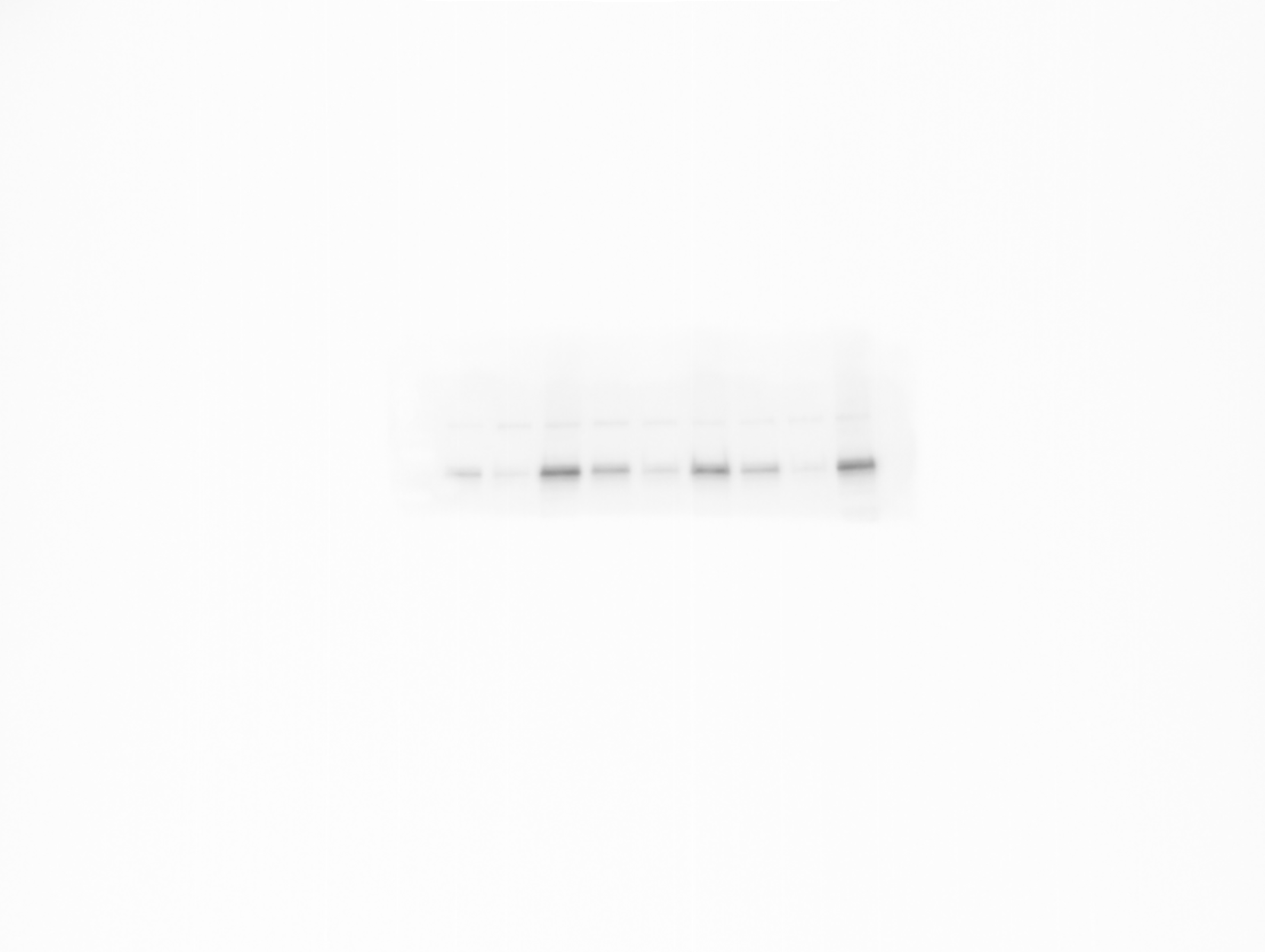

Supplement: Figure 1—figure supplement 1—source data 2. [file elife-100747-fig1-figsupp1-data2.zip › Figure 1 - Figure Supplement 1 - Source Data 2 (original western files)/gls_Proteintech_pico/S1F8-0422-161122_pub.tif]

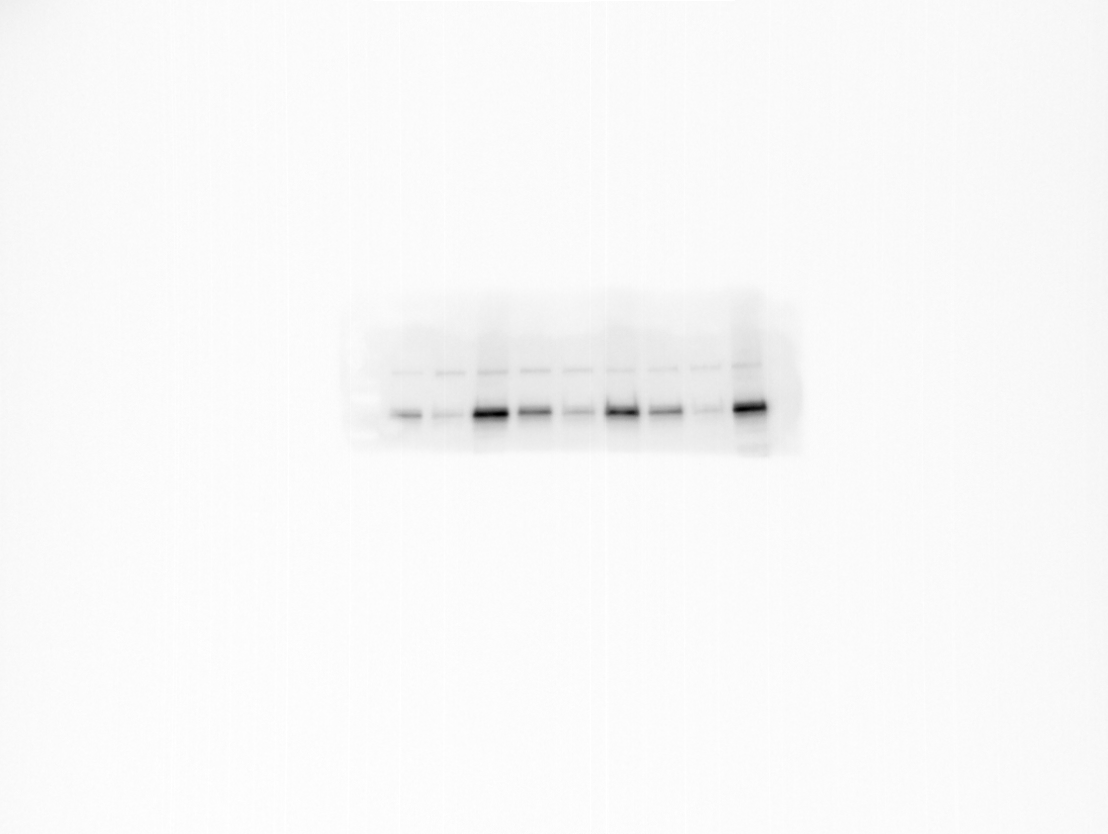

Supplement: Figure 1—figure supplement 1—source data 2. [file elife-100747-fig1-figsupp1-data2.zip › Figure 1 - Figure Supplement 1 - Source Data 2 (original western files)/gls_Proteintech_pico/S1F9-0422-161123.tif]

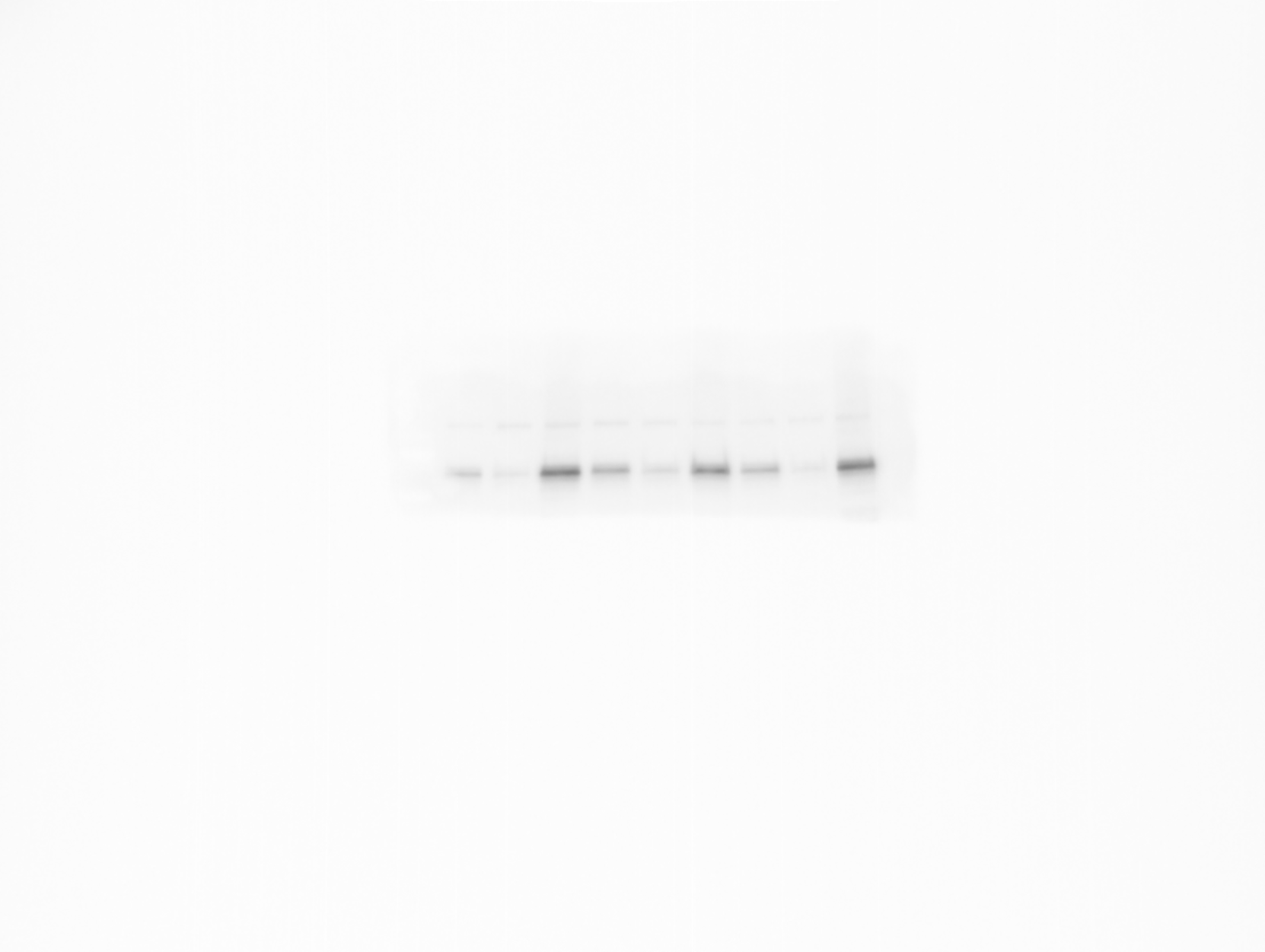

Supplement: Figure 1—figure supplement 1—source data 2. [file elife-100747-fig1-figsupp1-data2.zip › Figure 1 - Figure Supplement 1 - Source Data 2 (original western files)/gls_Proteintech_pico/S1F9-0422-161123_pub.tif]

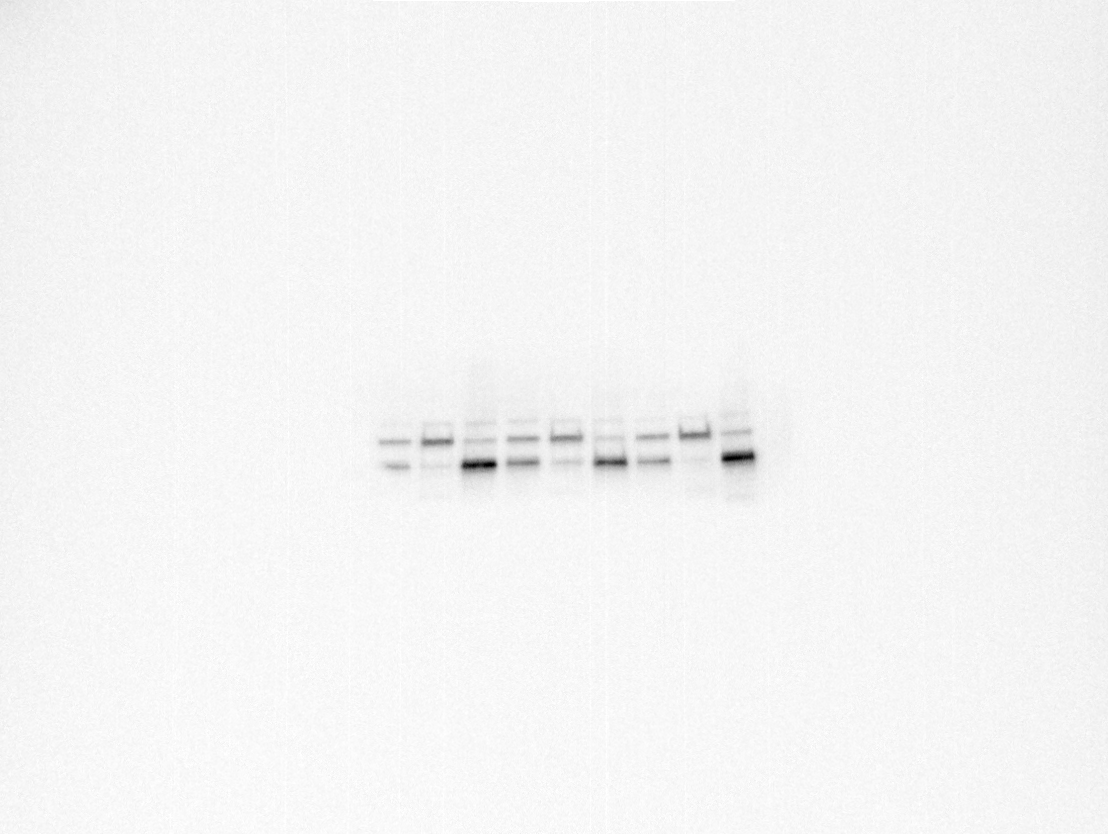

Supplement: Figure 1—figure supplement 1—source data 2. [file elife-100747-fig1-figsupp1-data2.zip › Figure 1 - Figure Supplement 1 - Source Data 2 (original western files)/hsp90_cyto_pico/2022-0425-122601.tif]

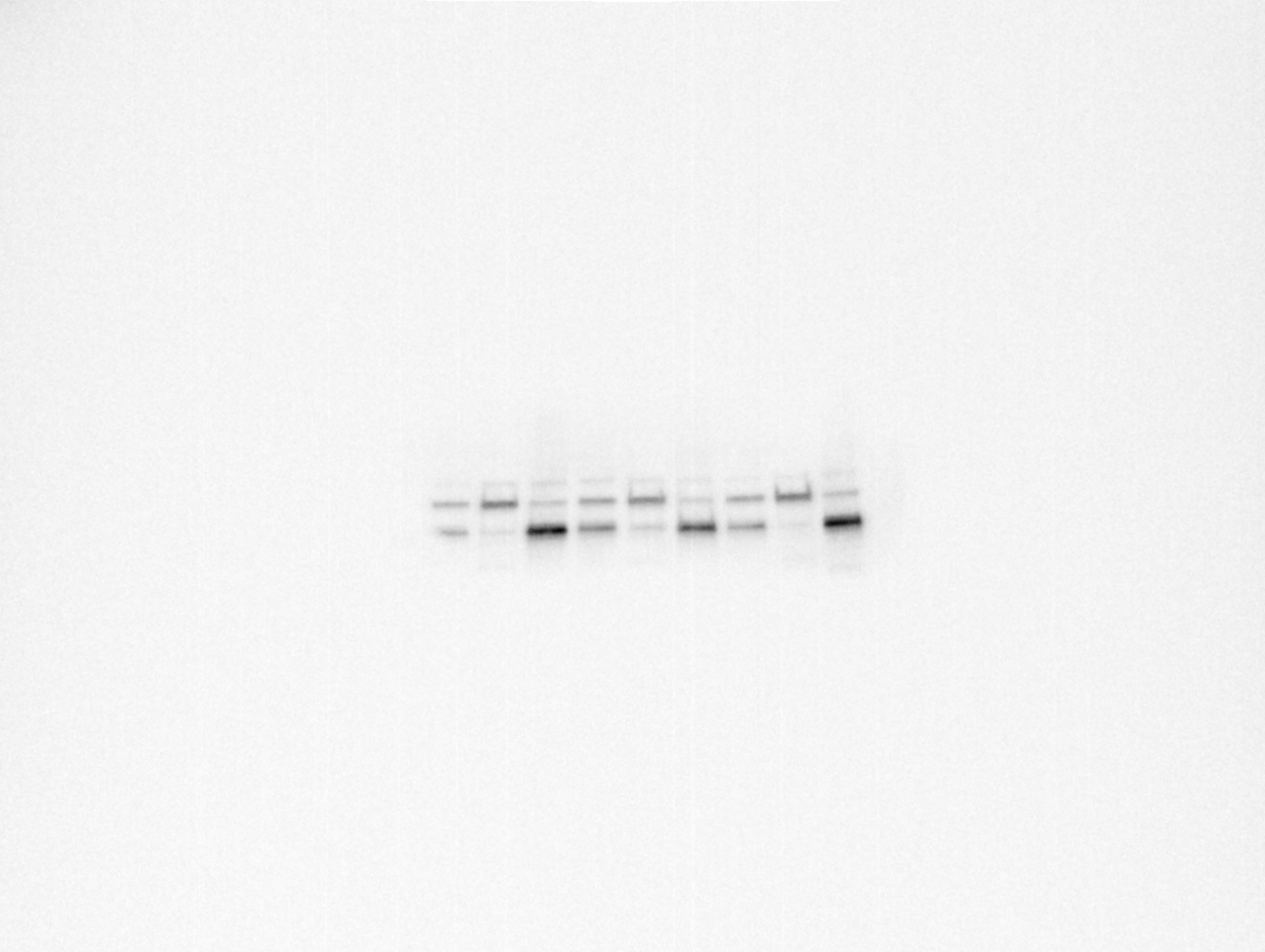

Supplement: Figure 1—figure supplement 1—source data 2. [file elife-100747-fig1-figsupp1-data2.zip › Figure 1 - Figure Supplement 1 - Source Data 2 (original western files)/hsp90_cyto_pico/2022-0425-122601_pub.tif]

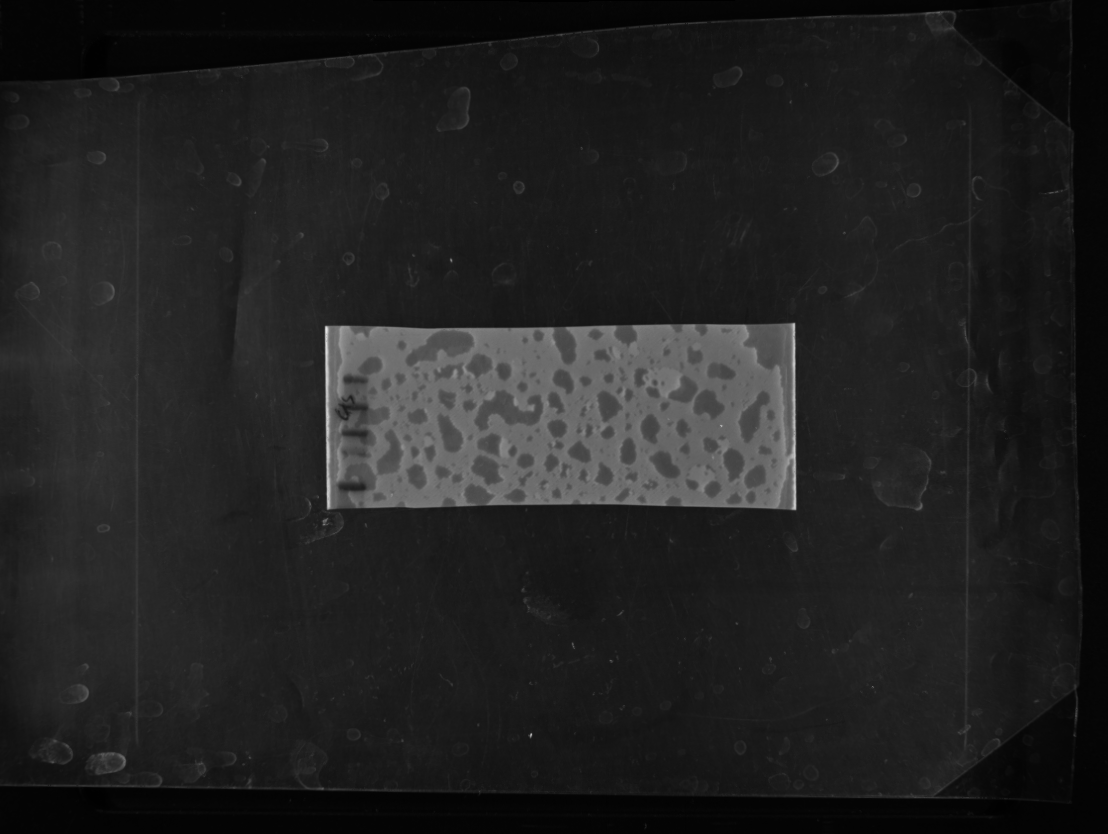

Supplement: Figure 1—figure supplement 1—source data 2. [file elife-100747-fig1-figsupp1-data2.zip › Figure 1 - Figure Supplement 1 - Source Data 2 (original western files)/hsp90_cyto_pico/2022-0425-122603.tif]

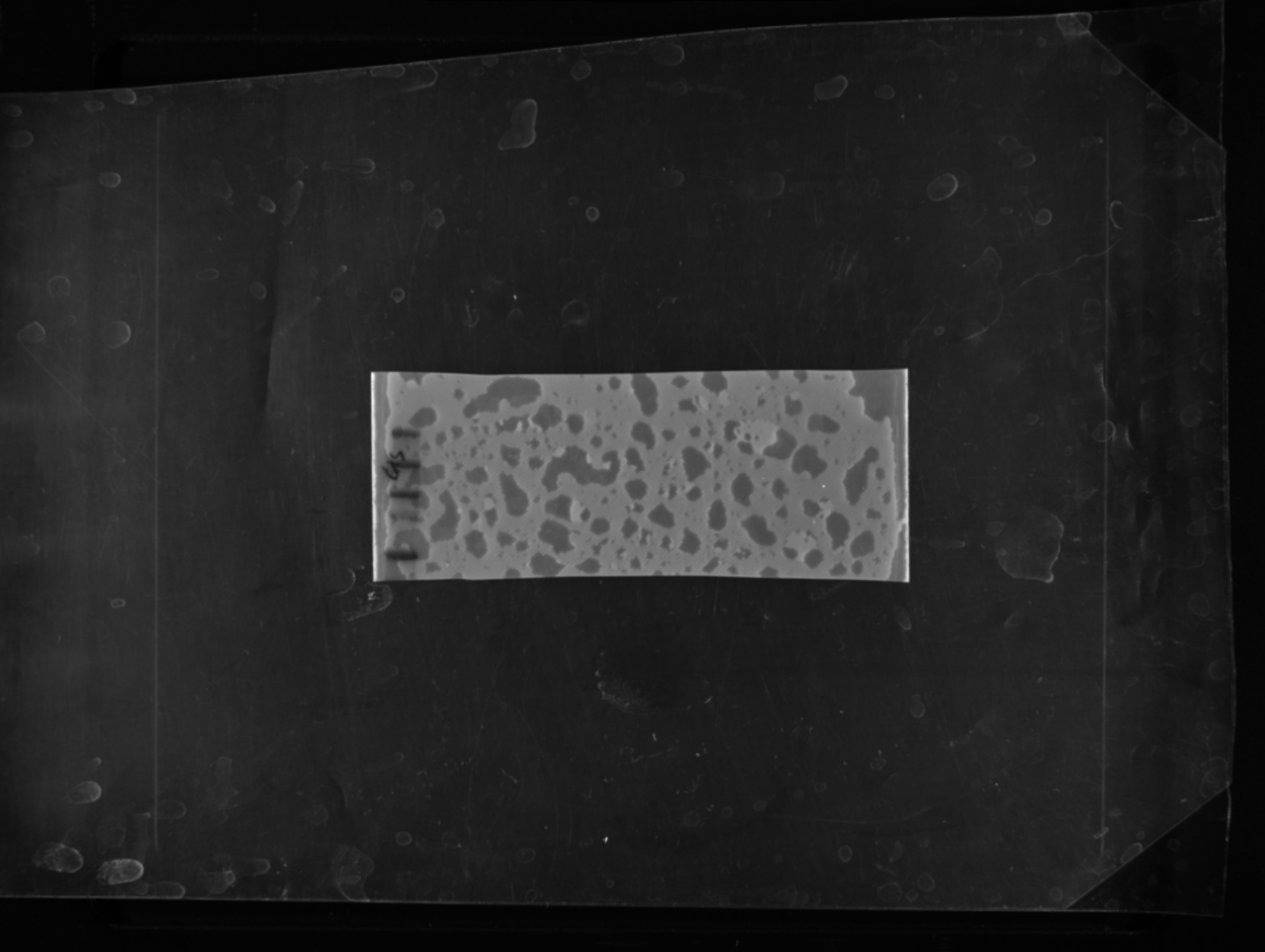

Supplement: Figure 1—figure supplement 1—source data 2. [file elife-100747-fig1-figsupp1-data2.zip › Figure 1 - Figure Supplement 1 - Source Data 2 (original western files)/hsp90_cyto_pico/2022-0425-122603_pub.tif]

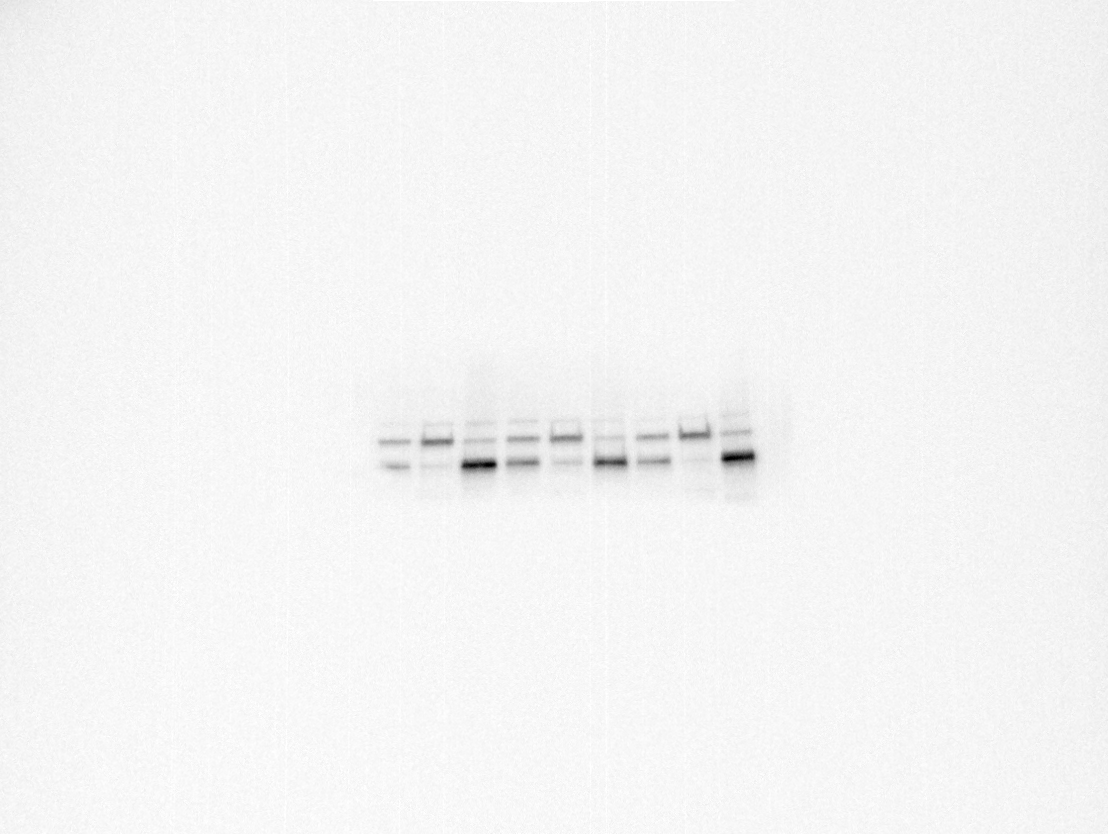

Supplement: Figure 1—figure supplement 1—source data 2. [file elife-100747-fig1-figsupp1-data2.zip › Figure 1 - Figure Supplement 1 - Source Data 2 (original western files)/hsp90_cyto_pico/2022-0425-122605.tif]

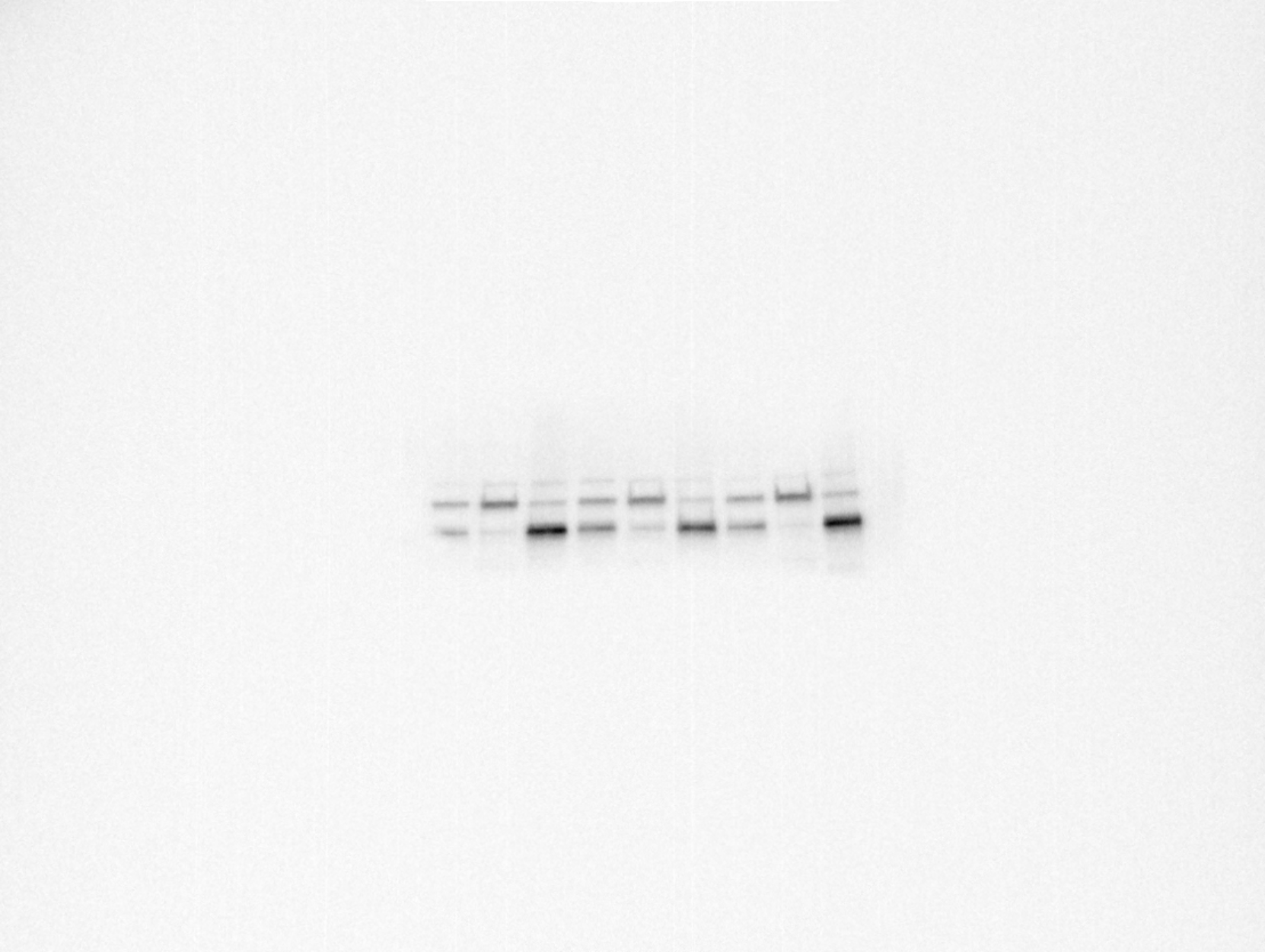

Supplement: Figure 1—figure supplement 1—source data 2. [file elife-100747-fig1-figsupp1-data2.zip › Figure 1 - Figure Supplement 1 - Source Data 2 (original western files)/hsp90_cyto_pico/2022-0425-122605_pub.tif]

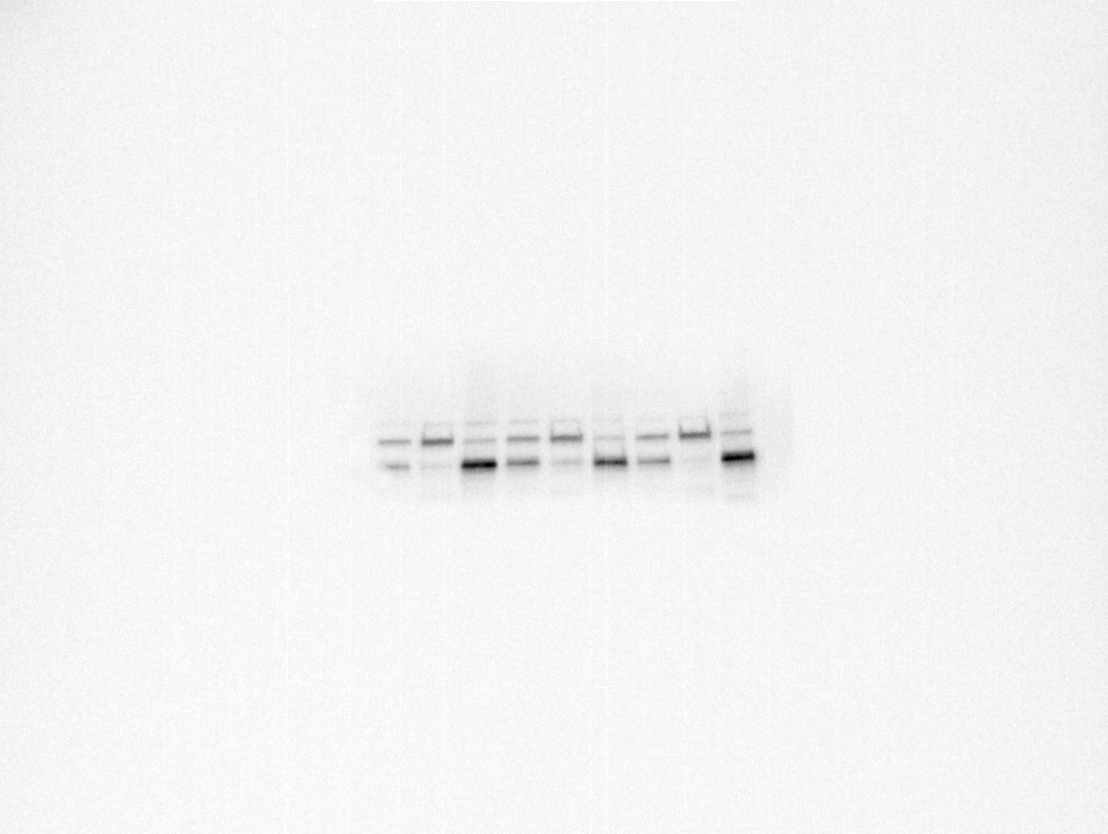

Supplement: Figure 1—figure supplement 1—source data 2. [file elife-100747-fig1-figsupp1-data2.zip › Figure 1 - Figure Supplement 1 - Source Data 2 (original western files)/hsp90_cyto_pico/S1F1-0425-122607.tif]

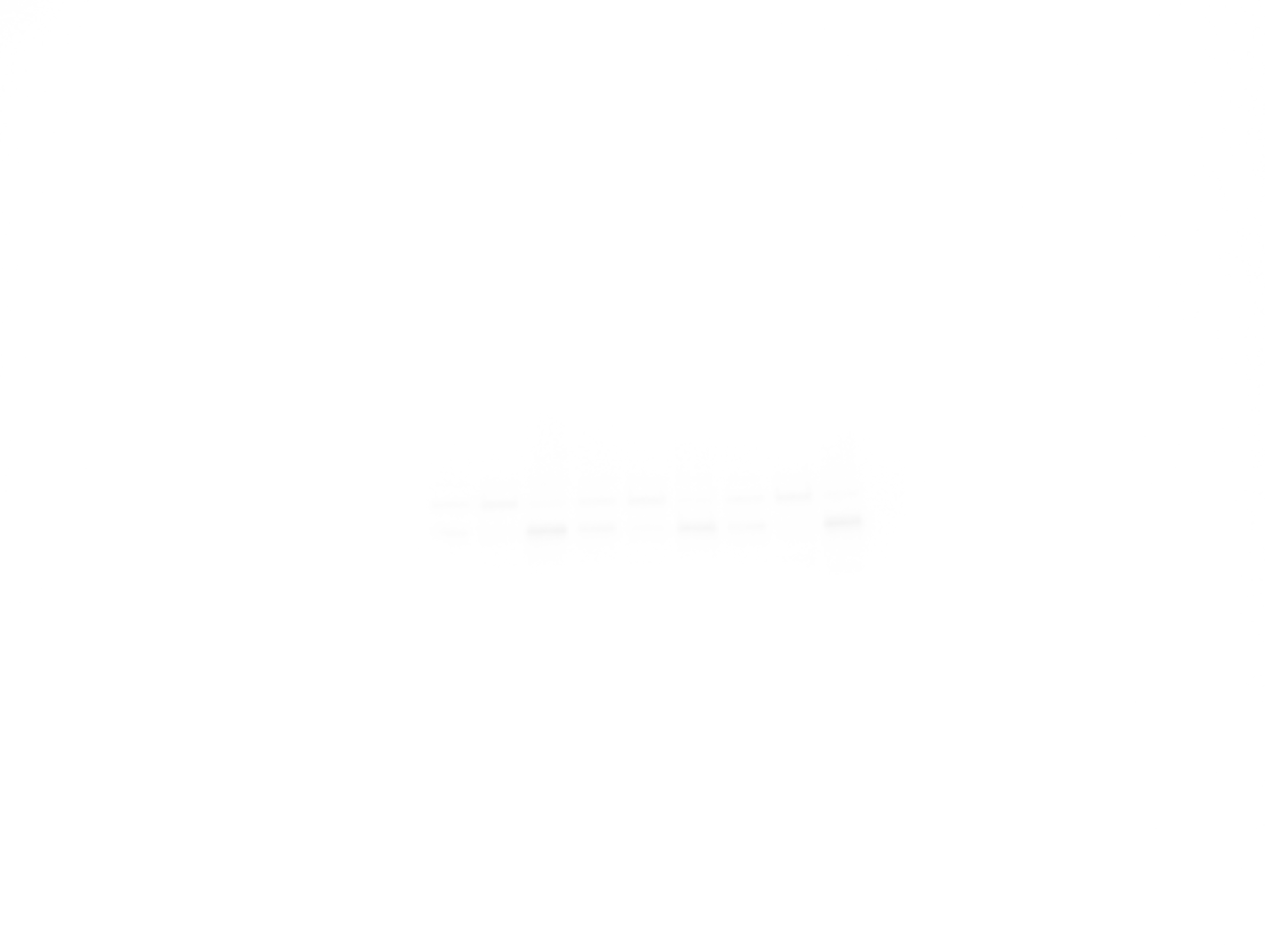

Supplement: Figure 1—figure supplement 1—source data 2. [file elife-100747-fig1-figsupp1-data2.zip › Figure 1 - Figure Supplement 1 - Source Data 2 (original western files)/hsp90_cyto_pico/S1F1-0425-122607_pub.tif]

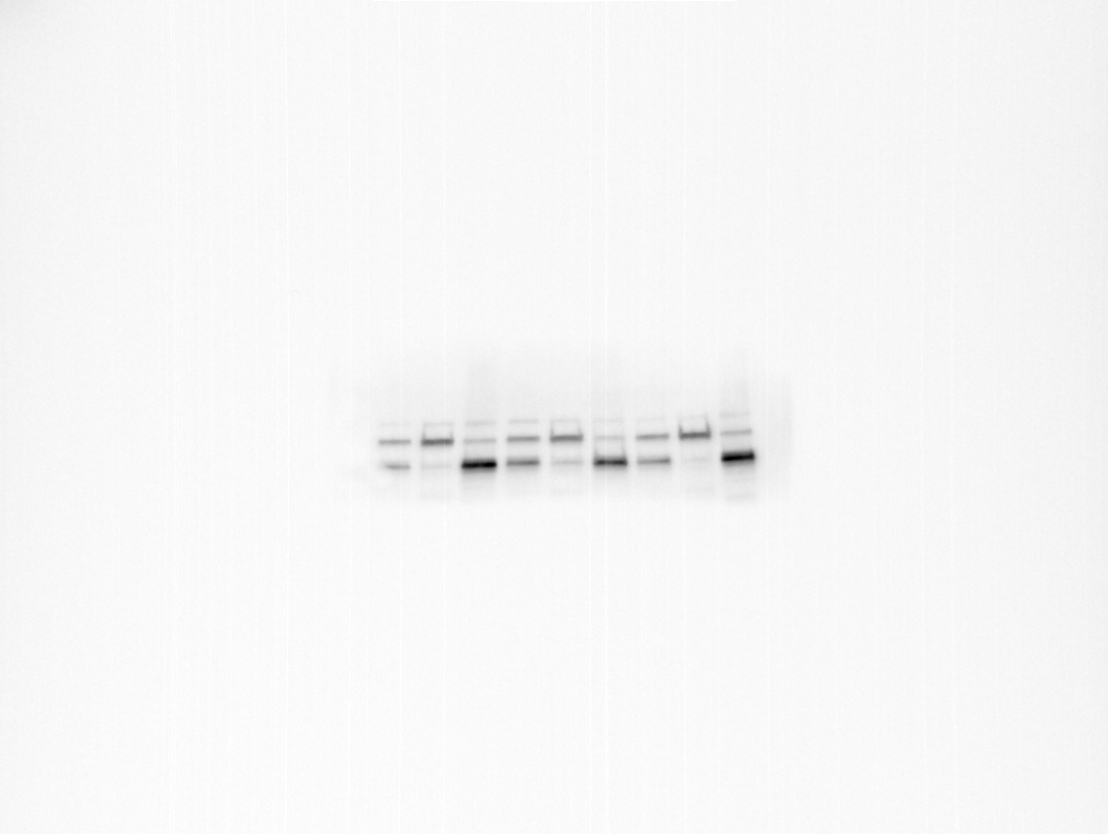

Supplement: Figure 1—figure supplement 1—source data 2. [file elife-100747-fig1-figsupp1-data2.zip › Figure 1 - Figure Supplement 1 - Source Data 2 (original western files)/hsp90_cyto_pico/S1F10-0425-122621.tif]

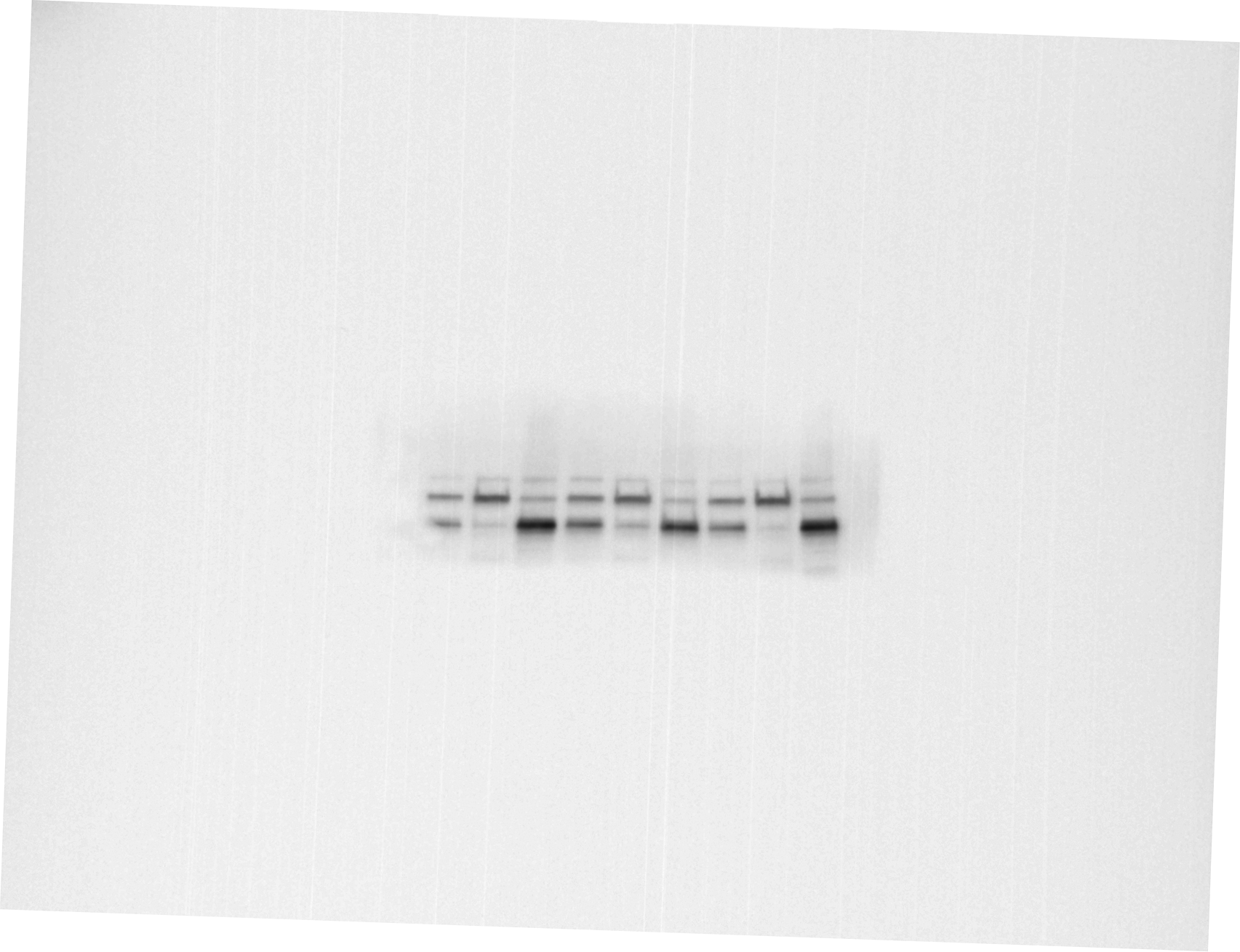

Supplement: Figure 1—figure supplement 1—source data 2. [file elife-100747-fig1-figsupp1-data2.zip › Figure 1 - Figure Supplement 1 - Source Data 2 (original western files)/hsp90_cyto_pico/S1F10-0425-122621_pub.tif]

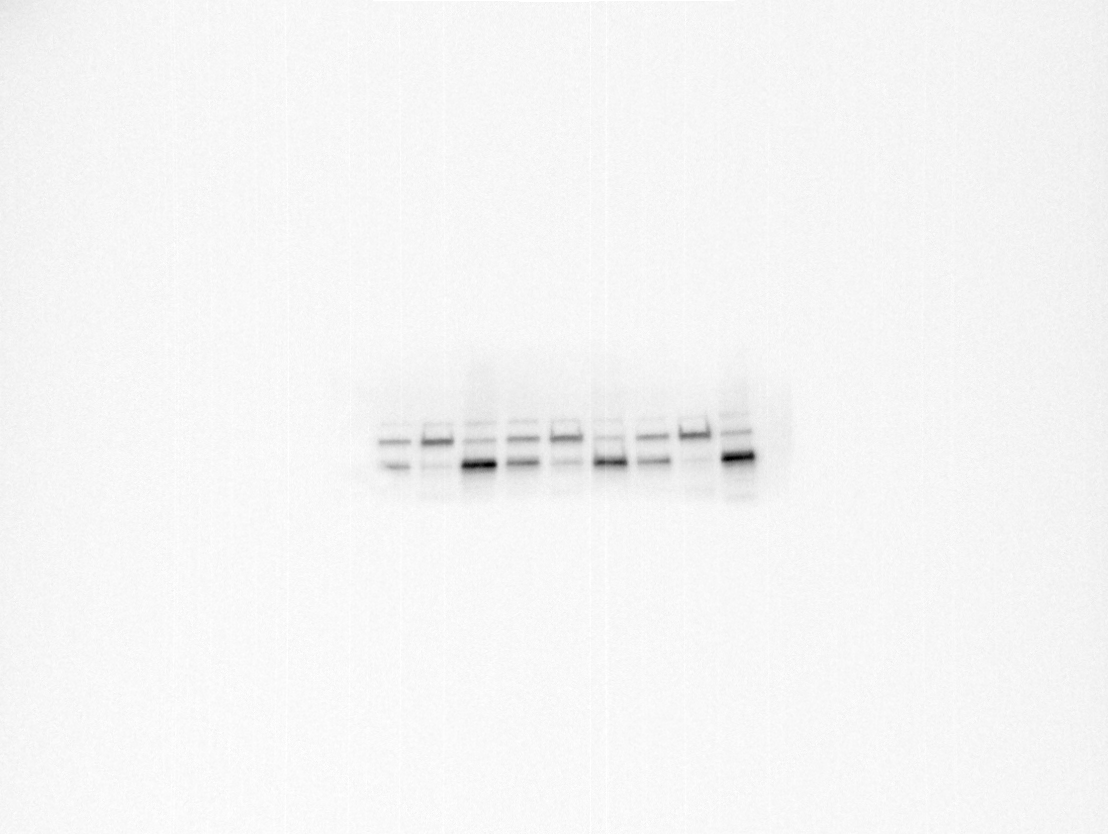

Supplement: Figure 1—figure supplement 1—source data 2. [file elife-100747-fig1-figsupp1-data2.zip › Figure 1 - Figure Supplement 1 - Source Data 2 (original western files)/hsp90_cyto_pico/S1F2-0425-122608.tif]

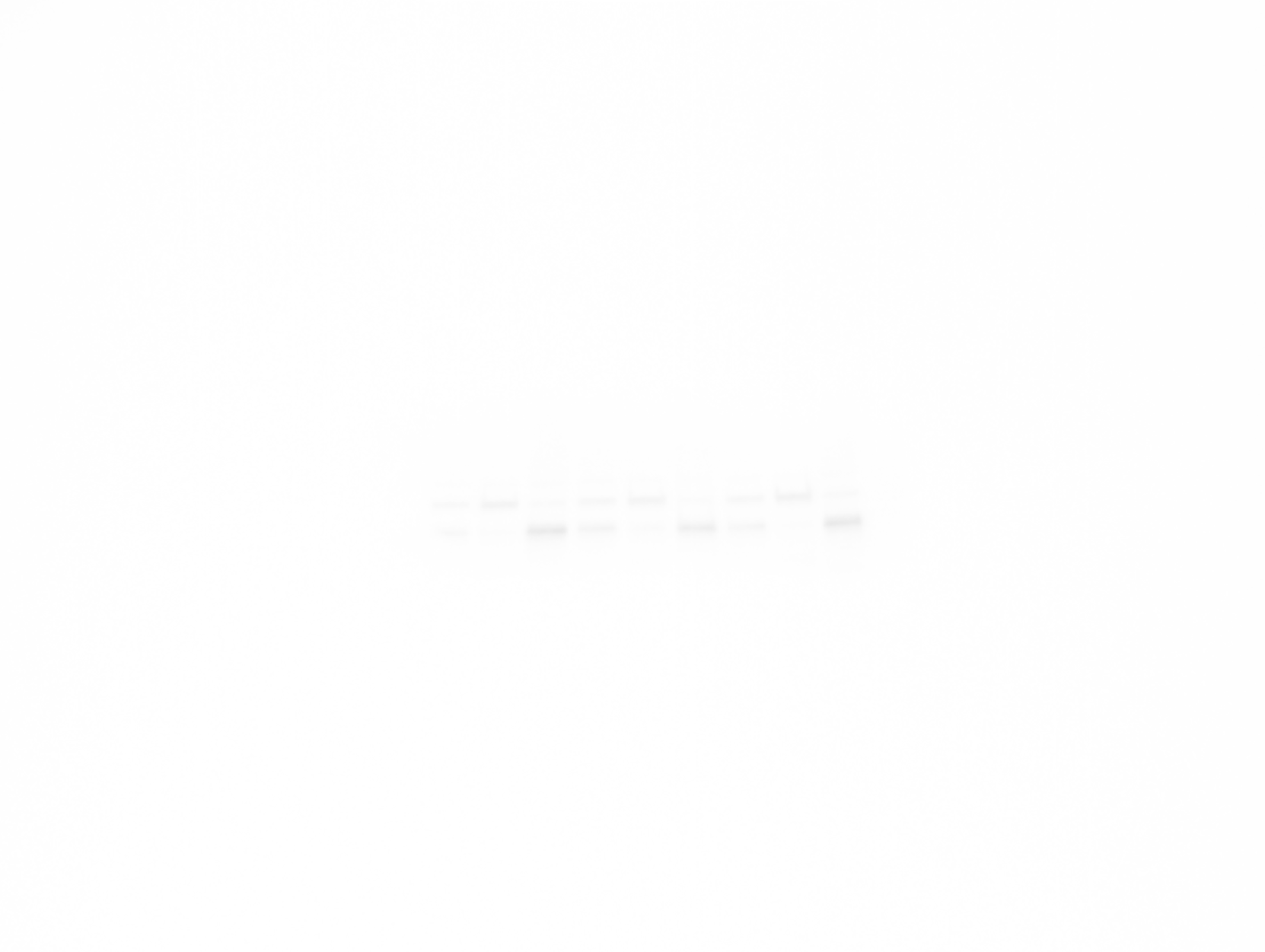

Supplement: Figure 1—figure supplement 1—source data 2. [file elife-100747-fig1-figsupp1-data2.zip › Figure 1 - Figure Supplement 1 - Source Data 2 (original western files)/hsp90_cyto_pico/S1F2-0425-122608_pub.tif]

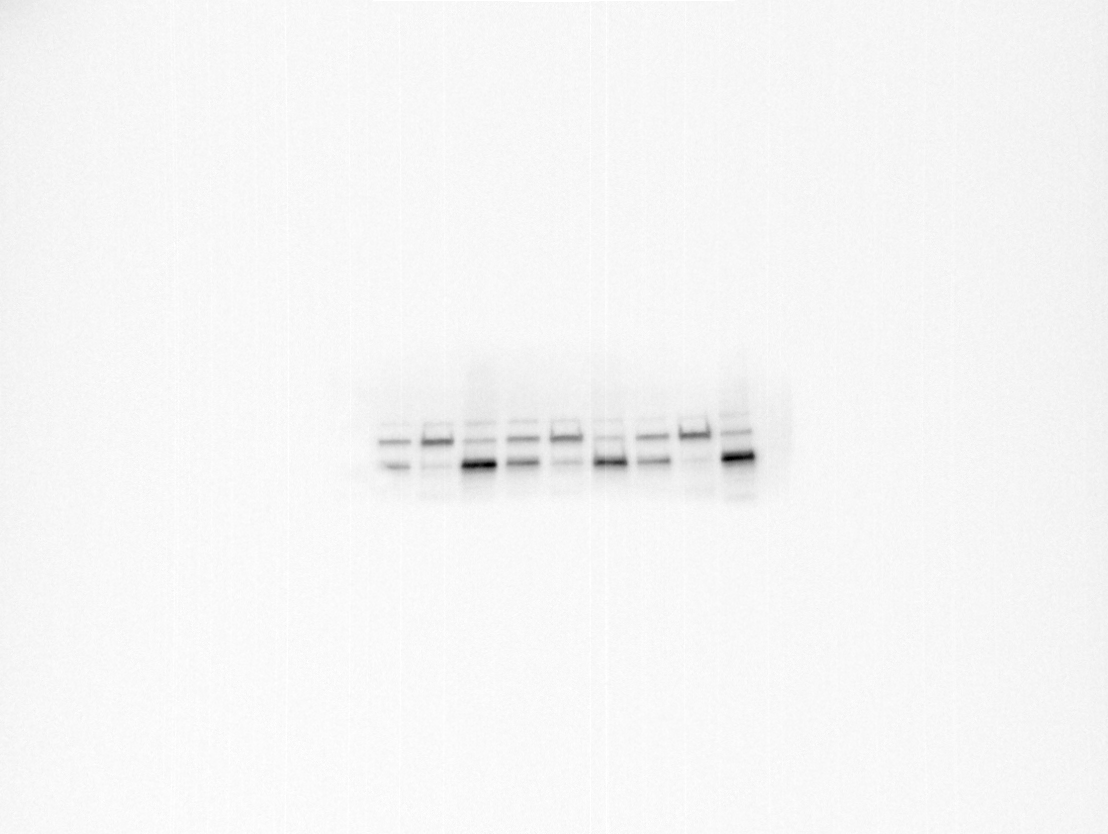

Supplement: Figure 1—figure supplement 1—source data 2. [file elife-100747-fig1-figsupp1-data2.zip › Figure 1 - Figure Supplement 1 - Source Data 2 (original western files)/hsp90_cyto_pico/S1F3-0425-122610.tif]

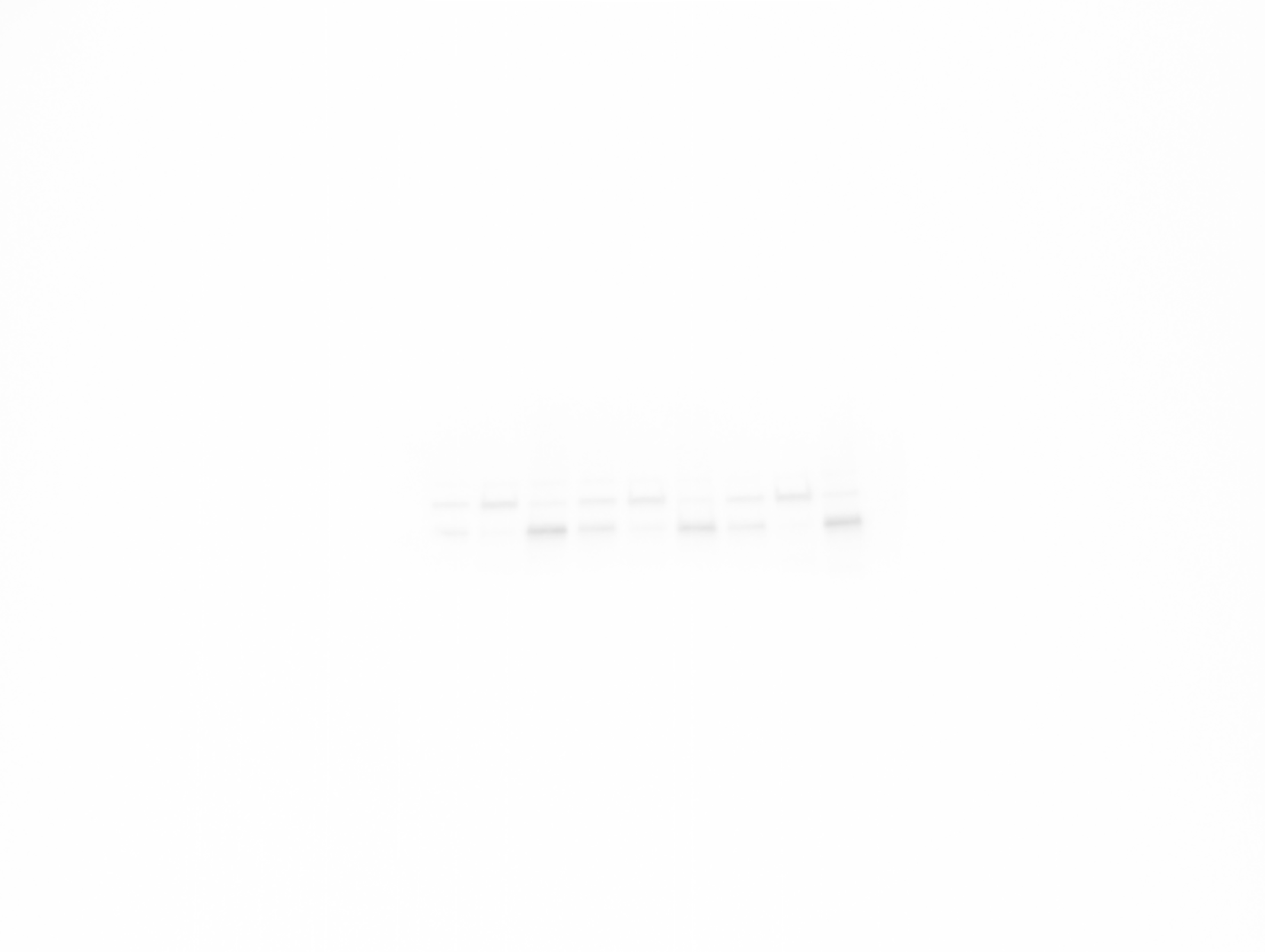

Supplement: Figure 1—figure supplement 1—source data 2. [file elife-100747-fig1-figsupp1-data2.zip › Figure 1 - Figure Supplement 1 - Source Data 2 (original western files)/hsp90_cyto_pico/S1F3-0425-122610_pub.tif]

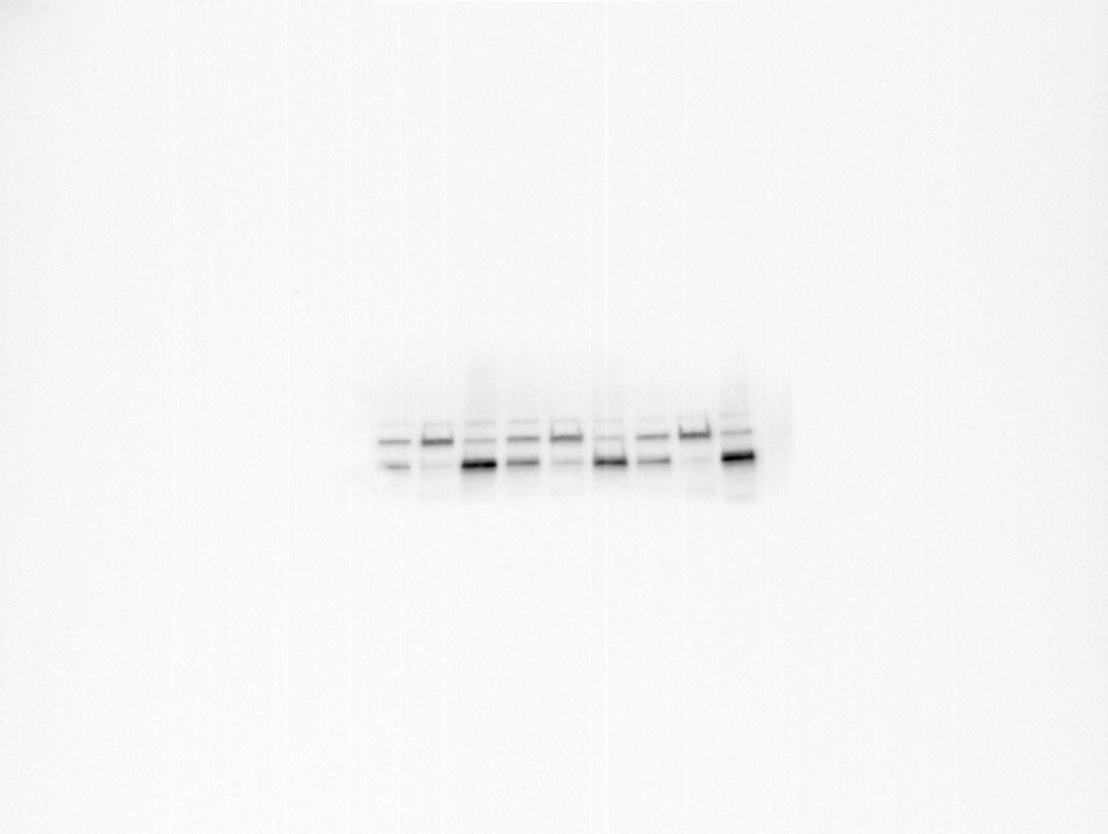

Supplement: Figure 1—figure supplement 1—source data 2. [file elife-100747-fig1-figsupp1-data2.zip › Figure 1 - Figure Supplement 1 - Source Data 2 (original western files)/hsp90_cyto_pico/S1F4-0425-122611.tif]

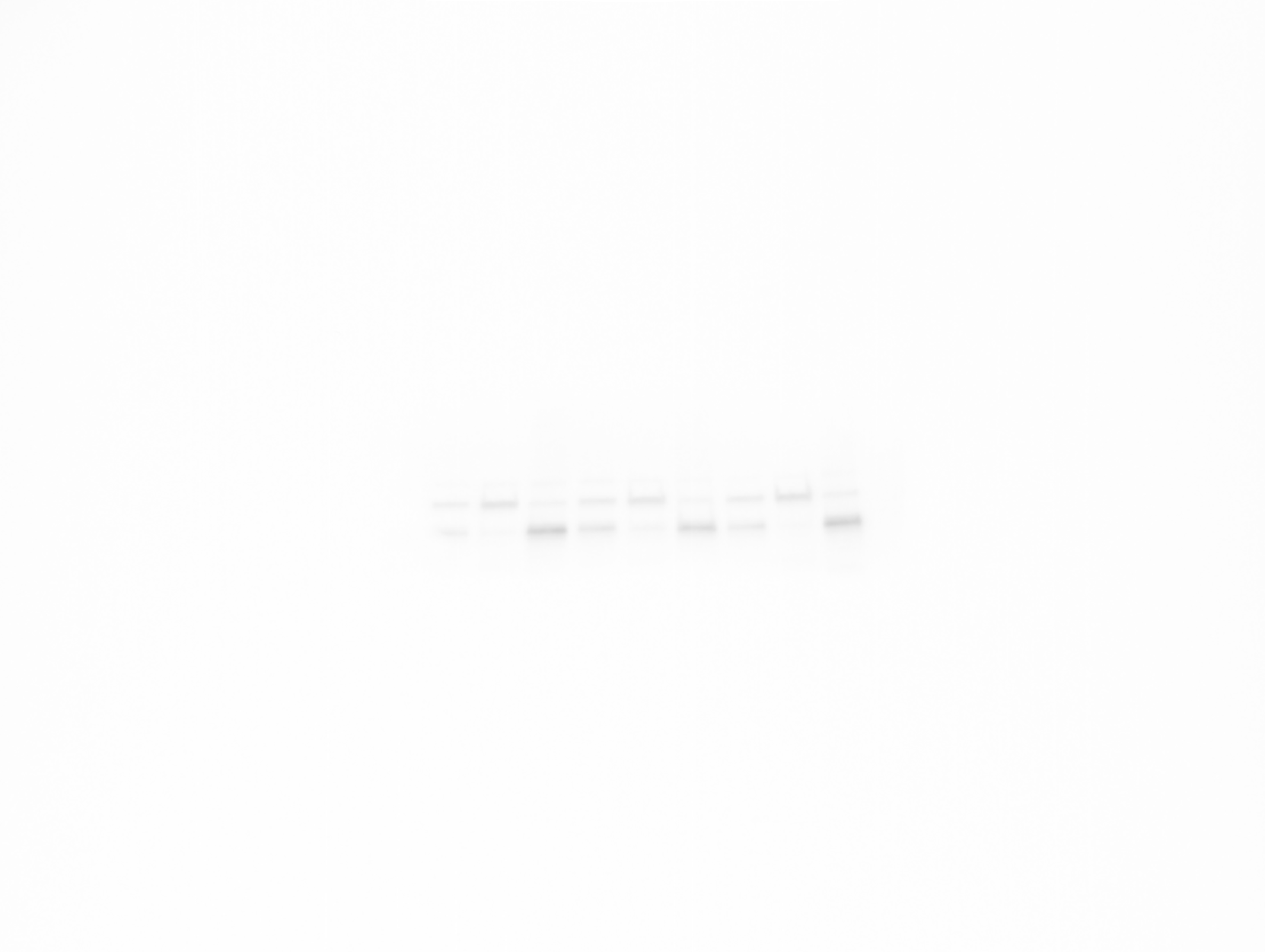

Supplement: Figure 1—figure supplement 1—source data 2. [file elife-100747-fig1-figsupp1-data2.zip › Figure 1 - Figure Supplement 1 - Source Data 2 (original western files)/hsp90_cyto_pico/S1F4-0425-122611_pub.tif]
