# Supplementary figures and images for: Glutamine catabolism supports amino acid biosynthesis and suppresses the integrated stress response to promote photoreceptor survival (part 2 of 4)
Source: eLife. 2025 May 21;13:RP100747. doi: 10.7554/eLife.100747 (PMC12094702; doi:10.7554/eLife.100747)

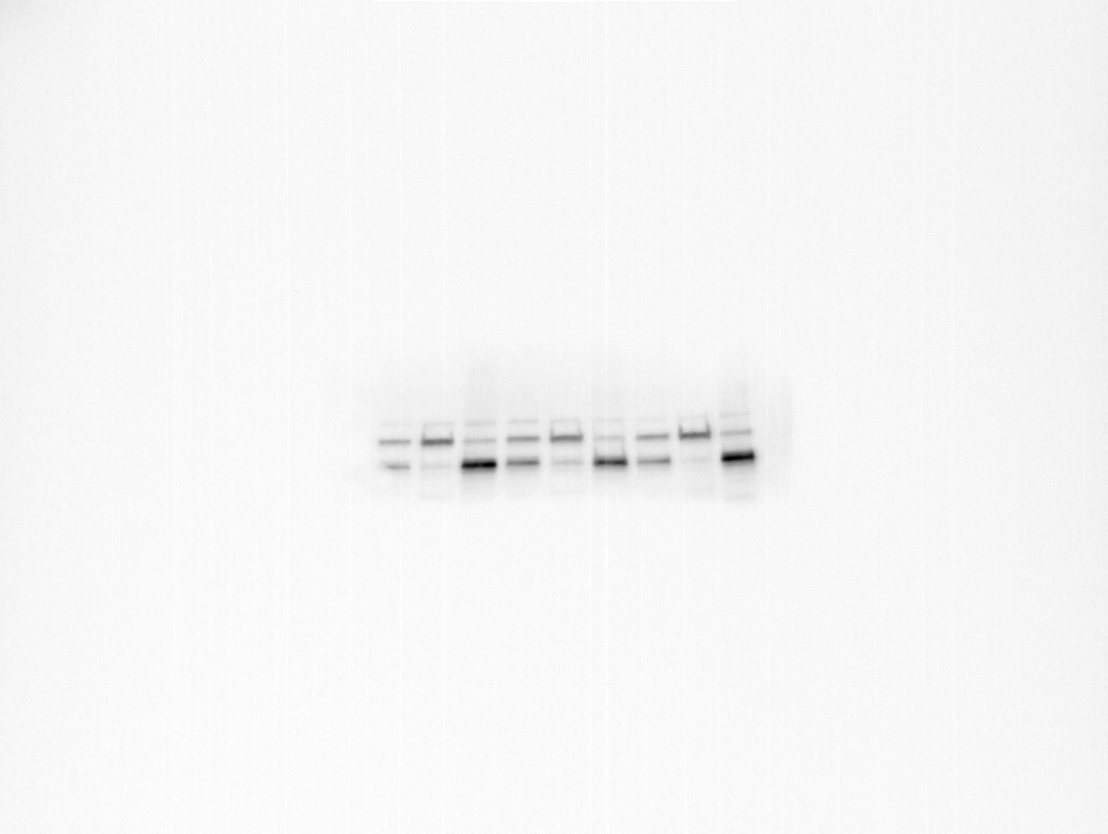

Supplement: Figure 1—figure supplement 1—source data 2. [file elife-100747-fig1-figsupp1-data2.zip › Figure 1 - Figure Supplement 1 - Source Data 2 (original western files)/hsp90_cyto_pico/S1F5-0425-122613.tif]

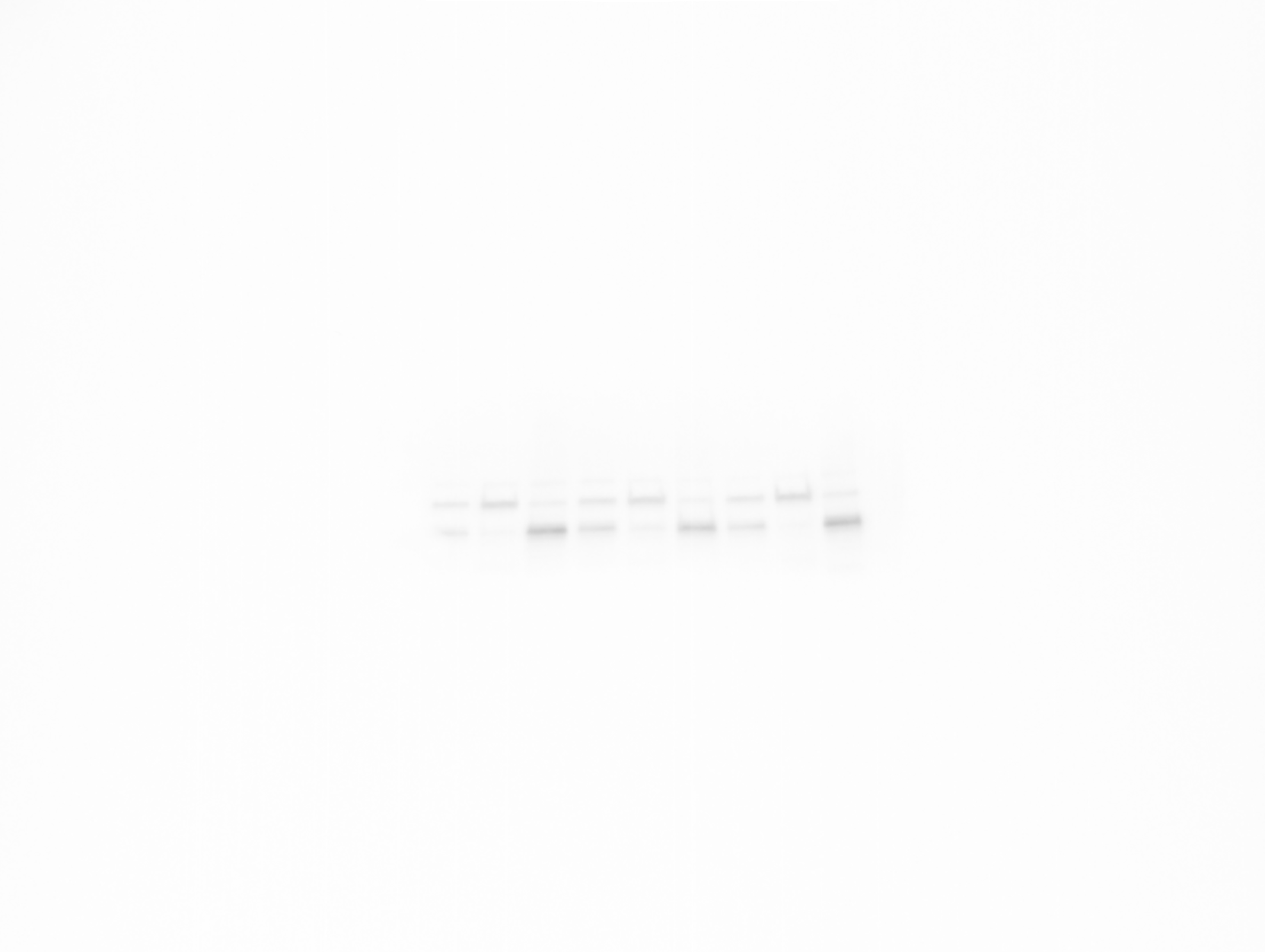

Supplement: Figure 1—figure supplement 1—source data 2. [file elife-100747-fig1-figsupp1-data2.zip › Figure 1 - Figure Supplement 1 - Source Data 2 (original western files)/hsp90_cyto_pico/S1F5-0425-122613_pub.tif]

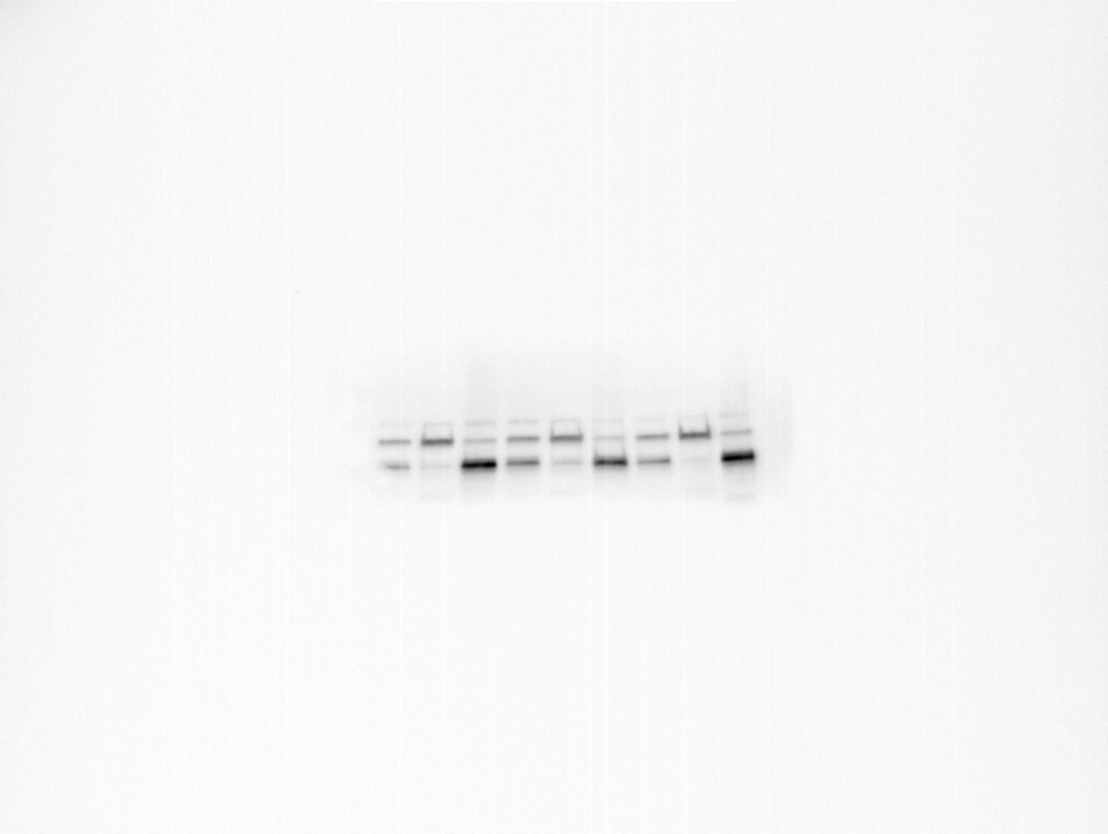

Supplement: Figure 1—figure supplement 1—source data 2. [file elife-100747-fig1-figsupp1-data2.zip › Figure 1 - Figure Supplement 1 - Source Data 2 (original western files)/hsp90_cyto_pico/S1F6-0425-122615.tif]

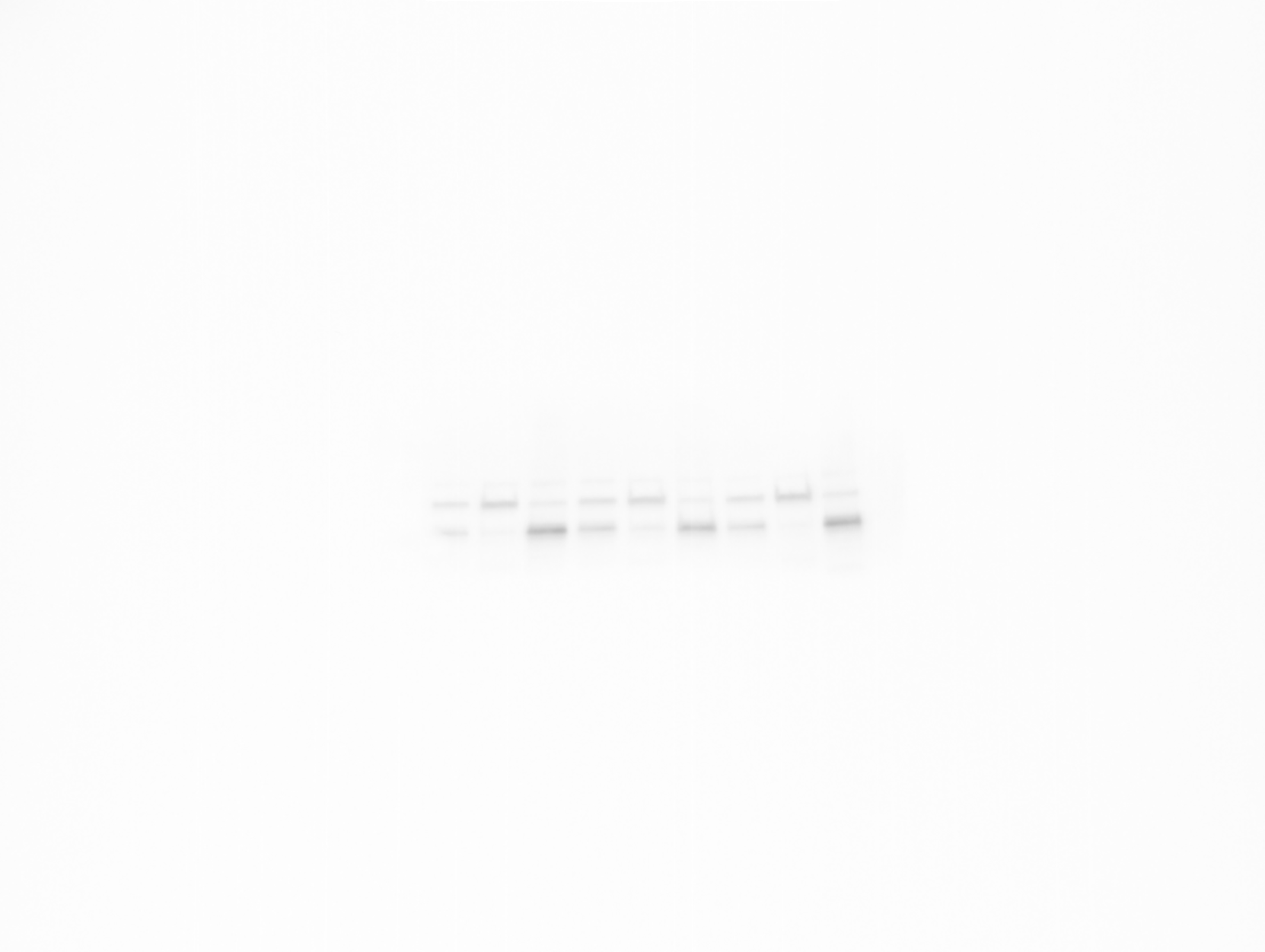

Supplement: Figure 1—figure supplement 1—source data 2. [file elife-100747-fig1-figsupp1-data2.zip › Figure 1 - Figure Supplement 1 - Source Data 2 (original western files)/hsp90_cyto_pico/S1F6-0425-122615_pub.tif]

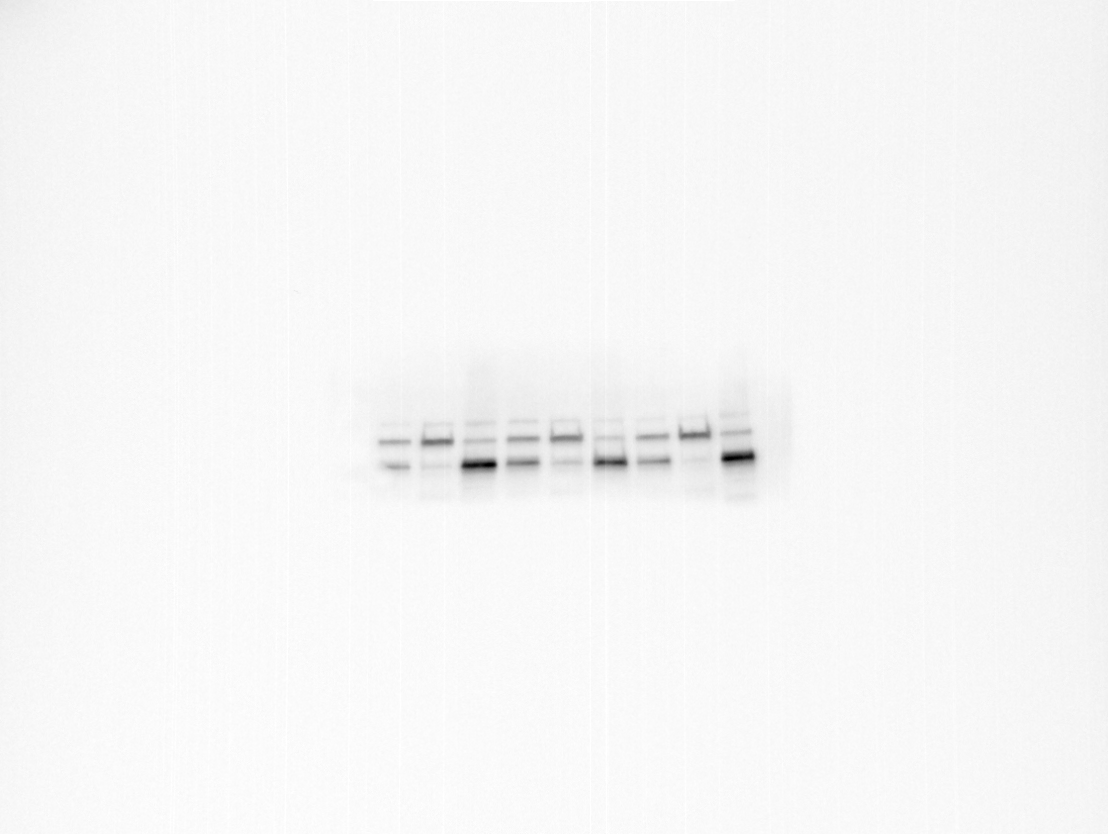

Supplement: Figure 1—figure supplement 1—source data 2. [file elife-100747-fig1-figsupp1-data2.zip › Figure 1 - Figure Supplement 1 - Source Data 2 (original western files)/hsp90_cyto_pico/S1F7-0425-122616.tif]

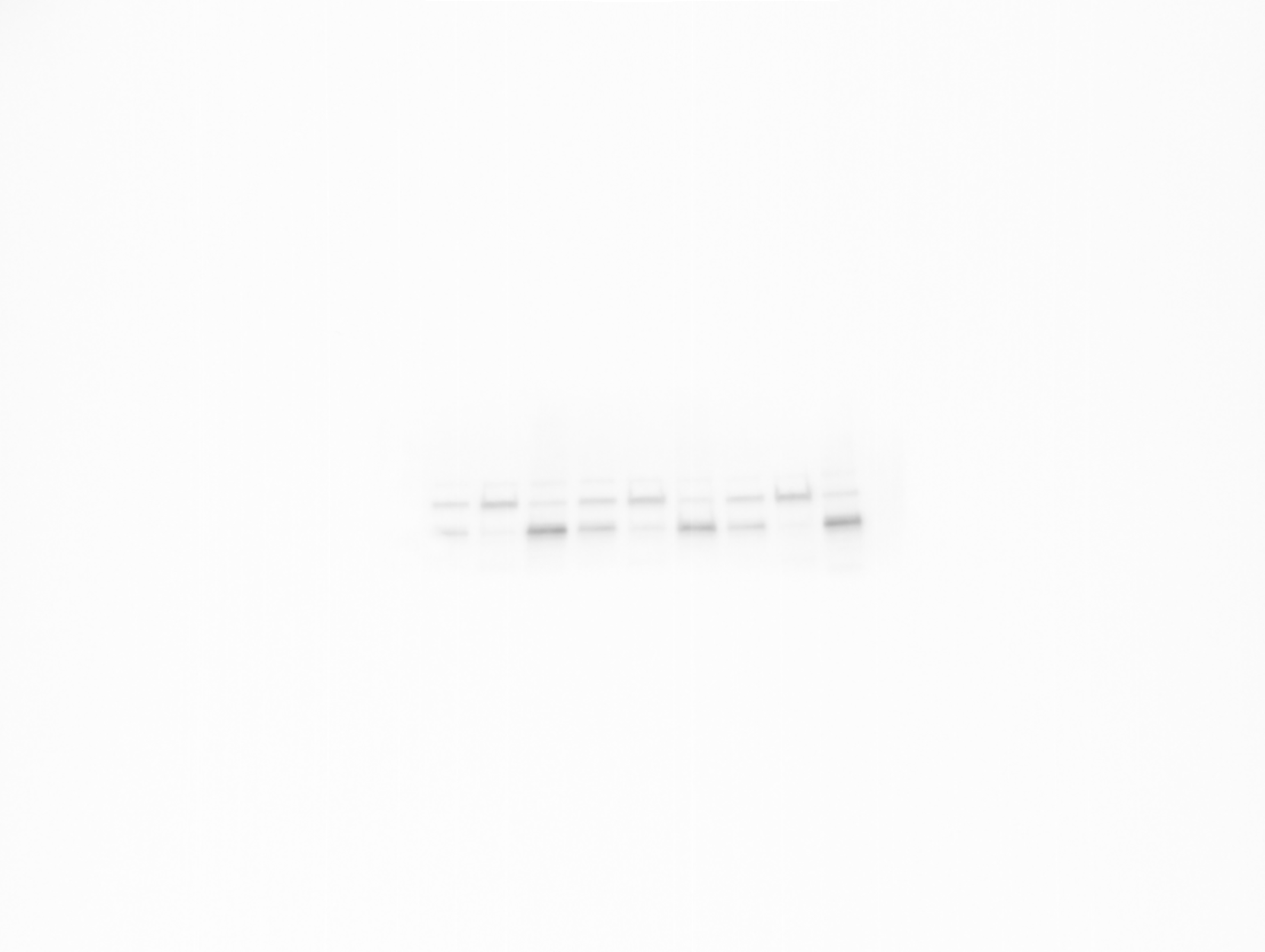

Supplement: Figure 1—figure supplement 1—source data 2. [file elife-100747-fig1-figsupp1-data2.zip › Figure 1 - Figure Supplement 1 - Source Data 2 (original western files)/hsp90_cyto_pico/S1F7-0425-122616_pub.tif]

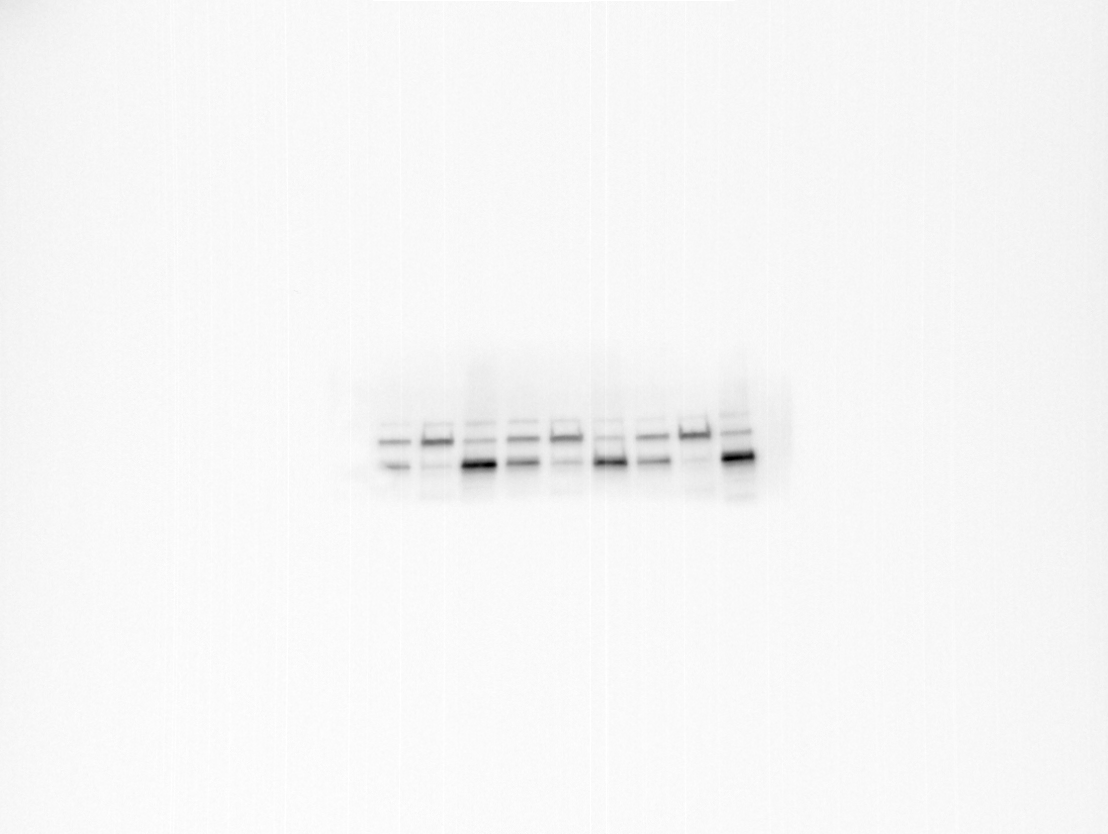

Supplement: Figure 1—figure supplement 1—source data 2. [file elife-100747-fig1-figsupp1-data2.zip › Figure 1 - Figure Supplement 1 - Source Data 2 (original western files)/hsp90_cyto_pico/S1F8-0425-122618.tif]

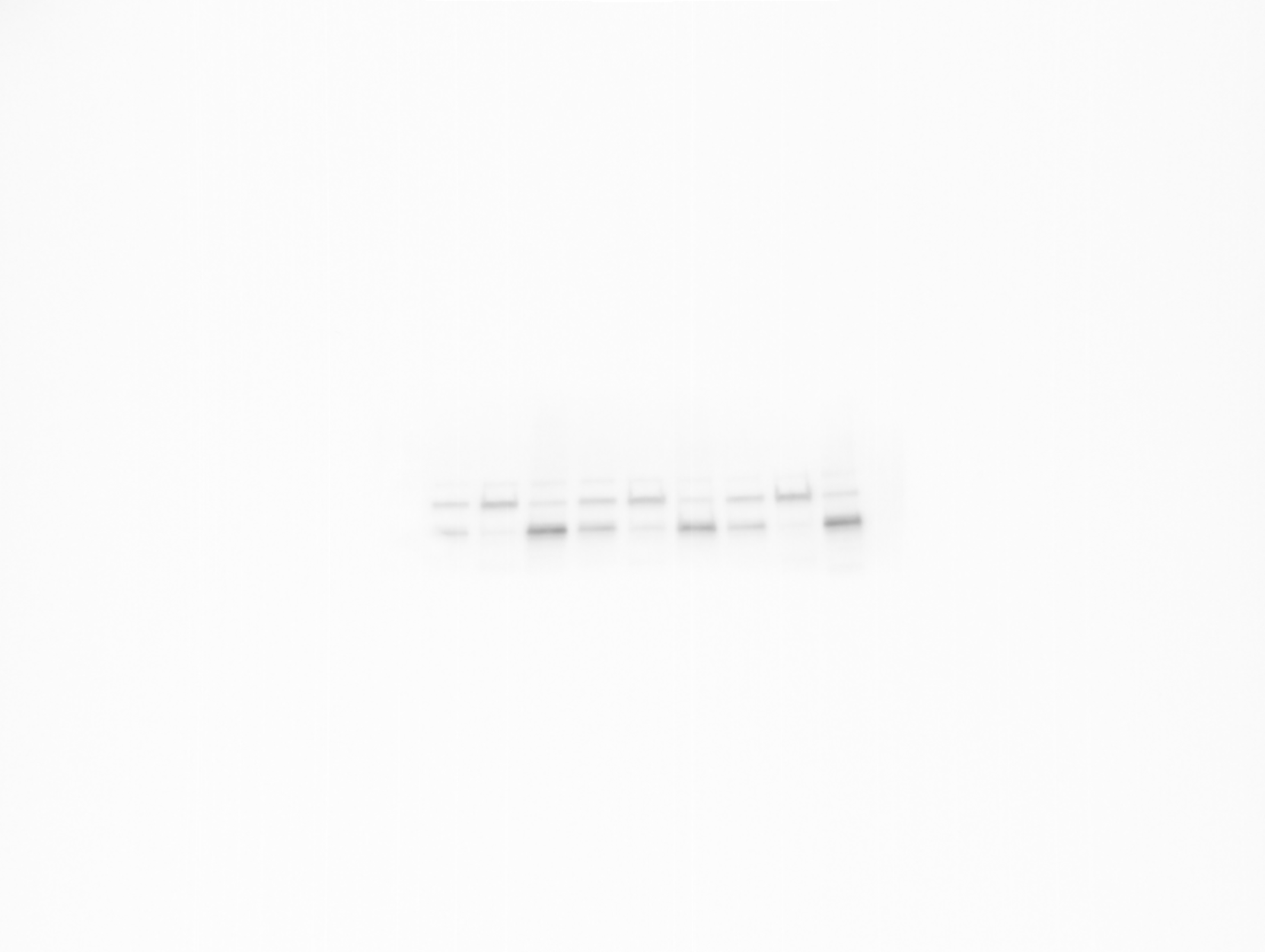

Supplement: Figure 1—figure supplement 1—source data 2. [file elife-100747-fig1-figsupp1-data2.zip › Figure 1 - Figure Supplement 1 - Source Data 2 (original western files)/hsp90_cyto_pico/S1F8-0425-122618_pub.tif]

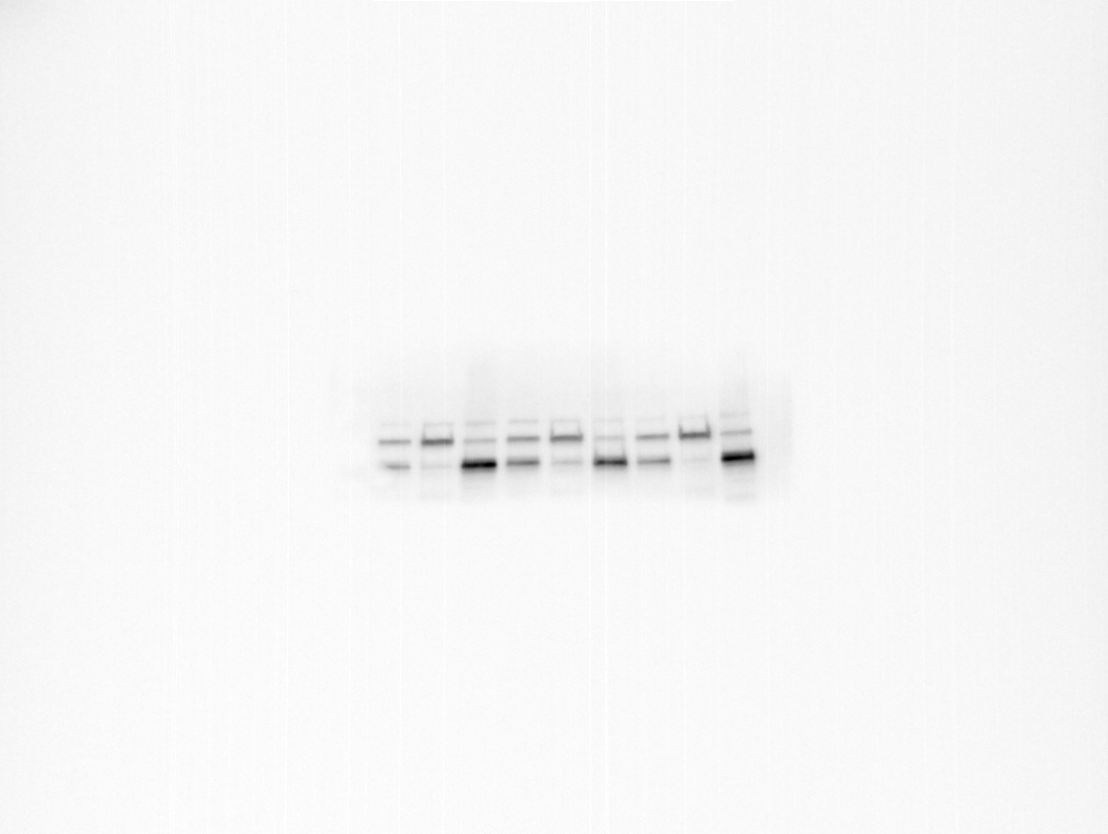

Supplement: Figure 1—figure supplement 1—source data 2. [file elife-100747-fig1-figsupp1-data2.zip › Figure 1 - Figure Supplement 1 - Source Data 2 (original western files)/hsp90_cyto_pico/S1F9-0425-122620.tif]

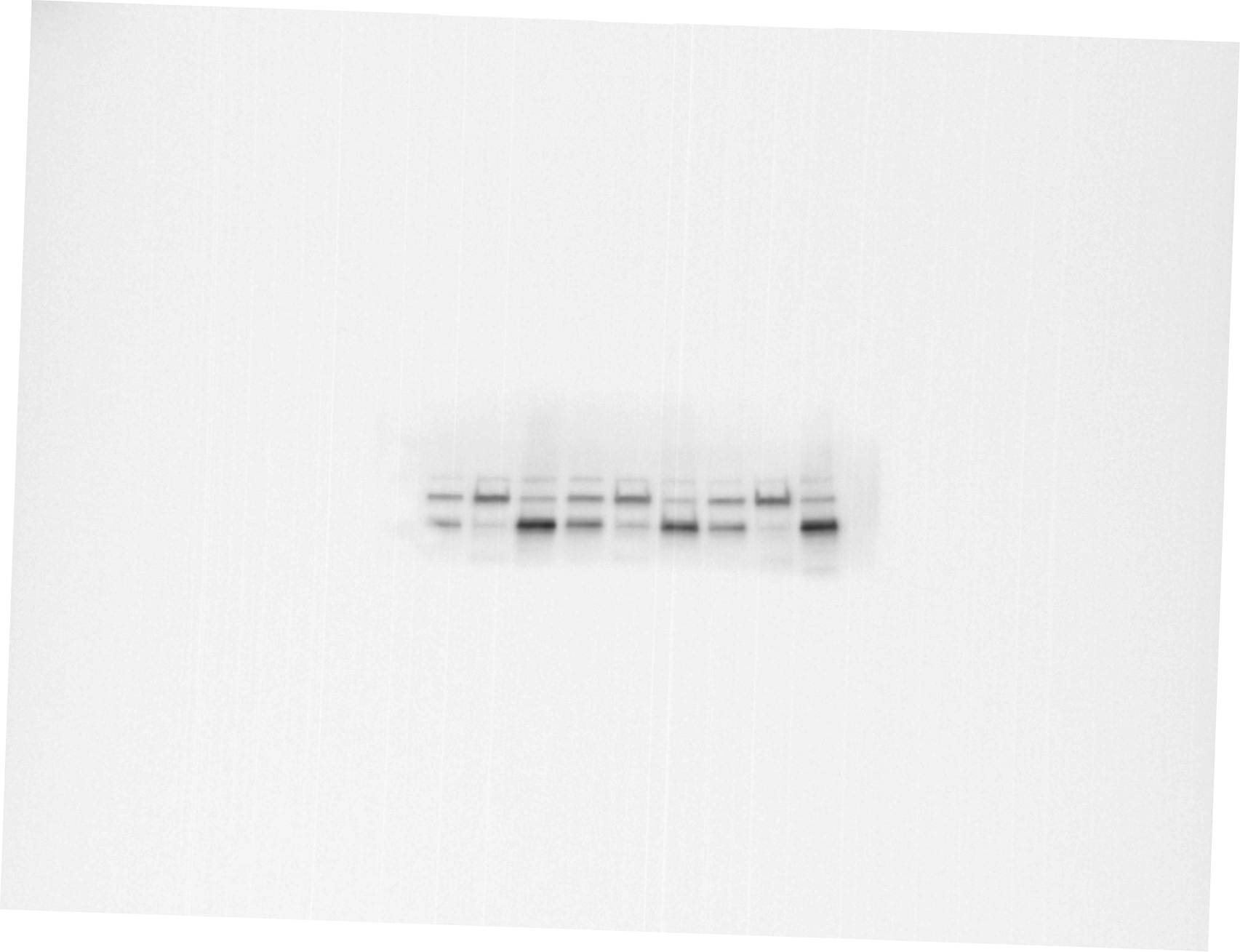

Supplement: Figure 1—figure supplement 1—source data 2. [file elife-100747-fig1-figsupp1-data2.zip › Figure 1 - Figure Supplement 1 - Source Data 2 (original western files)/hsp90_cyto_pico/S1F9-0425-122620_pub.tif]

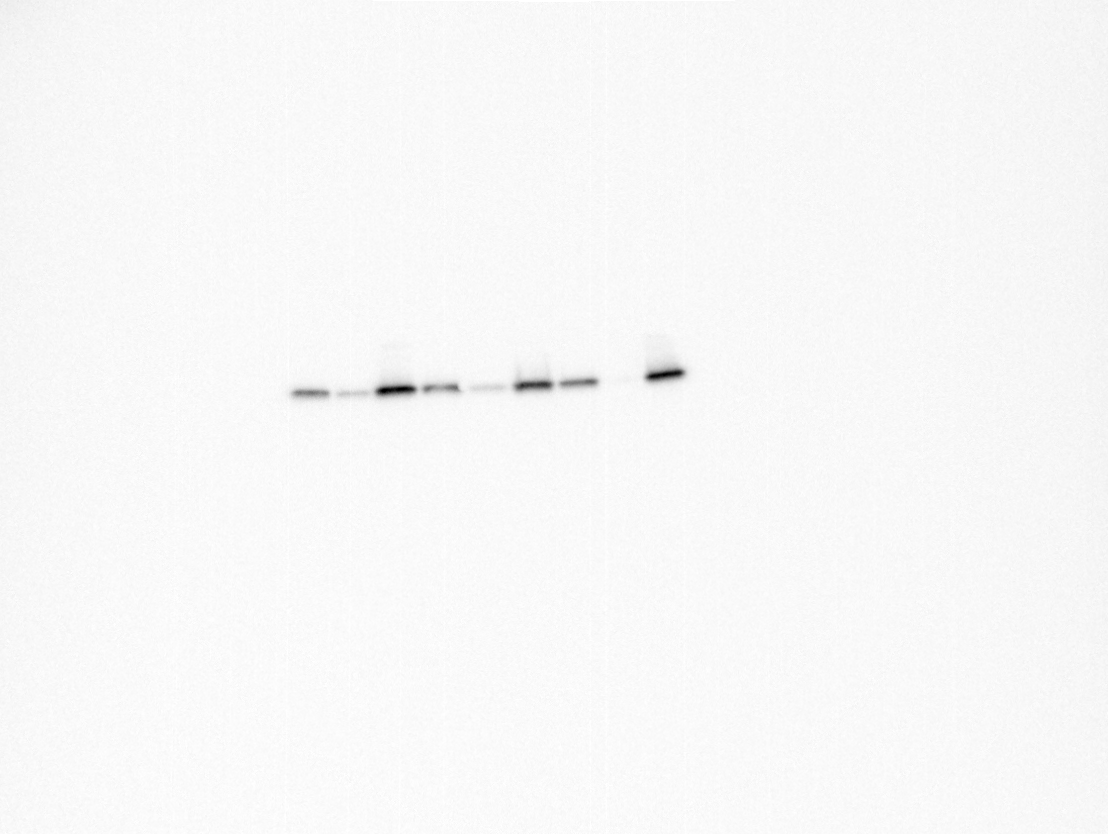

Supplement: Figure 1—figure supplement 1—source data 2. [file elife-100747-fig1-figsupp1-data2.zip › Figure 1 - Figure Supplement 1 - Source Data 2 (original western files)/Tim23_mito_pico/2022-0422-161438.tif]

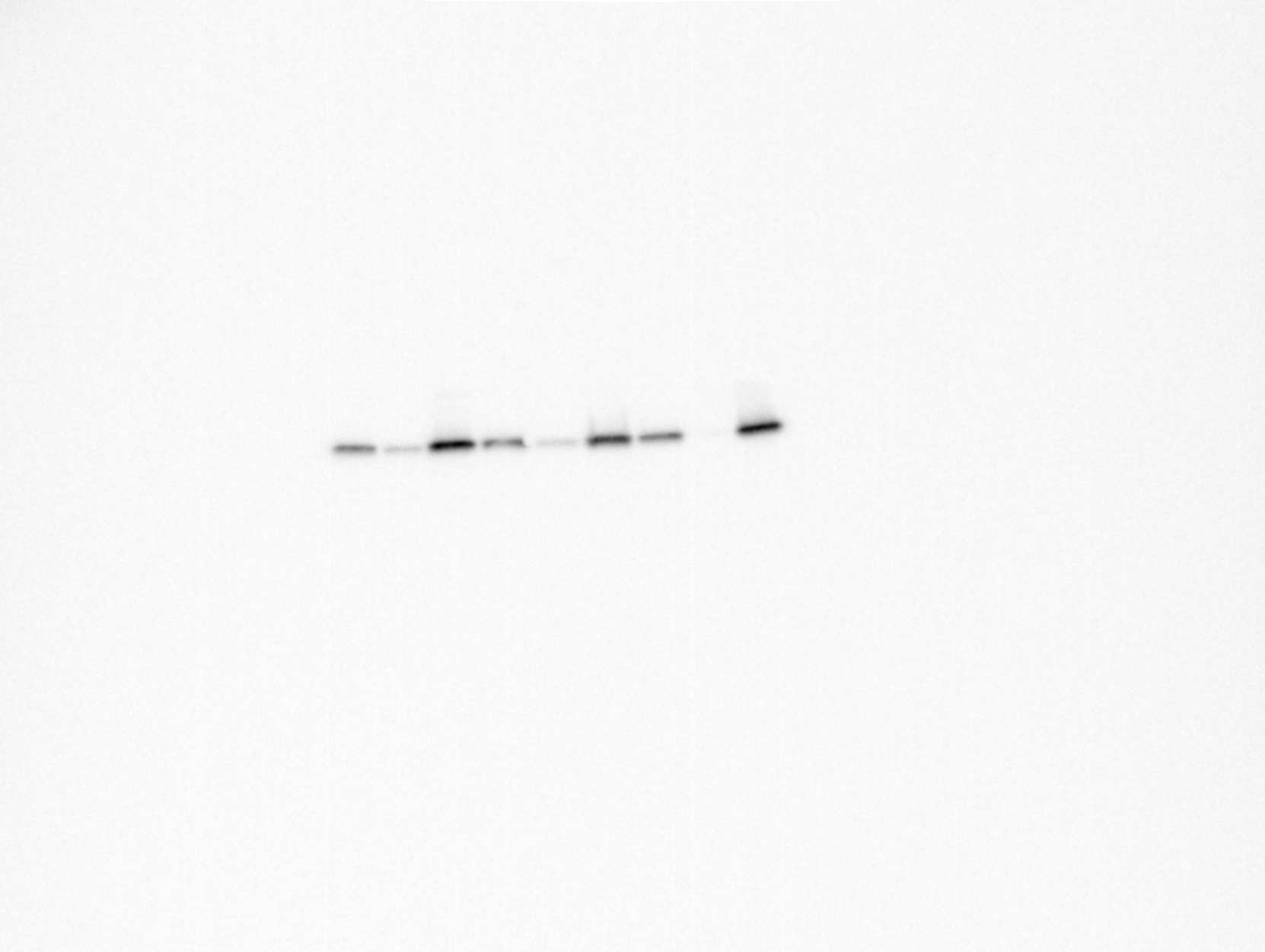

Supplement: Figure 1—figure supplement 1—source data 2. [file elife-100747-fig1-figsupp1-data2.zip › Figure 1 - Figure Supplement 1 - Source Data 2 (original western files)/Tim23_mito_pico/2022-0422-161438_pub.tif]

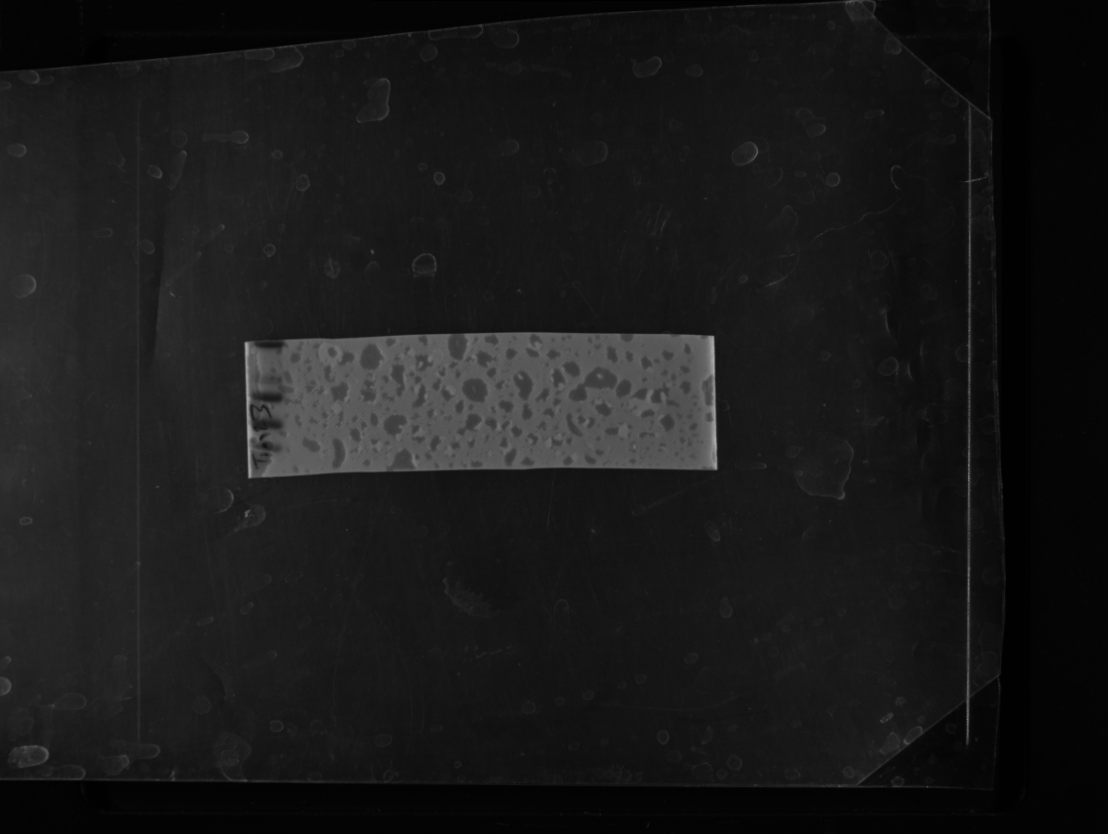

Supplement: Figure 1—figure supplement 1—source data 2. [file elife-100747-fig1-figsupp1-data2.zip › Figure 1 - Figure Supplement 1 - Source Data 2 (original western files)/Tim23_mito_pico/2022-0422-161440.tif]

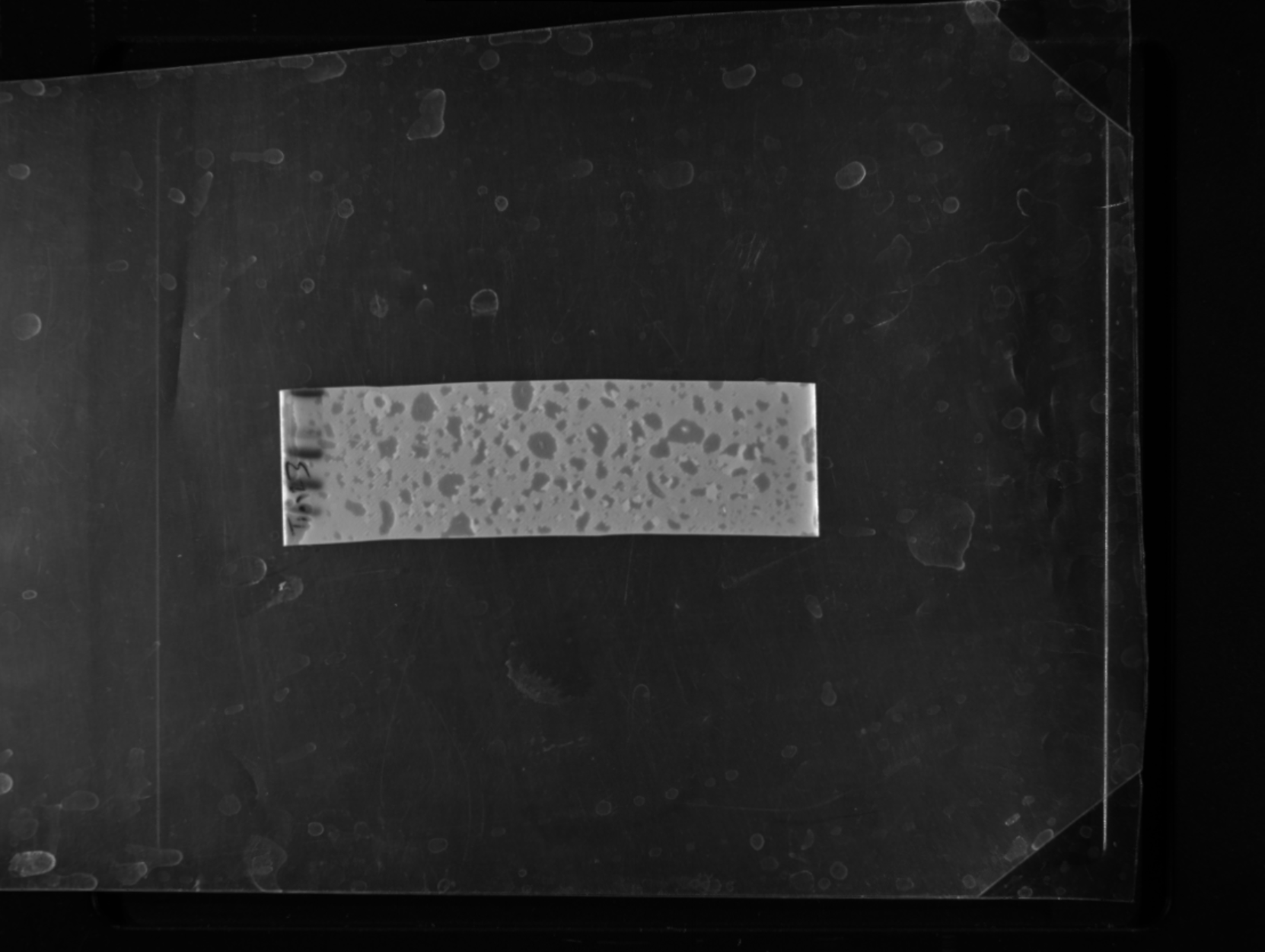

Supplement: Figure 1—figure supplement 1—source data 2. [file elife-100747-fig1-figsupp1-data2.zip › Figure 1 - Figure Supplement 1 - Source Data 2 (original western files)/Tim23_mito_pico/2022-0422-161440_pub.tif]

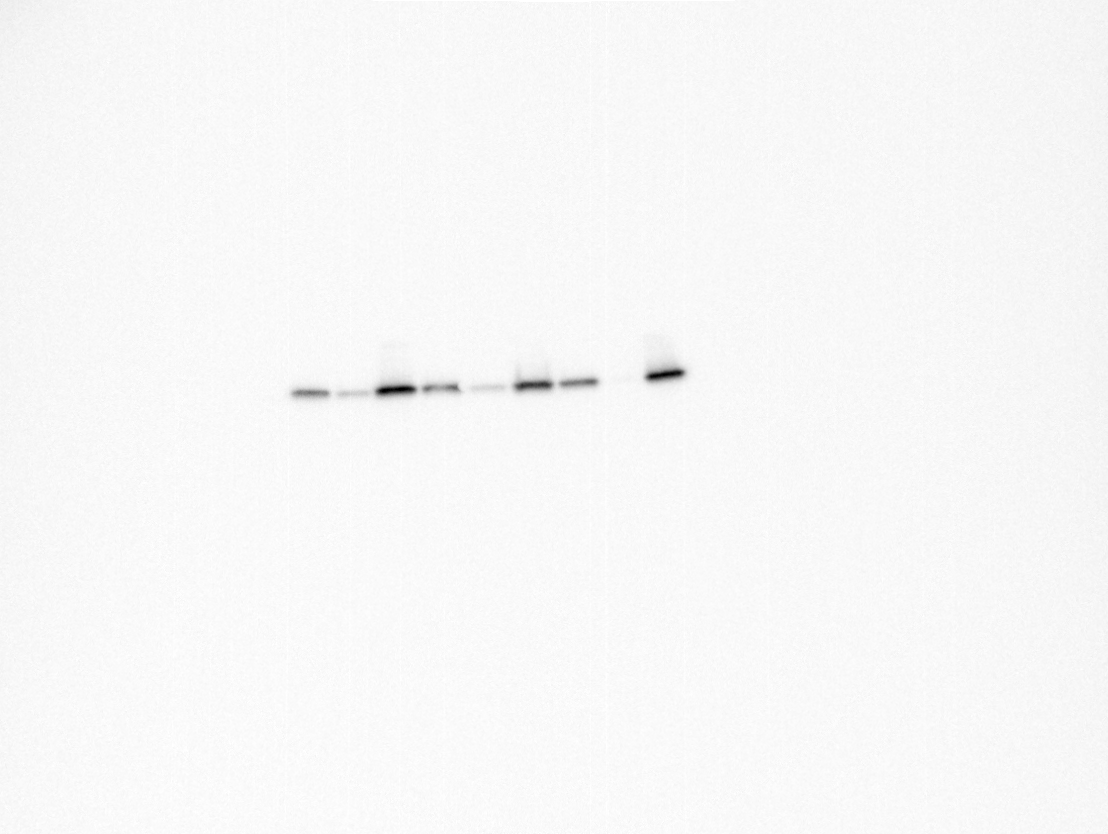

Supplement: Figure 1—figure supplement 1—source data 2. [file elife-100747-fig1-figsupp1-data2.zip › Figure 1 - Figure Supplement 1 - Source Data 2 (original western files)/Tim23_mito_pico/2022-0422-161441.tif]

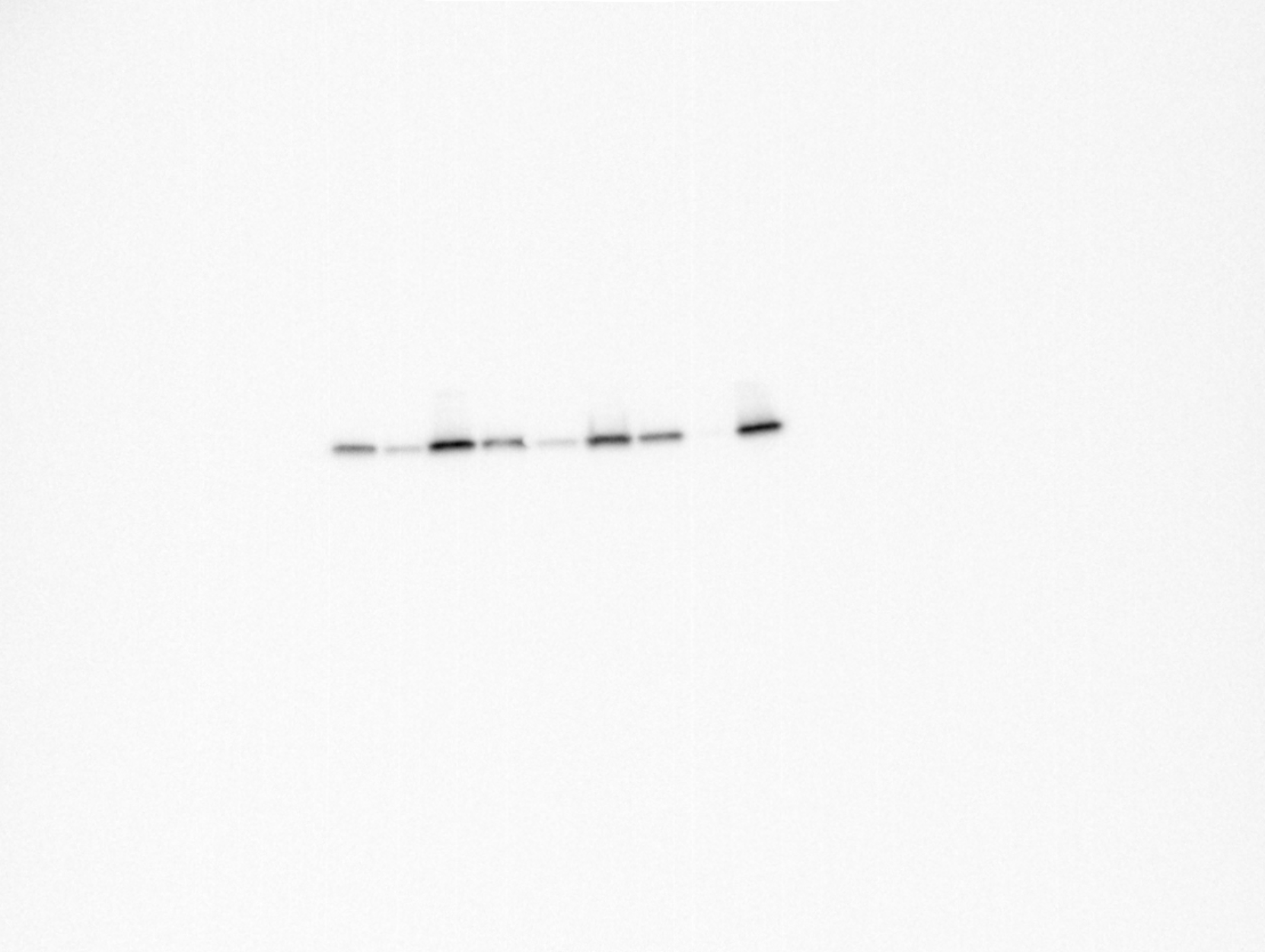

Supplement: Figure 1—figure supplement 1—source data 2. [file elife-100747-fig1-figsupp1-data2.zip › Figure 1 - Figure Supplement 1 - Source Data 2 (original western files)/Tim23_mito_pico/2022-0422-161441_pub.tif]

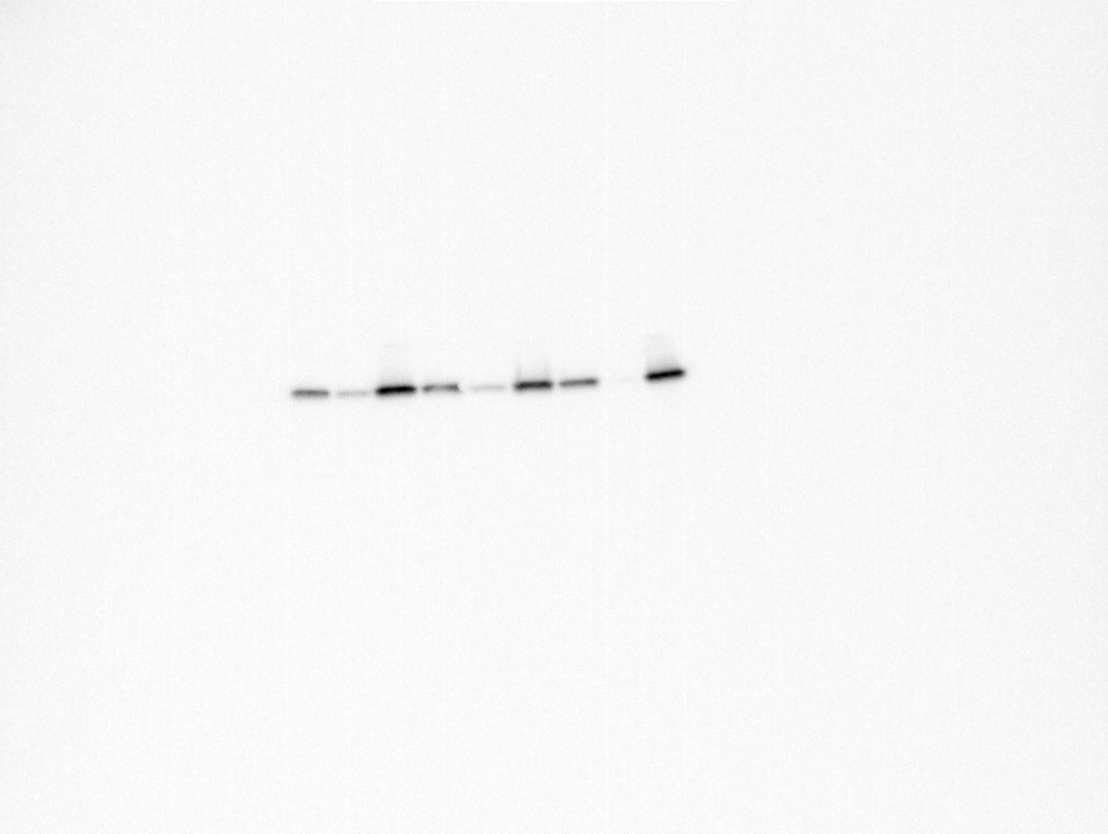

Supplement: Figure 1—figure supplement 1—source data 2. [file elife-100747-fig1-figsupp1-data2.zip › Figure 1 - Figure Supplement 1 - Source Data 2 (original western files)/Tim23_mito_pico/S2F1-0422-161443.tif]

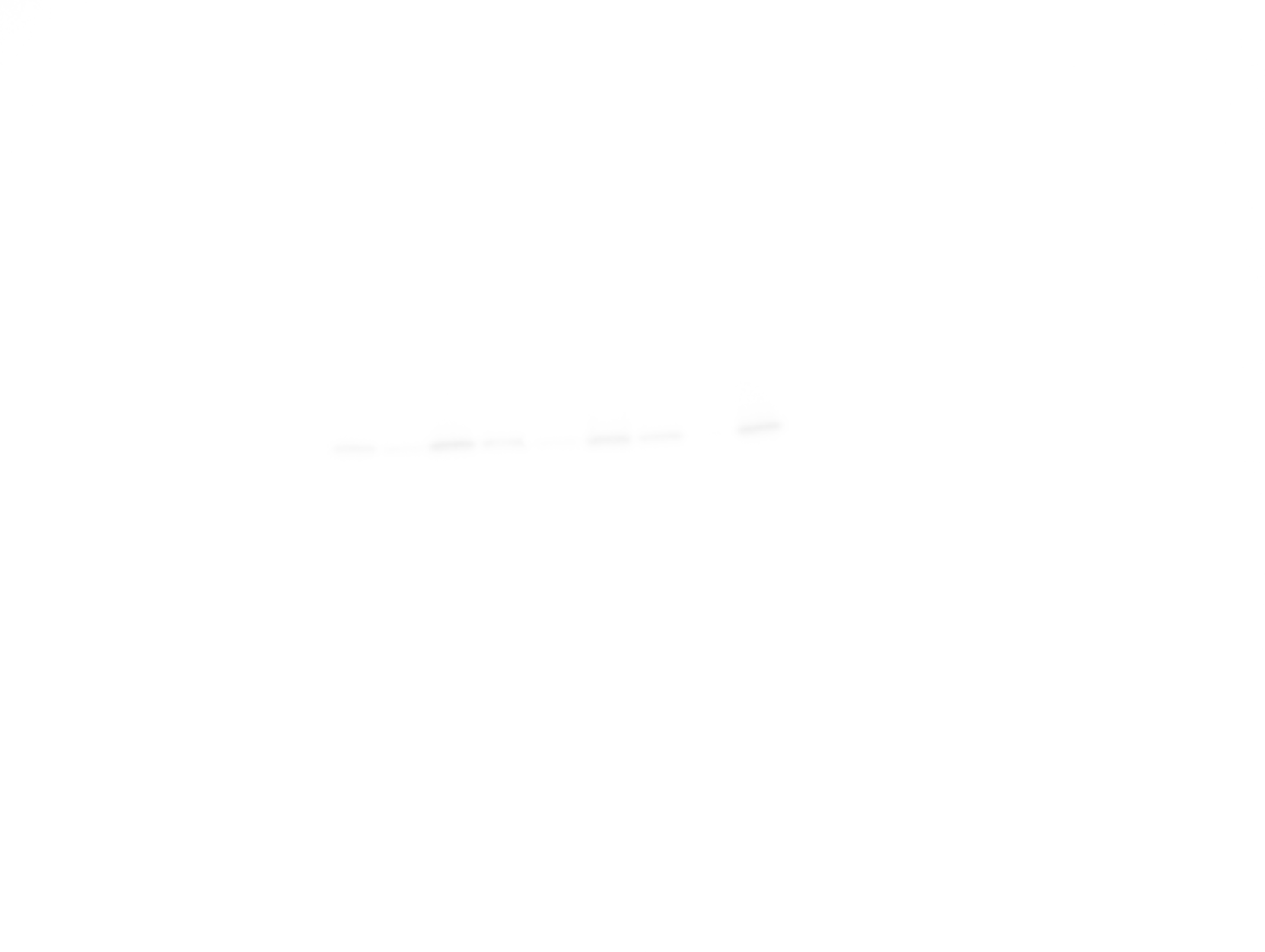

Supplement: Figure 1—figure supplement 1—source data 2. [file elife-100747-fig1-figsupp1-data2.zip › Figure 1 - Figure Supplement 1 - Source Data 2 (original western files)/Tim23_mito_pico/S2F1-0422-161443_pub.tif]

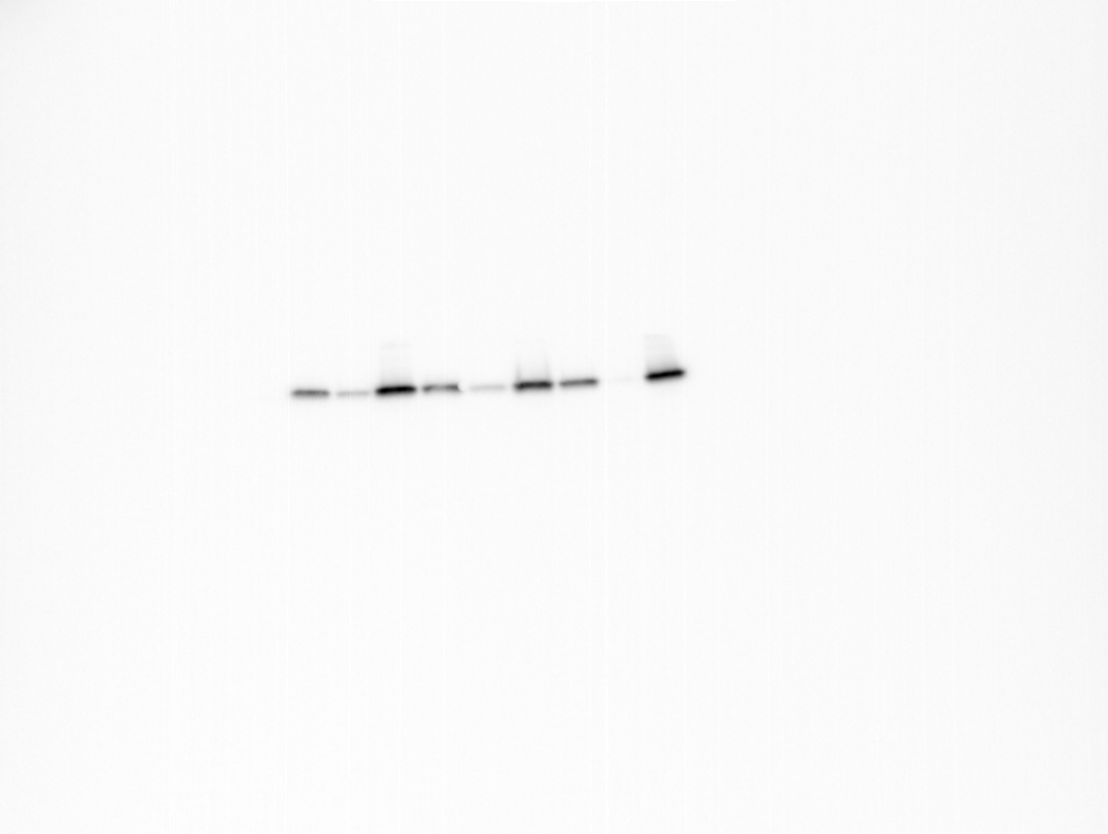

Supplement: Figure 1—figure supplement 1—source data 2. [file elife-100747-fig1-figsupp1-data2.zip › Figure 1 - Figure Supplement 1 - Source Data 2 (original western files)/Tim23_mito_pico/S2F10-0422-161458.tif]

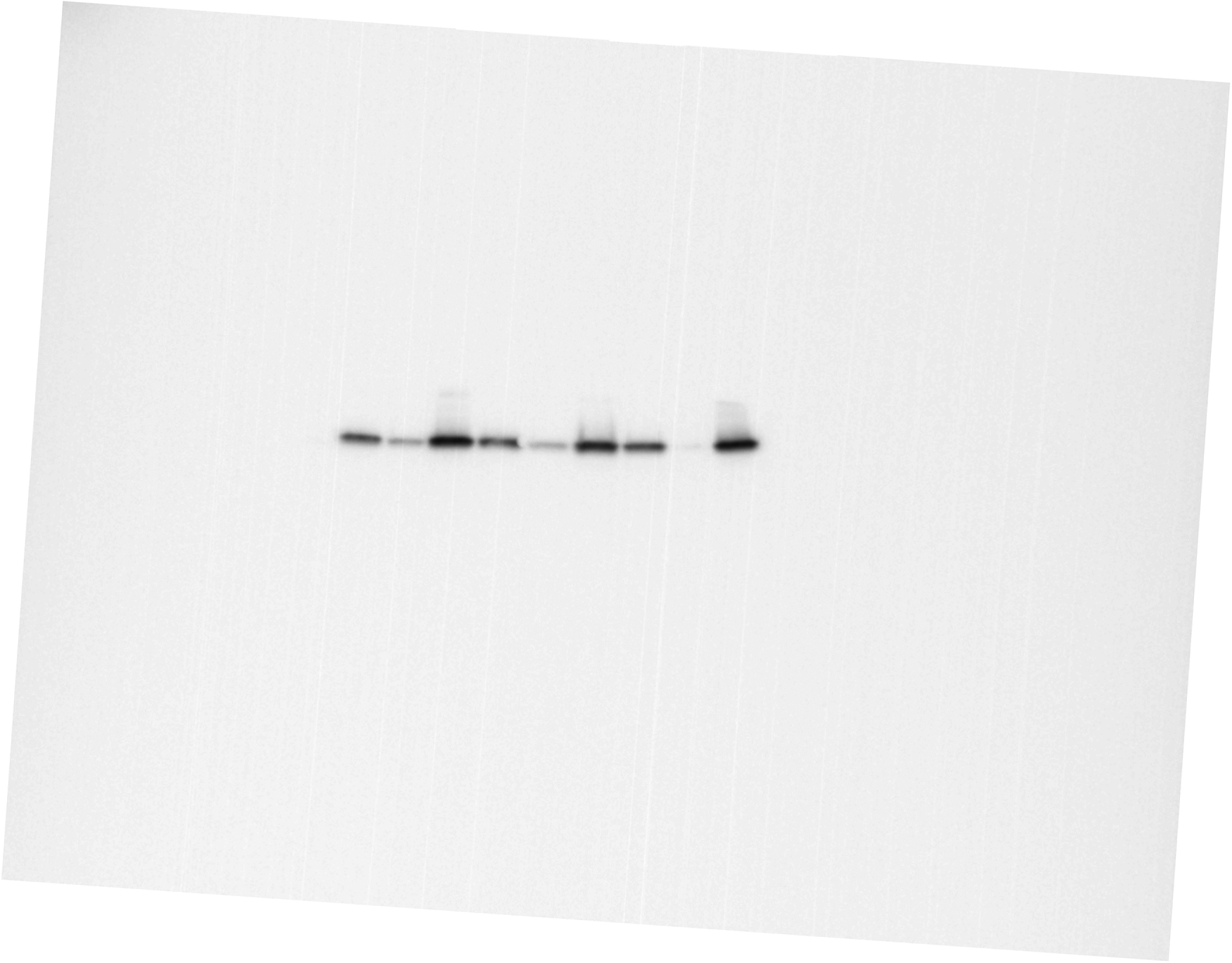

Supplement: Figure 1—figure supplement 1—source data 2. [file elife-100747-fig1-figsupp1-data2.zip › Figure 1 - Figure Supplement 1 - Source Data 2 (original western files)/Tim23_mito_pico/S2F10-0422-161458_pub.tif]

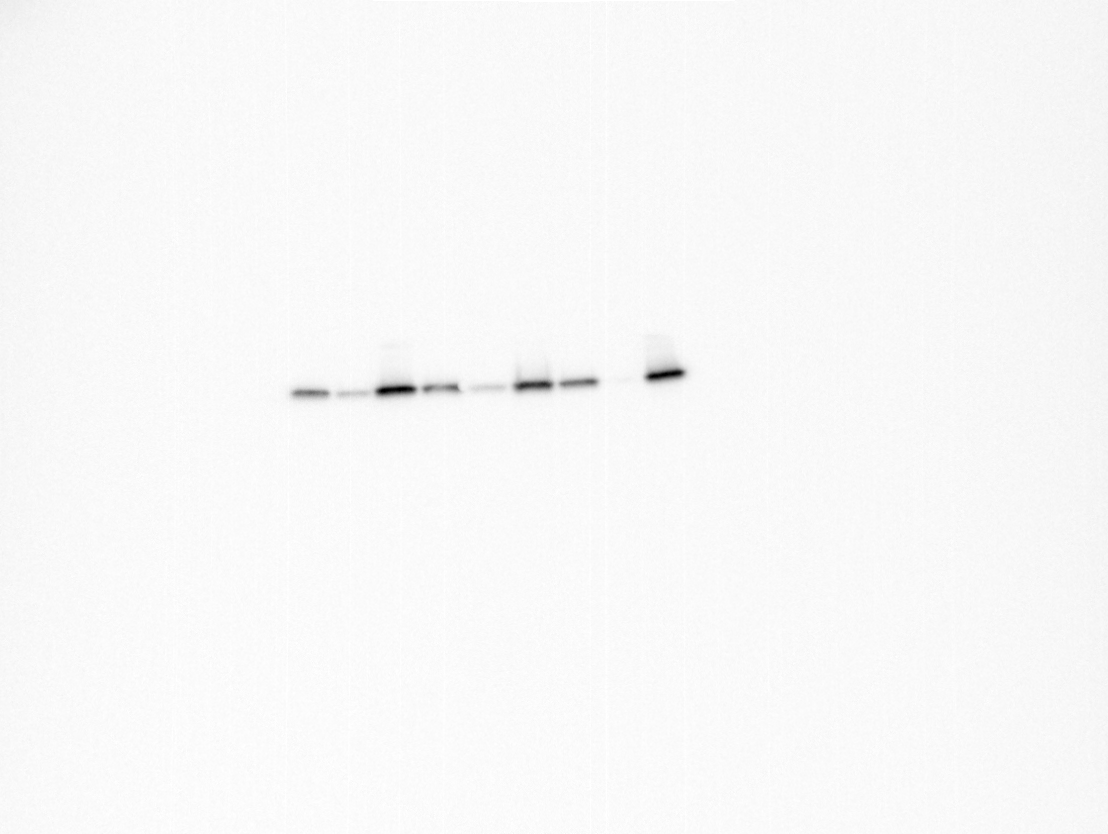

Supplement: Figure 1—figure supplement 1—source data 2. [file elife-100747-fig1-figsupp1-data2.zip › Figure 1 - Figure Supplement 1 - Source Data 2 (original western files)/Tim23_mito_pico/S2F2-0422-161445.tif]

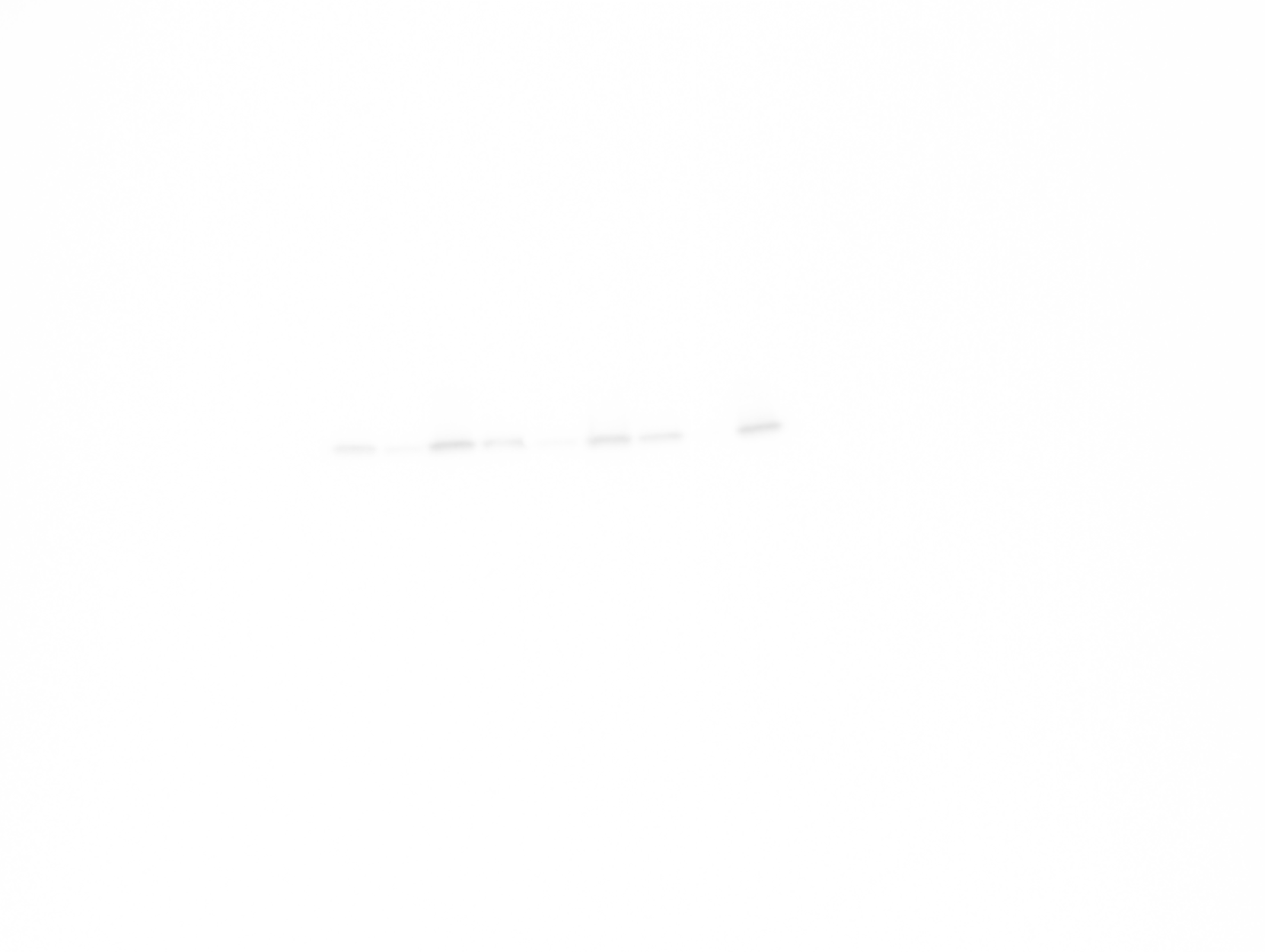

Supplement: Figure 1—figure supplement 1—source data 2. [file elife-100747-fig1-figsupp1-data2.zip › Figure 1 - Figure Supplement 1 - Source Data 2 (original western files)/Tim23_mito_pico/S2F2-0422-161445_pub.tif]

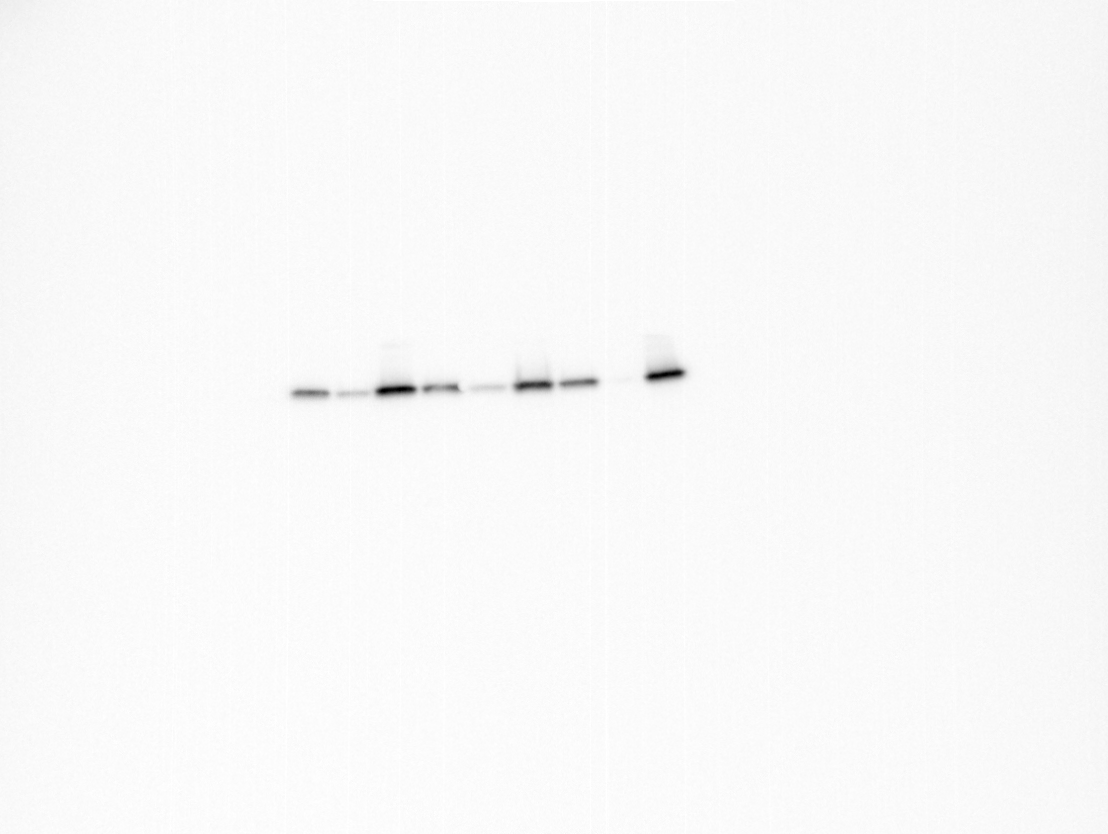

Supplement: Figure 1—figure supplement 1—source data 2. [file elife-100747-fig1-figsupp1-data2.zip › Figure 1 - Figure Supplement 1 - Source Data 2 (original western files)/Tim23_mito_pico/S2F3-0422-161446.tif]

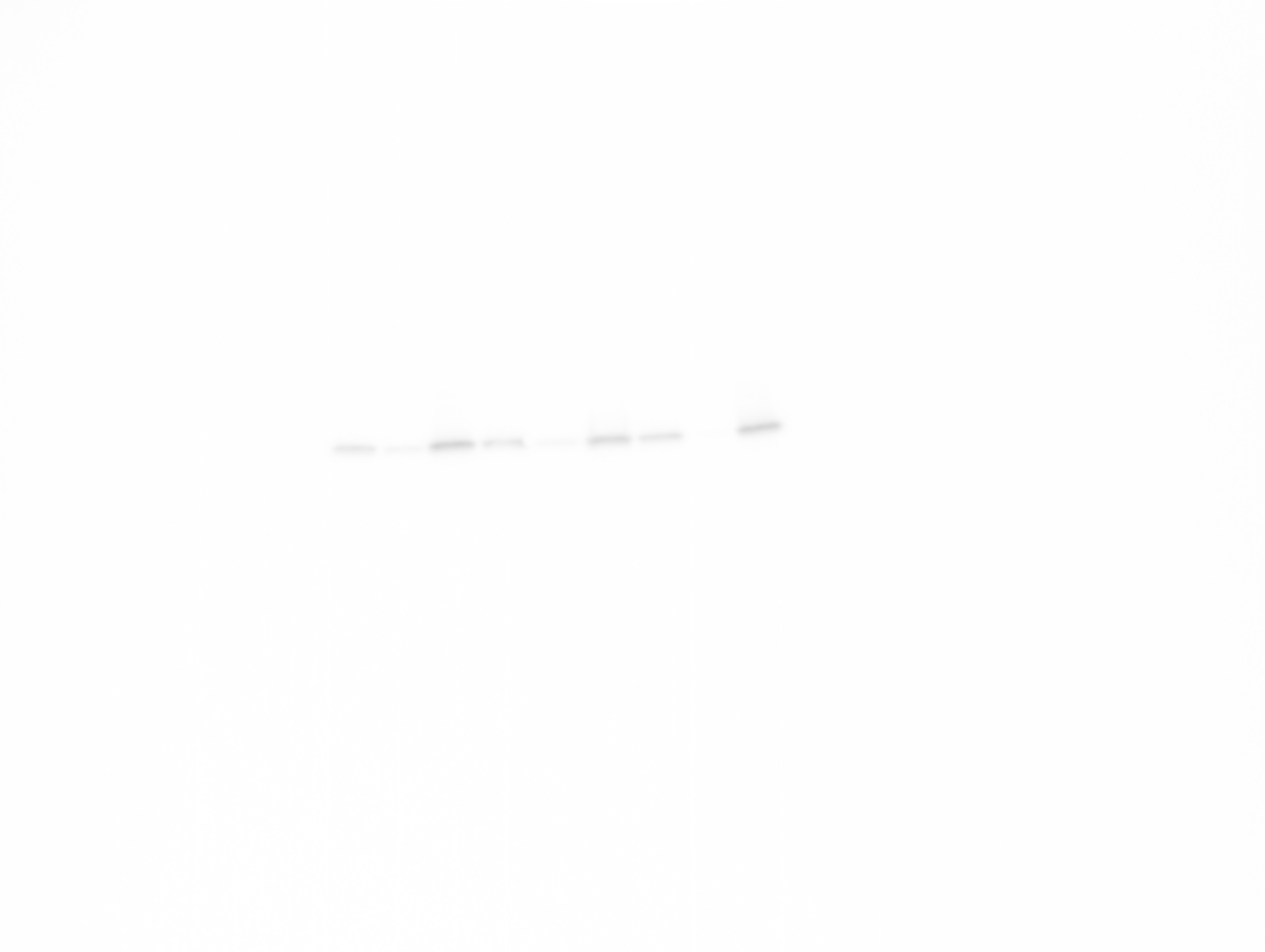

Supplement: Figure 1—figure supplement 1—source data 2. [file elife-100747-fig1-figsupp1-data2.zip › Figure 1 - Figure Supplement 1 - Source Data 2 (original western files)/Tim23_mito_pico/S2F3-0422-161446_pub.tif]

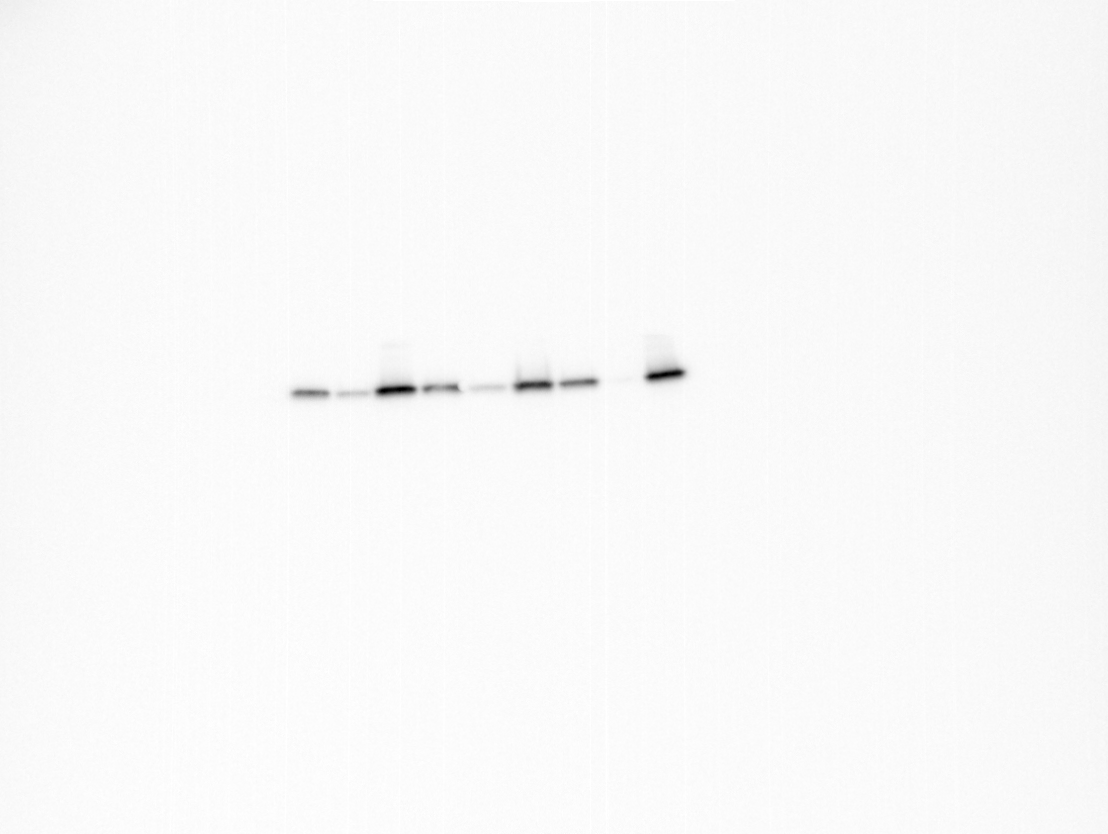

Supplement: Figure 1—figure supplement 1—source data 2. [file elife-100747-fig1-figsupp1-data2.zip › Figure 1 - Figure Supplement 1 - Source Data 2 (original western files)/Tim23_mito_pico/S2F4-0422-161448.tif]

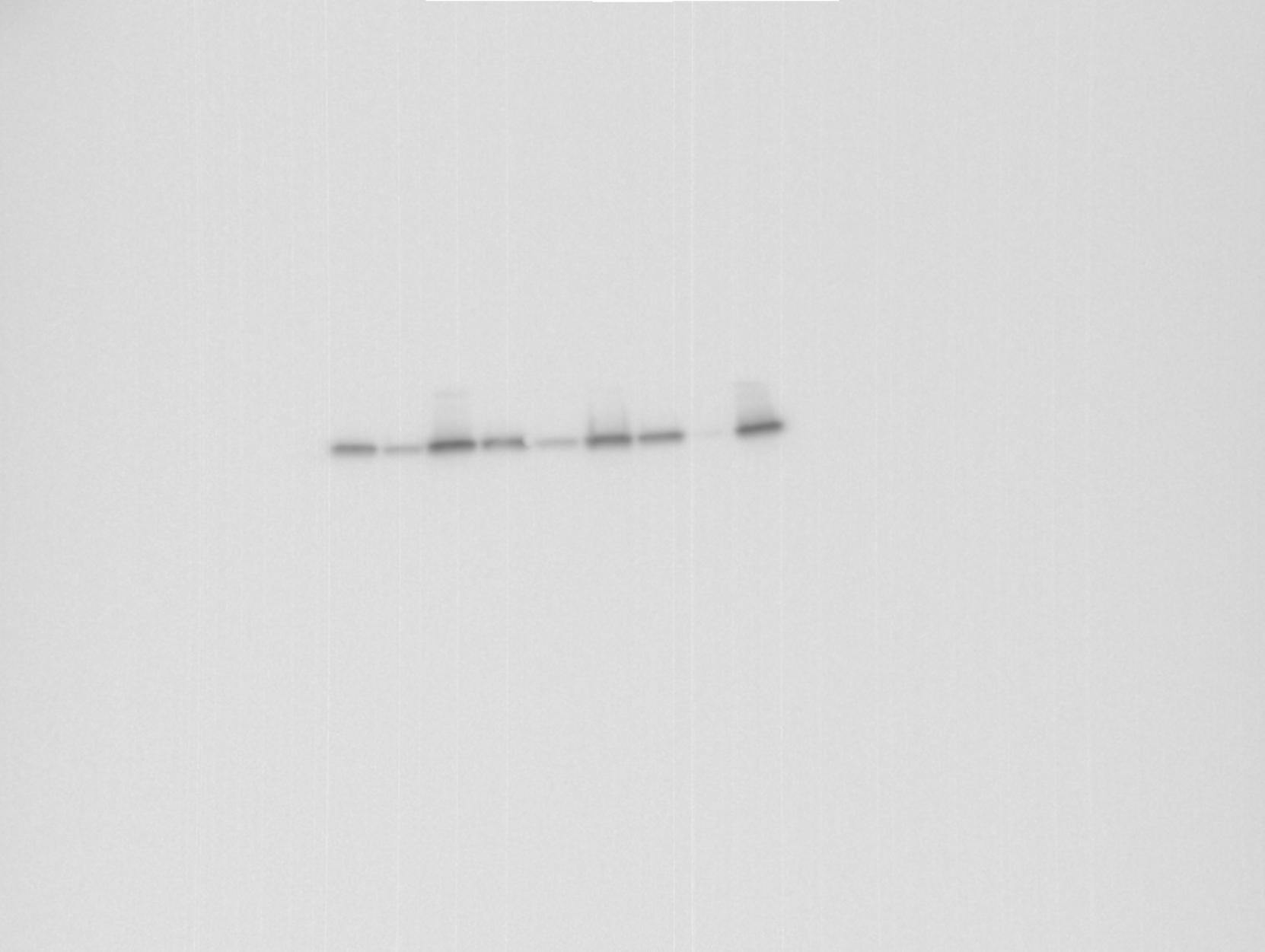

Supplement: Figure 1—figure supplement 1—source data 2. [file elife-100747-fig1-figsupp1-data2.zip › Figure 1 - Figure Supplement 1 - Source Data 2 (original western files)/Tim23_mito_pico/S2F4-0422-161448_pub.tif]

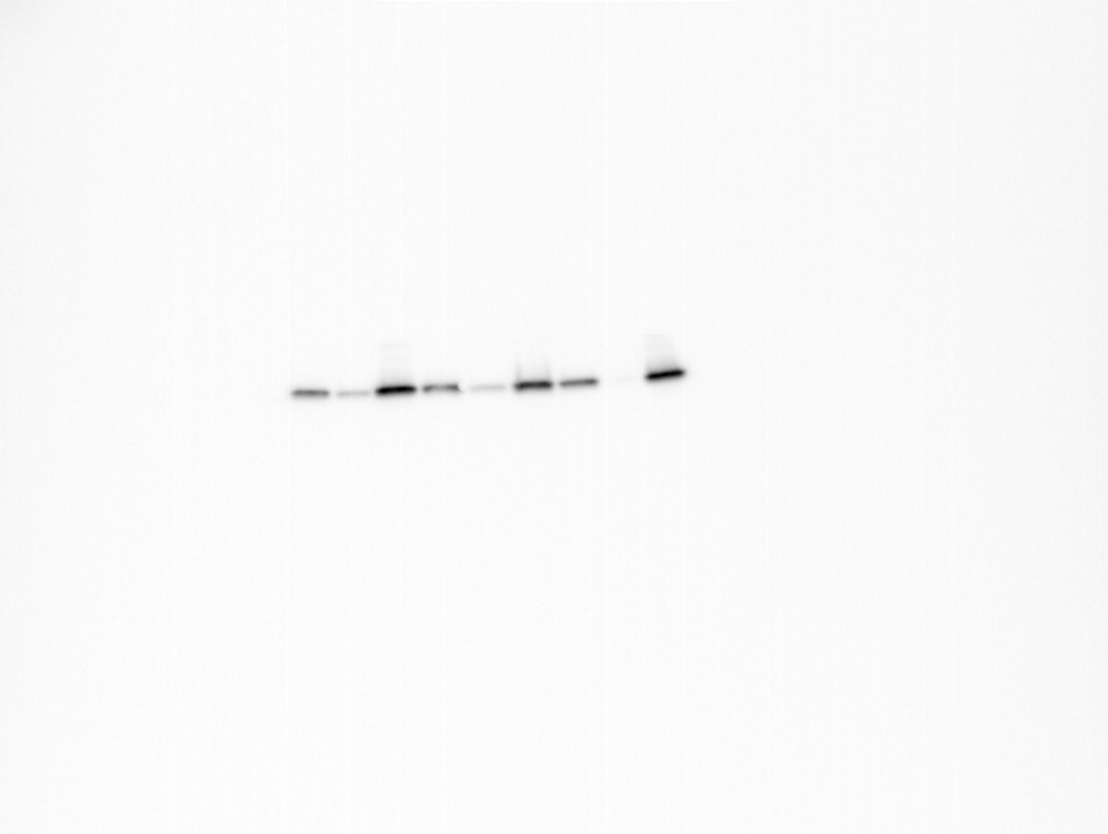

Supplement: Figure 1—figure supplement 1—source data 2. [file elife-100747-fig1-figsupp1-data2.zip › Figure 1 - Figure Supplement 1 - Source Data 2 (original western files)/Tim23_mito_pico/S2F5-0422-161450.tif]

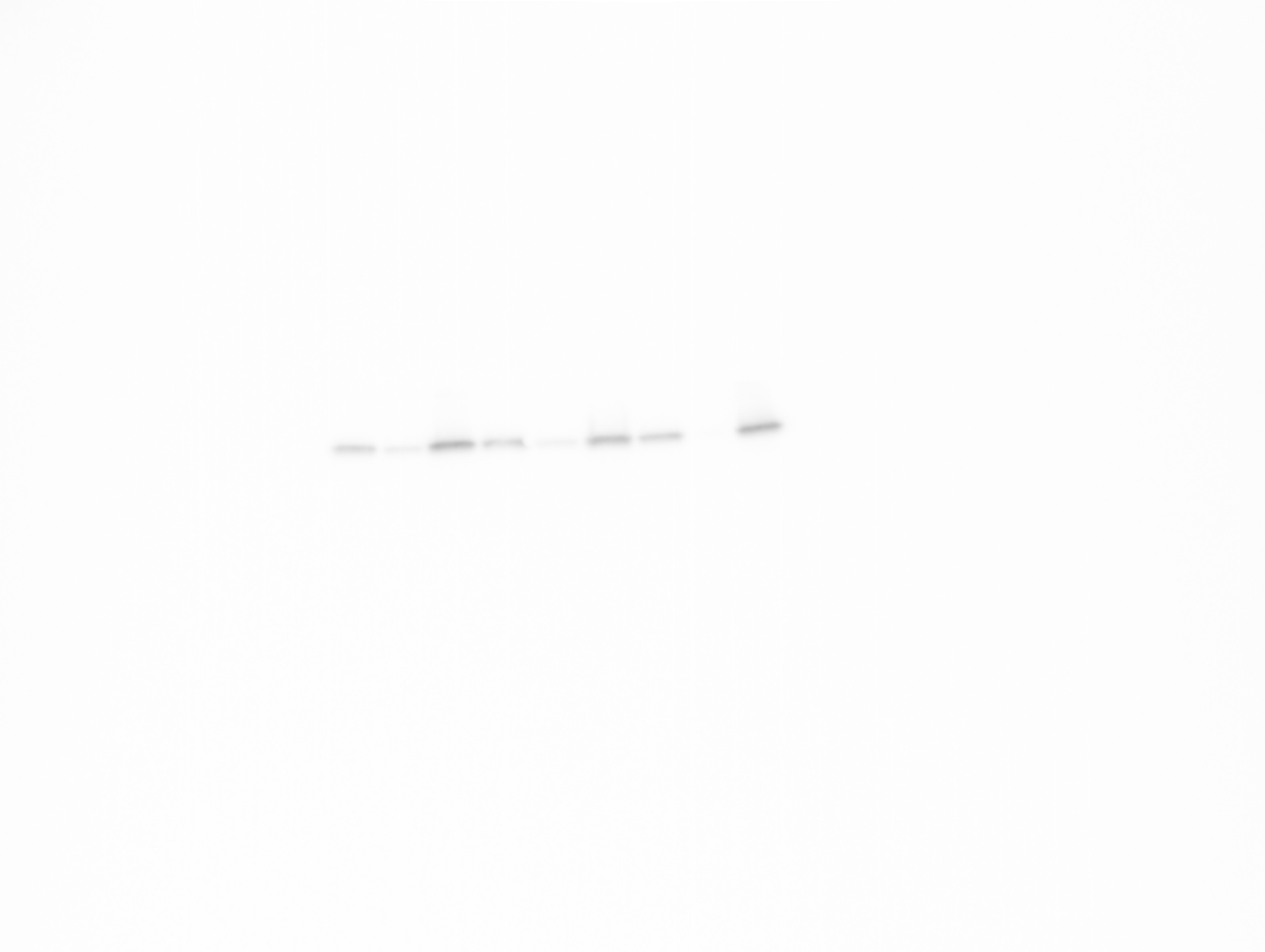

Supplement: Figure 1—figure supplement 1—source data 2. [file elife-100747-fig1-figsupp1-data2.zip › Figure 1 - Figure Supplement 1 - Source Data 2 (original western files)/Tim23_mito_pico/S2F5-0422-161450_pub.tif]

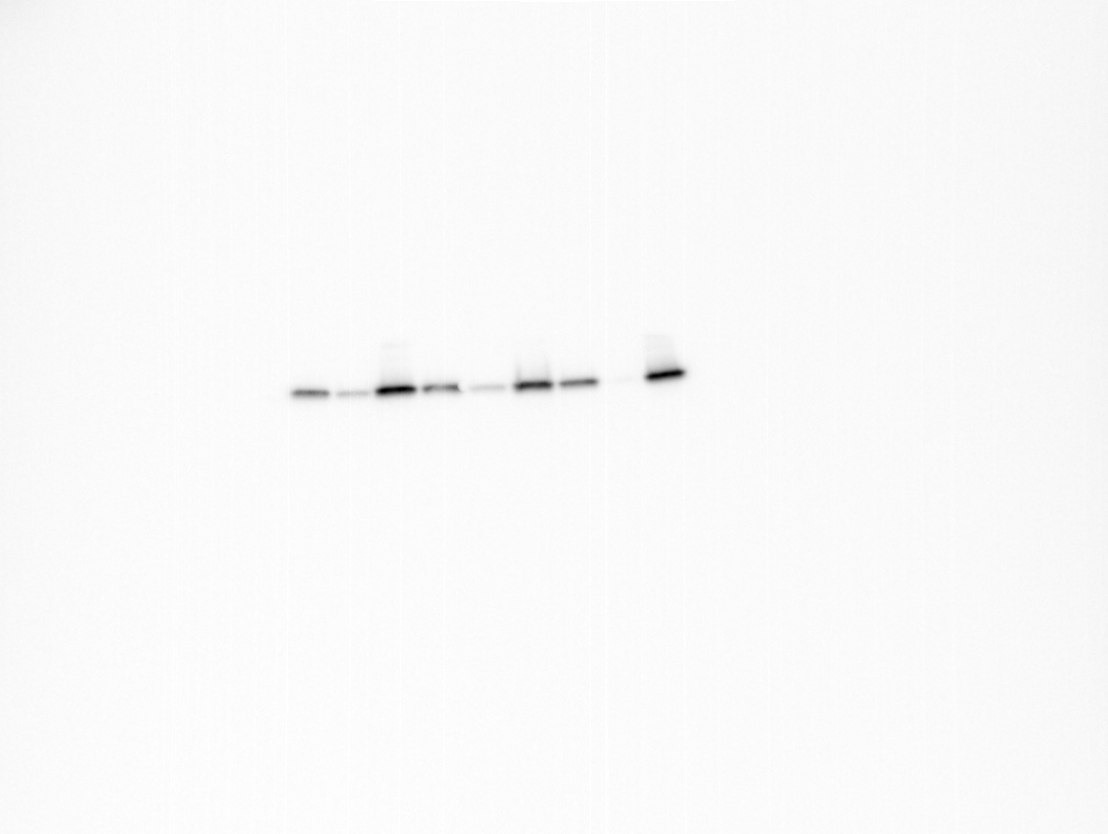

Supplement: Figure 1—figure supplement 1—source data 2. [file elife-100747-fig1-figsupp1-data2.zip › Figure 1 - Figure Supplement 1 - Source Data 2 (original western files)/Tim23_mito_pico/S2F6-0422-161451.tif]

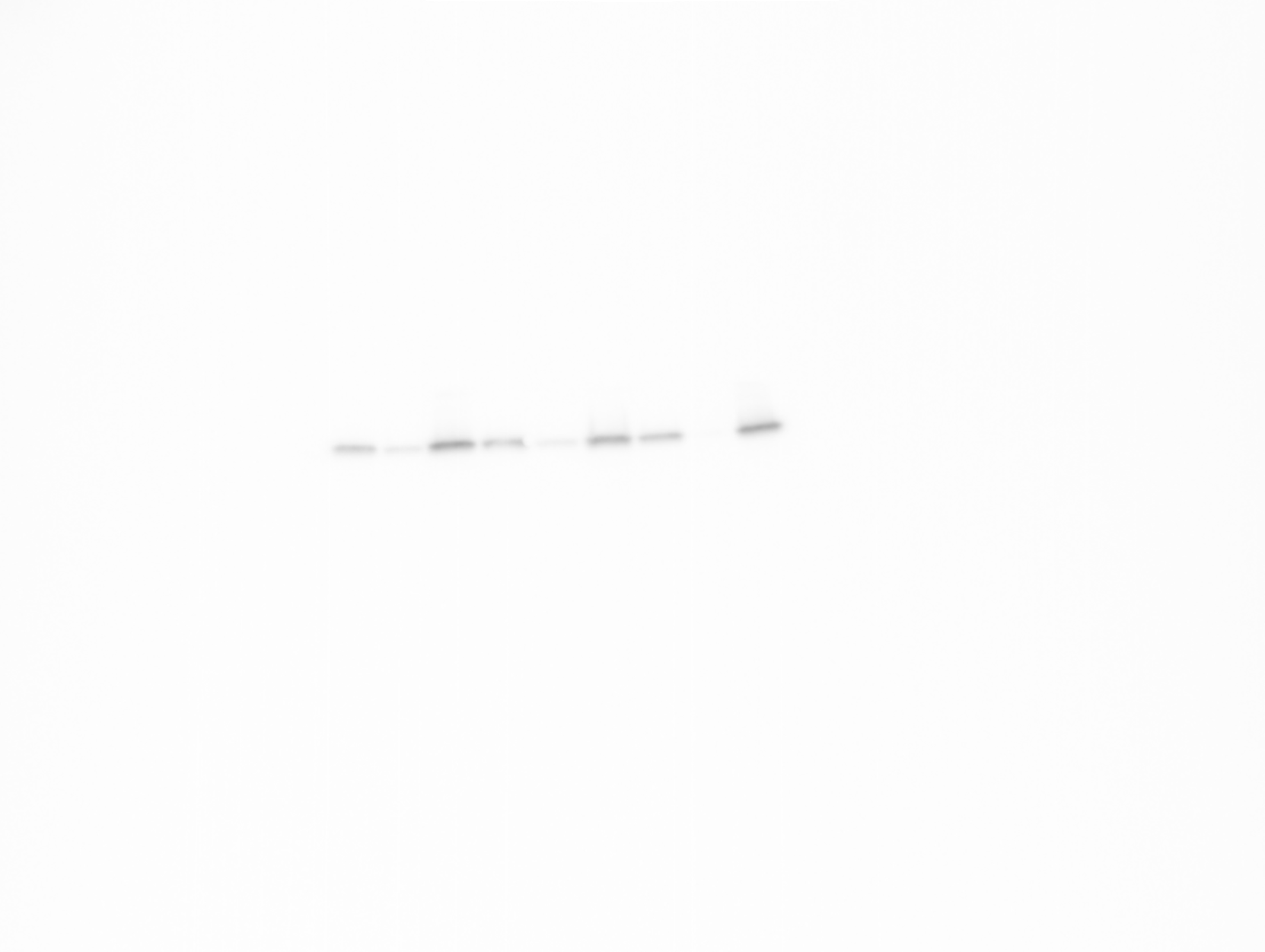

Supplement: Figure 1—figure supplement 1—source data 2. [file elife-100747-fig1-figsupp1-data2.zip › Figure 1 - Figure Supplement 1 - Source Data 2 (original western files)/Tim23_mito_pico/S2F6-0422-161451_pub.tif]

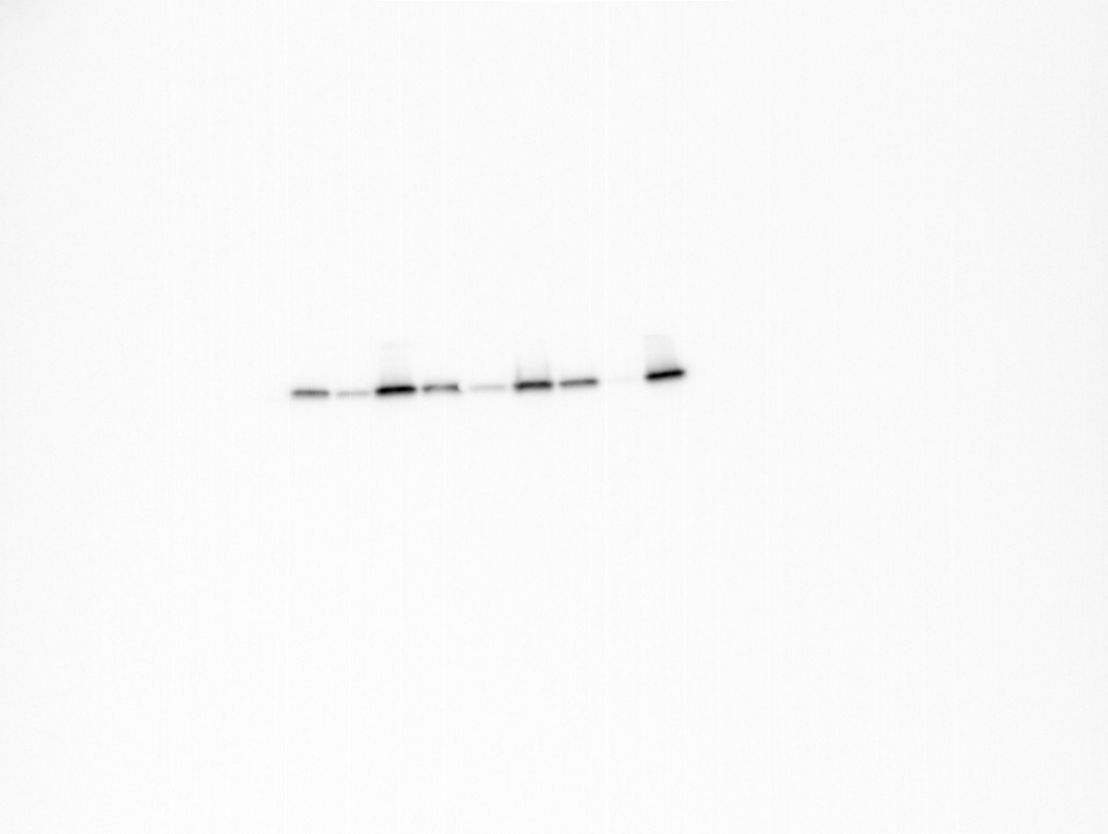

Supplement: Figure 1—figure supplement 1—source data 2. [file elife-100747-fig1-figsupp1-data2.zip › Figure 1 - Figure Supplement 1 - Source Data 2 (original western files)/Tim23_mito_pico/S2F7-0422-161453.tif]

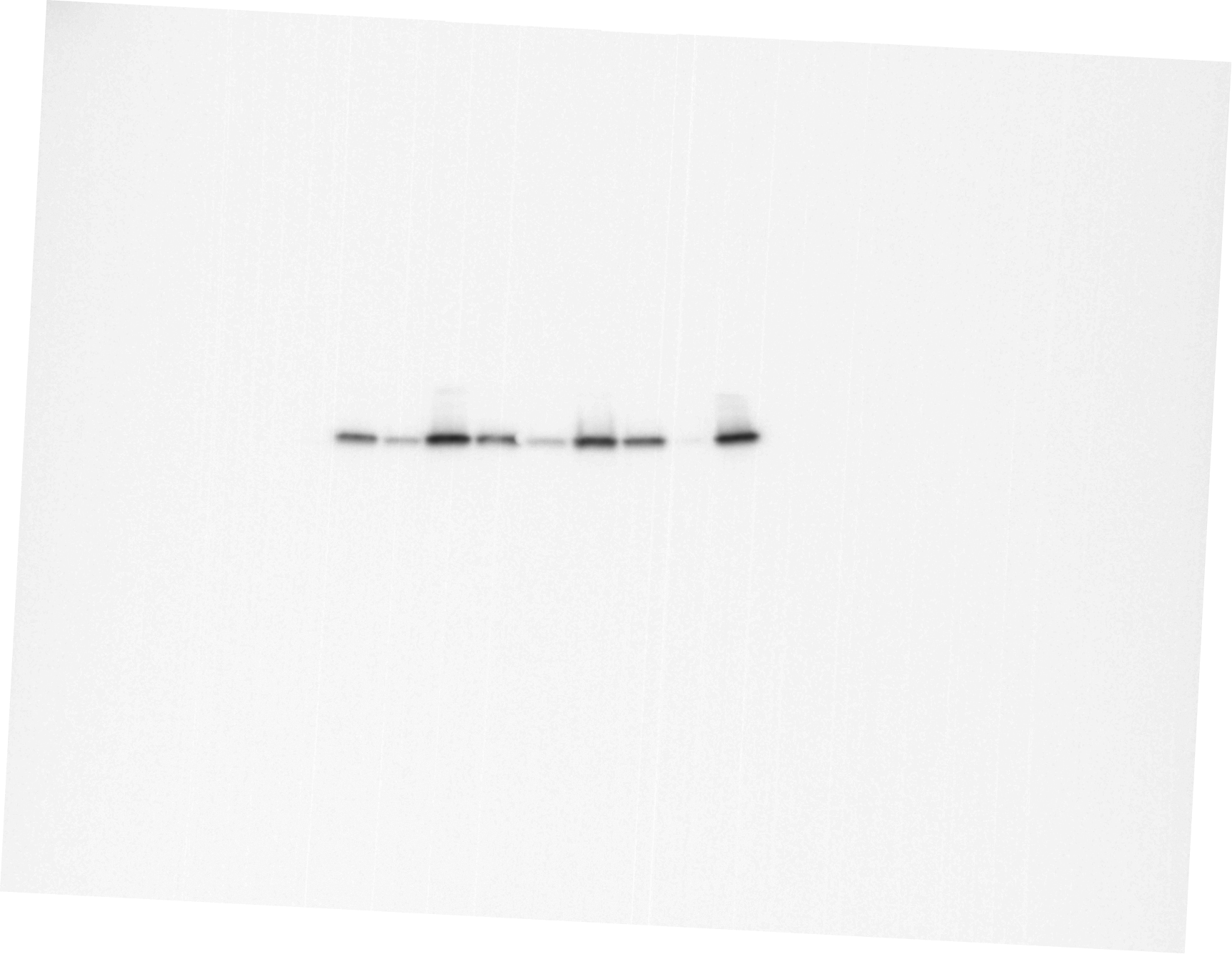

Supplement: Figure 1—figure supplement 1—source data 2. [file elife-100747-fig1-figsupp1-data2.zip › Figure 1 - Figure Supplement 1 - Source Data 2 (original western files)/Tim23_mito_pico/S2F7-0422-161453_pub.tif]

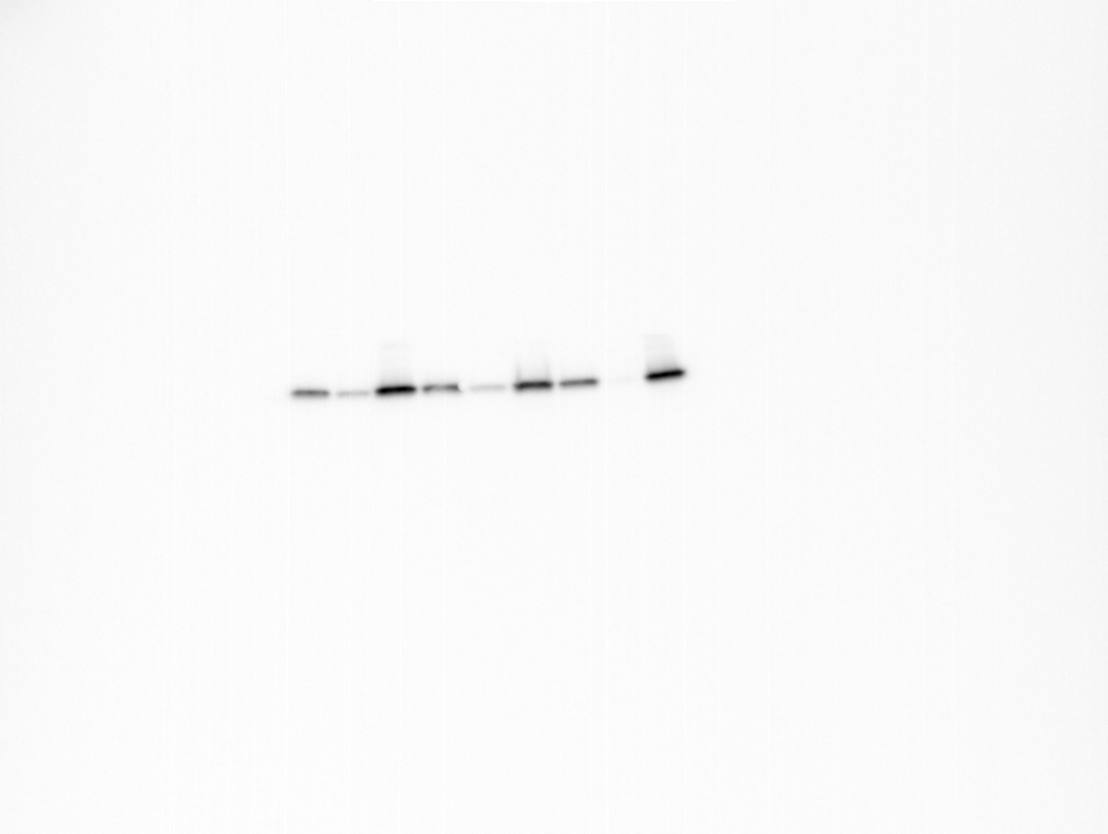

Supplement: Figure 1—figure supplement 1—source data 2. [file elife-100747-fig1-figsupp1-data2.zip › Figure 1 - Figure Supplement 1 - Source Data 2 (original western files)/Tim23_mito_pico/S2F8-0422-161455.tif]

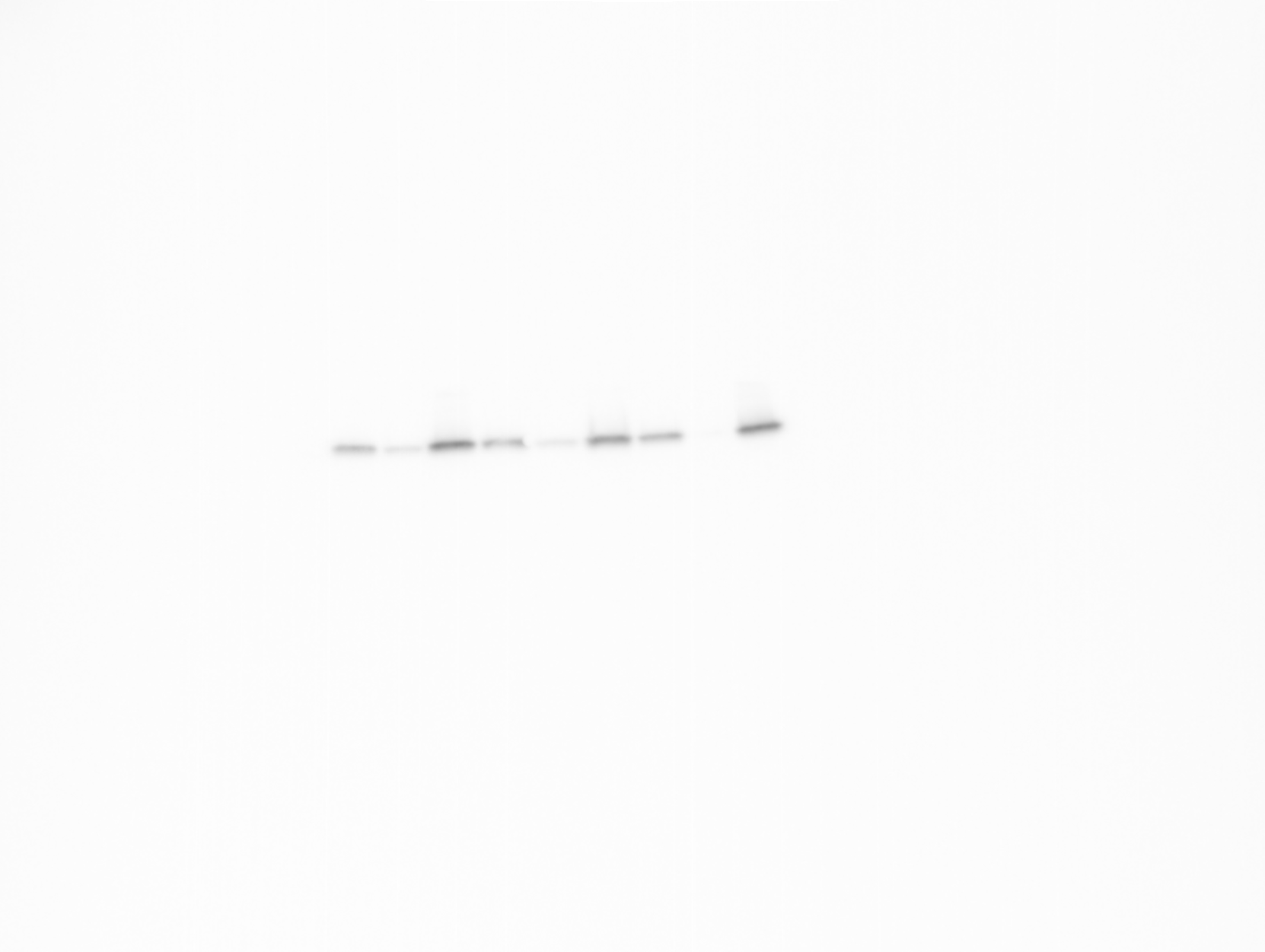

Supplement: Figure 1—figure supplement 1—source data 2. [file elife-100747-fig1-figsupp1-data2.zip › Figure 1 - Figure Supplement 1 - Source Data 2 (original western files)/Tim23_mito_pico/S2F8-0422-161455_pub.tif]

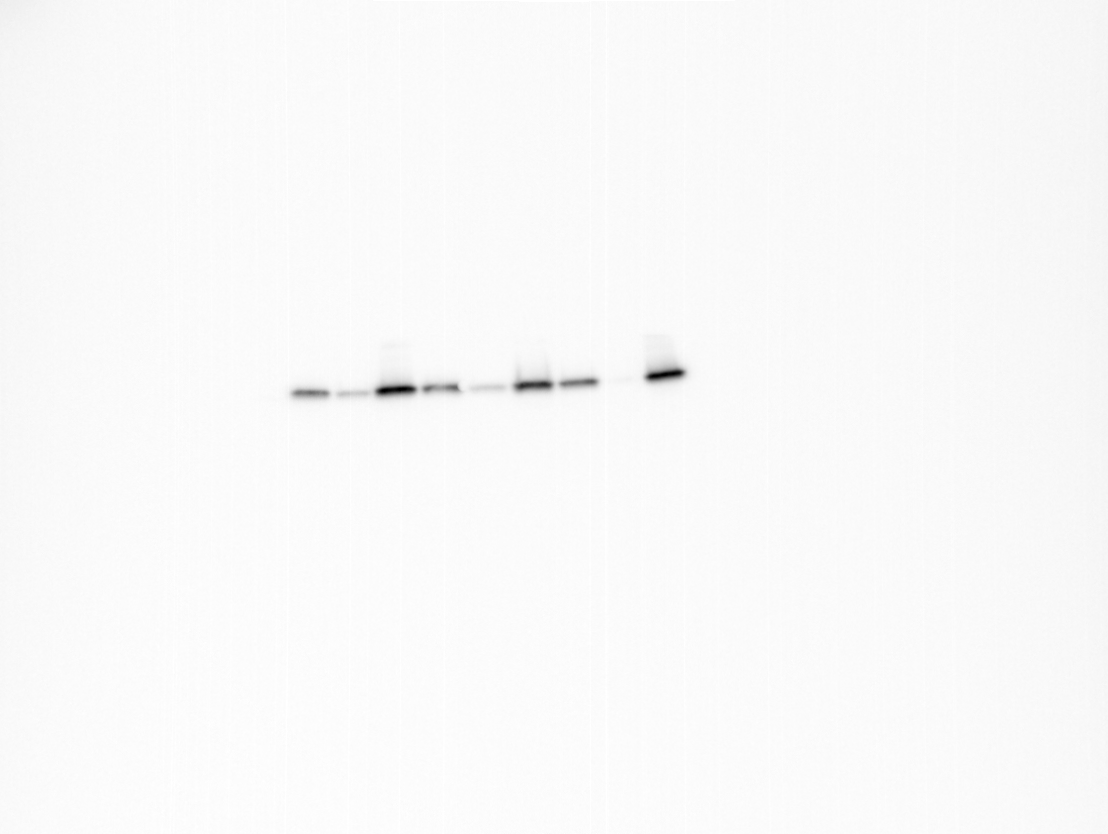

Supplement: Figure 1—figure supplement 1—source data 2. [file elife-100747-fig1-figsupp1-data2.zip › Figure 1 - Figure Supplement 1 - Source Data 2 (original western files)/Tim23_mito_pico/S2F9-0422-161456.tif]

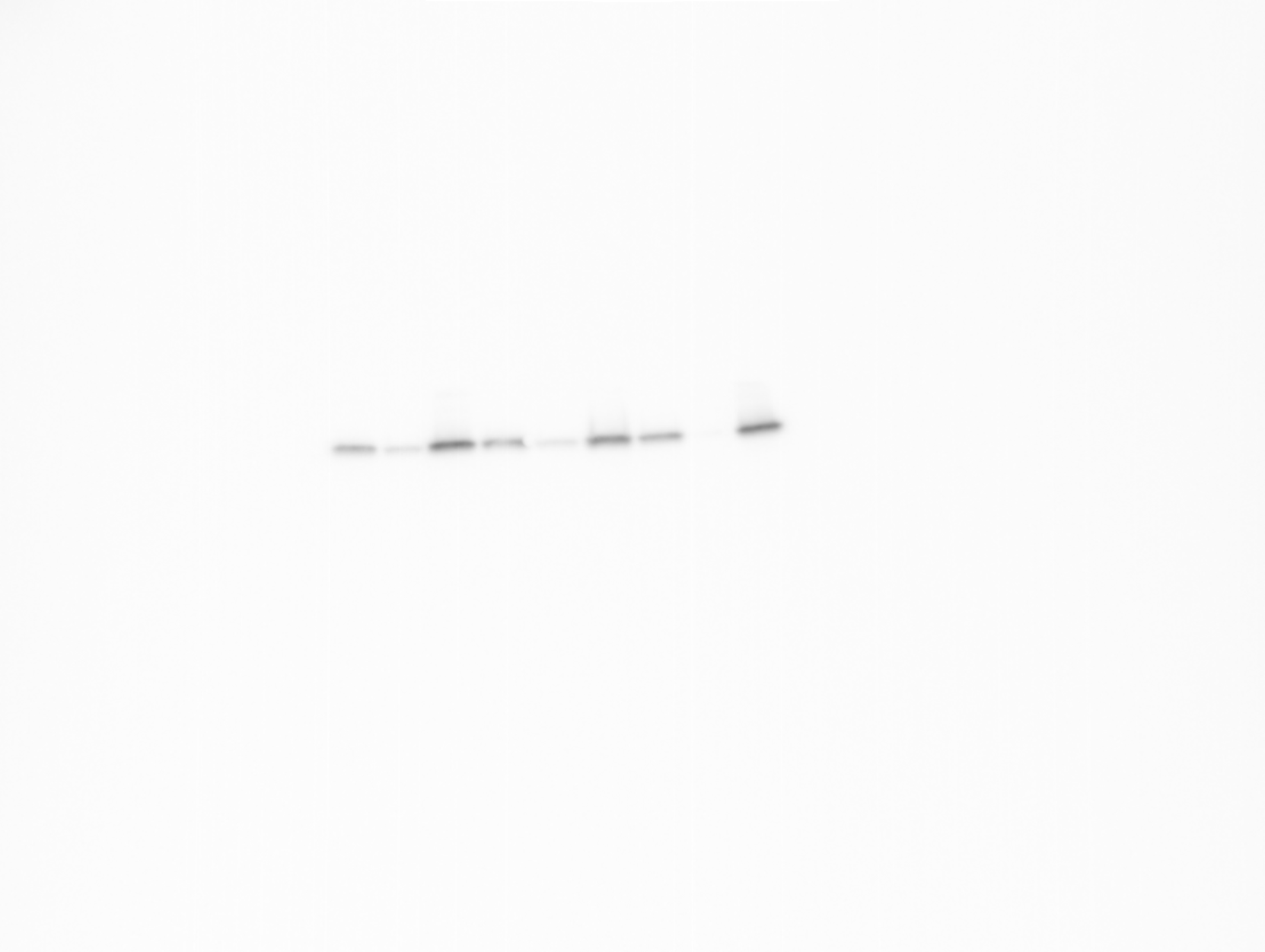

Supplement: Figure 1—figure supplement 1—source data 2. [file elife-100747-fig1-figsupp1-data2.zip › Figure 1 - Figure Supplement 1 - Source Data 2 (original western files)/Tim23_mito_pico/S2F9-0422-161456_pub.tif]

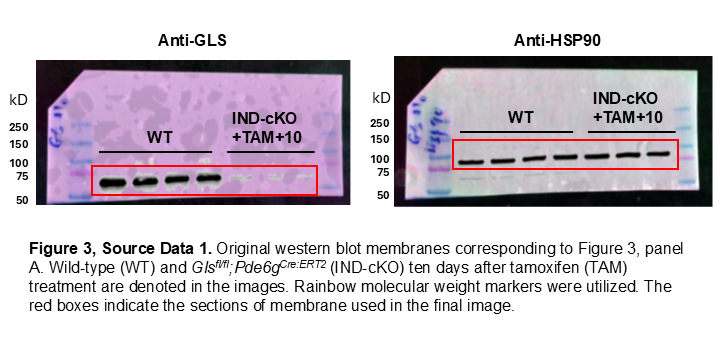

Supplement: Figure 3—source data 1. — Wild-type (WT) and Glsfl/fl;Pde6gCre:ERT2 (IND-cKO) 10 days after tamoxifen (TAM) treatment are denoted in the images. Rainbow molecular weight markers were utilized. The red boxes indicate the sections of membrane used in the final image. [file elife-100747-fig3-data1.zip › Figure 3 - Source Data 1 (annotated western file)/Figure 3 - Source Data 1.tif]

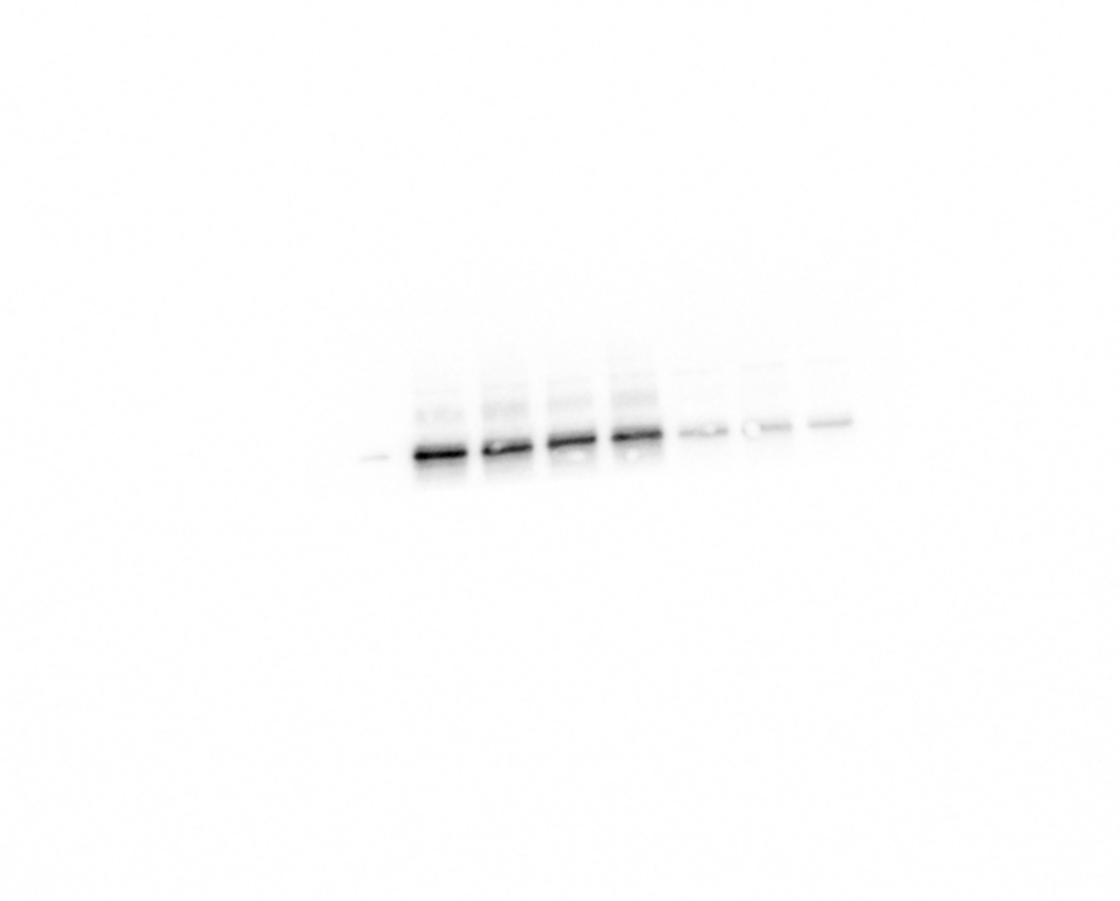

Supplement: Figure 3—source data 2. [file elife-100747-fig3-data2.zip › Figure 3 - Source Data 2 (original western files)/gls/23.07.26_15.19.01.tif]

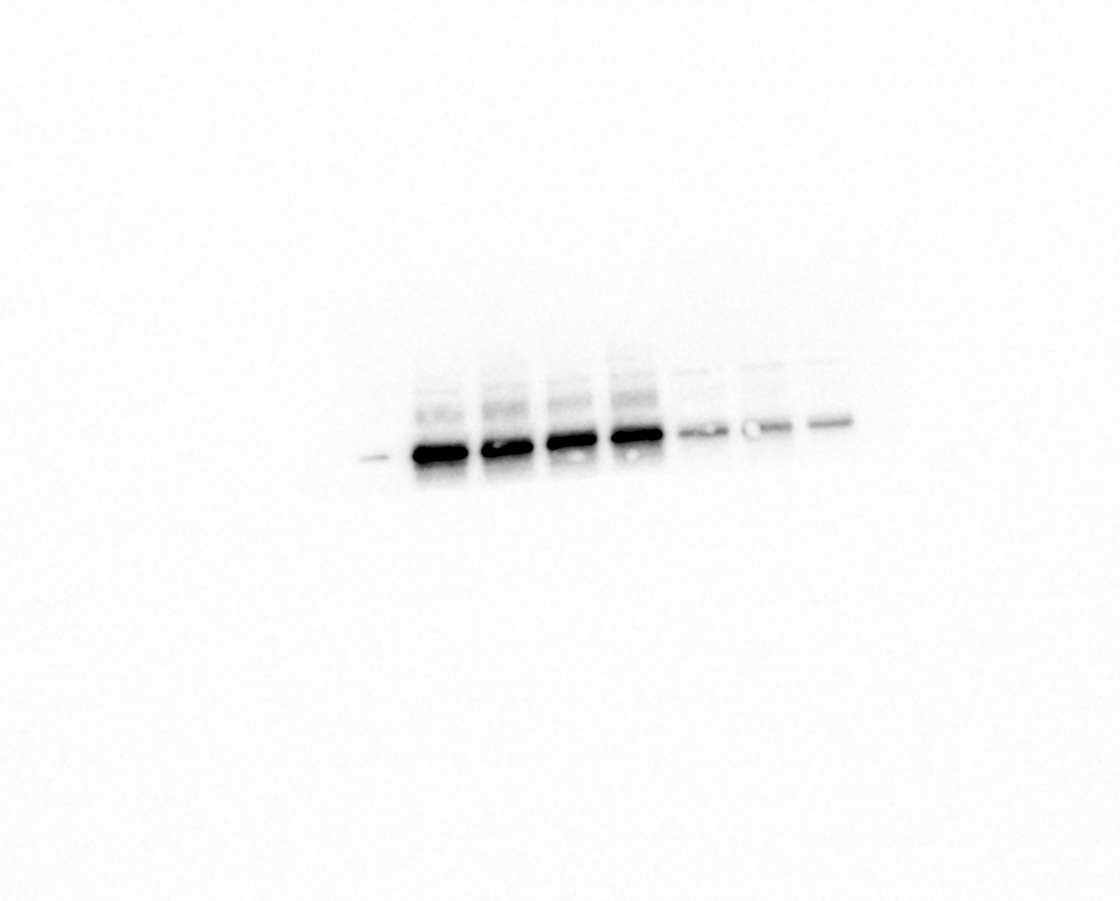

Supplement: Figure 3—source data 2. [file elife-100747-fig3-data2.zip › Figure 3 - Source Data 2 (original western files)/gls/23.07.26_15.19.01_PUB_600.tif]

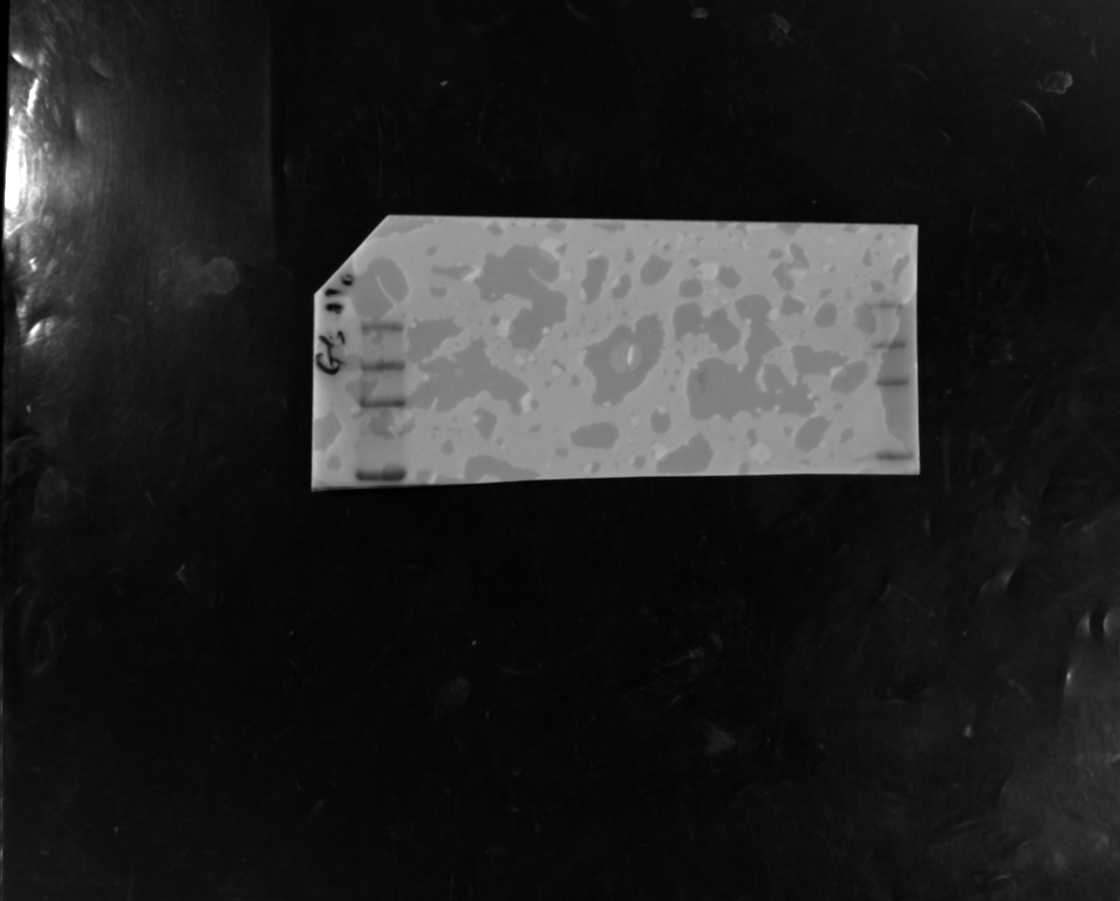

Supplement: Figure 3—source data 2. [file elife-100747-fig3-data2.zip › Figure 3 - Source Data 2 (original western files)/gls/23.07.26_15.22.05+Marker.tif]

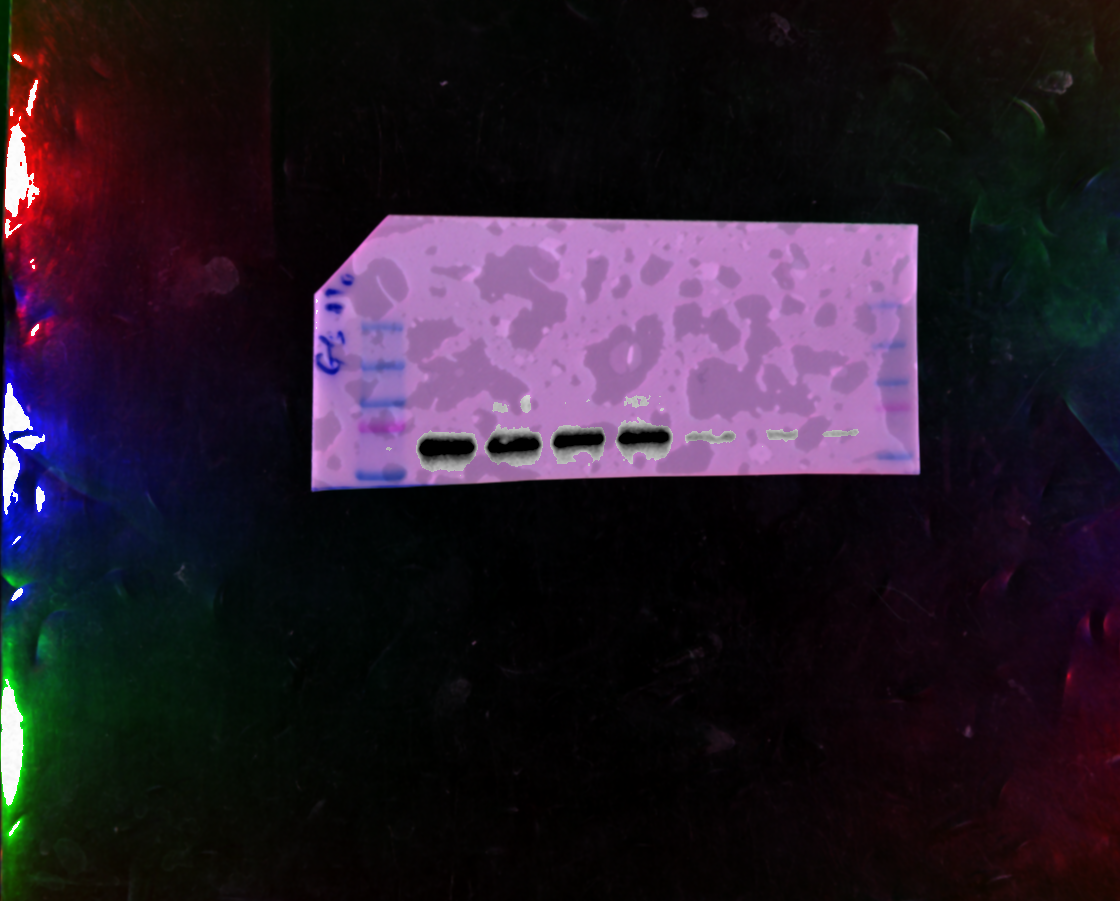

Supplement: Figure 3—source data 2. [file elife-100747-fig3-data2.zip › Figure 3 - Source Data 2 (original western files)/gls/23.07.26_15.22.05+Marker_PUB_600.tif]

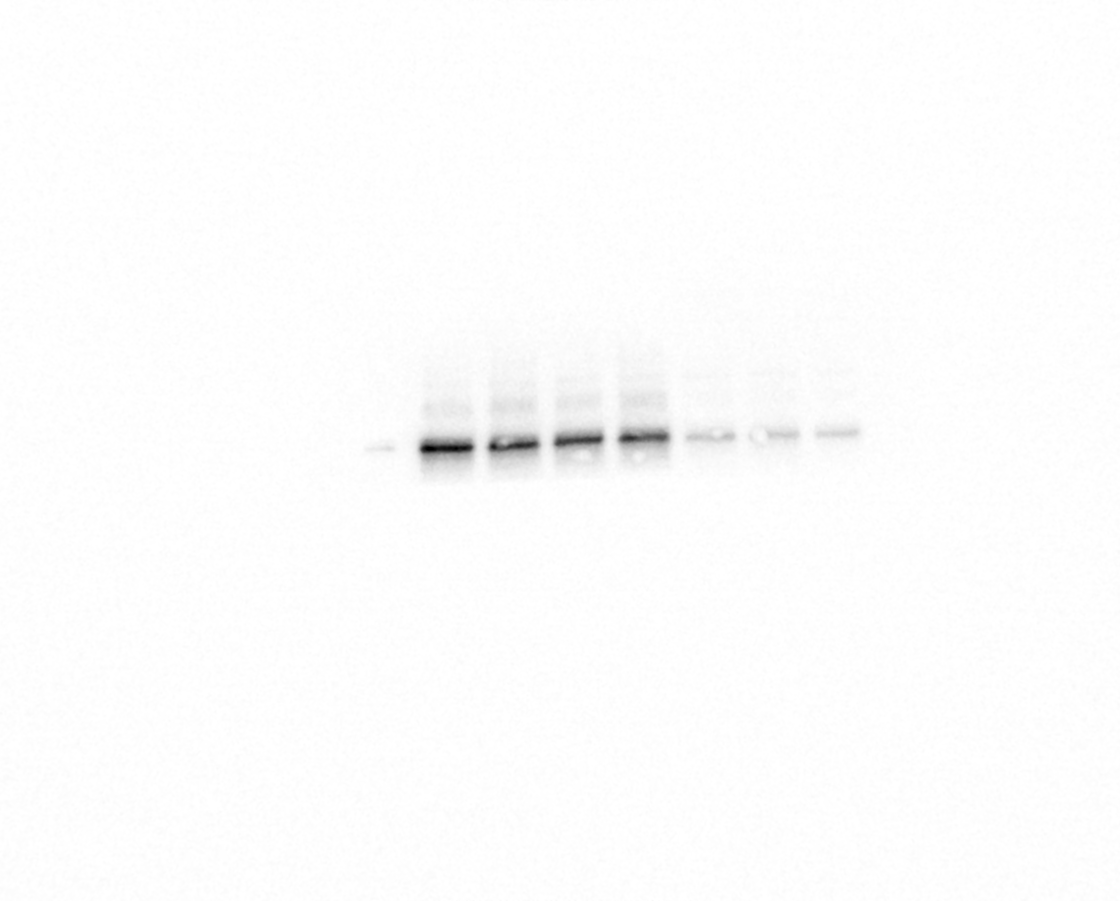

Supplement: Figure 3—source data 2. [file elife-100747-fig3-data2.zip › Figure 3 - Source Data 2 (original western files)/gls/23.07.26_15.22.05.tif]

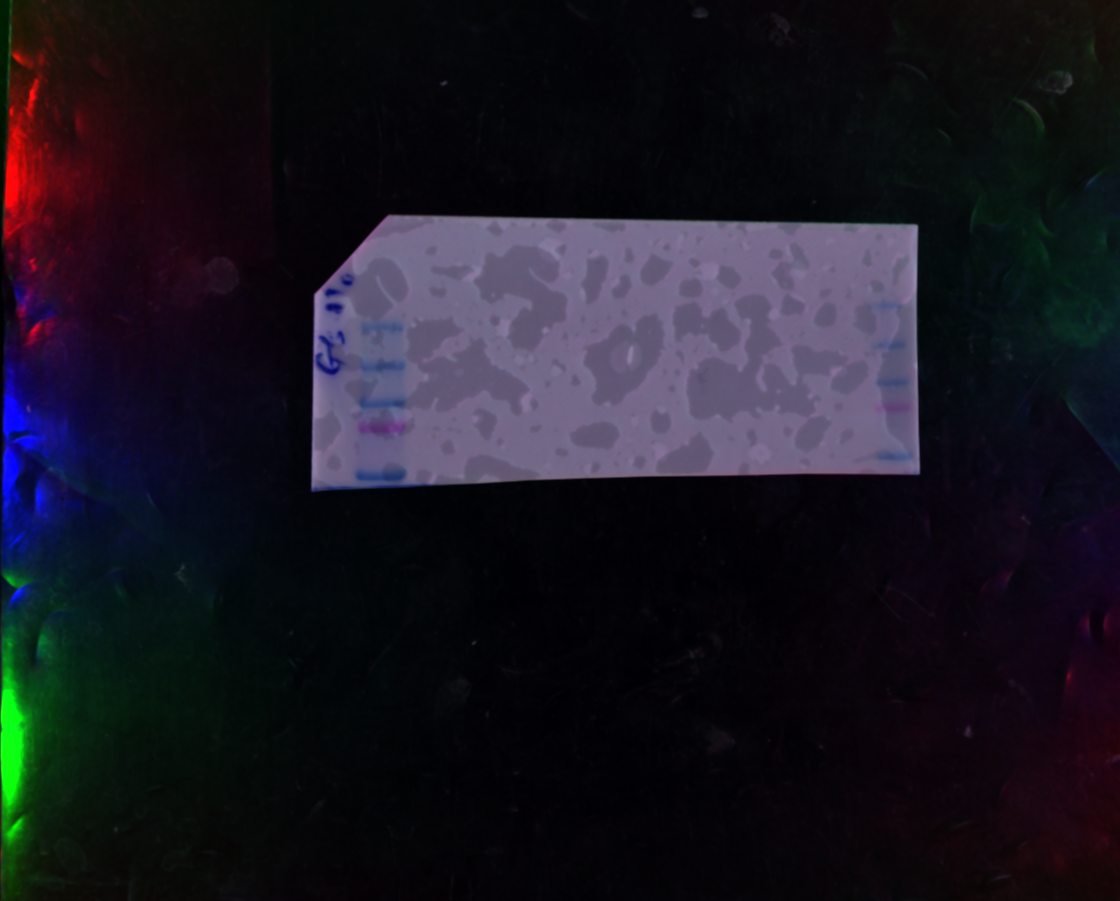

Supplement: Figure 3—source data 2. [file elife-100747-fig3-data2.zip › Figure 3 - Source Data 2 (original western files)/gls/23.07.26_15.22.05_marker.tif]

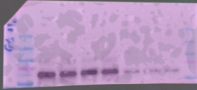

Supplement: Figure 3—source data 2. [file elife-100747-fig3-data2.zip › Figure 3 - Source Data 2 (original western files)/gls/23.07.26_15.22.05_marker_PUB_600.pdf]

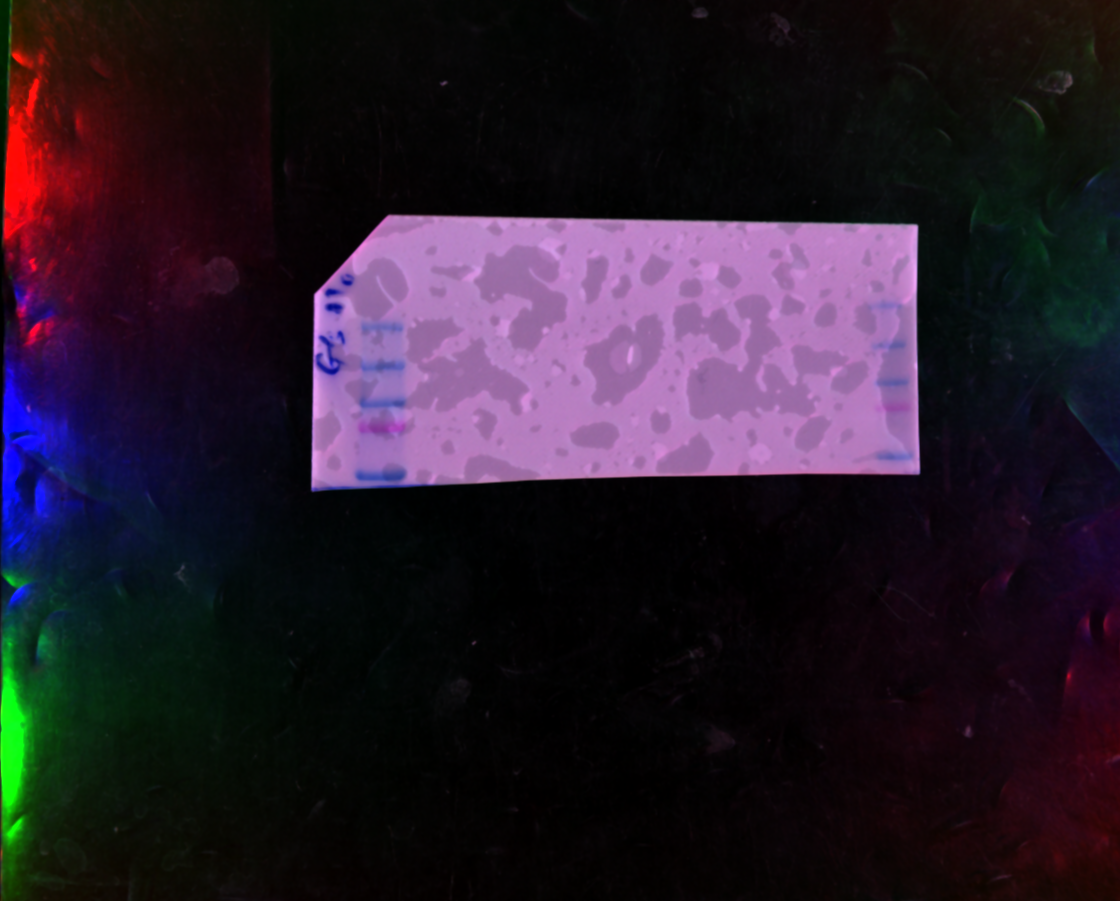

Supplement: Figure 3—source data 2. [file elife-100747-fig3-data2.zip › Figure 3 - Source Data 2 (original western files)/gls/23.07.26_15.22.05_marker_PUB_600.tif]

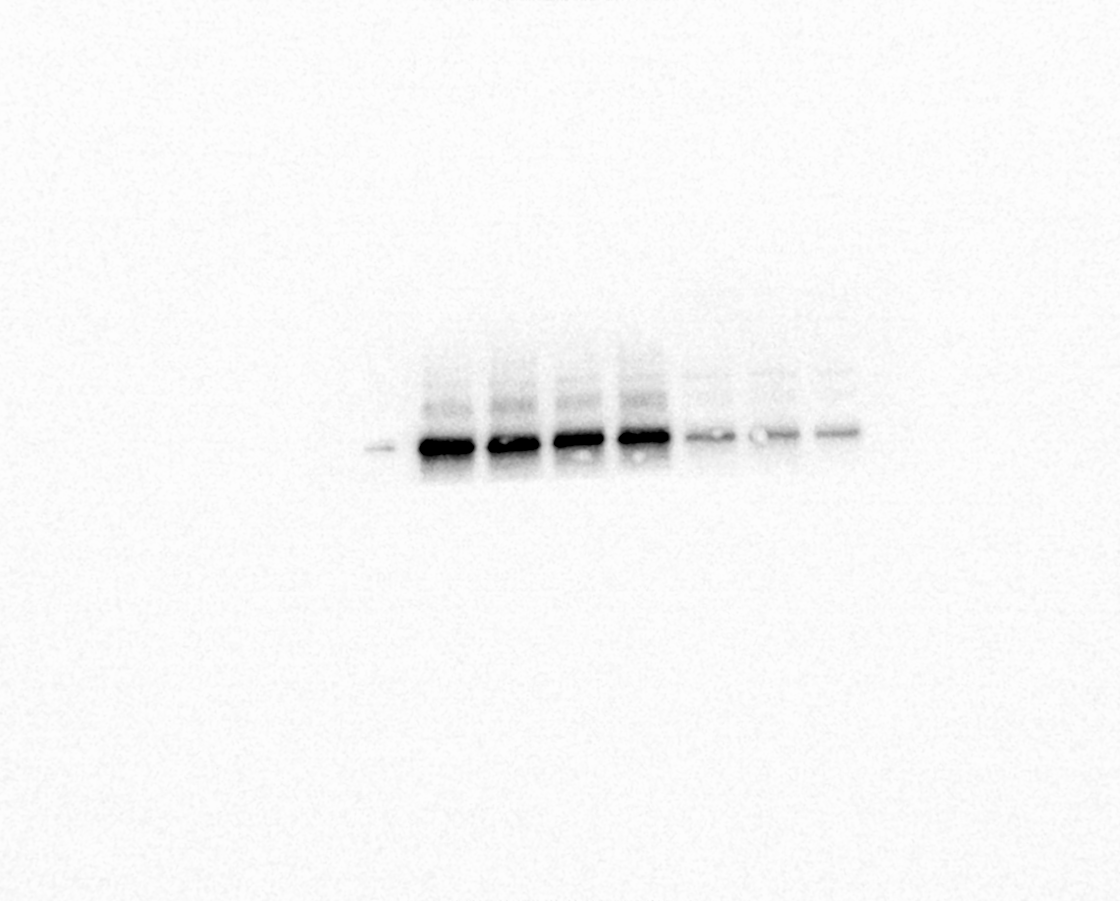

Supplement: Figure 3—source data 2. [file elife-100747-fig3-data2.zip › Figure 3 - Source Data 2 (original western files)/gls/23.07.26_15.22.05_PUB_600.tif]

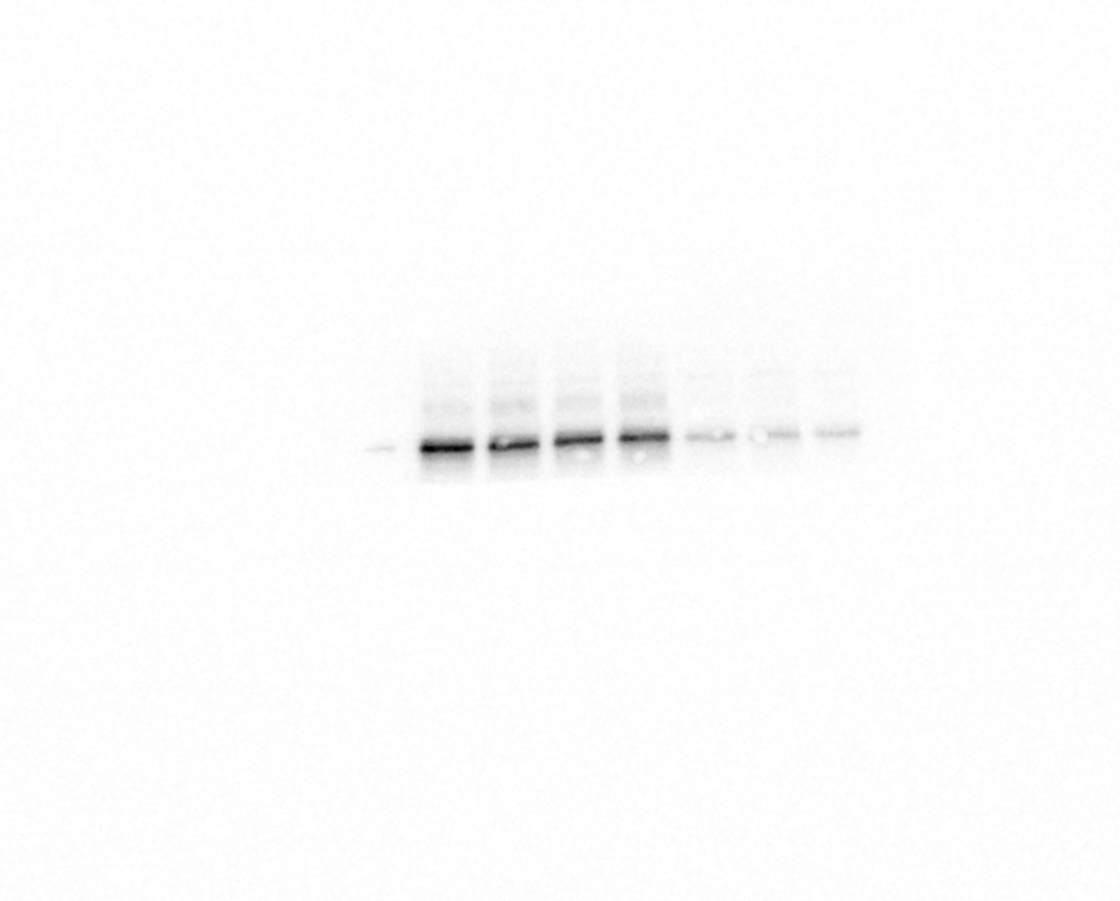

Supplement: Figure 3—source data 2. [file elife-100747-fig3-data2.zip › Figure 3 - Source Data 2 (original western files)/gls/23.07.26_15.23.10_S1_F01.tif]

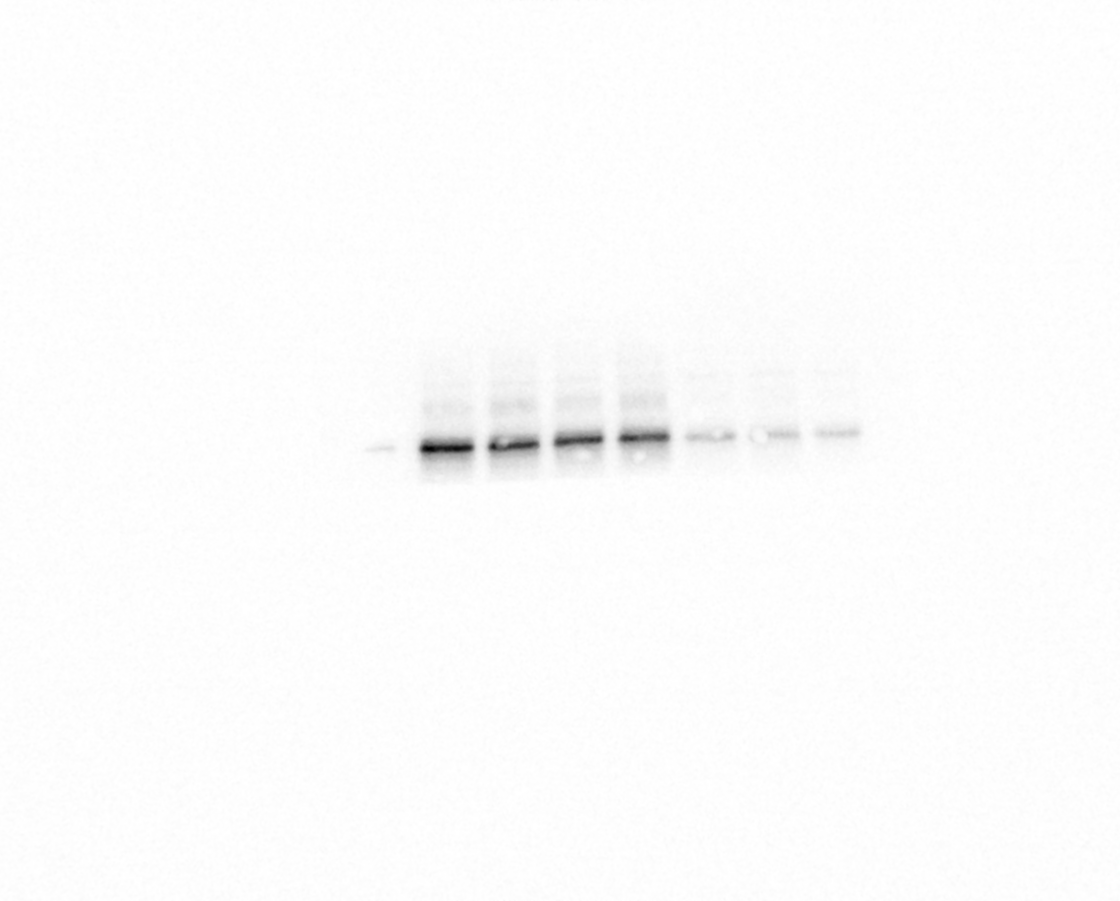

Supplement: Figure 3—source data 2. [file elife-100747-fig3-data2.zip › Figure 3 - Source Data 2 (original western files)/gls/23.07.26_15.23.10_S1_F01_PUB_600.tif]

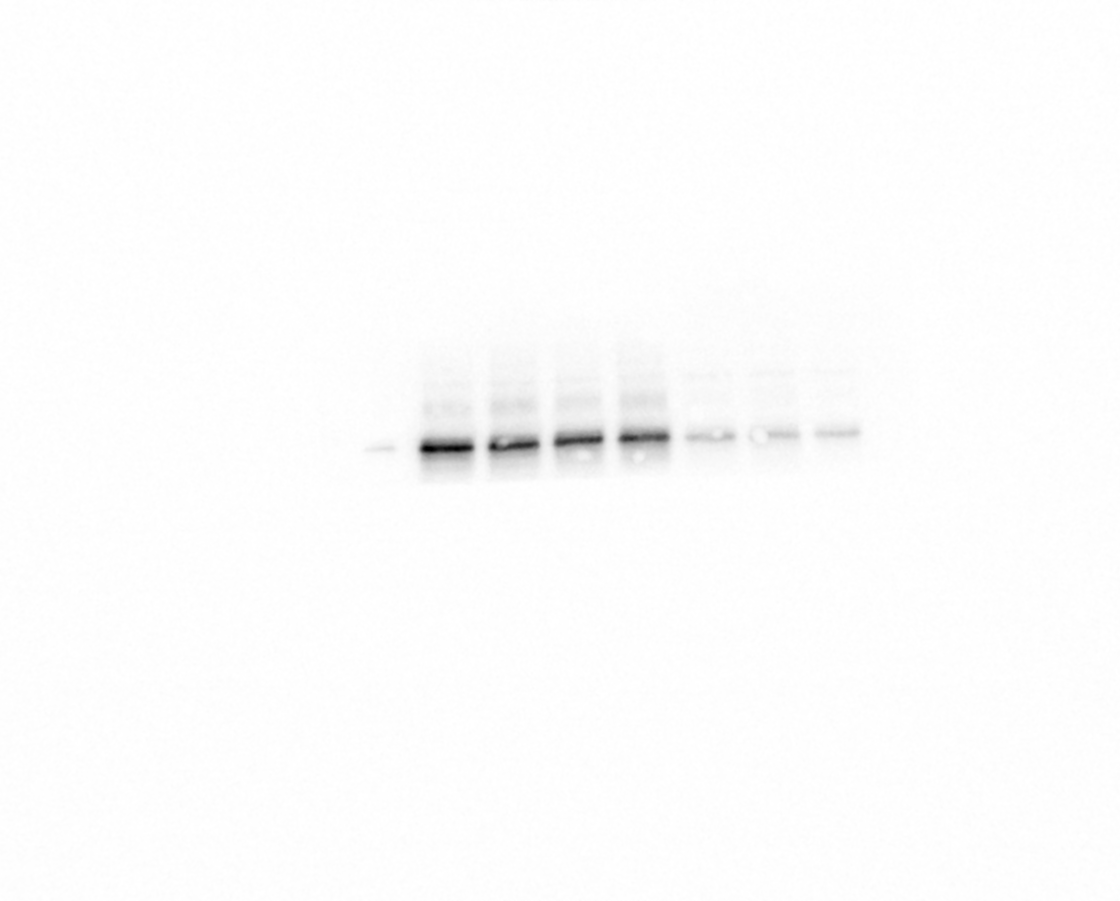

Supplement: Figure 3—source data 2. [file elife-100747-fig3-data2.zip › Figure 3 - Source Data 2 (original western files)/gls/23.07.26_15.23.10_S1_F02.tif]

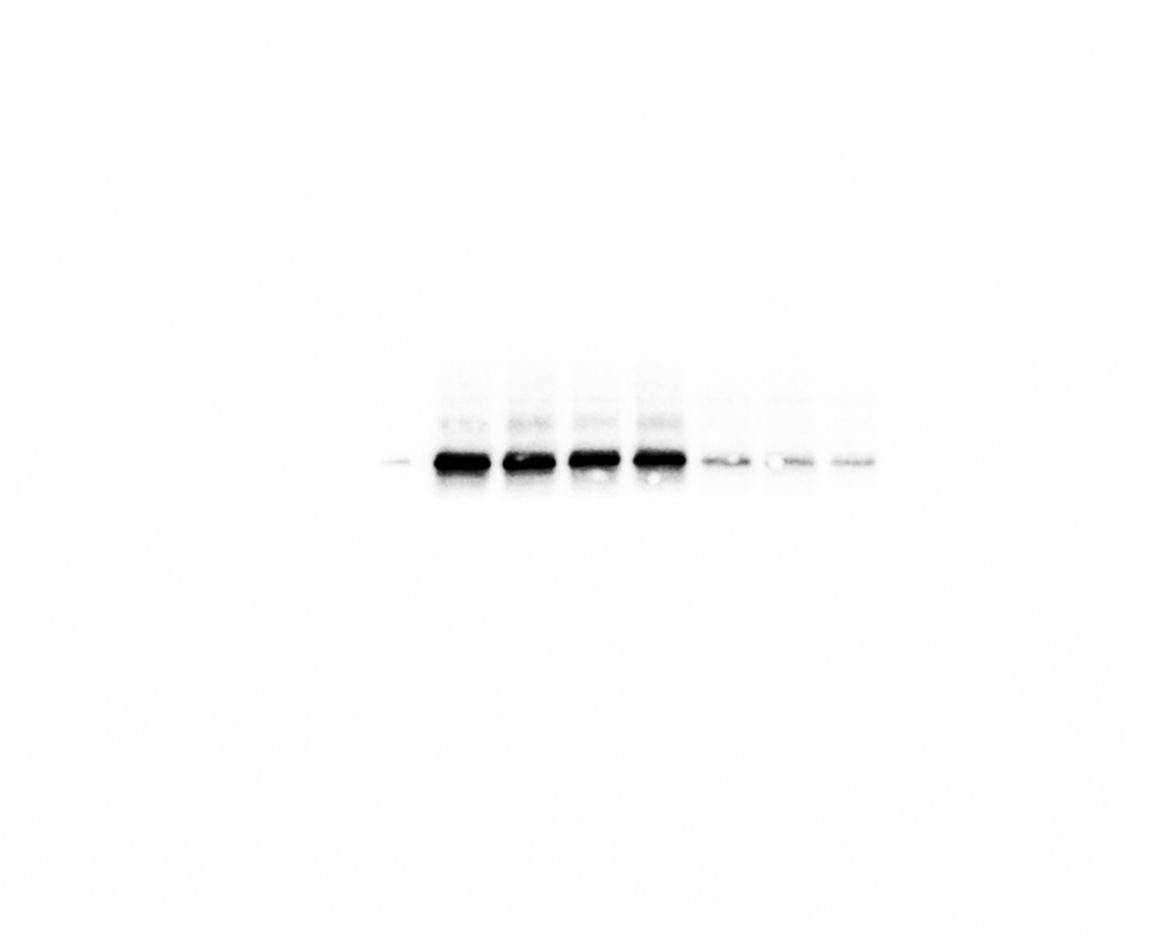

Supplement: Figure 3—source data 2. [file elife-100747-fig3-data2.zip › Figure 3 - Source Data 2 (original western files)/gls/23.07.26_15.23.10_S1_F02_PUB_600.tif]

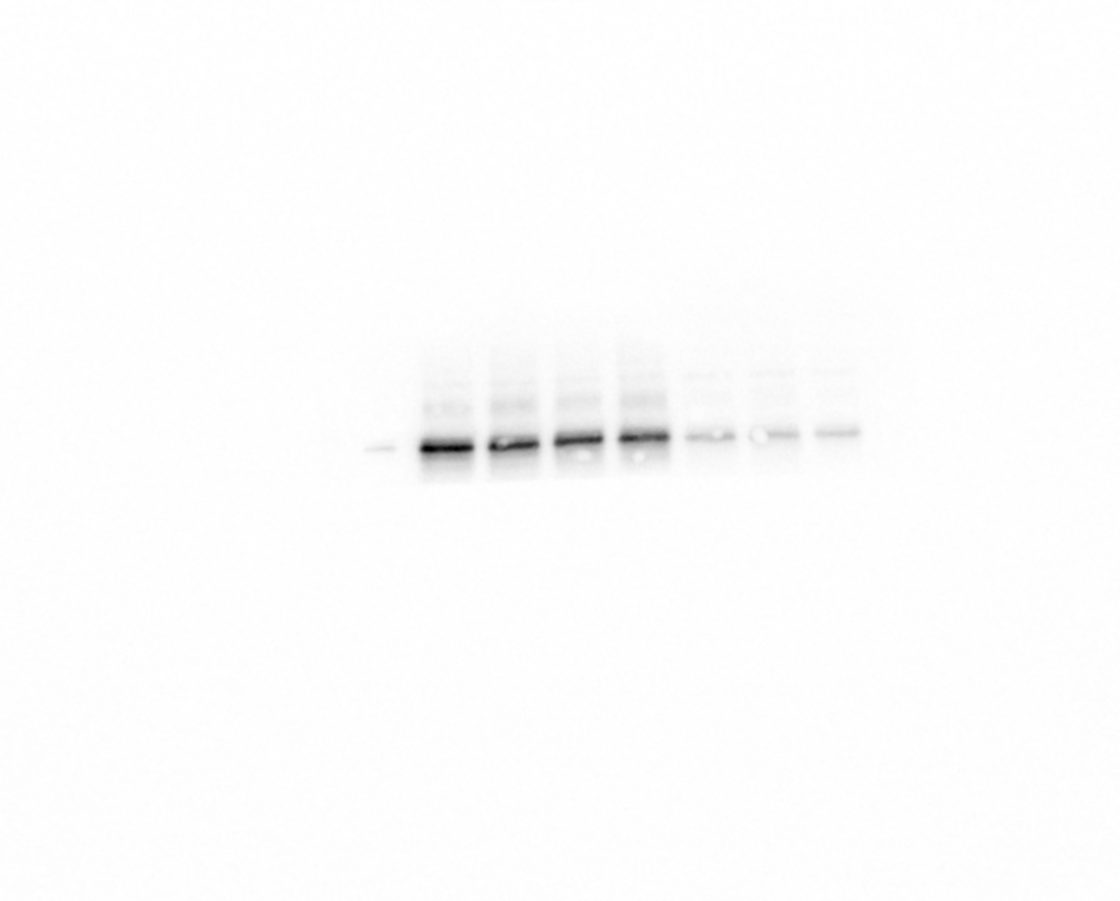

Supplement: Figure 3—source data 2. [file elife-100747-fig3-data2.zip › Figure 3 - Source Data 2 (original western files)/gls/23.07.26_15.23.10_S1_F03.tif]

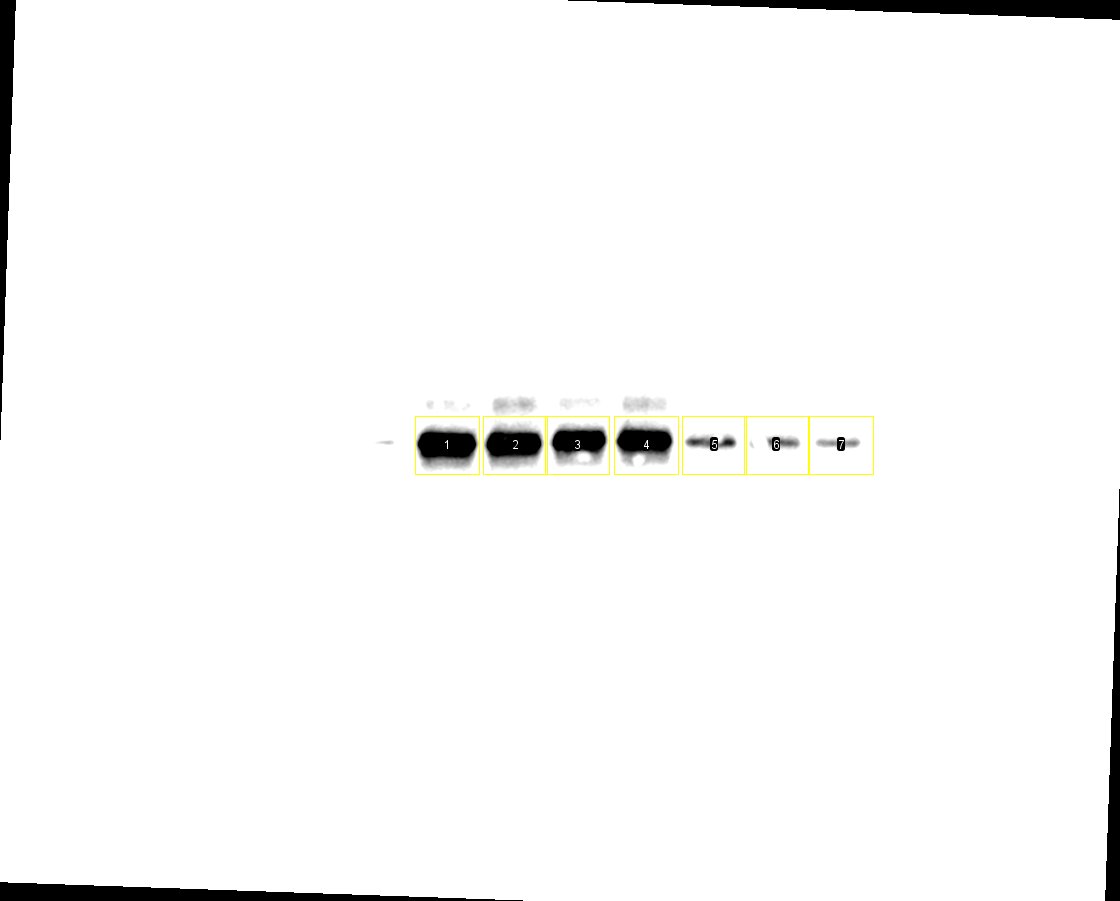

Supplement: Figure 3—source data 2. [file elife-100747-fig3-data2.zip › Figure 3 - Source Data 2 (original western files)/gls/23.07.26_15.23.10_S1_F03_PUB_600.jpg]

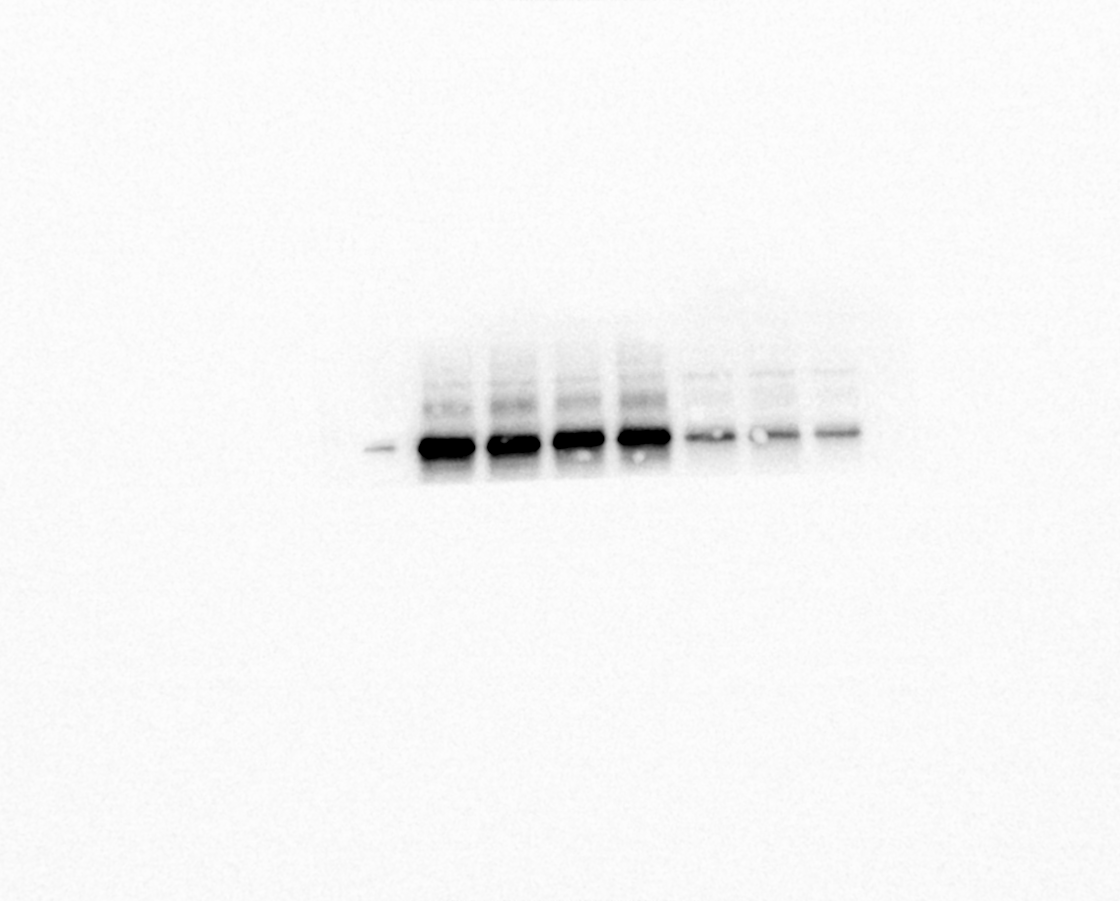

Supplement: Figure 3—source data 2. [file elife-100747-fig3-data2.zip › Figure 3 - Source Data 2 (original western files)/gls/23.07.26_15.23.10_S1_F03_PUB_600.tif]

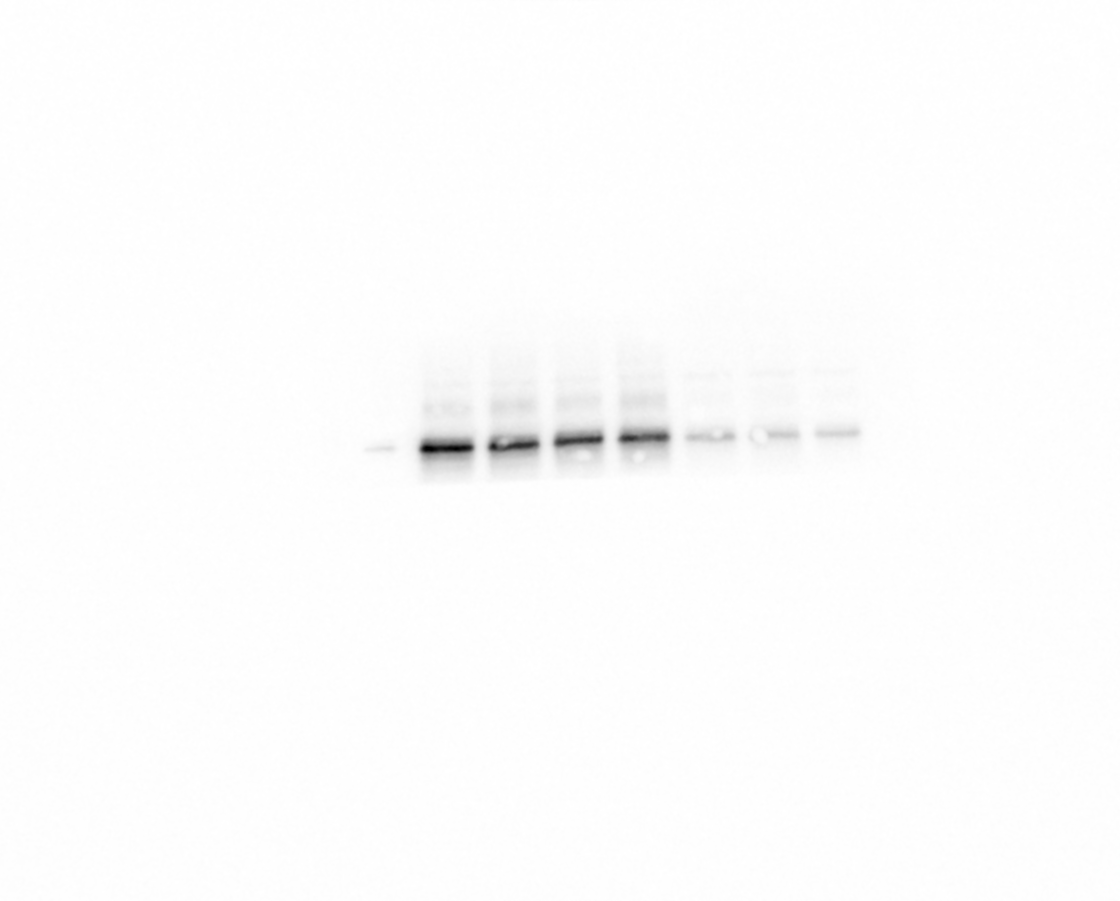

Supplement: Figure 3—source data 2. [file elife-100747-fig3-data2.zip › Figure 3 - Source Data 2 (original western files)/gls/23.07.26_15.23.10_S1_F04.tif]

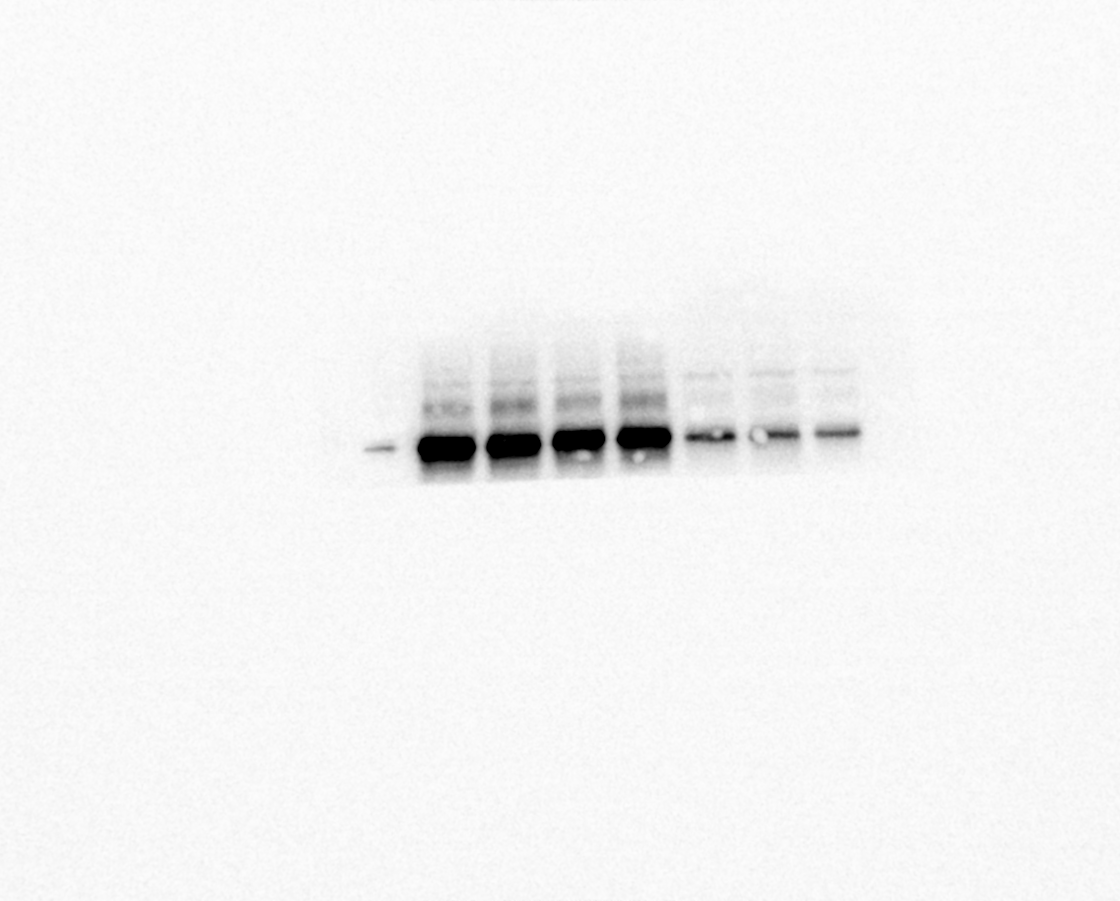

Supplement: Figure 3—source data 2. [file elife-100747-fig3-data2.zip › Figure 3 - Source Data 2 (original western files)/gls/23.07.26_15.23.10_S1_F04_PUB_600.tif]

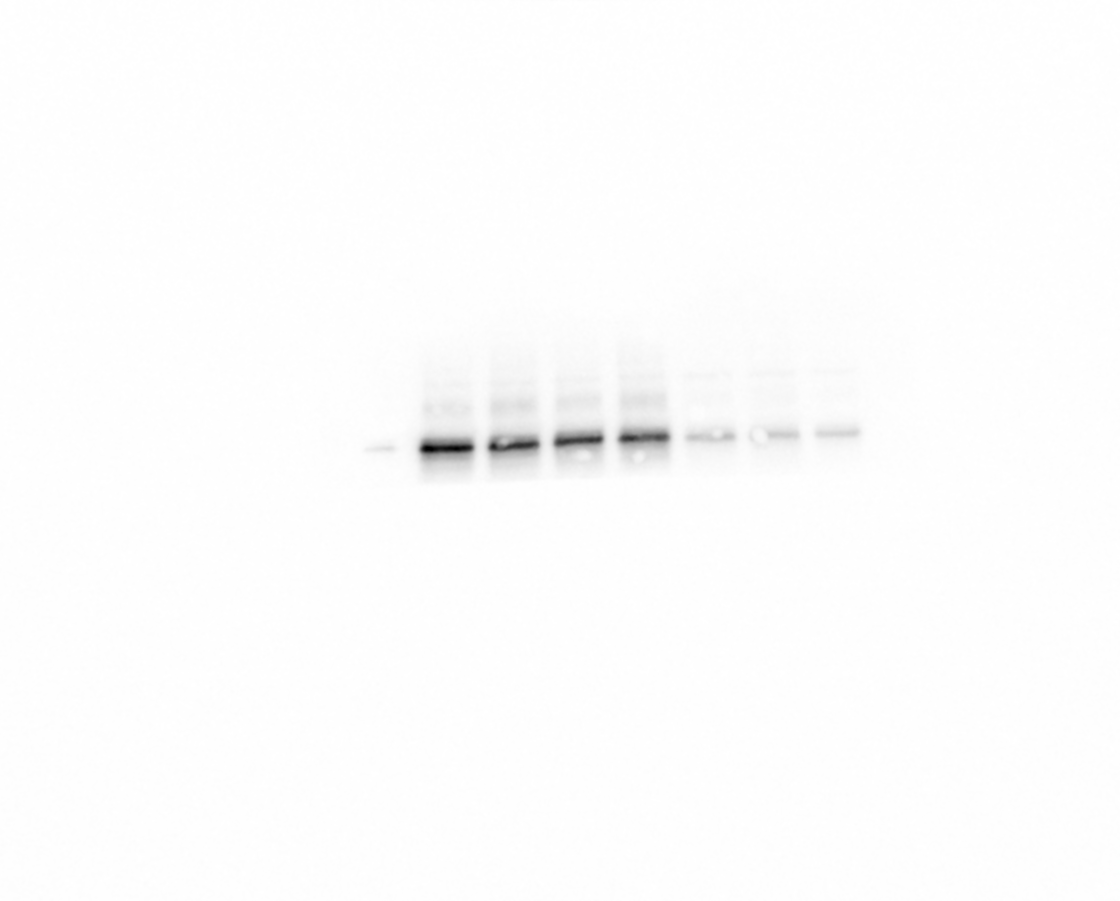

Supplement: Figure 3—source data 2. [file elife-100747-fig3-data2.zip › Figure 3 - Source Data 2 (original western files)/gls/23.07.26_15.23.10_S1_F05.tif]

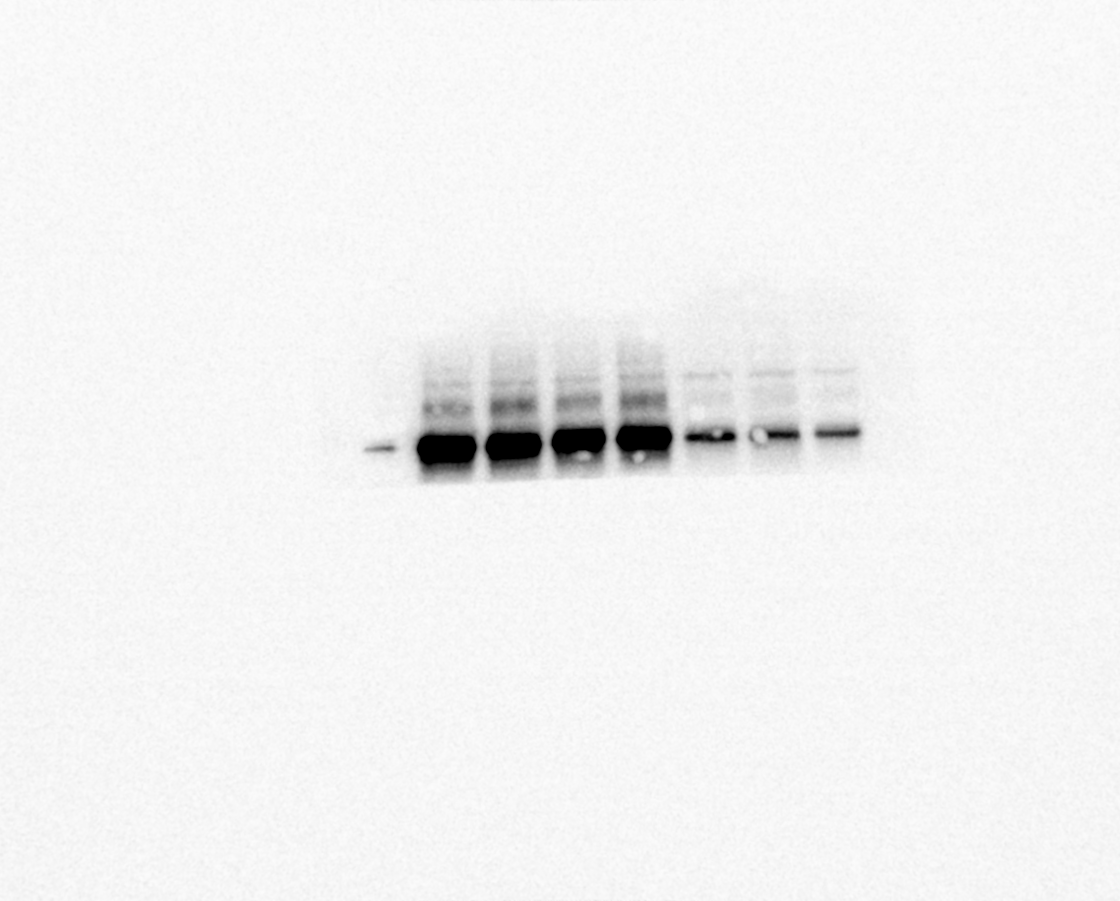

Supplement: Figure 3—source data 2. [file elife-100747-fig3-data2.zip › Figure 3 - Source Data 2 (original western files)/gls/23.07.26_15.23.10_S1_F05_PUB_600.tif]

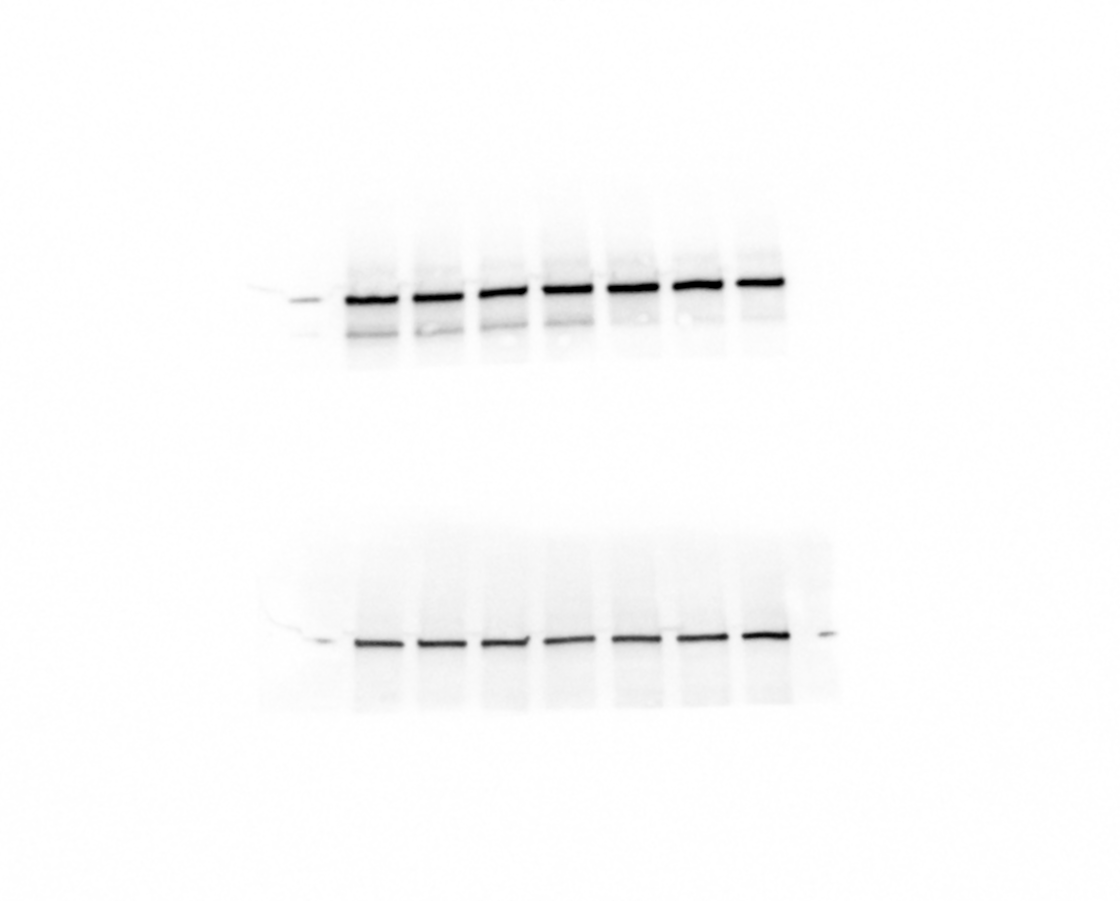

Supplement: Figure 3—source data 2. [file elife-100747-fig3-data2.zip › Figure 3 - Source Data 2 (original western files)/hsp90/23.07.27_17.20.06.tif]

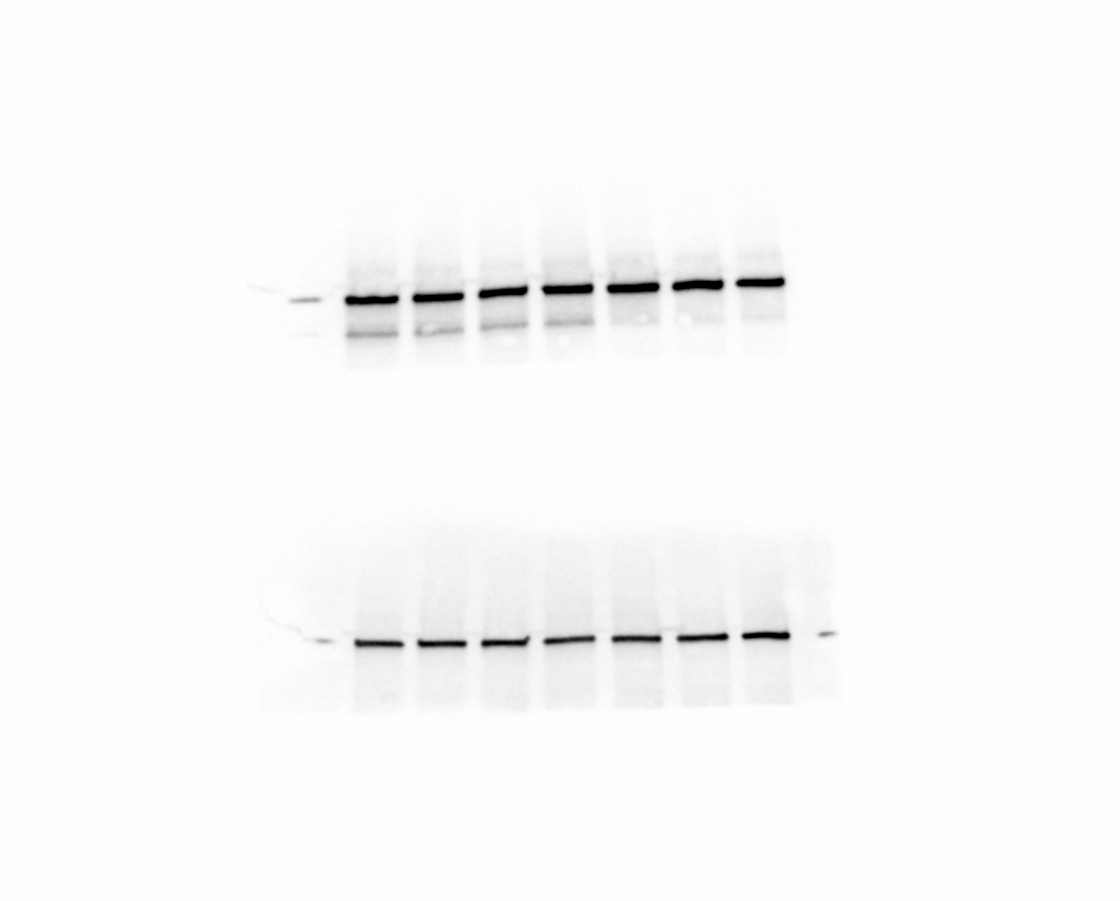

Supplement: Figure 3—source data 2. [file elife-100747-fig3-data2.zip › Figure 3 - Source Data 2 (original western files)/hsp90/23.07.27_17.20.06_PUB_600.tif]

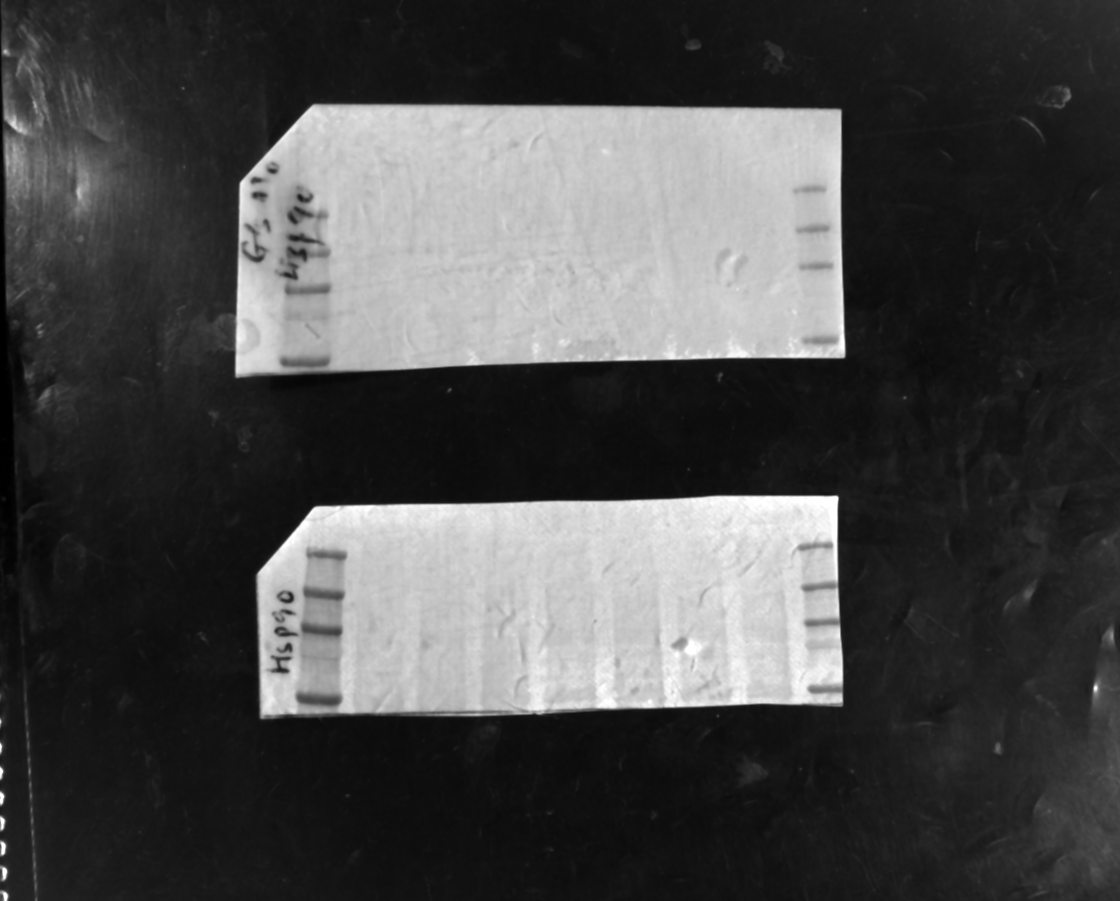

Supplement: Figure 3—source data 2. [file elife-100747-fig3-data2.zip › Figure 3 - Source Data 2 (original western files)/hsp90/23.07.27_17.20.42+Marker.tif]

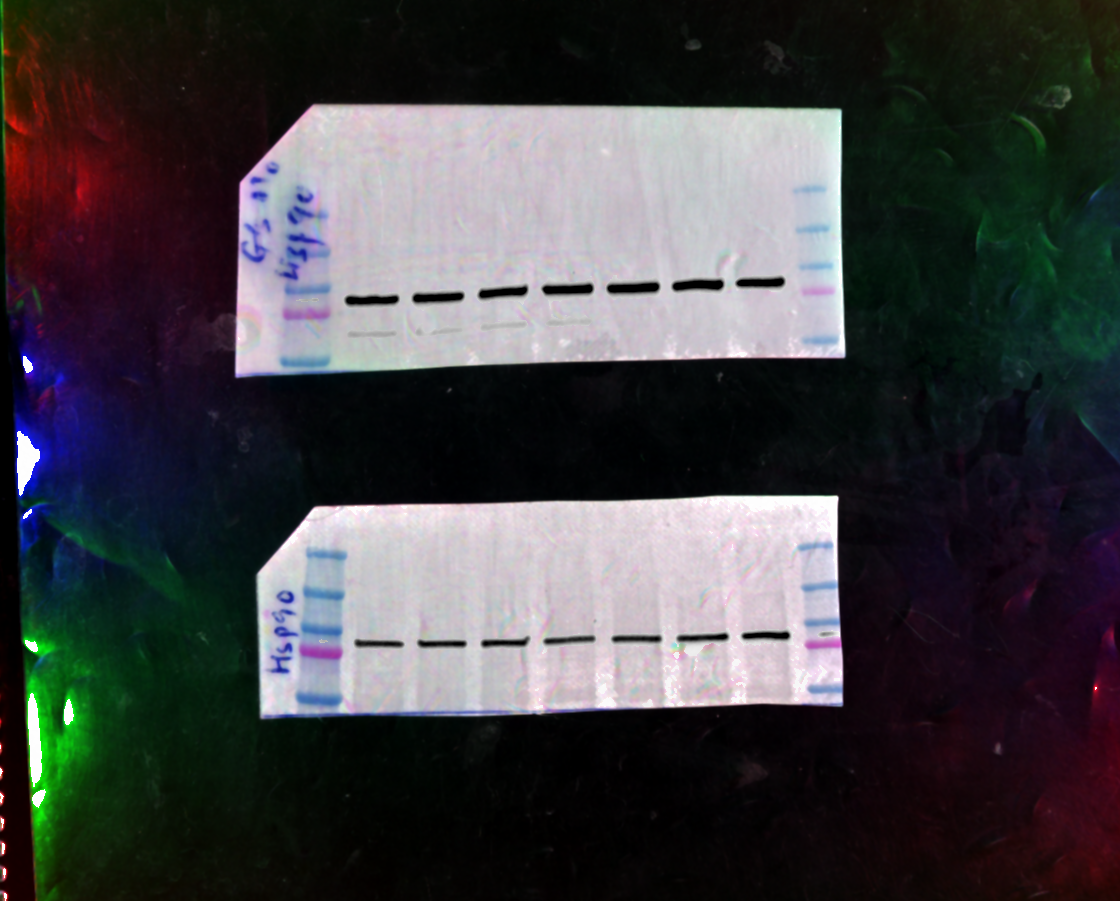

Supplement: Figure 3—source data 2. [file elife-100747-fig3-data2.zip › Figure 3 - Source Data 2 (original western files)/hsp90/23.07.27_17.20.42+Marker_PUB_600.tif]

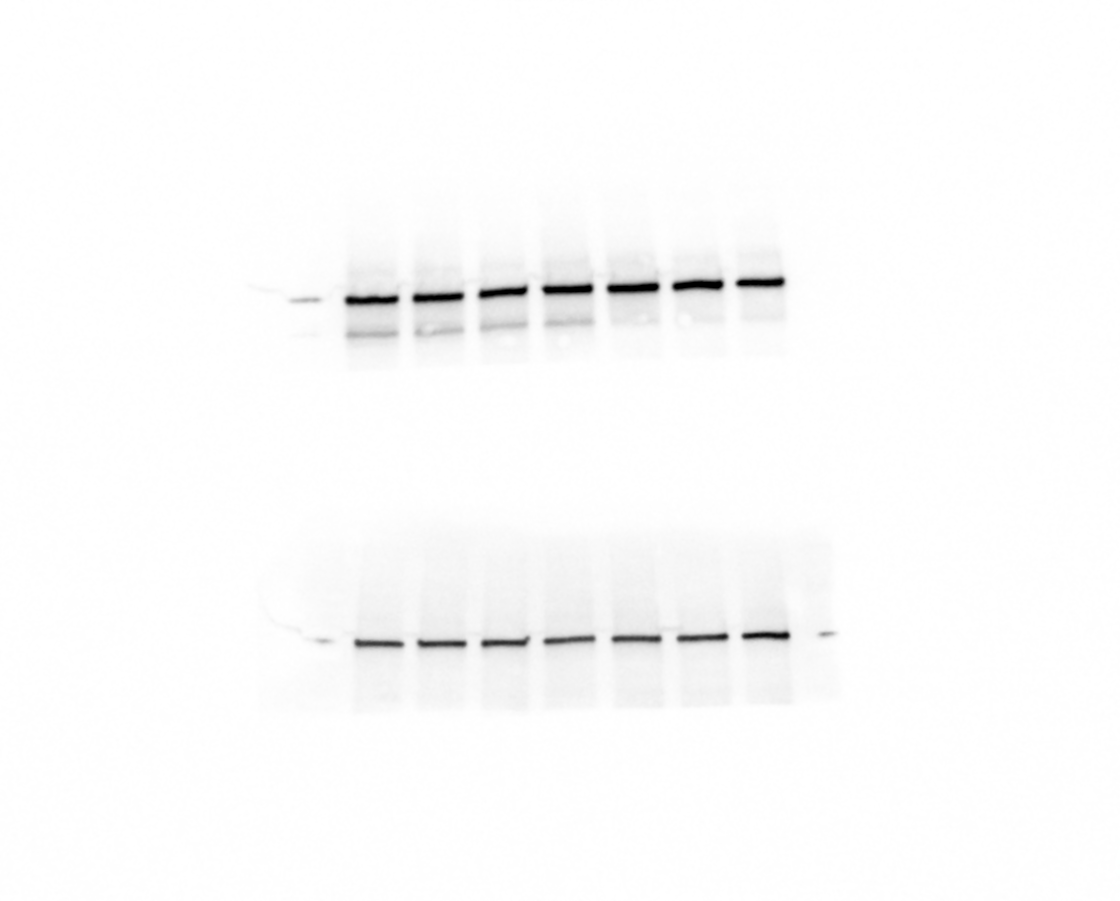

Supplement: Figure 3—source data 2. [file elife-100747-fig3-data2.zip › Figure 3 - Source Data 2 (original western files)/hsp90/23.07.27_17.20.42.tif]

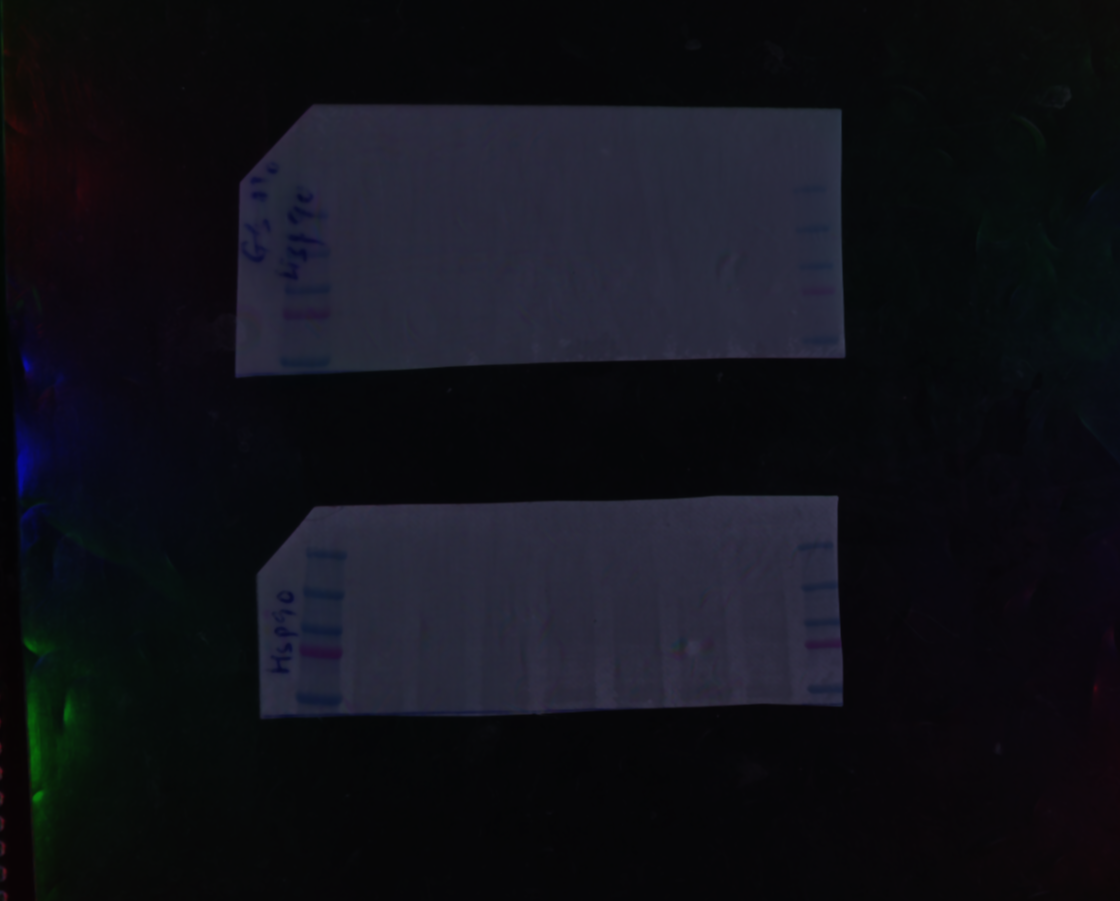

Supplement: Figure 3—source data 2. [file elife-100747-fig3-data2.zip › Figure 3 - Source Data 2 (original western files)/hsp90/23.07.27_17.20.42_marker.tif]

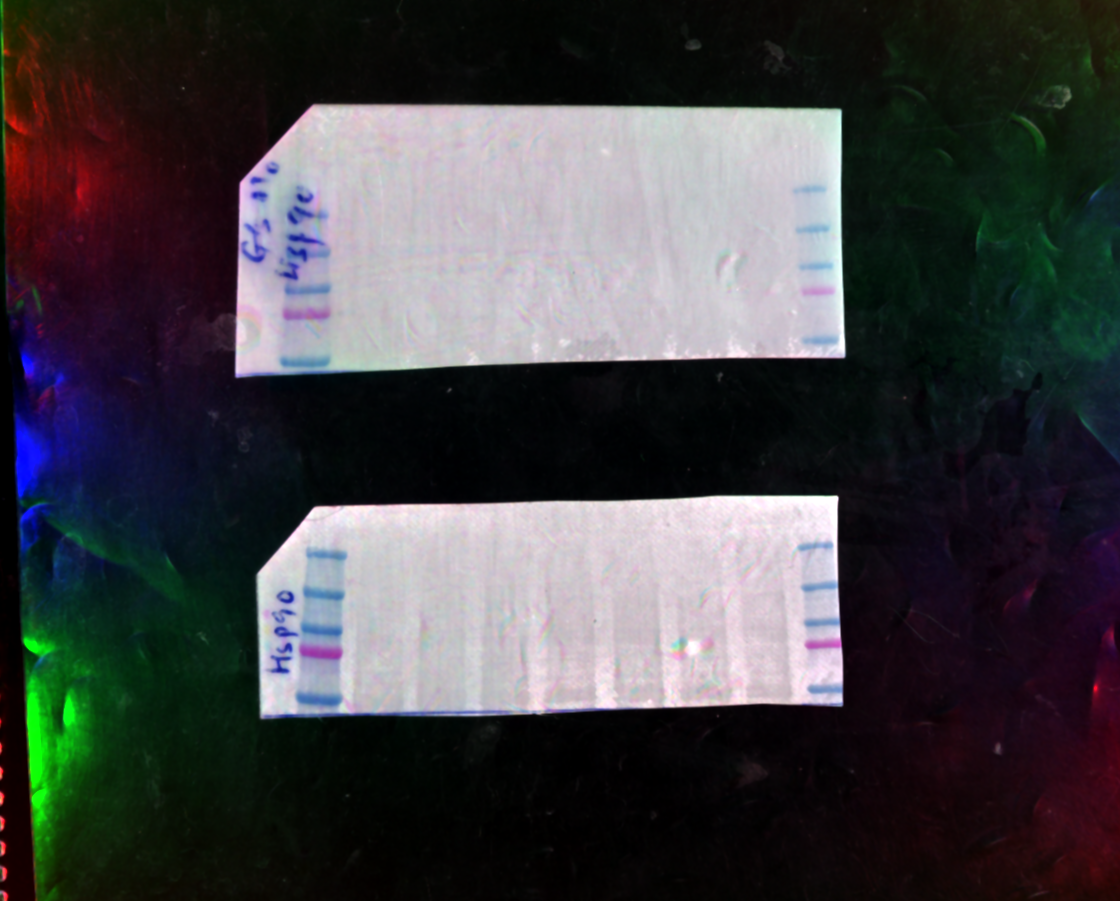

Supplement: Figure 3—source data 2. [file elife-100747-fig3-data2.zip › Figure 3 - Source Data 2 (original western files)/hsp90/23.07.27_17.20.42_marker_PUB_600.tif]

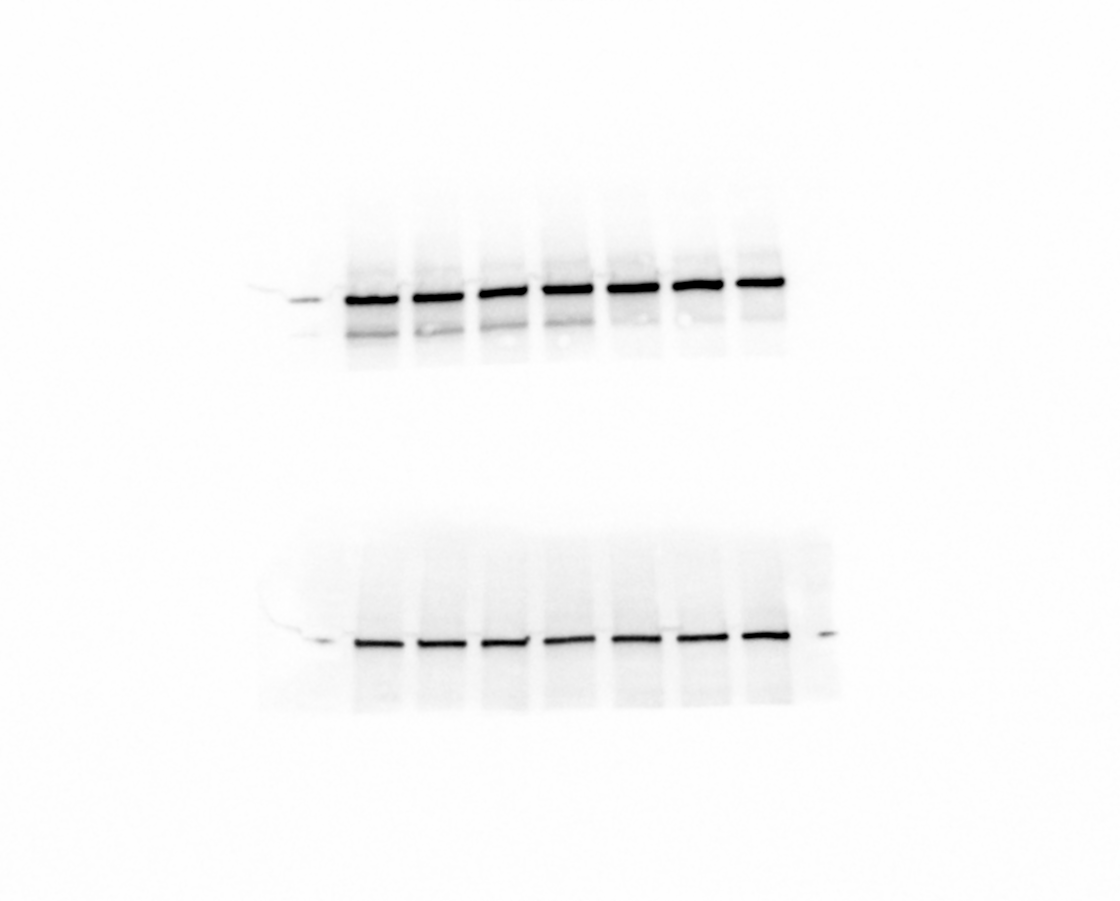

Supplement: Figure 3—source data 2. [file elife-100747-fig3-data2.zip › Figure 3 - Source Data 2 (original western files)/hsp90/23.07.27_17.20.42_PUB_600.tif]

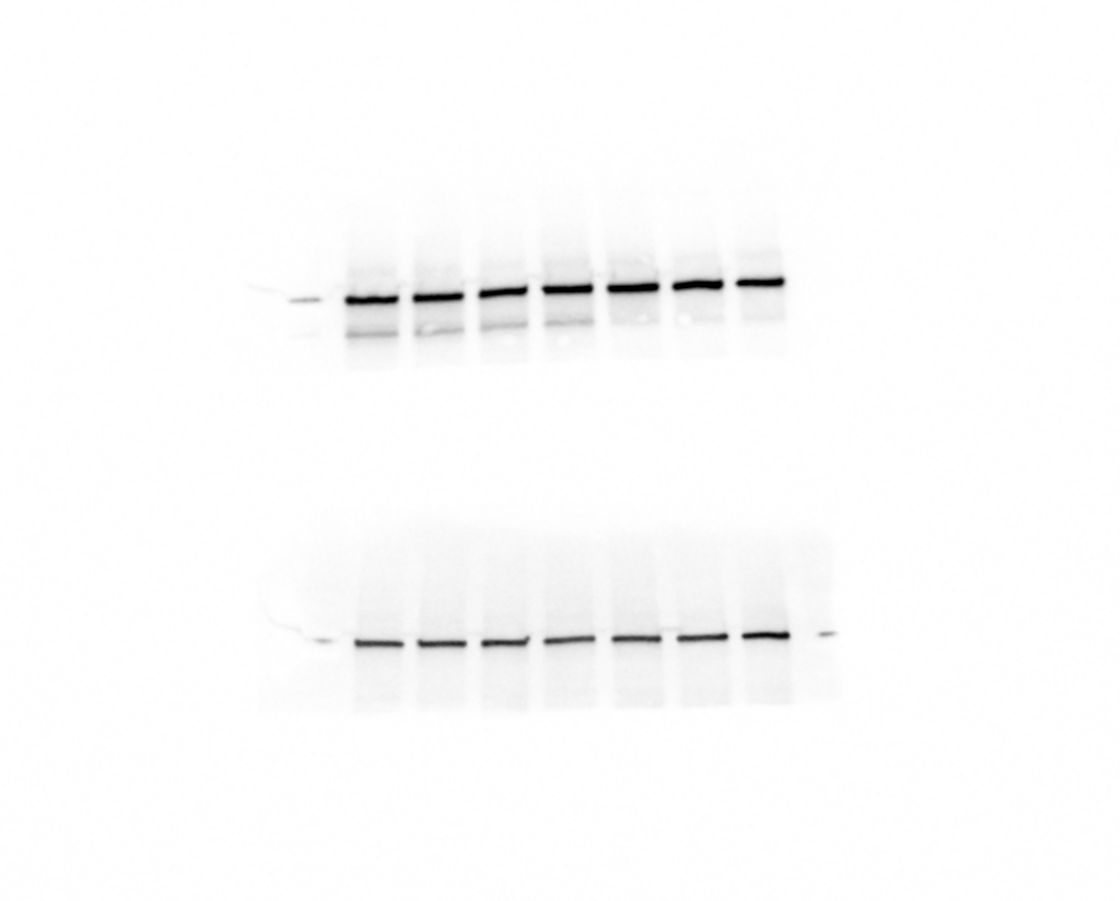

Supplement: Figure 3—source data 2. [file elife-100747-fig3-data2.zip › Figure 3 - Source Data 2 (original western files)/hsp90/23.07.27_17.21.11_S1_F01.tif]

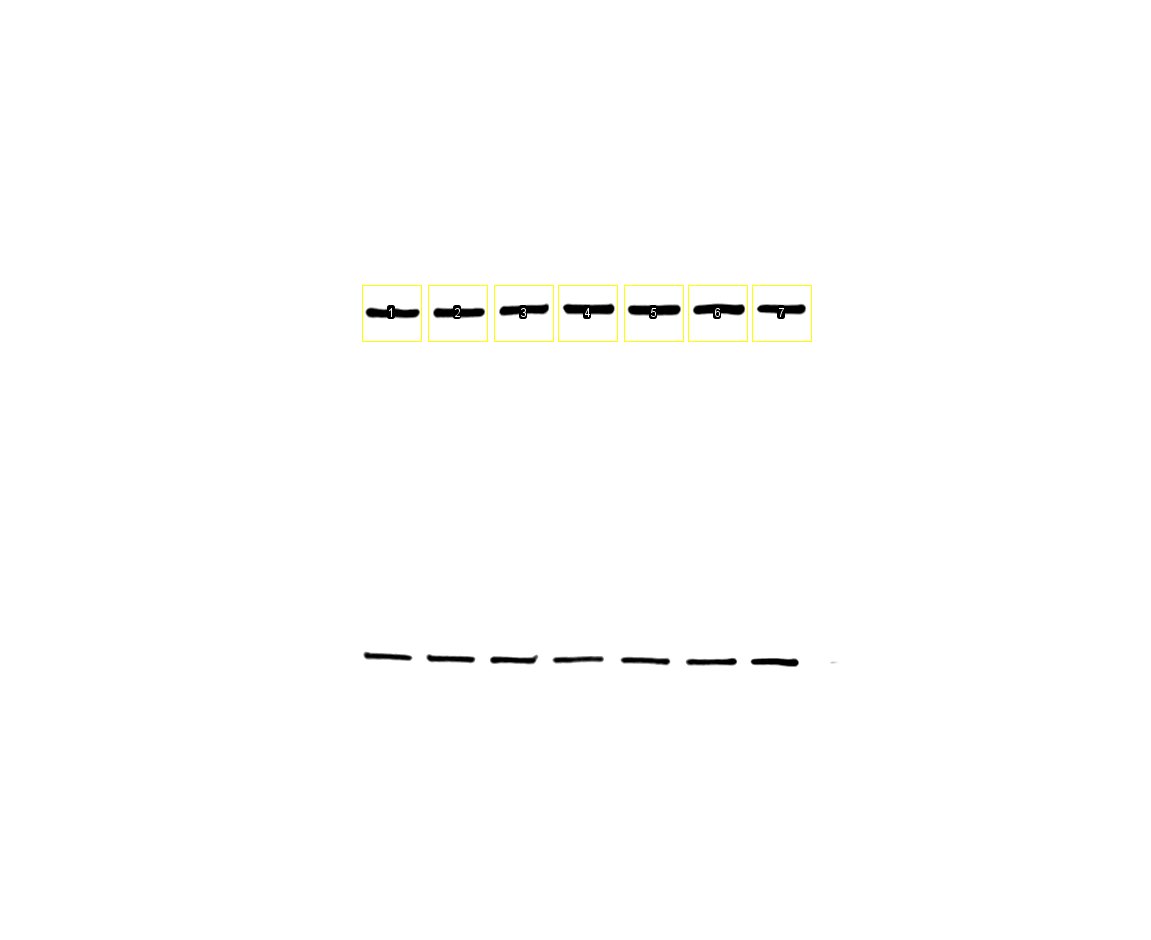

Supplement: Figure 3—source data 2. [file elife-100747-fig3-data2.zip › Figure 3 - Source Data 2 (original western files)/hsp90/23.07.27_17.21.11_S1_F01_PUB_600.jpg]

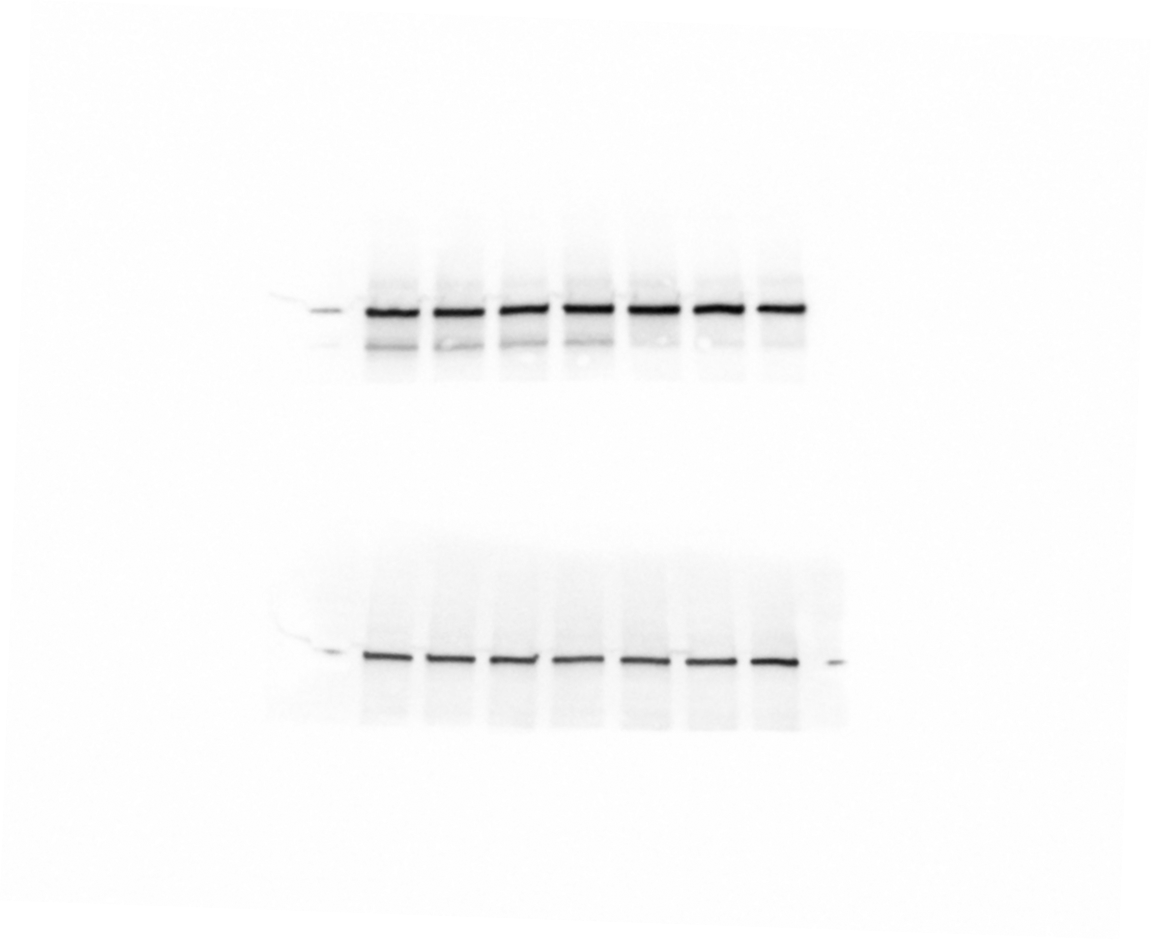

Supplement: Figure 3—source data 2. [file elife-100747-fig3-data2.zip › Figure 3 - Source Data 2 (original western files)/hsp90/23.07.27_17.21.11_S1_F01_PUB_600.tif]

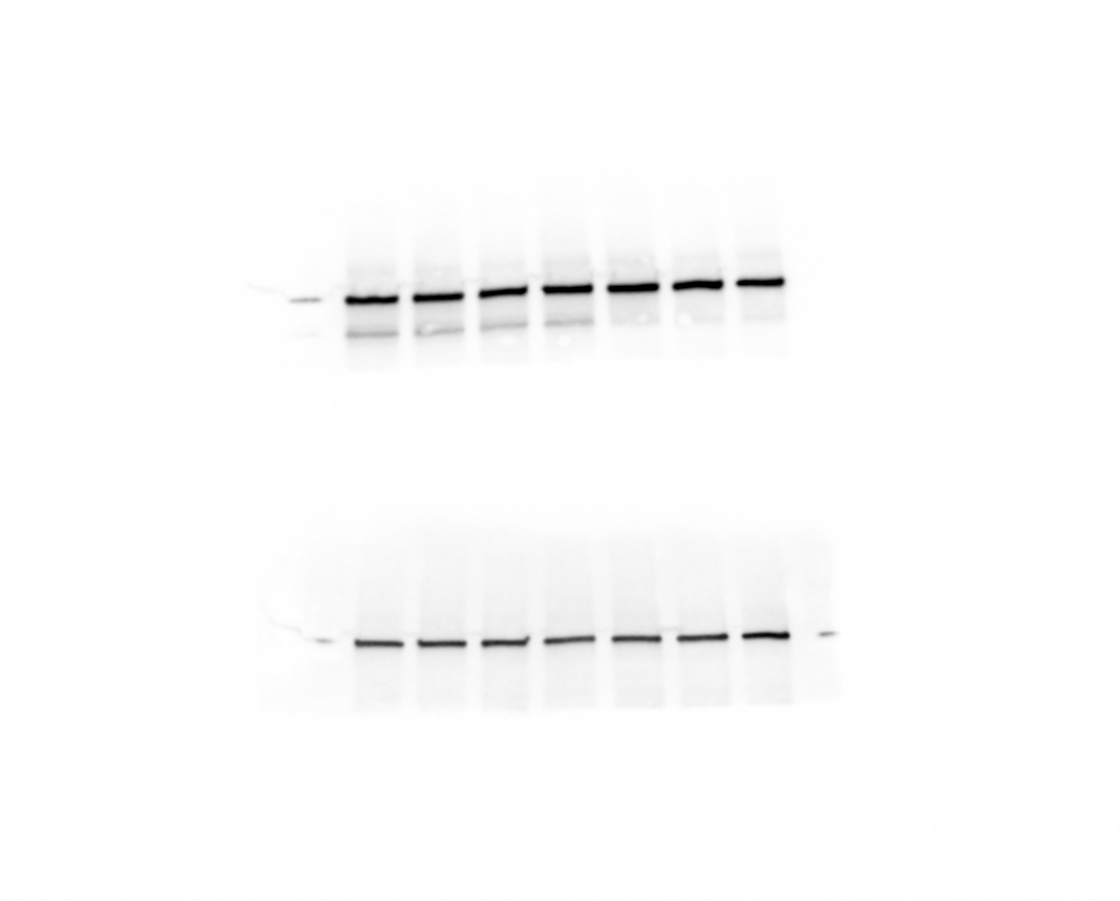

Supplement: Figure 3—source data 2. [file elife-100747-fig3-data2.zip › Figure 3 - Source Data 2 (original western files)/hsp90/23.07.27_17.21.11_S1_F02.tif]

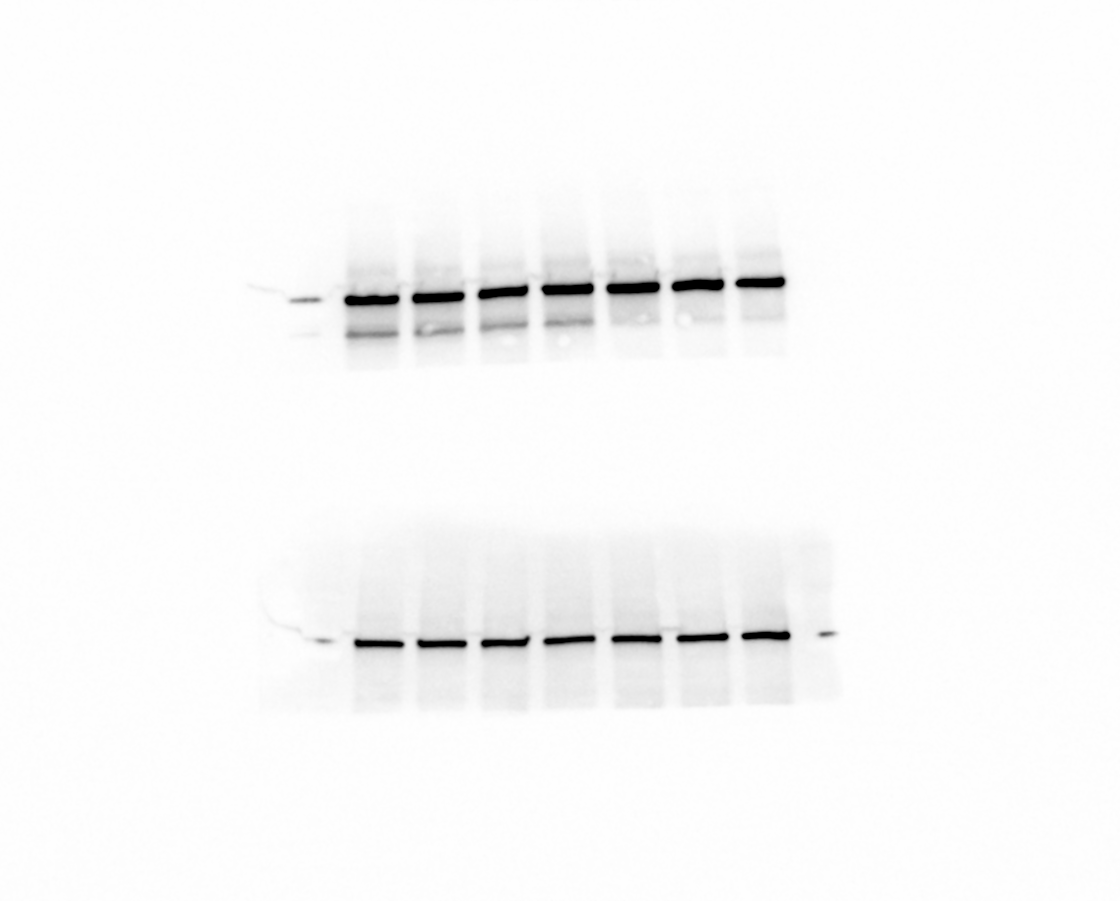

Supplement: Figure 3—source data 2. [file elife-100747-fig3-data2.zip › Figure 3 - Source Data 2 (original western files)/hsp90/23.07.27_17.21.11_S1_F02_PUB_600.tif]

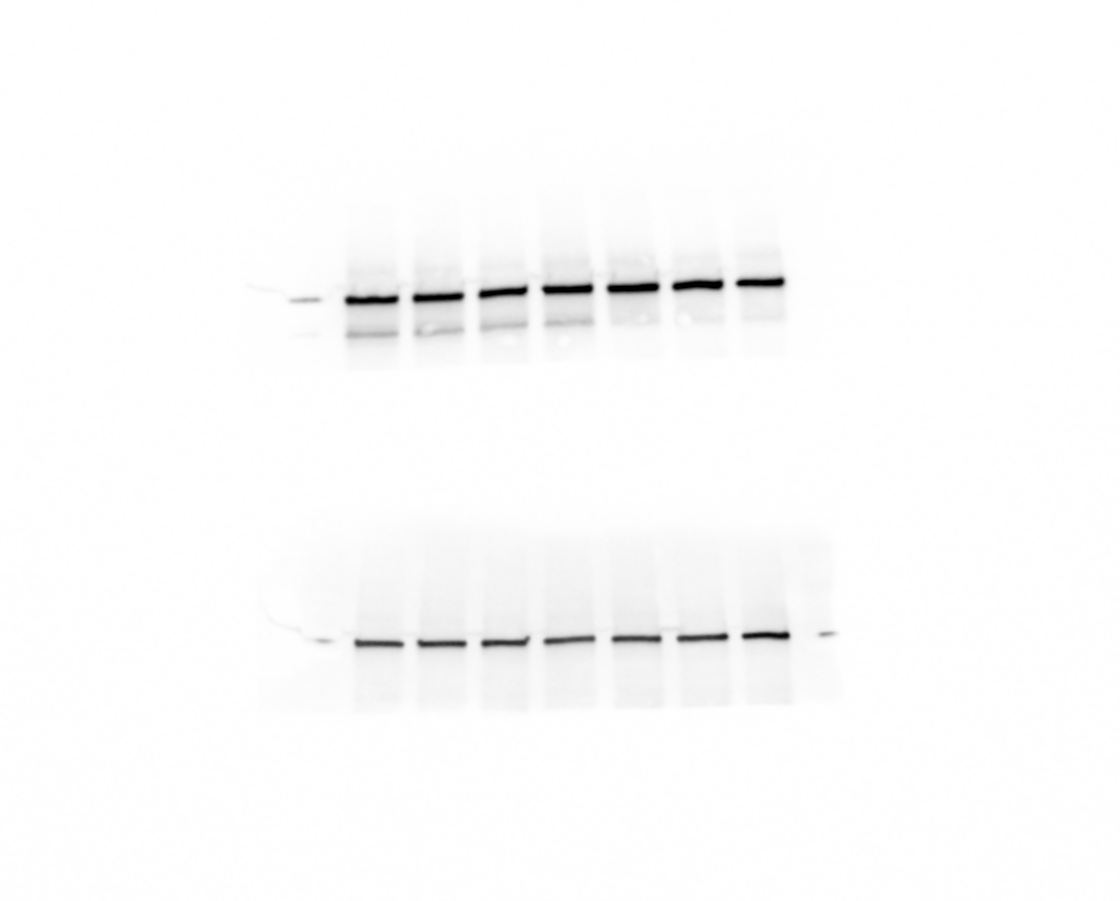

Supplement: Figure 3—source data 2. [file elife-100747-fig3-data2.zip › Figure 3 - Source Data 2 (original western files)/hsp90/23.07.27_17.21.11_S1_F03.tif]

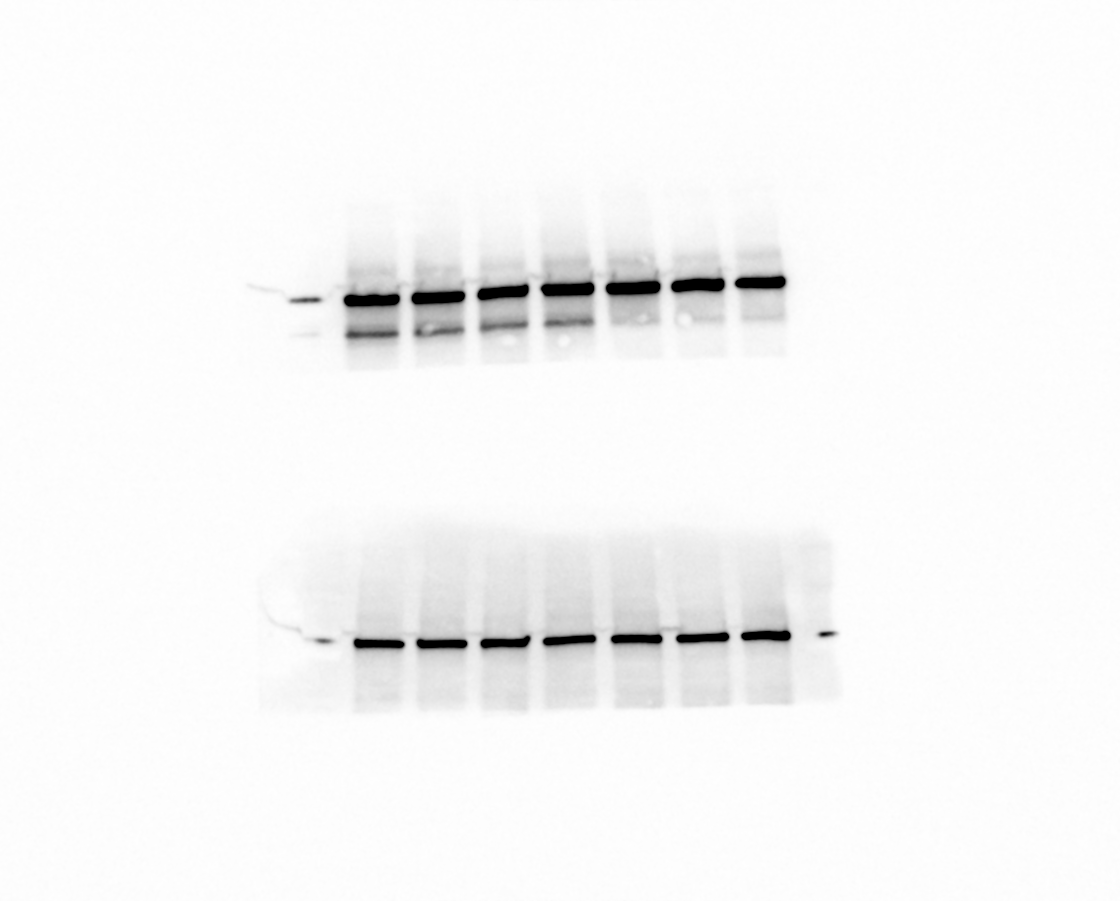

Supplement: Figure 3—source data 2. [file elife-100747-fig3-data2.zip › Figure 3 - Source Data 2 (original western files)/hsp90/23.07.27_17.21.11_S1_F03_PUB_600.tif]

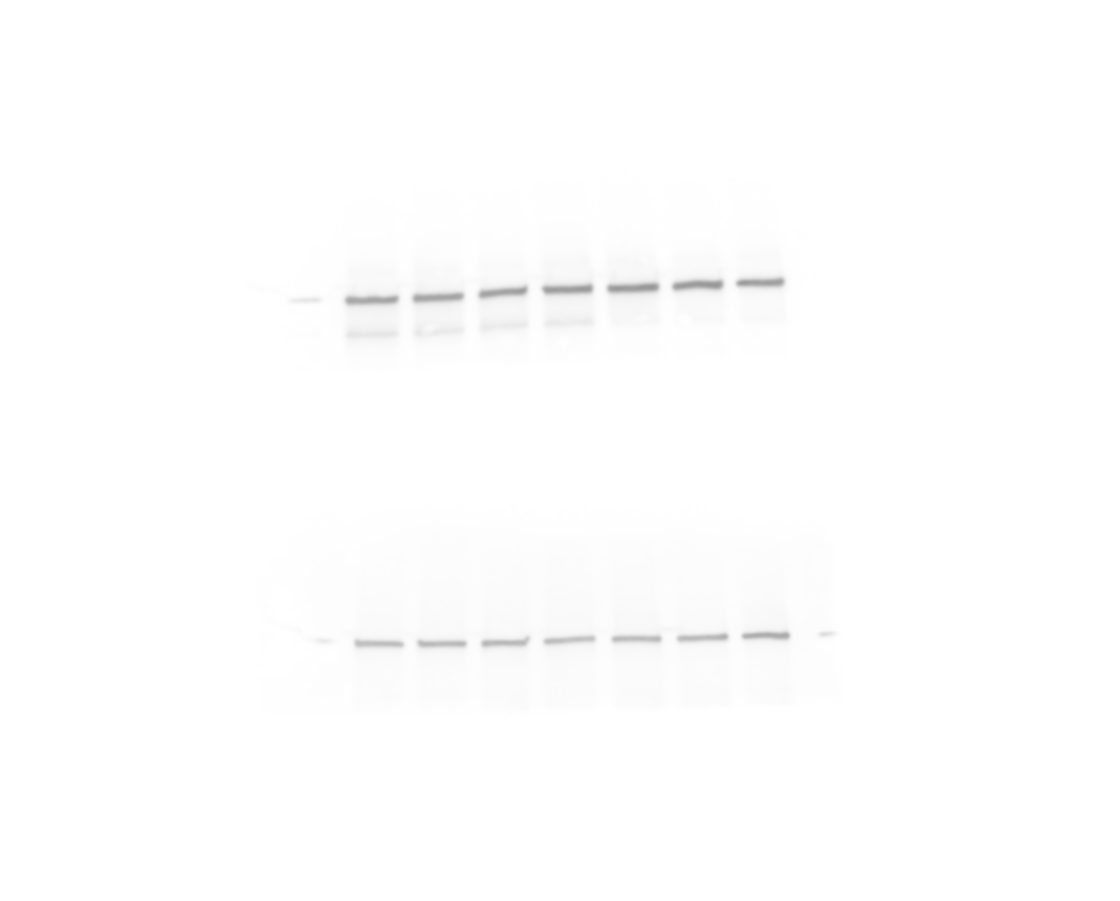

Supplement: Figure 3—source data 2. [file elife-100747-fig3-data2.zip › Figure 3 - Source Data 2 (original western files)/hsp90/23.07.27_17.21.11_S1_F04.tif]

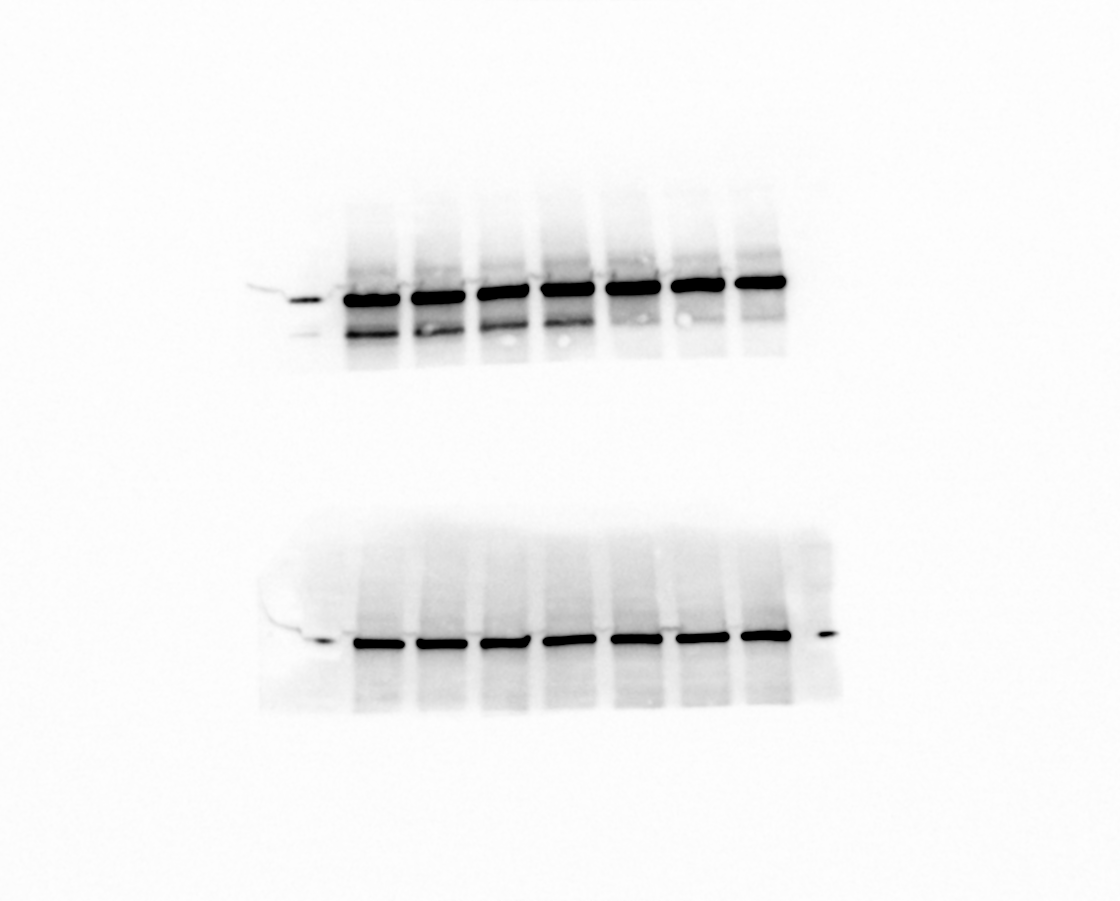

Supplement: Figure 3—source data 2. [file elife-100747-fig3-data2.zip › Figure 3 - Source Data 2 (original western files)/hsp90/23.07.27_17.21.11_S1_F04_PUB_600.tif]

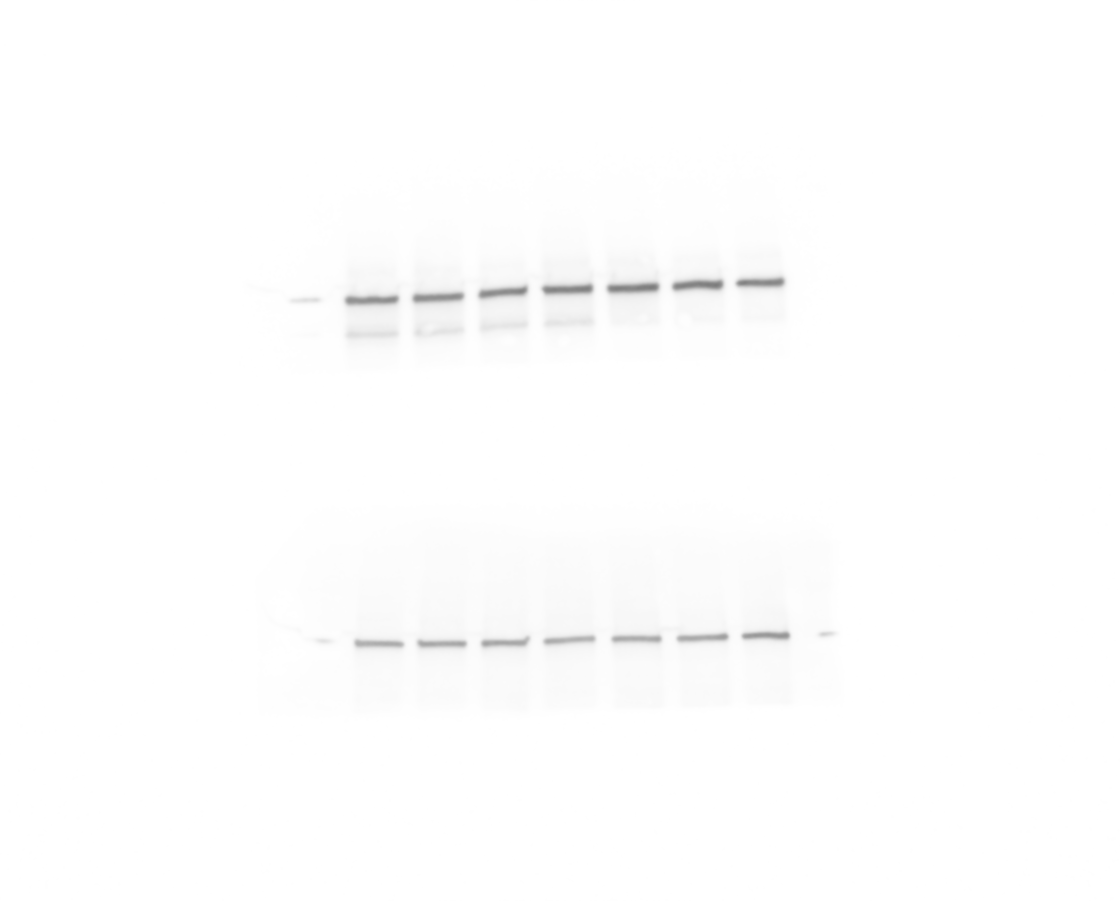

Supplement: Figure 3—source data 2. [file elife-100747-fig3-data2.zip › Figure 3 - Source Data 2 (original western files)/hsp90/23.07.27_17.21.11_S1_F05.tif]

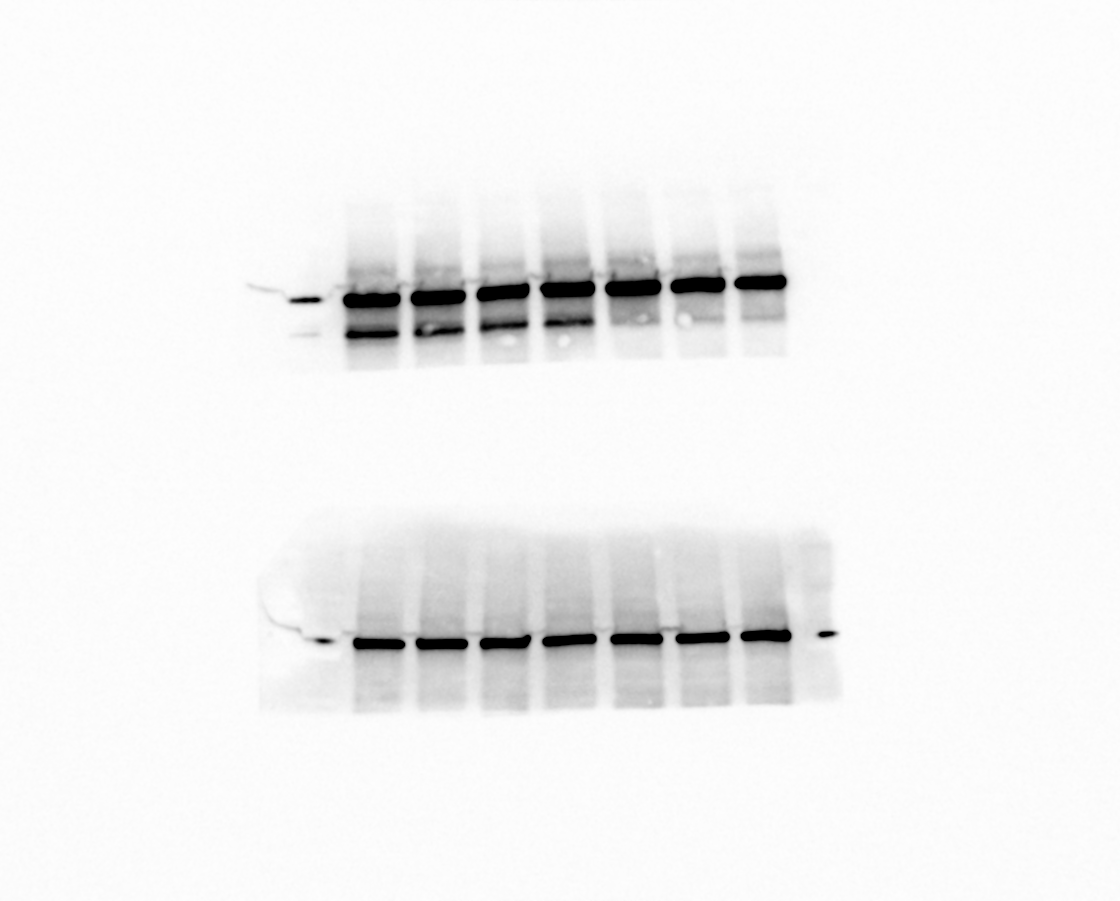

Supplement: Figure 3—source data 2. [file elife-100747-fig3-data2.zip › Figure 3 - Source Data 2 (original western files)/hsp90/23.07.27_17.21.11_S1_F05_PUB_600.tif]

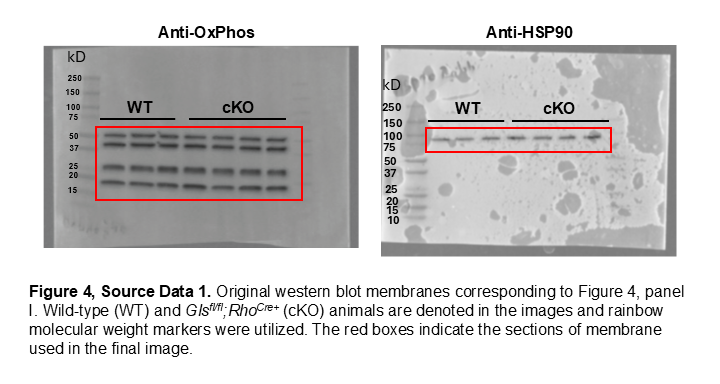

Supplement: Figure 4—source data 1. — Wild-type (WT) and Glsfl/fl;RhoCre+ (cKO) animals are denoted in the images and rainbow molecular weight markers were utilized. The red boxes indicate the sections of membrane used in the final image. [file elife-100747-fig4-data1.zip › Figure 4 - Source Data 1 (annotated western file)/Figure 4 - Source Data 1.tif]

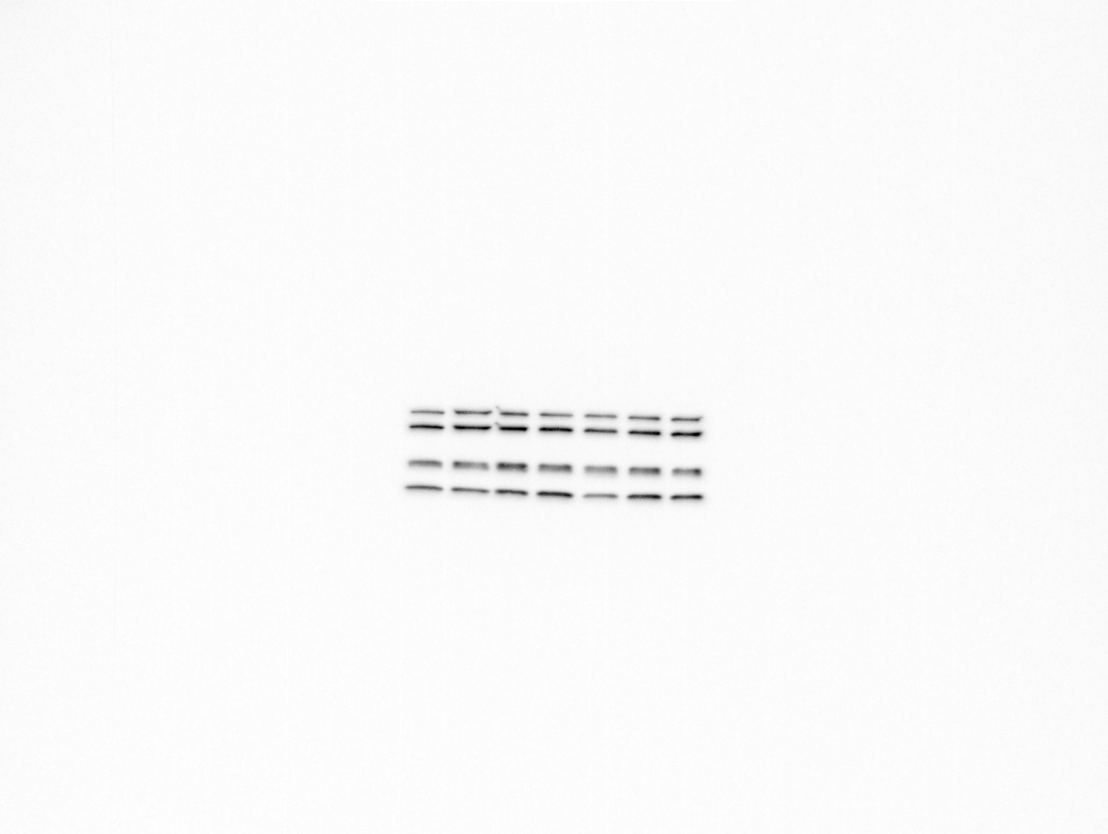

Supplement: Figure 4—source data 2. [file elife-100747-fig4-data2.zip › Figure 4 - Source Data 2 (original western files)/oxphos/2022-1118-155726.tif]

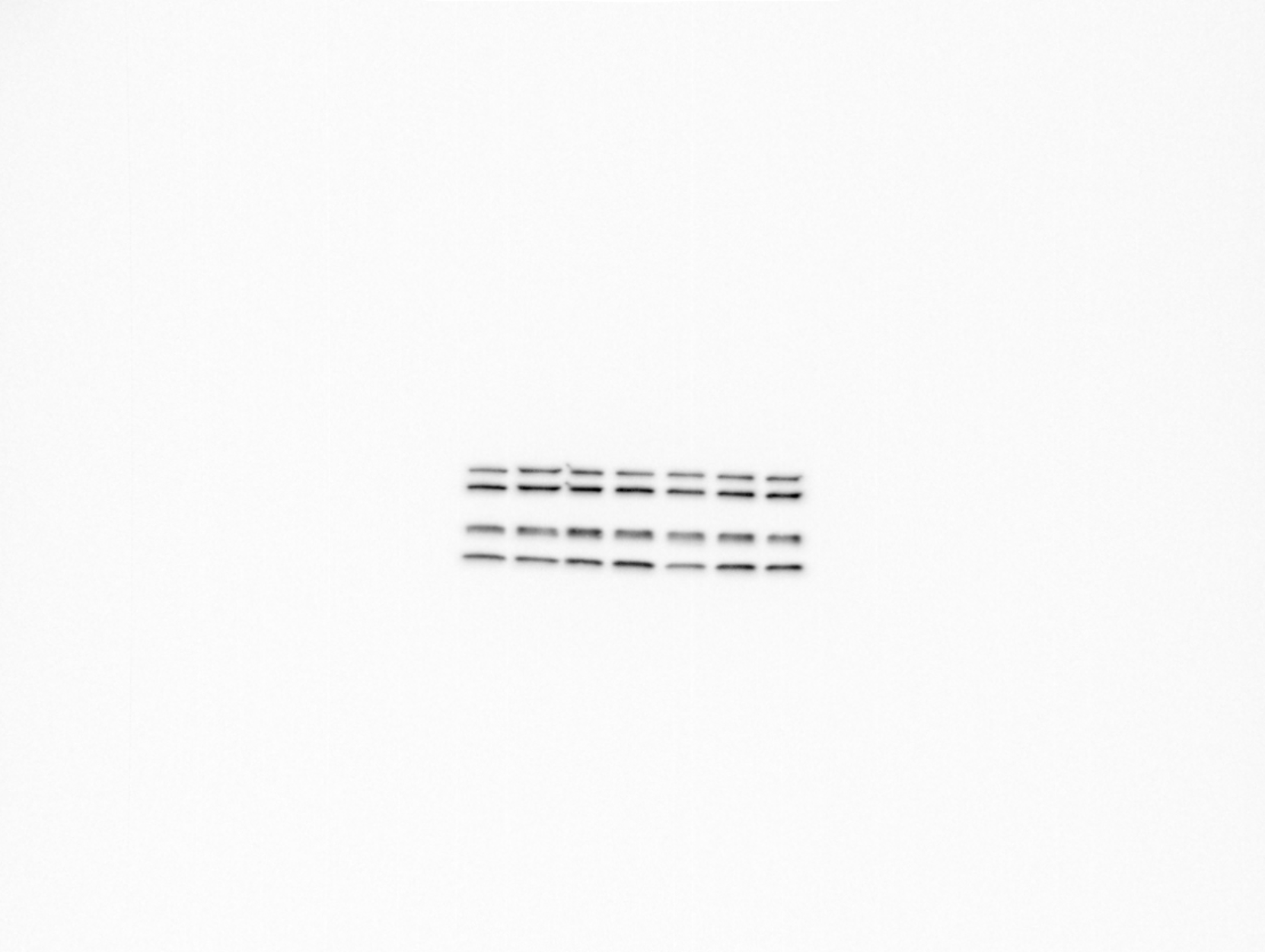

Supplement: Figure 4—source data 2. [file elife-100747-fig4-data2.zip › Figure 4 - Source Data 2 (original western files)/oxphos/2022-1118-155726_pub.tif]

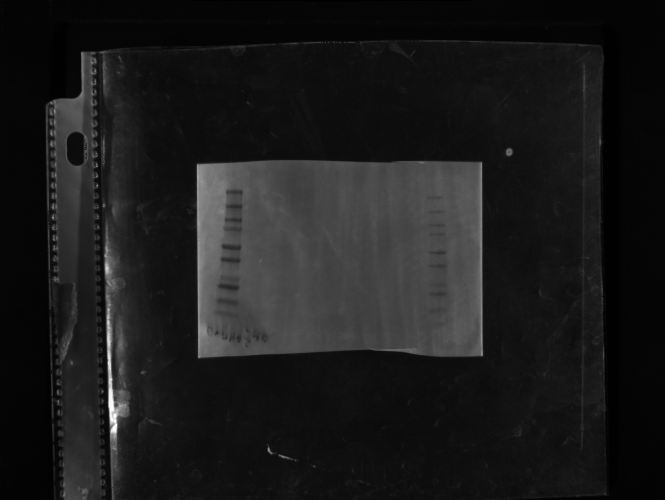

Supplement: Figure 4—source data 2. [file elife-100747-fig4-data2.zip › Figure 4 - Source Data 2 (original western files)/oxphos/2022-1118-155728.tif]

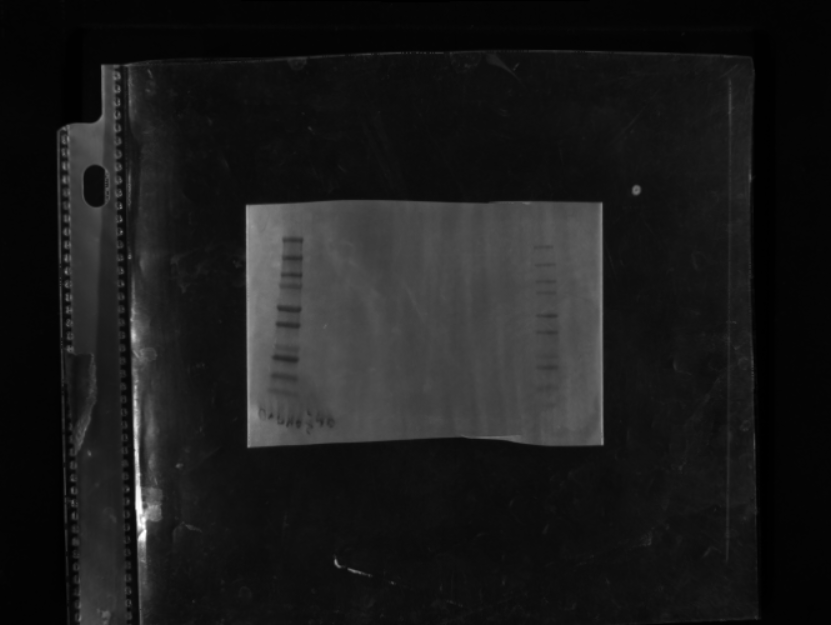

Supplement: Figure 4—source data 2. [file elife-100747-fig4-data2.zip › Figure 4 - Source Data 2 (original western files)/oxphos/2022-1118-155728_pub.tif]

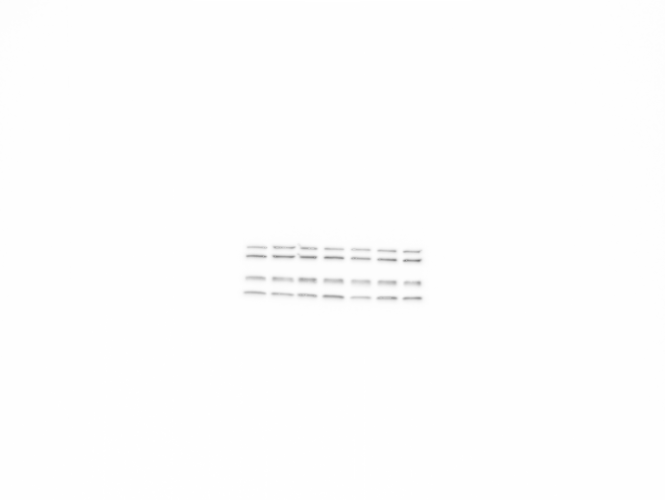

Supplement: Figure 4—source data 2. [file elife-100747-fig4-data2.zip › Figure 4 - Source Data 2 (original western files)/oxphos/2022-1118-155729.tif]

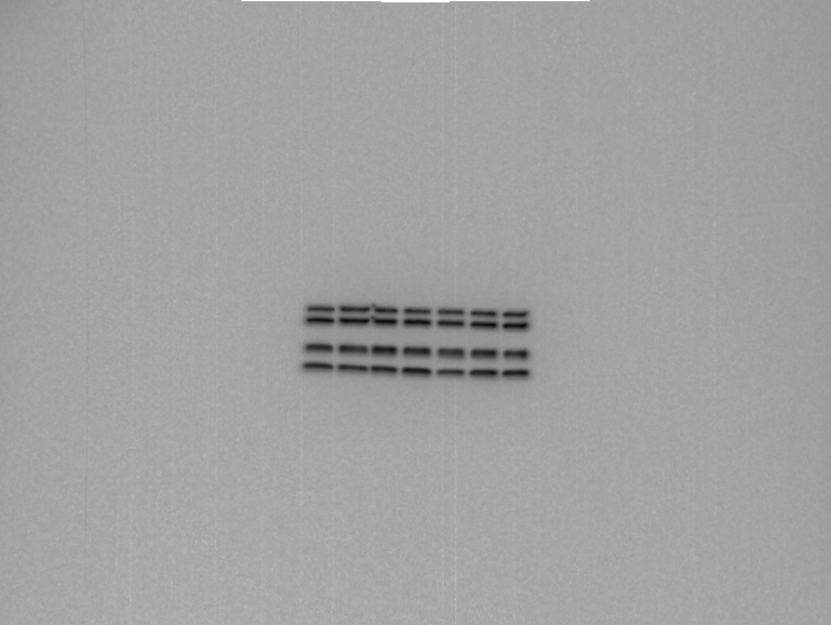

Supplement: Figure 4—source data 2. [file elife-100747-fig4-data2.zip › Figure 4 - Source Data 2 (original western files)/oxphos/2022-1118-155729_pub.tif]

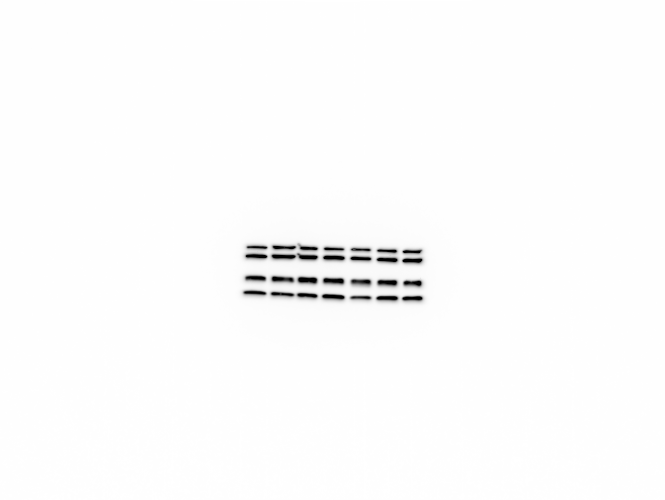

Supplement: Figure 4—source data 2. [file elife-100747-fig4-data2.zip › Figure 4 - Source Data 2 (original western files)/oxphos/2022-1118-155751.tif]

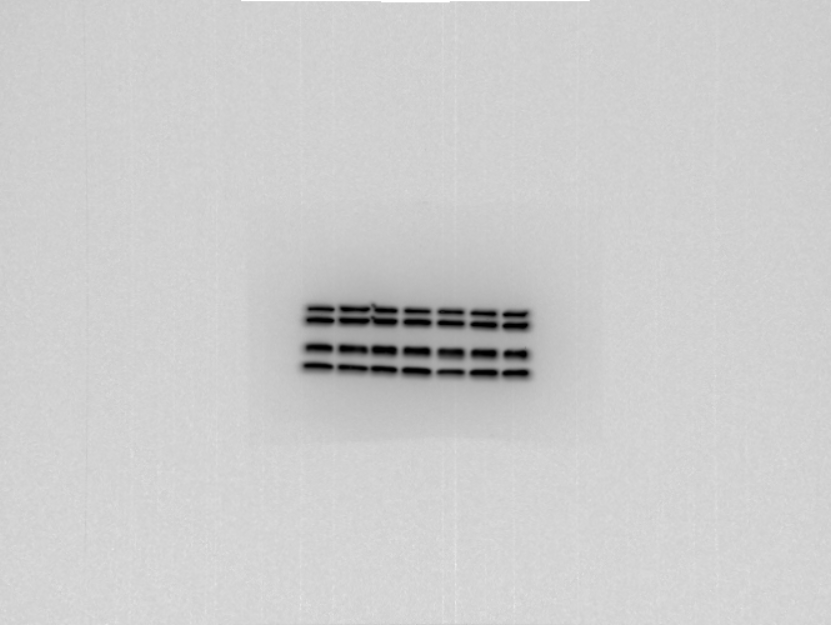

Supplement: Figure 4—source data 2. [file elife-100747-fig4-data2.zip › Figure 4 - Source Data 2 (original western files)/oxphos/2022-1118-155751_pub.tif]

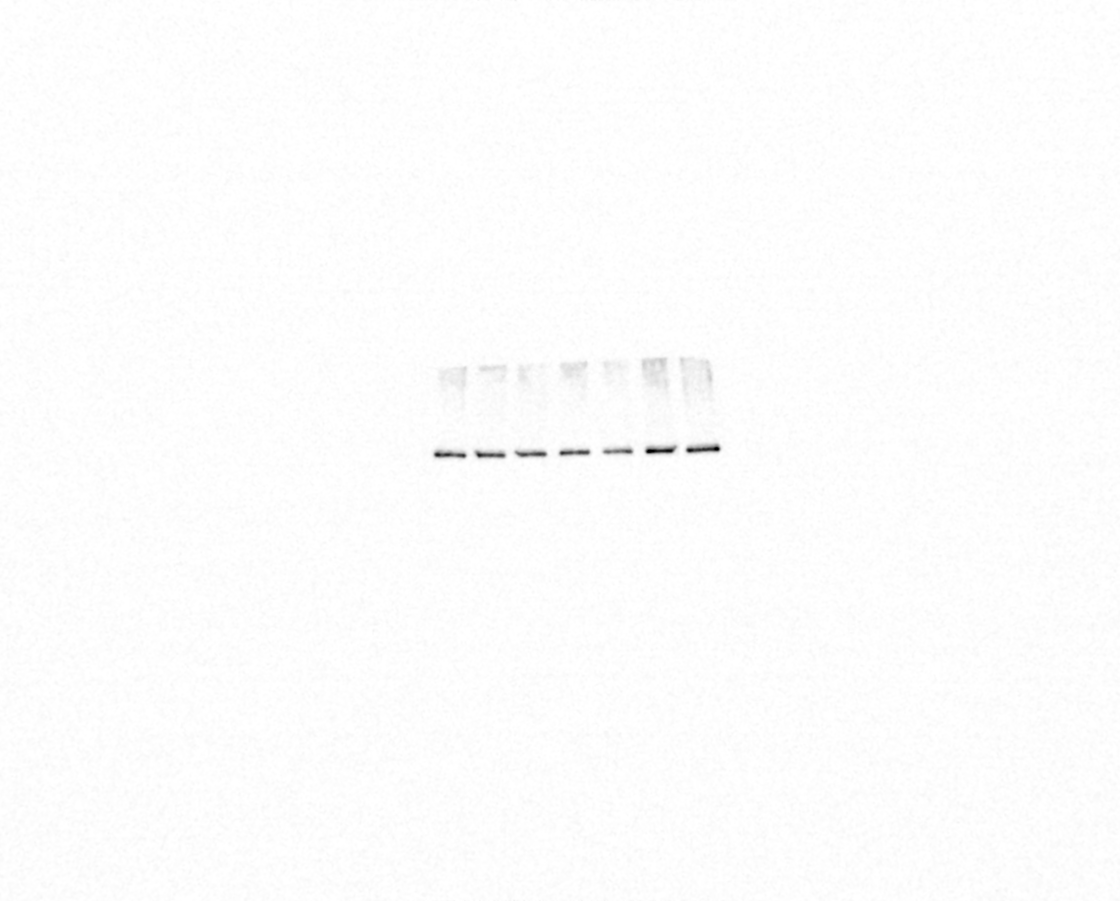

Supplement: Figure 4—source data 2. [file elife-100747-fig4-data2.zip › Figure 4 - Source Data 2 (original western files)/oxphos/hsp90/gel1/23.01.25_12.00.56.tif]

Office DEPOT®

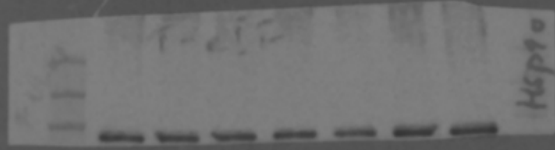

Supplement: Figure 4—source data 2. [file elife-100747-fig4-data2.zip › Figure 4 - Source Data 2 (original western files)/oxphos/hsp90/gel1/23.01.25_12.01.42.pdf]

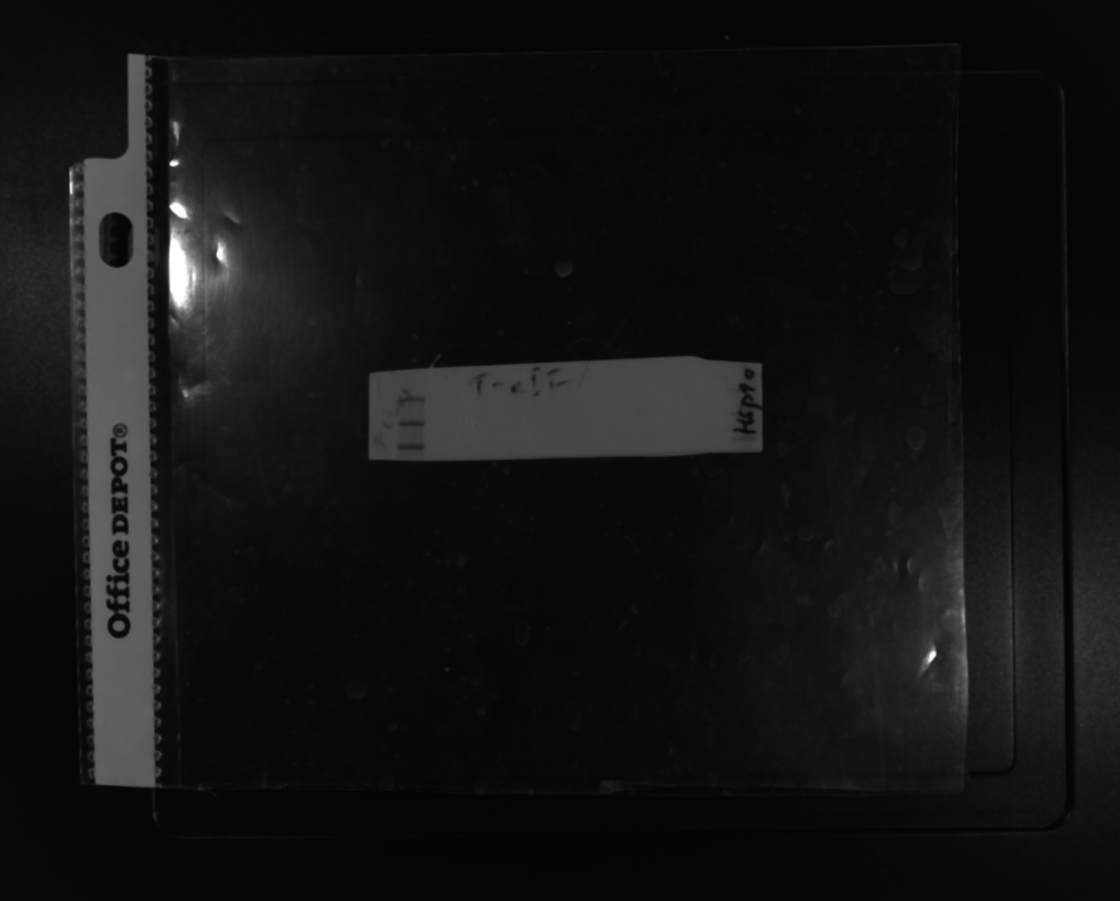

Supplement: Figure 4—source data 2. [file elife-100747-fig4-data2.zip › Figure 4 - Source Data 2 (original western files)/oxphos/hsp90/gel1/23.01.25_12.01.42.tif]

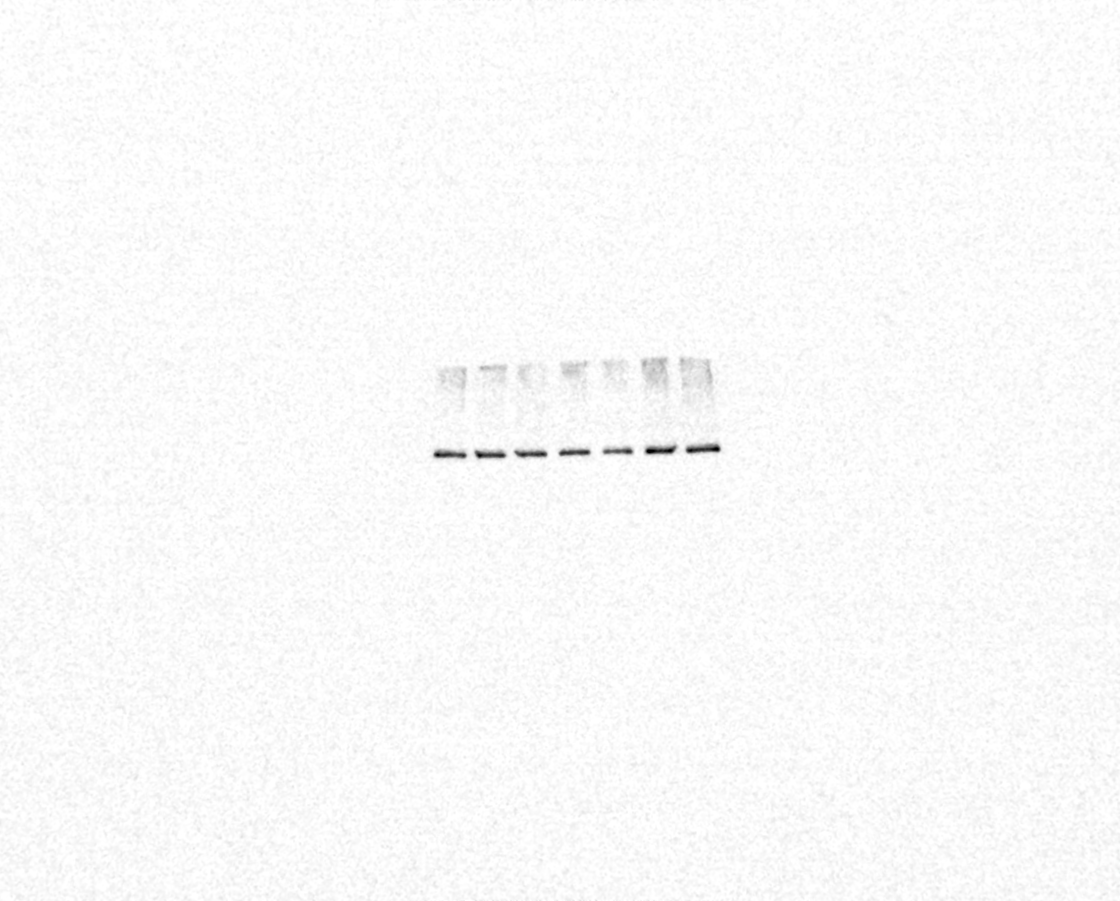

Supplement: Figure 4—source data 2. [file elife-100747-fig4-data2.zip › Figure 4 - Source Data 2 (original western files)/oxphos/hsp90/gel1/23.01.25_12.02.07_S1_F01.tif]

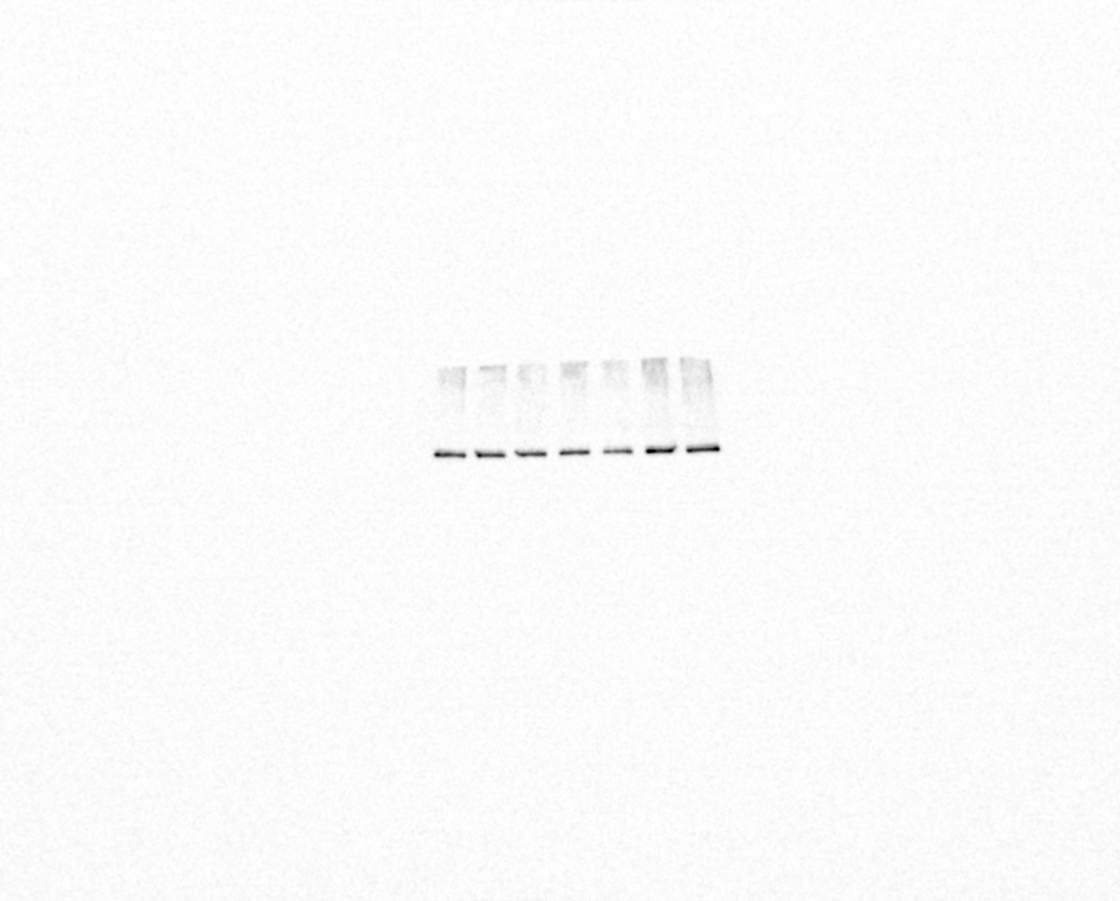

Supplement: Figure 4—source data 2. [file elife-100747-fig4-data2.zip › Figure 4 - Source Data 2 (original western files)/oxphos/hsp90/gel1/23.01.25_12.02.07_S1_F02.tif]

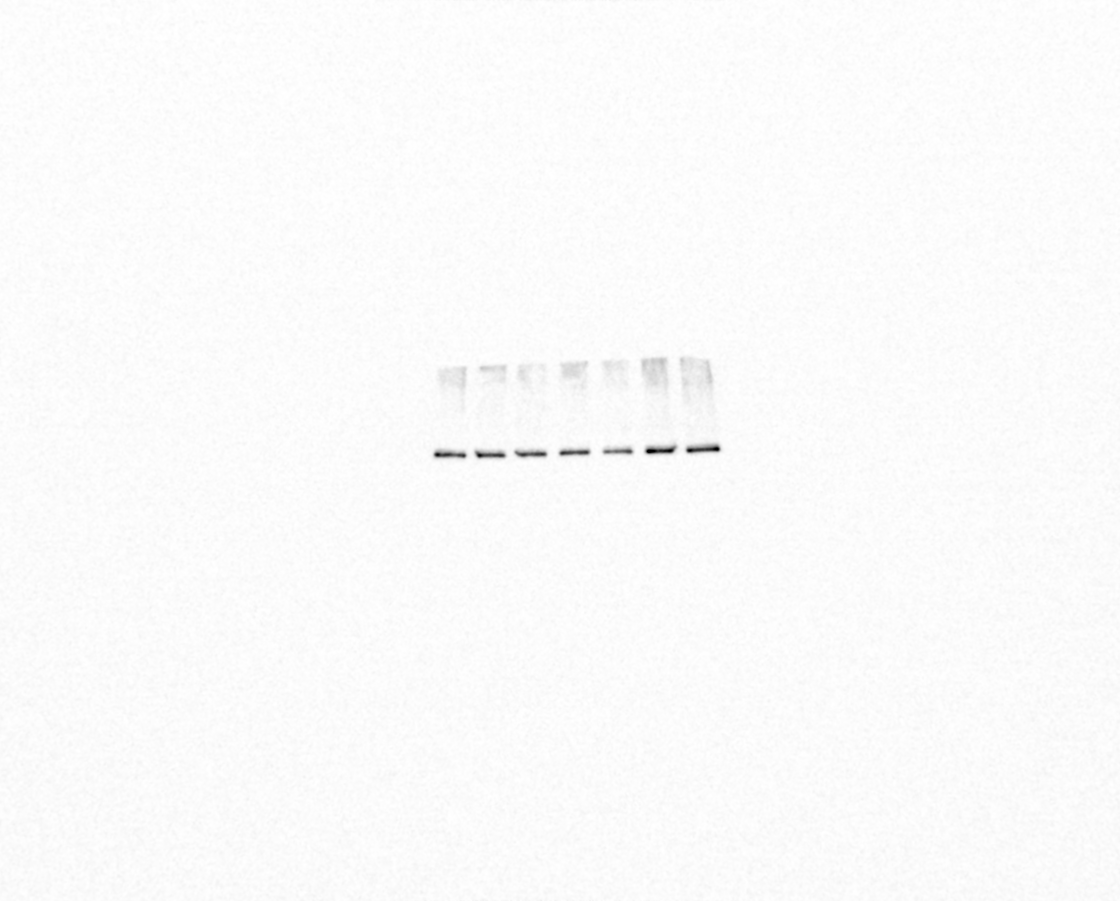

Supplement: Figure 4—source data 2. [file elife-100747-fig4-data2.zip › Figure 4 - Source Data 2 (original western files)/oxphos/hsp90/gel1/23.01.25_12.02.07_S1_F03.tif]

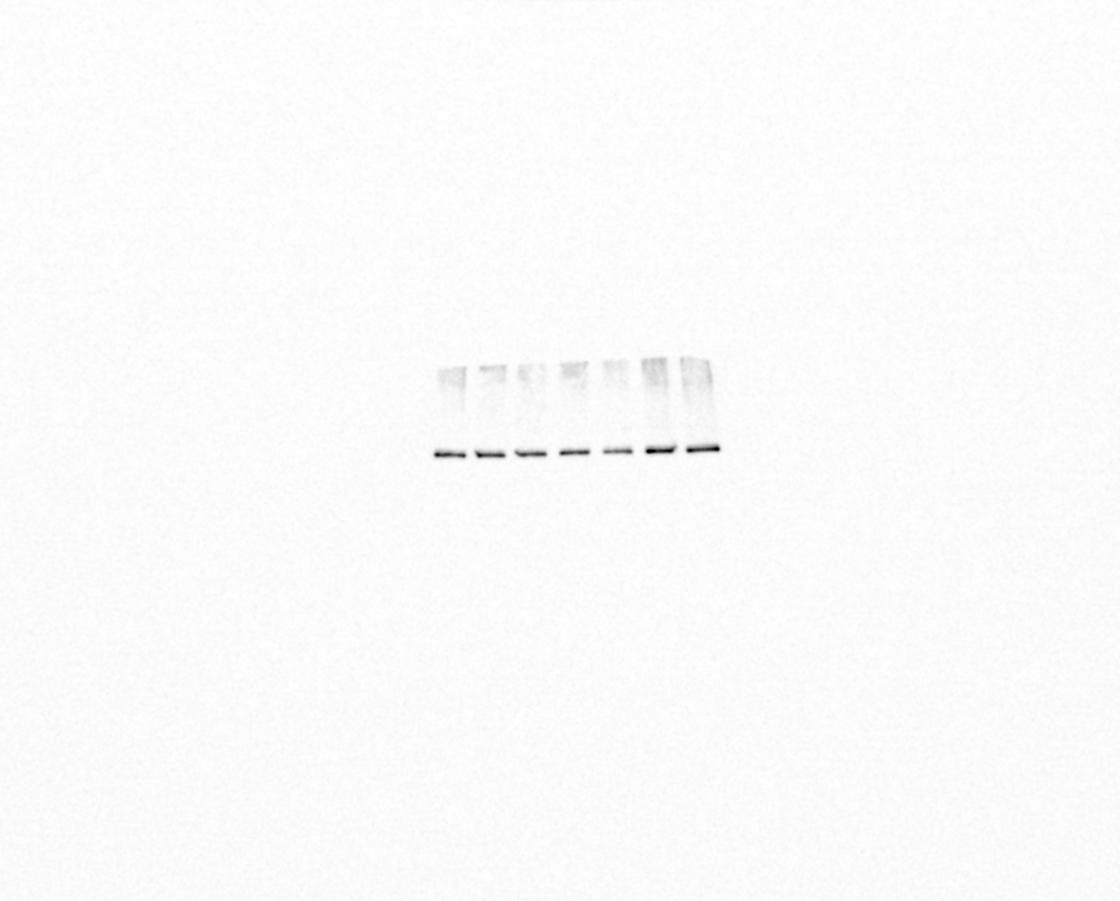

Supplement: Figure 4—source data 2. [file elife-100747-fig4-data2.zip › Figure 4 - Source Data 2 (original western files)/oxphos/hsp90/gel1/23.01.25_12.02.07_S1_F04.tif]

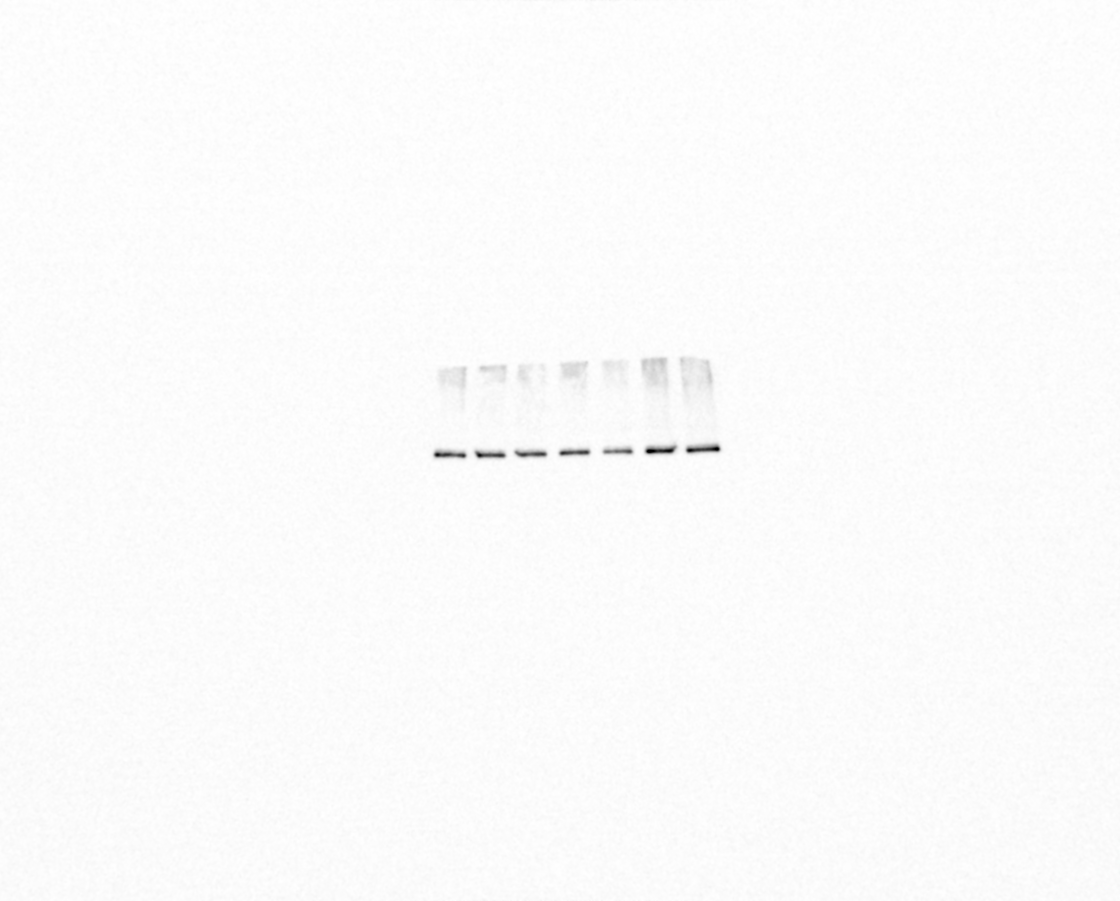

Supplement: Figure 4—source data 2. [file elife-100747-fig4-data2.zip › Figure 4 - Source Data 2 (original western files)/oxphos/hsp90/gel1/23.01.25_12.02.07_S1_F05.tif]

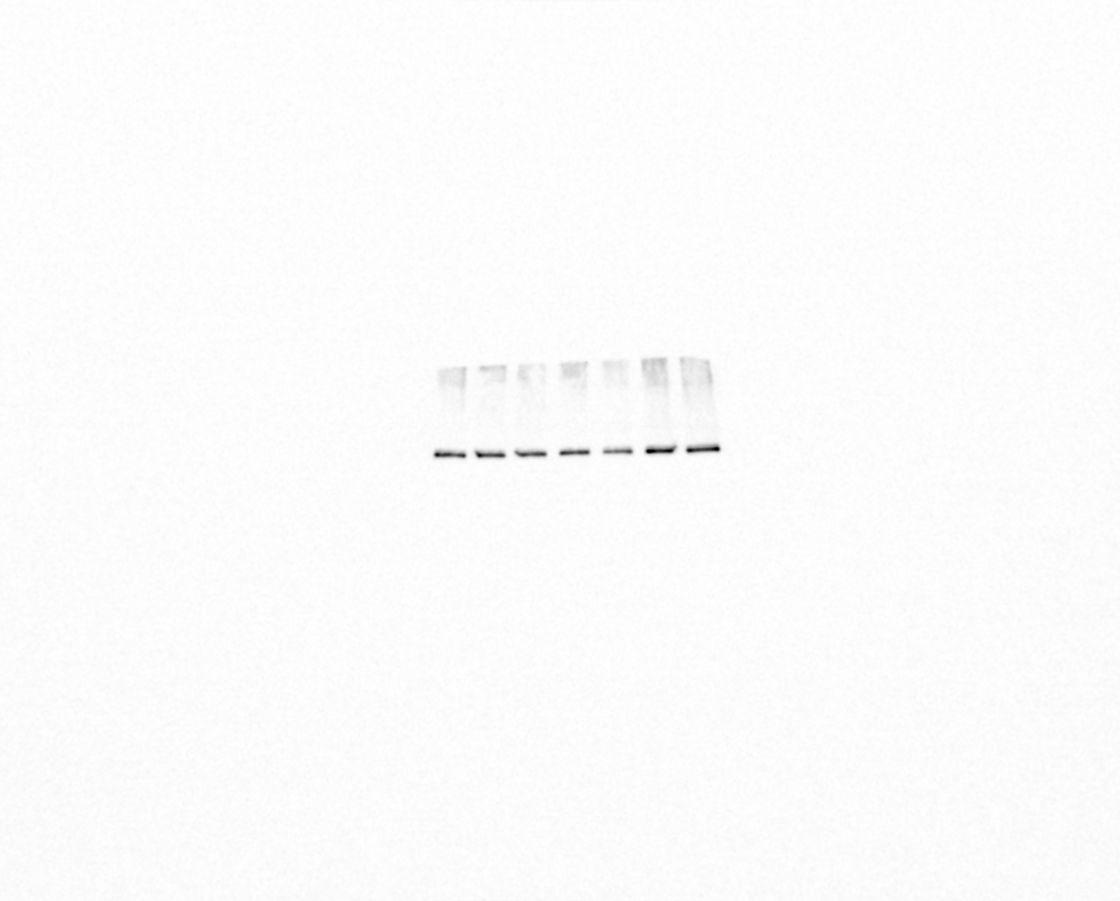

Supplement: Figure 4—source data 2. [file elife-100747-fig4-data2.zip › Figure 4 - Source Data 2 (original western files)/oxphos/hsp90/gel1/23.01.25_12.02.07_S1_F06.tif]

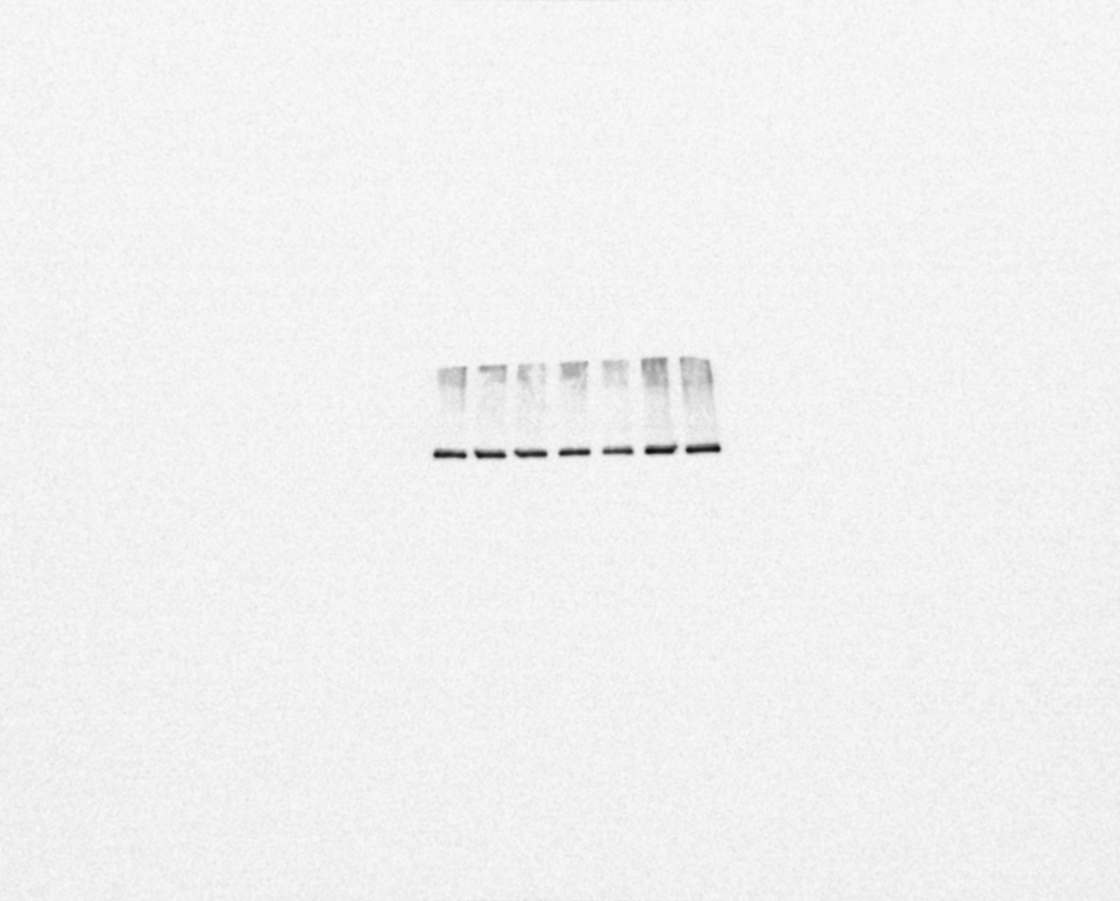

Supplement: Figure 4—source data 2. [file elife-100747-fig4-data2.zip › Figure 4 - Source Data 2 (original western files)/oxphos/hsp90/gel1/23.01.25_12.02.07_S1_F07.tif]

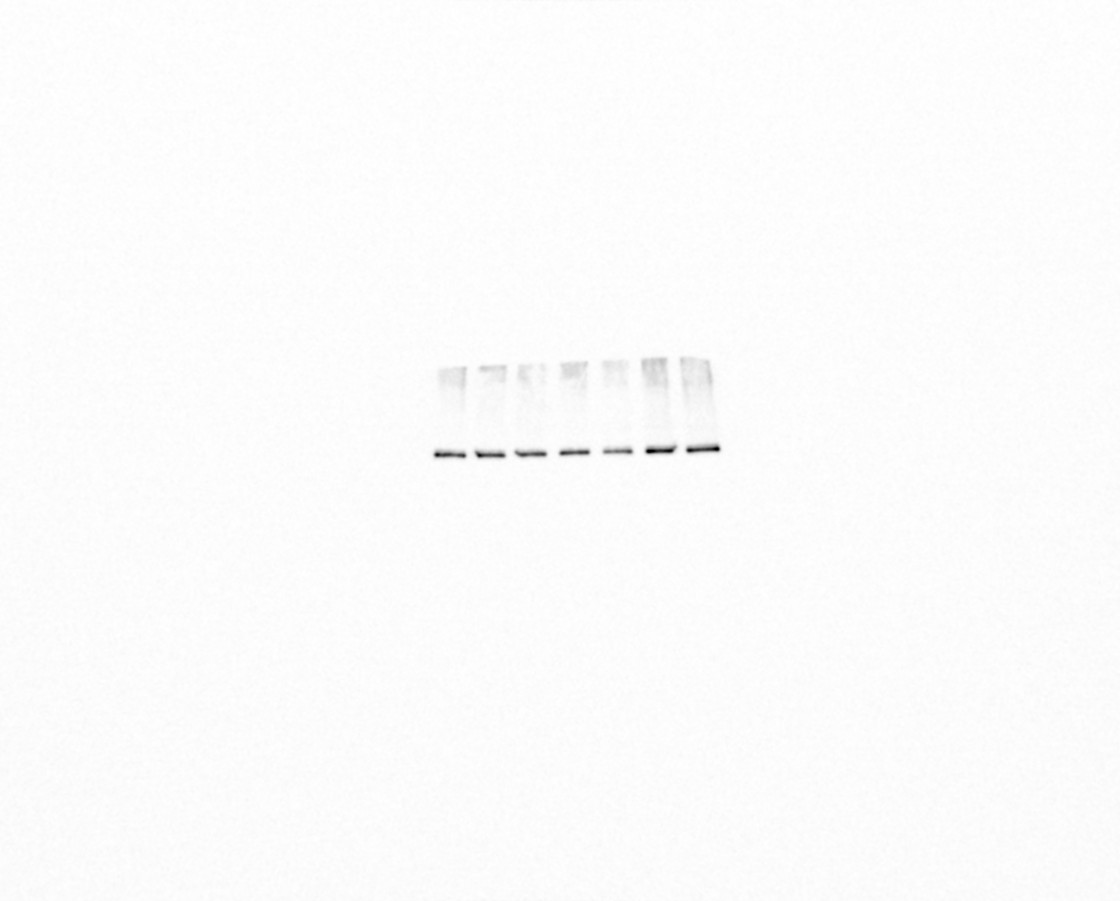

Supplement: Figure 4—source data 2. [file elife-100747-fig4-data2.zip › Figure 4 - Source Data 2 (original western files)/oxphos/hsp90/gel1/23.01.25_12.02.07_S1_F08.tif]

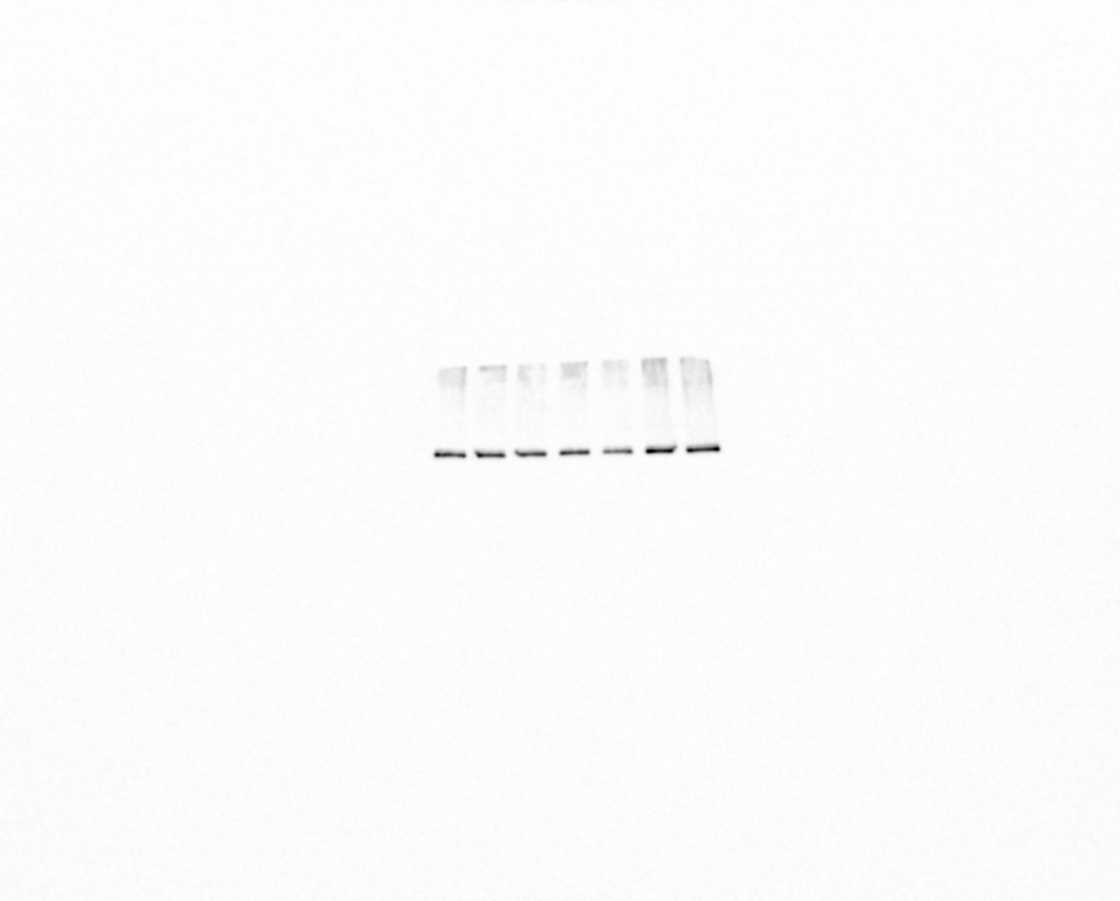

Supplement: Figure 4—source data 2. [file elife-100747-fig4-data2.zip › Figure 4 - Source Data 2 (original western files)/oxphos/hsp90/gel1/23.01.25_12.02.07_S1_F09.tif]

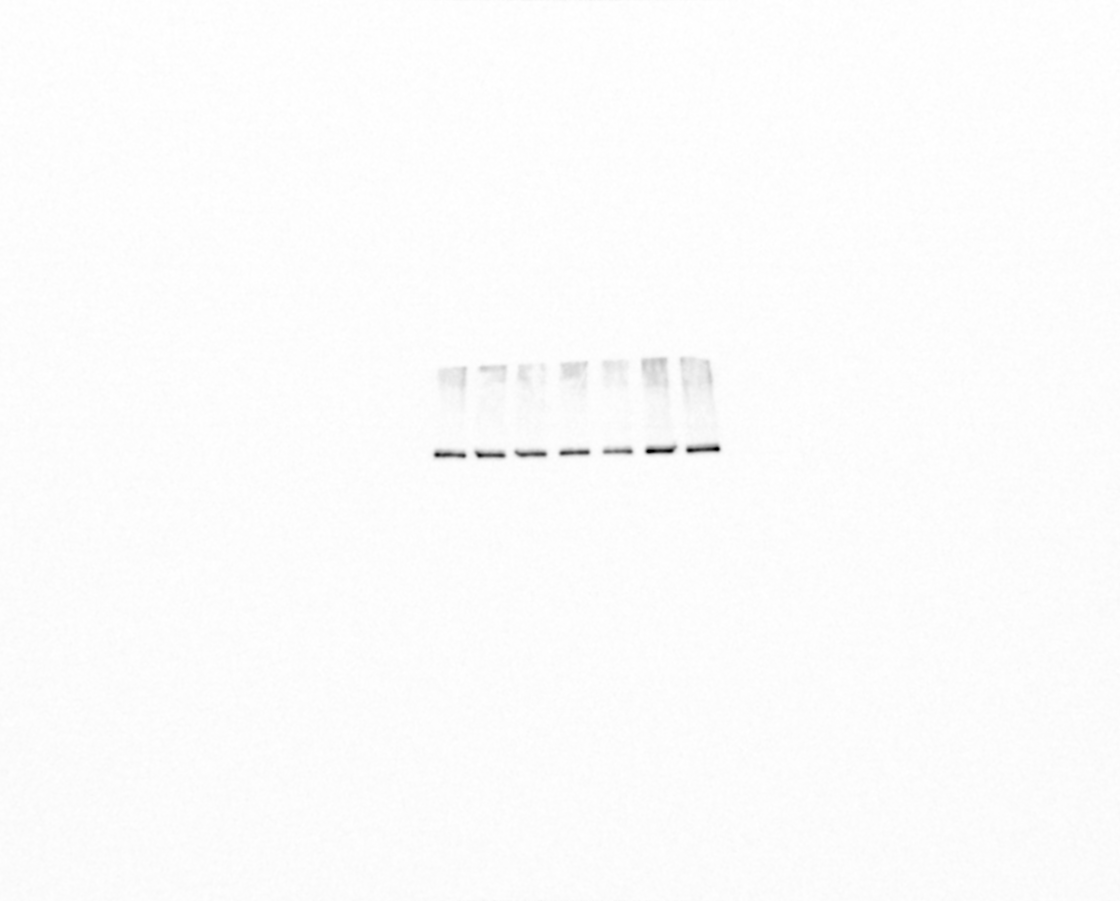

Supplement: Figure 4—source data 2. [file elife-100747-fig4-data2.zip › Figure 4 - Source Data 2 (original western files)/oxphos/hsp90/gel1/23.01.25_12.02.07_S1_F10.tif]

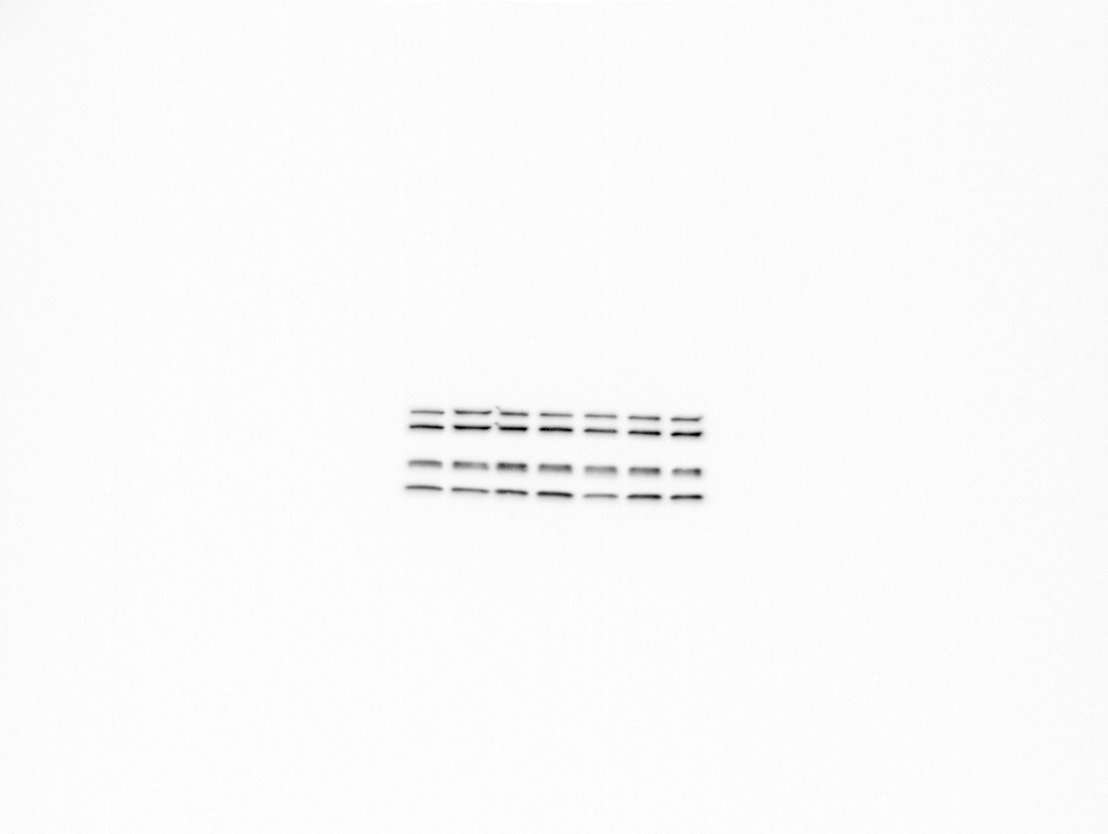

Supplement: Figure 4—source data 2. [file elife-100747-fig4-data2.zip › Figure 4 - Source Data 2 (original western files)/oxphos/S3F1-1118-155730.tif]

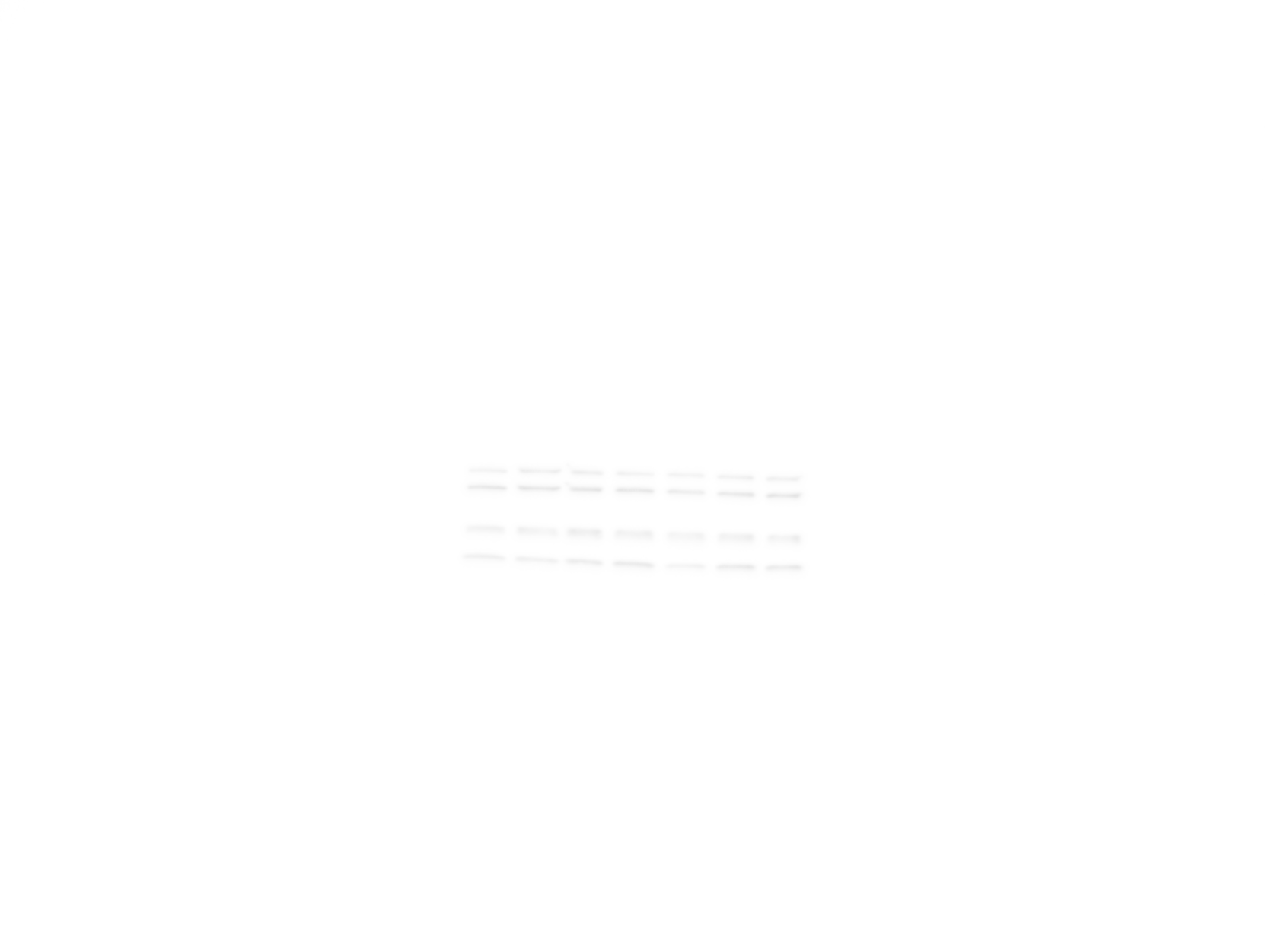

Supplement: Figure 4—source data 2. [file elife-100747-fig4-data2.zip › Figure 4 - Source Data 2 (original western files)/oxphos/S3F1-1118-155730_pub.tif]
