# Supplementary figures and images for: Glutamine catabolism supports amino acid biosynthesis and suppresses the integrated stress response to promote photoreceptor survival (part 3 of 4)
Source: eLife. 2025 May 21;13:RP100747. doi: 10.7554/eLife.100747 (PMC12094702; doi:10.7554/eLife.100747)

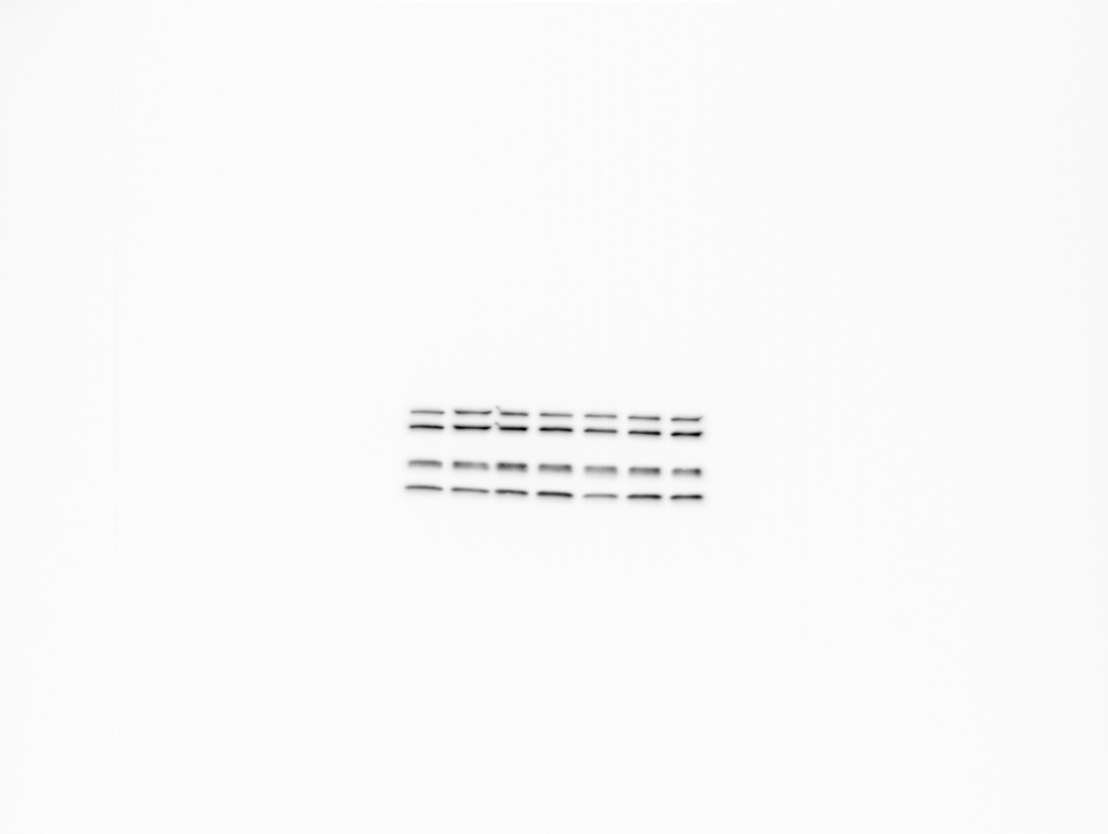

Supplement: Figure 4—source data 2. [file elife-100747-fig4-data2.zip › Figure 4 - Source Data 2 (original western files)/oxphos/S3F10-1118-155749.tif]

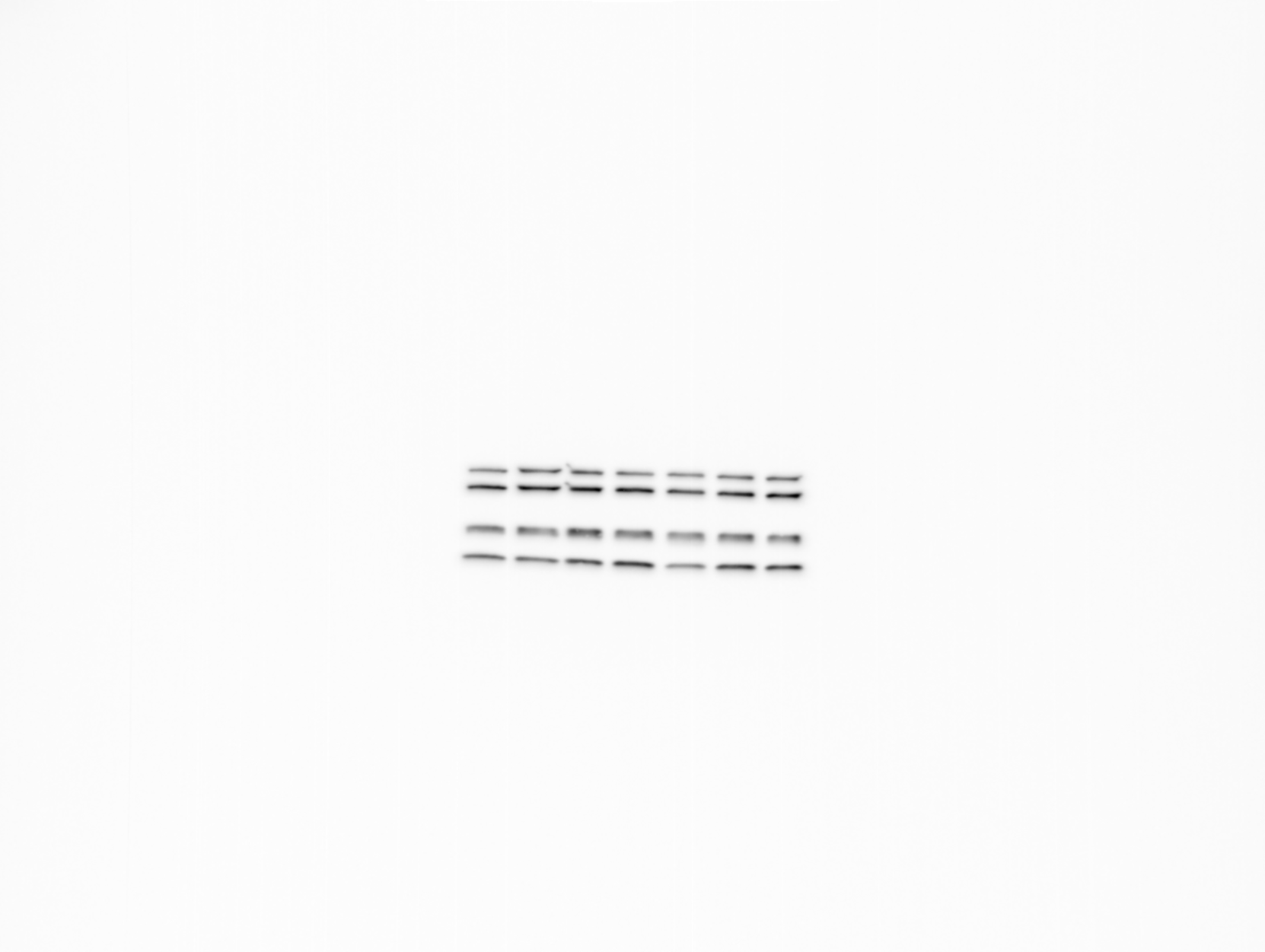

Supplement: Figure 4—source data 2. [file elife-100747-fig4-data2.zip › Figure 4 - Source Data 2 (original western files)/oxphos/S3F10-1118-155749_pub.tif]

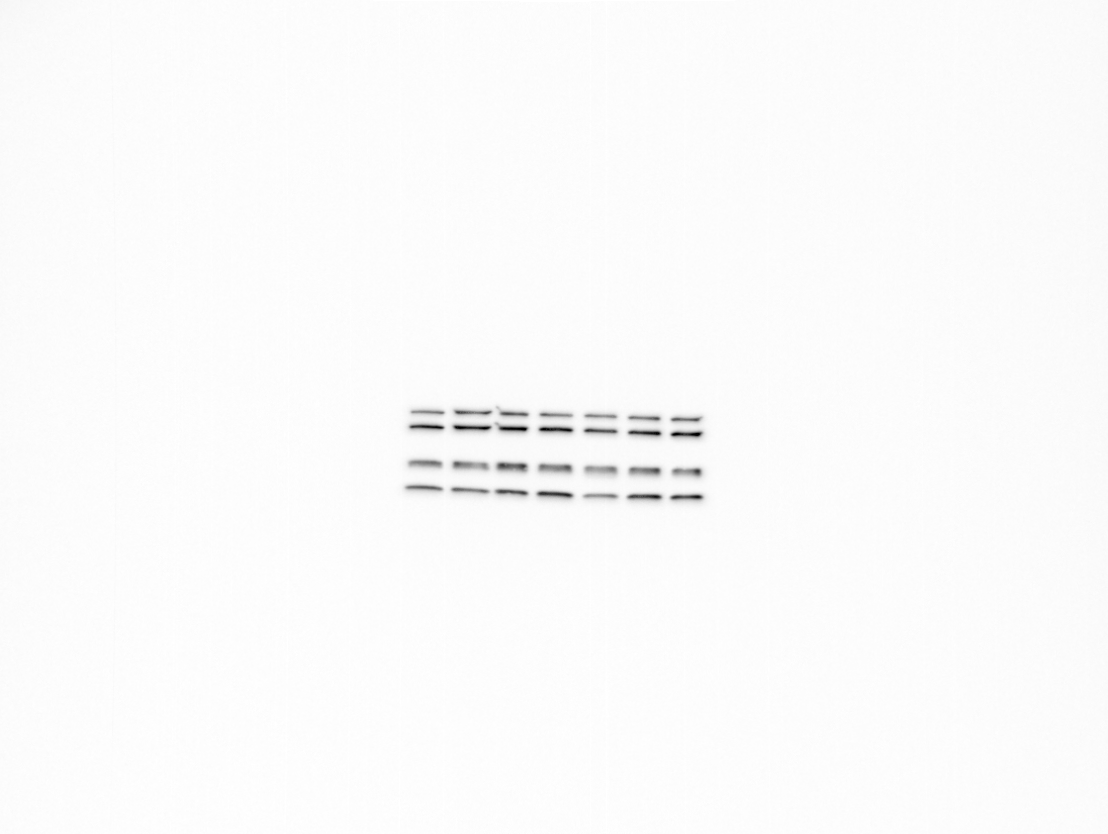

Supplement: Figure 4—source data 2. [file elife-100747-fig4-data2.zip › Figure 4 - Source Data 2 (original western files)/oxphos/S3F2-1118-155732.tif]

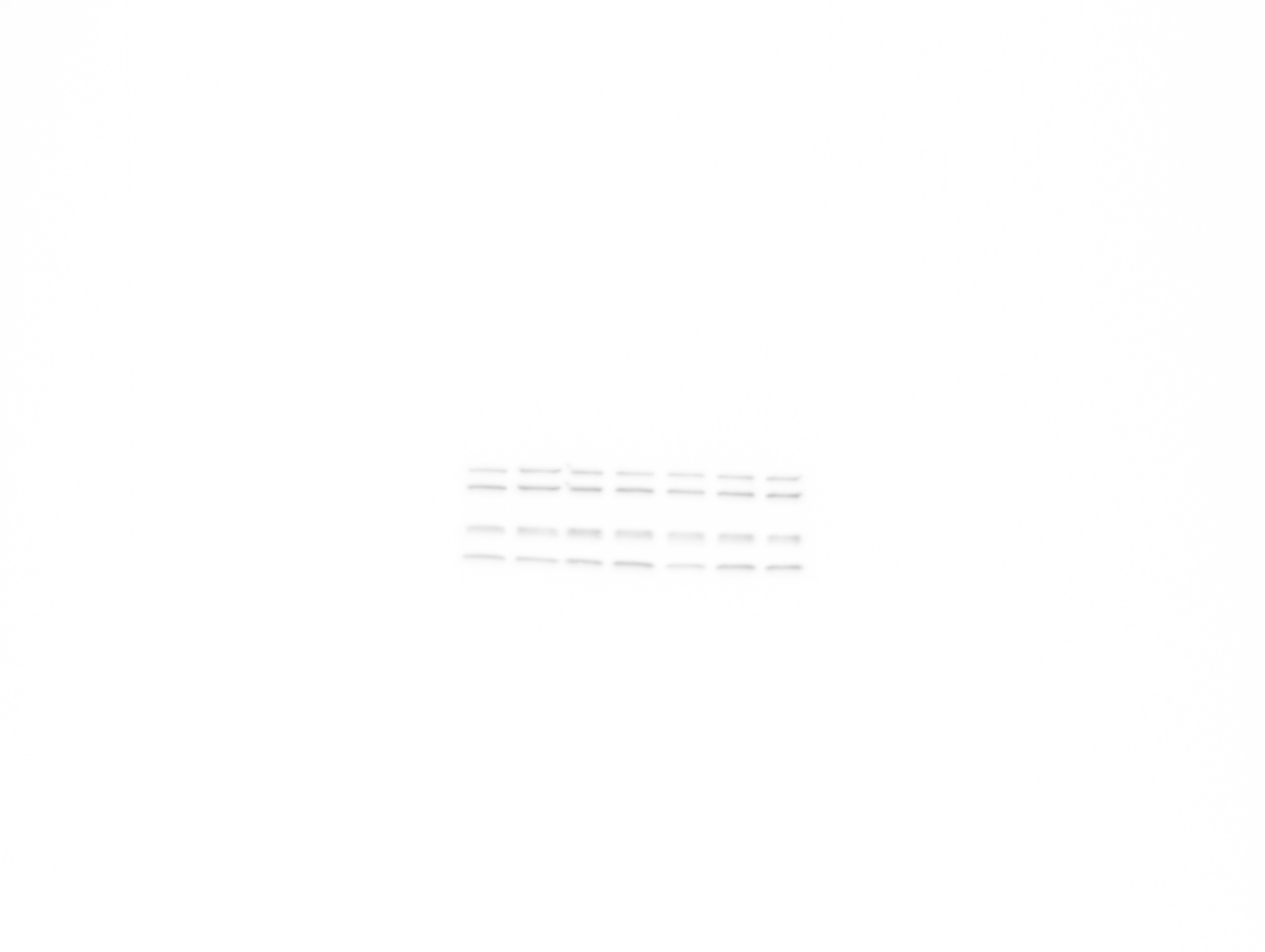

Supplement: Figure 4—source data 2. [file elife-100747-fig4-data2.zip › Figure 4 - Source Data 2 (original western files)/oxphos/S3F2-1118-155732_pub.tif]

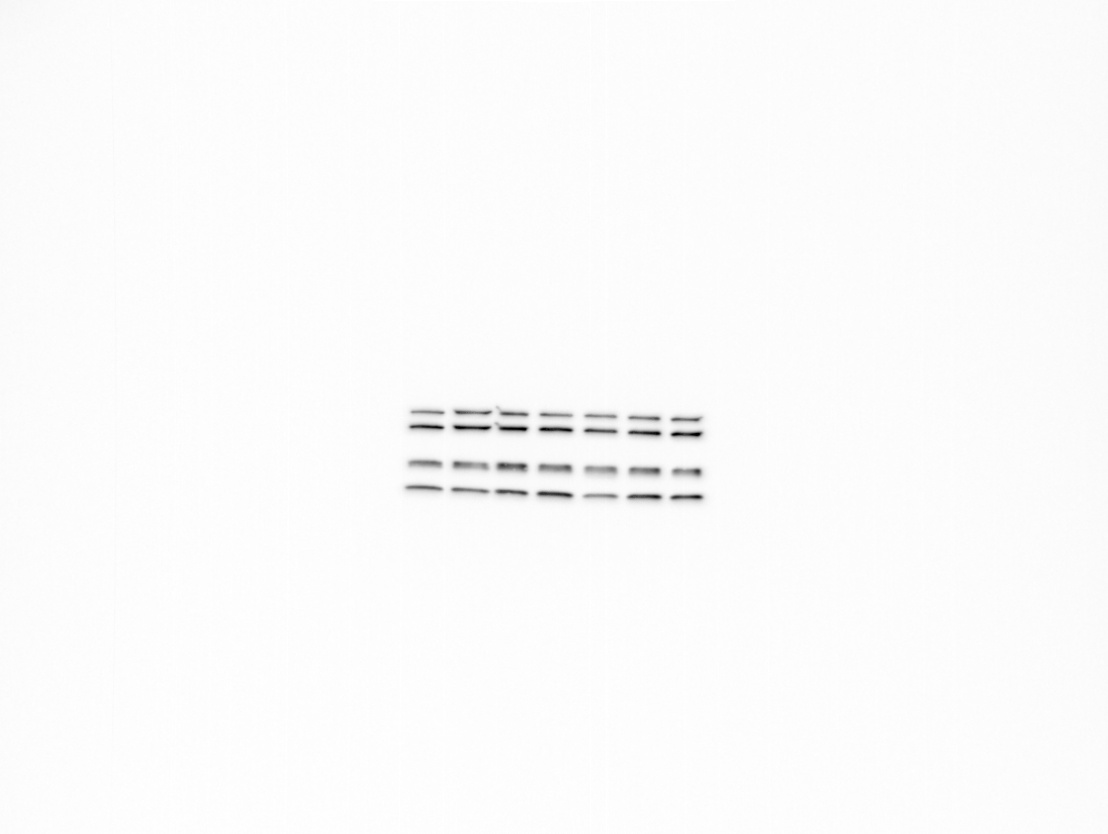

Supplement: Figure 4—source data 2. [file elife-100747-fig4-data2.zip › Figure 4 - Source Data 2 (original western files)/oxphos/S3F3-1118-155734.tif]

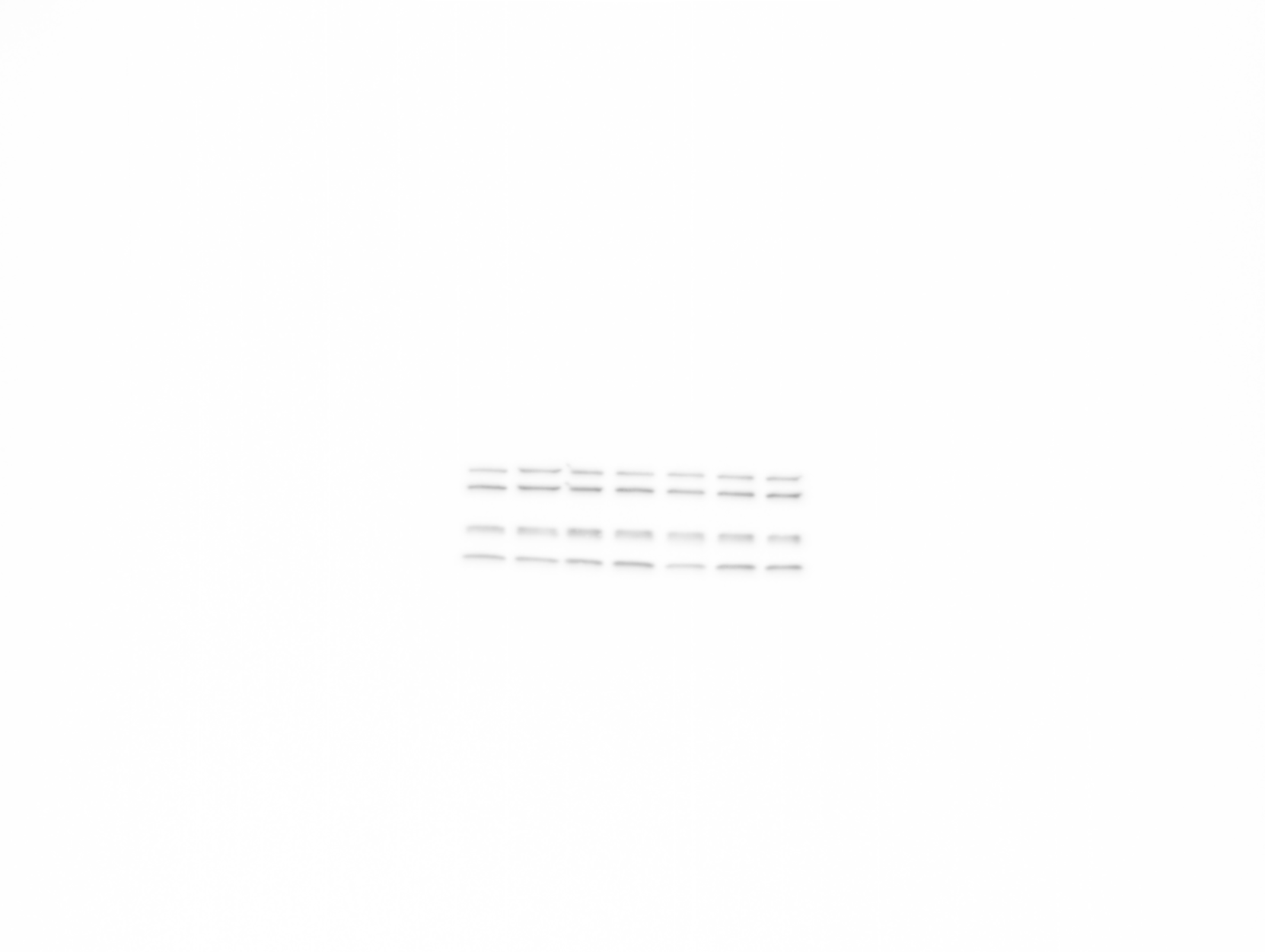

Supplement: Figure 4—source data 2. [file elife-100747-fig4-data2.zip › Figure 4 - Source Data 2 (original western files)/oxphos/S3F3-1118-155734_pub.tif]

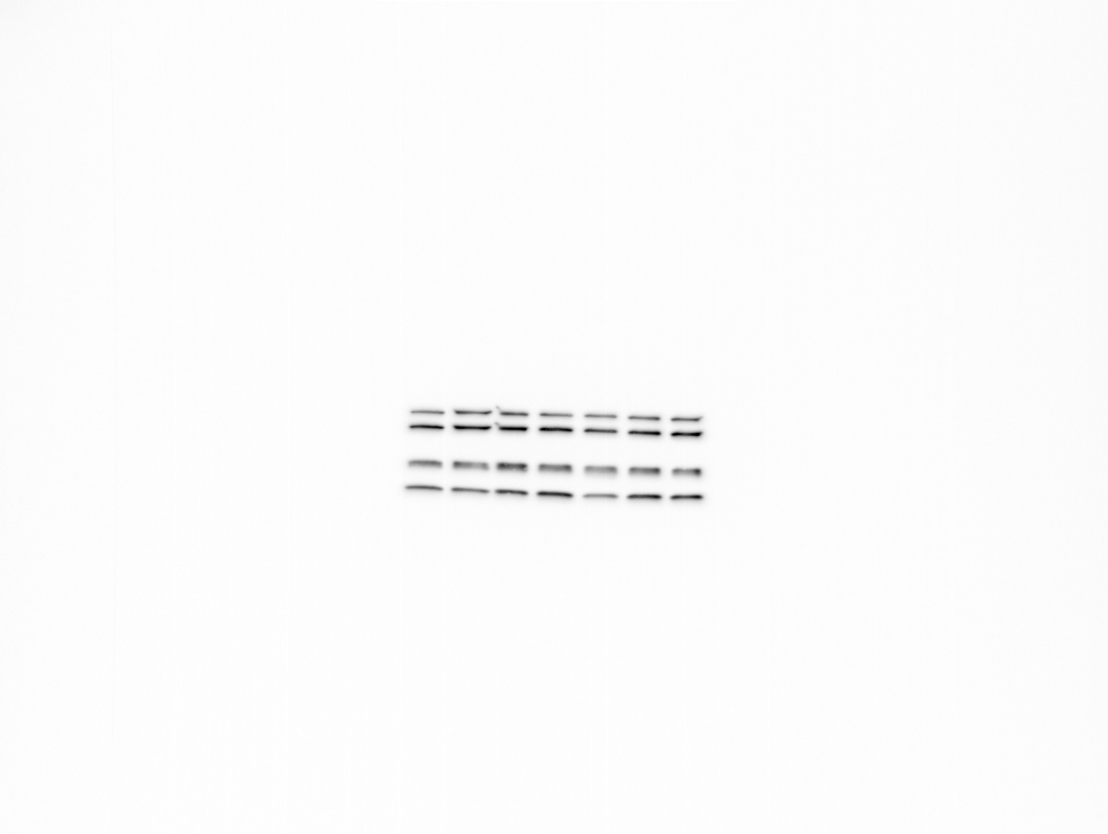

Supplement: Figure 4—source data 2. [file elife-100747-fig4-data2.zip › Figure 4 - Source Data 2 (original western files)/oxphos/S3F4-1118-155736.tif]

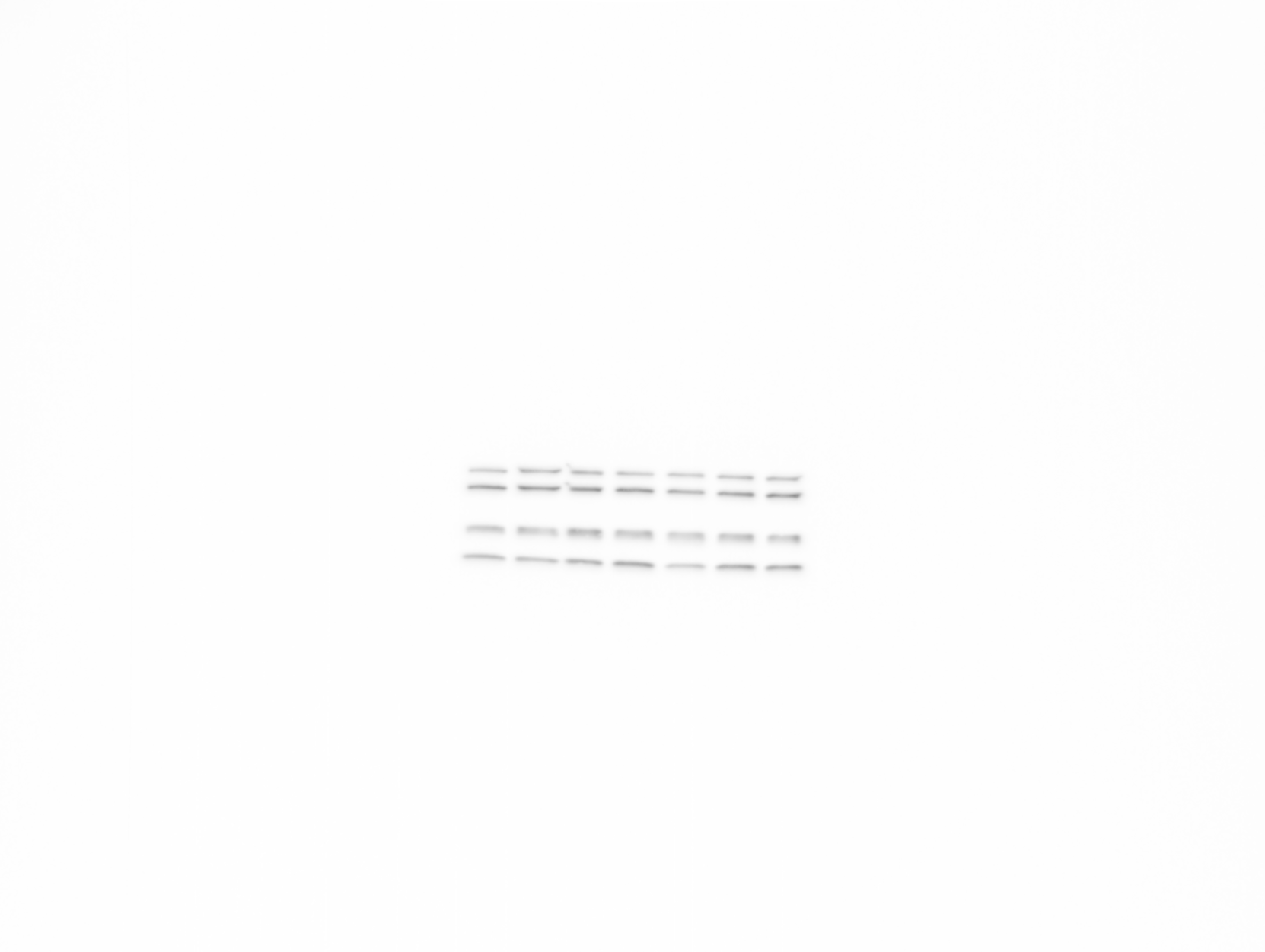

Supplement: Figure 4—source data 2. [file elife-100747-fig4-data2.zip › Figure 4 - Source Data 2 (original western files)/oxphos/S3F4-1118-155736_pub.tif]

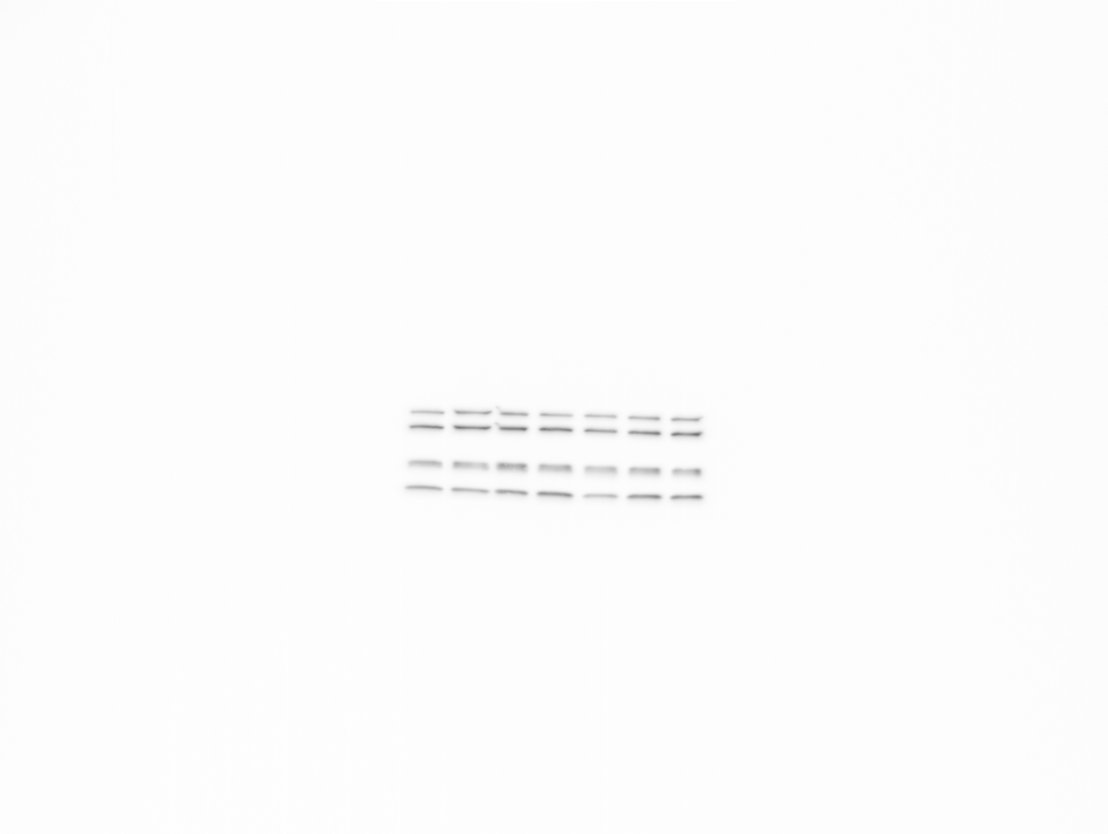

Supplement: Figure 4—source data 2. [file elife-100747-fig4-data2.zip › Figure 4 - Source Data 2 (original western files)/oxphos/S3F5-1118-155739.tif]

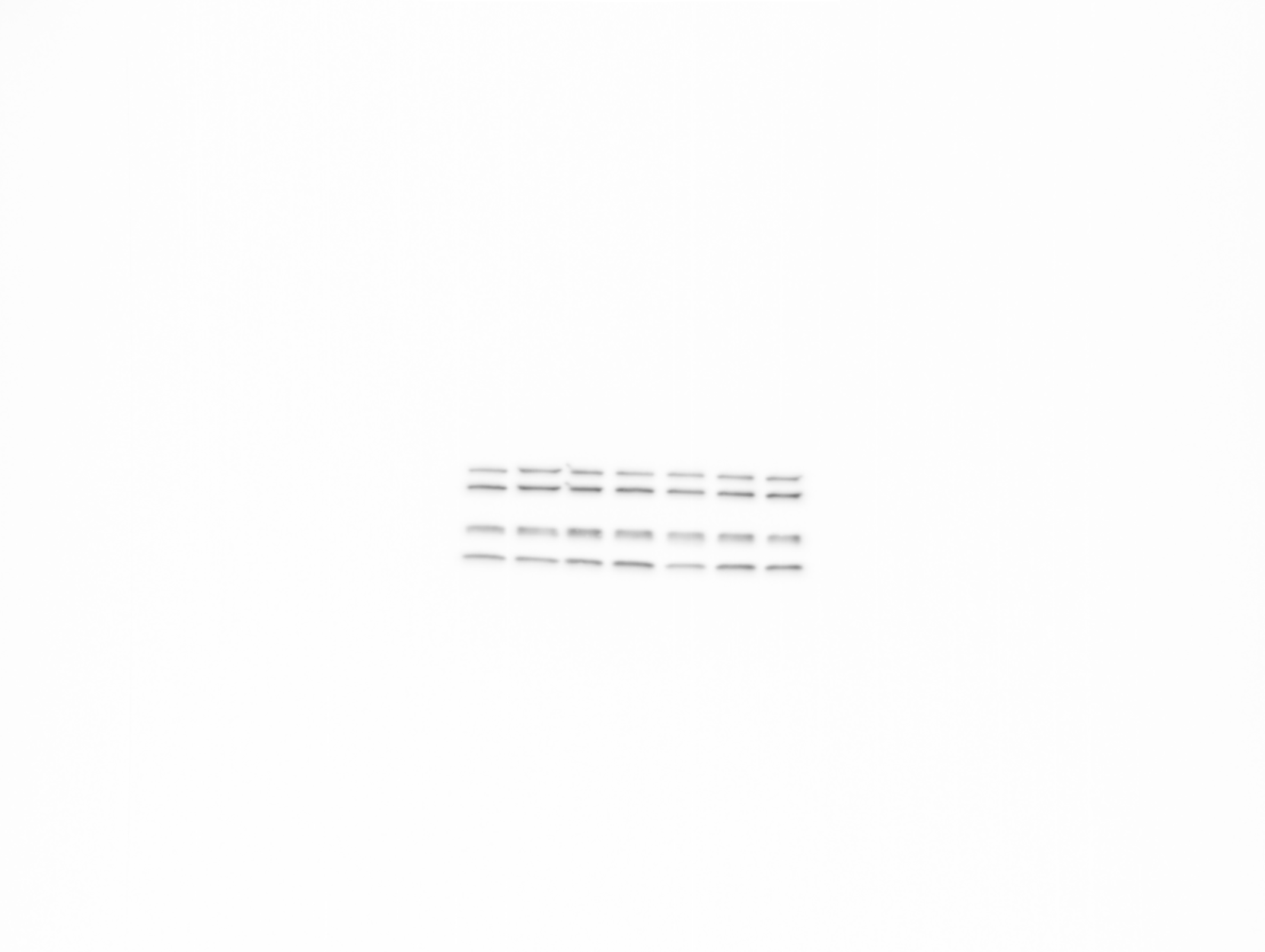

Supplement: Figure 4—source data 2. [file elife-100747-fig4-data2.zip › Figure 4 - Source Data 2 (original western files)/oxphos/S3F5-1118-155739_pub.tif]

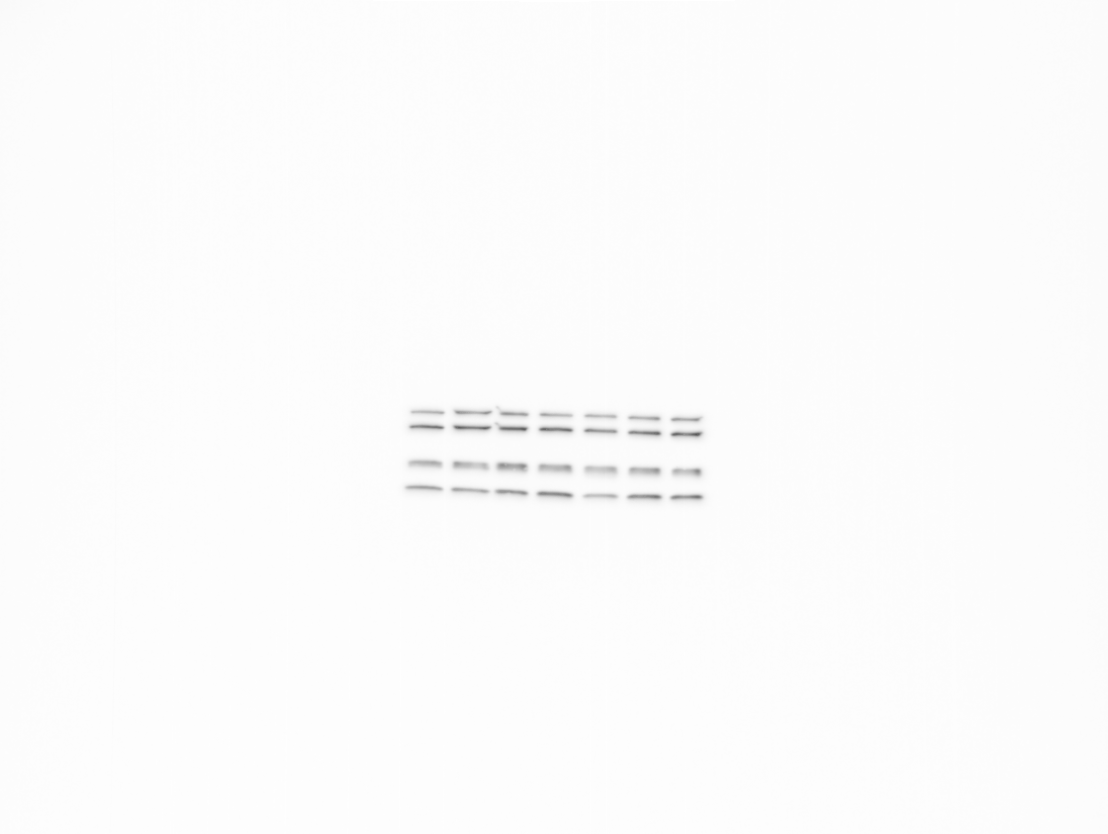

Supplement: Figure 4—source data 2. [file elife-100747-fig4-data2.zip › Figure 4 - Source Data 2 (original western files)/oxphos/S3F6-1118-155741.tif]

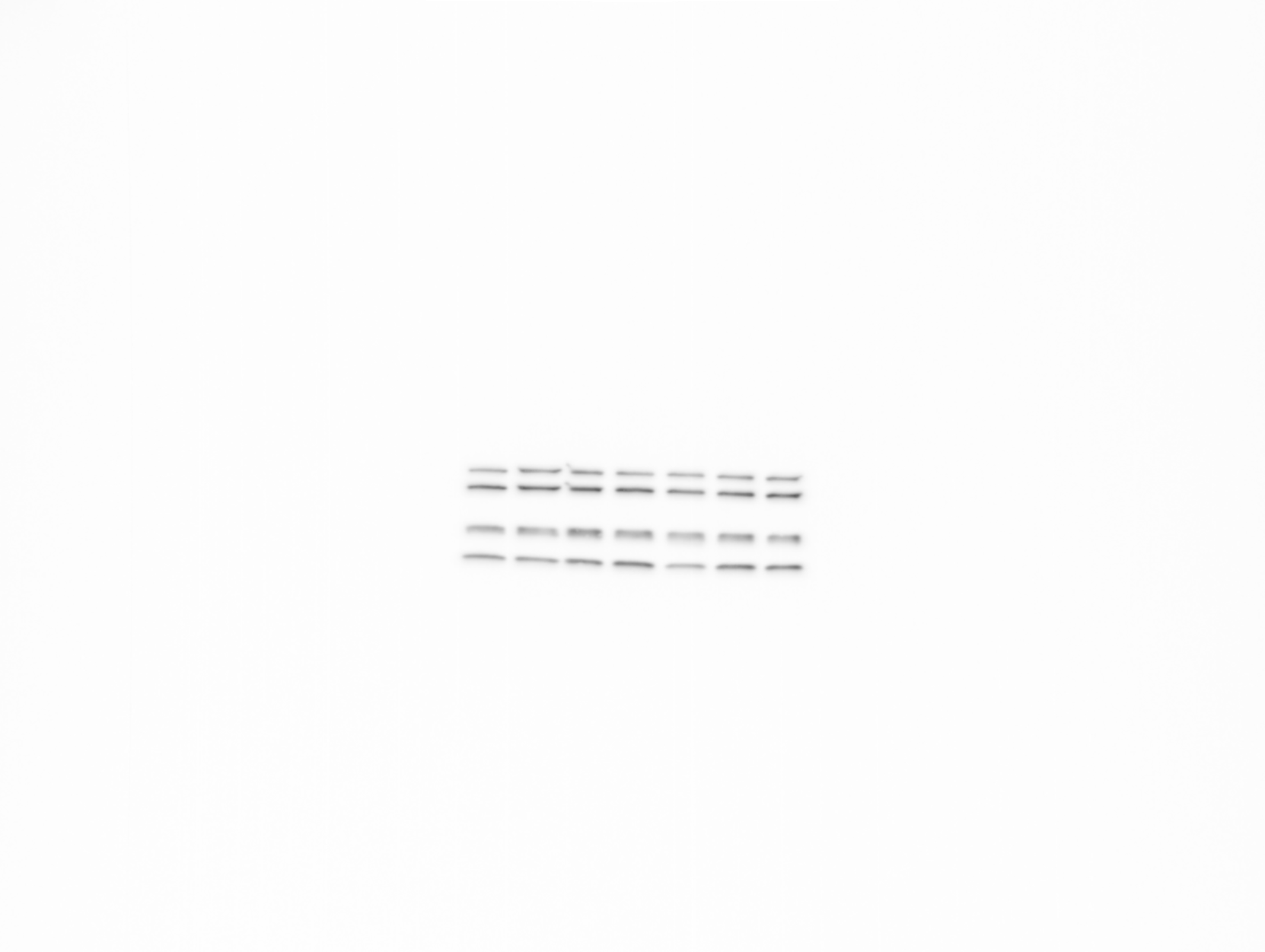

Supplement: Figure 4—source data 2. [file elife-100747-fig4-data2.zip › Figure 4 - Source Data 2 (original western files)/oxphos/S3F6-1118-155741_pub.tif]

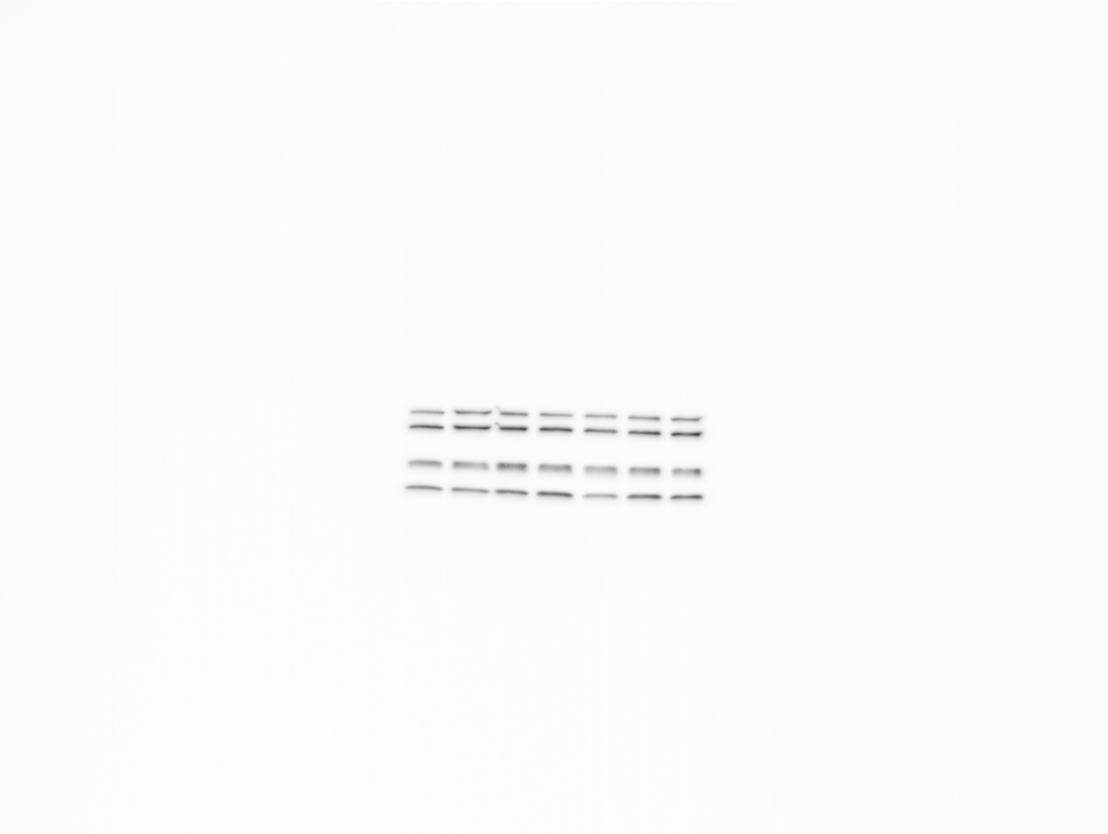

Supplement: Figure 4—source data 2. [file elife-100747-fig4-data2.zip › Figure 4 - Source Data 2 (original western files)/oxphos/S3F7-1118-155743.tif]

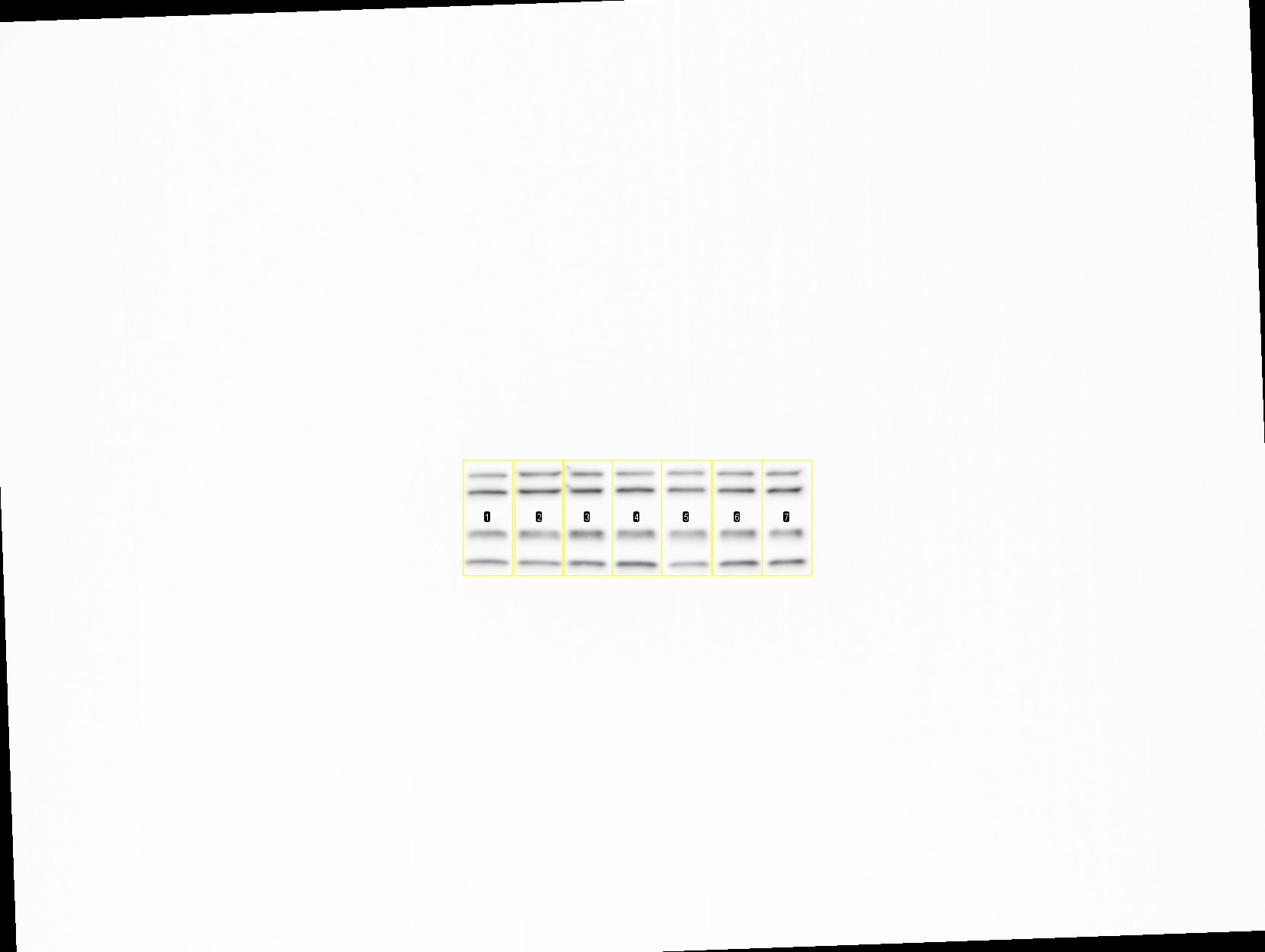

Supplement: Figure 4—source data 2. [file elife-100747-fig4-data2.zip › Figure 4 - Source Data 2 (original western files)/oxphos/S3F7-1118-155743_pub.jpg]

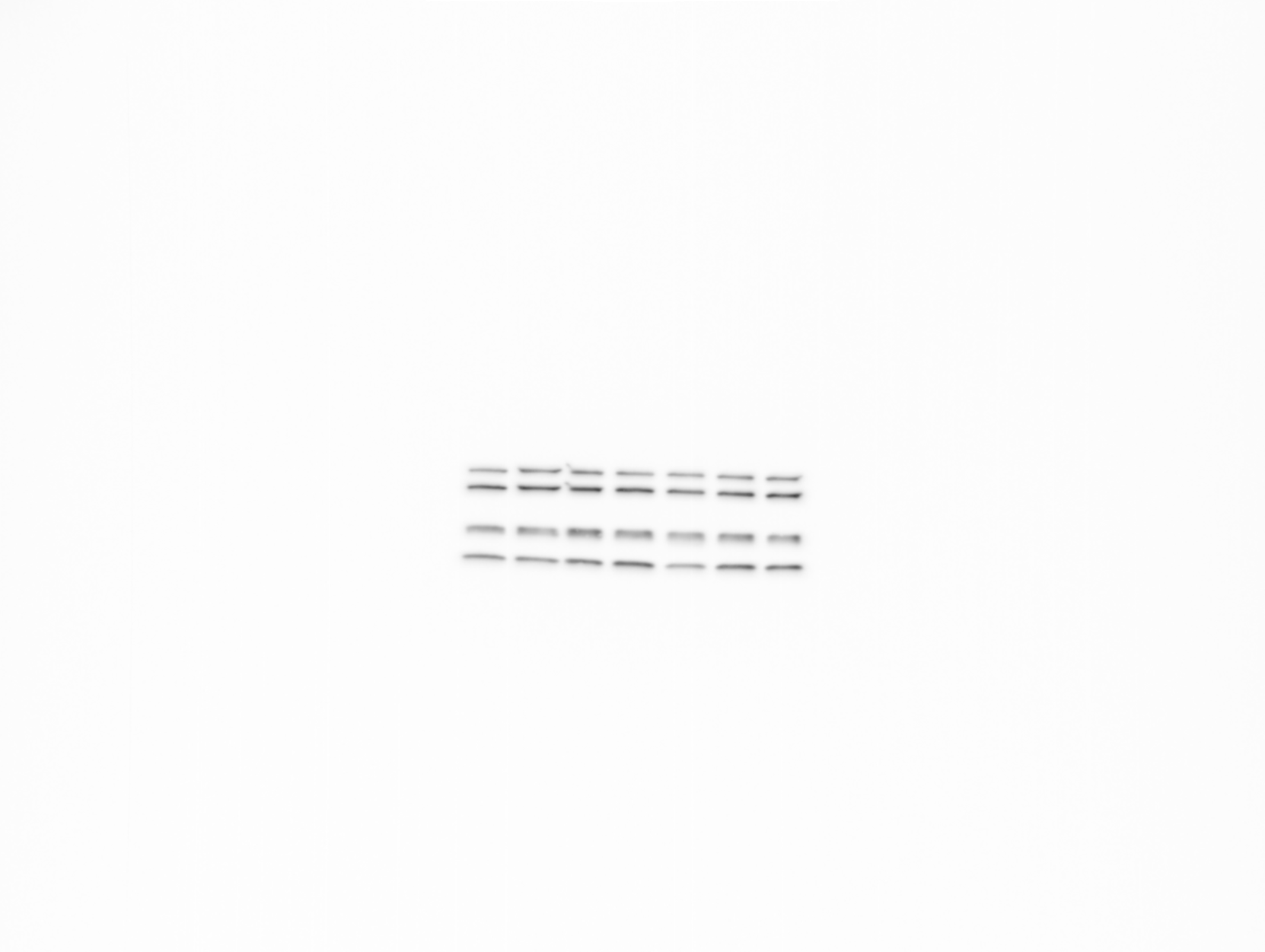

Supplement: Figure 4—source data 2. [file elife-100747-fig4-data2.zip › Figure 4 - Source Data 2 (original western files)/oxphos/S3F7-1118-155743_pub.tif]

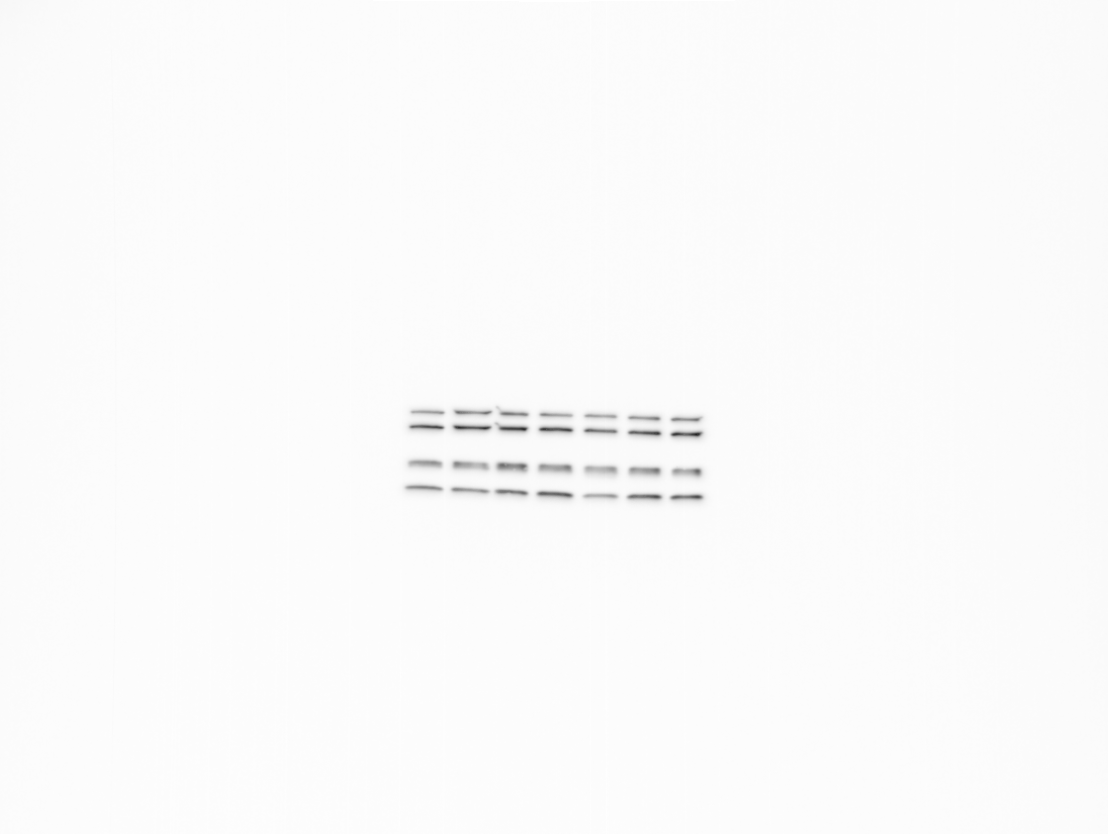

Supplement: Figure 4—source data 2. [file elife-100747-fig4-data2.zip › Figure 4 - Source Data 2 (original western files)/oxphos/S3F8-1118-155745.tif]

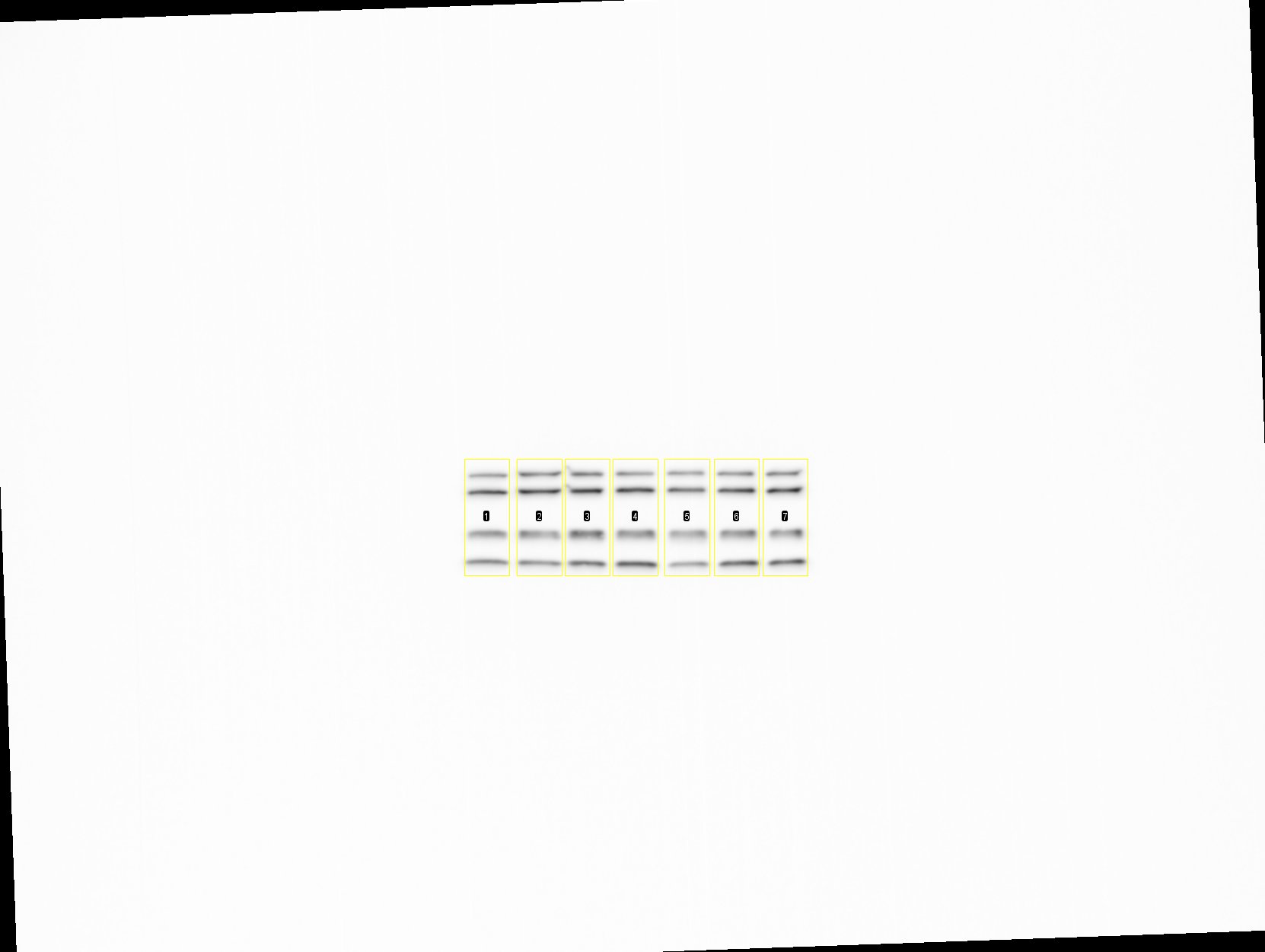

Supplement: Figure 4—source data 2. [file elife-100747-fig4-data2.zip › Figure 4 - Source Data 2 (original western files)/oxphos/S3F8-1118-155745_pub.jpg]

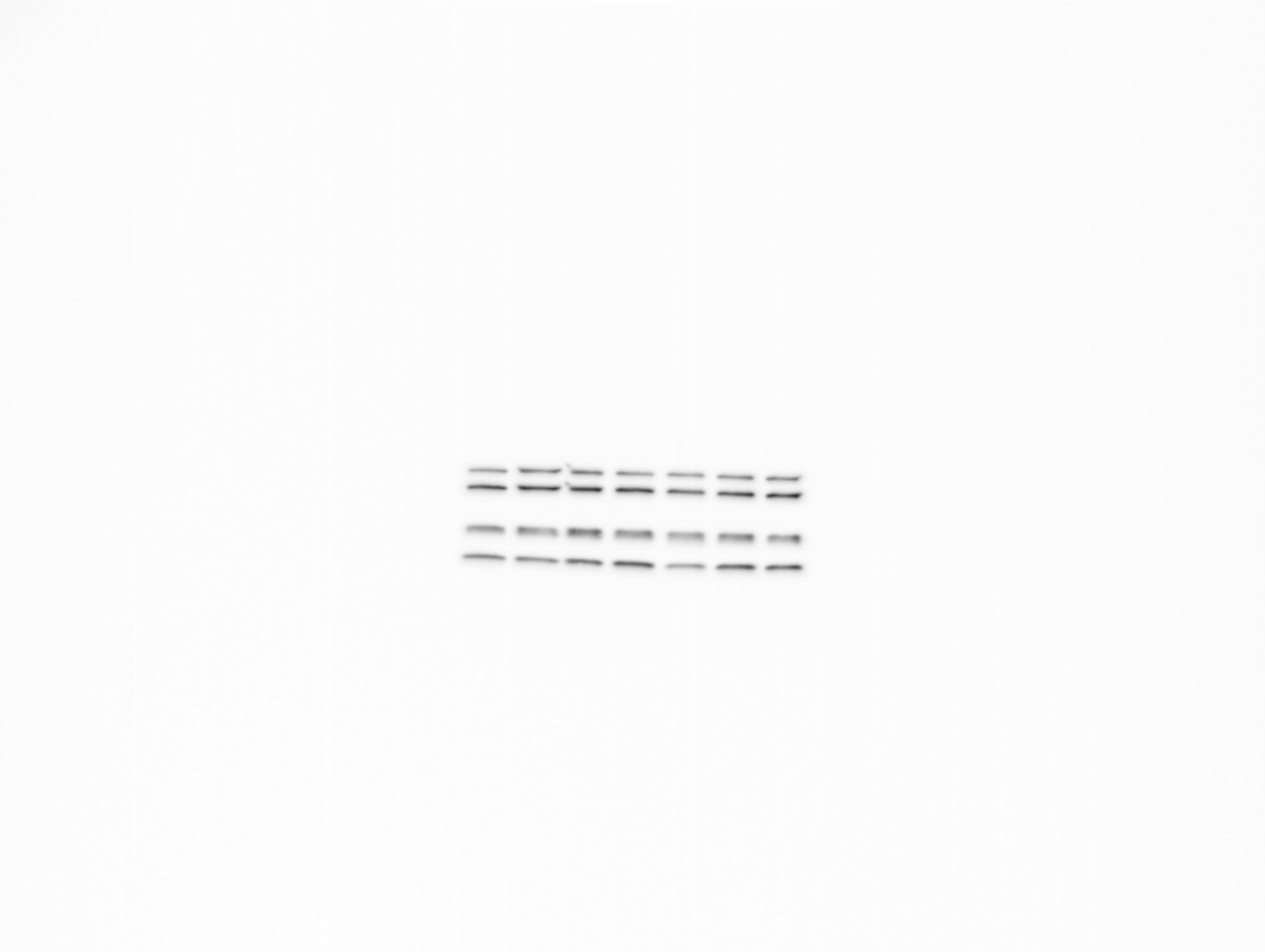

Supplement: Figure 4—source data 2. [file elife-100747-fig4-data2.zip › Figure 4 - Source Data 2 (original western files)/oxphos/S3F8-1118-155745_pub.tif]

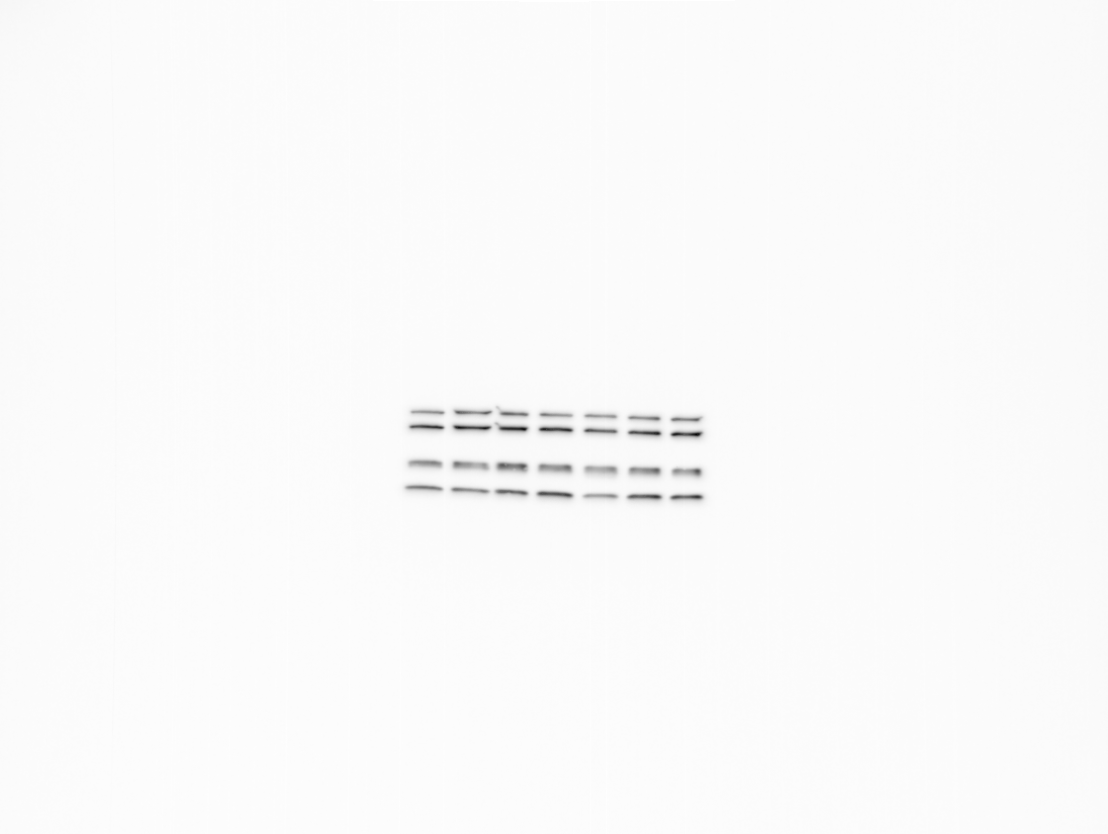

Supplement: Figure 4—source data 2. [file elife-100747-fig4-data2.zip › Figure 4 - Source Data 2 (original western files)/oxphos/S3F9-1118-155747.tif]

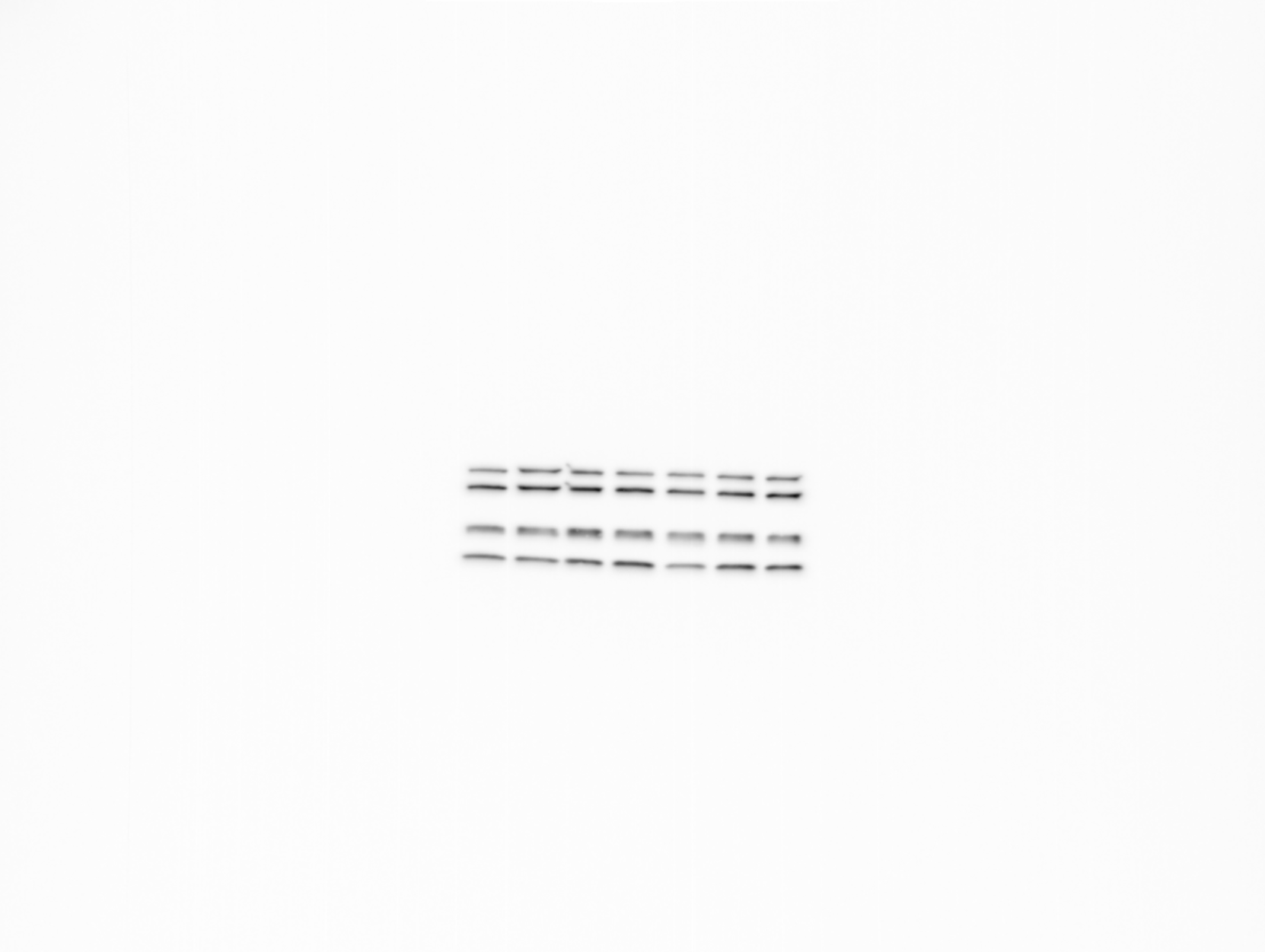

Supplement: Figure 4—source data 2. [file elife-100747-fig4-data2.zip › Figure 4 - Source Data 2 (original western files)/oxphos/S3F9-1118-155747_pub.tif]

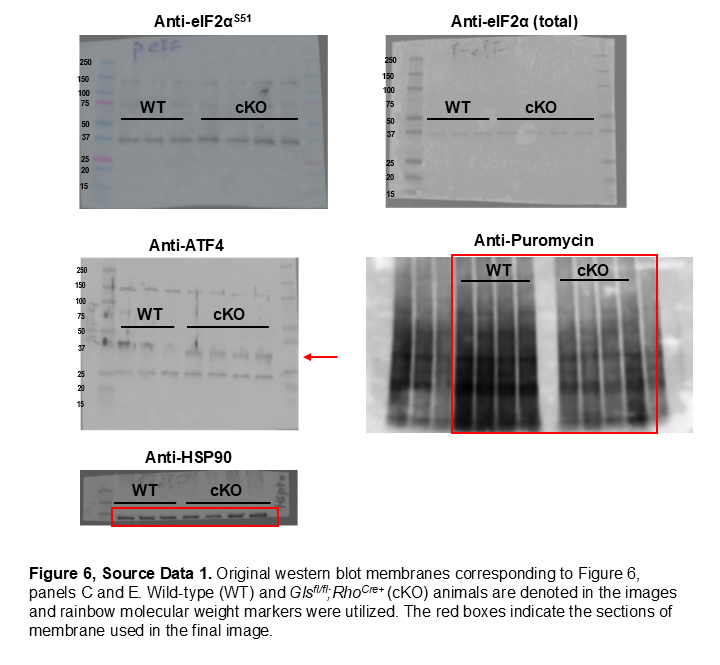

Supplement: Figure 6—source data 1. — Wild-type (WT) and Glsfl/fl;RhoCre+ (cKO) animals are denoted in the images and rainbow molecular weight markers were utilized. The red boxes indicate the sections of membrane used in the final image. [file elife-100747-fig6-data1.zip › Figure 6 - Source Data 1 (annotated western file)/Figure 6 - Source Data 1.tif]

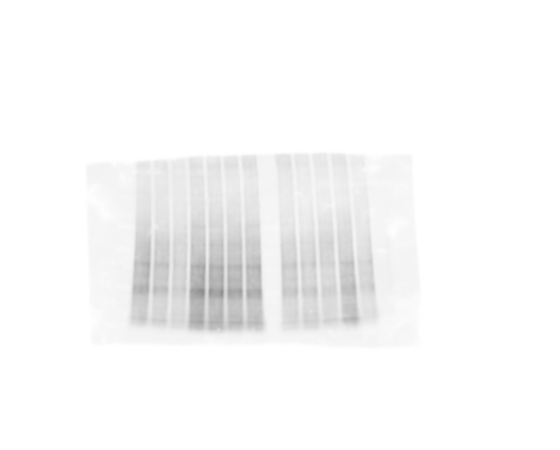

Supplement: Figure 6—source data 2. [file elife-100747-fig6-data2.zip › Figure 6 - Source Data 2 (original western files)/01312023_Wstn_Puro/23.01.31_13.00.36.tif]

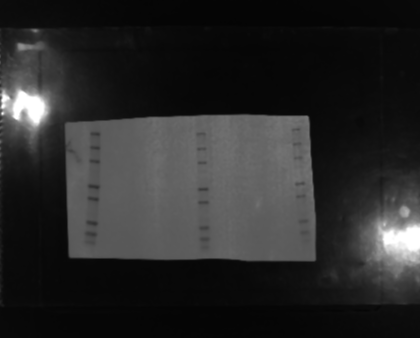

Supplement: Figure 6—source data 2. [file elife-100747-fig6-data2.zip › Figure 6 - Source Data 2 (original western files)/01312023_Wstn_Puro/23.01.31_13.01.56.tif]

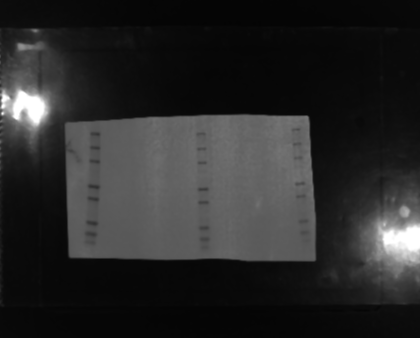

Supplement: Figure 6—source data 2. [file elife-100747-fig6-data2.zip › Figure 6 - Source Data 2 (original western files)/01312023_Wstn_Puro/23.01.31_13.02.35.tif]

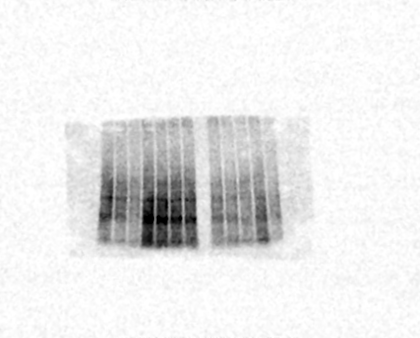

Supplement: Figure 6—source data 2. [file elife-100747-fig6-data2.zip › Figure 6 - Source Data 2 (original western files)/01312023_Wstn_Puro/23.01.31_13.04.20.tif]

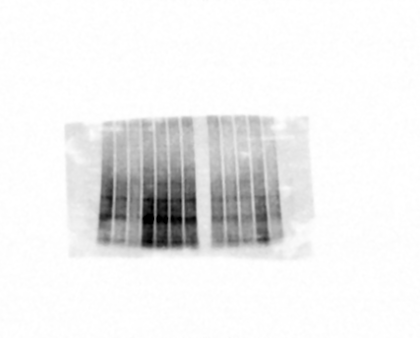

Supplement: Figure 6—source data 2. [file elife-100747-fig6-data2.zip › Figure 6 - Source Data 2 (original western files)/01312023_Wstn_Puro/23.01.31_13.04.51.tif]

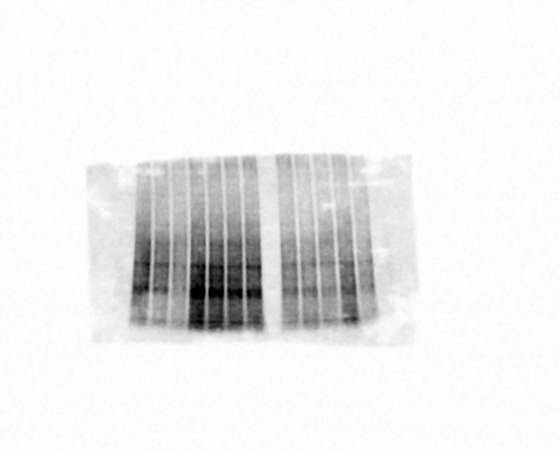

Supplement: Figure 6—source data 2. [file elife-100747-fig6-data2.zip › Figure 6 - Source Data 2 (original western files)/01312023_Wstn_Puro/23.01.31_13.05.03.tif]

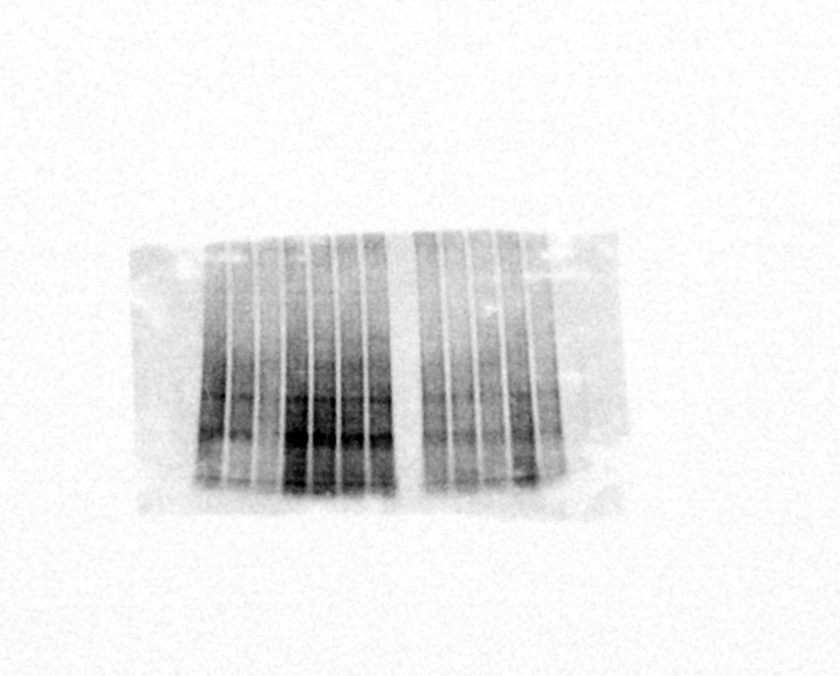

Supplement: Figure 6—source data 2. [file elife-100747-fig6-data2.zip › Figure 6 - Source Data 2 (original western files)/01312023_Wstn_Puro/23.01.31_13.05.17.tif]

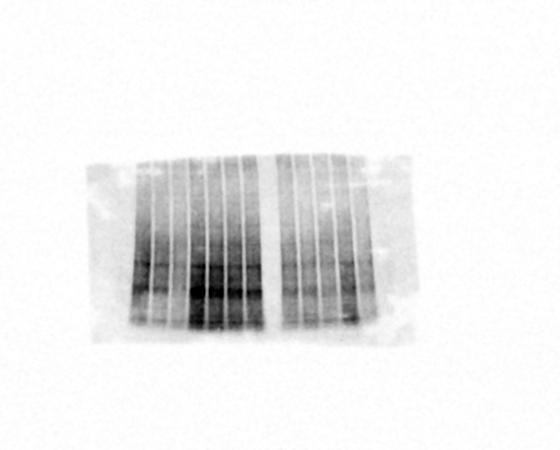

Supplement: Figure 6—source data 2. [file elife-100747-fig6-data2.zip › Figure 6 - Source Data 2 (original western files)/01312023_Wstn_Puro/23.01.31_13.05.26.tif]

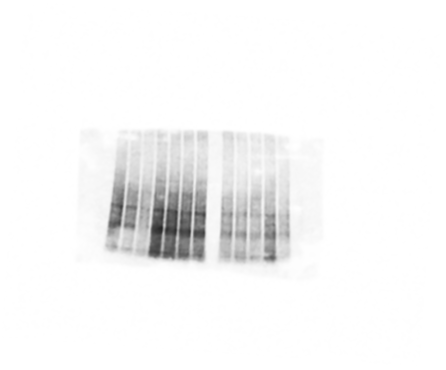

Supplement: Figure 6—source data 2. [file elife-100747-fig6-data2.zip › Figure 6 - Source Data 2 (original western files)/01312023_Wstn_Puro/23.01.31_13.05.37.tif]

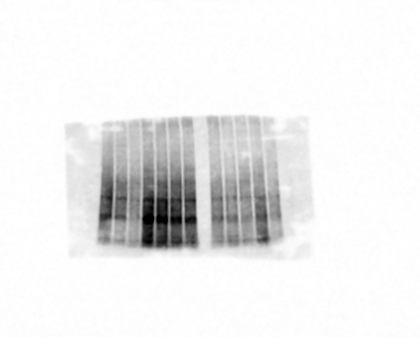

Supplement: Figure 6—source data 2. [file elife-100747-fig6-data2.zip › Figure 6 - Source Data 2 (original western files)/01312023_Wstn_Puro/23.01.31_13.05.47_S1_F01.tif]

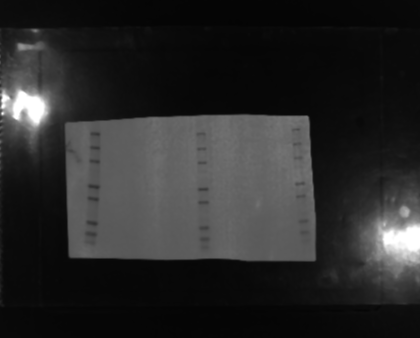

Supplement: Figure 6—source data 2. [file elife-100747-fig6-data2.zip › Figure 6 - Source Data 2 (original western files)/01312023_Wstn_Puro/23.01.31_13.05.47_S1_F02+Marker.tif]

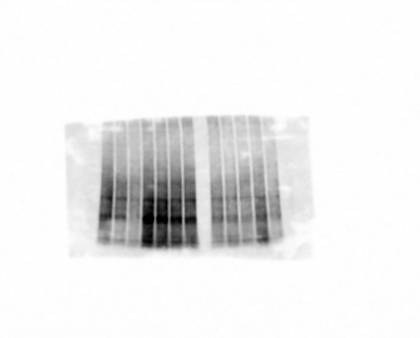

Supplement: Figure 6—source data 2. [file elife-100747-fig6-data2.zip › Figure 6 - Source Data 2 (original western files)/01312023_Wstn_Puro/23.01.31_13.05.47_S1_F02.tif]

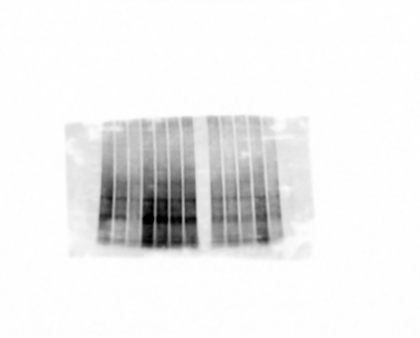

Supplement: Figure 6—source data 2. [file elife-100747-fig6-data2.zip › Figure 6 - Source Data 2 (original western files)/01312023_Wstn_Puro/23.01.31_13.05.47_S1_F03.tif]

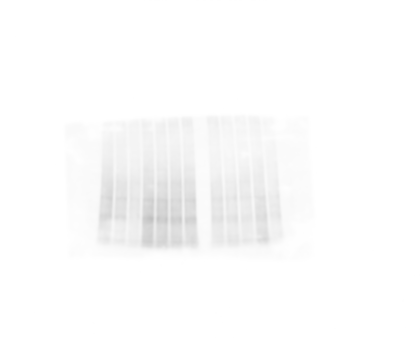

Supplement: Figure 6—source data 2. [file elife-100747-fig6-data2.zip › Figure 6 - Source Data 2 (original western files)/01312023_Wstn_Puro/23.01.31_13.05.47_S1_F04.tif]

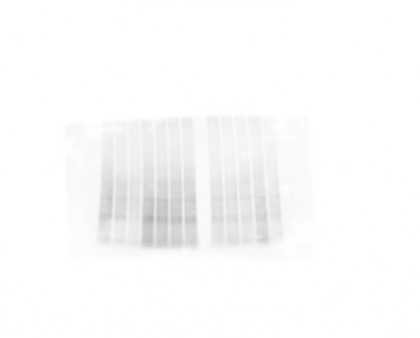

Supplement: Figure 6—source data 2. [file elife-100747-fig6-data2.zip › Figure 6 - Source Data 2 (original western files)/01312023_Wstn_Puro/23.01.31_13.05.47_S1_F05.tif]

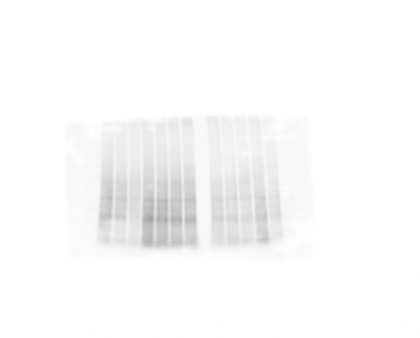

Supplement: Figure 6—source data 2. [file elife-100747-fig6-data2.zip › Figure 6 - Source Data 2 (original western files)/01312023_Wstn_Puro/23.01.31_13.05.47_S1_F06.tif]

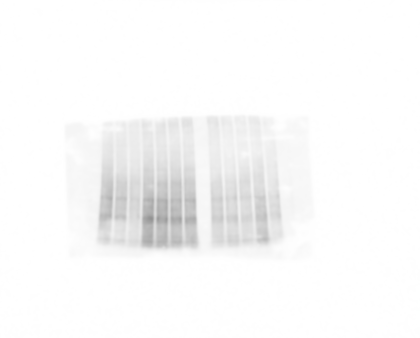

Supplement: Figure 6—source data 2. [file elife-100747-fig6-data2.zip › Figure 6 - Source Data 2 (original western files)/01312023_Wstn_Puro/23.01.31_13.05.47_S1_F07.tif]

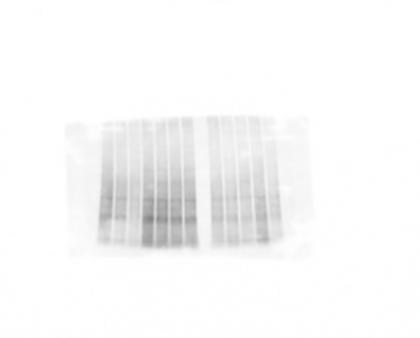

Supplement: Figure 6—source data 2. [file elife-100747-fig6-data2.zip › Figure 6 - Source Data 2 (original western files)/01312023_Wstn_Puro/23.01.31_13.05.47_S1_F08.tif]

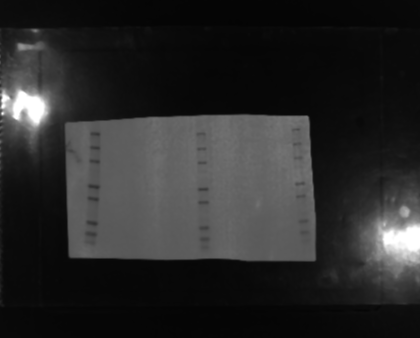

Supplement: Figure 6—source data 2. [file elife-100747-fig6-data2.zip › Figure 6 - Source Data 2 (original western files)/01312023_Wstn_Puro/23.01.31_13.05.47_S1_F09+Marker.tif]

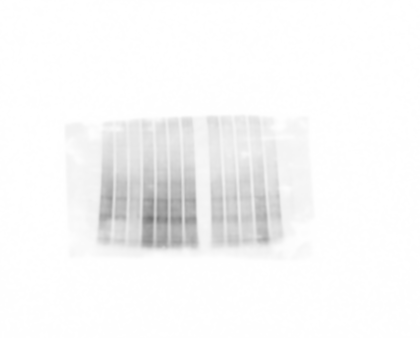

Supplement: Figure 6—source data 2. [file elife-100747-fig6-data2.zip › Figure 6 - Source Data 2 (original western files)/01312023_Wstn_Puro/23.01.31_13.05.47_S1_F09.tif]

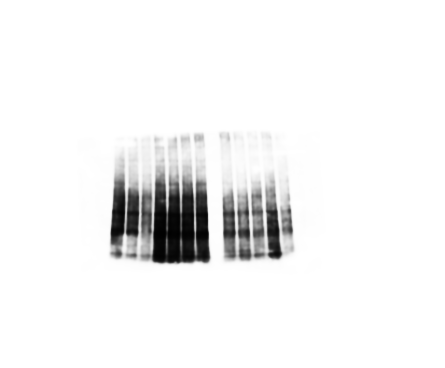

Supplement: Figure 6—source data 2. [file elife-100747-fig6-data2.zip › Figure 6 - Source Data 2 (original western files)/01312023_Wstn_Puro/23.01.31_13.05.47_S1_F10.tif]

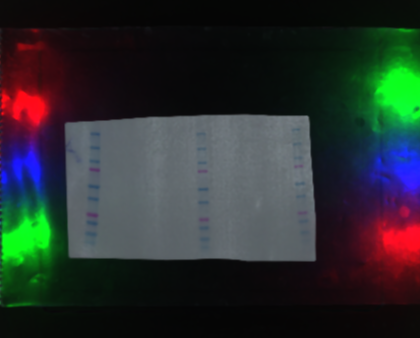

Supplement: Figure 6—source data 2. [file elife-100747-fig6-data2.zip › Figure 6 - Source Data 2 (original western files)/01312023_Wstn_Puro/23.01.31_13.05.47_S1_marker.tif]

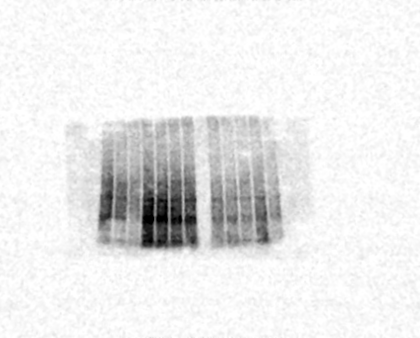

Supplement: Figure 6—source data 2. [file elife-100747-fig6-data2.zip › Figure 6 - Source Data 2 (original western files)/01312023_Wstn_Puro/23.01.31_13.07.51_S2_F01.tif]

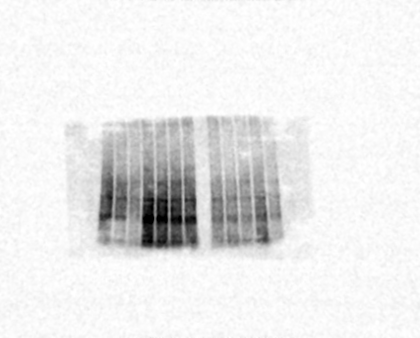

Supplement: Figure 6—source data 2. [file elife-100747-fig6-data2.zip › Figure 6 - Source Data 2 (original western files)/01312023_Wstn_Puro/23.01.31_13.07.51_S2_F02.tif]

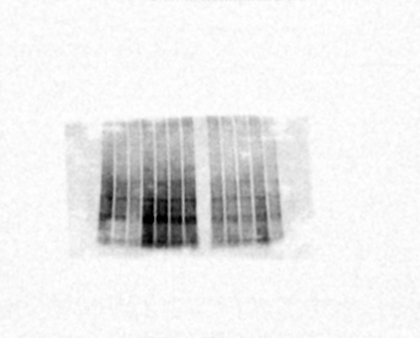

Supplement: Figure 6—source data 2. [file elife-100747-fig6-data2.zip › Figure 6 - Source Data 2 (original western files)/01312023_Wstn_Puro/23.01.31_13.07.51_S2_F03.tif]

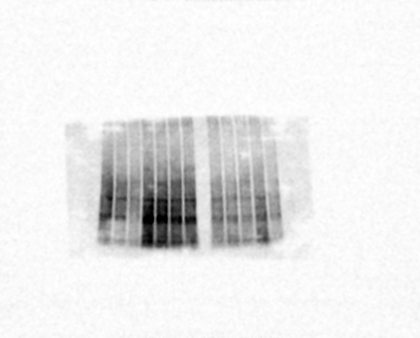

Supplement: Figure 6—source data 2. [file elife-100747-fig6-data2.zip › Figure 6 - Source Data 2 (original western files)/01312023_Wstn_Puro/23.01.31_13.07.51_S2_F04.tif]

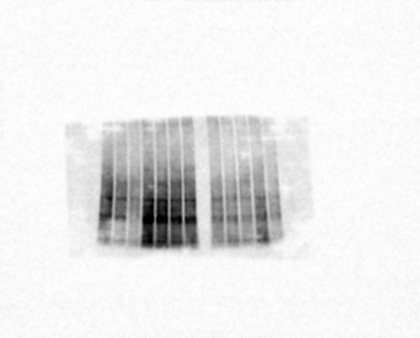

Supplement: Figure 6—source data 2. [file elife-100747-fig6-data2.zip › Figure 6 - Source Data 2 (original western files)/01312023_Wstn_Puro/23.01.31_13.07.51_S2_F05.tif]

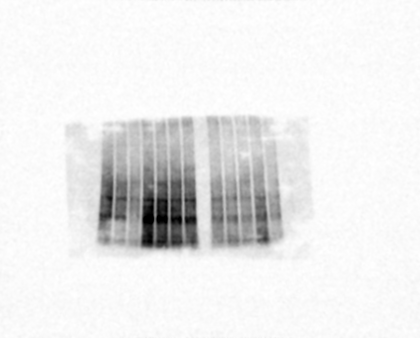

Supplement: Figure 6—source data 2. [file elife-100747-fig6-data2.zip › Figure 6 - Source Data 2 (original western files)/01312023_Wstn_Puro/23.01.31_13.07.51_S2_F06.tif]

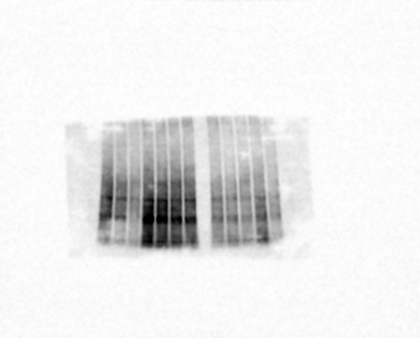

Supplement: Figure 6—source data 2. [file elife-100747-fig6-data2.zip › Figure 6 - Source Data 2 (original western files)/01312023_Wstn_Puro/23.01.31_13.07.51_S2_F07.tif]

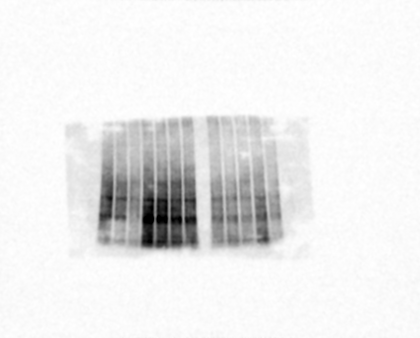

Supplement: Figure 6—source data 2. [file elife-100747-fig6-data2.zip › Figure 6 - Source Data 2 (original western files)/01312023_Wstn_Puro/23.01.31_13.07.51_S2_F08.tif]

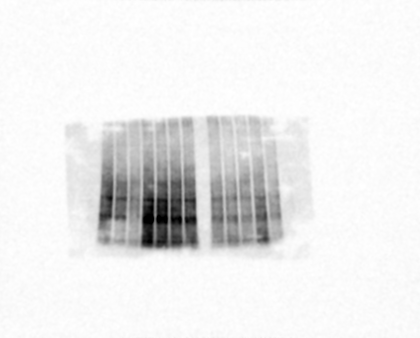

Supplement: Figure 6—source data 2. [file elife-100747-fig6-data2.zip › Figure 6 - Source Data 2 (original western files)/01312023_Wstn_Puro/23.01.31_13.07.51_S2_F09.tif]

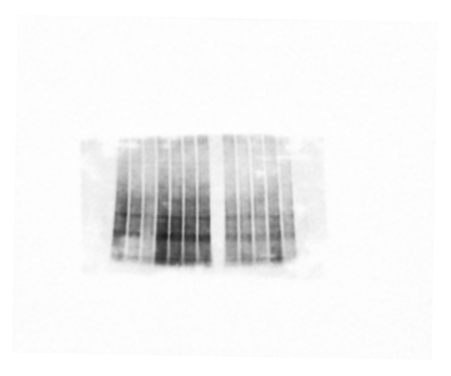

Supplement: Figure 6—source data 2. [file elife-100747-fig6-data2.zip › Figure 6 - Source Data 2 (original western files)/01312023_Wstn_Puro/23.01.31_13.07.51_S2_F10.tif]

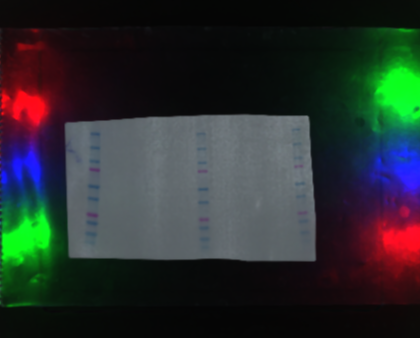

Supplement: Figure 6—source data 2. [file elife-100747-fig6-data2.zip › Figure 6 - Source Data 2 (original western files)/01312023_Wstn_Puro/23.01.31_13.07.51_S2_marker.tif]

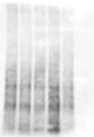

Supplement: Figure 6—source data 2. [file elife-100747-fig6-data2.zip › Figure 6 - Source Data 2 (original western files)/01312023_Wstn_Puro/KO quantitation.tif]

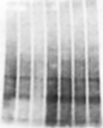

Supplement: Figure 6—source data 2. [file elife-100747-fig6-data2.zip › Figure 6 - Source Data 2 (original western files)/01312023_Wstn_Puro/WT.tif]

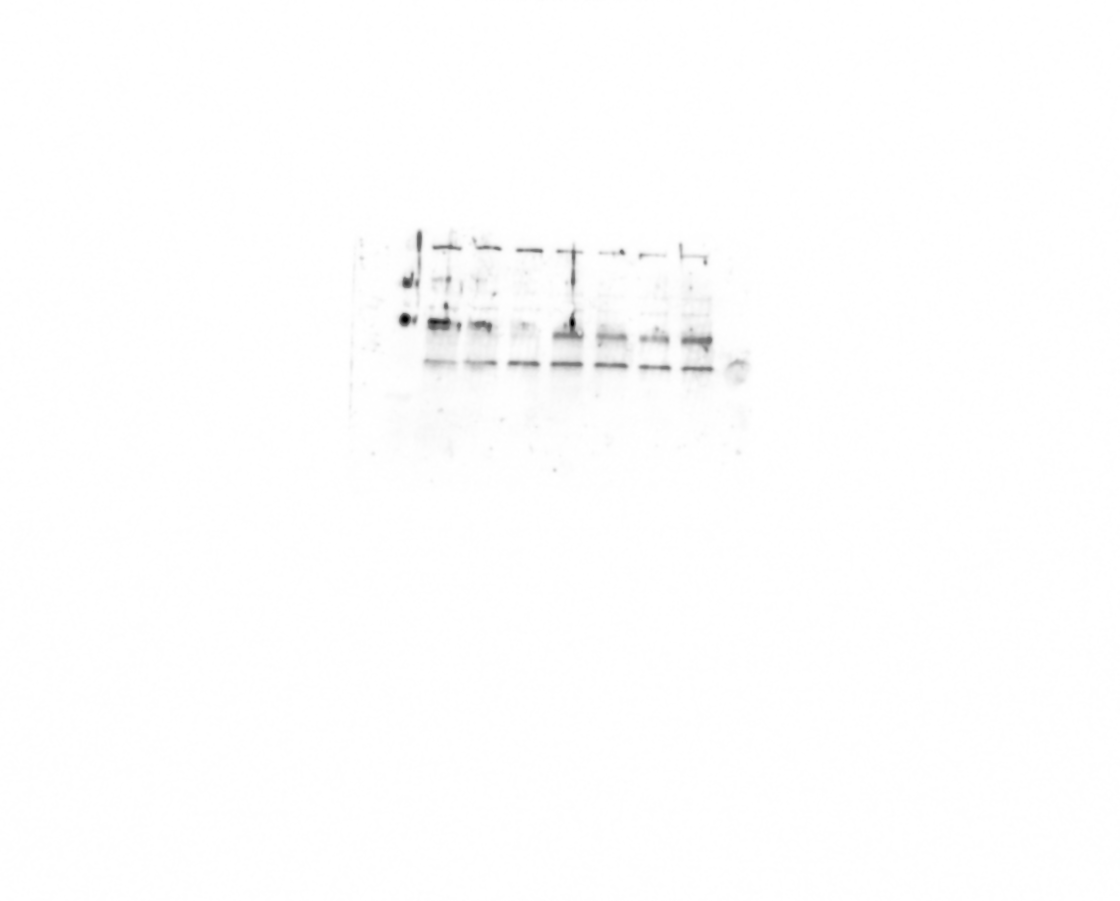

Supplement: Figure 6—source data 2. [file elife-100747-fig6-data2.zip › Figure 6 - Source Data 2 (original western files)/ATF4-cell signaling/23.01.16_16.33.38.tif]

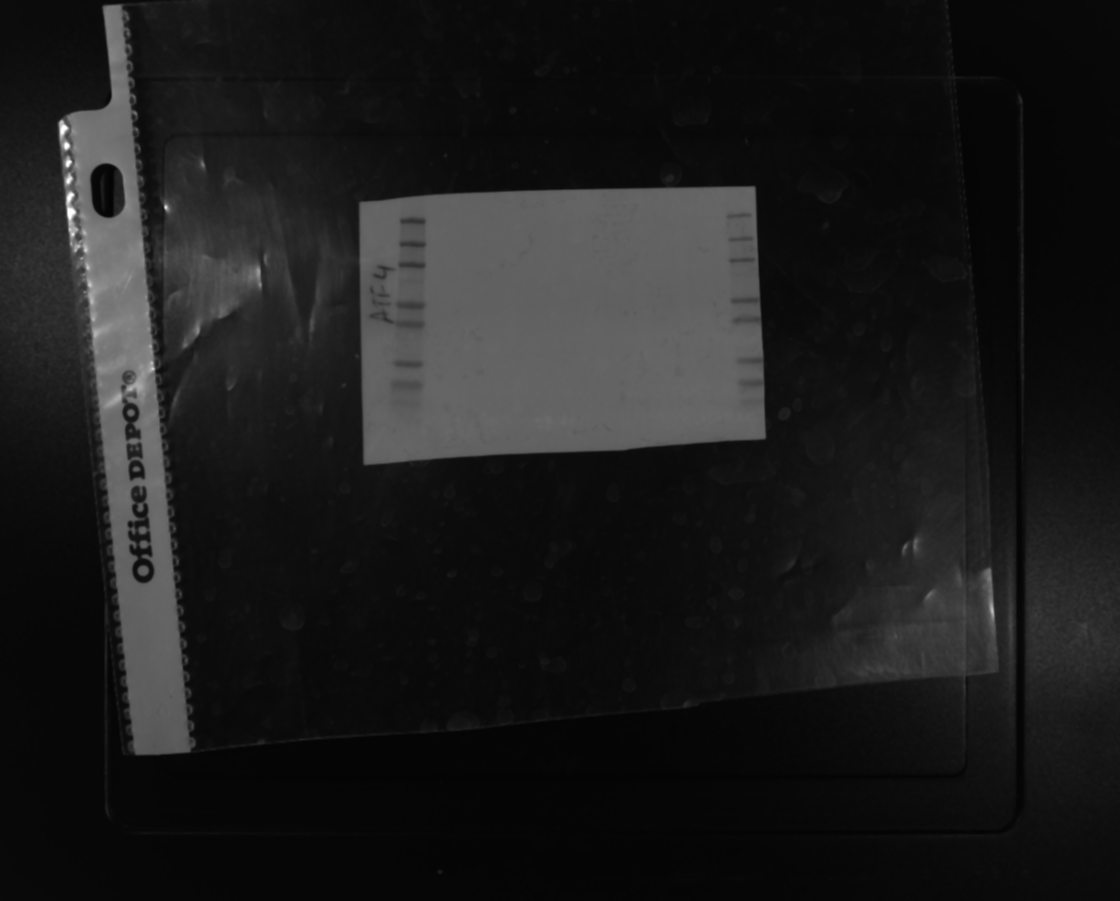

Supplement: Figure 6—source data 2. [file elife-100747-fig6-data2.zip › Figure 6 - Source Data 2 (original western files)/ATF4-cell signaling/23.01.16_16.34.26.tif]

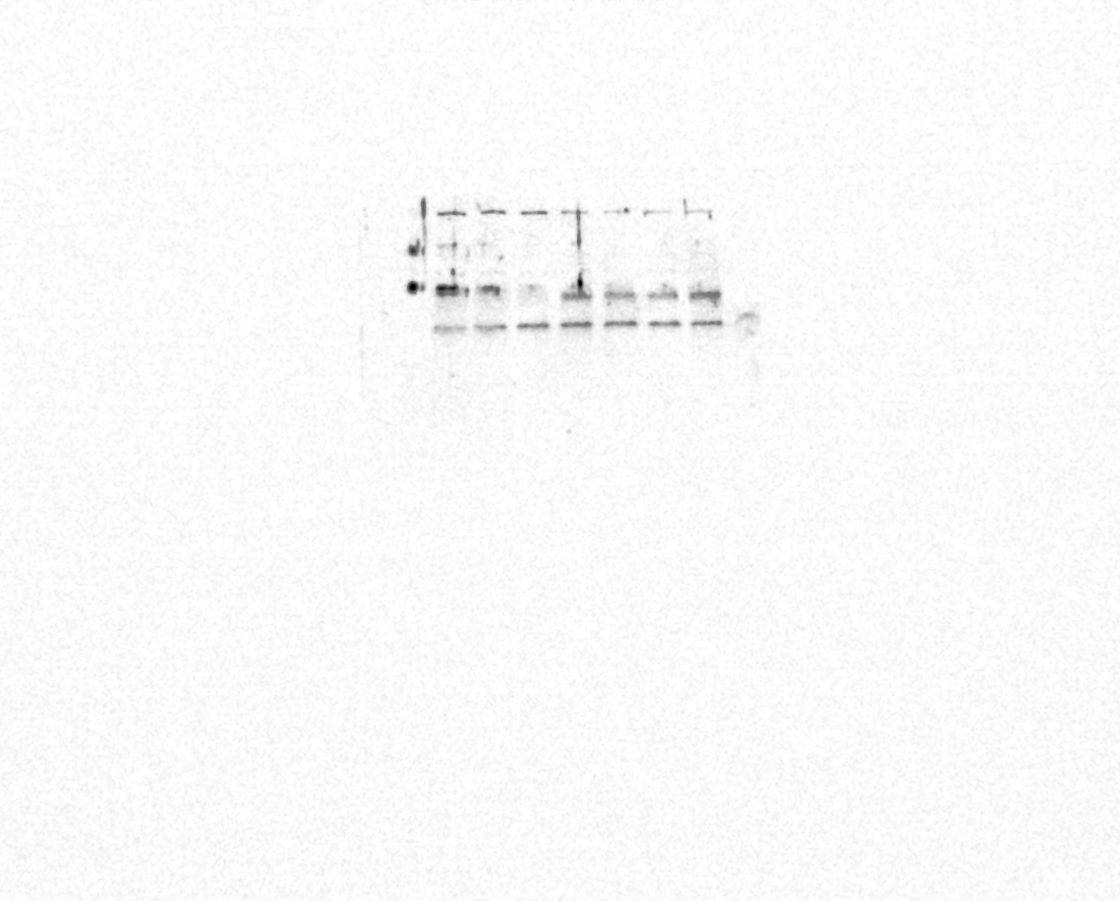

Supplement: Figure 6—source data 2. [file elife-100747-fig6-data2.zip › Figure 6 - Source Data 2 (original western files)/ATF4-cell signaling/23.01.16_16.35.38_S2_F01.tif]

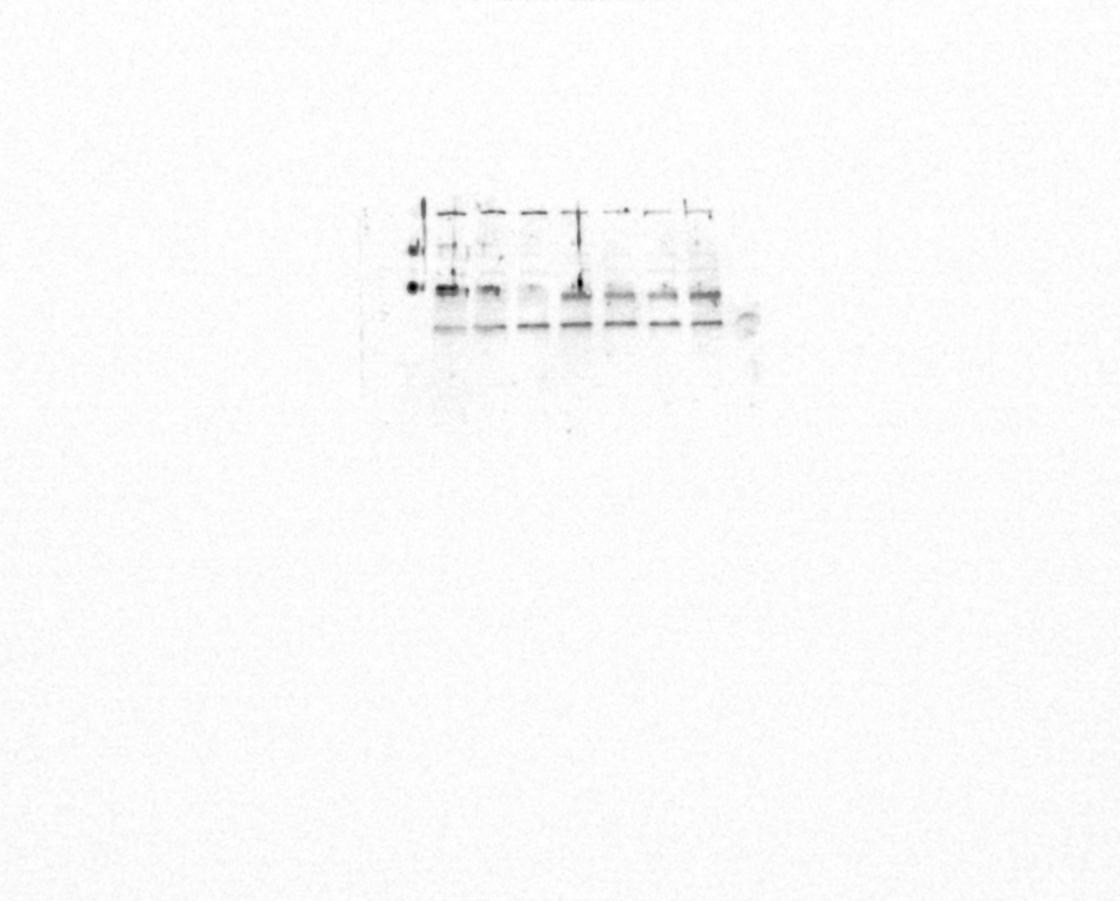

Supplement: Figure 6—source data 2. [file elife-100747-fig6-data2.zip › Figure 6 - Source Data 2 (original western files)/ATF4-cell signaling/23.01.16_16.35.38_S2_F02.tif]

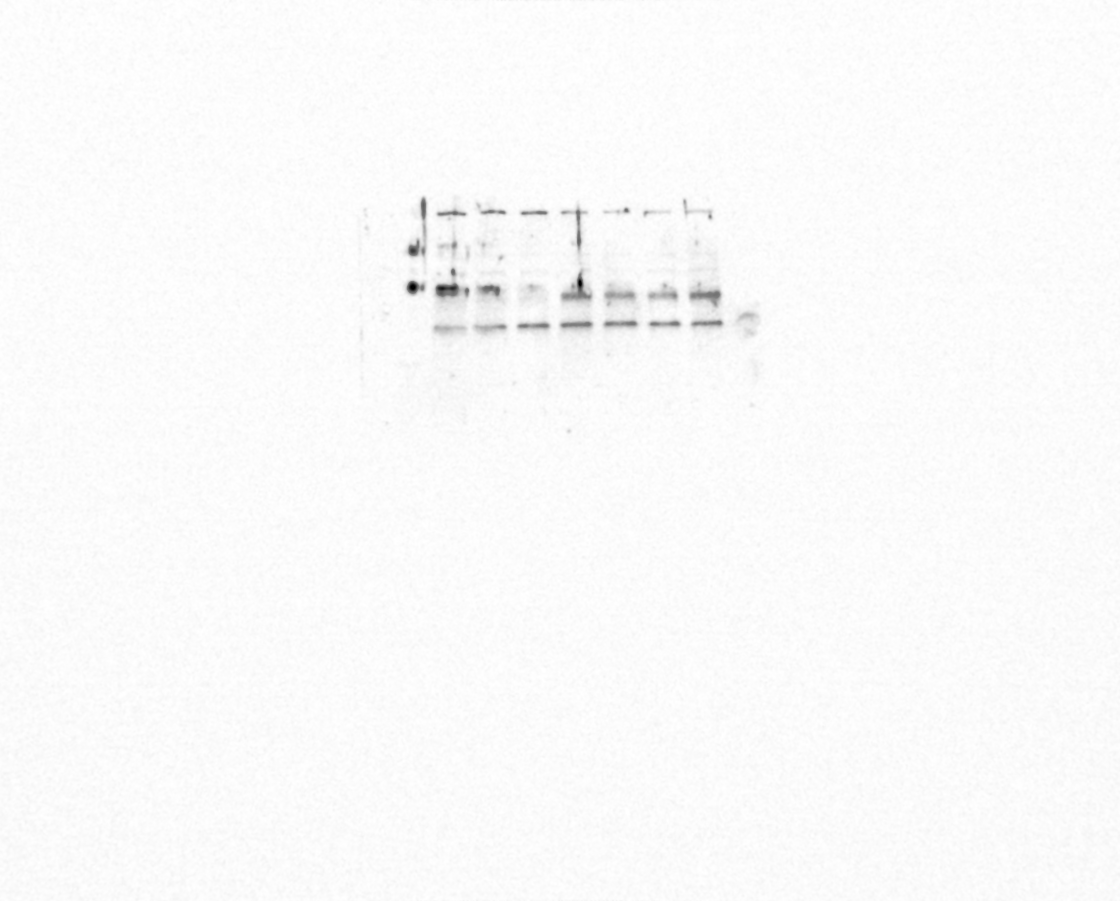

Supplement: Figure 6—source data 2. [file elife-100747-fig6-data2.zip › Figure 6 - Source Data 2 (original western files)/ATF4-cell signaling/23.01.16_16.35.38_S2_F03.tif]

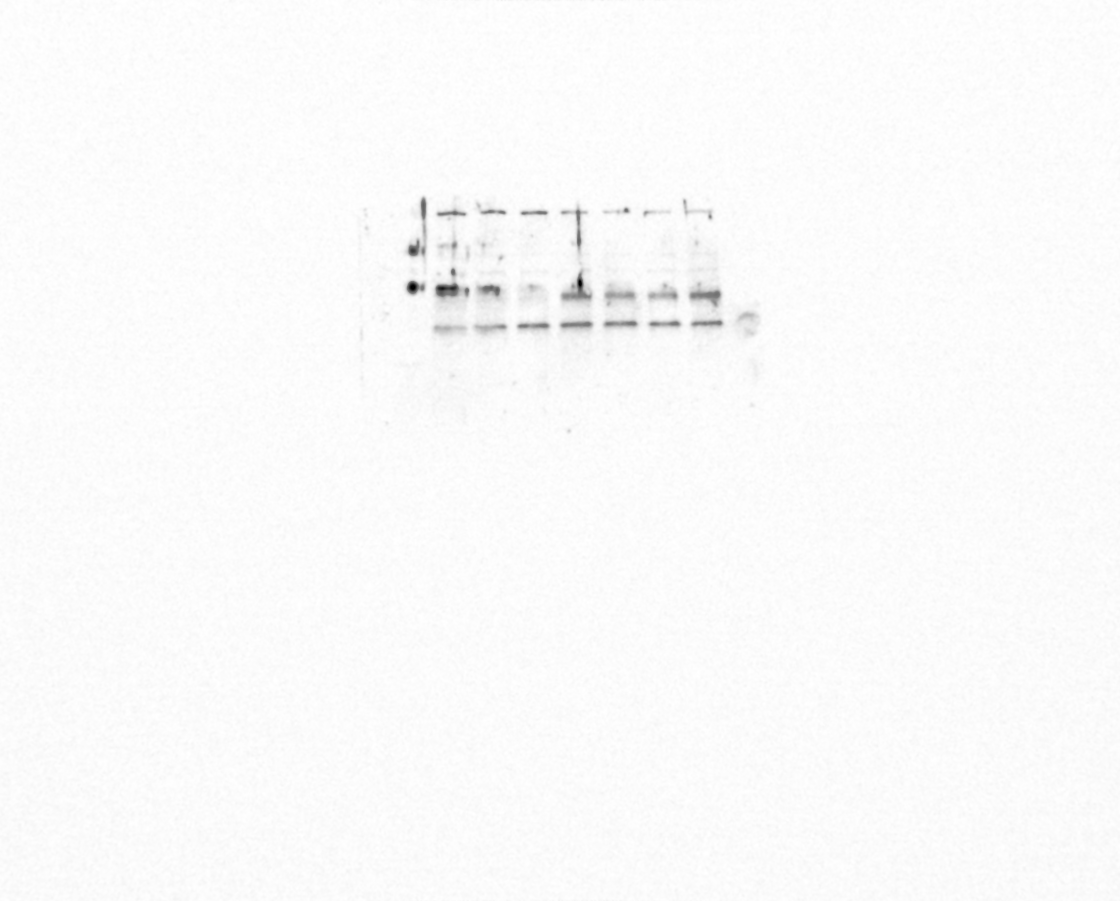

Supplement: Figure 6—source data 2. [file elife-100747-fig6-data2.zip › Figure 6 - Source Data 2 (original western files)/ATF4-cell signaling/23.01.16_16.35.38_S2_F04.tif]

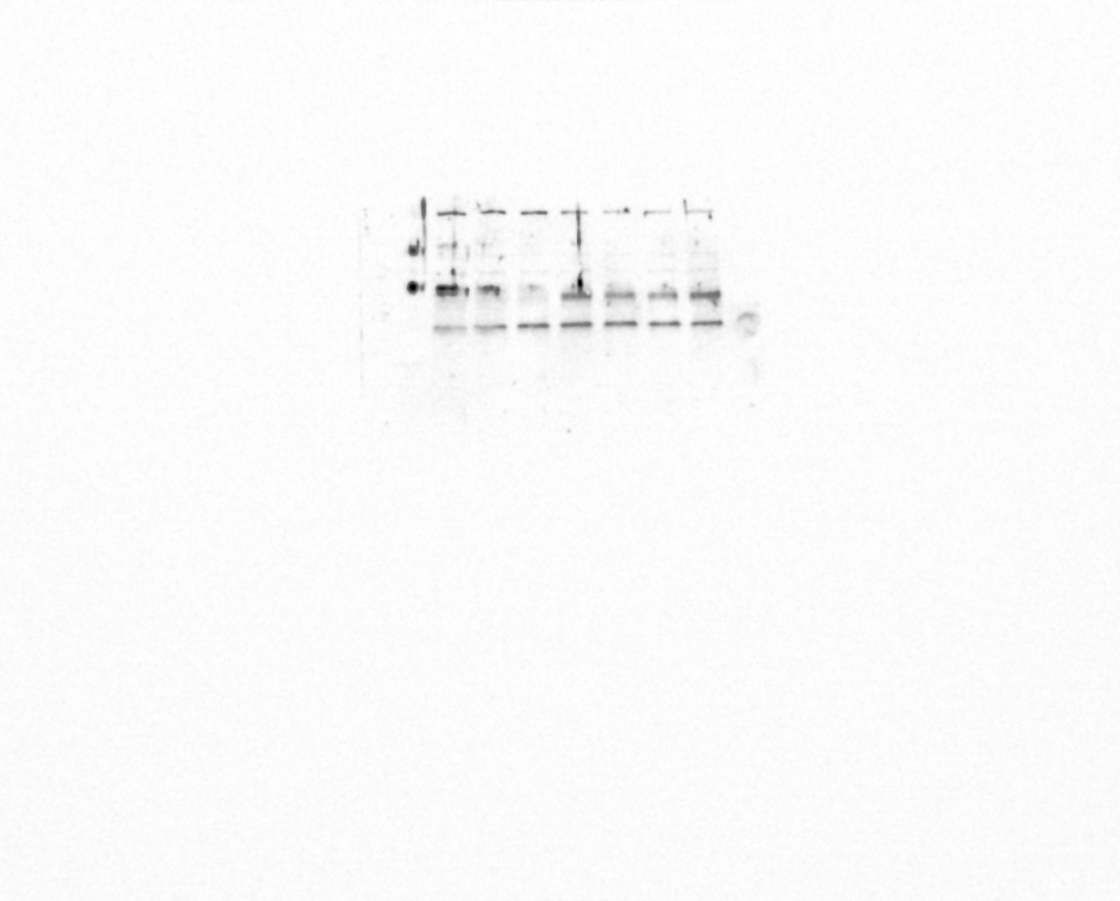

Supplement: Figure 6—source data 2. [file elife-100747-fig6-data2.zip › Figure 6 - Source Data 2 (original western files)/ATF4-cell signaling/23.01.16_16.35.38_S2_F05.tif]

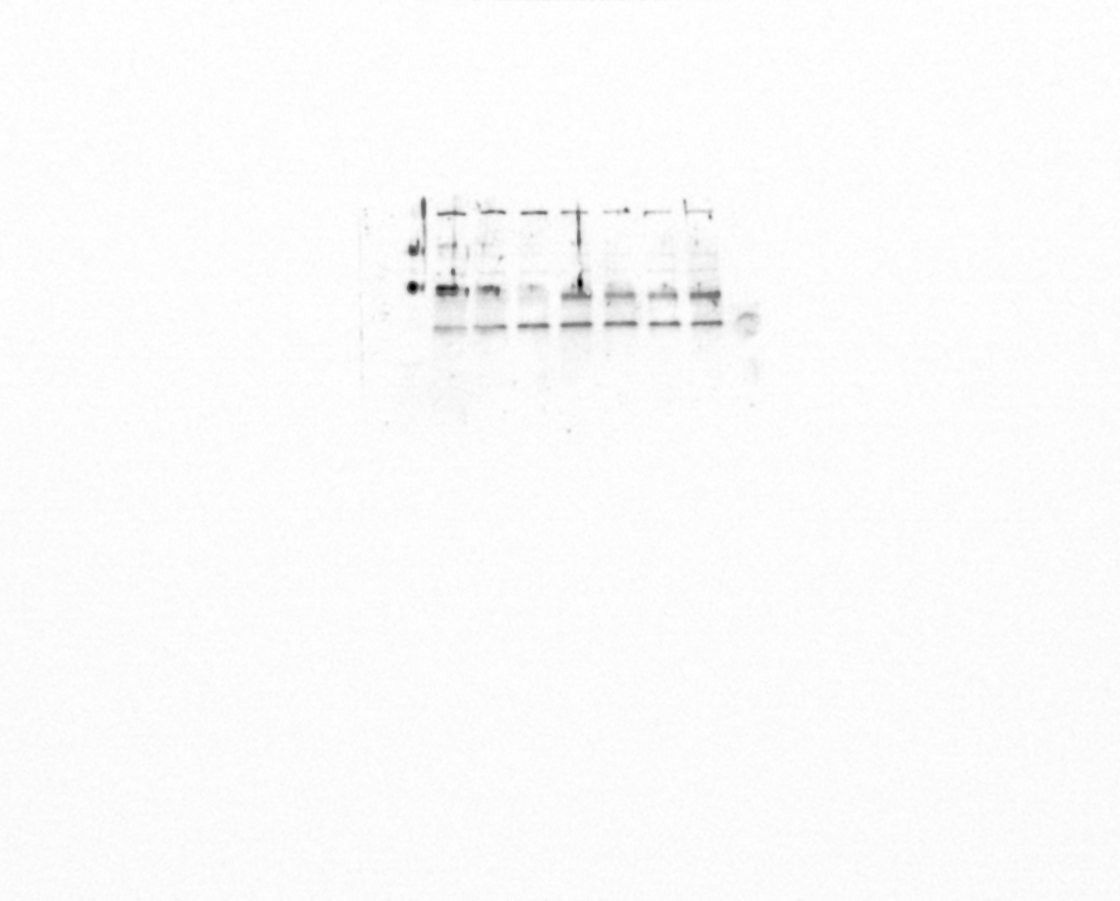

Supplement: Figure 6—source data 2. [file elife-100747-fig6-data2.zip › Figure 6 - Source Data 2 (original western files)/ATF4-cell signaling/23.01.16_16.35.38_S2_F06.tif]

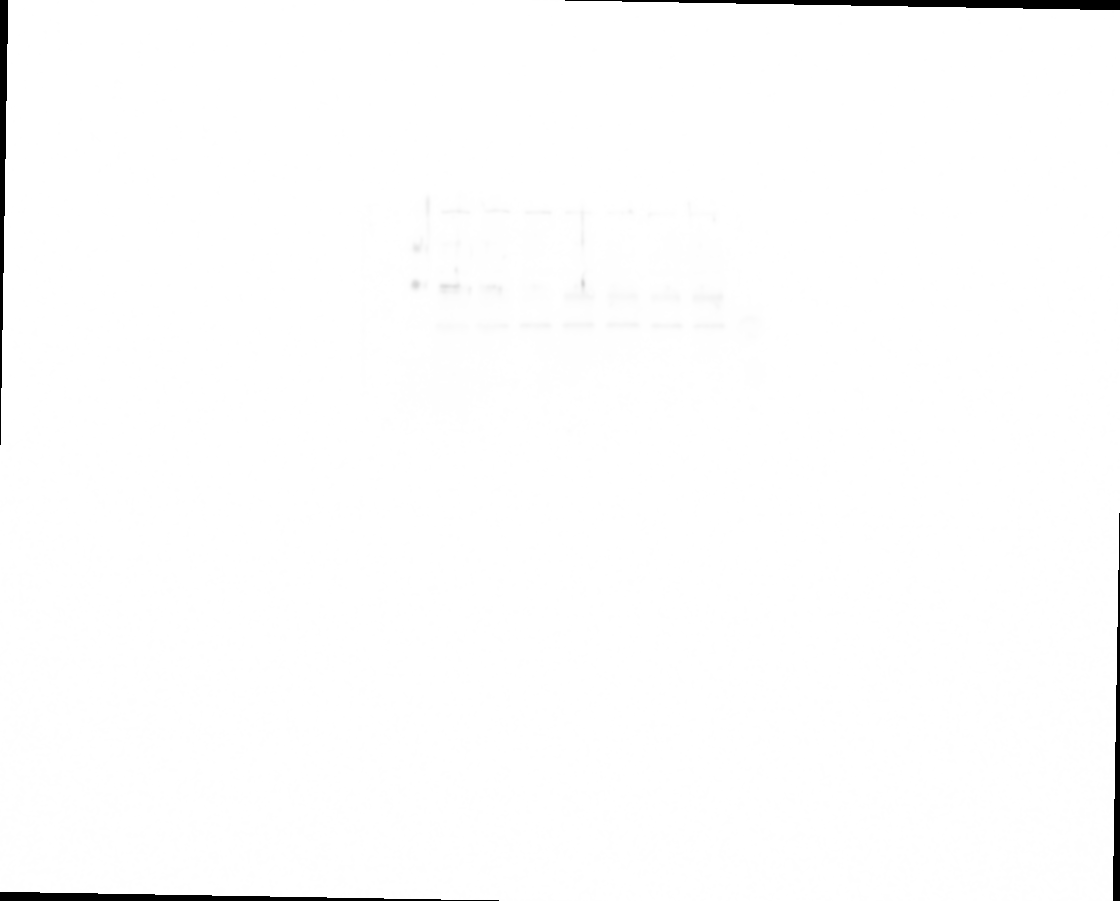

Supplement: Figure 6—source data 2. [file elife-100747-fig6-data2.zip › Figure 6 - Source Data 2 (original western files)/ATF4-cell signaling/23.01.16_16.35.38_S2_F07.tif]

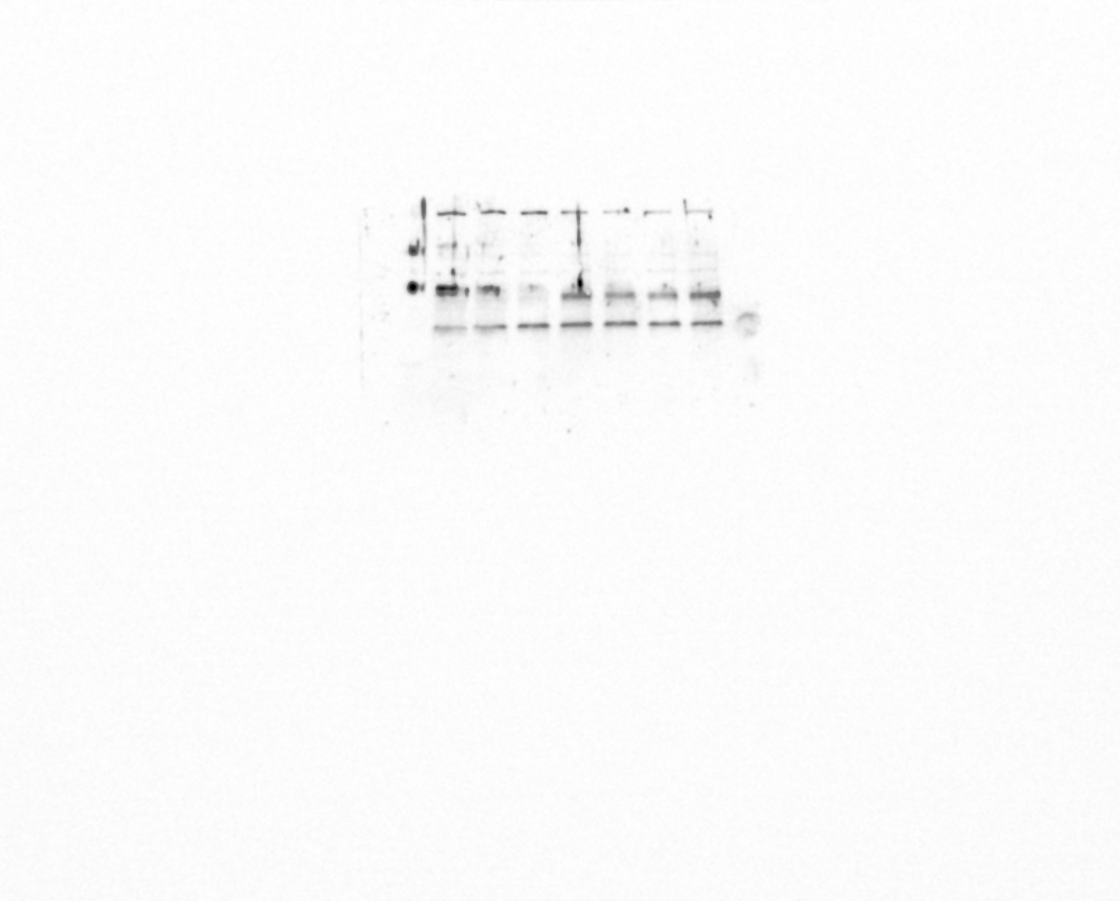

Supplement: Figure 6—source data 2. [file elife-100747-fig6-data2.zip › Figure 6 - Source Data 2 (original western files)/ATF4-cell signaling/23.01.16_16.35.38_S2_F08.tif]

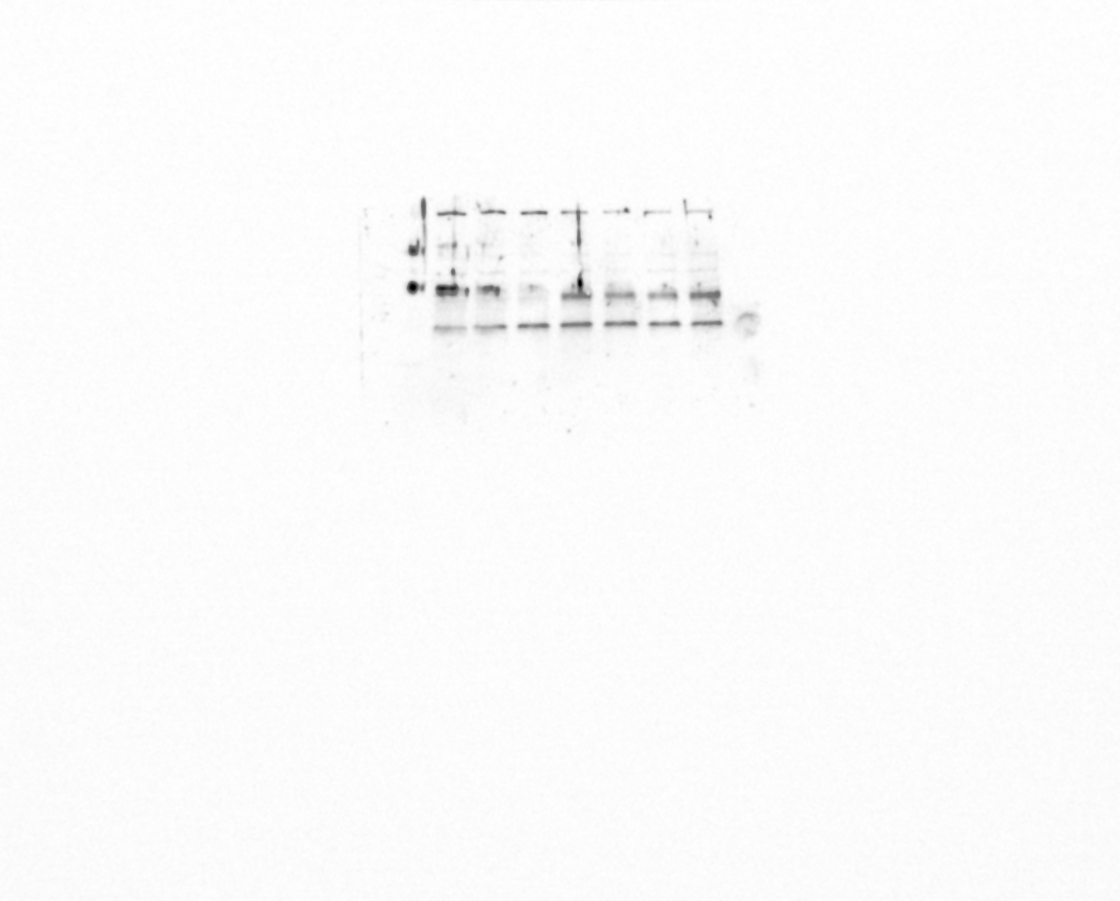

Supplement: Figure 6—source data 2. [file elife-100747-fig6-data2.zip › Figure 6 - Source Data 2 (original western files)/ATF4-cell signaling/23.01.16_16.35.38_S2_F09.tif]

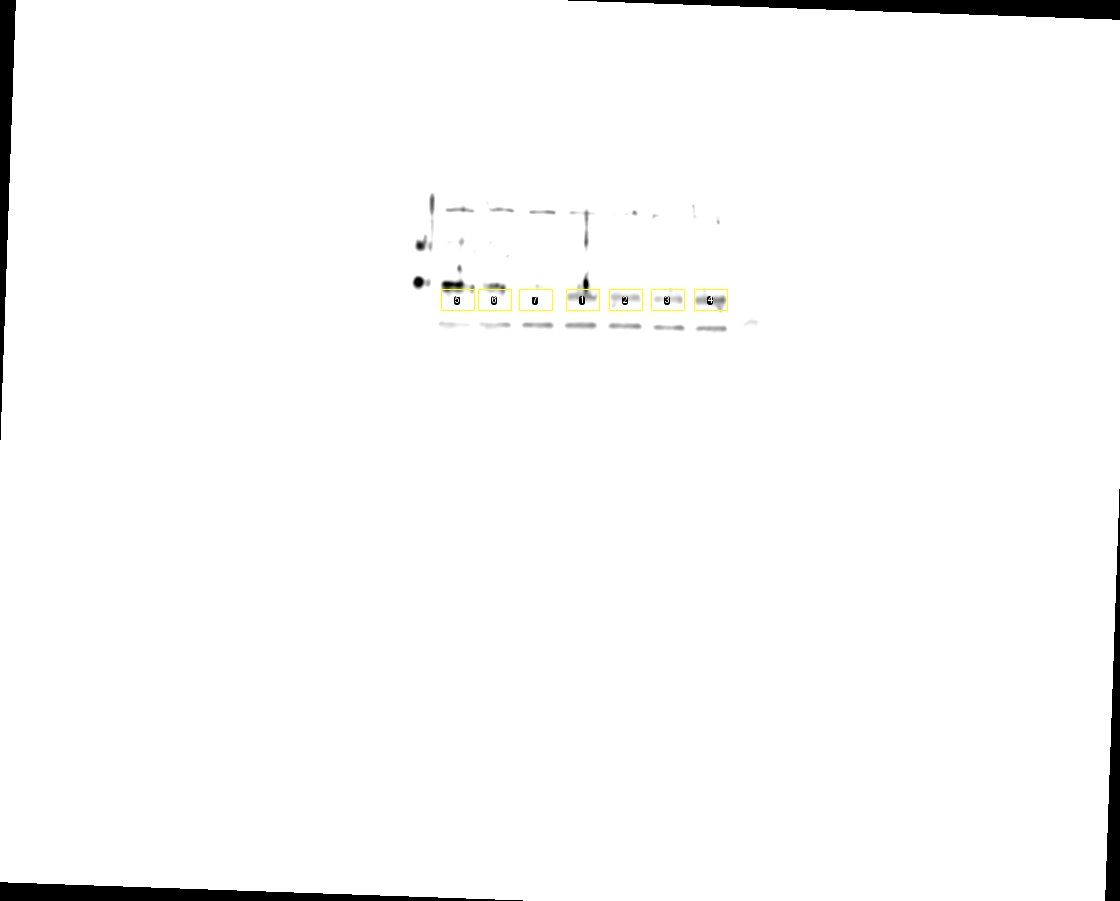

Supplement: Figure 6—source data 2. [file elife-100747-fig6-data2.zip › Figure 6 - Source Data 2 (original western files)/ATF4-cell signaling/23.01.16_16.35.38_S2_F10.jpg]

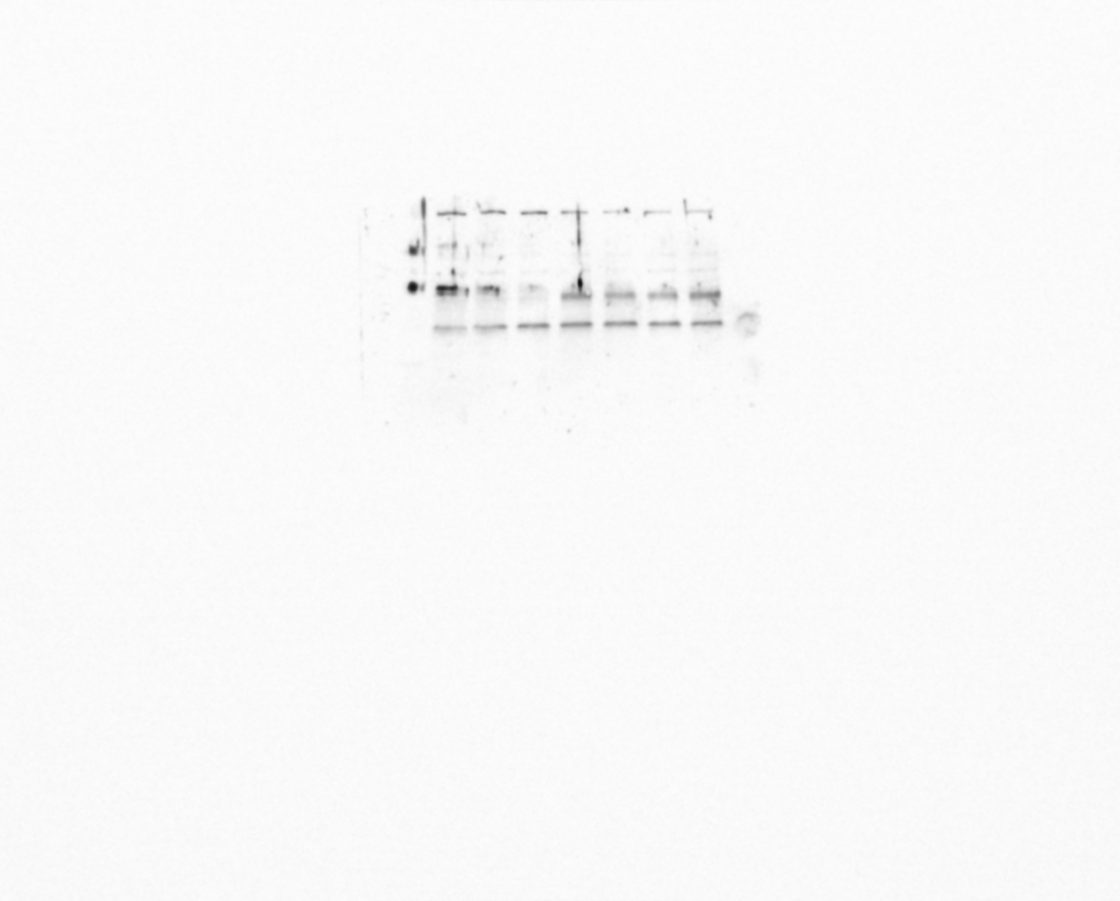

Supplement: Figure 6—source data 2. [file elife-100747-fig6-data2.zip › Figure 6 - Source Data 2 (original western files)/ATF4-cell signaling/23.01.16_16.35.38_S2_F10.tif]

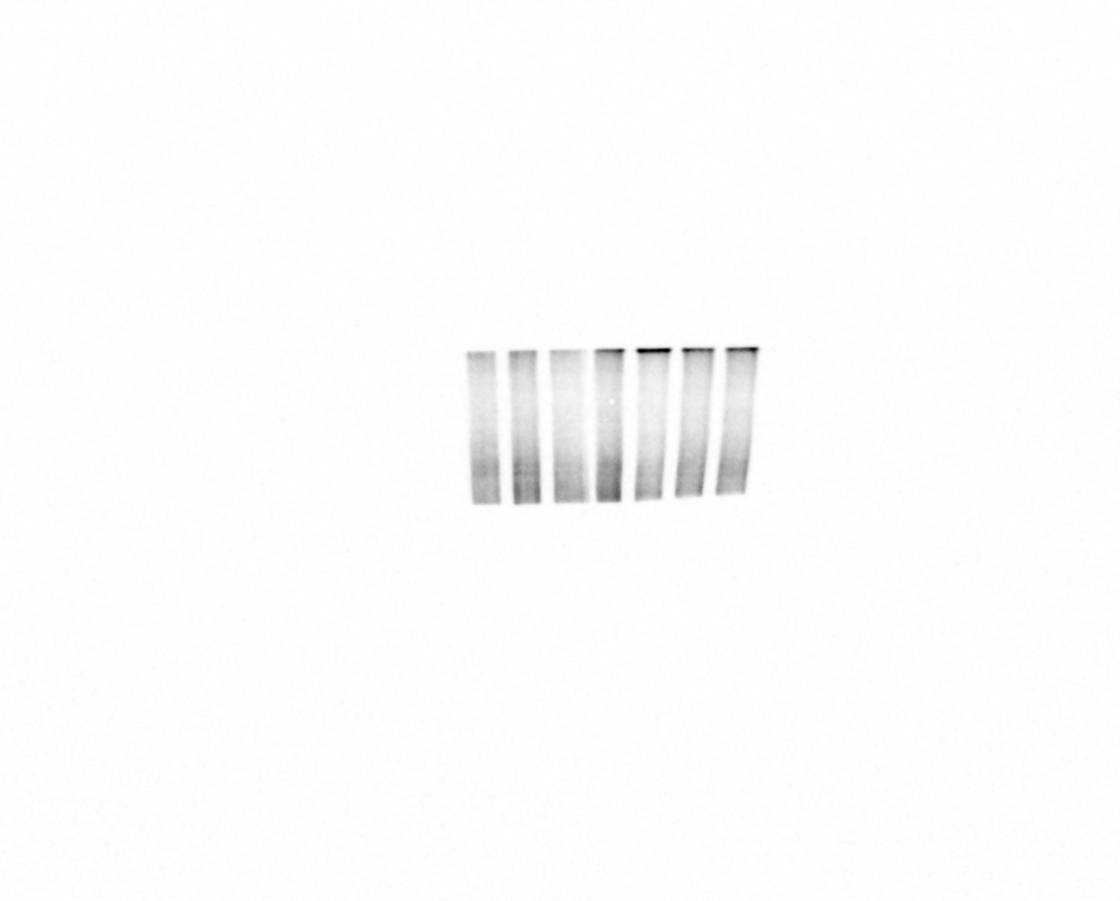

Supplement: Figure 6—source data 2. [file elife-100747-fig6-data2.zip › Figure 6 - Source Data 2 (original western files)/eIF1a-Total/23.01.16_16.37.30.tif]

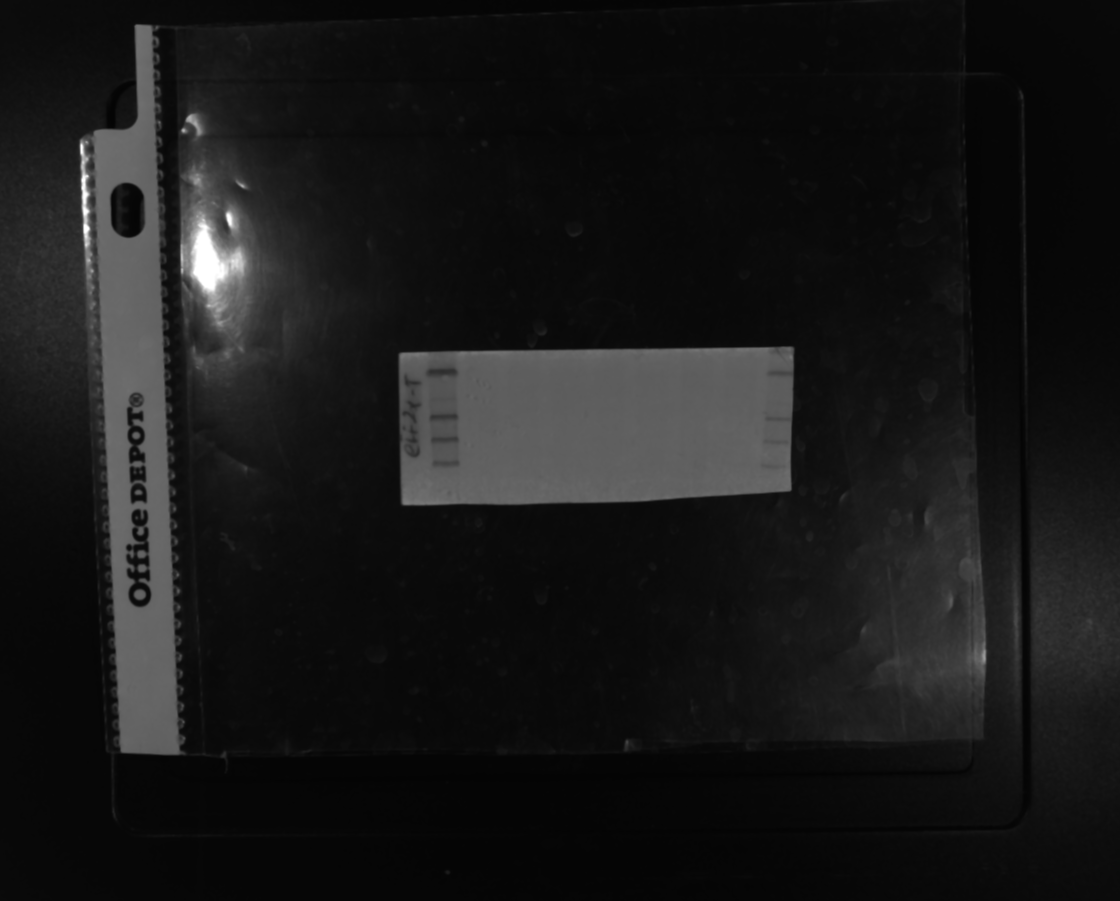

Supplement: Figure 6—source data 2. [file elife-100747-fig6-data2.zip › Figure 6 - Source Data 2 (original western files)/eIF1a-Total/23.01.16_16.38.05.tif]

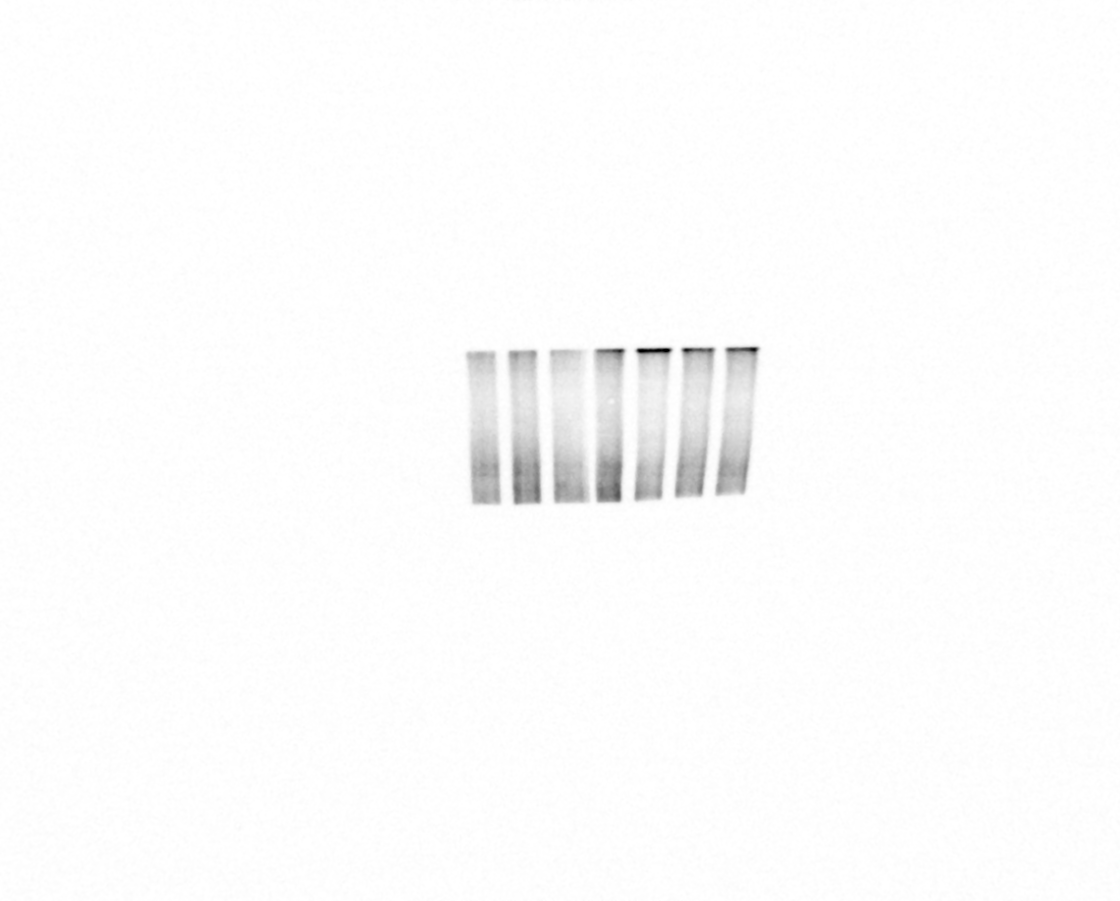

Supplement: Figure 6—source data 2. [file elife-100747-fig6-data2.zip › Figure 6 - Source Data 2 (original western files)/eIF1a-Total/23.01.16_16.39.29_S3_F01.tif]

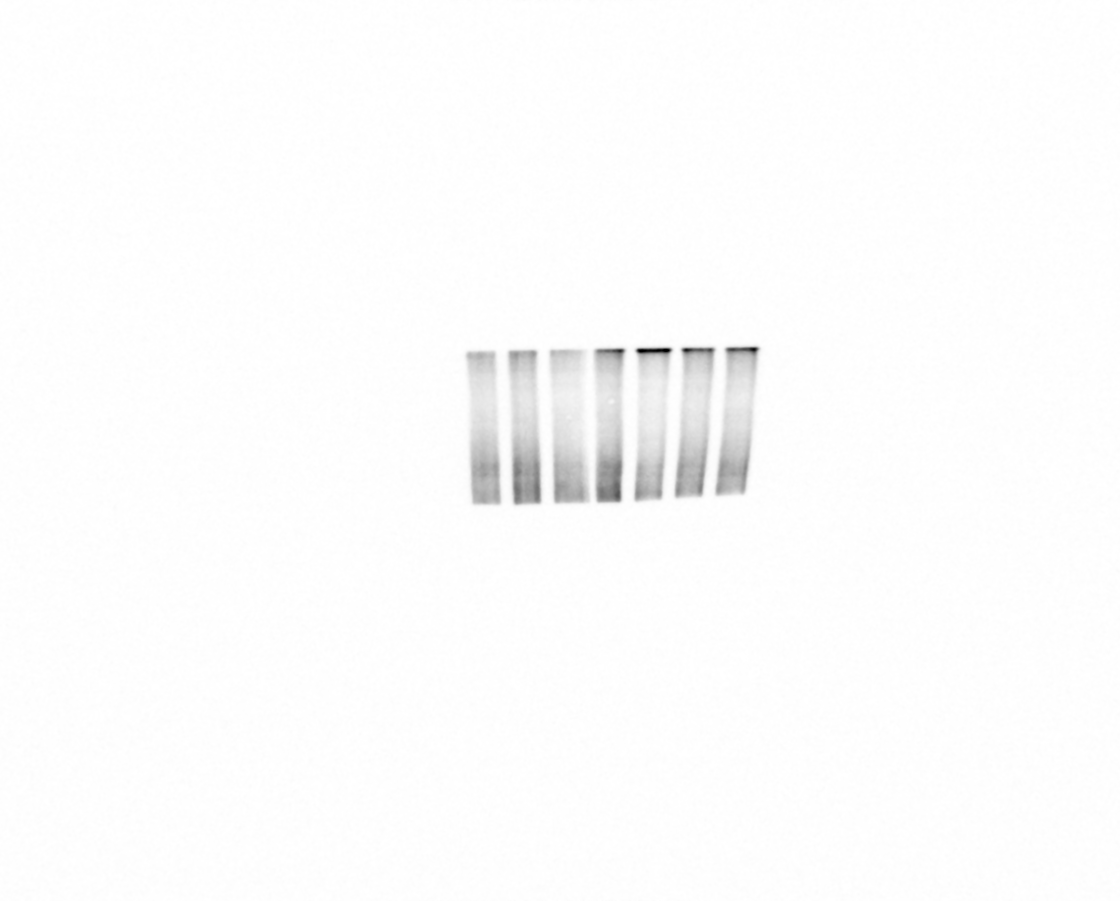

Supplement: Figure 6—source data 2. [file elife-100747-fig6-data2.zip › Figure 6 - Source Data 2 (original western files)/eIF1a-Total/23.01.16_16.39.29_S3_F02.tif]

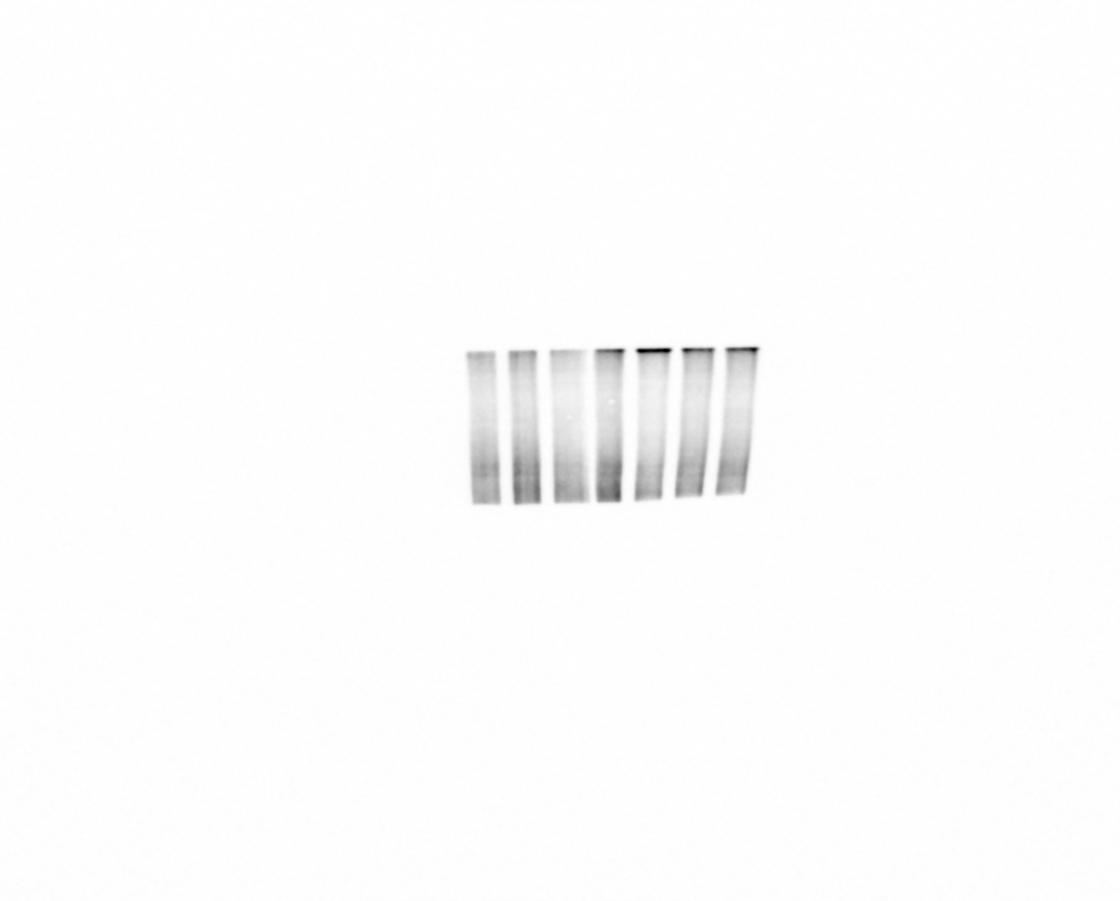

Supplement: Figure 6—source data 2. [file elife-100747-fig6-data2.zip › Figure 6 - Source Data 2 (original western files)/eIF1a-Total/23.01.16_16.39.29_S3_F03.tif]

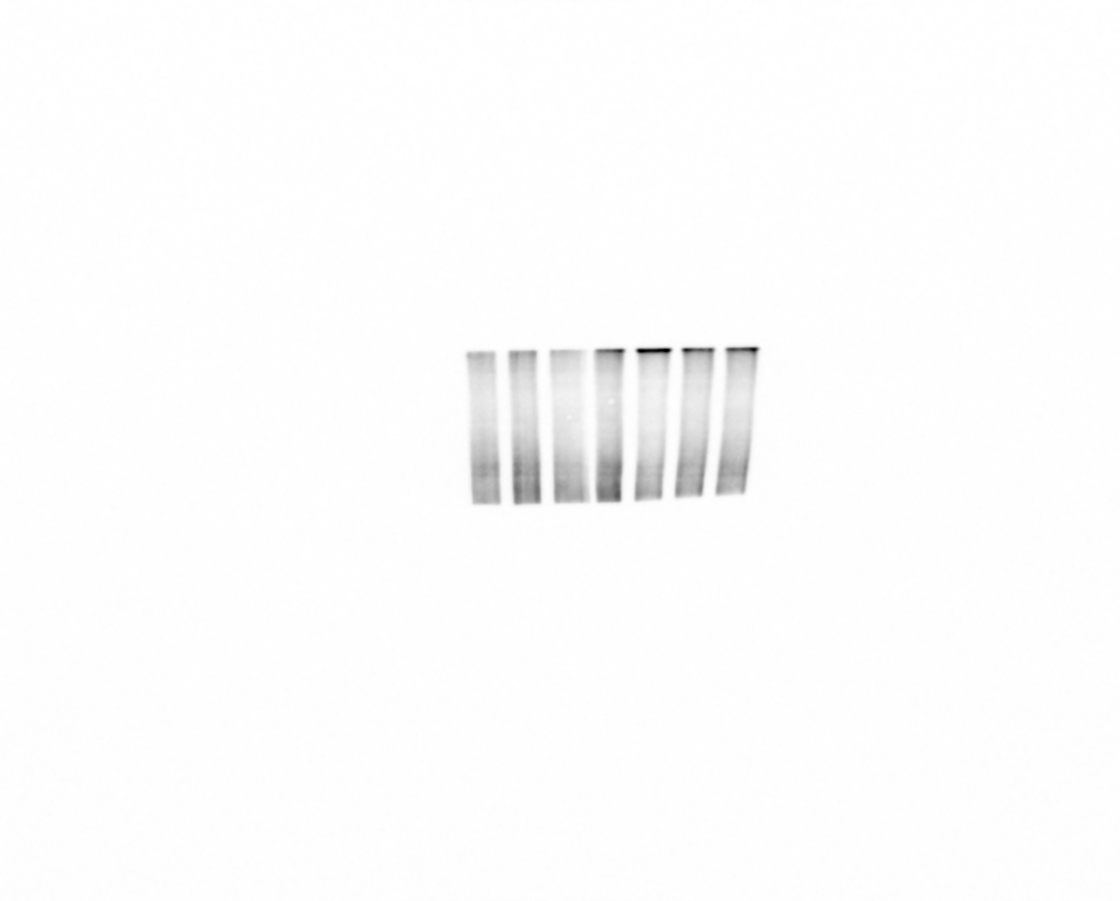

Supplement: Figure 6—source data 2. [file elife-100747-fig6-data2.zip › Figure 6 - Source Data 2 (original western files)/eIF1a-Total/23.01.16_16.39.29_S3_F04.tif]

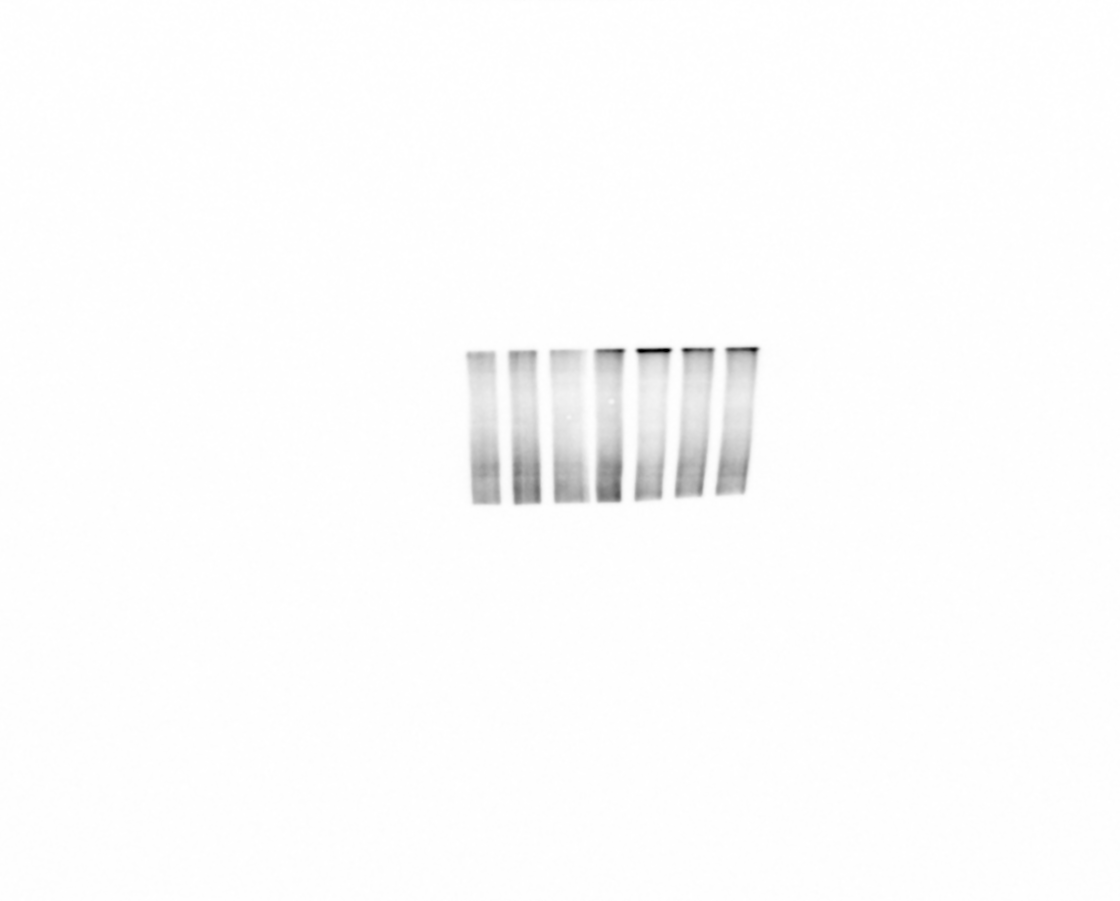

Supplement: Figure 6—source data 2. [file elife-100747-fig6-data2.zip › Figure 6 - Source Data 2 (original western files)/eIF1a-Total/23.01.16_16.39.29_S3_F05.tif]

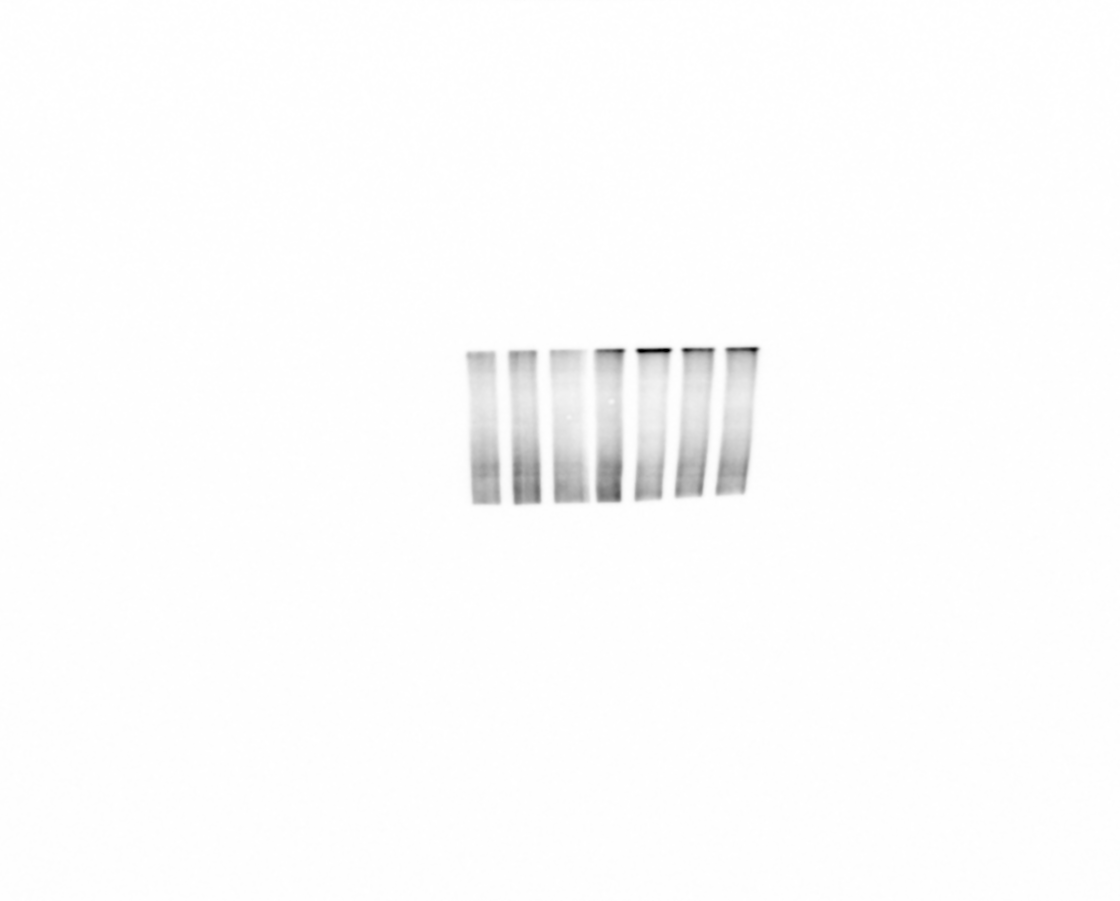

Supplement: Figure 6—source data 2. [file elife-100747-fig6-data2.zip › Figure 6 - Source Data 2 (original western files)/eIF1a-Total/23.01.16_16.39.29_S3_F06.tif]

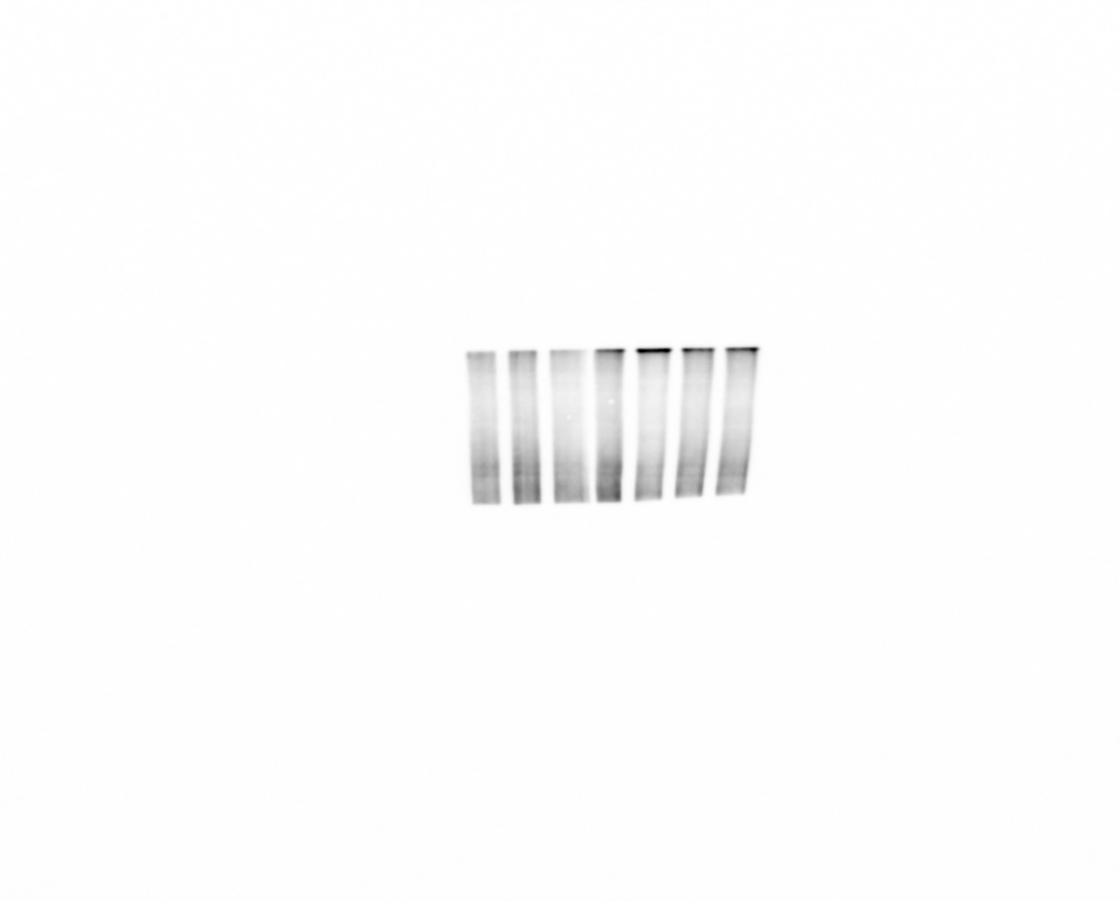

Supplement: Figure 6—source data 2. [file elife-100747-fig6-data2.zip › Figure 6 - Source Data 2 (original western files)/eIF1a-Total/23.01.16_16.39.29_S3_F07.tif]

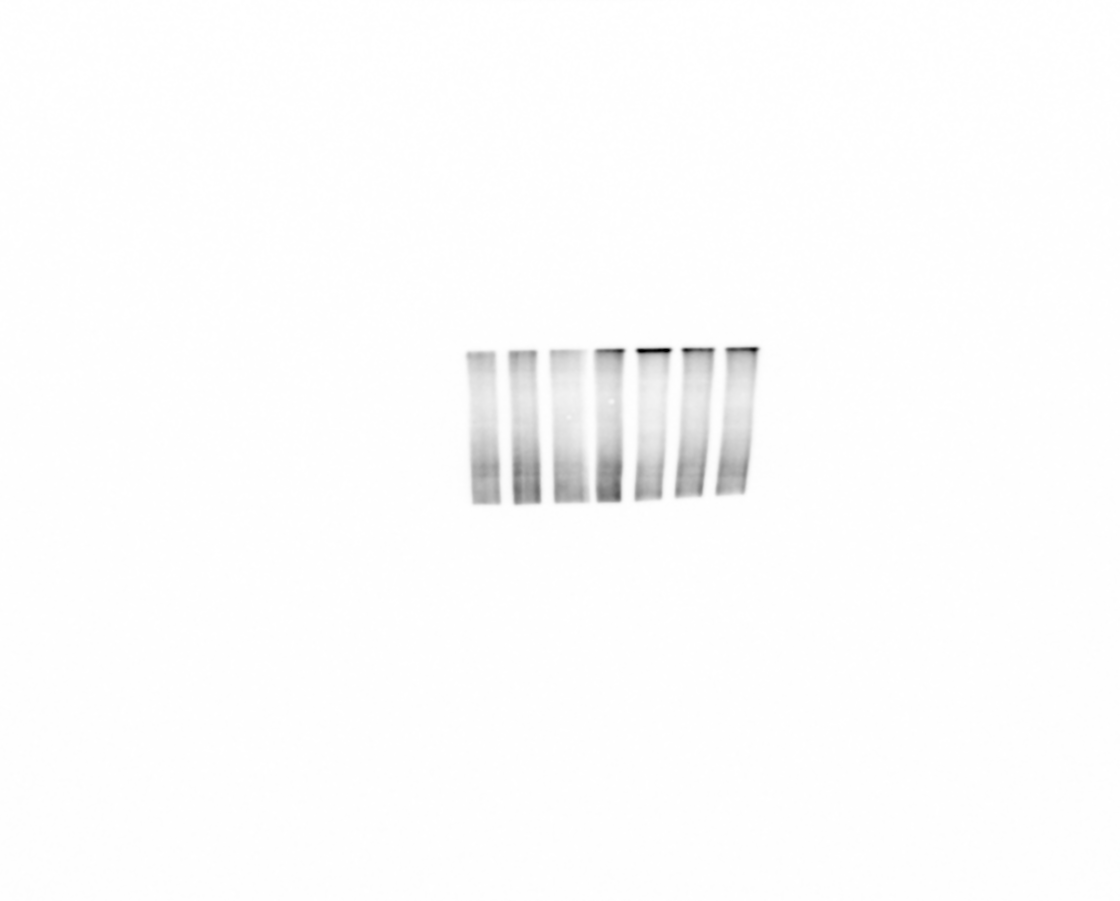

Supplement: Figure 6—source data 2. [file elife-100747-fig6-data2.zip › Figure 6 - Source Data 2 (original western files)/eIF1a-Total/23.01.16_16.39.29_S3_F08.tif]

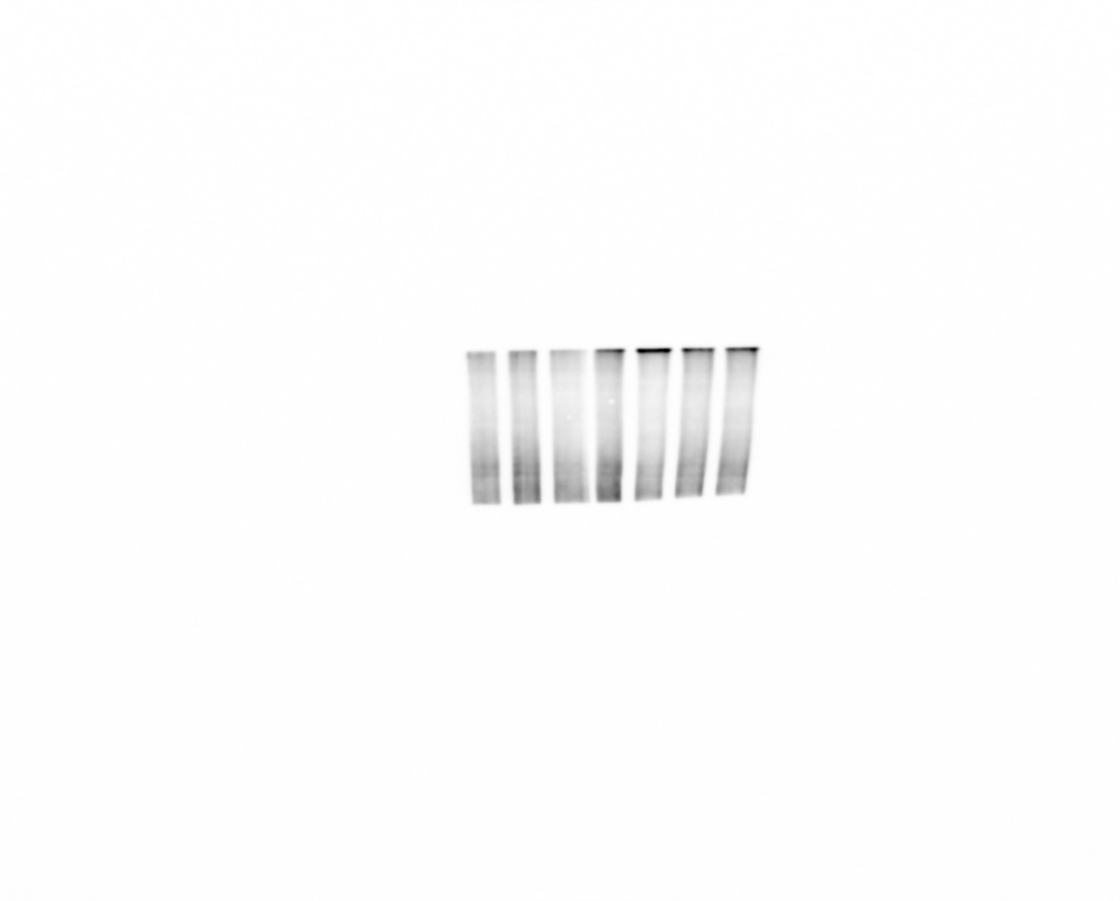

Supplement: Figure 6—source data 2. [file elife-100747-fig6-data2.zip › Figure 6 - Source Data 2 (original western files)/eIF1a-Total/23.01.16_16.39.29_S3_F09.tif]

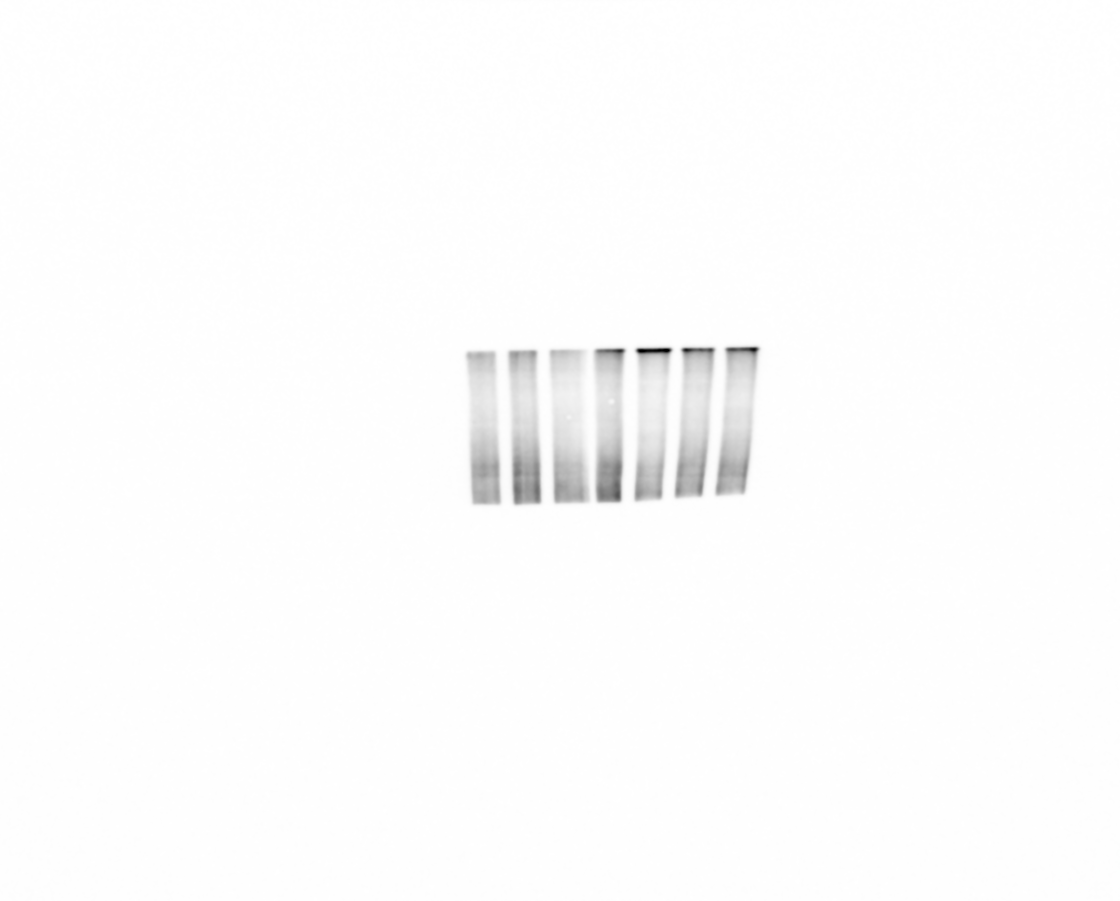

Supplement: Figure 6—source data 2. [file elife-100747-fig6-data2.zip › Figure 6 - Source Data 2 (original western files)/eIF1a-Total/23.01.16_16.39.29_S3_F10.tif]

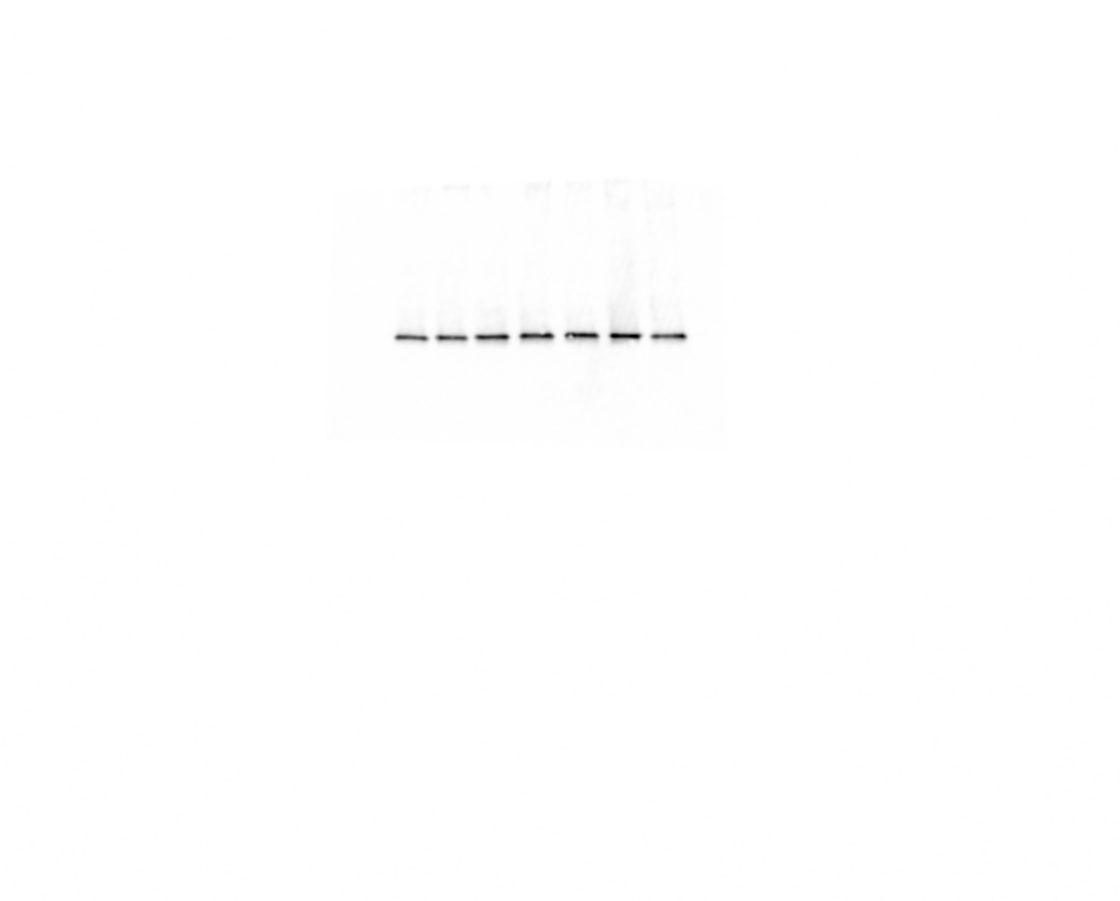

Supplement: Figure 6—source data 2. [file elife-100747-fig6-data2.zip › Figure 6 - Source Data 2 (original western files)/eIF1a-Total/23.01.20_14.53.32.tif]

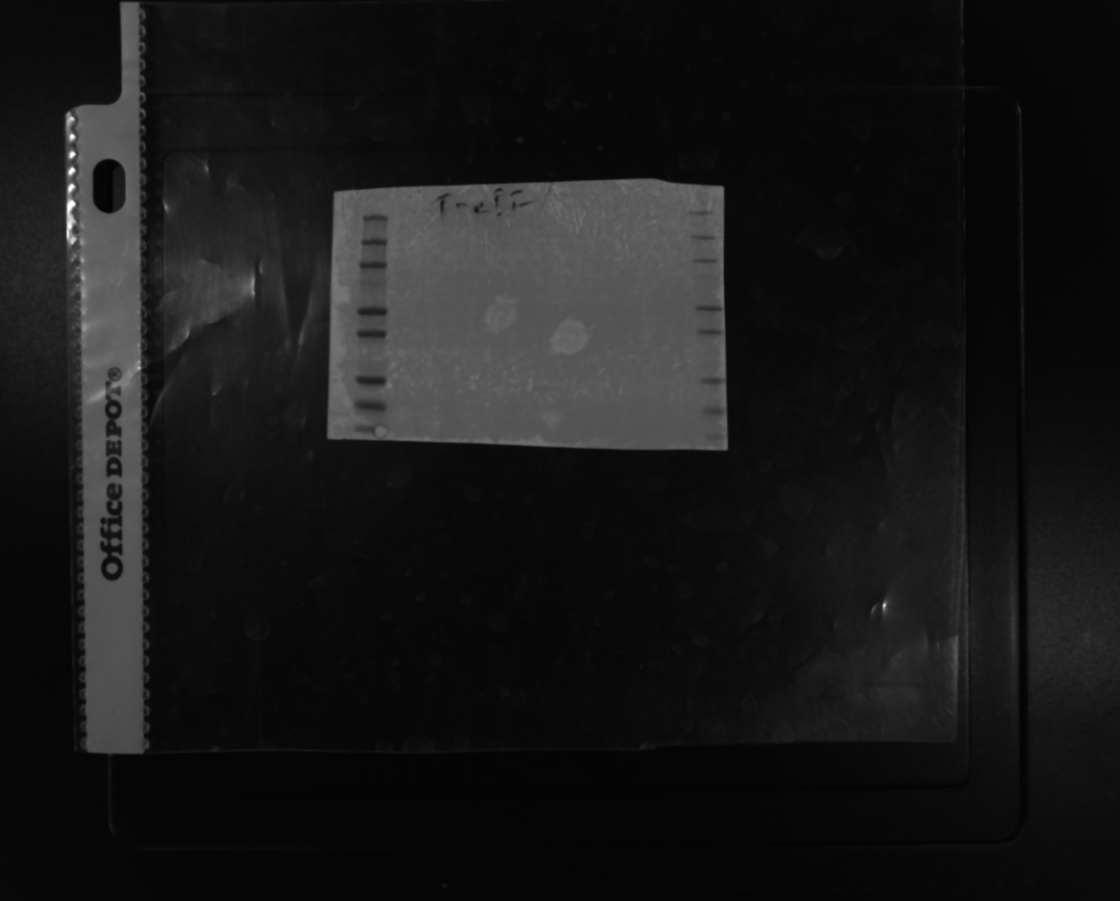

Supplement: Figure 6—source data 2. [file elife-100747-fig6-data2.zip › Figure 6 - Source Data 2 (original western files)/eIF1a-Total/23.01.20_14.54.06.tif]

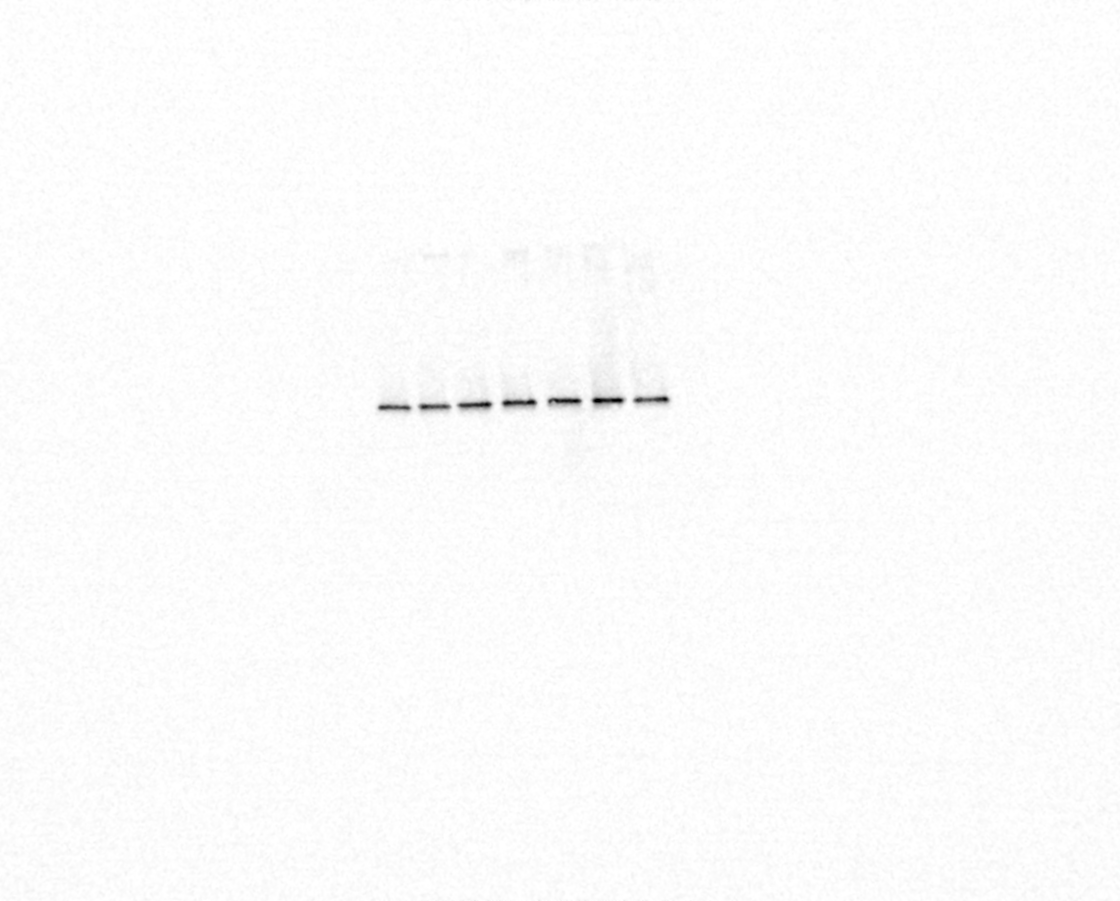

Supplement: Figure 6—source data 2. [file elife-100747-fig6-data2.zip › Figure 6 - Source Data 2 (original western files)/eIF1a-Total/23.01.20_14.54.57_S1_F01.tif]

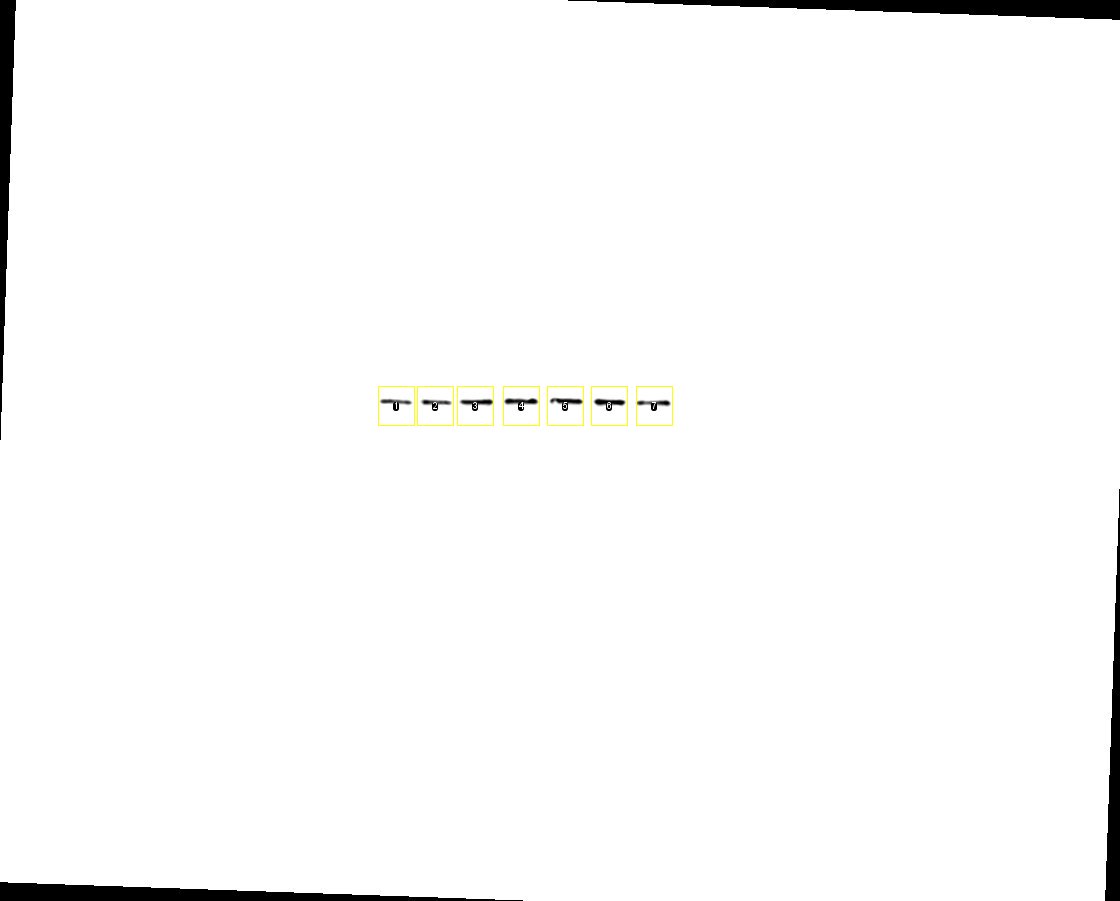

Supplement: Figure 6—source data 2. [file elife-100747-fig6-data2.zip › Figure 6 - Source Data 2 (original western files)/eIF1a-Total/23.01.20_14.54.57_S1_F02.jpg]

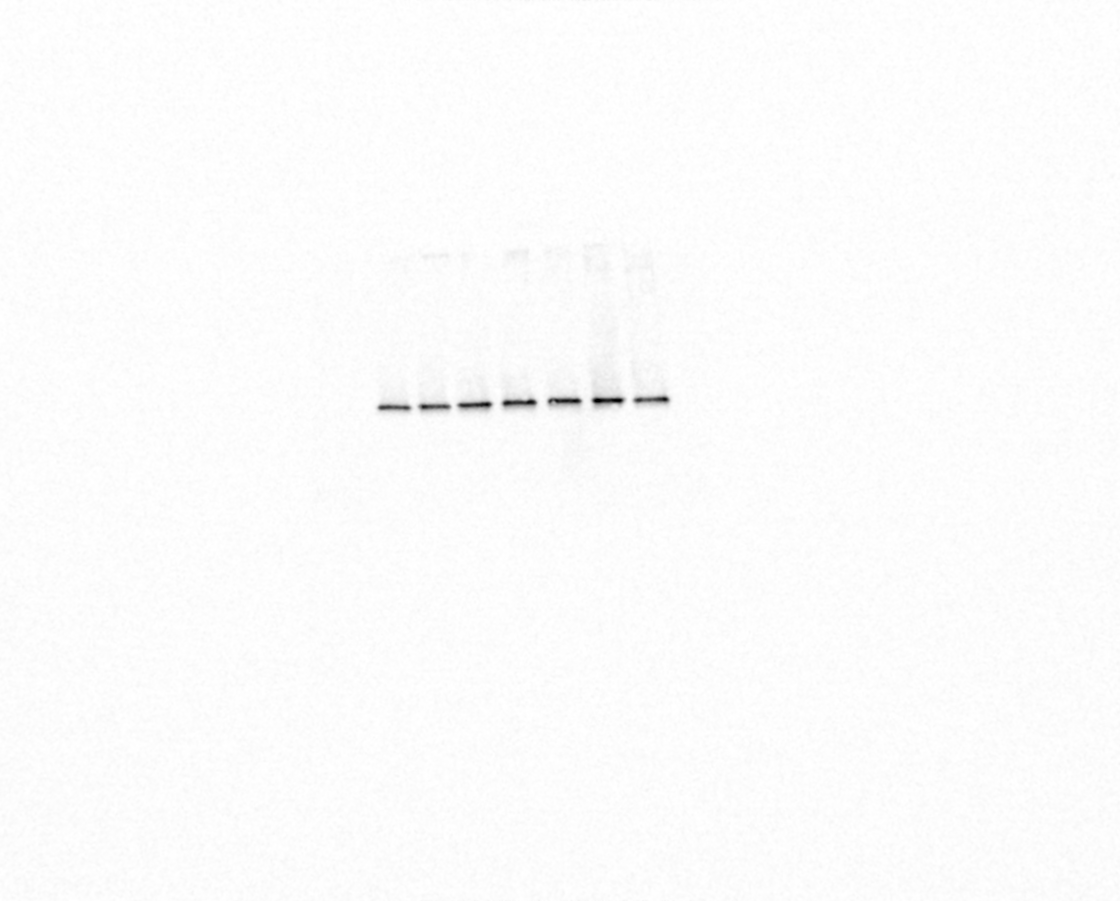

Supplement: Figure 6—source data 2. [file elife-100747-fig6-data2.zip › Figure 6 - Source Data 2 (original western files)/eIF1a-Total/23.01.20_14.54.57_S1_F02.tif]

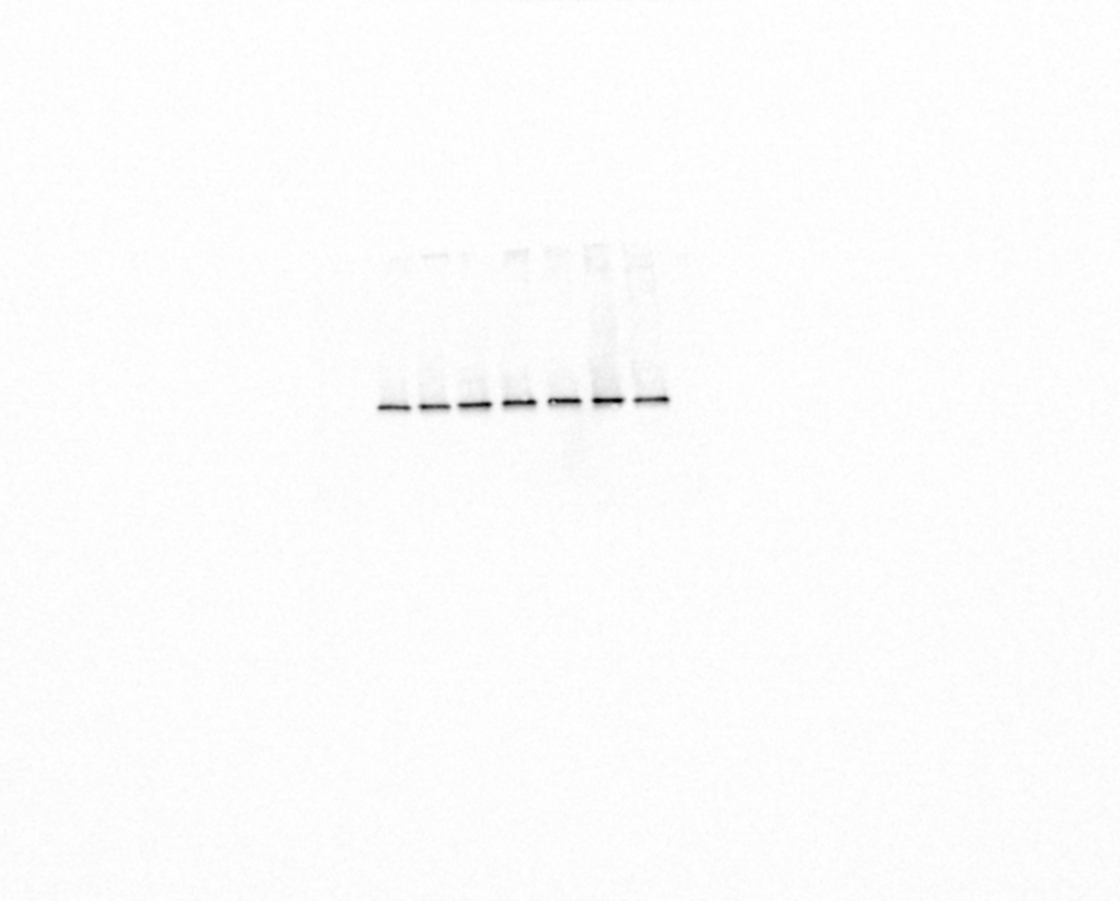

Supplement: Figure 6—source data 2. [file elife-100747-fig6-data2.zip › Figure 6 - Source Data 2 (original western files)/eIF1a-Total/23.01.20_14.54.57_S1_F03.tif]

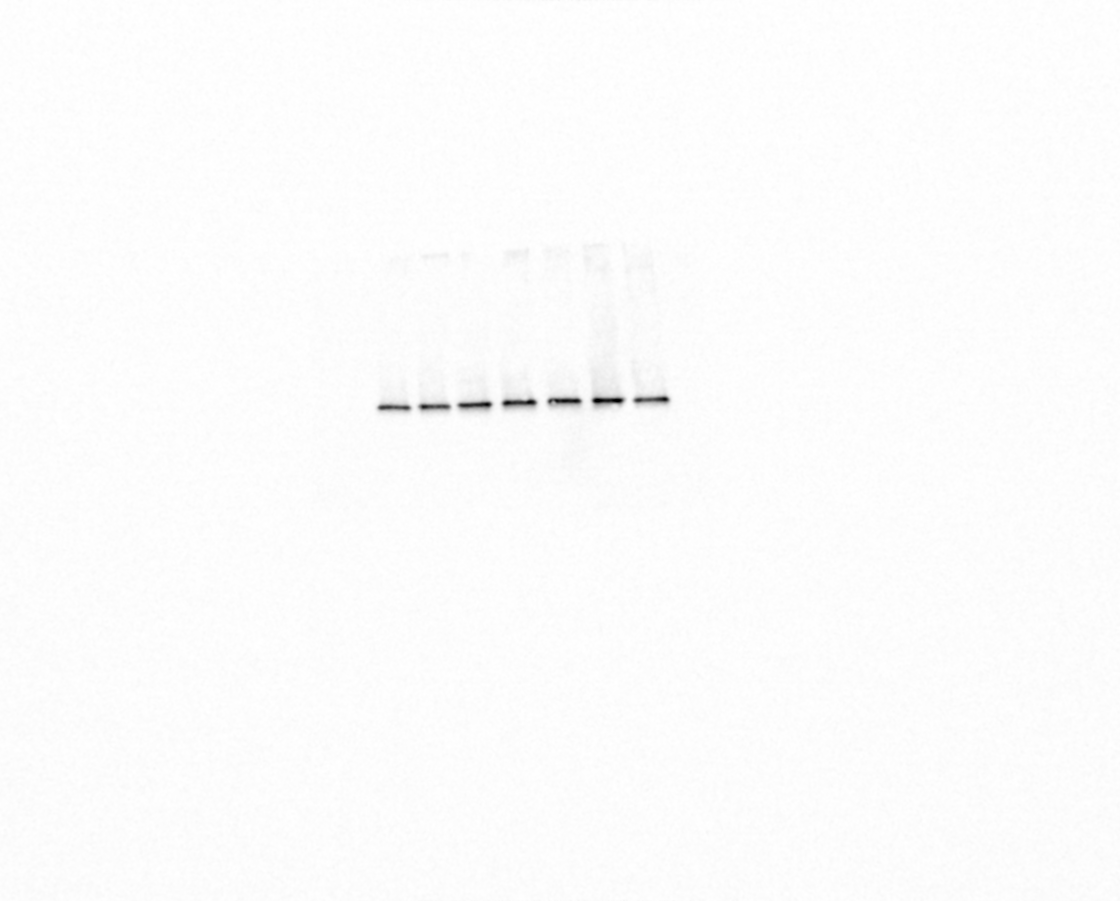

Supplement: Figure 6—source data 2. [file elife-100747-fig6-data2.zip › Figure 6 - Source Data 2 (original western files)/eIF1a-Total/23.01.20_14.54.57_S1_F04.tif]

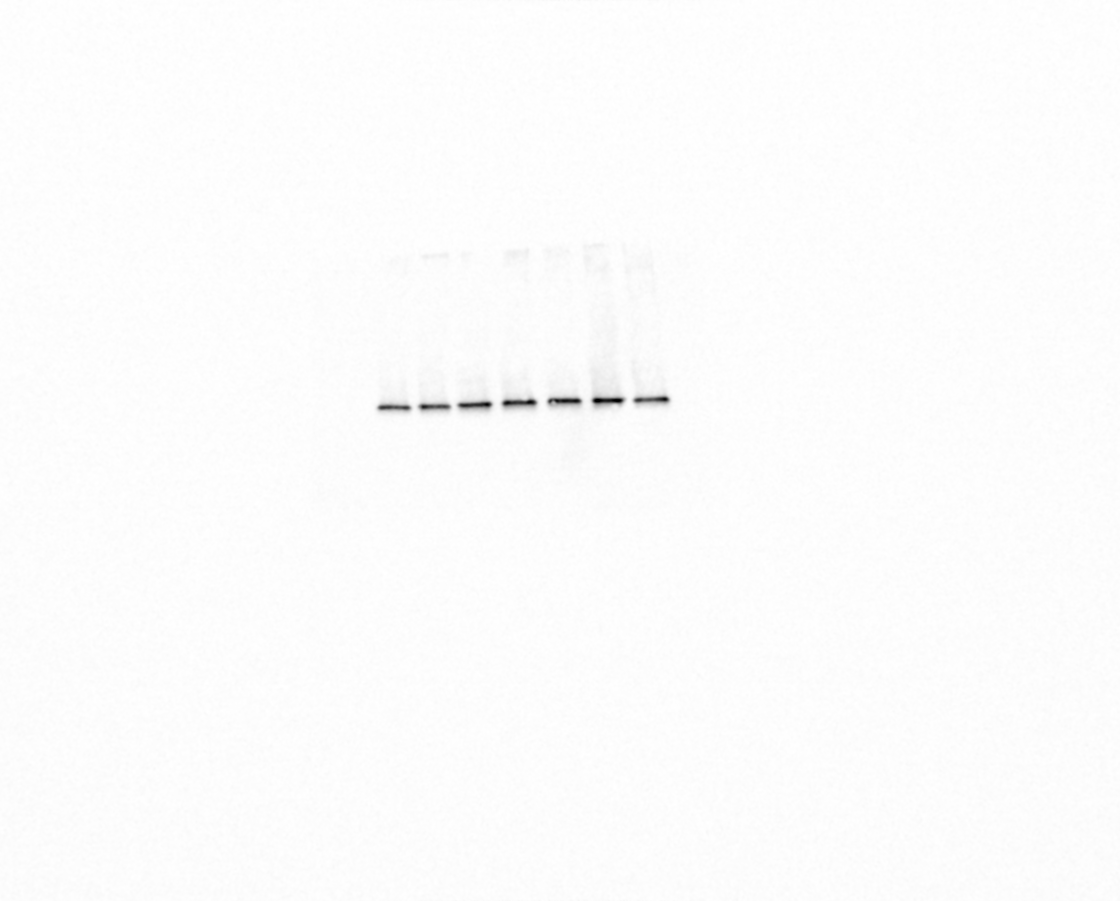

Supplement: Figure 6—source data 2. [file elife-100747-fig6-data2.zip › Figure 6 - Source Data 2 (original western files)/eIF1a-Total/23.01.20_14.54.57_S1_F05.tif]

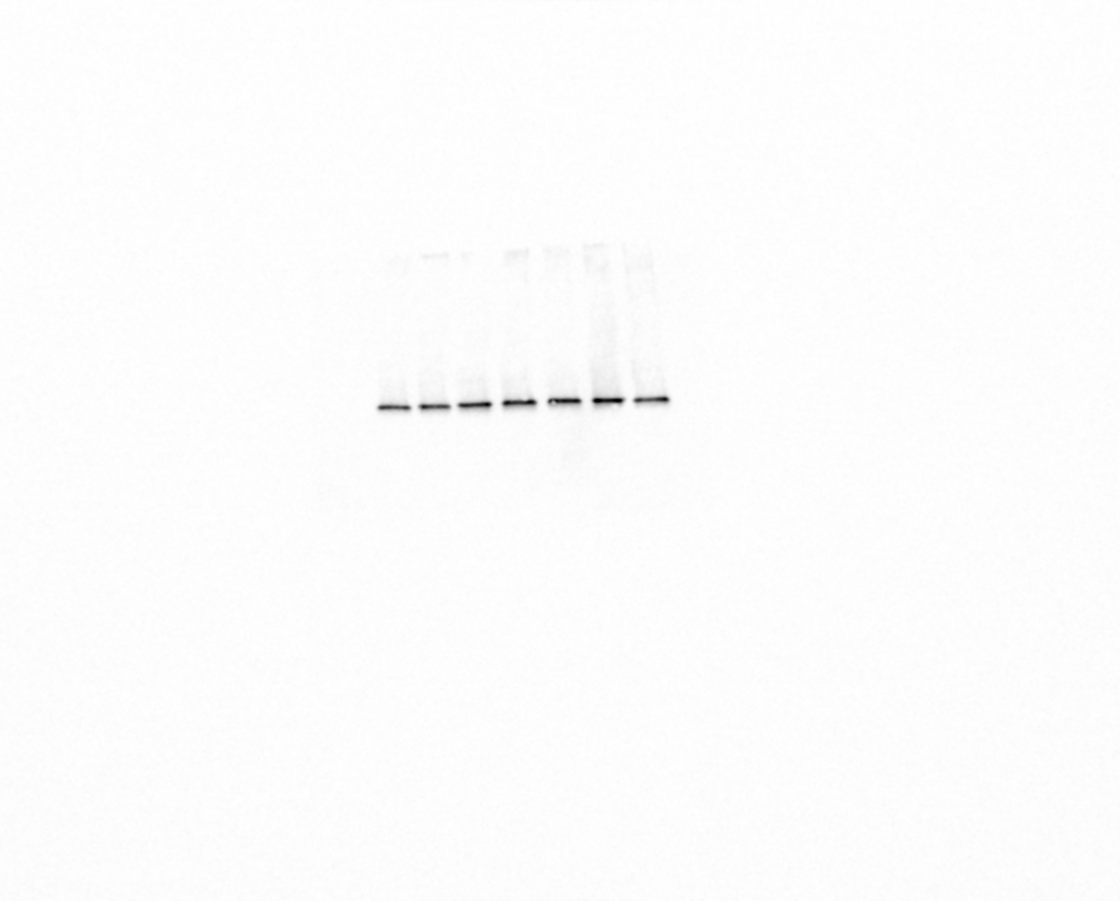

Supplement: Figure 6—source data 2. [file elife-100747-fig6-data2.zip › Figure 6 - Source Data 2 (original western files)/eIF1a-Total/23.01.20_14.54.57_S1_F06.tif]

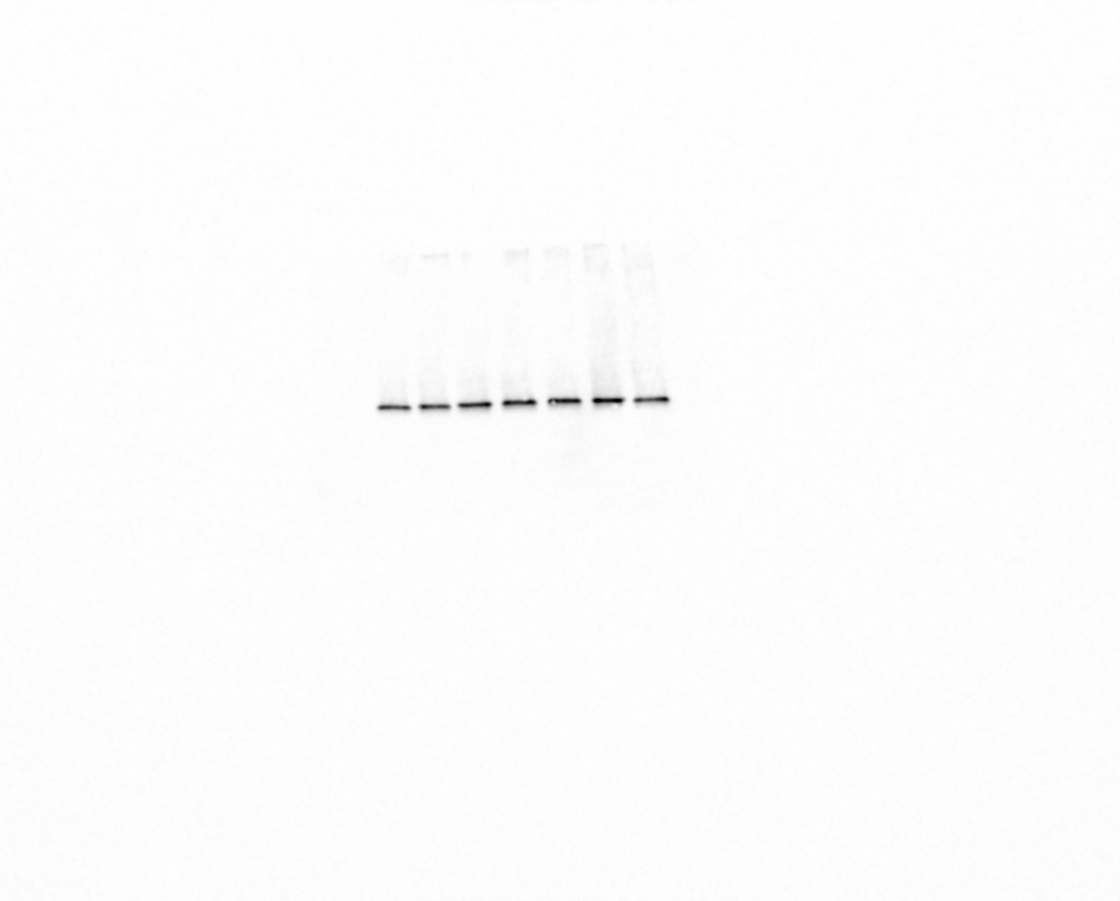

Supplement: Figure 6—source data 2. [file elife-100747-fig6-data2.zip › Figure 6 - Source Data 2 (original western files)/eIF1a-Total/23.01.20_14.54.57_S1_F07.tif]

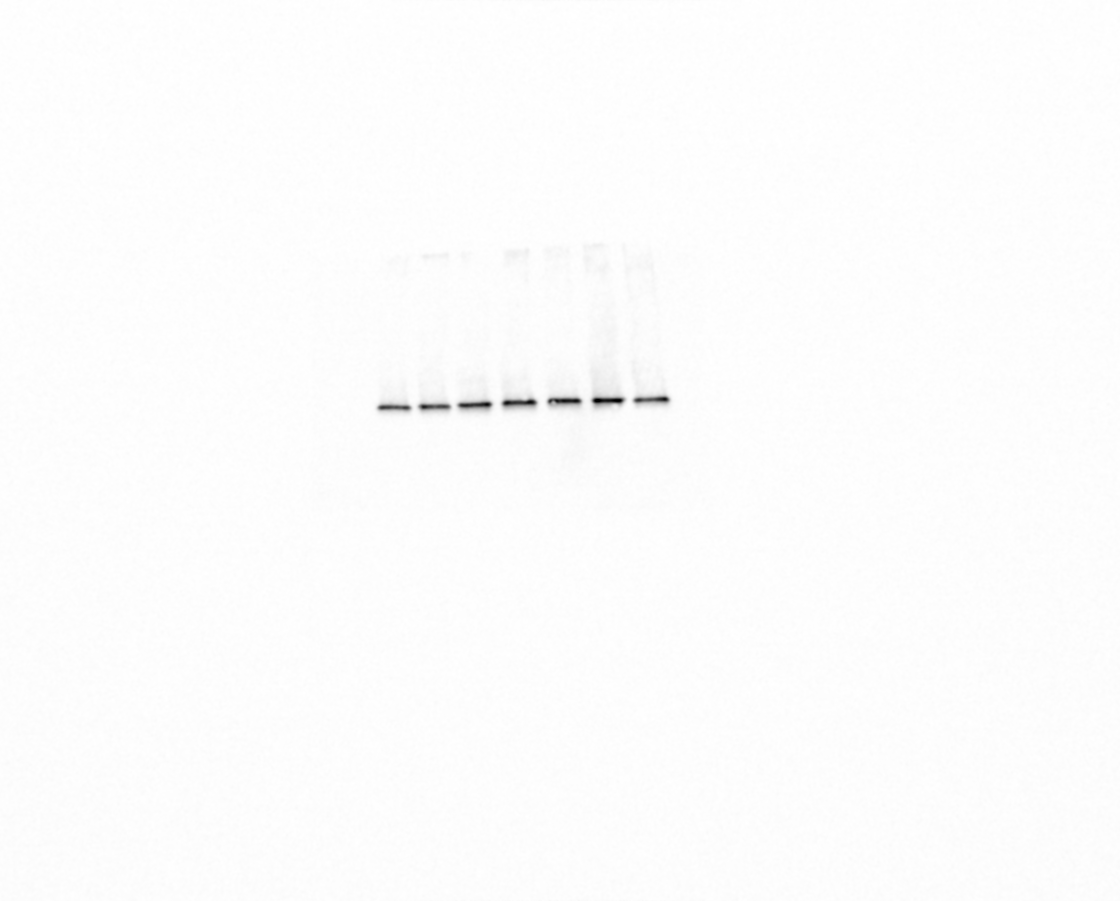

Supplement: Figure 6—source data 2. [file elife-100747-fig6-data2.zip › Figure 6 - Source Data 2 (original western files)/eIF1a-Total/23.01.20_14.54.57_S1_F08.tif]

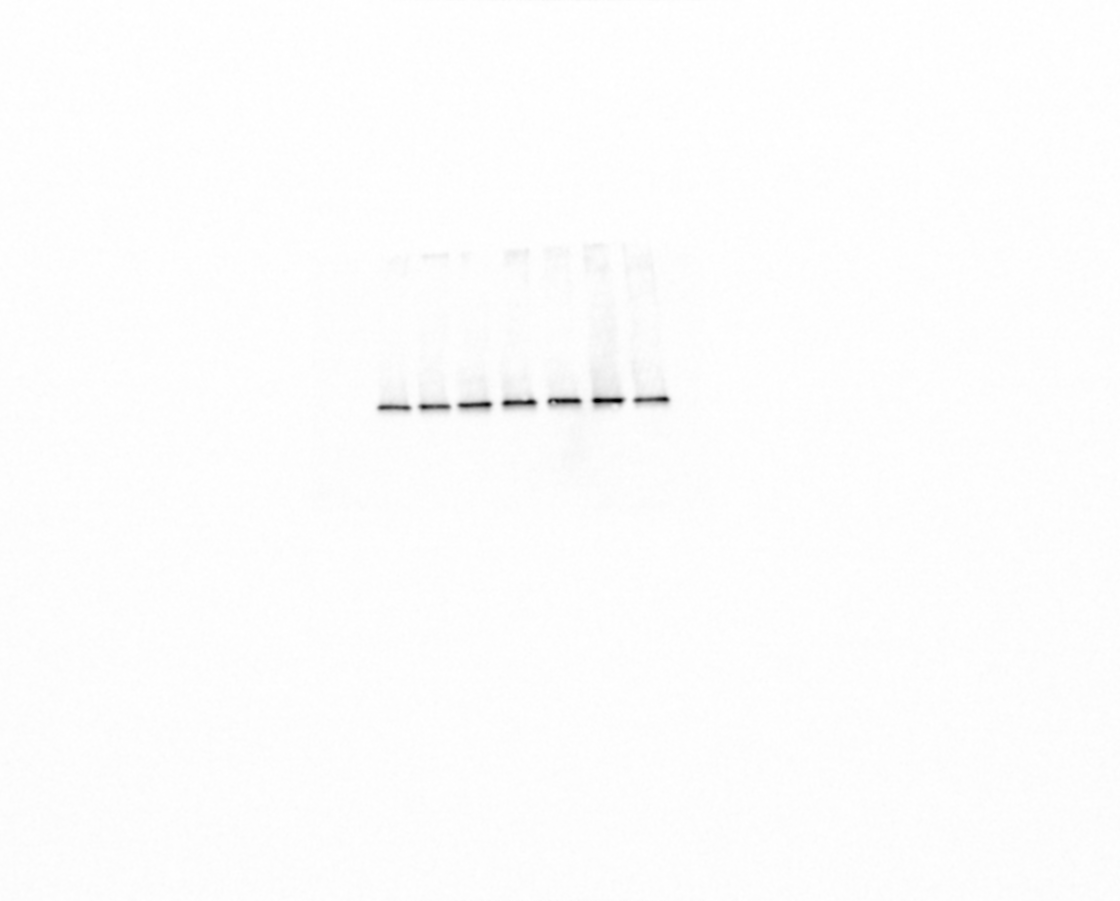

Supplement: Figure 6—source data 2. [file elife-100747-fig6-data2.zip › Figure 6 - Source Data 2 (original western files)/eIF1a-Total/23.01.20_14.54.57_S1_F09.tif]

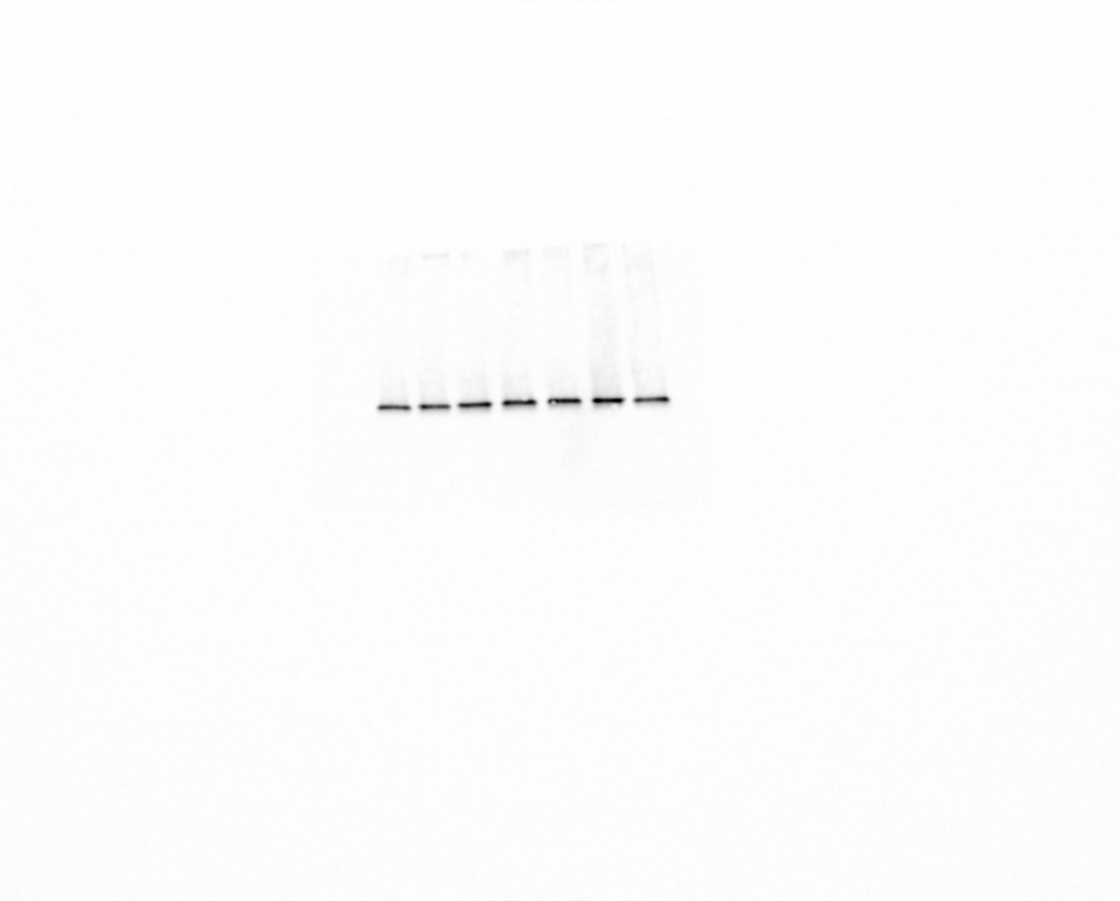

Supplement: Figure 6—source data 2. [file elife-100747-fig6-data2.zip › Figure 6 - Source Data 2 (original western files)/eIF1a-Total/23.01.20_14.54.57_S1_F10.tif]

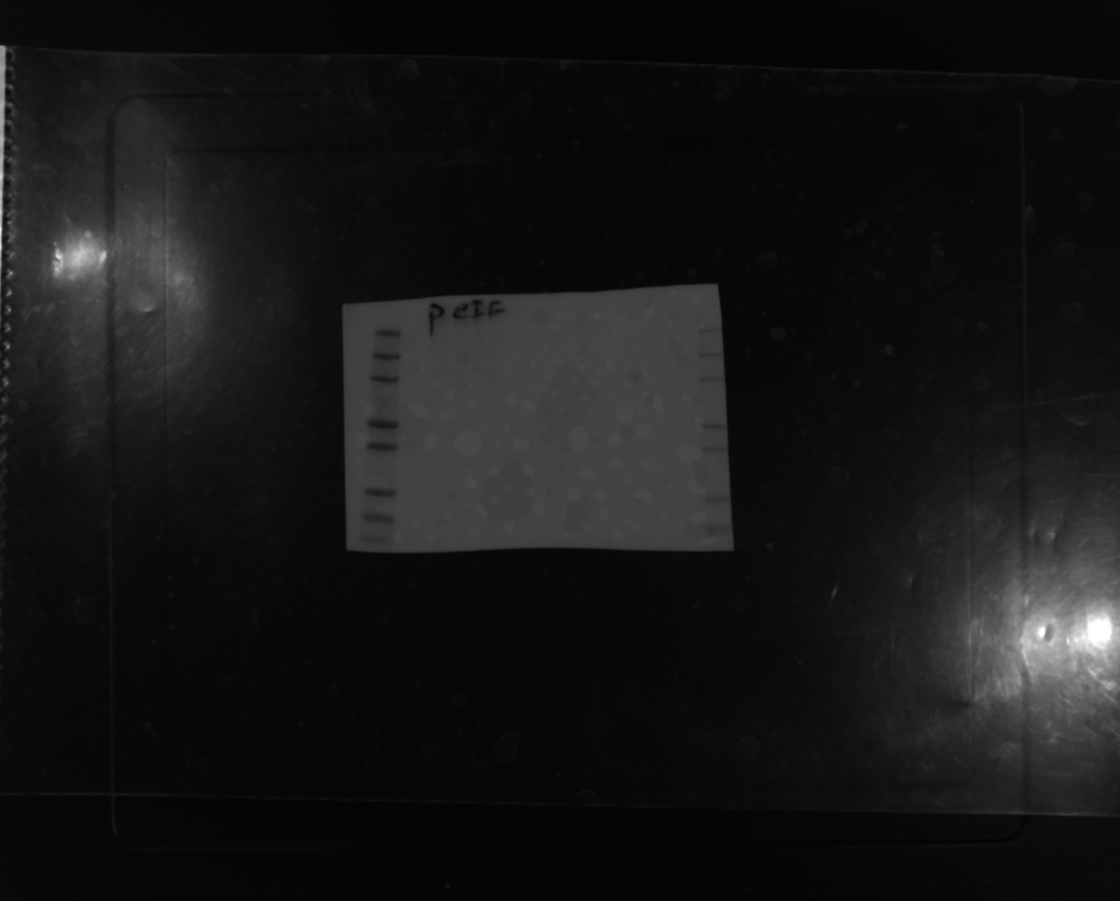

Supplement: Figure 6—source data 2. [file elife-100747-fig6-data2.zip › Figure 6 - Source Data 2 (original western files)/eIF2a-P-S51/23.01.20_15.12.53.tif]

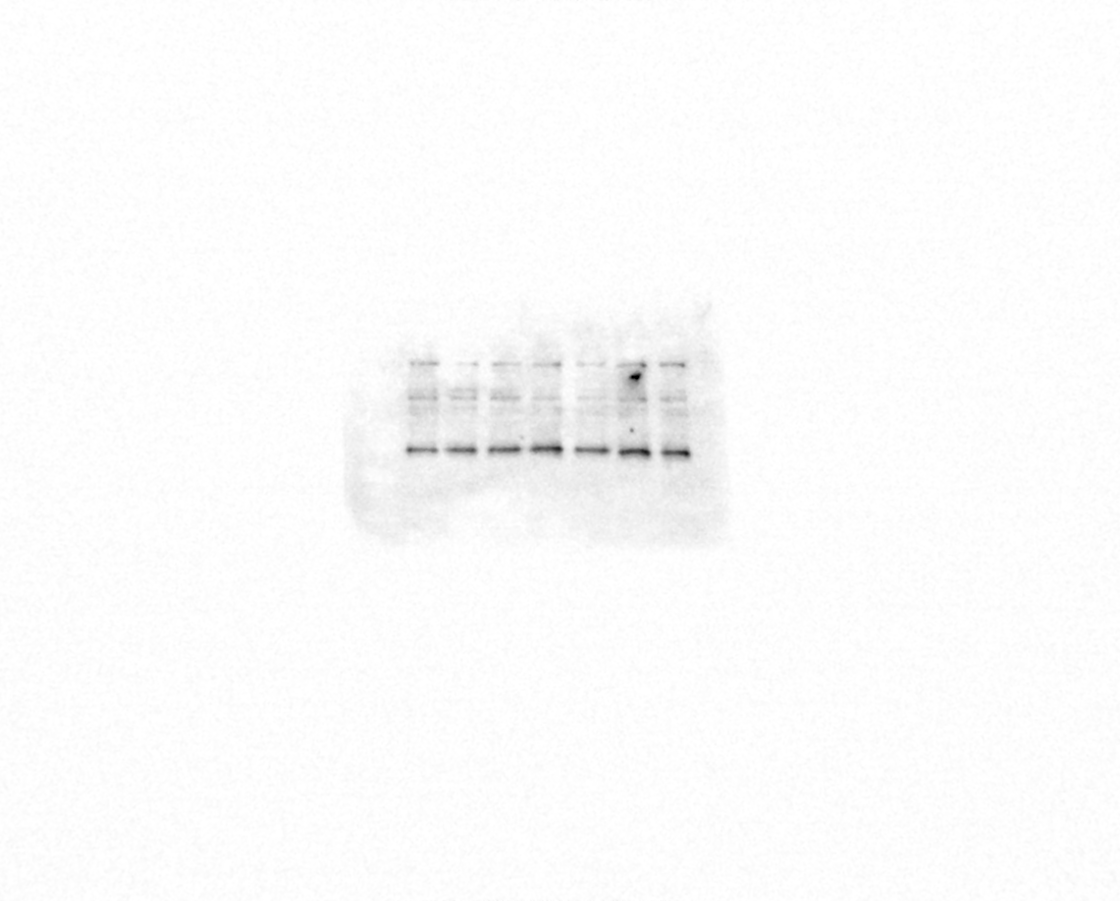

Supplement: Figure 6—source data 2. [file elife-100747-fig6-data2.zip › Figure 6 - Source Data 2 (original western files)/eIF2a-P-S51/23.01.20_15.13.23_S6_F01.tif]

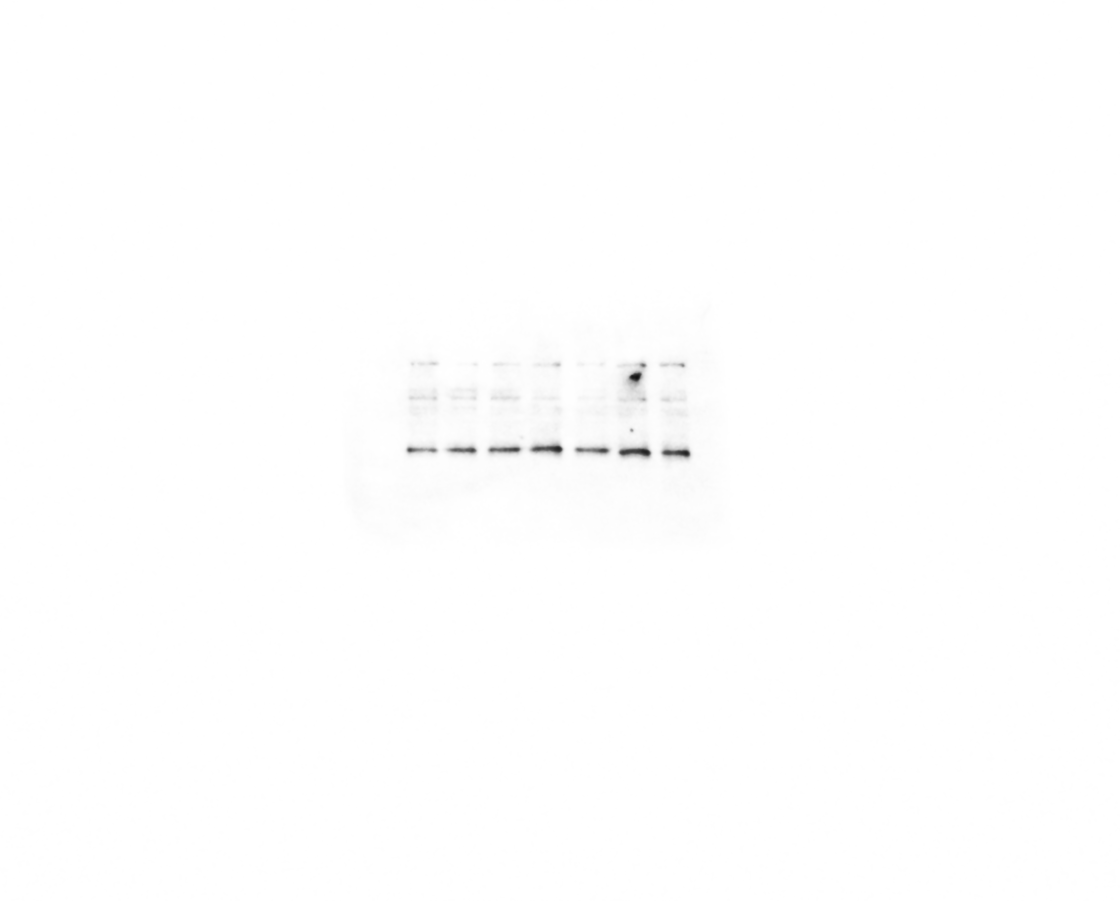

Supplement: Figure 6—source data 2. [file elife-100747-fig6-data2.zip › Figure 6 - Source Data 2 (original western files)/eIF2a-P-S51/23.01.20_15.13.23_S6_F02.tif]

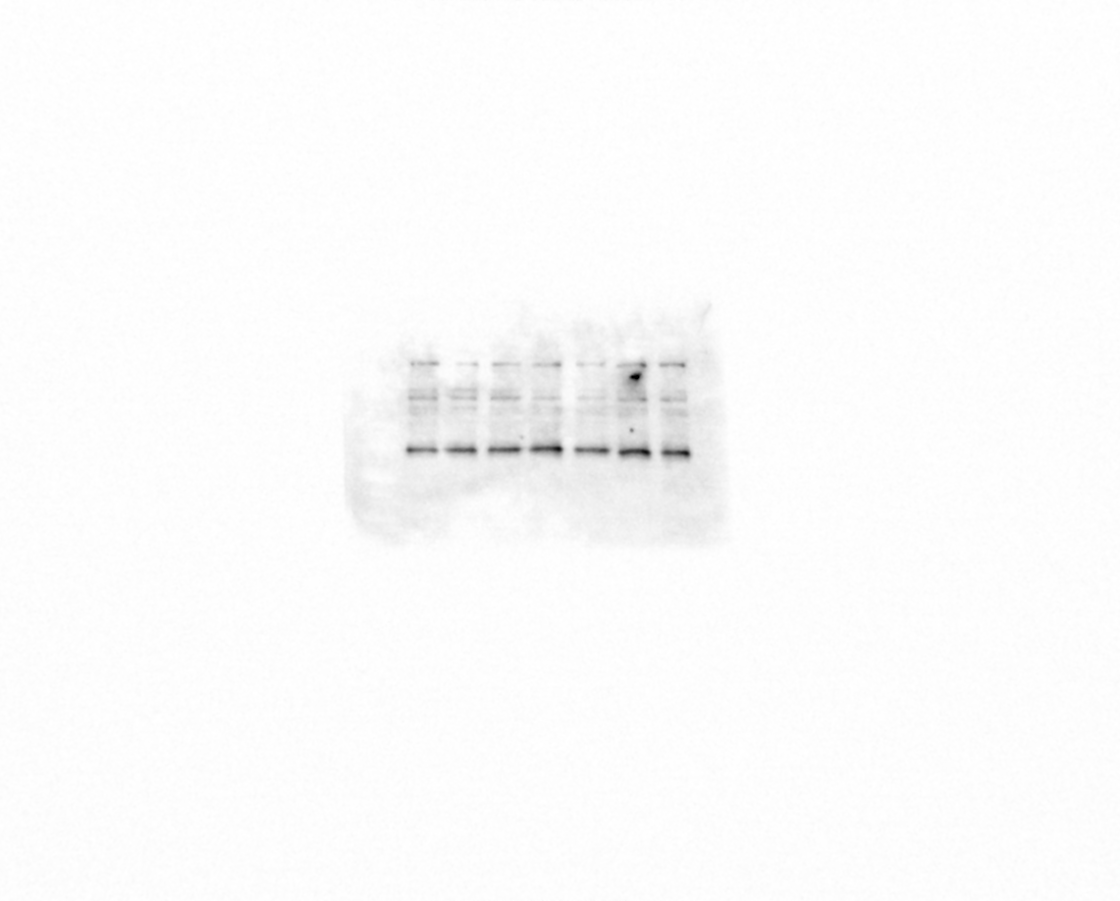

Supplement: Figure 6—source data 2. [file elife-100747-fig6-data2.zip › Figure 6 - Source Data 2 (original western files)/eIF2a-P-S51/23.01.20_15.13.23_S6_F03.tif]

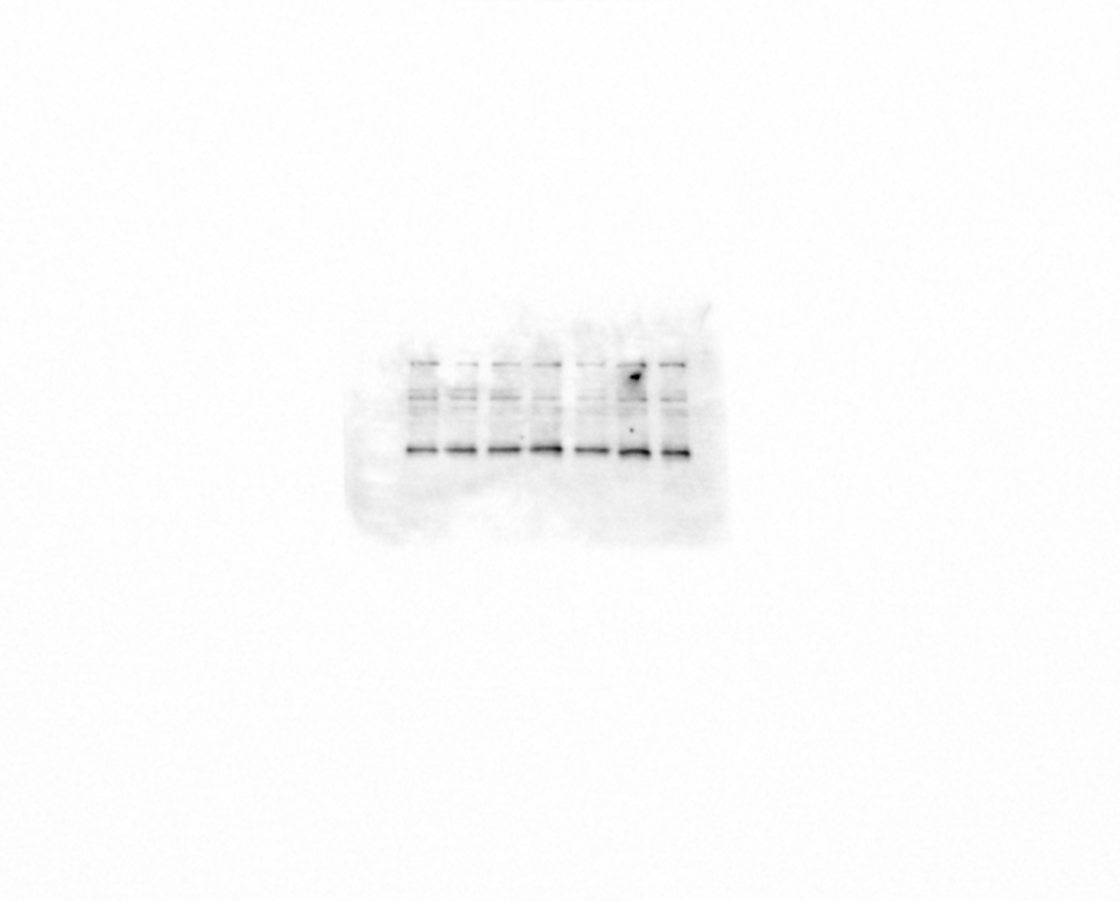

Supplement: Figure 6—source data 2. [file elife-100747-fig6-data2.zip › Figure 6 - Source Data 2 (original western files)/eIF2a-P-S51/23.01.20_15.13.23_S6_F04.tif]

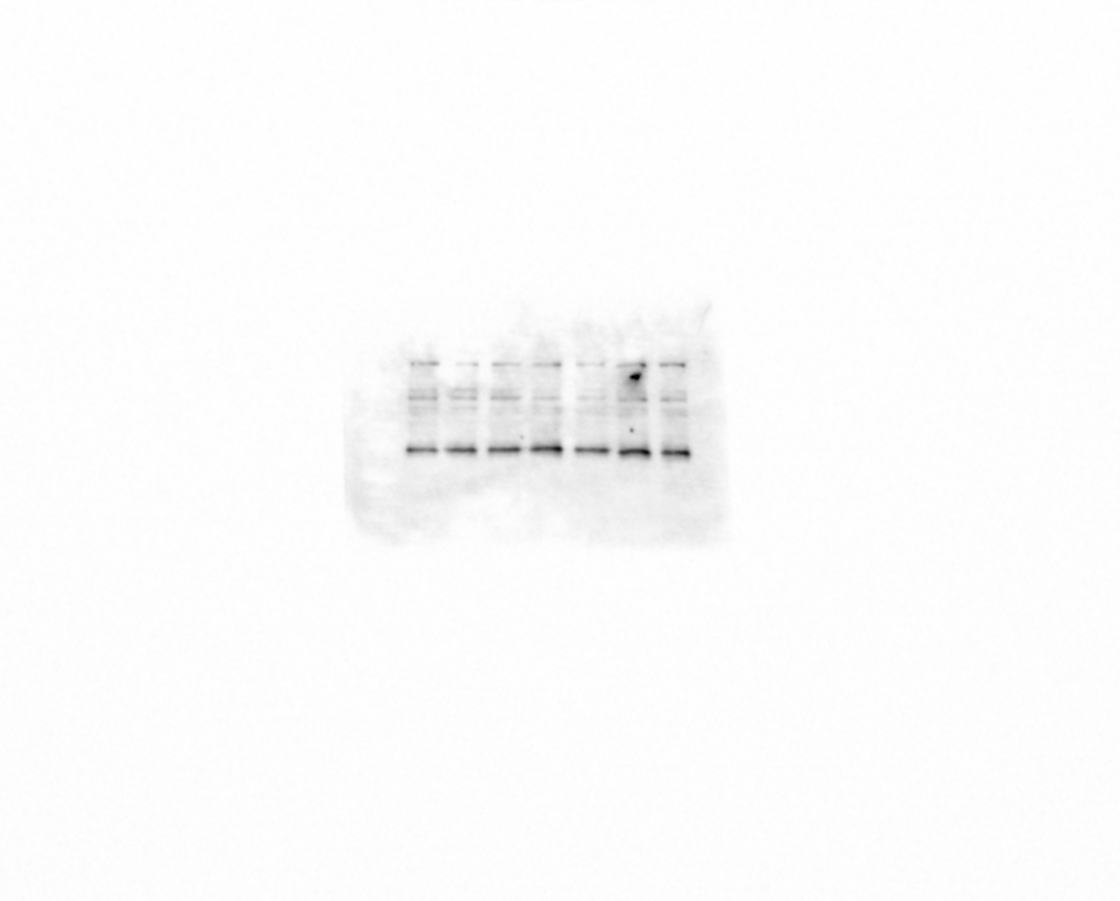

Supplement: Figure 6—source data 2. [file elife-100747-fig6-data2.zip › Figure 6 - Source Data 2 (original western files)/eIF2a-P-S51/23.01.20_15.13.23_S6_F05.tif]
