# Supplementary figures and images for: Glutamine catabolism supports amino acid biosynthesis and suppresses the integrated stress response to promote photoreceptor survival (part 4 of 4)
Source: eLife. 2025 May 21;13:RP100747. doi: 10.7554/eLife.100747 (PMC12094702; doi:10.7554/eLife.100747)

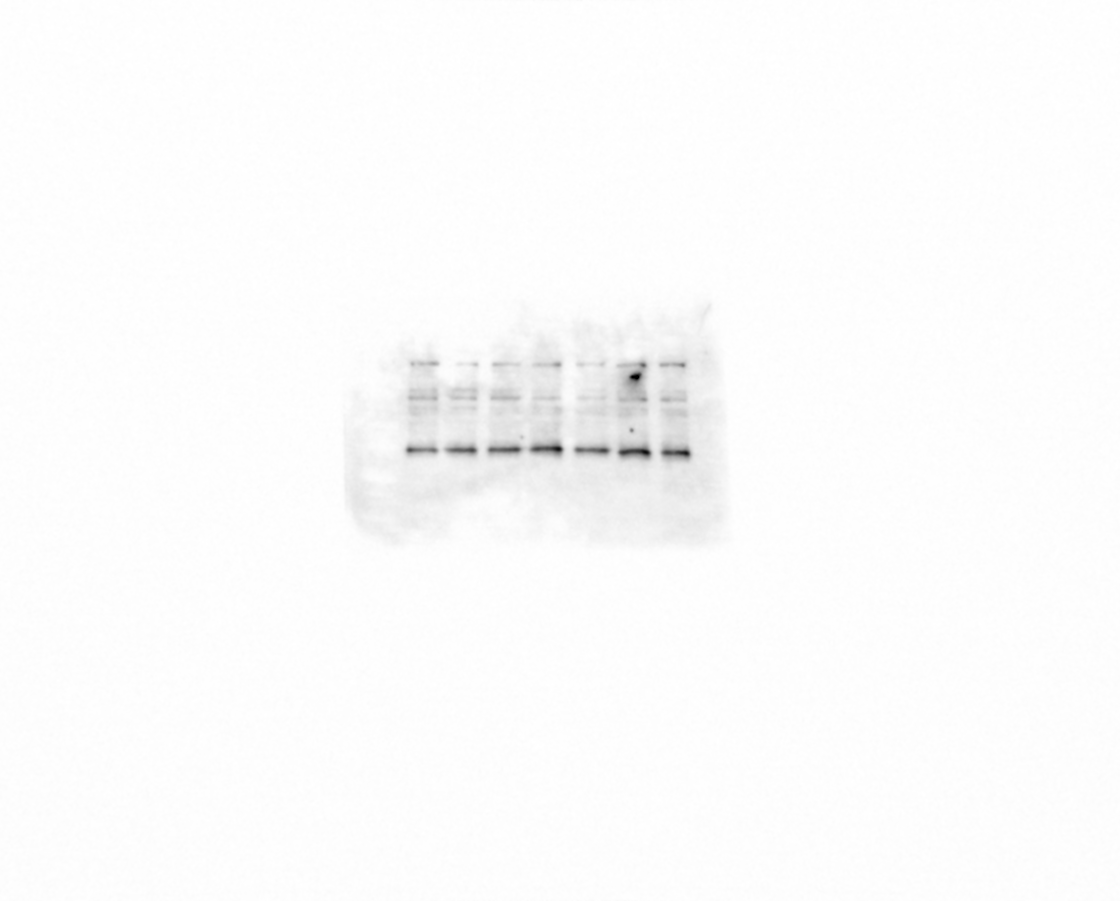

Supplement: Figure 6—source data 2. [file elife-100747-fig6-data2.zip › Figure 6 - Source Data 2 (original western files)/eIF2a-P-S51/23.01.20_15.13.23_S6_F06.tif]

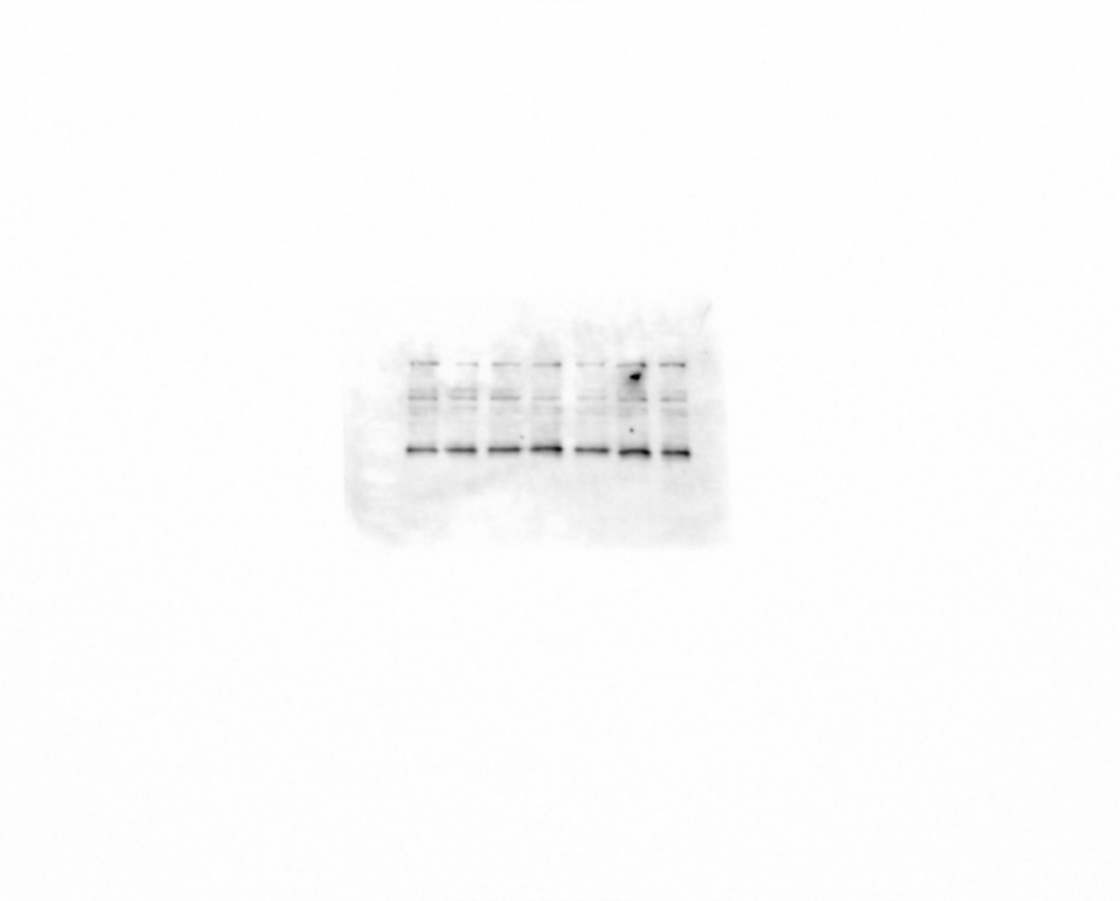

Supplement: Figure 6—source data 2. [file elife-100747-fig6-data2.zip › Figure 6 - Source Data 2 (original western files)/eIF2a-P-S51/23.01.20_15.13.23_S6_F07.tif]

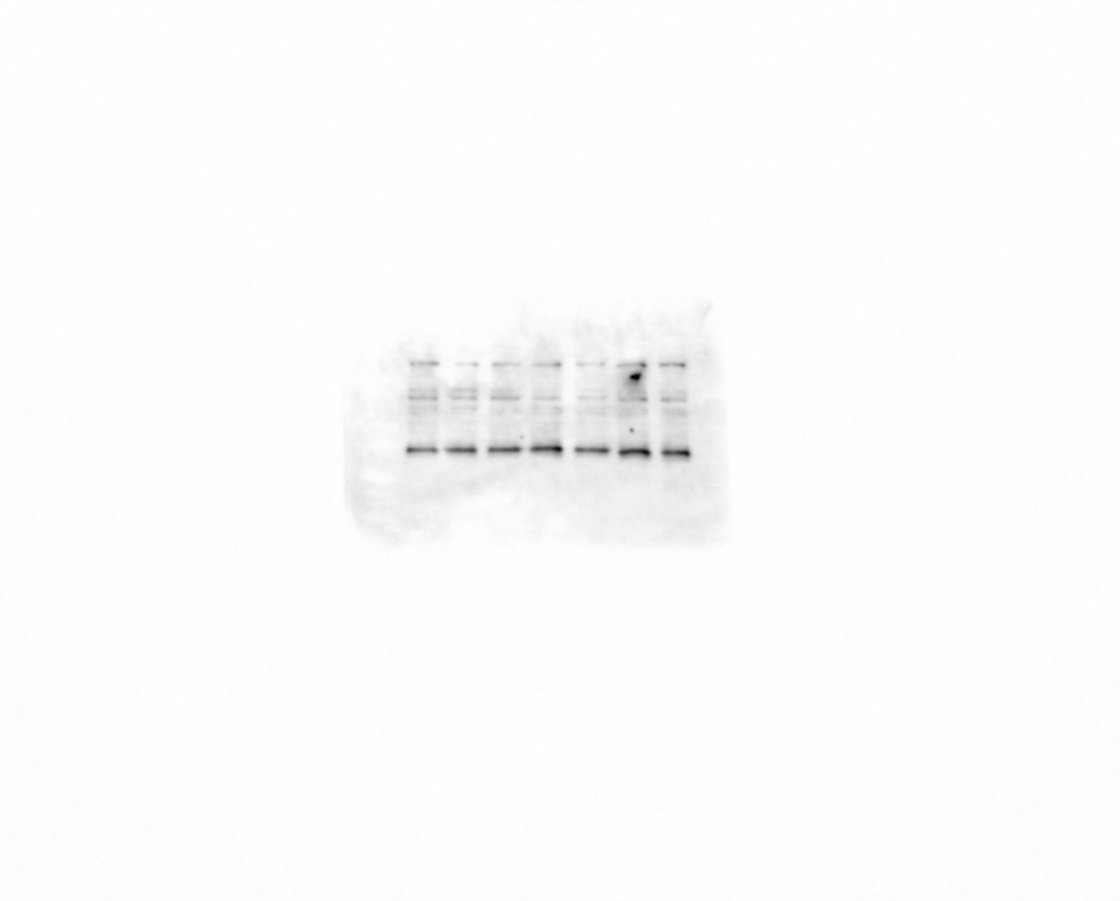

Supplement: Figure 6—source data 2. [file elife-100747-fig6-data2.zip › Figure 6 - Source Data 2 (original western files)/eIF2a-P-S51/23.01.20_15.13.23_S6_F08.tif]

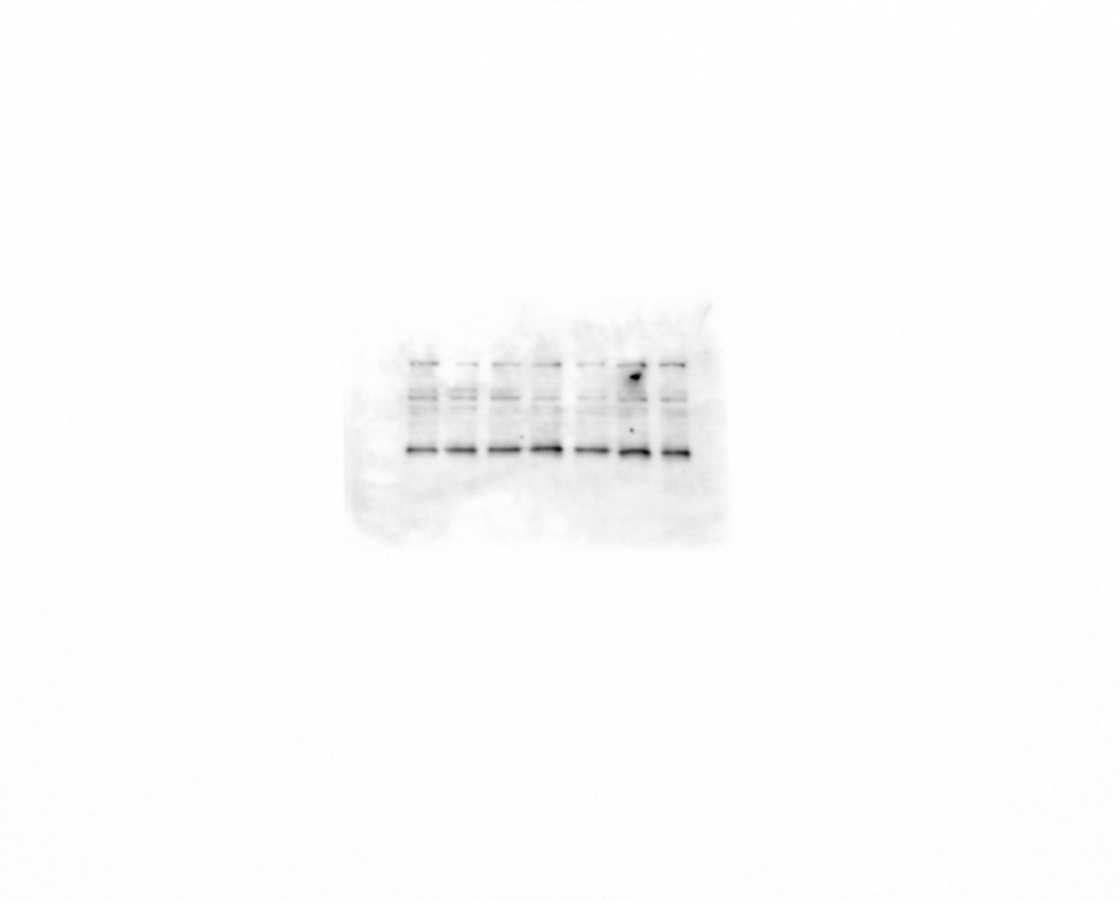

Supplement: Figure 6—source data 2. [file elife-100747-fig6-data2.zip › Figure 6 - Source Data 2 (original western files)/eIF2a-P-S51/23.01.20_15.13.23_S6_F09.tif]

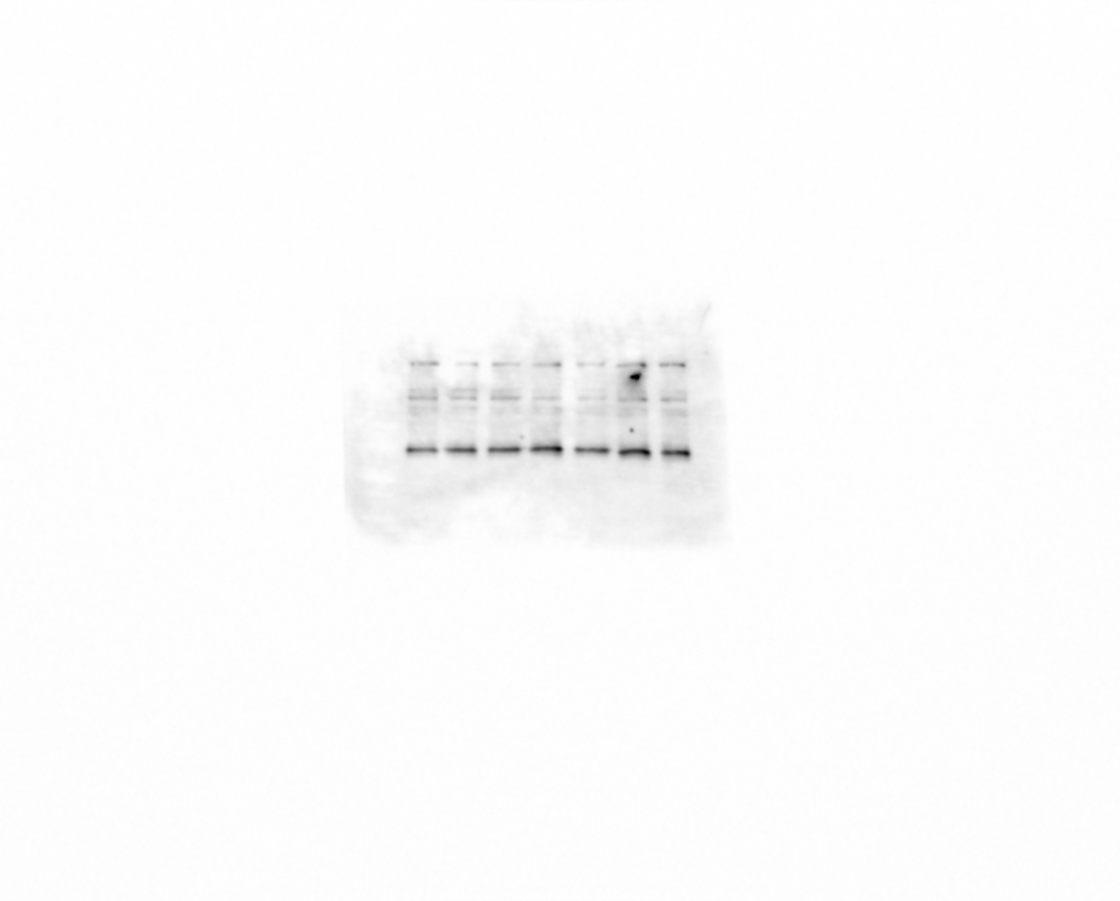

Supplement: Figure 6—source data 2. [file elife-100747-fig6-data2.zip › Figure 6 - Source Data 2 (original western files)/eIF2a-P-S51/23.01.20_15.13.23_S6_F10.tif]

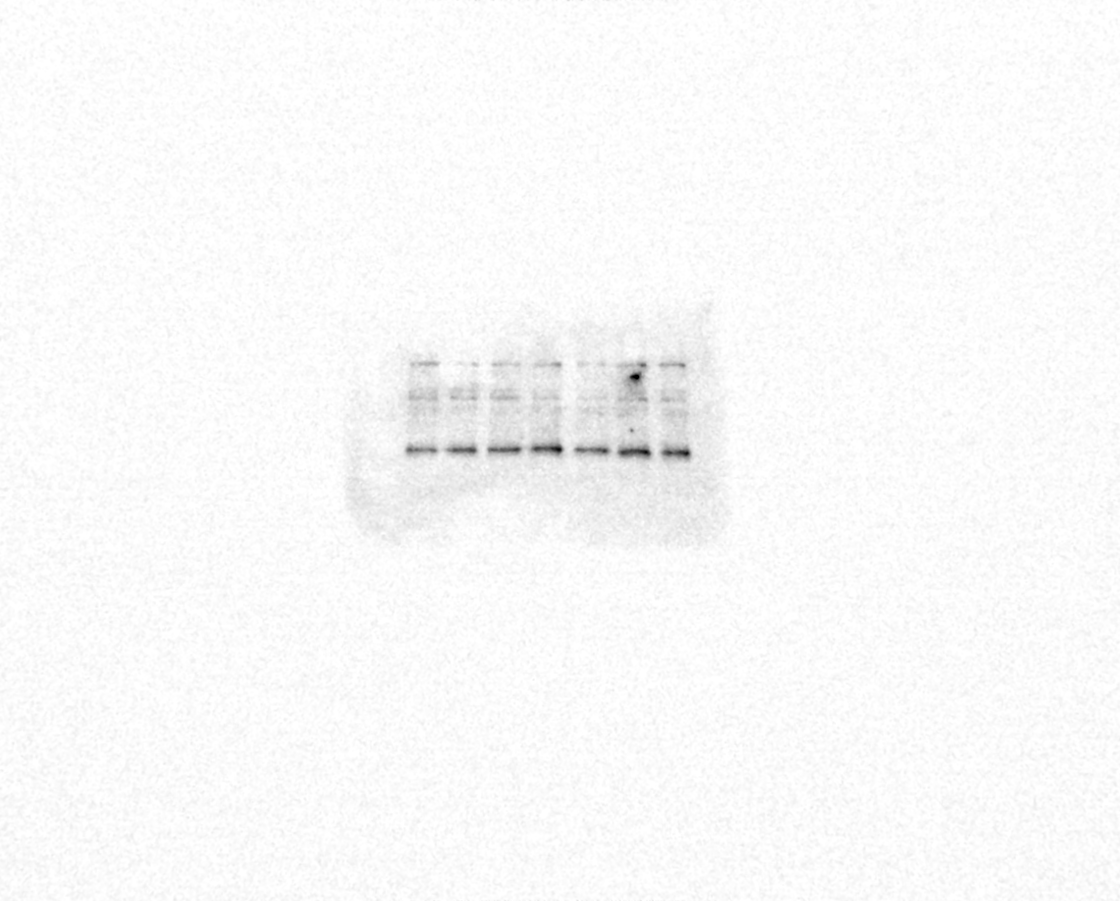

Supplement: Figure 6—source data 2. [file elife-100747-fig6-data2.zip › Figure 6 - Source Data 2 (original western files)/eIF2a-P-S51/23.01.20_15.14.25_S7_F01.tif]

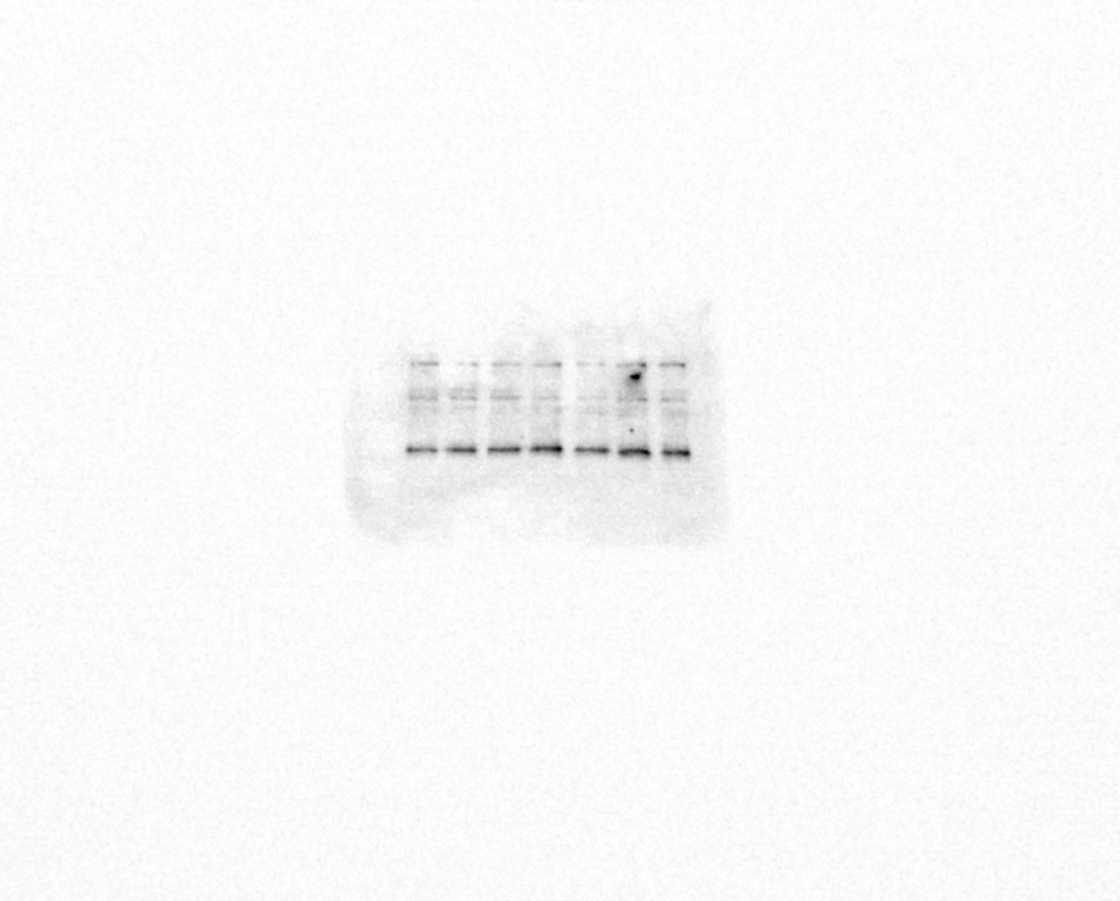

Supplement: Figure 6—source data 2. [file elife-100747-fig6-data2.zip › Figure 6 - Source Data 2 (original western files)/eIF2a-P-S51/23.01.20_15.14.25_S7_F02.tif]

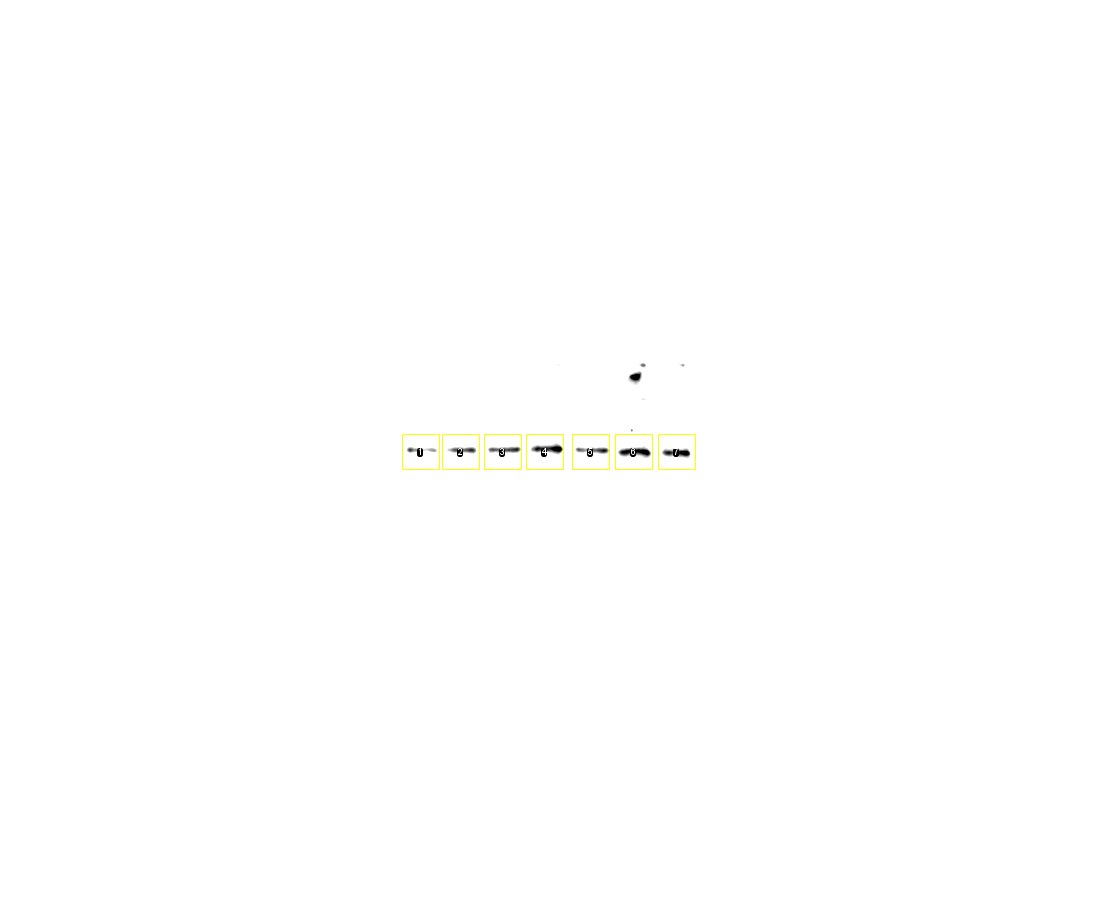

Supplement: Figure 6—source data 2. [file elife-100747-fig6-data2.zip › Figure 6 - Source Data 2 (original western files)/eIF2a-P-S51/23.01.20_15.14.25_S7_F03.jpg]

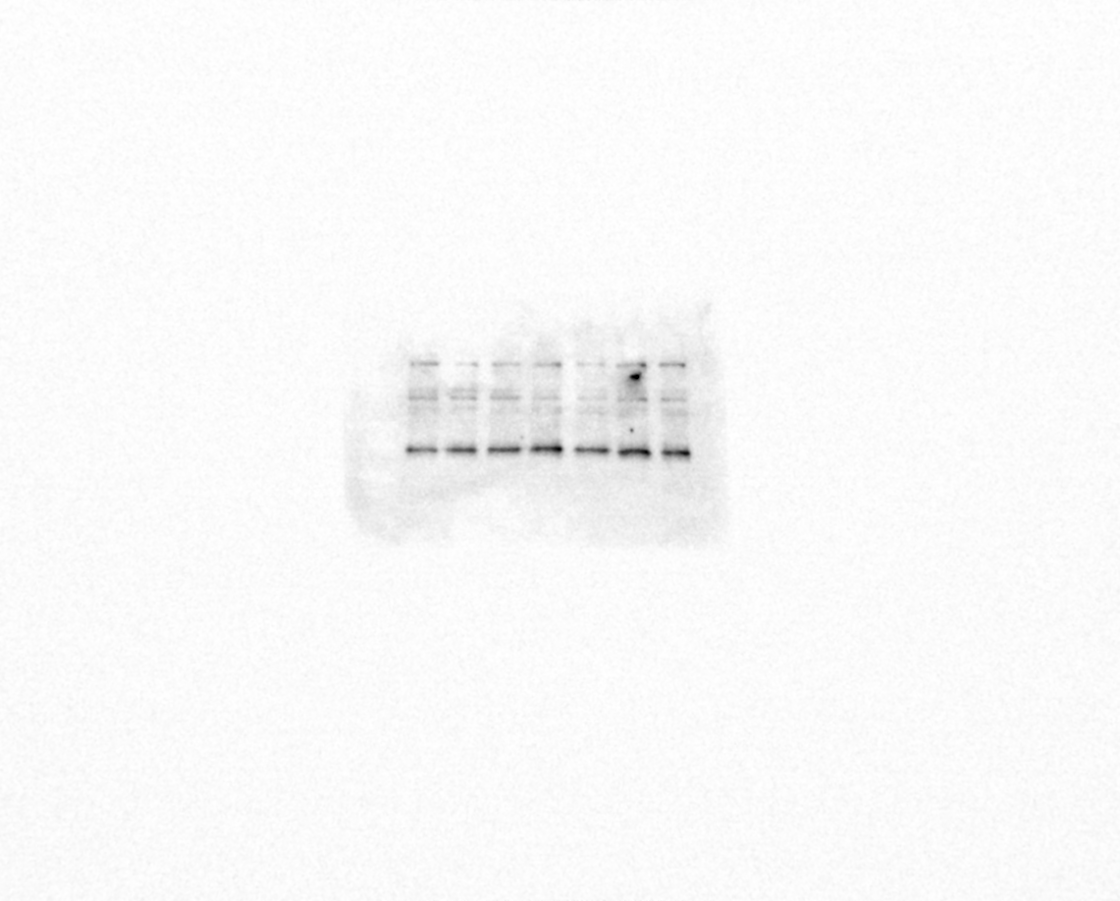

Supplement: Figure 6—source data 2. [file elife-100747-fig6-data2.zip › Figure 6 - Source Data 2 (original western files)/eIF2a-P-S51/23.01.20_15.14.25_S7_F03.tif]

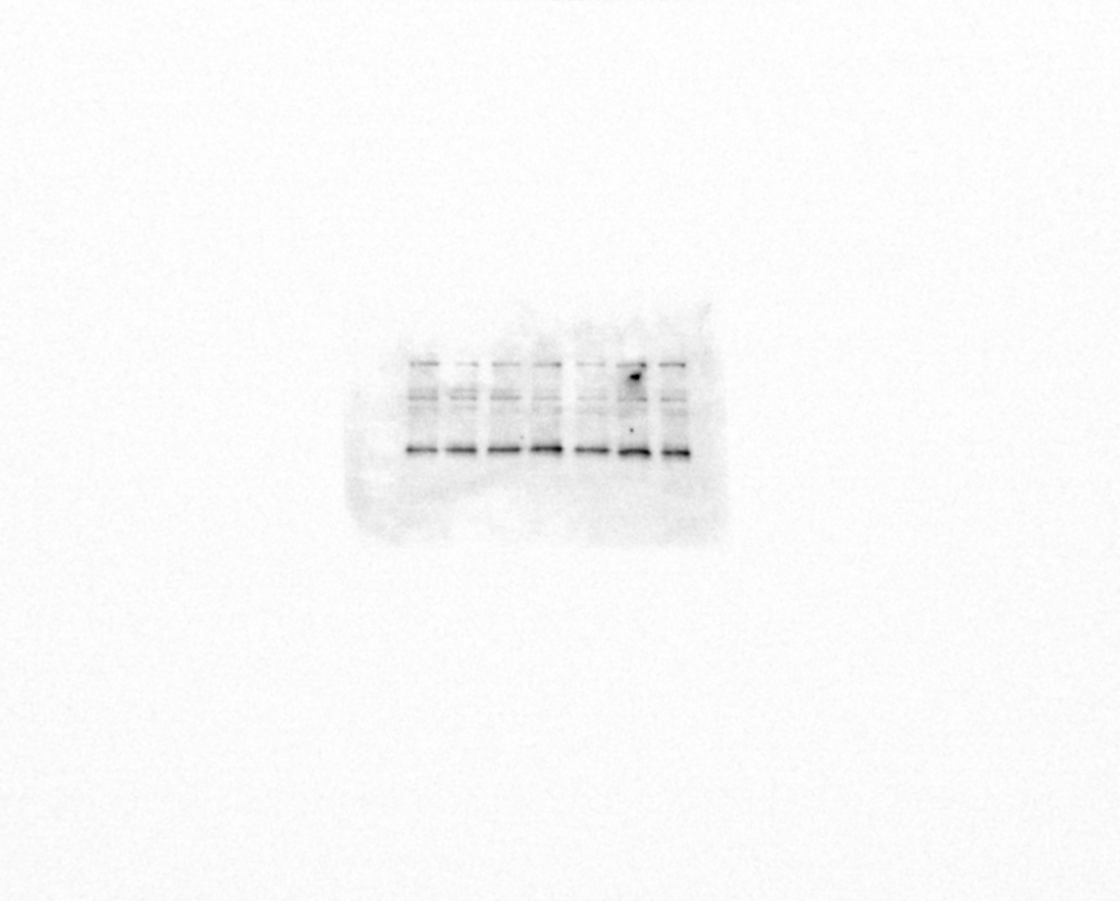

Supplement: Figure 6—source data 2. [file elife-100747-fig6-data2.zip › Figure 6 - Source Data 2 (original western files)/eIF2a-P-S51/23.01.20_15.14.25_S7_F04.tif]

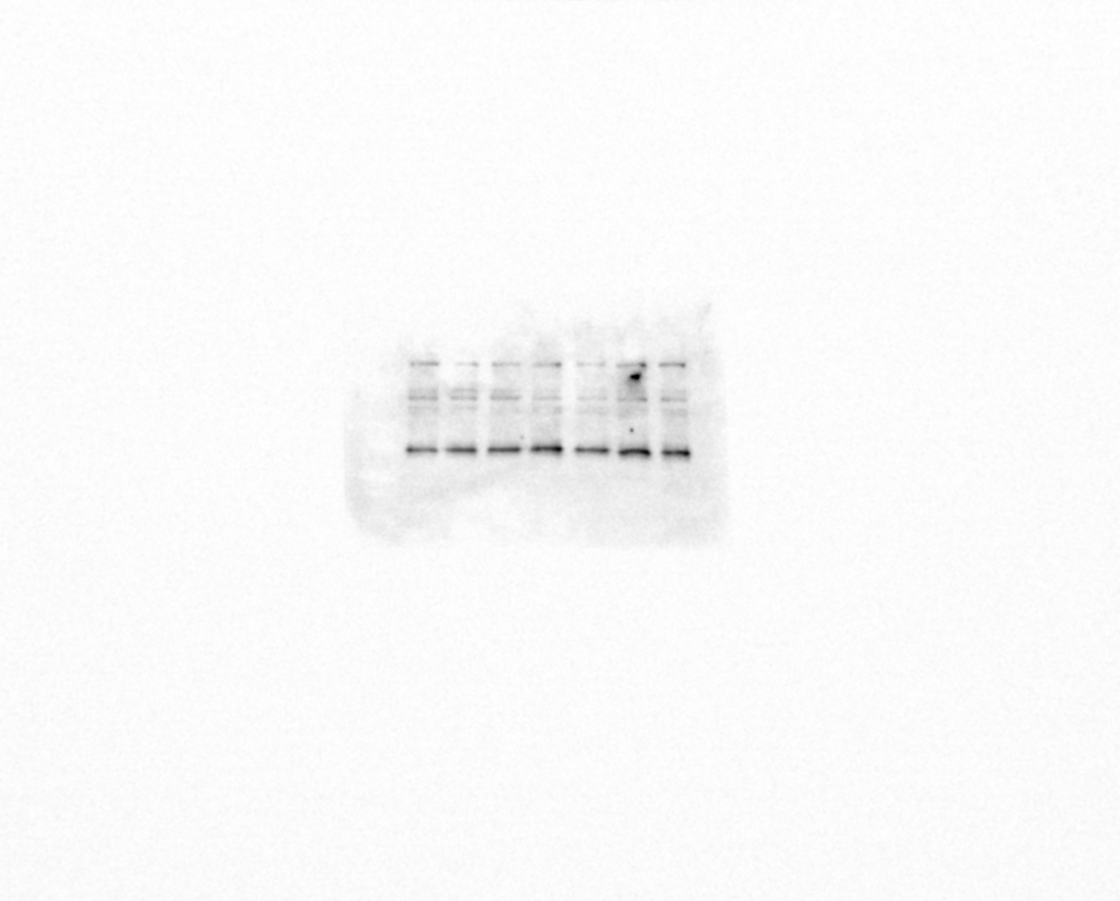

Supplement: Figure 6—source data 2. [file elife-100747-fig6-data2.zip › Figure 6 - Source Data 2 (original western files)/eIF2a-P-S51/23.01.20_15.14.25_S7_F05.tif]

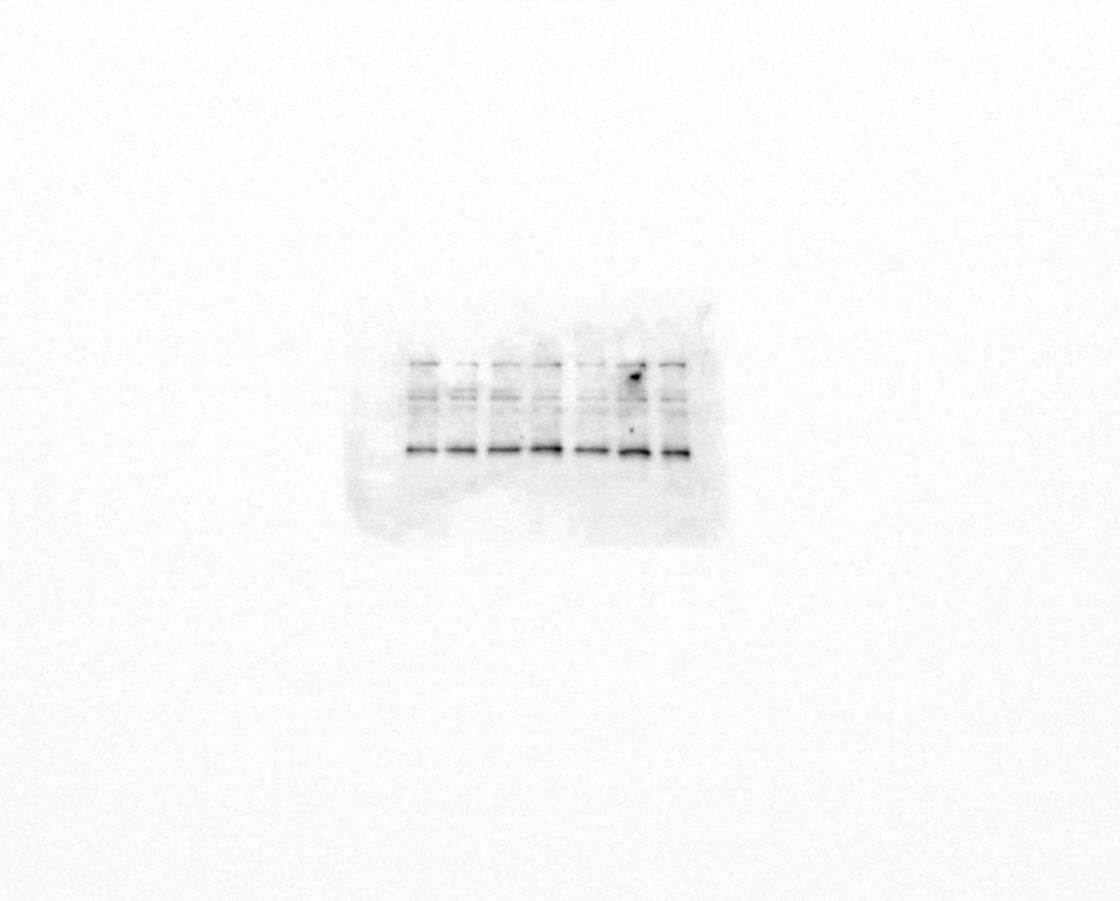

Supplement: Figure 6—source data 2. [file elife-100747-fig6-data2.zip › Figure 6 - Source Data 2 (original western files)/eIF2a-P-S51/23.01.20_15.14.25_S7_F06.tif]

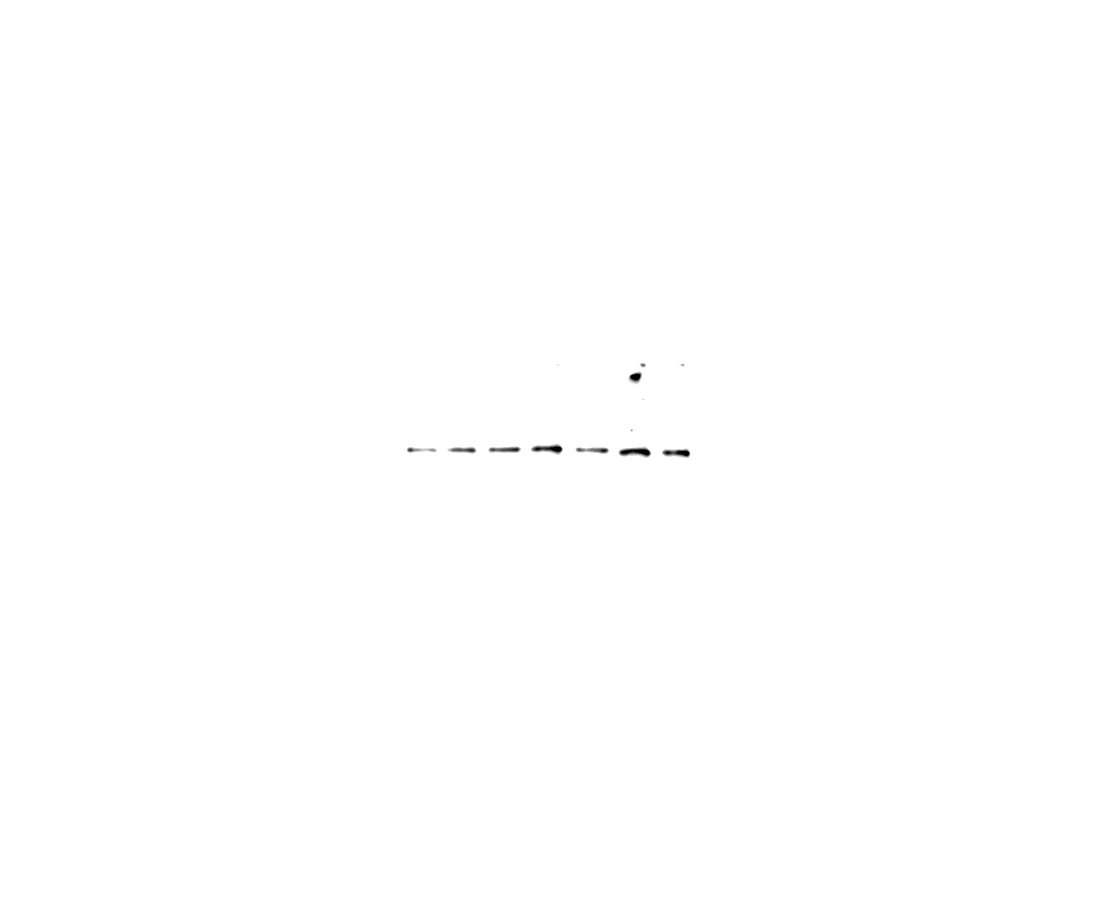

Supplement: Figure 6—source data 2. [file elife-100747-fig6-data2.zip › Figure 6 - Source Data 2 (original western files)/eIF2a-P-S51/23.01.20_15.14.25_S7_F07.jpg]

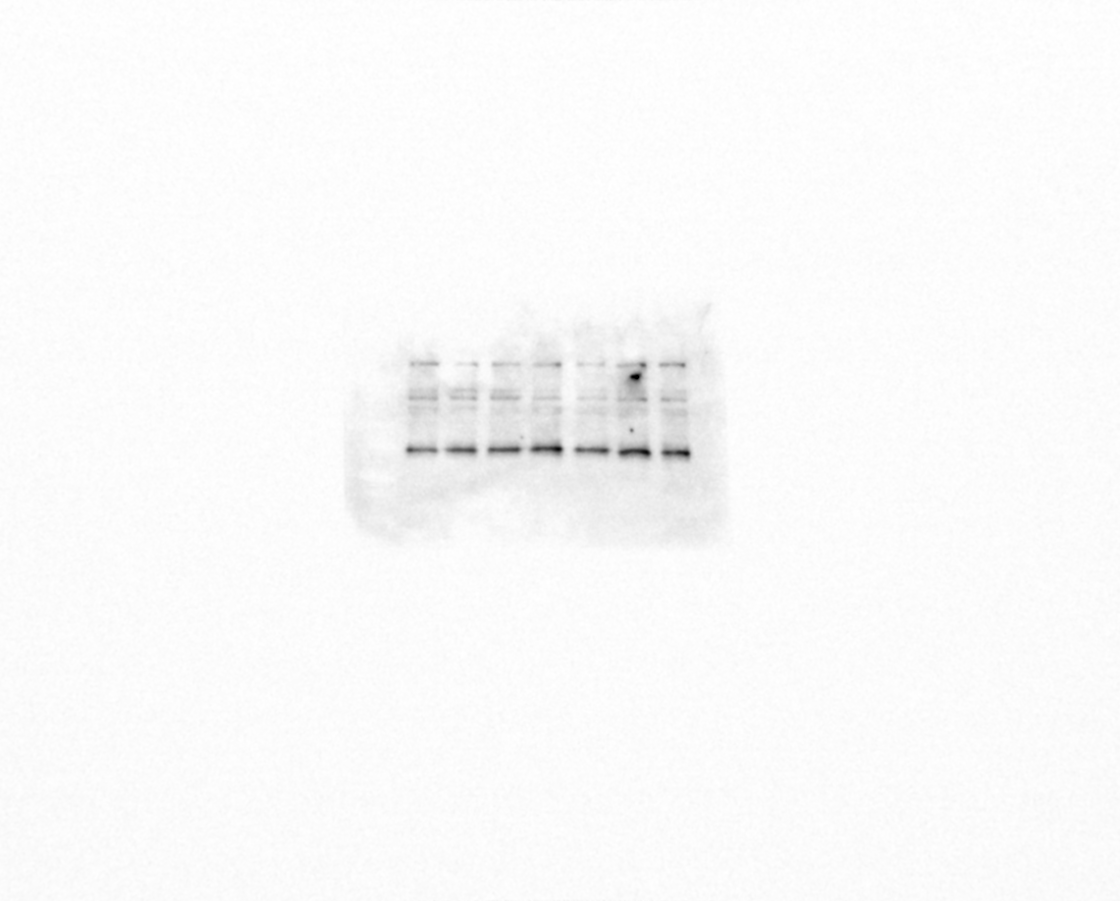

Supplement: Figure 6—source data 2. [file elife-100747-fig6-data2.zip › Figure 6 - Source Data 2 (original western files)/eIF2a-P-S51/23.01.20_15.14.25_S7_F07.tif]

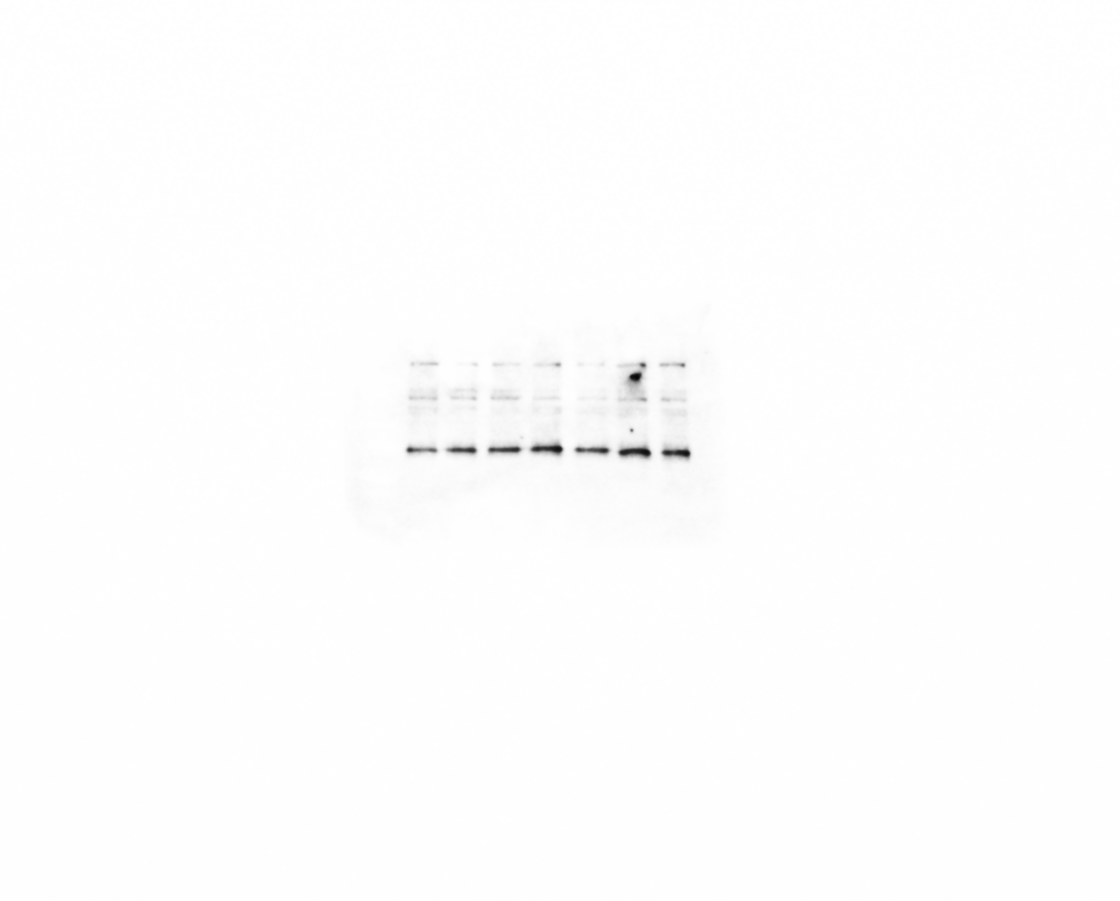

Supplement: Figure 6—source data 2. [file elife-100747-fig6-data2.zip › Figure 6 - Source Data 2 (original western files)/eIF2a-P-S51/23.01.20_15.14.25_S7_F08.tif]

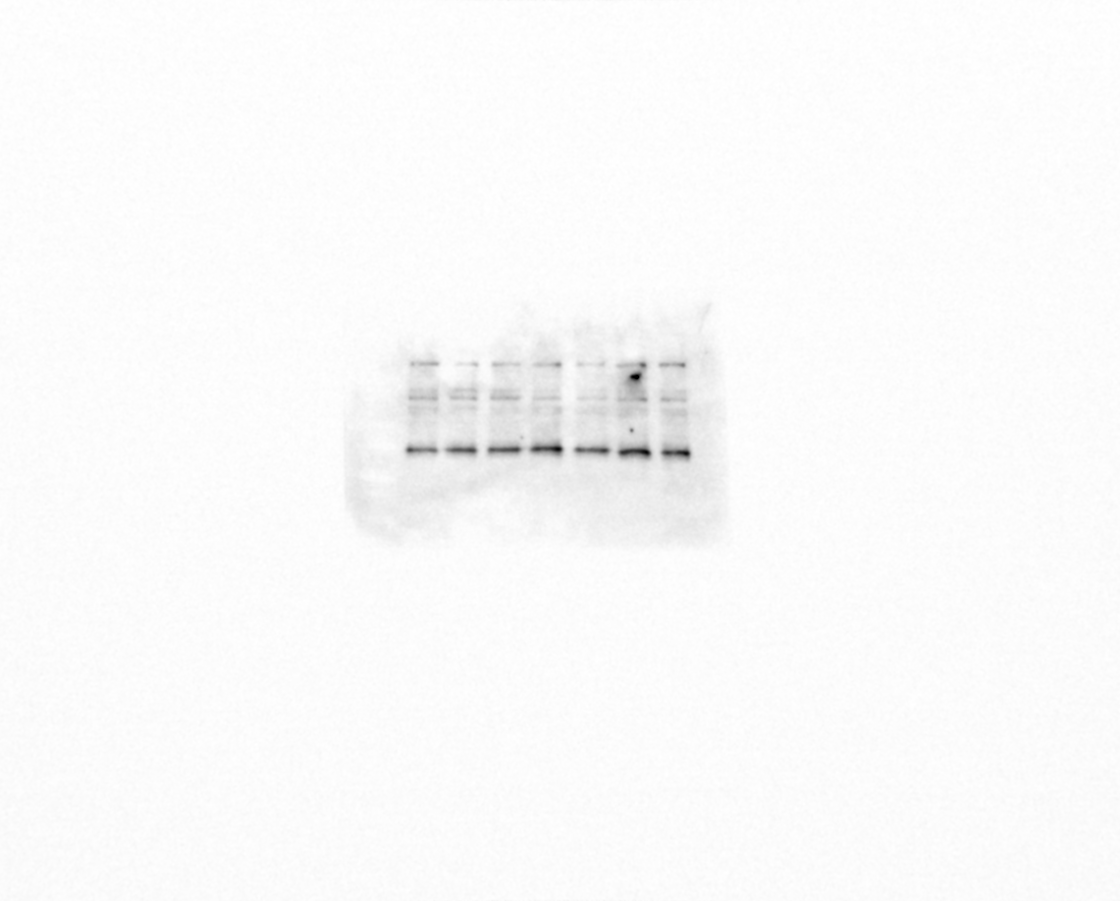

Supplement: Figure 6—source data 2. [file elife-100747-fig6-data2.zip › Figure 6 - Source Data 2 (original western files)/eIF2a-P-S51/23.01.20_15.14.25_S7_F09.tif]

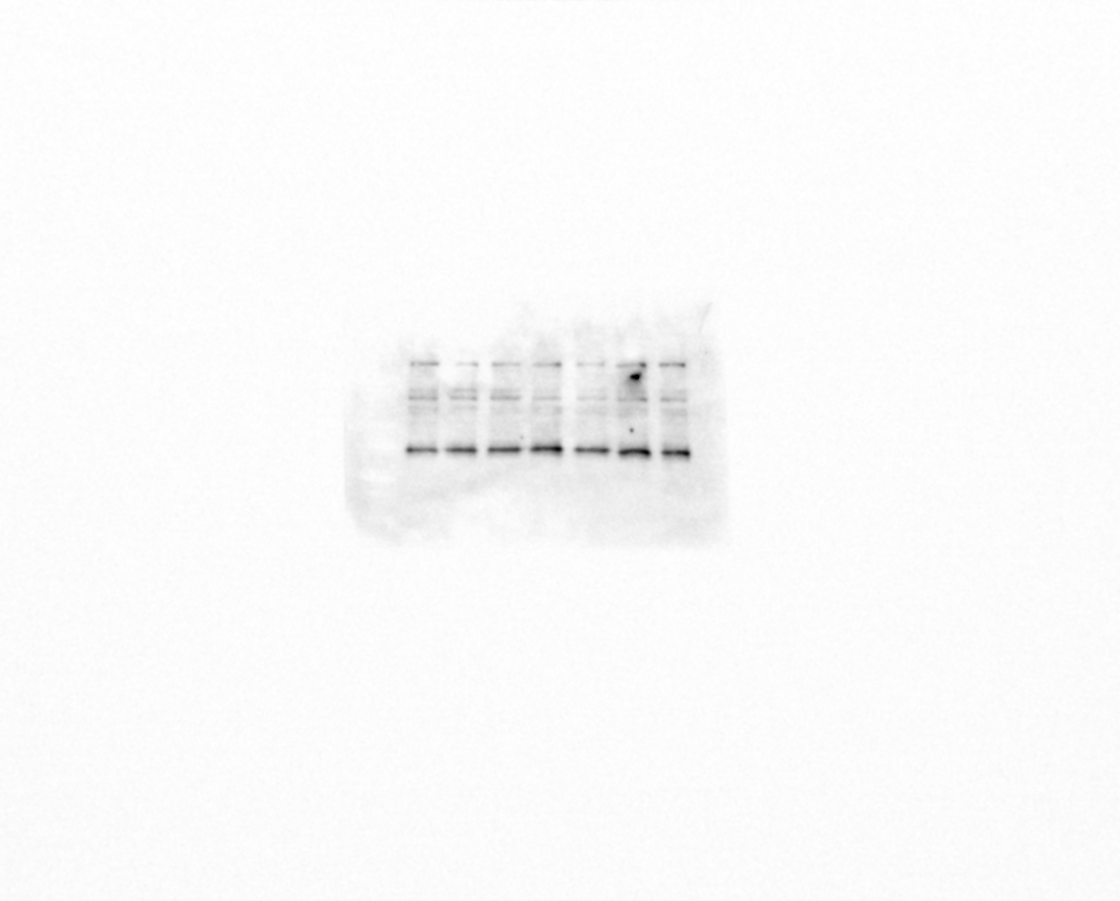

Supplement: Figure 6—source data 2. [file elife-100747-fig6-data2.zip › Figure 6 - Source Data 2 (original western files)/eIF2a-P-S51/23.01.20_15.14.25_S7_F10.tif]

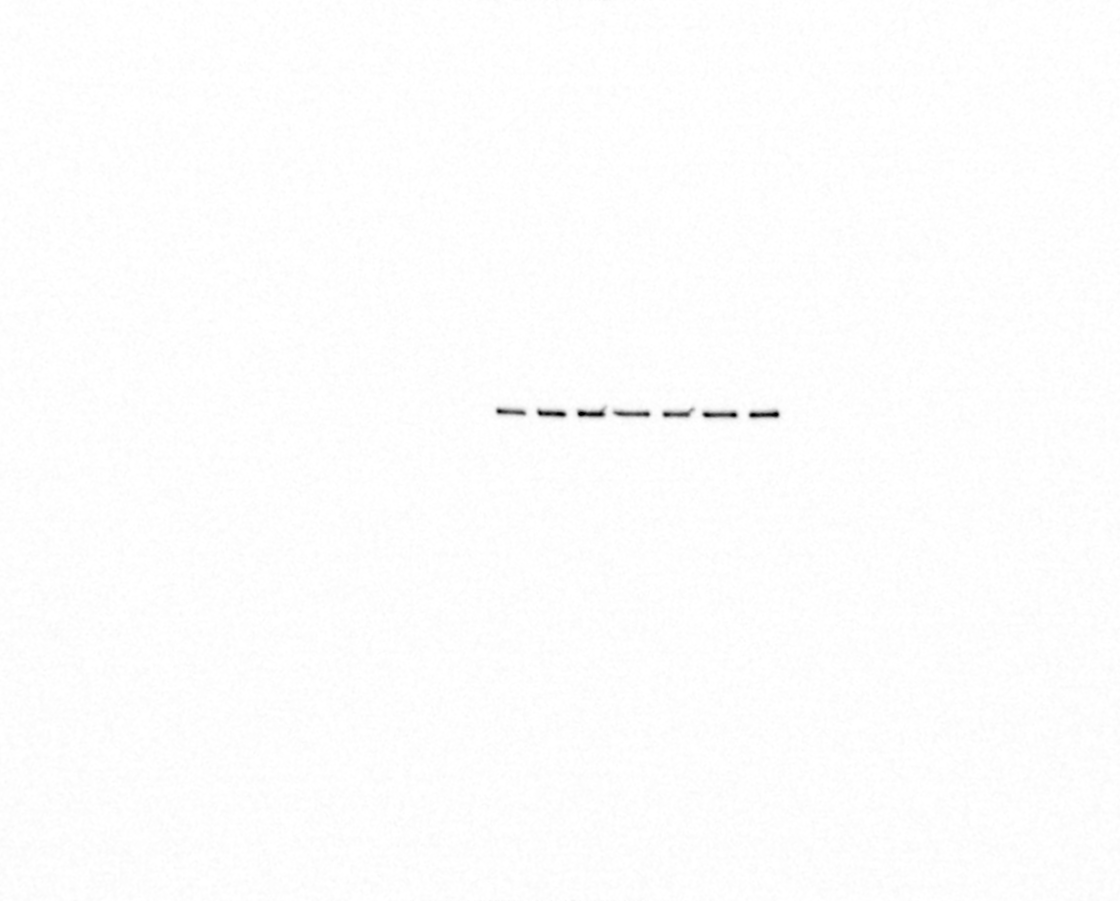

Supplement: Figure 6—source data 2. [file elife-100747-fig6-data2.zip › Figure 6 - Source Data 2 (original western files)/hsp90/23.01.25_12.04.30.tif]

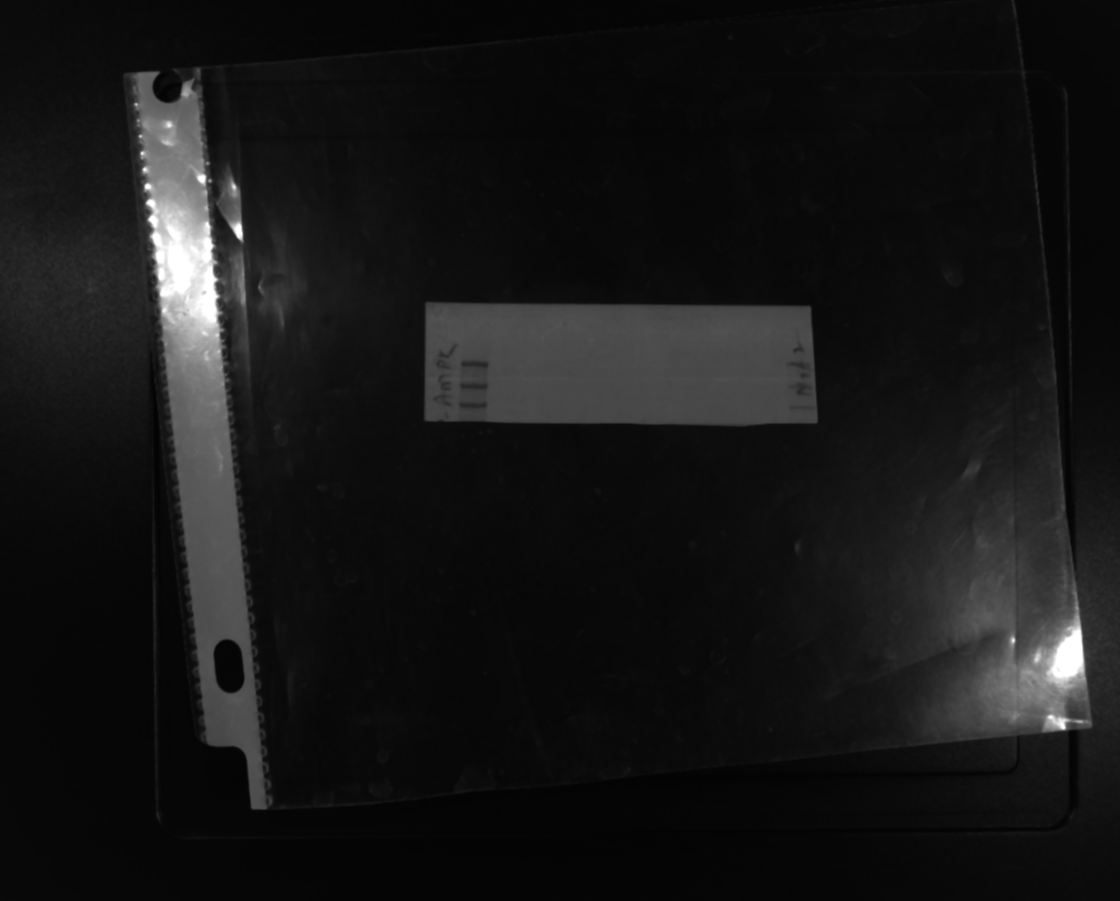

Supplement: Figure 6—source data 2. [file elife-100747-fig6-data2.zip › Figure 6 - Source Data 2 (original western files)/hsp90/23.01.25_12.04.57.tif]

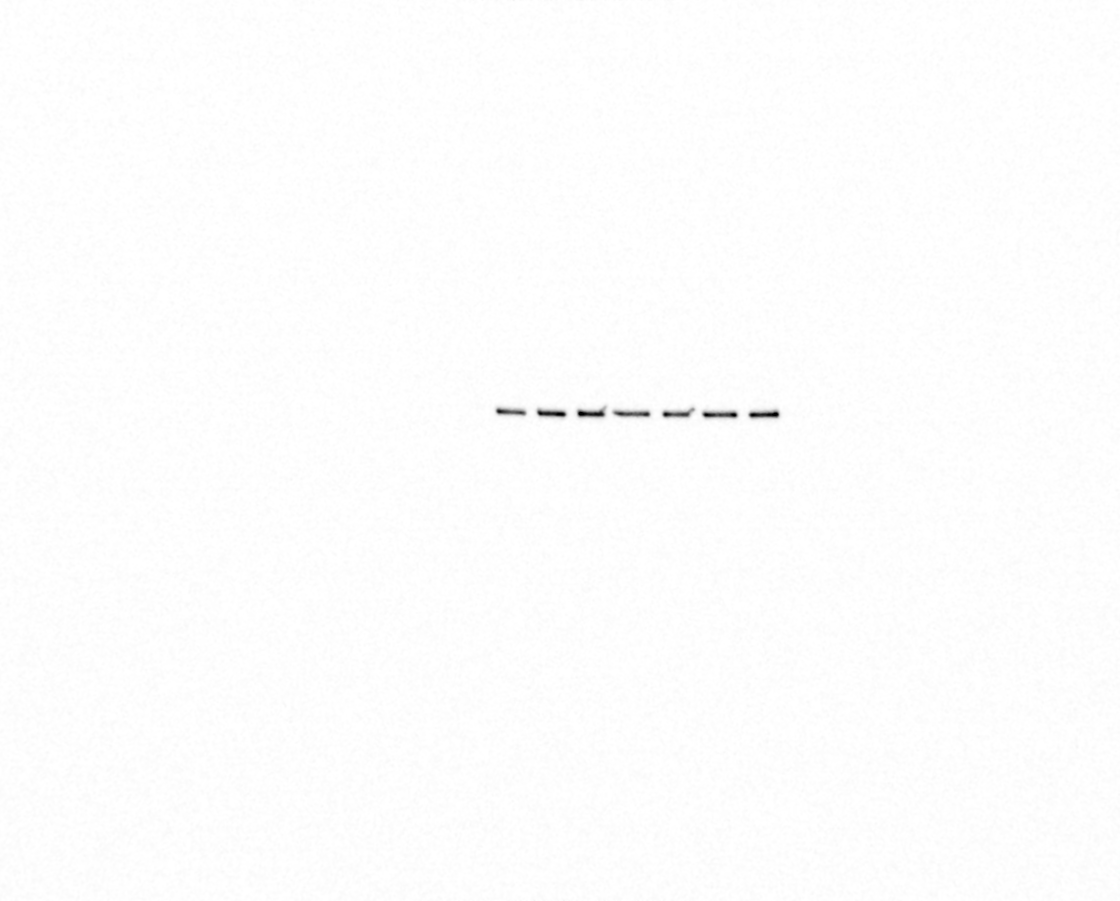

Supplement: Figure 6—source data 2. [file elife-100747-fig6-data2.zip › Figure 6 - Source Data 2 (original western files)/hsp90/23.01.25_12.05.21_S2_F01.tif]

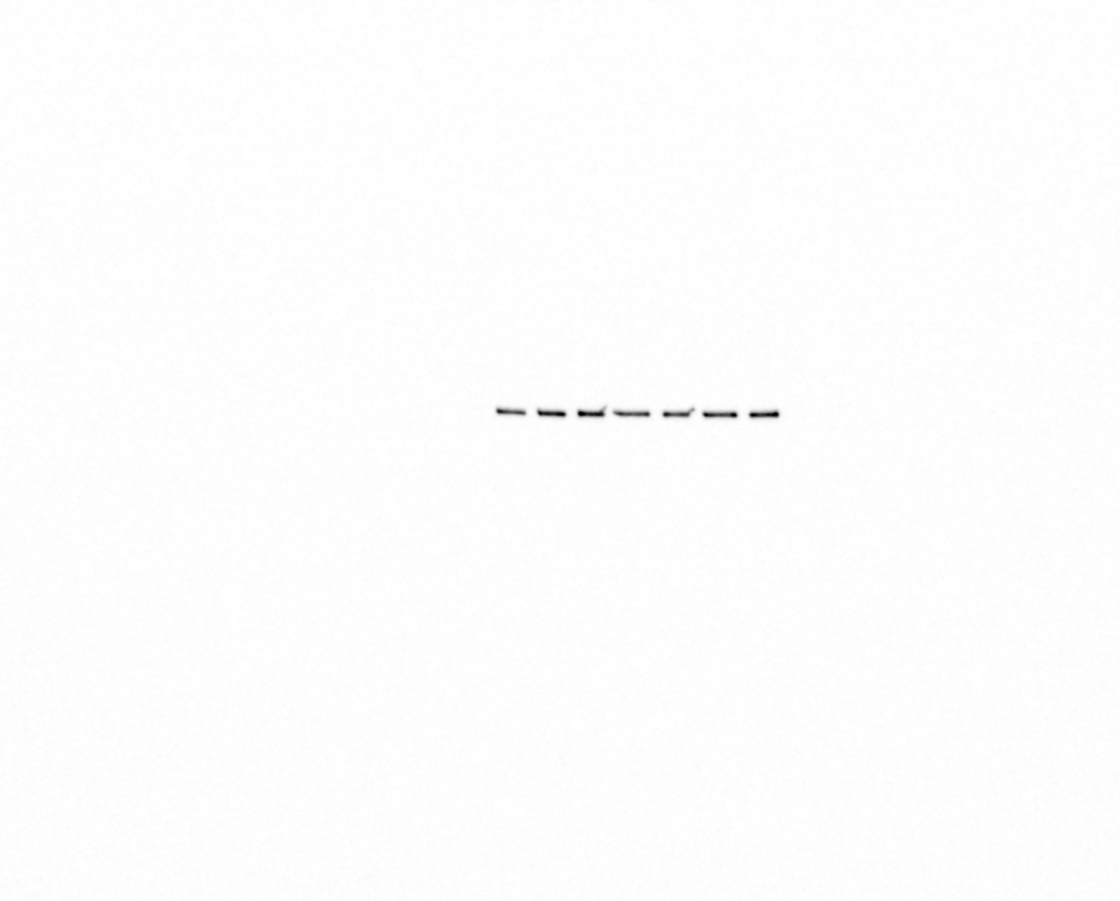

Supplement: Figure 6—source data 2. [file elife-100747-fig6-data2.zip › Figure 6 - Source Data 2 (original western files)/hsp90/23.01.25_12.05.21_S2_F02.tif]

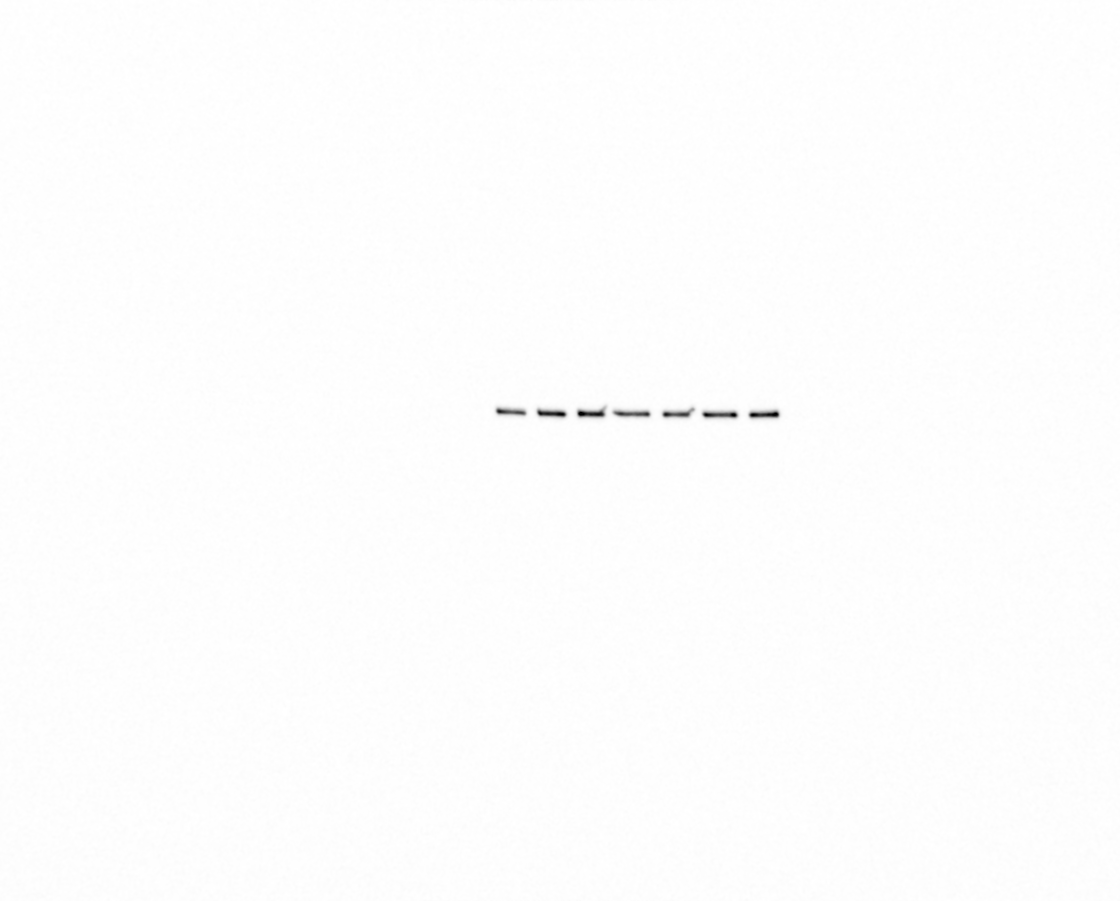

Supplement: Figure 6—source data 2. [file elife-100747-fig6-data2.zip › Figure 6 - Source Data 2 (original western files)/hsp90/23.01.25_12.05.21_S2_F03.tif]

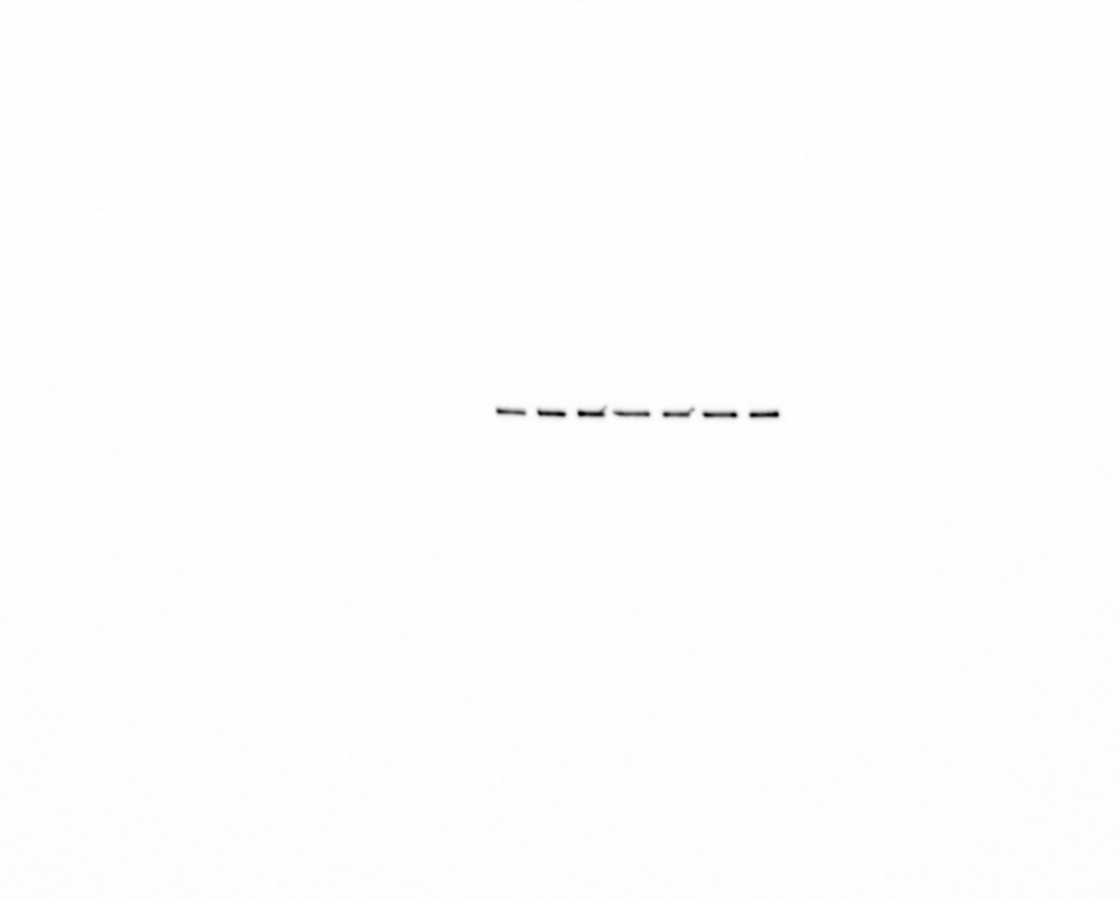

Supplement: Figure 6—source data 2. [file elife-100747-fig6-data2.zip › Figure 6 - Source Data 2 (original western files)/hsp90/23.01.25_12.05.21_S2_F04.tif]

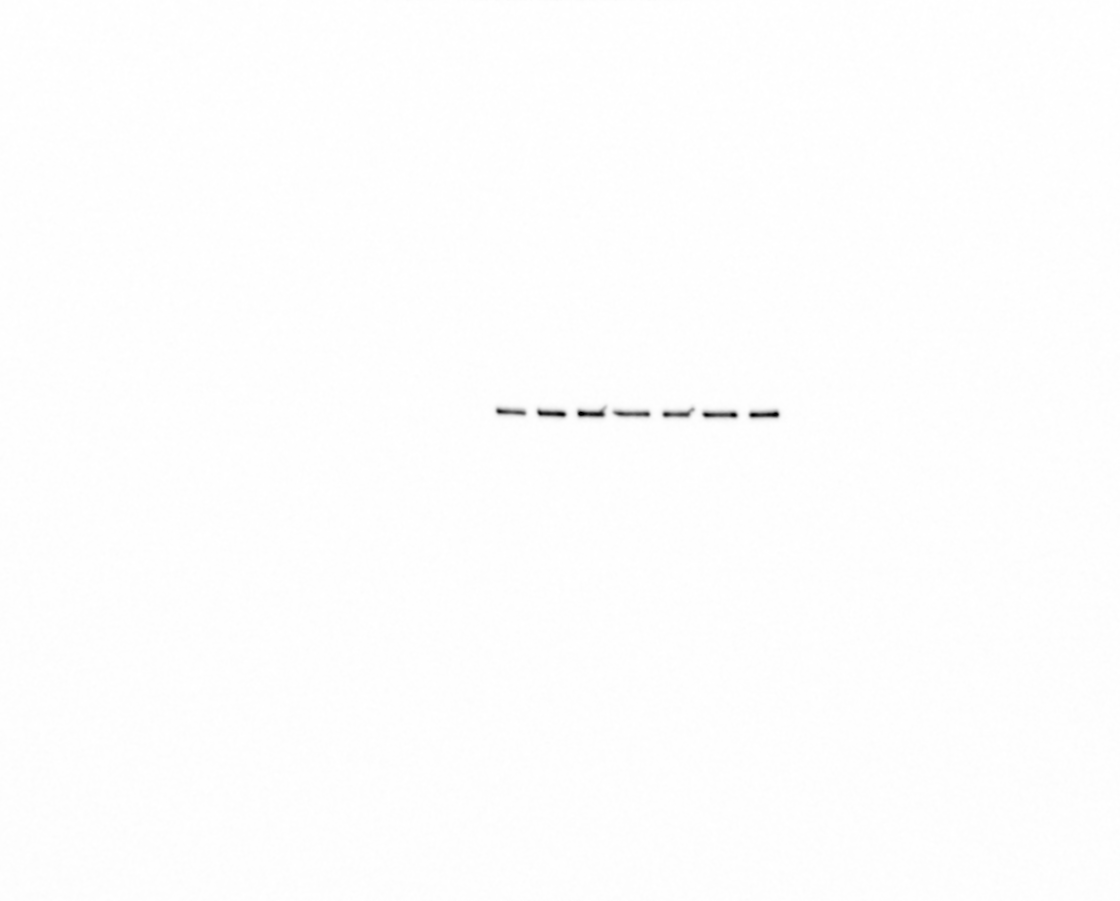

Supplement: Figure 6—source data 2. [file elife-100747-fig6-data2.zip › Figure 6 - Source Data 2 (original western files)/hsp90/23.01.25_12.05.21_S2_F05.tif]

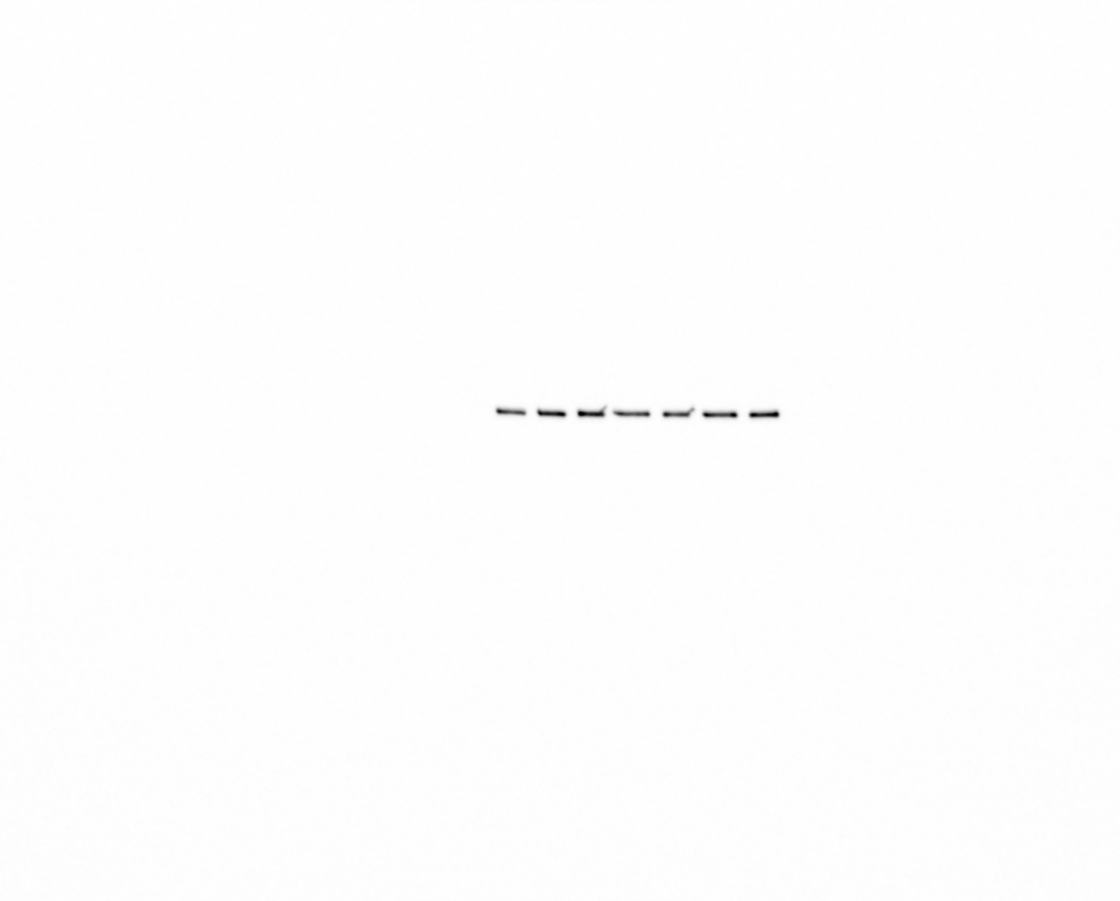

Supplement: Figure 6—source data 2. [file elife-100747-fig6-data2.zip › Figure 6 - Source Data 2 (original western files)/hsp90/23.01.25_12.05.21_S2_F06.tif]

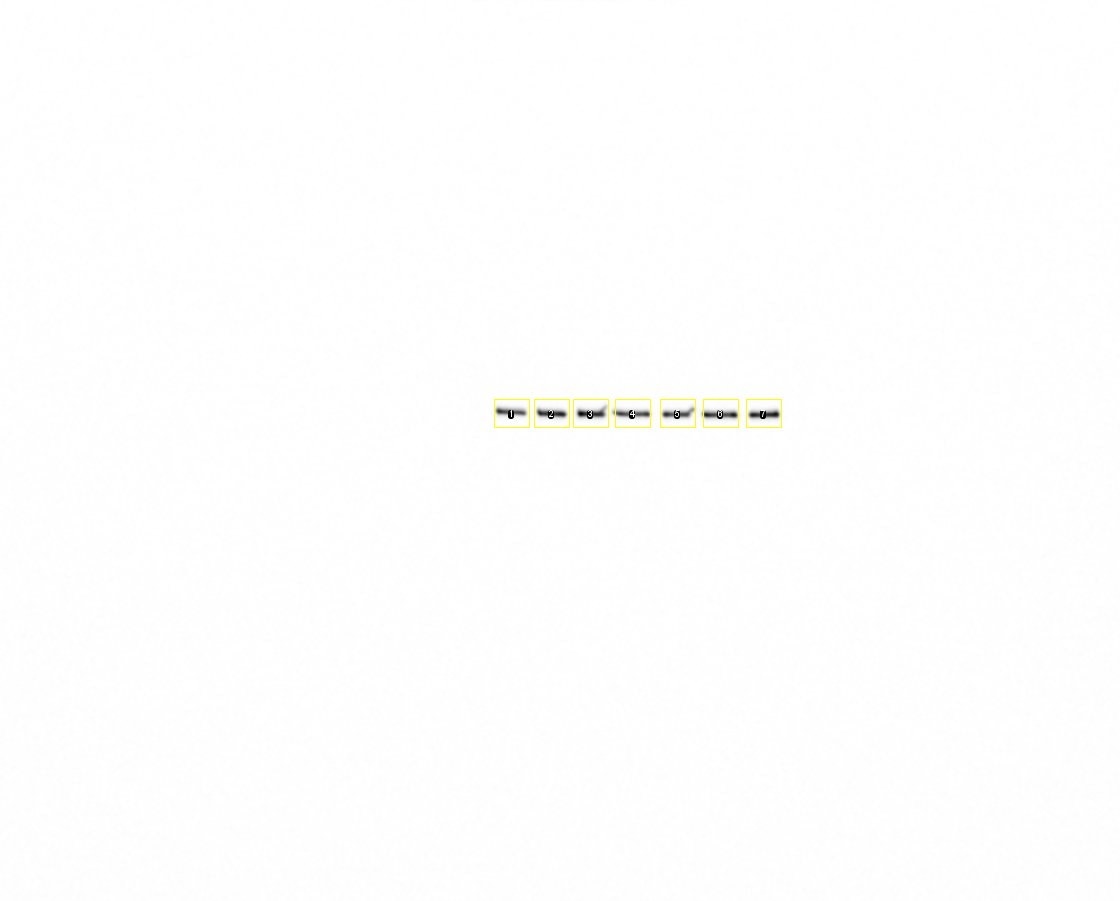

Supplement: Figure 6—source data 2. [file elife-100747-fig6-data2.zip › Figure 6 - Source Data 2 (original western files)/hsp90/23.01.25_12.05.21_S2_F07.jpg]

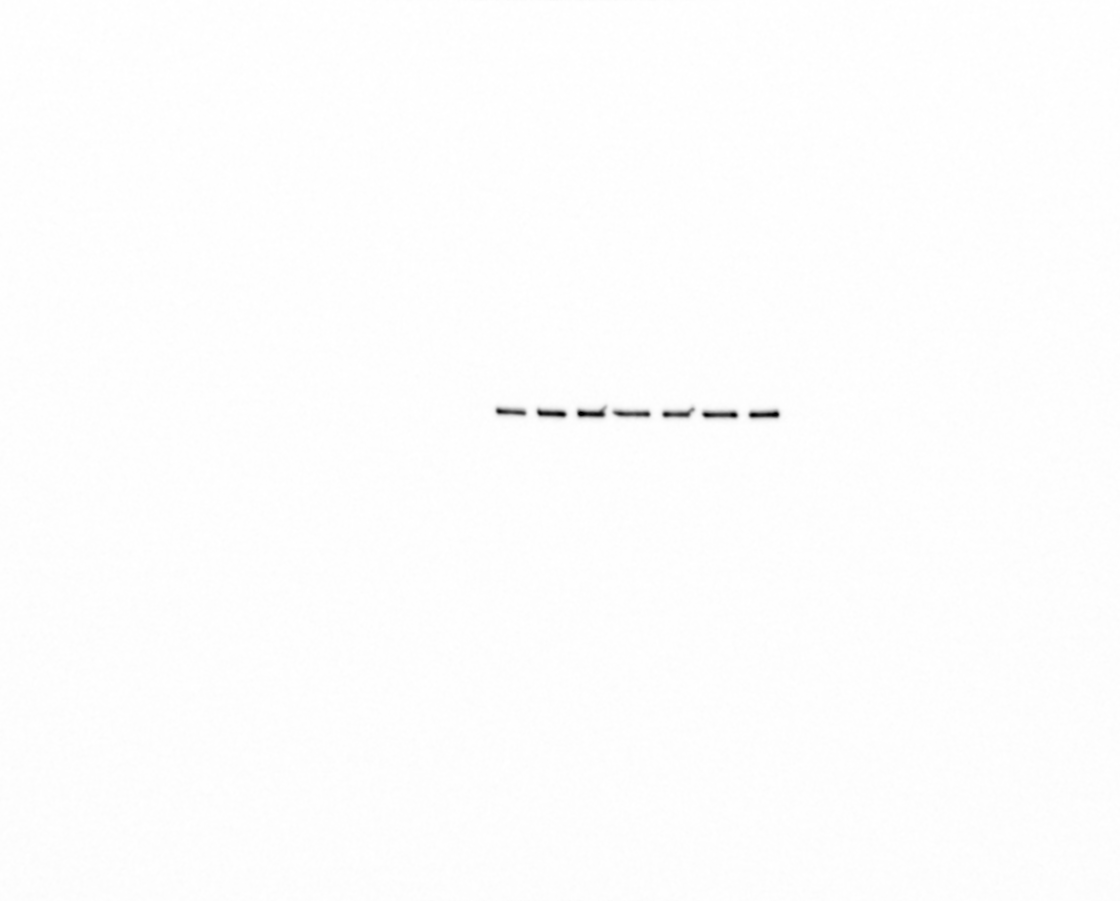

Supplement: Figure 6—source data 2. [file elife-100747-fig6-data2.zip › Figure 6 - Source Data 2 (original western files)/hsp90/23.01.25_12.05.21_S2_F07.tif]

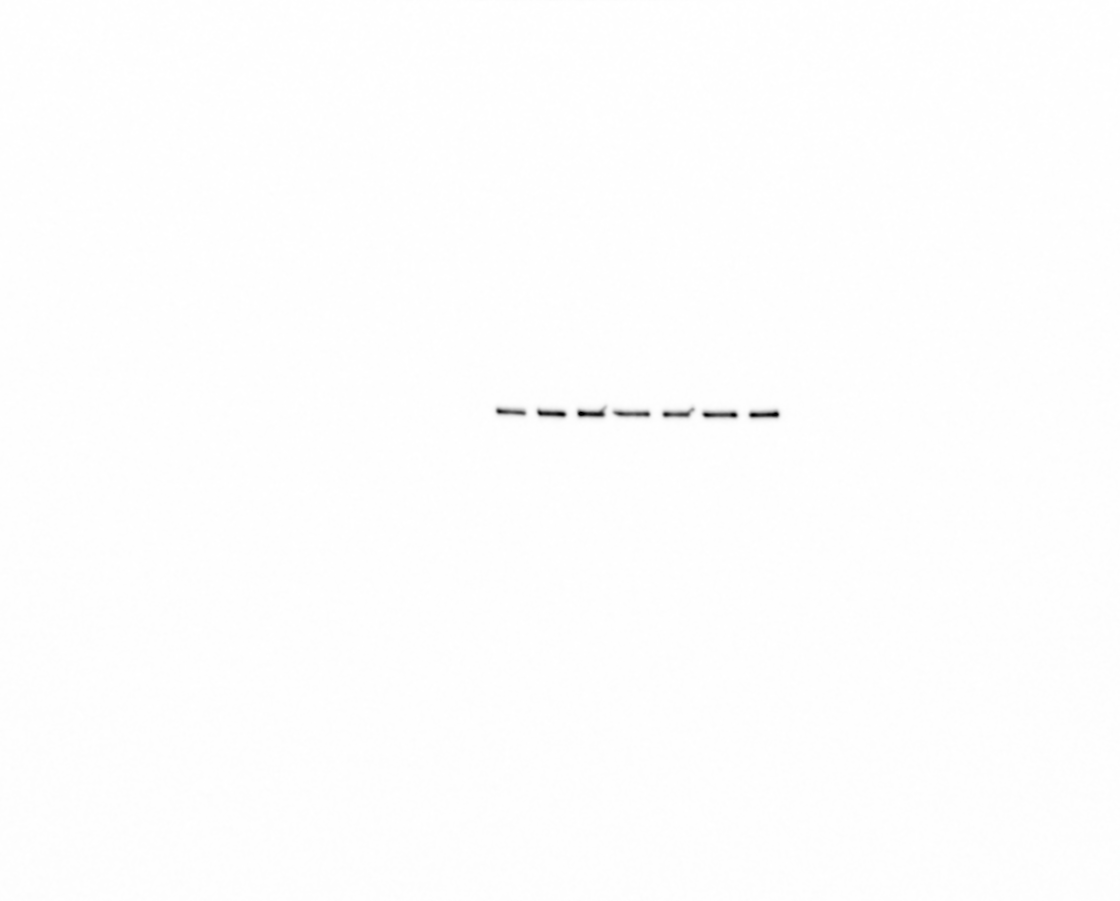

Supplement: Figure 6—source data 2. [file elife-100747-fig6-data2.zip › Figure 6 - Source Data 2 (original western files)/hsp90/23.01.25_12.05.21_S2_F08.tif]

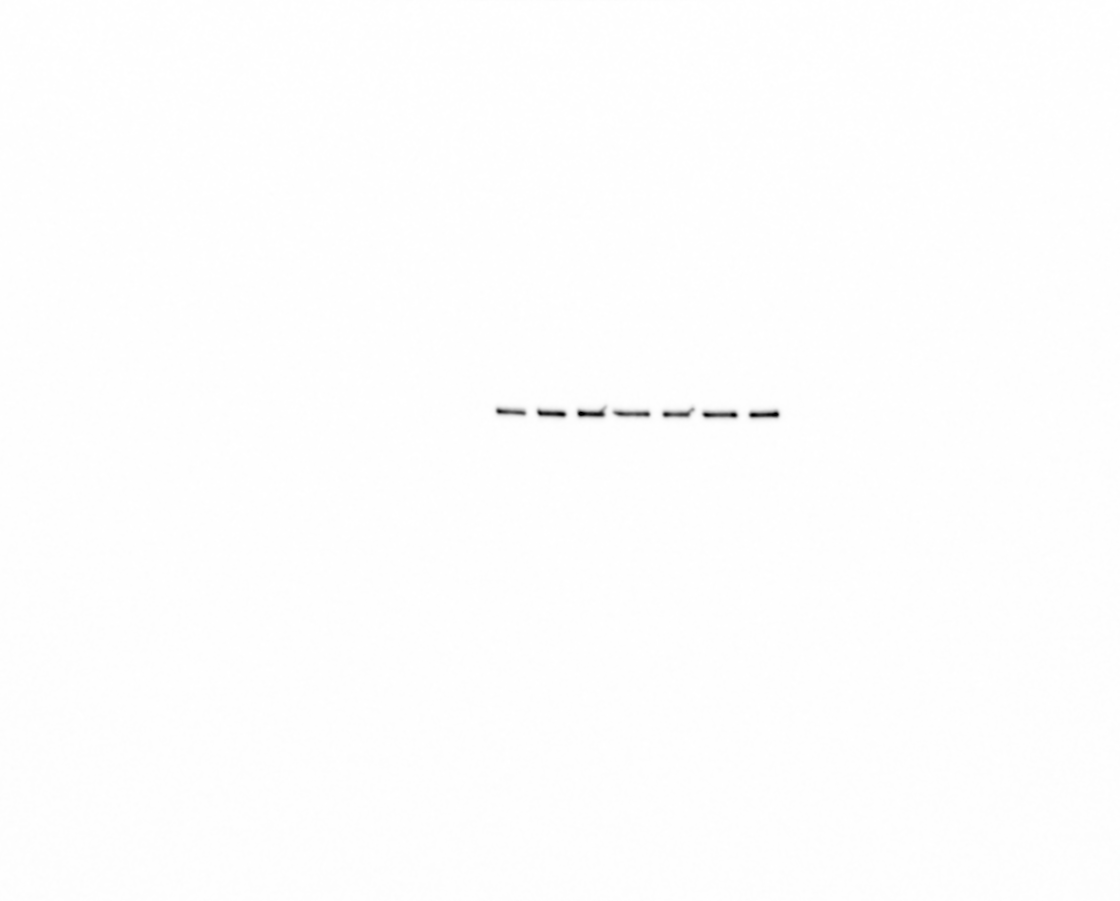

Supplement: Figure 6—source data 2. [file elife-100747-fig6-data2.zip › Figure 6 - Source Data 2 (original western files)/hsp90/23.01.25_12.05.21_S2_F09.tif]

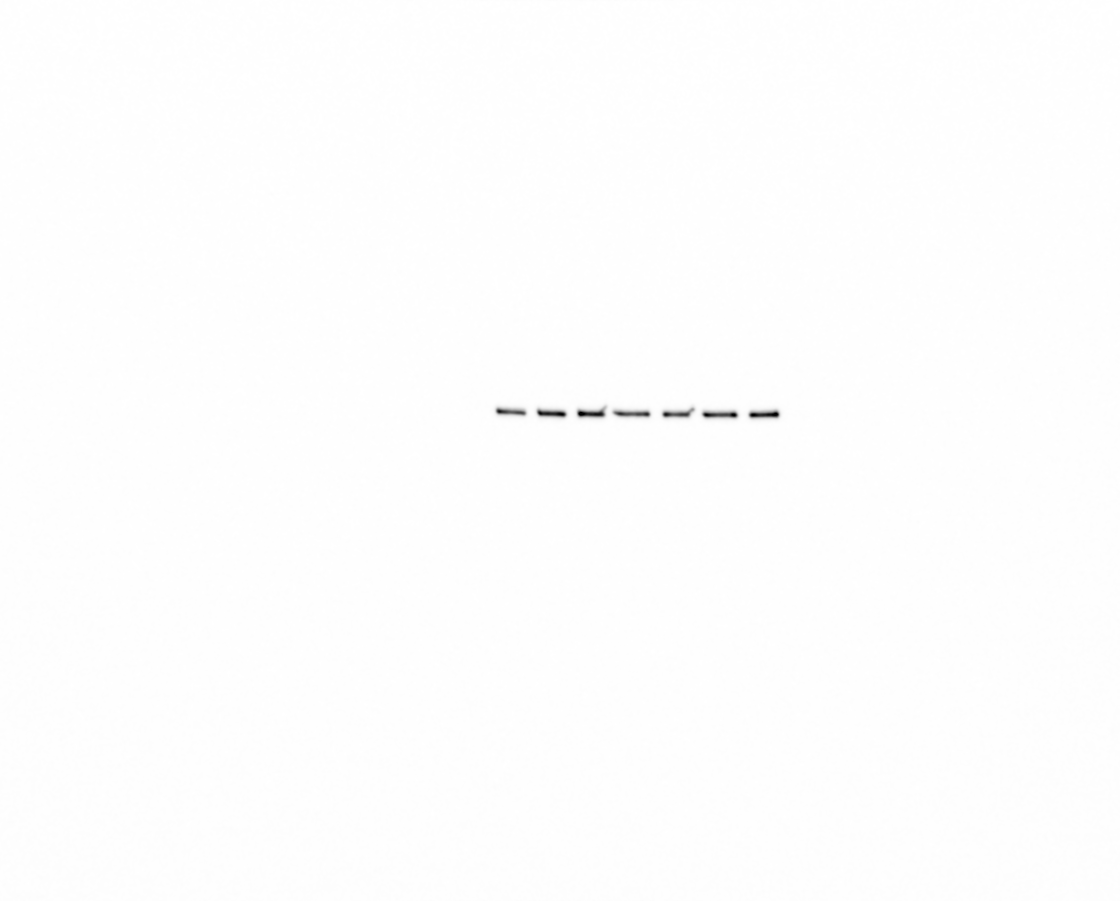

Supplement: Figure 6—source data 2. [file elife-100747-fig6-data2.zip › Figure 6 - Source Data 2 (original western files)/hsp90/23.01.25_12.05.21_S2_F10.tif]
